# Supplementary material for: Genome-wide Identification of WRKY transcription factor family members in sorghum (Sorghum bicolor (L.) moench)
Source: PLoS One. 2020 Aug 17;15(8):e0236651. doi: 10.1371/journal.pone.0236651 (PMC7430707; doi:10.1371/journal.pone.0236651)
Supplement: S1 File — (DOCX) [file pone.0236651.s001.docx]

**Supplemental File 1. Detail information on sequence similarity of putative paralogous pairs in a 100 kb region within an individual chromosome.**

| **Paralogous Pairs** | **Identities** | **Similarity** | **Gaps** | **Score** | **Distance (in Chromosome, kb)** | **Tandem duplicates** |
| --- | --- | --- | --- | --- | --- | --- |
| *SbWRKY3- SbWRKY4* | 72/180 (40.0%) | 96/180(53.3%) | 27/180 (15.7%) | 306.0 | 4.672 | N |
| *SbWRKY15- SbWRKY16* | 184/362 (50.8%) | 207/362 (57.2%) | 91/362 (25.1%) | 711.0 | 21.31 | N |
| *SbWRKY17-SbWRKY18* | 134/339 (39.5%) | 173/339 (51.0% | 67/339 (19.8%) | 495.5 | 15.37 | N |
| *SbWRKY23-SbWRKY24* | 93/336 (27.7%) | 127/336 (37.8.1%) | 113/336 (33.6%) | 249.0 | 13.516 | N |
| *SbWRKY24-SbWRKY25* | 70/258 (27.1.1%) | 110/258 (42.6%) | 39/258 (15.1%) | 238.5 | 3.437 | N |
| *SbWRKY27-SbWRKY28* | 70/212 (33.0.4%) | 95/212 (44.6%) | 42/212 (19.8%) | 247.5 | 14.406 | N |
| *SbWRKY29-SbWRKY30* | 78/284 (27.5%) | 117/284 (41.2%) | 44/284 (15.5%) | 214.5 | 2.266 | N |
| *SbWRKY34-SbWRKY35* | 49/163 (30.1%) | 68/163 (41.7%) | 38/163 (23.3%) | 174.5 | 25.966 | N |
| *SbWRKY37-SbWRKY38* | 79/250 (31.6%) | 108/250 (43.2%) | 54/250 (21.6%) | 254.5 | 22.947 | N |
| *SbWRKY38-SbWRKY39* | 81/266 (30.5%) | 106/266 (39.8%) | 70/266 (26.3%) | 236.5 | 29.379 | N |
| *SbWRKY39-SbWRKY40* | 98/327(30.0%) | 134/327(41.0%) | 86/327 (26.3%) | 292.5 | 20.018 | N |
| *SbWRKY40-SbWRKY41* | 62/190 (32.6%) | 89/190 (46.8%) | 37/190 (19.5%) | 235.0 | 35.007 | N |
| *SbWRKY51-SbWRKY52* | 277/373(74.3%) | 292/373(78.3%) | 49/373(13.1%) | 1286.0 | 51.743 | Y |
| *SbWRKY52-SbWRKY53* | 60/154(39.0%) | 87/154(56.5%) | 16/154(10.4%) | 273.0 | 30.790 | N |
| *SbWRKY53-SbWRKY54* | 44/124(35.5%) | 63/124(50.8%) | 12/124(9.7%) | 207.0 | 14.054 | N |
| *SbWRKY54-SbWRKY55* | 83/271 (30.6%) | 139/271 (51.3%) | 29/271 (10.7%) | 323.5 | 15.42 | N |
| *SbWRKY55-SbWRKY56* | 88/207(42.5%) | 118/207(57.0%) | 36/207 (17.4%) | 371.5 | 35.366 | N |
| *SbWRKY68-SbWRKY69* | 90/306 (29.4%) | 132/306 (43.1%) | 70/306 (2.7%) | 284.5 | 2.917 | N |
| *SbWRKY69-SbWRKY70* | 59/177(33.3%) | 90/177 (50.8%) | 31/177 (17.5%) | 248.5 | 5.26 | N |
| *SbWRKY70-SbWRKY71* | 47/129(36.4%) | 67/129 (51.9%) | 17/129(13.2%) | 188.0 | 8.756 | N |
| *SbWRKY84-SbWRKY85* | 121/443 (27.3%) | 160/443 (36.1%) | 145/443 (32.7%) | 306.0 | 13.945 | N |
| *SbWRKY87-SbWRKY88* | 234/263 (89.0%) | 234/263 (89.0%) | 29/263 (11.0%) | 1187.0 | 3.825 | Y |
| *SbWRKY88-SbWRKY89* | 76/259 (29.3%) | 110/259 (42.5%) | 46/259 (17.8%) | 214.0 | 9.681 | N |
|  |  |  |  |  |  |  |

Y: The paralogous pairs were tandem genes duplicates; N: The paralogous pairs were not tandem genes duplicates.
