# Supplementary material for: Genome-wide Identification of WRKY transcription factor family members in sorghum (Sorghum bicolor (L.) moench)
Source: PLoS One. 2020 Aug 17;15(8):e0236651. doi: 10.1371/journal.pone.0236651 (PMC7430707; doi:10.1371/journal.pone.0236651)
Supplement: S2 File — (DOCX) [file pone.0236651.s002.docx]

>SbWRKY1

MDIAEEAVAGTAAQGDLAEVVARAGAMAMAAAPSHHRRPPSPSPAAADHSHVMSRAAGQIMAIPPACYDEEQELRPAAACGDAVMFDVPSSMVVDPYHQLSSSAATAPPHGHGGYWLPPQQISQQACYGLDVAMGGAAAADADGDEPMMMRISPVTPPPPSHHQIMKSRKNEVKKVVCIPALPPASSRPGGGEVIPSDLWAWRKYGQKPIKGSPYPRGYYRCSSSKGCMARKQVERSRSDPNMLVITYTAEHNHPWPMQRNVLAGYSRPHTHMSNCKKKNSCRVEPTSSWPTSSSSSSSSKNANYFEHNVVPSSNIECQQMTNMMEDNAAGYVAYAIDTLDEEGVAMHQPINRNSIQPSDEVFAELEELEPSNNPVNANIYSRGVSYEWQKF*

>SbWRKY2

MKAMEVVEEANRAAVESCKKLVAVLSLSGADAFRPLPVAAETDEAVARFGKVVAVLSDRLGHARARVAGKRSPPAPPVDASCLLEYHPSLAVAPRHTTNGGHLLVSATSPPPPPPPPPTTTTSLLASMTMRSAAEPMTMRSQKAEVVAPAVLVSPPCASNVTLTPAPAKKFDRSMFLETSLLELNNSCSVPPSSSPAMAVQKSSPKVAAPNPCTSTPHIQLQPTTQFQPPPQQQAAKKQKSFQFDQTPSGEQFHIEVPVPLPRGAAPAAKEVISFSFDNNSVCTSSAATSFFTSISSQLISMSDAATSSAARPATAKKMCGKGVEDGGGGVRCHCPKKKKPREKRVVRVPAISDKNADIPADNYSWRKYGQKPIKGSPHPRGYYRCSSKKDCPARKHVERCRSDAAMLIVTYENDHNHAQPLDPSVLTAANAEP*

>SbWRKY3

MDDRRGRRDAMGQRPFASAAQGQERVFDGGGGGGGGGPGPAFGGEFDQGSSSLMALLGAGGAVSSQPPPPTWGVEEVTAAPAINLVPQSLFSMANYAPPPPSYQQPTSFAPSPLGGRVDPYPPYLVADQPPQWPPPRPAAADSSMPHSNFTVFFPRNPYDHDMQLRATALFGGSSGLHAHALPPPPPAIEQPAKDGYSWRKYGQKQLKDAESPRSYYKCTRDGCPVKKVVERSFDGFIKEITYKGRHNHPRPQERGLAGGGNDALAAAEEDVDGPSDDDDDDVDGAPGRAADGVVAGQRVVKKPKIILQTPSEVDLLDDGYRWRKYGQKVVKGNHRPRSYYKCIADKCNVRKQIERASTDPRCVLTTYTGRHNHDPPGQGNEAAAATVAAGGSSADPGPPSRNTASGSGAFQENWGARQLKEEC*

>SbWRKY4

MAGAAGDRSEDVGADWPFGGGAADAFTEYSSVFAELGWPGGLLASGELPVLDLPDPAAPLPSSSQLLSIEPSEDPAPARSGDAGASSSSSGDGDGAAPGNDDDDRKAAPAAEAAGRKPAAATAKKGQKRPRQPRFAFMTKSEIDHLEDGYRWRKYGQKAVKNSPFPRSYYRCTNSKCTVKKRVERSSTDPSVVITTYEGQHCHHIGPFQRGGGGGGGGAATARYHSAAAVALAEQMSSSSSFIPARQLYSLPPLHPPQSSLSSEAVVSSAATTSFHQHVNDGDELRQASYSSRVSMAQSPSTPSSVPPAISVEKAGLLDDMVPHGVRHGTP*

>SbWRKY5

MGPSSIQEMEEARRTAVQSCHRVLALLSNPHGQLVPSKDLMAATGEAVAKFGSLTAKLTNSNSNSNGNGLQLQGHARVRKIKKPLPIFDSNLFLESSAVAAAAAATVAKTPSPSPITGLQLFPRYHQMEGSSSKDPVRIPTQFPKRLLLENPAAGLEGLPSKAPPVQMVQPVSVAPPAGTPTPALPAAHLHFLQQNQSYQRFQLMHQMKIQNEMMKRSNLGDQGGSLSGGGGGKGVNLKFDSSNCTASSSRSFLSSLSMEGTLASLDGSRASRPFQLVSGSQTSSTPEMGLVHRKRCAGREDGGGRCTTGSRCHCSKKRKLRIRRSIKVPAISNKVADIPADEFSWRKYGQKPIKGSPHPRGYYKCSSVRGCPARKHVERCVDDPSMLIVTYEGDHNHNRVLAQPA*

>SbWRKY6

MDSSSQPGAIGSGGGGERNQREEDEAAAAAAAEAGYGRQLVMPEDGYEWKKYGQKFIKNIQKIRSYFRCRHKLCGAKKKVEWHPRDPSGDLRIVYEGAHQHGAPAAAAPPGPGGQHHGGGASDFNRYELGAQYFGGAGRSH*

>SbWRKY7

MEEVEEANREAVESCHRVLALLSQPHDPAQVRSIALGTDEACAKFRKVVSLLSNGGVGVGEAGPSGASGSGSHPRAKLVSRRQNPGFLTQKGFLDSNTPVVVLNSAHPSPASAQVYPRTAGALDAQGVHPLGGPPKLVQPLSAHFQFGNVSSRYQFQNQQQQQQKLQAEMFKRSNSGVNLKFESTSGTGTMSSARSFLSSLSMDGSVASLDGKSSSFHLIGGPAMSDPVNVQQAPRRRCTGRGEDGTGKCAVTGRCHCSKRSRKLRVKRSIKVPAISNKIADIPPDEYSWRKYGQKPIKGSPHPRGYYKCSSVRGCPARKHVERCVDDSSMLIVTYEGEHNHTRMPTQSAQV*

>SbWRKY8

MCDFFWLSPADQAGDLSDVVRASLQPPPPPPHHHRLPPPEEEEGLLLLQARVNFDGDDHDEARSQQLVHGNGSMRLMLGSNGSGGCACDHAAALCPQHHPEAERLIPQPPMSGPQPQLCAASSFVVERDDDDAPVLEEHVLDMATAPHPHPHTSAIKRRKSQTKKVVCIPAPVAAPPPGVGGRPSTSGEVVPSDLWAWRKYGQKPIKGSPYPRGYYRCSSSKGCSARKQVERSRTDPSMLVITYTSDHNHPWPTQRNALAGSTRPAFSSSSAARSHHHHHHHHHSAAAVPDTTLAHRHASNVAVADNATPGWSIISASVHHQLLKQEVVDVDNHPISKQPAQDAAADRDCLDMFADMDGALDVLCASNFHPKKQQQQVTAAQHLEKLPEEEDEHLLLGPDPFSFSFLDWVGASFGVGETAADNGDHS*

>SbWRKY9

MAAREASAAAPAPGPAGDGPSRPPRPTLALPPRSAVESLFASSGASSAGAAAETSPGPLTLAAALFPDGAPSPAFHGSFTQLLVGAIGSPAAVPSPPSPFAVPPGLSPATLLGSPGLFSPTGSFEMSHQQALAQVTAQAVHSQYNMINHADYAIPFSSTTTPALITAQHANSSANVTSAQEKPALPSHTGNSKIESNEVSQGLKPSAPTFDKPADDGYNWRKYGQKAVKGGEYPRSYYKCTHASCPVKKKVERSAEGYITQIIYRGQHNHQRPPKRRSKDGGGLLNEADDFHENEDTSTRSEPGSQDHSGKHEGSNDGIAGPSVSRRGEGHEQLSGSSDSDEERDDEQRAGNGDPGYANANRRHVPTPAQRIIVQTNSEVDLLDDGYRWRKYGQKVVKGNPHPRSYYKCTYQGCDVKKHIERSSQDPKAVITTYEGKHSHDVPAARNSSHAAANANCSSSTSVPHRVQSSASSSRRVADLQSTSSASSMLLKEENEIT*

>SbWRKY10

MASSGSDVPAARAAVAVNDLIQARDGAARLRAFLLQLDDQRAAWAQLQIDGVLTKLSSAMSALDVSDAAGSDDGARPRPQSGSSCGNKRKQSSSRRSQRPSDKKITANLEDGHVWRKYGQKEIQDSPYPRSYYRCTHKTDQGCSARRQVQRCETDTSKYVVTYYGEHTCRDPSTIPLIDHAAGALAELDRANNLISFGPSGTSNDANAAAAASNAGASSSQYLQAMGGSAAADQLSTSWCTSDDVFSSSAGSFMQVDQLIGAVVGGSAGVVTSAAAPDRGVVLGGVASGGRGTASFPTSPNSLGFVVGSLGSIGGGGEDDDDMFRLDP*

>SbWRKY11

MSPAPTSHHSQINSRKEKRMRKVDTFAPHNDGHQWRKYGEKKINNTNFPRYYYRCTYKDNMNCPATKQVQQKDHSDPPLYAVTYYNEHSCNSAFLPLSPSEFQLQTSSGKAVSICFDSSSGAAAPQEPPATAAATNASGGGGSPSSSAAAVAAARRGTPPEISNPPVLRRSETYPWGAGAAGVVEQKPASCSTECHDAFSGAAGAVPEEVVDAGRFGSIRFFHFL*

>SbWRKY12

MQQAQRRPVTGRAYTLSTQHMASSPSKPPAPGKTTKSSAAGQKRARQPRFAFMTKSDVDHLEDGYRWRKYGQKAVKNSPFPRSYYRCTNSKCTVKKRVERSSDDPSVVVTTYEGQHCHHTVAFPRAHHLHAALAAAGHHHMPFNFSAAAHHHHLYGTTSGVVTDHGHLPPLLLPTTPAPQHNALNDSDNNGSPLACRTSTTTSSLLRPLDCNHQELLLAAAASYPLSSSAAMSSMPVPSMSTTTTTSLPPPASSAVDKGLLDDMVPPAMRHG*

>SbWRKY13

MAPVQSALAATWTPSCRTSLIHCVKRSLLCGSAPVGSQRPPASPPTGAQPVPAAGDQQRQGELLNGPVLQNGVHDSYTWRKYGQKEILGARFPRSYYKCGRRPGCPAKKHVQQCDADPSKLEVTYLEAHTCDDPPPSSSHAVPDPTAGSDALLVPPVPTVPFPSAQCYGGGRPSPPPLPPYQVPYAATTLGSNVLTLTATGVLLPSASYDPVPDVTDCTPSLEQEQDHDLLHIPSPACSQSELLPMEAAKLSPHAHGLPLSLEHTLDCDFAVPEL*

>SbWRKY14

MSSMEAPSPTTSPLDGSILKLPGKLDRLLHQRHRYGCILPKGVEDEIPLIKGDLEEIMAMLSNLDDYEAMMVRCWRKEVRELSYDMEDYFIDQYEHTSAAESSLSLMMMTGSVSRRQRRNKSKTTVSRLGDKILIANNKMRESRVRAQELFQRYMSMYNRDAAAVPVSGSSSTSRCHHSNSTPRGGEEKNLDDHHAMKEALELSYDMENFIEHYGLLTPESLLAAAGSISVRSRRQVTHHQRPSRKSKTIILSRLGEKLRQRLCMANMIREFSLRAQEALGRYNTYKLDAISAGPAASSIRSCTTTDDGVYSGSSWKWNSTTSTCEDDVIVGISAAMENLQELLLTMHDEGHQQNLKVVSIVGSGGIGKTTVAMELYRKLGHQFDCRAFVRTSQEPDMRRIFVSMLSQIGPHQPPDNWTIHSLISTIRAHLRDKRYLIVVEDLCATSTWDIVKRALPDTNCCSRILTTTEIEDLALQSCDHDPKYVYKMKPLGEDDSRTLFFSSIFGPQHECHSELREISHAIISKCGGLPLAIVTVAGVLSSKPGLADQWDYVNKSIGYSLSINPTSEGMKQVLDLSFNILPQHLKACILYTGLYEEDIIIWKDDLVNQWIAEGFIEATEGQDKKEIARSFFDRLISRKLILPVCINKNGEVLSCVVHRMVLNLVIRYKSVEENFVTAIHHSQTITTLSDKVRRLSLQFGNAEDVILPINMRLSQVRTLVFWGVFKCSPTIVLFHLLQVLILHFWGDKDNINFDLTRISELFRLRYLKVTSNVTLELGNKIRGLQSLETLTIDARVNTVPSEIVYLPSLLHFSVLPETDLPNGIGHMTSLHTLGYFDLSSNSIENVQSLSMLTNLVDLKLTCSTGQPENMYNKMQFLLTSILGRLSNLKSLTLVPRASTNNAKSTDEAGATGMAISGGFSSLSSAPGLLQSLEVSPQICIFYWIPKWIGQLHKLRILKIGLTKIDRDDVDVIRGLTALAVLSLYSQTKPAARIVVGKTGFPVIKYFKFKCCDPLLKFEEGSMPNLCKLKLVFNANHAHQHITIPVGIRYLSNLKELSAKIGGAGSDESHRRAIELAFRDAVRVHARCERVSIQCVQQIIGGKDDQYSLGRVEDYGDEEDSDELVEMMPEHYGEAVDTDADNRSLLSELHILLLLRNKLDNQCDDITYSIPIVDVYSYDPWNLESSGSTHRGLLLFFRPLPEARGVVPVKTPSGYWKVTGLPGYIYSDERLAVGMKRTMEFYHDHLTSGTKTKWKIKEFTAFQHATAGEICTPMMPRSEMSLCQLYTELAANPDCSPAGVQYDEKLSVKQNKIPAEEPSVESDIVQTRKHADKRKRRVDETTKTVVTIASPDVNDGYTWRKYGSKQILGSNYPRDYYKCTQRRGCPARKHMQRRDGEPILYDVCYFGEHSCDLQQGHSSEQGSETNTISGGWEAAGLPLSVPHAHGISSVEAMISSGSLTARPNPGVSSLRPSSMVTSQVVMSNPDDDGYSWTKYGQKNILGAKHPISYYRCAHWIAQGCTATKRLHRKEDADTLGFDAIYYGQHTCDQIAHSTDNISSPLGCTTTGSTSKLGTDKIHPEQGGL*

>SbWRKY15

MAASVVDGNGGSGGLVVTELGHVKELARQLEAQLGGSSPDLCKHLASQISSIAERSISLLITTSSGLAGARKRSAVPFVKGTKKRKTMDKKRHEVRVSSAAGDHPADDGHSWRKYGQKDILGAKHPRGYYRCTHRHSQGCAATKQVQRTDEDPTSFDVVYLGDHTCVQSQWAAAAGQAAADALAPEYNGKPGTNLTVKTEGPTVEPAEQQVQGWDAPTPFCFSSTPATATASWCLVPELSPPFSAPSTSNNWGVSPATSDSNHVVSFPPFEVAGDDVQFGRFEEVMSAIDRADGDGFLDDLDIDVSSFLV*

>SbWRKY16

MATTEMEMGMGVVVGGGSNSNNGGGSSSSSGLVVTELSHIKELVRQLEVHLGGSPDLCKHLASQIFSLTERSIGLITSSNLDAGAARRKRSASDAAGLASPLSATPTSDVTDGPFKNNTKKRKVMGQRRERVSSAGGENPVDDGHSWRKYGQKEILGAKHPRGYYRCTHRHSQGCPATKQVQRTDEDATLYDVIYHGEHTCVHRPAVPAEHNADAHAHLQTLSAGLTVKTEGLPTAATPLYLSASTPLAPASTASENWGVVSPATSDSNHVAASYLPFDDAEWRGHAELQEVVSALVAASAPPPPPLPPAVDSLDDLLFDIDIASYFA*

>SbWRKY17

MLLMDSARRAGCSPSPVCLDLSVGLSPSSPGSSGPETTADTDDRLDRPAAGCRVASSLSDEQAKTLEAKLTQVSEENRRLTEMIAYLYASQVARQSSSSPDTTSRKRSRDSLEPPSNSSDGNANAKAEPGDHAAVESALSDEGTCRRIKVTRVCTRIDPADATLTVKDGYQWRKYGQKVTRDNPSPRAYFRCAYAPSCPVKKKVQRSAEDSSLLVATYEGEHNHPSPTRAGELPSSASATASGPVPCSISINSSGPTITLDLTKNGGGGGVRVLDAAEAPDLKKLCQEIASPDFRTALVEQMARSLTSDSKFTHALAAAILQQLPEY*

>SbWRKY18

MAMDSTNGECSSPTASAVGLLPLFGSSRPPPQAESLEEKLRRVSEENRRLAPALDAILSADRSNHPRALATSPPAQQQGNAALTTQAATGVVVTAEPRHKVRTVRARAEPADADANHLKDGYHWRKYGQKVTRDNPYPRAYFRCAYAPSCPVKKKVQRSADDNLMLVATYEGEHNHEQHAQSEYSYINDASTTSQQQQPQAGGSSSSTLPCSIISINSLGRTITLGLADQRRPGSSSNAEAAAVVVGEIVTPELRKVLVDELASLLKNDPEFIESLATAVADRVMERIPAAGHIL*

>SbWRKY19

MTNVIRITRHSFQLREISGMAGASNHGSLMDEWLPPPTPSPRTLMSSFLNEEFSSEPFSGFFSEHGTNKPHDQSEKSREVVNSSEEVPAHAVNDPFQKGFSLKPNLFSANHKSNSNGGLAERRAARAGFSVAKIDTSRVGSSAVIRSPVSIPPGLSPTTLLESPVFLYNKMAQPSPTTGTLPFLMATNDKSTIPPAAKITEDSPFDNDVFSFQPHLGSEATGFSTAEKDYGAYQQKQSLSNIHQQESSLQSSFTAVKDNTSATIVKAKTSSSMFSDSHYSADQQQADETNIKVQGKGVEARSAAFLPVSAHSDASLLESQDAVDVSSTLSNEEERATHGTVSIECDGDEDETESKRRKLELDALGATAITTTSTTSTIDMGPGASRAVREPRVVVQTTSEVDILDDGYRWRKYGQKVVKGNPNPRSYYKCTHPGCSVRKHVERASHDLKSVITTYEGKHNHEVPAARNSGQGSSGSGSAPSAPQAGGSHRRQESAQASFAHFGTTSPFGSFGLPPSRQLGPTTGNFRFGMVPPGMTIPMPSLGSLAPTKMVGSSSSMQGYPGLMMPGEPKVEPVSQPLFPMANASPPAYQQMLSRPPFGHQM*

>SbWRKY20

MADSPNPSSGDLPAGAGGSTEKPVLADRRVAALAGAGARYKAMSPARLPISREPCLTIPAGFSPGALLESPVLLNNFKVEPSPTTGTLSMAAIINKSTHRDILPSPRDNSAGSGQEDGGSRDFEFKPHLNSQLAAPAVNNQNRHDTPMQNHSSNHASPSSNLMTENKPLCSRESSHTANVSSAPNQPVSIVCPSDNMPAEVGTSEMHQINSSENAAQEAQTENVAEKSAEDGYNWRKYGQKHVKGSENPRSYYKCTHPNCEVKKLLERSLDGQITEVVYKGRHNHPKPQPNRRLAAGAVPSSQGEERYDGVAPIEDKPSNIYSNLCNQVHSAGMIDTVPGPASDDDVDAGGGRPYPGDDANDDDDLDSKRRKMESAGIDAALMGKPNREPRVVVQTVSEVDILDDGYRWRKYGQKVVKGNPNPRSYYKCTHTGCPVRKHVERASHDPKSVITTYEGKHNHEVPASRNASHEMSTAPMKPVVHPINSNMPGLGGMMRACDARAFTNQYSQAAESDTISLDLGVGISPNHSDATNQMQPSVPEPMQYQMQHMAPVYGSMGLPGMPVAAVPGNAASSIYGSRDEKGNEGFTFKATPLDRSANFYSSAGNLVMGP*

>SbWRKY21

MASSAGGGGRRSPPATAAPRMVMDRLMEVHEGATKLQTMLQESPTPSIAAAAGTTSELRLTIDRMLSSLSSAMSAWNTTGAAQGPGQGRRRRRGEAAAGSGPQRRSSTRRRSHSPFVKMVTTSKLDDGKAWRKYGQKRIHESPNPRSYYRCTHRPEQRCMATRQVQASDANPSEFIISYYGQHTCQDPSTIPLVIPDTAPPPDCANLISFGGCTTIAAGASSSSTTTTAVPPQQALSFDPTTTPMLMLSRFGYSSSLPAAQAQQDYRCGSEEVLSSRSSPAAQLATMVVGSAGTMASSSTVGSAPAEYWPGGTSGMACGPGSFPSSPSSLGFMTGSPFGSFGNAGDDDLFGFDP*

>SbWRKY22

MDKAHLGVGGGLLALDASPRPLGFLSPTAFHRARTTAMEAADDGNGTPPPGSRVRRSVEVDFFSDQKIAADAANNNTCGRTTVSPGSGSGASCLAIKKEDLTINLLPGTGSNANDDEAATRLRLLDQDKQSRNTNEMQAELARMNDENQRLRGMLTQVTSSYQALQMHLVALMQARAGGQAQLMLPPVAQALPPTTDGAAAAVMPLPRQFLGLGPAAAAEETSNSSTEVGSPRRSSSTGGNRRAERGDSPDASTRQQQVAQQQQEASMRKARVSVRARSEAPIIADGCQWRKYGQKMAKGNPCPRAYYRCTMANGCPVRKQVQRCADDRSILITTYEGTHNHPLPPAAMAMASTTSAAASMLLSGSMPSGDMMTSNFLARAVLPCSSSMATISASAPFPTVTLDLTHGPPAAARPQPHFQVPLPPHQQVQQQHHHLQAAALYNAHQSSSKFSGLHMSSSSTSDNNNNVGTSSRAAVAAADAPPHMDTVTAAAAAITADPNFTVALAAAITSIIGGGGGHPIPIAIHHGQGQGQEQGQQQAPTSNSNANNNNNAVVTSSSNNTATSNSETQ*

>SbWRKY23

MAASLGLAHDASCYAAYPPAAAAASSYFPSPPPPGDLVAEFPPTAAATAMADDYYYYYFQFGEEMGGARAPGCGGGYCSPPAPAFDNGMSLLSYGGVDGDGRRPMSGPAAGTGGNGGGGRPPASRIGFRTRSEVDVLDDGFKWRKYGKKAVKSSPNPRNYYRCSSEGCGVKKRVERDSDDPRYVITTYDGVHNHAAPGAAYLCPPPPRGATATAAAPCFSSPCSGSASAALVAAPSWSGAFDAWEAQLAAAAAHSSESSY*

>SbWRKY24

MEVAVERPPPAPQVKAEEKRPDAKPEIAARPPMVGSALPIVFESFPSTQRDAAGGINVKQEERRLEAARAEMGEVREENERLKSMLSRIVSQYQSLQMHFLDVVKVQEQASSAAKVAEKKLPVAPAPAPNPGTDDDGPDDLVSLSLGTRANSGGAPRRKGHERSSSSSGTAETTTAADADDQGHHQLSLGLGFARGNGLPSSTTTATDDDKASHASTAPVLNLTSDSSGSADDNDDAKPALAAAGTARKSPSAGAGAGDRSADDEVQQQAKKARVSVRVKCDTPTMPDGCQWRKYGQKISKGNPCPRAYYRCTVAAHCPVRKQVQRCAEDTSILITTYEGAHNHPLTPAATAMASTTSAAVAMLTSGSTTSAASASLVHGHGHPLPAAAGLFGPTTMVSTAASCPTITLDLTSPAAPHSLMHSSPYAAAAAAAAAAGFESKAFPAAWSNGYLAYGGAHPSYYSKSSTSPALGHLFGGSLGVPSRPEQLYAQSYLQRASSLGGGHGAVAPAAVTDTLAKAITSDPSFQSALAAAITSVMGRGGAAAAQK*

>SbWRKY25

MFPSPGRTVMALGHGGQHMTSSSTAGAAGGMAAASSSSTPTITFAFQPSPPPTSGLALAHHGVLGYGSSSLLLDHHHHPTTTTTSSAASSSHAASSITLHHHLHGHAAAAAPHASLSPPTRASPPPHPWSTTTTACEEAGGPAPAHDRQAGQQGGRPPRGKGAAAVISEGSAAAALGVGAVRMKKAGGGGGGGGGKARRKVREPRFCFKTMSDVDVLDDGYKWRKYGQKVVKNTQHPRSYYRCTQDNCRVKKRVERLAEDPRMVITTYEGRHVHSPSRDDDDDAARANAEMSFIW*

>SbWRKY26

METRPERHRHDHVHEQQAQEGEEDDAAAAVMEHGAALSLQRGATLFGRRRQHDEEADRRRRGEIREVDFFSRDSGARGQDDGGGRGVPGGGRDDVNIGLDLLTTATAATTSAAGEEMMAVKNQKIEASAVEVELRRVVEENRRLRGMLEELNRSYGALYQQLLQVTQHRQHPADLMINRSSLAHTHLTTTAASHNTSSTRQLLEARASSTAMAQPHAVAAGGDDEASDGAEEASPSLSNGGNNNDDADGKRKTSPDRTAPPRENGGEQASSELPGRKARVSVRARSEAPMISDGCQWRKYGQKMAKGNPCPRAYYRCTMAVACPVRKQVQRCAEDKTILVTTYEGHHNHPLPPAATTMANTTSAAAAMLLSGPATSRDGAAAALLGHPALFHHSSSIPYASTMATLSASAPFPTITLDLTQAPGGVAGSGGGGLLPHGLGLHRPPGGIHPVTAVPAMPFPVPSPLASMFLPQRAPTGPPMPTGLQVARQQQSVMMETVTAAIAADPNFTTALAAAISSVMAGGAAHQAQPTPRGSNIIGIAAGDQANGSAGAAAIAGPTAAGAHAASAGSPRFATQSCTTST*

>SbWRKY27

MSSSYSSLLSPSRAHADDHRVLLGGDADDDDMAAVSSYLSLDDIVDDVVGGEWYRPLAEESSSAAAAELQPEPLLFATLQAEDGYCVSGSGGEQSSAALANDNHDRIDLTQDGGSRRLLRSEHGKIAFKTRSDVDVLDDGYRWRKYGKKLVKNSPNPRNYYRCSSEGCRVKKRVERERDDARFVITTYDGVHNHPAAAPPRSPAYRLGEPPHGHHV*

>SbWRKY28

MELKTFVFKELMRGAADKMKLGDEPAAGPFLSLSLGPANAIGASLRTRGGEAINASQAPPHAASNADDGIGLALGLRCDSDGGGEPVLAAVVGSAAGTKRQRVAILSDDSGGNRGSNKALRLPALLAPPQQQRPAGRVTFRARCSAATVNDGCQWRKYGQKVAKGNPCPRAYYRCTGAPDCPVRKKVQRCAHDAAVLVTTYDGAHNHPLSPYAAAMASAMLASSSSSSSSATAASCDDDAPRLAFPISVLPPAPQRYSCSRDVAIGAGPPPAAPAATSHGDLVPMANIMQKAVGDPNFRAAVMAAVASYVGEQCGGRNISTTSSPCHLPANN*

>SbWRKY29

MRACGGRALLDCQCQKSTHPFLLLQSPAYGQERSSTHASPLCSSKAPARRARSWFMRGVLLRMAEHFNDWDLQAVVRSCGSVAAHPDPAAPRAEPDAAPPEPTTTTPVAAPPERPPGARATPPTPVAVPVPVPVRGQEQRAPPPVAAKAAALLYDLEYLDLDHKPFLMPVVAPSPRAGDNGRGEREVMISFPAGAASTSGMQQRASPPGRKPGARTPRPKRSKKSQLKKVVREMPVADGGSSSSDPWAWRKYGQKPIKGSPYPRGYYKCSSMKGCMARKLVERSPAKPGVLIVTYMAEHCHPVPTQLNALAGTTRHKTSSSGAASSPKSHEQGQAVEKAAGRGAGDREHGNNETSSSMAGEFGGEEIAVAIDDDEFWPAGMDLDELLAPVDDDFDFEHVVEEEDGVLGRRLSL*

>SbWRKY30

MSSGGGGSSGGGGDHHGVYHQHGHGHLARADAGAEYVFHSNDMESFFFNQPAASAGVDGSGSRTTGAADELMPPYSSITDYLQGFLQDPSGLARHLDAPCLPAEDAPLKHELSVDVSHDSQGTSGAPGGEGAAMHTPNSSVSLSSSDREGEGGQQPRRCKKGRPKAEDAEGDEKEQEDGENSSKANKSKKKAEKRQRQPRVAFLTKSEVDHLEDGYRWRKYGQKAVKNSPYPRSYYRCTTPKCGVKKRVERSYQDPSTVITTYEGQHTHHSPASLRAGGAHLFMSNAHGGLPPHLMPSSFGRPDLMSMMHPAMGANPSMFLPSMPPPHMSTPSPAPPLQQHHFTDYALLQDLFPSTMPNNP*

>SbWRKY31

MEGGVPEEKCALVAELVQVLEMARQLETHMAVVVQQQQQGGGGAGGGADQRYRALVSTMRASIDRAVHMAVSCCAEGRPGTGQLPESPPSGGDGSSPRSGGSDHAGELRGRGNAAAGQCKKRKTLPKWSTQVRVSAVQDVSPLDDGLSWRKYGQKDILGAKYPRSYFRCTHRHTQSCQASKQVQRTDGDPLLFDVVYHGAHTCAQGAAAHPSNQQPAVQEQTTSPSPGFEAGTAVLPFSLRPASNKPTTGADAAATSSRFVTTGCVSVTASPFLSPATPESQLVSSSSSGYAVGGGGGVAMAGVRNVPDVELASTTNSPMAMGEMDFMFPLDAADFLELGNPASYF*

>SbWRKY32

MSSGDFHFHDELASLFAQRPAAPGEMMMAQQQQQQQQAPASWFADYLHGAGVPGMGGMDYDLLCRALDLPLPGDDVVKRELLVVDTGGGGGGLGFAAPTPSGGGTAPVTPNTTSSMSSSSSEAAGGGAAGGGGGGSFGGAGEEDSPHQGRCKKEEGDGEESKALDKGEEDADKGKKGSPAAAKGKGKGEKRQRQPRFAFMTKSEVDHLEDGYRWRKYGQKAVKNSPYPRSYYRCTTQKCPVKKRVERSYQDPAVVITTYEGKHTHPIPATLRGSTHLLAAQLHGGHHHHHHLGGAFPPPAPLPQQMAGAPFGRAGGGGGGVIDMLGLLPPRNNNHAAMPPAIGLASSRGMSGGGPMSTVAGATAATAAATTTSSSSPPSLQMQHFMAQDFGLLQDMLPSFVHGNGGNVQP*

>SbWRKY33

MHMALSSRSSFAAADVLLPAAMAYRQPCSGGGGGPATSSYFGSRPAAPFFPFGTAAQLDVFECLSDEGGAVPAPPAAVPGAFATPPPPLPLMPAERVVPDAAAGYSSHARSAAAAAAGEGPPRRTDRIAFRVRSDDEEVLDDGYKWRKYGKKSVKNSPNPRNYYRCSTEGCSVKKRVERDKDDQRYVVTMYEGVHNHVSPGTIYYATQDAASGRFFVAGMHQPGH*

>SbWRKY34

MDAEWSDGAAAASPPTVSGGESKPGAAGAVSSSADCPGSPPVSPAPPSTTSPAAAAAAAGSGRRRSANKRVVTVPLADVSGPRPKGVGEGNTPTDSWAWRKYGQKPIKGSPFPRAYYRCSSSKGCPARKQVERSRAEPDKVIVTYSFEHSHSDAVARAQQNRQQASKPKAVQRQPVPPEPAAESPSSGSYDVAAATVCGAGAPAAAAAGTEVGGAASVEVRDEFRWLYDGVSVTSSASPSDVEAADEMLYGAMFFGAAAAPPAAPLPDEFVGDVGGLFDYGEGGGEEDAMFAGLGELPECAMVFRRHAGDGLSVAGGVK*

>SbWRKY35

MENQHLQGDESSSSHALPSFPYFAVPSPPYAPPPPSEDQHSTLITALQQQPSSSACNNDDLPPLGLGPDQLAAVAAPMILPPMVDWSALLQQASLMGPQLVPGLLQQVPPLEPLDQSGENDGGDAGSSSSSKEKVVAKGGGGAGRSGKKKASRPRFAFQTRSVNDILDDGYRWRKYGQKAVKNSEHPRSYYRCTHHTCNVKKQVQRLAKDTSIVVTTYEGVHNHPCEKLMEALSPILKQLQFLSQF*

>SbWRKY36

MSSAEDGYCSSDSPRAESPDEPLLPAAVADADAESPRAAGSGMNKRERDLSDLPASPSSPLPPAKRSRRSVEKRVVSVPLAECGDRPRGATGEGPPPSDSWAWRKYGQKPIKGSPYPRGYYRCSSSKGCPARKQVERSRADPTVLLVTYTFDHNHEAPQPKSSSCHQQGKPSTRPPAPKPEPVVEQDELGPEHELAETEVPEQQEPVEEEQEQKVVPGLAGPEAEAEAEPTATVAPAAAEEDESFDFGWFDQYPTWHRSALYAPLLPPEEWERELQGEDALFAGLGELPECAVVFGRRRELSLATTAPCS*

>SbWRKY37

MAKRDDYMDSSCGCSNGTPKRLVQDCSSYAQAHAKKKVRISTRTEYTYAPYHDGYQWRKYGQKMIRGNAYPRCYYRCTYHQDHGCPATKHVEQTNSQDPPLFRVIYTNEHTCCSTHVSDYMASSIHIQQIADASLRKVEVEIPSLTHCFDGHGLIKEENDAIISSLTAISDYDVATSDGGHAAVQEDTPARMSRSSNEASPSISPVLLPASDNLKTDFIEQLEPQWFEPLDLGWFI*

>SbWRKY38

MKREQSFEFGDPSAQDAMGSAASESSYSPPGAVFGLSPPESASPRSGRHNRRRDRPSWVRLTYTPYFDGHLWRKYGQKKIKDAEYPRLYFRCSYREDRQCLASKLLQQKNGDDPPLYEVTYTYEHTCGAPPVSFPDIVAEPPPAAREGLVLRFDSPGGHGGHARMQQNGHCQQSTSRSPFMMLSFGSRSQTHDQHPAVFRSDLEAGSSSFPTEAPPAPPPPANGDGGDMLSTLNSFAYDFDNQMHFGDHTYLPHNNSNYDYDDY*

>SbWRKY39

MAFDRDAKLFDVLANGYHLNTQLQALLVGRPLNSIGQQEAMAFSQELSRVFKLSMSMLNCNTVTRLRTAPEIRAGDSSGVIIQAVKDKRARSDNGEVVTPVKKSREDGVTRKEITASPYKDGYEWRKYGQKNIQNCNYVRYYFRCSRDRRCEAKKKVQQQDDGSGRGQPLSPPMFEVTYVNEHTCHLLRAIANDGDAARMAASPRTTNRWSRVLGVVDTARDDDHGGGVLFNDLSSSFPRIGGGGGDDAQENETIVSCLATVISGGAAPSPPPWPPAAAEAGASDHPAAASSYGVPPPMQASGHSASVAEDGGGTTTTTTTMMIDDMDTDFCWDPSSFCAVGEGDQLMMDHRDMHVDVARLADTVWPRHTSAGASWR*

>SbWRKY40

MQTQSRLGGSGSSSSASEDEHEAVIRELTRGHELTAQLRAEALRALRGQGQAEATAAFILQEVSRAFTVCLSIMSSPARAPPSTSQPPPPTMEIMAPALLAPPRRSRDDSMPREQRRTSSPHCDGYQWRKYGQKRITKTQFPRCYFKCSFHRERNCRATKQVQQCSNDDPPQYVVIYFNEHTCDDTAAWDPTPTVPLDDLSSGLLVARQAGSLLLDERGVQEEHERRLLVSSLACVLGAQQQQSPAGSGTTAAVNVGHEQDQEPPPPRARTRDDAPAPAPAPAPAGVDDDAPGEMPRSIIDVDVAGLDVMDYYVTDALCFRDSYDLPSDGFSF*

>SbWRKY41

MTLDTPAAVVLELMTMGQQSAAHLRDLLRASSPAASSPHQELAAEILRCCGRVIDALRATTNGRKRKAAAAEYHQDAAATGGATWSPPPPPPGPPLKRRARGAEATREVTSGTTVDGFIWRKYGQKDINGHKHPRLYYRCAHKDQGCNATRRVQQTQDQPAAYEIAYYGDHTCKGAATAWQQLGAAPAVVDFGSNSWGSADANNNGGSPAASMSQGGWSPSASSEVGFDFEALHEWHDTAAPDPVMEFLDGCFGWESVLQDSSDFGGLLLHDIATFQ*

>SbWRKY42

MTTSSSGSIEAPAASSRPGSFSFASTSFTDMLGGSADAAAGGASRYKAMTPPSLPLTPSSFFSNIPGGLNPADFLDSPALLSSSIFPSPTTNAFASQQFSWLTTPGAEQGVKEEQRQSYPDFSFQTAPTTQEAVRTTTTFQPPIPAAPLGEEAYRSQQQQQQPWGYQQQQQQPAGMDAGSSQAAYGGAFQAGSSDAGAMAPHVPASGGYSHQAQRRSSDDGYNWRKYGQKQVKGSENPRSYYKCTFPSCPTKKKVERSLDGQITEIVYKGTHNHAKPQNTRRNSGAAAQLLQGGDASEHSFGGTPVATPENSSASFGDDEVGVGSPRAANAAGDEFDEDEPDSKRWRKDGDGEGISMAGNRTVREPRVVVQTMSDIDILDDGYRWRKYGQKVVKGNPNPRSYYKCTTAGCPVRKHVERASHDLRAVITTYEGKHNHDVPAARGSAALYRPAPPPPPSADNAGHYLAAQPGMAYQTGQQQYGFGGQGSFGLSGGAGAPAQSSGSFAFSSAGFDNPMGSYMSQHQQQQRQNDAMHASRAKEEPRDDMSFFPQSMLYTD*

>SbWRKY43

MGDGDYGLHPEPGAADVAVWPGELDEQLITELLSDDSLLLGTLPPPQQVPAGDDPEEQHCSRDTGGASSAPAAPCISGGGTAAEHRELLPQPEAVSRALCSVYTGPTIRDIEKALSTTRPYPWSWSSSRYSPTMHLGRLGALSRAPEKYTTKVRSCGGKTPSDGYKWRKYGQKSIKNNPHPRSYYKCTSSRCGAKKHVEKSTEDPEMLMVTYEGPHLHGPQPLFPRRQWLSIDLSGAAAAAAAASKTKQQQARVSSSSSPAASAALATSDDGGGGWPPSQQTTTRGRDTEAARGGPTAAAGETATPGPPQIGRAGDAVSTQPRLVLTADSCDDGSAASVPPPWASAAFPHCDSPPMMTWSCPDFPFAWSPDEAPLLL*

>SbWRKY44

MEEDLLLLEASVAVPPLAESINKYCWPNGSDFATTEELMMMSDLVDEAALSSSPVQQQEEEEPRRQRESMLNKLISTVYSGPTISDIESALSFTGADQAAAVDAHIYNSAGPVVFSPEKVLSKMENKYTLKIKTCGNGLAEDGYKWRKYGQKSIKNSPNPRSYYRCTNPRCNAKKQVERSTEEADTLVVTYEGLHLHYTYSHFLQPQPQPQPQQPKKPKLGGPPPQPQPIIMLEDLDGPAQQDITTCPLDATAMAPAPPPAALCYLDDMFQKPAFFEEELQQQHITNGGLLEDMVPLLVRRPCSSTGATTTTTGSSTTSSSPQLAPSPDLSTSSSVSWNPTSPYIDMAILSNIF*

>SbWRKY45

MDPWISSQPSLSLDLHVGLPPLSLHQAPVAAVALARPKVLVEENFLPPKKEPEVAALETELHRMSEENRRLTEALAAVASKYEALRSQYTEMVAAAAAGTNNNPSSTSEGGSVSPSRKRKSESMDTAPAPPAAAQQQQQHGTHLHQQQHPGLAAPDQNECTSGEPCKRIREECKPKVSKLYVHADPADLSLVVKDGYQWRKYGQKVTKDNPCPRAYFRCSFAPACPVKKKVQRSADDTSILVATYEGEHNHGQPPPAAPSQAAHDGSAAPGATKNAAAVAKPPSPPRPAAPAPAPAPHRPQLQLLQQEGVAMNVEQPVAAAASEMIRRNLAEQMAMTLTRDPSFKAALVTALSGRILELSPTKD*

>SbWRKY46

MEEERCFNNWDLDAVVRLGCRRRLSPPGQPDPFASFLPPPPPSPPHKEKPVVPAPAAKEPEPYAAWRFPDLGAAGGGQDGDELLRALLAAPPPPPPQPLPTPTPLPPPPPQQQRQPAVAAVDVPLPQARPAPARAQPSGRQVPGGVPRSKRRKNQVKKVVCHVPADGSSSDVWAWRKYGQKPIKGSPYPRGYYRCSSSKGCAARKQVERSRADPNTFILTYTGEHNHAAPTHRNSLAGTTRHKFPASATPQPPPPSVVVGGAGAGAGAAPGDAQHQHQQPSPSPTSTSTAGLSPTTPLRTPSMEEDDEEEEDELLVEDMEMAGEDELLFLNPDADAGAPMSSLFDVVDEPFLSSPWVTATSSAGEPATGAAGAGS*

>SbWRKY47

MITIDDLLRSCGGDSGSIPVPSSDDGRQMLAMGDHHQLTVSRIRTAVSMLNRRTGHARFRRGPVVAEQHASARPGVVALDFVNKACEARFSASASGTSSSLPSSLTVTAGEGSVSNGRAQPQGQYPFQPVSGGGGSDGHSARKPLPLAVSMQQQQHASPDHSAPAGTALKNGKCHDRARSENDAGGKTHGHRCHCSKKRKSRVKRTVRVPAISSRNADIPADDYSWRKYGQKPIKGSPYPRGYYKCSTVRGCPARKHVERDPGEPAMLIVTYEGDHRHDDQQQERSAGGAQTDHTTTSS*

>SbWRKY48

MCDYFLQRMEGDQHHQAGDLTDVVRAGGAMHQQGAIAELSSSTATGWQLPAEPAPAGPGLFLPPQPSSSDGGDGFADAFAGLPDPFASDFVRASSSSGGGPVPAADFFDFEAPAAAVGGGARRGGGGVLVDSGGGGVVVERGVPQMPALSPREIRPYPVTMIGGDTVKIGVPTMMPGLAVGPACAFDAIAGLQMPSPHGGGIKRRKNQARKVVCIPAPAAAGGRTTGEVVPSDLWAWRKYGQKPIKGSPYPRGYYRCSSSKGCPARKQVERSRTDPSLLVITYNSEHNHPWPTQRNALAGSTRSHHAKNSKNNPSQHNLQKPDLKAEPEHHQASAAVVPTGCATTATTAATSTTTTATTSTTSNSTPPPATMAVKEEAMVGSEMEKGMDHDASVLLDHGDLMQQMFSQSYYRPMIPEAGGGGGGHHADDFFADLAELESDPMSLIFPGGGDPGKEKEMMPNKSLGADPLFGMLDWGATNNGVATSAGSSFEQDESGW*

>SbWRKY49

MQAYMEGGQLSACLPGFLVPDHYAFPLPLPLQLPSSQNKLFQMPFVVDQEAETENHGGGGMLSSDHCGLYPLPALPFGSCSGAAGAATACGGKPTAGFMPSAIVAEEVCTSVTTKLGCNDSNGTWWKGSAATTIAERGKMKVRRKMREPRFCFQTRSDVDVLDDGYKWRKYGQKVVKNSLHPRSYFRCTHSNCRVKKRVERLSTDCRMVMTTYEGRHTHSPCSDDASSADHTDCFTSF*

>SbWRKY50

MSSKKKRAAIDLSLEVERSDEDHGSGGRGKGDRRRGKDDGEVDKKEEQFKEQGEEPKEETGEEEKVVVEVVVDQGGDGTKEIKYRTQQGEEMEDDKQSPADAHGDGESDGAEARAQDKHVVEAAGNGDGGDDSYTTMVQDEVSAMQEEMEKMKEENRMLRRVVDRTVRDYYELQKKVEACYQQQQADEPKEPEVFLSLGATAAGTGGAFPEPKRKERQAARRPSVGSDDTDDDDAKEDLGLSLSLRASSYEEEKLEAGHDDVEGASVVGADDGKAKGYTLLESSKLGAPAAGITSQSVNPANRKTRVSVRVRCQGPTMNDGCQWRKYGQKVAKGNPCPRAYYRCTVAPGCPVRKQVQRCLEDMSILVTTYEGTHNHPLPVGATAMASTTSAAATFMLLSSTTSSSSISEAGGGSAAPPYLSTPYLLNSTSHHSAASPLLSAPPSSSMPSSTPGAASGVQHLNMFGHSSSMLAQQAPHFGSNSKYPWSSDPLQGMGGGGGLPAGSKRPFWSTGGDEKTATLPDNVGAVMADPSKFSVAIAAAINSYMGKDGQVVGGKDGESSSSKSSNKWGVVESLPPP*

>SbWRKY51

MACVADREDAVREVTQVYELIKLQQPLLLLHSPQHPPPPPSTTCQLAQSLLAKALRALNVALSVMKQQPAPVTPISVIKAEPHQLSPPCSLASAESQAAIVLSTATRGAKRRRSTEGKKKNTSSSWATVTAVPYDDGYEWRKYGEKKINGTLFTRSYFRCTYKDDAGCLATKHVQQMDNNSDPPMFHVTYNNDHTCNTSARANTGSSSNLAALLAGCCNMKQEPTGHAAAAAAATMDMKQEVQEPPLLLPALVDLQPSACFHHEQIPRCQEPLFPVSMEQQFVCGPFRDDDSEIPSATGSCISGETSWDGYSGHMAAAEDDPLLDLERFLFMDY*

>SbWRKY52

MACVGEREAAVREVAQVYELIKLQQPLLLLHSPQHPPPPSTAKLAQSLLAKALRALNVALSVMKQQQPVVVVKAEPHQLSPPSPASANSQVAIVPSTATRGAKRRRSSVAIMEGKKKTSSSSWATVTAVPYDDGYEWRKYGEKKINGTLFTRSYFRCTYKDDAGCLATKHVQQRDDNSDLPMFHVTYNNDHTCNRAKAAGIANNGSSSNNLAALLAGCCSNGSGSGSGKGLTTMTTTNARPTEHAAAAAAMNMMKQEPPLLLPALIDLQQPSACFPNEQIPQCQKEPLFPTSMEQQFVCGALRDHDSPVDGDIPSATGSCNSGETSWWDGYSGDMAAQMAAEDDPLHDLDRFLQCDSFMDY*

>SbWRKY53

MALDSVPPYPCDLGSSRAAAAARTQQRIRKDERTWTSDTYAPYDDGHQWRKYGEKKLSNSHFPRFYYRCTYKNDMKCPATKQVQQKDTSDPPLFSVTYFNHHTCSTSSSAIGSARDITSQSSSKKAVSICFSPHTASEQPSFLTSPAMPQSTIMHPYSANQQSDRSAYAYQQLQWTGSVPSHASNGPAKMEVDDSAQPSPSSSSTSALSRTLLPIGQSRCIEYFHFL*

>SbWRKY54

MMNILESSNLGGYKEVINEVEHQRALMMNLHDLVLPILDPCSGQAKLIQQLFEEVFSSSGKIISSLELGDNSEKQAILIKHKGKGGKDNVENHILEENNKDRGNKRRKNANHISSVVTQTPYFDGCHWRKYGQKWISRAKHSRSYYRCAYSKEQGCPATKTVQQKENDGNGTVRLFNVNYYGQHICNSDGIVHPHVVGATQDSMPIVSQNQNSSSVFVNTDVHGVQDEIFESLFMVPDMPEYLTEFVDVEMARAFEITPMNSPMIPEDIWA*

>SbWRKY55

MKHQQYNSRCLAESSASDHRSAVKEIARGQSLVTQLRAIVLPVLQADERSELVAQMFQNILDCSSKAMAELQMHQSRSTRRPHDDDDVLVDDKKRVKKISSVDCKNEEGVTAAKPRHQHKRRRFDDSVSLETPVPHYDGRQWRKYGQKHINNTKHSRSYYRCTYRQEQGCKATKTVQQQDDSSGADHTLMYTVVYYGQHTCKDNDGANSSPDDSEINTRSSSDSHSSISSTCTDPCDHQNQTSLHEDKPFVNKSEELVTKDMYEPFEMTVFAPLDLDSWELDALLRFGP*

>SbWRKY56

MDIKSSLIMLPPCSHGWEMMETMRRQQELVMQLRAFVLPLLPGVIVDGTSAAEIAVQLFDDVIGCNIGVVSTLEGCLVSTGARGGSSGEPVDNKSLVRKNSCHVTEGETTDEQARHRSVVGQKRRRKNDKRSRSLVTHVPHYDGHQWRKYGQKNINGRQHPRSYYRCTYREQNCFATKTIQQQEQIDSIRRSATPGEEIAKYTVVYYGEHTCKDHSISIVQLPQLVSCMDLQNMEIAQTSSDVQDPEADLDLPALLEVFDNSVIDWEDIWKI*

>SbWRKY57

MENYHMLFGAASTHASSAATPSSYNFMATAAGTSGGGGGGFHDHDRGQRSSGHGGGGSSSSFFAELSSNNDDSKEYGGASSPPGPAAGSGRGESSVGPAAAAGEVDRPPKRKGEKKERRPRYAFQTRSQVDILDDGYRWRKYGQKAVKNNNFPRSYYRCTHQGCNVKKQVQRLSRDEGVVVTTYEGTHTHPIEKSNDNFEHILTQMQIYSGMGSTFSSSSHNMFH*

>SbWRKY58

MEGMPEEKCSLAAVAAELAQIHDMAKQLVEQVADPQQGGGDGDAAAGGGYQRVRELTSTICANVDKALHMLTSNSLDGSPAAGQPESTPSSGGHGSSRGAVLDSDQAGGGTGNAPGQGKDRKTLSKWSTQVRVSNAQDATYLDDGFIWRKYGQKDILGAKHPRGYYRCTHRHMQGCLATKQIQRTDGDPLLLDVVYIGSHTCTQPWGAAAHPNIQSMLPTTEQTTTSGSESGSVLTSEIPGSMASRKRDTGGETRLSKTMIEEPHSTPYKDVMAWFSMGKLRQKGRLSHTCMQQAGQELLGRDDIRQQVMEKILLDRNGVNNCTVICIYGWSGLGKTSLLHALYNDQQLLDAFDKRIWIQISDKIDISMLFRKIVEFAMNEHCSITNIDFLRELVVEEITDKKFLLFLDDADIVNQQFWTTLLEVLNTGAKGSVVVMATRSSTVAAVRNVATHSYSLNPLSEENNLMLLQQYAVVGTDIQSNPDLALIANRFISRFRYNLLHLKAIGGLLCHTDTFSVEKDKFEGSVMPLWICHDVLPVHLKRCLALCSLFPEGYIFGKHHMVLLWISHGCVRPVEGYELEDVGVEYFNELLCRSFFQCSPVHSDKNEMFVMHELMYKVVESVSPDKYFKSEDPVISIPENVFHCSLITSQFQTVELMHRMKQLKHLQTFMVVQPEWKPNNISLPTLNLVGLDDFFLKFTSLETLDLSHTETEELPASIAGLRNLRYLSVNSTNVRALPCELCSLSNLQTLEAKHCRFLTELPRDIKMLVKLRHLDLTKELGYVDLPHGIGELIELQTLPVFHVSGDSSCCSISELGSLHNLRGCLWLSGLESVKTGSKAKEANLKDKHCLNDLTLQWHDDGIDIEDEGEDSKDVADEQVLEGLKPHVNLQVLTIRGYEGRRFPAWMQGSSPSLPNLVTLTLDNCCNCTEFPTIVQLPSLKSLSVRKMYDVQQLSSHTDTHGNGSTAKFPSLELLNLWEMYGLEELFSKESEGDCPRLRKVCISRCPDLRRLPSARSLTELVLHCGKQLPDISELASLVSLKIEGFHGTKSFGLPAAAALRKLEIRSCKELASVDGLSAVLTTVQRLKIAGCPKLVLPGRNQ*

>SbWRKY59

MEGHVAMEWKDPKPGPESLMGFQTRGVRPDTVGGHSNEDAKPGFEKHGFSVDISSPQEEGRSLPLTPQFGQKTSPGSSLAERMQARAGFKVPKLNMPFSTAAGADNSVPGAPSPYLTIPPGLSPATLLESPVFVSNAMGQPSPTTGKLFMSGSTNDNDPIRFGGPPVGDGPDAFSFKPLDLKSSHYTAEAMKEQNTQVSVKTKTKTQPVQEANLLGQLNQQNHNVQTNMNIGGPHDSKLSRLASGTGACNEHVSPPDYGQTAEEGGDAREDYPPAMAAATAPAEDGYSWRKYGQKQVKHSEYPRSYFKCTHPNCQVKKKVERSHEGHITEIIYKGAHNHPKPTPSRRPGVQVQPVHPFGDAGGAQQADAAADNNLGSQSQQANAAAEANHQPWRAGVQDGMDAATSSPSVPGELCDSSASMQQVEYAPRGFGSPEGADVTSAPSDEVDGGDRVTLGSMSHAGADAEGDELESKRRKVEAYAMDMSTASRAIREPRVVIQTTSEVDILDDGYRWRKYGQKVVKGNPNPRSYYKCTHPGCTVRKHVERASHDLKSVITTYEGKHNHEVPAARNSGGHPSTAAAATGAGGGGPRRPEHTSSVHDGLMMRHLGGCGVPFGLPLQPPSRDPLAPMGNYPTYPFTALGGGGGSGGDGGLTSLPSLPMATGNLSAVEGLKLPMLATSSPLHHQHPLLRHRQAMQAAALAAAPMAQVKVEDNVAAGVTAAPSVYQQMVRSGLRLGHQM*

>SbWRKY60

MHACMEGSSQLLETCLPASSLYALSPHHPLLAPLPNQHKLLQMPLVQEQAAANNHGVMLYSDHHHHGGGLLYPLLLPGIPFCPFSAAADAATCDKTTTTGGFAALDAGEAGTSVAKAAGEIASTTTTCNGPSSCNWWKGPAAAGEKGGRMKVRRKMREPRFCFQTRSDVDVLDDGYKWRKYGQKVVKNSLHPSSLVSAFSKLSLCMRTSRSYYRCTHSNCRVKKRVERLSEDCRMVITTYEGRHTHSPCSDDADAAAGDHTGSCAFTSL*

>SbWRKY61

MPPCVWRWHTGRARPPPHLARARPCPCPCRLPLKRMEGDQAGGGGGDLTDIVRSGGAIPGNAAEMSSTAAADEWQLQGDPMLFPPLPSSTTSEAAACSAGGGTGADVFGADPFSGLVDPFSTDYSSGADFLDAMPDAMAKVGFDTAICGGSGSGCGGGGAGGGGQLIDMSRKQPLLPRGVQMPALGVLAPRMVLPSPLSSPREIRPYPPLAGDMVKLGITAGQVAGCAIDAAVVGMQMSSPRSAGGIKRRKNQARKVVCIPAPTAAGGRPTGEVVPSDLWAWRKYGQKPIKGSPYPRGYYRCSSSKGCSARKQVERSRTDPNMLVITYTSEHNHPWPTQRNALAGSTRNHHGKNSGGSSGSKSSQNEKQQQQQQPNNVKEEPKDPAATTTTTSTITTTTTSTSPAAVVKEETLAAGSSSEALGQQVMDTTALAVVDHNIELMDQVFGESYKPMIPEAGHSDDFFSDLAELESDPMSLIFSKEYMEAKPSSGGDRGHHQEKAMSKDLDPLFDMLDWSTNSSSAGSPFEQGKRG*

>SbWRKY62

MAVDLMGCYAPRRADDQLAIQEAAAESLRSLELLVSSLSTQAGAPHRAAHHLQQQQPFGEIADQAVSKFRKVISILDRTGHARFRRGPVESPPRAAAAPPVPAPAPALSLAPLAHVAPVSAAQPAPASQPPQSLTLDFTKPNLTMSGATSVTSTSFFSSVTAGEGSVSKGRSLMSSGKPPLSGHKRKPCAGAHSEATTNGGRCHCSKRRKNRVKRTIRVPAISSKIADIPPDEYSWRKYGQKPIKGSPYPRGYYKCSTVRGCPARKHVERATDDPAMLVVTYEGEHRHTPGAAGPSPLATASPVAAAVSAGNGHV*

>SbWRKY63

MAVDLMSSCGGRAGAYEQLAFQEAAAAGLRSLELLASSLSSPCGAGQRAESPPLGQIADQAVSRFRRVINLLDRTGHARFRRAPVAAVETETTLQAAVEEPQPPQKKAALTLDFTKPVPVPAAAATKPAAPAPAPAVSGTSTSFLSSVTAGGGGEGSVSKGCSLAVSSGKPPLPKRKLPCPASAPQQAQAHQHQHQHQHLAESSAGRCHCSKKKRSRQGLSRRTVRVPAAAAAAGAPGSHVPASSDIPADDYSWRKYGQKPIKGSPYPRGYYRCSSAKGCPARKHVERAADDPAMLVVTYEGDHRHDAAAAAVRARAA*

>SbWRKY64

MADGDPAPALALANEKLPAPAAADVDETRPAPPLEPSRGPDEEKRPLEEEEAEVEAHPPREPTGAPPVDRLGMEVVAAAEADMKANEVEKERGDRAKEKREKDKGKGKEGKEKEKVEEEAKLKVTAVVKVEGTEKEVKVTRPPAGASAETPILAVPVVAVPCFIAPPGFAGQFAMTHQAALASVTAQAQMHLQSPTSSACSEVPSSPFYMTPRSLVPLQQSPSVTEGNICKPIADKSFSSDSKSHHVVVNMVADGFNWRKYGQKQVKSSDNSRSYYRCTNSGCLAKKKVEHFPDGRVVEIIYRGAHNHEPPQKTRFAKERVTPIGVPSGGETLRLVNTEIVESSTPTCKLEQSAISETSEQHLFCSSDCEGDAGNKSENEHPSAEPLPKRRTLETTAPNLTPVLRTVREQKIIVQAGKMSDGYRWRKYGQKIVKGNPNPRSYYRCTHGGCPVRKHVEKAPDDVNNIVVTYEGKHNHDEPFRSSSIPVSAISPSATTTEQPNTSTTSDEKPPTITQKDANSESDKETTLEFGGEKALESAQTLLSIKTNSDDMKNSVLKETSAAVQVQNS*

>SbWRKY65

MADAVESGGRALLVSELGRVQDLVRQLEQQLRAPADAASVDLCRRLVHQIVALTDHSIGMLRASPADLAPSPPLSATGSPISGGDATSDHHHHHPFRAAGASPKKRKATARWTSQQVRVSAAGGGAEGPADDGHSWRKYGQKDILGAKHPRAYYRCTHRNSQNCPATKQVQRADDHPALFDVVYHGEHTCRPPAAAGGSGGAKRAQQQQHNPHAQAALQGLAARLTVATTTAAAAAAAAAALPPMTPESCPVRGASSPWSPVGSDSNGCLQHQGVSPCPVPGYGDWAPEGDLQEVVSSAFAAVSSAAPLPVLDDEFMSLECFAFDHNFDIDTAMPSLYYP*

>SbWRKY66

MEDWMLPSPSPRTLMPSFFNEEFSSAPFSNIFSDDRSNKPLDEIEKSKTFIGSSAQETSQDTKDHPQTESNLFSANQKSTSPGGLAERMAARAGFGVLKIDTSRVSSSGAPIRSPVTIPPGVSPRELLESPVFLPNATSQPSPTTGKLPFLMPNNFKSTMPSVPEKSEDHSHEDSAFSFQPILRSKPSTLWTAEKGSSVVHQTQSLTKDSQGLNVHANPTATKHETEENLVKPKTCDSMFDEQSEEIQNGEDSSAPDTGTADDGYFLRVNRRGMPLLDDGYNWRKYGEKQVKKSEHPRSYYKCTHPKCPVKKMVERSLEGHITEIVYRGSHSHPLPLPNSRPSVPLSHFNDSEADGNFSSKPGPGYDSSTSQGIAPKGQFQDVHSGALETKLSGSLTTTEIADTSVMESMDVSSTLSSNEKGDRAMNGAVPSTNDMNEDETESKRRKMEVSVASNTANIVTDMAAMASRTAREPRIVVQTTSEVDILDDGYRWRKYGQKVVKGNPNPRSYYKCTYAGCSVRKHVERASNDLKSVITTYEGRHNHEVPAARNSNGHPSYGSSAAPQGSSLHRRPEPPQFSMPHAAAAAAYGSLCLPPQLNAASGGFSFGMLPPAMAMAIPVPSLGNFMPAQMPGHGSPMQGCSGLMLPRGEEKVNPEQQSRLPVANGNAAATYQQLMGRWPQGHQM*

>SbWRKY67

MGSGSNSKEGEERVLSHGDVVLIRSDLAVLHGPCFINDRIIAFYFAHLSAGLQDDDLLLLPPSIPYLLSNLPDPASVAAVADPLRLASRRLVLLPVNDNPDASVAEGGSHWTLLILDNATSPSTPRFVHHDSLPGAPNLPVAARLADALRPLLLSGSDSKRGTVPLIEGPTPRQTNGYDCGVYVMAIARALCAWWNNGRDHQEGGDWFQVVRREVGAHSVKAMRADLLQLINTLIQEKAKANSPSKGDADPGSGKMQPHEKVAVLKPVASRPFSRFRPFPNVLQDFNANGSPTITVPEETELIRPKATRSASLLGNLPTQIAATIDAGSDAISEEVEANAEHLTCCDHVTACQAARRNGVRSRLSLDGYNWRKYGQKKVKGSEFPRSYYKCTHPSCPVKRKVETTIDGRIAEIVYSGEHNHLKPGKPCLPRKPLSSTSTEVVVCDMRGTDDTMRE*

>SbWRKY68

MALQVVGREEELLAQLRALLFLPSPAAAATPAAPAAVKVESAGGGSLMGSGGGGRRRRRLQGSKRDRDDDSKAKDEQNQEERAAATEPRHYSPPPCKRRKKKQQSKSKSLVTSVPDFDGYQWRKYGQKQIEGAMYPRSYYRCTRSAEQGCAAKRTVQRNDDDGGGAAAAPEYTVVYVSEHTCTANDSLEAPVILETTTTVVAPSNSAATANTTTYTDSIVVPTMSDHGSCSTITITTGTESPAISGDDITCWSSTSGGASSSDYNYADDDYYYDCGGLFGAAVHGGGWATGPADASSSSSLLEMEDMNGPIRSPVHVPAVGWTIIDPLLLQLVNEPAVCHF*

>SbWRKY69

MKHHHYLHDNRCLPQSSASDHHSLACDCDHRSAMKEISREQSLVTQLRAIVLPALQQADERYELVAQMFQSILDCSSKAMAELQRHHQSDDGARARPDDVLVDDKKRVKRSISDDCISKEEDVVKPRHKQLKRGRFDESMSLETPVPHYDGRQWRKYGQKHINKSKHPRNYYRCAYRQEQGCKATKTVQQQDDSTGTDHPVMFTVVYHDQHTCKDNNGINLGIDDSETNSQSSISTISTDPYGRETPSLDGNKLLDKSADLITRNSMYEPADMTVFEPLDLDSWALDAFLRFGA*

>SbWRKY70

MNILESSTHSGCQVVINEIEHQRALMTDLHDLILPTLDPCSRQDAQQLFQDIFSSSSKVISFLQLGDNSKKPANLIKYRRKGGKNNVESHMLGDEAKEIGNKRRKNAQHTGSVMTQAPHFDGYQWRKYGQKWISKAKHSRSYYRCANSKDQGCLATKTVQQKESDGSTGTVRLFNVEYYGQHICKKDDIIHPYVVETTDYSAPIANYNQSSSSSMFVHNDVLGIHDESFENFFMVPGMPEYLTDFTDFETAEALEVTSMIISEDIWA*

>SbWRKY71

MALESVPTYLSDLGSHQAARAQQQRIRKDERIWTSDTYAPYDDGHQWRKYGEKKLSNSNFPRFYYRCTYKNDMKCPATKQVQQKDTSDPPLFSVTYFNHHTCSSISNPIGSTRDVAAQSASSKAVSICFSPHYSFRDEPQSPIAHSFRGNQQPAERSAYATSQFQWTAASSPSPTSNDSPVKMEVDTFSGASASSSSSSSMGSLPRTRTLLPIGQSRCIEYFHFL*

>SbWRKY72

MAAAAPYARVMEDMVKGREYATQLQALLRDSPEAGRLLDRILHAMSRTIDTAKAAAAEEEEASEVQSDVTCAGTAGSSKRKAAGGGDKRASCRKRGQQGSSVVTKNIKDLEDGHSWRKYGQKEIQNSKYPKAYFRCTHKYDQQCVAQRQVQRRDDDPDTYTVTYIGMHTCRDPATAVASLVVHAAGVTGDDLHHHAGSRLISFAAANNNASAATTSTTTTGNTTNQQLAVLQPLKLECGGGGEQEEVLSSLTPAGSSAAAEAMRNGNAAAAAATTTGPEPDQGDVTSGLQLQQFYGAGDDLAYMARFSYDDTFDLEDIVVFGAPDSITDIYADE*

>SbWRKY73

MSARPPPPPRPRLALPPRSAAESLFTGAGDASPGPLTLASALFPSSDSDGGGGGGGGANSSSGAATTFTQLLTGSLAPPPQQQHEAERGRGGGGVARAGPALSVAPPASASAGASVFTVPPGLSPSGLLDSPGLLFSPAMGGFGMSHQQALAQVTAQATHSPLRMFDHLEQPSFSTAATTSGALQHINSAASMAGISDMTMATANNENASFQSAEASQRYQVNAPVDKPADDGYNWRKYGQKVVKGSDCPRSYYKCTHPSCPVKKKVEHAEDGQISEIIYKGKHNHQRPPNKRAKDGNSSAADQNEQSNDTTSGLSGAKRDQDNIYGMSEQASGLSDGDDMDDGESRPREADDADNESKRRNIQISSQRTLSEPKIIVQTTSEVDLLDDGYRWRKYGQKVVKGNPHPRSYYKCTFAGCNVRKHIERASSDPKAVITTYEGKHNHEPPVGRGNNQNAGISQQRGQNNISSNQASLPRPDFSNTNQMPLGILQFKSEQ*

>SbWRKY74

MEEVEVANRAAVESCHRVLALLSQQQDPALLKSIASETAEACAKFRKVAALLGSGSGGGGGCGHARGRFSRRVRPMGLVNQKSPLGSGSGGGGSPLEMMPSTAAAAAAVAAPSPSTSYAQMRARLNGVPDSRGLDLACSSSKSGGPHPFGAPKLVQPLSVQFQIGNVAHRYPFHQQPPSRQKLQAEMFKRSNSGISLKFESPSPSGGAAGTMSSARSFMSSLSMDGSMASLDGKRPFHLVGTPVASDPADAHRAPKRRCTGRGEDGRGKCATTGRCHCSKRRKLRIKRSIKVPAISNKIADIPPDEYSWRKYGQKPIKGSPHPRGYYKCSSVRGCPARKHVERCVDDPAMLIVTYEGEHNHNQLPAQAAQT*

>SbWRKY75

MGMEDPTATASLDGTISKLPGKLDRLLRHGTRRKKRLMHHVCTLPKGVVDEVPLIKADLEKILAISSDLEDDQAMTARCWRKEVRELSYDMEDFVDQYEHAHAVSCSRSMIRGRKIATQQRRKSKISLPWLREKLRRRLWMANKTREFSARTQEALQRHSLYNLDAVAGASASSRRTCAYSDPAWNSTPCGEEDAYVGINDAMEELLMMMHDDHGHQKLKVVSIVGFGGIGKTTLATELYHKLGHQFECRAFVRTSHKPDMRRIFISMLSQVRPHQPPDNWTVHSLISTIRTHLQDKRYLIIVEDVCTASTWDIVKYALPDSNCRSTILITTEIEDLALQSCDHDPKYVYKMKPLGEDNSRKLFFGLVFGQHECPLELREISCNIISKCGGLPLAIVTVASILSSQPGVQGQWDFVNKSIGESLLTNPTWEGMKQVLDLSYNNLPQQLKACILYTSLYEEDIIIWKDDLVNQWIAEGFIQTTGGQDKKQIGRSFFDRLISGKLILPVDINRNGEVLSCVVHRMVLNLVIRDKSMEENFVTAIHHSQADTMLADKVRRLSLQFGNAEDAIPPSNMRLSHVRTLAFSGVFKCLPSIEQFRLLQVLILHFWGDKDIISFDITRISELFRLRYLKVTSNVTLELGTQMRCLQSLETLAIDARVSAVPSDIVQLPGLLHLRLPAETNLPNGIGHMTSLRTLGYFDLSSNSLENVQSLSMLTNLGDLQLTCSTKEPESLNMKTPFLLMNMILEKLSNLKSLALVPTRTFSYYTKSIDDDAGATCITISDGFSTLSSAPALLQGFEVSPRICIFFCTPKWIGQLHKLSILKFGVRKIDRDGVDVLRGLPALAVLSLYVHTKPAARIVIGKIGFSVIKYFKLKCCDPCLEFEEGAMPNLRRLKLAFNACNADRPSTMPVGIKYLSELKEVSAKICGAEESHTRTAQLAFRDVIRVHAGCQSVNVQCVKQIISSEDDQSSQSSMKTAKYPRIQYLPSPAQAPGRNQNRHRRWPDSIPREQTTVRQMLTPTPYEDGYQWRKSGQRMINNERFPRCYYSCAYRRDRKCRAIKLVQQYNDGAPPLFAVKYCNHHTCVPEANPARRSRGRNKELPLGFGTSVAGAEQGTRLQIRNSYRGDSSSSESSRAPQSSDMIVFYDSTSEETMYVLGPGSWY*

>SbWRKY76

MDLVPKQQQQQRSKEKQEEEEEMMMALAEHGDRPQAAAFGHGGGGGGGRRSEIKEVDFFSTAGGAARRRTDDDDDGDREEAAAGALARGCHNTTVNTALDLLTTRAAAAAATPAAVDGGEGTASGRDTEVPIVDVAATAAVEGELRQASEENRRLRRMLEELTRSYGALYHQLIQAQAQQQQQQACSGGAANPMLPAATTTGVQFMDHAGRVAPAIAGEAAPPAFSGDRGDSDDGSGGNGGEADQNDGMKTPERGENVDRSPAAAAEAPLRRARVSVRARSEAPMISDGCQWRKYGQKMAKGNPCPRAYYRCTMATGCPVRKQVQRCAEDKAVLITTYEGTHNHQLPPAAAAMAKTTSAAAAMLLSGPAVSRDVGALFAGHHVAAPAPLFQYHHPYASAMAGATLSASAPFPTITLDLTHAPSPGAAAAAAAAAGLLQQRQLMPPPVPTMTPFPMYGFTAAAGHRPVPPPQPPAATTLFGLDGSNRSALETMTAAITNDPNFTTVVAAALSTIMAGGAEPPVPRSGAADAGDGSNGSVGIEPATAAAAGARENALHALLQRLHDSRQ*

>SbWRKY77

MQMAASLGLNPEALFASYSSAYSSSSPFVSDYAASFPAAVDSATAFSAELDDLHHFDYSPAPIFTAVGAGAGGDRNEKMMMWCEGGGDEKRLRSSGRIGFRTRSEVEILDDGFKWRKYGKKAVKNSPNPRNYYRCSSEGCGVKKRVERDRDDPRYVITTYDGVHNHASPGAAAIIQYGGGGGNSGFYSPPHSGSPSAASYSGSFVF*

>SbWRKY78

MDDVLRQIDEGFRLARDLMEELPAAQNERTYLADRCHGIVQAYVAAIRMLHPHGGTEDTASSPPPLRPPHPPSPHFGGDGSGSGQHDHEIPQLDLLRPFLGGAPSPSAPSSFPHNLGRLLAESSFINTTPVVDAFGAGTSSGGPVRRQASSSRSSPPVQLRQQHRRRRENGERMTIMVPVQRTGNTDLPPDDGYTWRKYGQKDILGSRFPRSYYRCTHKNYYGCEAKKKVQRLDDDPFTYEVTYCGNHTCLTSTTPLLTIPAGPATVASTAANMLNNSPTDSATALAAGHQDLFMPAAEHPAQALSTAIQLGISWMPSTLVGSSAPEGSSSQVNVPASGRDTAEYPVMDLADAMFNSGSRGGSSMDAIFLARHDRRDT*

>SbWRKY79

MASSTGSLEHGGFTFTPPPFITSFTELLSGTGDMLGGAGGADQERSPRGLFHRGARGGVGVPKFKSAQPPSLPISSPPPMSPSSYFAIPAGLSPAELLDSPVLLHSSSNILASPTTGAIPAQRFDWKQAADLIASQSHQQDDTRAAAAAGGFNDFSFHTATTSNAMPAQTTSFPSFKQEQQQQVEAAATTNKQSAVVASSNNKQGSSGGGNSSNTKLEDGYNWRKYGQKQVKGSENPRSYYKCTYHSCSMKKKVERSLADGRITQIVYKGAHNHPKPLSTRRNSSSGGVVAAGEEQQAAANSLSAAAAAAGGCGPEHSGATAENSSVTFGDDEAENASHRSDGDEPDAKRWKQEDGENEGSSGGAGGKPVREPRLVVQTLSDIDILDDGFRWRKYGQKVVKGNPNPRSYYKCTTVGCPVRKHVERASHDTRAVITTYEGKHNHDVPVGRGAASRAAAAAAVAPTMGALMAAGGHQQQQQPYTLEMLSGGVGGGAAYGGGYAAKDEPRDDLFVDSLLC*

>SbWRKY80

MVKLSNVYIGRRRVGLDSRHHFTILYAVARPAASRSIRLRLASLRQPPTTLLSSPAMTSTPGSFGGTLAANSGPVALSFPTTSFANFLGGGGSSASSSGAADNGGVGLSKFKAMTPPSLPLSSSHPPASPASYLHAFSGILDSPILLTPSLFPSPTTGAIPSEPFNWMGTSESLSGSVKTEQQQYTDFTFQTAASAPPATSTSTMTGASHSASYLQSSVLMAPLGRVGDSYNGGELQQQQQQPPWAYQEPCTQFEAPAAAQPDNSMLGNGGYGGAPGPAVSGCFREQSQSNRPSSDDGYNWRKYGQKNMKGSENPRSYYKCSFPGCPTKKKVERSPDGQVTEIVYKGAHNHPKPQSTRRSASSAPAPASHVLQSVGDAVPEHSFGALSGTPVATPENSSGSFGGDDEINGVSSRLAGNFAGADDLDDDEPDSKRWRKDGGDGDGGVSLSGNNRTVREPRVVVQTMSDIDVLDDGYRWRKYGQKVVKGNPNPRSYYKCTTAGCPVRKHVERACHDTRAVVTTYEGKHNHDVPPARGSSASLYHRAALAAHQMPQQAGGGSCYQQQQQHGGLVRTADGFGFGASGGLHGGAPMMQAAESGFALSGFGHPAGTAAYSYTSHQQQQTTTTNEAMYYAKDEPRDDMFFEQPLLF*

>SbWRKY81

MTLSPPQPPPSSSPRHAAIQELRRGTQLAELLRQQVELIPEPNRRQAAVVNVGEISMAMESSLSILQSEMEHPFVSEVMAAPTAYSDGGSTSRERNGPVARTRRVRHRRGRDGAELPIKEILTEAPENDHFHWRKYGEKNILYAEYPRLYYKCGYSDDHKCPAKKYVQQQSNTYPPLFLVTLINEHTCDTLFRDEPSSSSSGSQVLDFTKASLSPEEDSSMPVSMHRYSFSYDGY*

>SbWRKY82

MENSPLHGVIRQPNTPPLATGTCLAPLPPAVAAPQPPEQHACSSDATTTSLVPGAATMMSCPPAAVDWASLLLPRAPGTLHVGTTPPPVASEVESGGSSAVTVAGSSASATAAGEGDNNYKAGKAGKAGGGGRGKKKASRPRFAFQTRSDNDVLDDGYRWRKYGQKAVKNSAFPRSYYRCTHHTCDVKKQVQRLAKDTSIVVTTYEGVHNHPCEKLMEALSPILKQLQLLSQLQSCTNQLL*

>SbWRKY83

MAAVGARPVLYHHPAPAGDAASMSSYFSQGGSSTTSSSASASFSAALAPTTTTLAEQFDISEFLFDDAGVAGAPGVFADGSAPVVVSDAAAAAGGGAISAAAGSAAAAAEAVPERPRTERIAFRTRSEIEILDDGYKWRKYGKKSVKNSPNPRNYYRCSTEGCNVKKRVERDRDDPSYVVTTYEGTHNHVSPSTVYYASQDAASGRFFVAGTQPPGSLN*

>SbWRKY84

MSGARHEHHLSGDFQFHDELASLFAHQRPDAAPMAQPWFMDYLHATAAAASPLDCDAFVGDFIDVPAVAADEVVKRELVMVDTAAAGSGGGTPTPTTAPLTPNSMSMSSTSSEACGAGAGAGEESAAGKCKKEDGEEEGLESKDDGSAAGKGDGGEGEEKNKKGAANKGKGKGEKRPRQPRFAFMTKSEVDHLEDGYRWRKYGQKAVKNSPFPRSYYRCTTQKCPVKKRVERSYQDAAVVITTYEGKHTHPIPATLRGSSHLLAAAHHHPMGGLHHVHPHFRMAPPPPPAALGGFRPGGGANAFDALGLGLLQPPSSSQQQQQGHHHHHGAAAMQQLAVSGGAAGVQQVNAAAAMASHAALPDDGDQHGLAAIAGAAGTTTAATTAASAPLRMQHFMAQDYAGLLQDMFPSFVHSDDDGHHHHH*

>SbWRKY85

MESSYLGKRRLNGGADRETTTARAPAASSFLPAAAMGYEYGDAAEAADHHHLPRRVAAGEMDFFKKERKDAAAAAALAAFVPSSSDEHGIKEDDLTINMGLHHVSGRKSSIRSEESSVDDGVSSNGVDHRETKAELALAKSELGRLNEENKQLKDMLSRMTIKFNAFQVQMPVYTTLMQQQQQRTNNHQALLRGAPGHELMNVDPETKDHQEGSGGSHLLPRQFISSLGTAPDDPLRSVGSDAMHGGGNSSGSSTSNAEPPPPQPLDYCPGNGLMVSSKEMMPLPAFEHGHQQPQQHLAHEMGSSSRADEPPQPHHLAAAQQGWLSNKVHKFLPSKGPEPVPEAATMRKARVSVRARSEAPMINDGCQWRKYGQKMAKGNPCPRAYYRCTMAAGCPVRKQVQRCAEDRTVVITTYEGHHNHPLPPAAMPMASTTAAAASMLLSGSMPSADGGSLMAGSNFLARAVLPCSSNVATISASAPFPTVTLDLTQPPPGAASASASAFAQPPASAPAQARATGTEPSQLQAALADAAGRPMPLTTQLFGQKLYDPSSKAPAAQADAAGDTVSAAAVIASDPNFTAMLAAAIKSYIGSSGSGSNGAGGSSGTTVLPPAGASSAGDSSRDDKVGEQGS*

>SbWRKY86

MDGYGSYGGEKSALASELAQVLAMVRELEARMDQDPLPAAARELCAELASSVDRSIRIARSCCVDSPASGSGSPRSDGGNAGAAQSKRRKGTPCVRRQLRAASVQDAAALDDGLSWRKYGQKDILGAKYPRAYFRCTYRHSQGCLATKHVQRADGDPLLHDVVYHGAHTCAQAAHPSAQQLRQELQLQPGHGAQEDQASPLALETEGLRAALLEPMTPYSFATVAGAGAGASAGADFAGWCPLLSPTALDWQFEELFTNAMEPFQWDLYTAN*

>SbWRKY87

MVVQLSDSRRDADADGQMAGAAAVTPANSSVLSSSSCEAGADANDDDEEPSRRRCGKKGRIEGEEEQEGEGEADDDAADRNCKSSKENKKRRGEKKAREPRVAFMTKSEVDHLEDGYRWRKYGQKAVKNSTYPRSYYRCTTARCGVKKRVERSQQDPSTVITTYEGQHTHPSPIDLLRRGGGAAALMRSAAVAGGFRRPDDLLKIDDYAGTPIGFLPLLPPGGIGAGGGGRLLHHRARSSQLAAVDAYGGMLELDFIPSIPR*

>SbWRKY88

MSGGATGVGGGSGYGGFYHGDDPATSDQLITAFDNDGGGGFFFQQTVSPPCAGEVDGGTAPYASIADYLQGFLDPAGLAAHFGSDDAPPPCRLGGGADDEYDAVVAVKQEMVVQLSDSRRDADADGQMAGAAAVTPANSSVLSSSSCEAGADANDDDEEPSRRRSSKENKKRRGEKKAREPRVAFMTKSEVDHLEDGYRWRKYGQKAVKNSTYPRSYYRCTTARCGVKKRVERSQQDPSTVITTYEGQHTHPSPIDLLRRGGGAAALMRSAAVAGGFRRPDDLLKIDDYAGTPIGFLPLLPPGGIGAGGGGRLLHHRARSSQLAAVDAYGGMLELDFIPSIPR*

>SbWRKY89

MPKSTVSVYMNPLPVSPHLGTHTHARSIAMDCSNDWDLQALVRSCGGGGTAAAAACNSGAAPTATRGGYDAPSREAADDASVVVGGGGRVVATAAAGQEFLGQPVAAWRRNLDYLDLVDHELLRMPFSITPSSSRETTSGGAPGQQMIRQPRRQPGRKPGVRTPRAKRSKKRQVKKVVCEVPAAGGGVSSDLWAWRKYGQKPIKGSPYPRGYYKCSSLKSCMARKLVERSPAKPGVLVVTYIADHCHAVPTMLNALAGTTRNRPAAESPDDGDHHHHQEHHDHETSDGAPAASADNKLDDDGADAASTMTVEENDAWLVDHMALEDDVVDGDCPFDDFLWPFDDDLDQFLDVDGGGVLGRRLSL*

>SbWRKY90

MIGTRIRETYSAANSDVEINQIKDDDHILDGNLFKSLHESSSRKEASSSSLRERSETDEASNQTSANHNKVDKDKLASTRAEMGEVREENKRLKTMLSRIVEDYRSLQLHFHDVLQKGQAKKLADPSTIMPTGIEEPEFVSLSLGTTTSMHRKEDKNSAAEGKGREDFMSIKEEGLSLGLSACKDGATNNNVKIQPEVMTLSPEVSSEDAKDDAMEAADQQWPPSKAEKSLRNVGTGPEDDIGPLPQAKKARVSVRARCDAPTMNDGCQWRKYGQKIAKGNPCPRAYYRCTVGAGCPVRKQVQRCADDMSILITTYEGTHNHPLSASATAMATTTSAAASMLTSGSSTSLRFPAASPAAAGLSFGFPPAAAHDPSKHFFLPNGGAASITSTPSYPTITLDLTSPAATSQAFSLGNRFSSSLVHGGARYHHPTSLSFSNSGPSALSGAAWPAAGGAGYLSYGSPAASLFNGGAALSSINGRQQGGEFPVLYQPQQKASAAASGSAPEGVLTDTIAKVITSDPSFQTVLAAAITSYVGTQGGNRSSAGGEGGSQLQGLKWGQHLGLGPSPSSPGAACSSALLARSSSTTAAAVVEQGSNGHRSFLQPSLGLSGSHSTSTSPVENREH*

>SbWRKY91

MQDGAGAEAAGSSMQALLALLADGEEQARQLGEMMADDPWSRAEHYRGAARRLQCTLGKAAAVARAIEAAAPGSSRGTDDRSDSPRSADESSGRTTTEVQERQSMFKRRKGLPRWTAKFRVPDASLDATPDDGFSWRKYGQKDILGAKFPRGYYRCTYRTAQACGATKQVQRSDTDLCVFDVTYQGEHTCHQKQRASATVAAAPAHGAGSQSPPPPPPLEQQQQQQDPSMMQLLRLGFKRVLKVETTPGLHDHGIGHRDSGPASAPAAPFSFPSASPFHLAGEATDNPAAAFSPPPASSYFPAPHPVAVDGSFYDYEASPVALMRGAEPSELGEVVTRAITTGPAAFDYSSLFHHQAELDDPHLPFPPFGGPPHGPYQ*

>SbWRKY92

MDDGRDGNNWDLNAVLRSGCHGPMPPPPPPPTRTANNPVARYAPPPPAQPSYAFTVLAGGLGHQAISVLPQPQALDQDPPHAAGRGPSLDLPLLPEPDYTAAVGNTPAPLNPPWPRNEIPVPSVQQRPADKHKTPPSSGCDAAEGSSRSKKRDNRTTKESKVVLVLAEDPTPPDSWAWRKYGQKSIKDTPYHRSYYRCSTDKKCKARKHVQRCLTQSFLAVSYIGEHSHPMPLARNGQAGTTHQKPPPRQPTSPFIRTPAKEDQPHHQAPAPPPATSSSPFAMISAAKQTPAPPAALLPPPSVEFDNEKDDDAVAVRMLLNDMDMTPEDALKFVNPEEEPLDGVGDDLLIPTPEELAPFYYGDEENMLYPMASEPASGGSRNTKA*

>SbWRKY93

MEGDLRWCCGSSSNDWDLHAVVRLASCSGGSRSRVTSPSPWASDESFSCLPPPPQSQKDEVTTDAAALQQPLISPAVDDLCGLQQAFLAATPQPRSEAPPPQPPAKPRTSYRNNDGGVGGGPTRSKRKKKKSQVTSKEVTRVPVGTSADPWAWRKYGQKPIKGSPYPRGYYRCSTDKDCRARKQVERCRTDASTLIVSYTGEHSHPVPLHRNALAGTTRNKPQPAPSTSPAEQPPAASPIVGVEYEEDDTVAASVLLEDAETEGEEDVLSLFLELAPSPSNGSGSKDVMVSTELHNGRGSQKVVALSKLHEFRHPATTSSSRTSDGLGAAPAAMNVTHENCPFSGLRLTT*

>SbWRKY94

MDPWVGHQPSLSLDLNVGLLPTARPAVPAKSTKVLVQENFMAVKKDNREVEKLEAELRRVGEENRRLSEMLRAVVAKYTELKGQVDDMVVATANHTGSSTSEGGSAASPSRKRIRSAGDNSLDTAAQHHHRRKPSPPLAAAVAAHDQTECTSAAVSVTAAAFRRAVREECRPKVSRRYVHADPADLSLVVKDGYQWRKYGQKVTKDNPCPRAYYRCSFAPSCPVKKKVQRSADDSTVLVATYEGEHNHGQPPQHDGGRAARSTATAQAQVASEAAVRPVAAPLPLQHPHQQQKQKQKQEAATTVPSSEVARKNLAEHMAVTLTRDPGFKAALVSALSGRILELSPTRD*
