# Supplementary material for: Genome-wide Identification of WRKY transcription factor family members in sorghum (Sorghum bicolor (L.) moench)
Source: PLoS One. 2020 Aug 17;15(8):e0236651. doi: 10.1371/journal.pone.0236651 (PMC7430707; doi:10.1371/journal.pone.0236651)
Supplement: S3 File — (DOCX) [file pone.0236651.s003.docx]

>SbWRKY13

TTCTCATATATTTCTAGGAGAATGAACCAGGTGCTAATTATTAAGGATAAGATAAAGCGTGCAGTGAGTTGGTCAGAAGTCAACCAACCATCTCAAGGCACTGCTTGCCAAGCTGCTGCCGGCGGCGGCGGCGGGAGCAGGCTATGGCGCCGGTGCAGAGTGCTCTGGCGGCGACGTGGACGCCGTCCTGTCGGACATCACTGATTCACTGTGTCAAGCGATCACTTCTCTGCGGATCCGCACCGGTGGGCAGCCAGCGACCGCCGGCATCGCCGCCAACAGGAGCCCAGCCGGTGCCGGCGGCAGGAGATCAGCAGCGCCAAGGAGAACTTCTCAACGGGCCAGGTGCCAGCCGCCCACCTTTTACTCCACTTATCATCAGTTCCTGTGCTAATTGACTTTAGTGTTACACCAAAGCTAGTAGTTTCAGTTACTAGTTTGTGAATTCCTCGTCAGTCACCTCACCTCTGTGGTATTCTTGAGTCTTGACCTCTTCATGATCCAGGATGGATGCTGCATCCCGGATGACAGTGCTACAGAATGGAGTTCATGATTCGTACACATGGAGGAAATACGGGCAGAAGGAAATTCTGGGCGCCAGATTTCCAAGGTATCTCGGATTCTTTTCATGCGTTACTAAATTACTGACTCTCTTTTATTAGTTTTATGTTAATCCCATCAGTAGCCGAGTTAGTTAAGAATTTCTCATGGAAATTAAAACAGAGTATTCAACCATTCACTGGAGTATCCTGTCATTAATTAATTCTGTACACTCTAATCATTAATATTAGTAGTAAATAGATTTAGTTGGTTACTTCTAATGACCGAGTTGTGTCATTATTGGAAAAATAAGATCTCTTTAGTCATGAATATTTCTATCTAAACTATTCACGTACTTCCCCGTCATCTAATATAGTGTATTATAGCATTCAAAAATTATCCTCAAATATAATATTCTAATTAGAAAACAAAATAAGAATACATGTGTCTTTTATATTTTATCTTAATTTATCTTAAGACTCCTAGAATGTACTATATTACAGATTATGTCATTATGAAGCATAATCTAAAATAAAGCACTAAGTTTAAAAATAAATCAACAAAAATCTCTAGCACGCCAAAACCAATAAATATGTTTAATATTAGGTAAGAGTACTTACTGTACAATTATGCCCTATGTTCTGCAATTAGCACATAGTACAAGCATTCAAAAATTGTCAAGTACGGACTATAAGAGATTCCAGGGGGGACAGAAACTAGTTTTGTATTAAAATTCATATGATGGTTGCTGCAGGAGTTACTACAAATGTGGCCGCCGGCCGGGCTGCCCCGCGAAGAAGCACGTGCAGCAATGCGACGCGGATCCGTCCAAGCTGGAGGTCACCTACTTGGAGGCACACACGTGCGATGATCCACCACCGTCGTCGTCCCATGCTGTTCCAGATCCGACGGCCGGCTCCGACGCTCTGCTCGTACCACCAGTCCCGACCGTTCCGTTTCCATCAGCTCAGTGCTACGGCGGCGGCCGACCGTCGCCGCCGCCGCTGCCGCCGTACCAGGTGCCGTACGCCGCGACGACGCTCGGCTCCAACGTCCTGACGCTGACGGCCACCGGTGTTCTTCTGCCTAGTGCAAGCTACGACCCTGTTCCGGATGTCACGGACTGCACGCCGTCGTTGGAGCAGGAGCAAGACCATGAT

CTGCTTCACATACCTTCGCCGGCTTGTTCACAGTCAGAGCTGCTGCCGATGGAGGCTGCCAAGCTTTCACCGCACGCGCACGGGCTGCCTCTGTCGTTGGAGCACACGCTGGATTGCGACTTTGCTGTACCCGAGCTTTAATTAGCGTTCTTGATGTTAAAGAGTGTGATCGACCCTTTCACGGTAGCAGATTACATACAACAGCATCGATATTCTTGTTTTCTGTACAGCTATTACTGTAATGCAATGCGATTAGAGTAGTAGTGGTTTCGGTACAATATATGAATCAGAATTCACAAGGGAGAGCCCAGAATATTATGTACTTAATCCATCCCAAATTGTAAGTCGTTTGAGTTTTTTAATATCAAGTTTGACCACTCGTCTTATTCAAAGTTTTGTATAAAATATC

>SbWRKY6

TCTACATGTTTCATTCATTAAACTTTGCGATAACATGGGAGCAGCAGTAGAGCACAGGACGTTGTAGACGTACGGTCACTGGCGGCGTCCATGGATTCAAGCTCACAGCCCGGCGCAATGTATGCATCTCTCTCTCTCTCTCTCTCTCTCTCTCTCTCTCTCTCTCTCTCTCTCTCTCTCTCTCTCTACGCTGTGTTTGATGCGTTTGCCTTAAACCAGCTTTGGTTCTCATGCATGCATGTATGGTTCATCATGTTTTTGTCAAATTTTCATGTAGCAACATATATTGTCCTCCGTCCACAACAGATAAGCTGATCCTGCTAGTCATAGCTGCTATATACAGATCAGCTTATTAAGTTTGCAGGTTGTTGTTATGCGTGTTCTAATGTTCCTTGGCACAAAAACTAACTGTGTAGTGATGCACGCAGAGGCAGCGGAGGAGGAGGAGAGAGAAACCAAAGGGAGGAGGACGAGGCGGCGGCGGCGGCGGCGGCAGAGGCCGGCTACGGCAGGCAGCTGGTGATGCCCGAGGACGGGTACGAGTGGAAGAAGTACGGCCAGAAGTTCATCAAGAACATCCAGAAAATCAGGTACTTGCTCCGTTCGATCCAACATGCATACGTAGCATTTTTTGCATCGAGATTGATCTCGAGCTCTCACATAAAGCTAGTGCAAACTTGATCACATATACCATTTTTTCGTGGTCAAATCGTTTCCCGCCATACGCGTGTACATCGGATTAATCAATAGCTCGACGTTGACCAAGCTTGTTGACTTGTTCATCTTCGTTCCTGTGCATCAAATCGTTTTATTAATTAATTGAGTCGATGTGACGCCGCCCATCGATCGAACACTGGTATAATGGAATGTATGGGTTGCCCGCCGTCCCCGTGCATATATGCATACGTGCAATGCTTTGCTGCCAGATCTTATCTTTCGAAGAAGAATCAACGGAAGAATAATATCCTCGCTTTATTATATTATTGATAACGGTCAACCAAATAAAAAGCCCTGATGATGACTTGATGAGCAAACTGCACAAGTGTGTTTTGCATTGCATGCCAACTGATGATACCGTACGTGGGGTGGTCCATGATGCATGTGTGTGATCCAAATCCAACAATGGCGCAGGAGCTACTTCCGGTGTCGGCACAAGCTGTGCGGCGCCAAGAAGAAGGTGGAGTGGCACCCGCGGGACCCCAGCGGCGACCTCCGCATCGTCTACGAGGGCGCGCACCAGCACGGCGCCCCGGCGGCGGCGGCTCCTCCCGGTCCCGGCGGCCAGCATCACGGCGGCGGCGCCTCCGACTTCAACAGATACGAGCTGGGCGCGCAGTACTTCGGCGGGGCCGGCCGGTCGCATTGACGCGGGGAGCCAGGGTCTTGTTTACTTTCTAAAATATTTTATAAAAATTTTCACATTCTTTATTACATTAAATTTTGCGGTACATACATGATGCACTAAATATAGATAAAAAAAATAACTAGTTACATAGTTTATCTGTCATTTGTGAGACGAATCTTTTGAGCCTAATTAGTTTATGATTGAACAATATTTGTCAAATACAAACGAAAGTATTGACAAACCGACAAGAAAGGCCGGCGGCGTTGCGTCACGTACGCATGCATCAGCTCCTGTGCTGGCCTCTGCTGGCTGCCGCTGCATCGATCGATCGCTTTCGCTGCGCACCGGAGGGCAG

CGGCAGGTGCTGCCGGTGCCGGTTGACGCCTTGCGCCGGCGCAACGTGATGTTGAGTGCGGATTAATTGTTGCTGCTCCGGTTAACTCTCTGGTCTAGTGCTAGTGTACGGCTACTATTAGGACGATGGTGCATAATTGTAATTTTGATATTGTACATGCATAAAAAACAATATTTAGCTGAAAGTGGGAAGTAGCACCGTCGCTATTATGTTTTGTTTTCTGCAAAGTGTAAACTTGTCGAAAGTCTCCAGAGTTGGGTTCGAGGCCCT

>SbWRKY43

TAGCTTCCAACCTGTACTTGATGGGAGATGGGGACTATGGGCTGCACCCTGAACCCGGCGCCGCCGACGTGGCCGTCTGGCCCGGCGAGCTCGACGAGCAGCTCATAACCGAGCTCCTCAGCGACGACAGCCTCCTCCTGGGCACCCTGCCGCCGCCGCAGCAGGTCCCCGCCGGCGACGACCCGGAGGAGCAGCACTGCTCGCGTGACACAGGCGGCGCGTCATCTGCTCCCGCCGCGCCGTGCATCAGCGGCGGCGGCACCGCTGCCGAGCACCGGGAGCTGCTTCCGCAGCCGGAGGCGGTGAGCAGGGCCCTGTGCTCGGTGTACACCGGCCCGACGATCCGGGACATCGAGAAAGCGCTGTCGACAACCAGGCCGTACCCTTGGAGTTGGAGCAGCAGCCGCTACAGCCCAACAATGCATCTGTAGGTGCATGCATCTCATGGCTTTTTTGATGAAATCAGGAGGGGTTTCCCCCTACTGCATCGATCCGTGCATGGGTTGAACAAAATCTTGGTGGTAGCTAGATGGCTGATGACGACACATGCATGGTTTTGGTTGAAAATTGAAGTGGCAGGTTGGGAGCGCTGAGTCGAGCGCCGGAGAAGTACACTACGAAGGTGAGGAGCTGCGGCGGCAAGACGCCGAGCGACGGGTACAAGTGGAGGAAGTACGGGCAGAAGTCCATCAAGAACAACCCCCATCCCAGGTGCAGTACCACGATCCACGAGTCTACTAGCTCTACTTCTAAGAGTTTTAGTACACGTTCTTTGTTAAATTTGACCCTTAATCTTTTTTCCCCTTATAAACTACAGCATTTTTCAAAATTGCTCGTGTTGTTCTTGACATATATTCATTGGATCGCAAATAGACCGTCTAATTCGGGGTCATGTATGCACATGCGTGATCTCGCAGGAACATACATATGCAATCATGCCATATAAGTTATGATTTTGTCAAATTGGTCATACCTACCATATGATTGCATCGCTTGTAGTACTGTTACGTACTGCACATCTAGATTTTGTCAACTTATGGCATGATCGTCGATGCAATCATGCAATATGCACGCGATTTTAAACAATGCCGCCGCGCGCAGGTGAACAATACCCGGCACGACCCCAGGGGCGTGTGCGCTCGGCCGCCGGCCGGAGCGGTGCGGCCATACGCTTGCCATGAACTAGCAGTTTCGATGCAGCTGGAGAGCAGAATCGCTGCAGCAGCACACGTTGCGTTGGTCGTGTAGTGTCGGCTTGTGTCACGGTCGATGCATGGAGTACGTGTGTGTGCTAGATATCTCCGATGGTAGACCAGACATGGACACGTTTCTGGATTCTACCGGCCGGTAGCTAGTGCCGGATGCAGACCCTCTCACAGCGGCTGTTCAAGTCATGTCATGCATGAGCTGAGCTGATGACTGGAGATGGCAGATGAGGCCATGAAGGCCTTTGGCAGGGAACCGGGGTATAGACCAAAGTACTGACACGTACGAGAGCAAGAAGAGAAGGACTATACTTGTTGTATATGTCATGGAGTGAAAAGAGGTTGGCAAAAGGAGTAATTATTAGTTAGAGAGAAAGCATTTGTGTGACCGACTC

CGATCTATAGTAACTTCTGTTTTTTCAAAGACTCATTTTGTACCAGCTCTTACTCGTTTCACCGGTTAGCTAACTATACTATATACGTCATTAATAGGCTGCTCCTCTATAGTAGTACCTTTTTGTGTGTGACAAACAAATCAGTGTGCTCGCCGCTCATGCATTCATATTGTTCTGCATGATAACAGTATATACGACATGCATGTATACCTTGATATAGCTTATTTCTATATTGGAAGATAGAACTGCACCCTTTGAAGAACCCATCCACACACAAGTCTTTAAGTCGACAAGTATCCTCTTAAACCTCACTTATTCTCGTTTGCATAATAATTCTCTTACCAAACTGCATGCGAAGATTAGTCCTCTCTGCCTCTGCCTTGACTTACCTATTATTTCTTCTATATTTCTTATTCCATCCCAAAAACATATAGTATATATGACAACTTTATACTATACTTATCTAACTCCTTAAGTGCAGAAGCACCTATTGTATGGTCCTCCCTTAGCAGATCAACGACCCTGTCCAAAACACATAATGTAATTCACTGCAAATTGCTTAACATAGACCATATGTAAAGAGAACACATATTACATTCAAACAATGAAGGAGTATACATGGCCAAATCAAGGTGTGGCATGAACCATACCTTGCCATACCGAGCCCTCCACCCTTGAACCCTTAGATCAAGGTATGGCACGCACCATATATGGTACCTTGCCCTCTCGAGCCCTCCGGCCTTGAACCCATGTCTCTTCATCATCACTAAGAGATTTTGCTTCTTTGAATGTCTTTGCATTCTTTGACTCCTTTGTCCCAACTTGTGCACTTGCTCTAGAAGATGCCCTATAGATCAACAGATGCTTTCTGATCAACATTGGTATATGAATCTTGTATTGGCTCAGTAGATTAGCTAGCCTACTGTTGGCTAAGTTACTGCCACTAGCCACAGTCACATATATAGCCTTCTAATGCCCAATAGGAGTAGATTAGCTAGCCTACTGTTGGCTAAGTTACTGCCACTAGTCACAGTCACATATATAGCCTTCTAATGCCCAATAGCAGTAGATGTCAGTTGCATTTCTCAGTCGCAAGCTAGTAATTAAGAAGTTAGAGTGGACTACCCTTCTATATCAAACTGATAGATCCTTGAAATTGGTTATATCCATATAGAGAAAACTAGTTGAAAATATGCGGTTTCAATTCGTAGTACACGTGCCTACCGTCATAGCAAATCTCATAATAAATTGGAATGTGCACAGTAATCTTGCAAACAGAGTAACCAATCCATCCGAAAATCAGGTCAAGAGAAATAGGAACAAATCACTACCATCTGTGAAAATCAAGGCAAACAAACTCATAGTTTTGACAACATCGAGTGACACCATCTTCATCCTTTCCTTGACAAAATGCAGTAGCAGCACCCTTGGGAGGCTAACTGGGATAGGGTTCGAGTTGGGTGCACAGAAACCCTAGCTCGAGTCGATTTATGCTAAAGGGTAGTTAAAATCAGACTATGGGCATCATGTGTCATTTTGAAAAGTTTCTCATTCGGTCGATTAGAAATAGTGTTTTATTAGAAATAGCCATGATTTTTTAATAGTTGGCATCTAGTAAATTTCTATGTTTGCAGTATAAGCTGGCCGACTCCACCTATGTTCAGTGCACTTGAGCAAAATTTGTAAAATAGAAAAAAAGA

AGAAAAAAAGAGAAAGAGCTCCGTTTTGGTGGCGAGGCAGCCATGGCGGCATATAATAGCCAGTGCCGGCCCTAAGGGGAGGGCAGCAGGTGCGACGGCTGAGAGCCCTCGCGGGATGGGGGCCTAAGCTTTAGTATATAATACTATATACCTTTTAACTATAGTCCTTATTTATTAGTACATACATATATCTGGAGCAGTGATCTATTACCCAAAAAATTGTGAGCCAAGGAGACGAGCCACCAATACCCGTACACACGCTGCCACACATAGTGATCACGAGTCGCGACTTCTGGACTTCTACTGAGACGGCGAAACAGCGAGGAGAGACGCAGCAACATCGACGATACAATCACCAGTTCACCACACGATCACAAGCTAGTGAATAATACCAAAACCTCTGCGCCCGAACTCTCTTCTTCCAAAATCCAAACGATAGTGGCGACCAACCGATATGGCTCGACGAGGACTACGACATGTGATCTTGGTTATTTCCTTCTACCCTTTTATTTTTTTTATGATTTATGAATCTTGGTTCTGATTCTGAATTAGTTCACGTCCTAATTGTCTAGGCACTAGGTTTTTTGTTTAATTTTTGTCTAGAACTTTGTACTCATATAGTCATATTTTTCTTGTAGTTTAGGTCAGGTGTTAGAATTTTTAAATATTGCCTAAGAAACATTTGGCCGGGACTGTGAAAAGAAATAAAAAAGACAAGAAAAGTAGTTTATCTATATATGATTGAATTGTCTTTCCCTTTATGAATAAAATTAGCTTATATTATTATAAGATTTTATAGTTTTAAAGCGTTGTTGCGATAAATCCGCCAAAGAGGCCTCAAATTTGTAGGATCGGCGCTGATAACAGCCCCCTGGAAACATTGCCTCGTTGATAGAACGTGAGTACTATATGTAGGGCCACTAATAATTCAGAGCACCATCTCGTGGATTATTGGTTATACCAGTGCCTTTGCTAGCAGTAAAGAGAGTACGGAGACACATACTATAAACCATCTCGCCGTTCTGTGGTGCCACTGTGTAAAGACAGTGCAAACTTACTCCATACACAGTAAGACTACTAACAAAGAAAGGTGCTACACTTTCCTGCTGGACCTGCCAAGACCTGGCTTGTCAAATGTTTATTGTTAGATTAGAGAAACAGGCCAGCTGATAGCGACATTCGCGTCGCTTACCGCCTAGAAGCAGAAATAACTGTTCTAGCAGGAGTTCATCATATAGTCCCTCCTTGTCACTGGATCTATCATTATTCAACCCGTTGGACTGACTATCCTCAGGATCAAGCCTTATTTAGTTCCTAAAAATTTTCACCGTCACGTCAAATCTTACGACACAGGGACAAAGTATTAAATATAGATAAAATAAATAAATAATTATACAGTTTATCTGTAATTTACAAGACAAATTTTTTAAGCCTAGTTAGTTCATAGTTAGATAATAATTACCAAATACAAACAAAAGTGTTACATACCTAAAATAAAAAAAAATGCTAACTAAACAAGGCCACCGAACAATAAATAAAGATGTGCACGGCACGGCAACCACTAACAACCATCTACGTCTCATTAACTGGCTCTAAATTAACTAAAAAGGGTTGGTTTCAGTCTTTCAGGATGACCTGTCTTTTCAGGTCCAGGAAACCTGAATGCGTGATGCGTACCACCTGTTTAGCGAGAATAGATT

TTGTTTCGGTCACGTTGACGACCGATACTTCCTCCGTTTTCAGAACAAACTCTGATCATTTATAGTTTTATCCTAAGTTAAAAAATATGTAAATCTTTCAGTCATCATCAGTTCCTATAAATTATTGGTAGTATGATTTAACAACATGTAAATTTTCTTGTGAAAGTAATTCTCATGGTGAGTCTAATAAAAGACAGTATTTTTTGATTCCATATTATTCTAAAAAAGCGATGTAAACTACTCGCTGGTCATAAATCTGTGTGCACGCCTGTCTGTGACGCAGGAGCTACTACAAGTGCACGAGCTCCCGGTGCGGCGCGAAGAAGCACGTGGAGAAATCCACGGAAGACCCGGAGATGCTGATGGTCACCTACGAGGGTCCGCACCTGCACGGCCCGCAGCCGCTCTTCCCGCGCCGGCAGTGGCTGTCGATTGACCTGTCCGGCGCTGCGGCGGCGGCGGCTGCGGCCTCGAAGACGAAGCAGCAGCAGGCCAGGGTCTCCTCCTCCTCCTCCCCGGCCGCGTCAGCCGCGCTCGCAACAAGCGACGACGGGGGCGGCGGCTGGCCACCGAGCCAGCAAACGACGACGCGCGGGCGCGACACCGAGGCAGCGCGAGGAGGGCCCACGGCTGCTGCAGGCGAGACGGCGACGCCCGGGCCGCCTCAGATCGGACGCGCGGGTGACGCTGTGTCGACGCAGCCGCGCCTGGTGCTGACCGCGGACTCGTGCGACGACGGCTCGGCCGCCTCCGTGCCGCCGCCGTGGGCTTCTGCGGCGTTTCCTCACTGTGACTCGCCGCCTATGATGACCTGGTCGTGCCCTGACTTCCCCTTCGCCTGGTCCCCTGATGAAGCTCCTCTGCTTCTGTAGAAGCCGGTAACCAAAACCAGCTCAAAAGTCTAGGAATAGGATATATAAAATGGCGATGCATACGTACGTAGATATCTCAAAAGGGATCAAAAGATCAGAGACCCCAGCGGTTTTAGTATATGTATATATATACTGCATGCAAAAATAACCCAAGTGACTCAAGCCCACAGAACACTGACGTGAAGCTACCAAAGTTGGTGCAGCGACAGTGACAAAAGGGCTTACCACTTCTCTAGTTAACCAGACGACGACTATTACTACTAGCTATATTTGTACAGTAAGCTGGGCATCACAAGTGCCAATGTGTGCCTTATCACCAGAAGAAAAGGTTACTTTCCTCTTTTACCCAGACGTTAATCGTAAAACCTCACTGGTTATTCAGGTAA

>SbWRKY78

AAACCCAAAGAAGAAGAAAATAAAAGGTAAAATGAAAACAAGAAAAGAATAAGGAAAGGACAGCAAATAAAATAAAATTGAAAAATAACGACCGAAAAGAAATGAAAAAGGAAGAATATAGACAAAAAAATAAAATAACAATGAAGAAACATAGAGTACGTGGATTTAAAAACCCGTAACAACGTACGGGCATTAACCTAGTTATATATACTAGTAAACGTATAACAACTTTGTATTAAATATGTAGCAACTTAGGTATTAAACGTGCAACAACTTATGTATTAAGCGTGCAACAACTAATTTTTAGTTTGGTTTTTATGTGTTTTTTTTAGAAAAAAGTTTCATATTTCATATTTTAGTTTCTAGTCTCGTTTTTTTCAGTTATTTTTATTTTAGTCTCTGTCACTCTTTTACTTACAAATGTCTTCCTAAATCCAACGTATTAGGTTCCTCACCTATAATGTATAAGTTGTTGCAAAATGGTAGTCATTGCACCTATAAGTTGTCATAGAAGAATTTATCCCCAGTTTTTGTTTTCAATCTAGTTTTTTTTATATTTTTTAGTTTGAGGTTTCAATATCTTTTTAATGTCTTTTTAGTTTTTGTCCTTAGTCGCTCTTTTAGGTTCAAATGTATTCTTGAATTTTACATATTAGTTGTATAATAATTTATCCTCTAAGTTAGCATCTTATGCAATGTTTCTTGTAATATTTTGCTCACCAGGATGCAACTTATCTTATTTTTAAGGTCGCTACTCTTGTCTTTTTAATAAATGTCTCTAATTTTTAGTTAGTCATGTTTCTCGGTCTATAAATTGGGTTTTATAATAACTTGCCTTTGCCCCTCTAGTTGGCAGCTTATGCAACGCTAGATGCTGTAAATTAGACTCGATCTATAGAAAATGTGTGGAACATTTATATCTCCAAAAAAAATTATTAAAAAATTAGATTTAAAGATCTTTCAAATGATACTAATTATGTAACATAAATATTAATATTTTTAATATATATTTAGTTAAAATTGTTTCTAGGGAAGCGAAAATGACAAATATTTAGGGACAGAGGGAGTACCATGTAACAACTTTTGTATATTTTTAGAGCAGCAACTATAGTAGTACATGACATAAATTACTACTAGAAAAAATATTTAACATATATGCAAGTGGAATTTTGTTGTTTGTCTTACCGCATAGACACACTGATACAGGCAGAATTAAAAATAAATACACTATTTGAGAGTTGTAGCAATTTAAAATAAATATTTTAATTTTGTTGACCTACGACAGCATGACAATCAGCAACGAAAGAGATATTTTCATGTTGCACCTTGGATCGCTGGTTGAAAATGGAAAGTGGCGAGGACAGTAGCTTTGCCCGTACACATGCCACGGGAGAGAGCCGGTCGCGGTAGGAGCAGGTCGCGCGCTTGCCCTACAGCGCGATGGGTCGCGCGTTATCTGCGTCCAAATAATATAACGTGTGGTTCCGCTCCTTGGCGAAGACTCAGAACTCTTCCAAGGAAAAGTTGCACCAAGTCGTCGACCAAAGAACGGACGCCAGAATTAAGATCCTTCCACGTTGAACGCCCTTTTCAACGTCCGCGGTCTTCCCCATGACCCAACCAAGATCAAGATTCAAGACGGGGATTGCACAGTGAGAAAGTGATGACCACATCAACGCAGCATAACACTACGACTACTC

CTTCCTACTAGTAATCGCCATGACCAATGACCCGTTCTGCTTTCTTACCGCATACAGTGAGTCGTTCCCCCTGCTGGCCGTGTACGTACCACGTTGTTTCCTGCTATATACTCCGCCCTGCCATGGCCGGCAGCATGAAGCACAAATCGTTGCATTGGAGCTAGTATACATAAGTTAACCTCCGAGAGGCTCCGACGACACAGATAGATTCATAGATAGATACGTACCAAGCCTATAGGTGCACAATCACAGAAAACAACACCACCACCCATCATATCGTTGATGCAGGTCGTCCTTACGGCACACCCAGCAGCAGCTCCTCCACTCGCGCTGTTTGCTGGCGTTCTTGACCACCGGTACAGCTAGCACAAAAGGAGCTGAGACGACCGACCATGGACGACGTGCTGCGGCAGATCGACGAGGGGTTCCGCCTCGCCAGGGATCTGATGGAGGAGCTCCCCGCGGCCCAGAACGAGCGGACCTACCTCGCCGACCGCTGTCACGGCATTGTCCAAGCCTACGTCGCGGCCATCCGCATGCTTCACCCGCACGGCGGCACGGAAGACACGGCATCGTCCCCACCGCCGCTTCGGCCGCCTCATCCTCCTTCTCCTCACTTCGGCGGCGATGGTAGTGGAAGCGGCCAGCACGACCACGAGATCCCTCAGCTGGACCTCCTCCGCCCGTTCCTCGGCGGTGCACCTTCTCCTTCGGCGCCGTCGTCGTTCCCGCACAACCTCGGCCGCCTGCTGGCAGAGTCATCGTTCATCAACACCACGCCCGTGGTTGACGCGTTCGGCGCTGGCACATCGTCAGGCGGCCCGGTGAGACGGCAGGCTTCGTCGTCACGGTCGTCACCGCCGGTCCAGCTGCGGCAGCAGCACAGACGAAGGTACGTGCATGCGTTGCCGTTCCTTTGCGTTCCCCGGCTTGGCGTAAATTAGGGTTCTGGCAGCCTACTGTTCGTTATGTTTTGGCGGTCAAGAGTTCAAACCCGGCCTGGCCGAGCGAAGACCCTTTACCACGTCATGGCACGGAATTTTCCACGCACGCACCTGGTATTTGTTCTCCGATTGCGACGCCCCCGAATACGTTCTGCGTGCCGAGTCACGGGAAGTTACACGAGCGTTTACTTGGGGATTAACAGATACTGTTTTTTTTTGGGATTATGAACACTGCTTTGTTAGCTATATTTATACTGATTAGGAGTTGTTGATGGTATCAGGTCTTATATTACTCTAACATGCCTGCGAAAGAGACAGGGGAACTAGTACTAATTAAGATATTTTTTGTTACCCCTTGCTGATTATTTTTAAATTTCAGGAGAGAGAATGGTGAGAGGATGACAATTATGGTTCCGGTCCAGCGGACGGGCAACACAGATCTGCCACCGGACGACGGCTACACGTGGCGCAAGTACGGACAGAAGGATATACTGGGATCAAGATTTCCAAGGTATTTCAATTTTTGAATTCCTACAGAAAATTACTTTGTTATTTGGCATGATTGAATTCTTCTAAAACATCGAAACATCTTTCAGTTACCTTATGATTAGTTGAGGCGAACAGAGGTTCATATTGTTGAGTCAATATTATTGCCAATCTCGTCTACATAGCGTAGTAGCTAACTTGTATGATATTACAAGTAAAAAAAGATTTAACCGGCGGGGACACTTTGTTTATATAATTTCTATAAACATT

TTGTATAGTACTTATCCGCTATGTTATTATAGTACTTATCCACTATGGTACAACCAACCACGGTTAAAGGGTGGGGCTTTAGCCCCAGTGCTCTGAACCGGGACTAAGTGTGGGCCTTTAGTTCTATAAGGACAGCACCGGCTCGGAAACCGAGGCAACAGGCCTTTGCGGACCTATGCTAGTGCATGAACATATAGTGAGTAGTATTATGCTATCGACTGAGCTTTAGAATGGGGAATATACATTAACCAAGAGGATACTCCACATGTTGCAGCGAGTATGAATATTAAATATAAATAGGTTAAAATGTGTATTGTCTAGTTGGACAGCTTGCATGTGCATAGAAATAGGTGACTTGCTAACGGGGATATTACAATTGCATGAGCTAGTGAATTTTAATTGTGTGTGATAGTGGATTGCCTAGTTGTATAGCTTGTATGTTGAGAGAAATATATAGTGGGATAGTGGATTATCTAGTTGGATAGTTTGCATGTTGAGAAAAATATGTAGTGAAGTTTTGCTTTATAAAAATATGTAGTGAAGTTTTGCATGTTGAGAGAAATATGCAGTGAAGTTGATTATTATAAGCGTGCACAAAATAAACCAGAAAATCCCAAAGACTATTTTGAGTCATATAGCGAAACAAATTATCAGAAGGAACTAAAAGGCAATTCTATTACTAAAAAGCTCAAGCCATGCC

TTGTGAATACCTCCATCGCGGCTGTATCAATAGTTTTGCAACCCAAATTGGTCAAGGGCAATCCTCTTCCTTCTCAAATAGGAATTAATACTTGATCTAATAAGTCCTCTTGAAGATAACCTGTAAATAAAATGGTACTTCATCCTTACTAAAAACAATATCAACCATAGCGCCCAGATAATGATACTTGCACCTACAAGAATTTGAGATTTTACTTTTTTGTTTAATCCTTCCAACCAACTTGTAAACAGCTGCTGTCTTTTGGTCACGAGATTCAAGTTAAGCATGCTTCCACATCATTTCCTAACCGCATGGTTATGGGTCATTCACATTTGACTTCATTTTGCCTTAATTGCTTCACTTTTCCTGCTTTCTCACGGCAAAGGCCAACATCAGCAGGTTCAAAAGTCCATATATATATATATATGGAAATTCCTTTCTACACGTACGTGTAGTTACTCTCATCTTCAATAAACTACATTTTGTATGTATTCATACTTGTTTATCGGTTTGAGTATGTTTATATACTCATATTAAGATACTATAGATCTTACCATACGAAAATTTTTCAAAAAAAAATTATATTTTAAATATATTACTAAAATGCCACTAATATATTATATATAATAAGTATAACCATATACTCTATATATGCATGCATACTTAACATACATATCACTCTAGATGGTCTAGTATGATCATATACTTTGTCTATATACATTTCGTTCGGTCATATACACCTTAAATCTAGAGATATATAGATGAGAGTAACTACACACGCGTGTAAAATAAATGCGTCCTATATATATACNNNNNNNNNNNNNNNNNNNNNNNNNNNNNNNNNNNNNNNNNNNNNNNNNNNNNNNNNNNNNNNNNNNNNNNNNNNNNNNNNNNNNNNNNNNNNNNNNNNNACCGCAAAAGTCACATTTATATATATATATATATATACTGCACCACCTCAAATTTGAAAAAATAGTAGTTTTAGCTTAAATTTTACCATATATGCACCATCCCTGATACAAGTATATGCACCCACAAAGCAAGTGCACCATCCTTCAATTTACCCTAGCTTCGCGTTTAAGGTTTAGGGTTTAGGATTTTGTCTTATATTTCACCTGGCTCGTGCGTATCCTGTGTCAATGTGTGCGCACAATAAATTGATTAGATACAAGAAAACATAGCACGAAGTAGTATGAATGGCTCGCCCTAGAAATGGCTGCTTTGTCGAGTGCACGAGACACTCGGCAAAGCCCAAAAAAATACTCAAAAACCTTCTCGGCAAAGACCATTAAGTGAAAAAAACATCAAAAAAGTATTAGAAAGACCCTTGACAAAAAAAATTCTAAAAAACCAAAAAATAGTAATATTTTTATCGGGGGAGGCCCAACCGGCAATTTTTGATGTAGATTTCACAGGTAAGTGACCAATTGGATTCAAACCCATAACTTTCTCCTTGCGTGTAACCACGTCTACCACTACATCACACCATCACATGTGTCTAGAATCCGTTTTGGTTCCTCACATATTATACTCAATCGAGAGTAAATTGTTTGTTTAAGATCCTAAATAAATTCAAATAAAACAGTTGCCAATTATAATATTTCATAACTTTTTGAGATCTATAACTTGGATTTTGTTAGTTTCTCGTCATTTACAAAATTTGAATTTCAAATTTGAGAAATTCAAACATAGTTTTCGTT

GAGAAGGTGACTTAAAATCAGAAAGCTGTCAACTACAAAGTTTCATAACTTTTCAAGATCAACAACTTTCATTTAGGTTGTTTTTCCACCCGAGGTCGGTTGAAAAATTCGAATTTTAAATTTTAAAATTCAAACATAGTTTTGCATAACAAGATGAACTCAAATGAAAAAGTTATCAACTACGAGTTCTATAACTCTTTGAGATCTATAACTTTCAATTAGGATGTTTTTCTATCCGAGATCATTTGAAAAATTCAAATTTTAAAATTTTAAAATTCAAACATAGTTTTGCATGACAAGATAAACTCAAATGAAAAAGTTGCCAACTACACAATTCTATAACTTCTCAAGATCTACAAAGTTTATTTTGGTTGTTTTATCATTCATTCATCAGATATGGTGGTTCTAATATTGTTCACAAATCTTATACATTTGTCTTGTAGTTTCATAAAACTTATGAGAGAAATGTAGATTTTGTGAACAATGTTACTATCGCTTTGTCGAATAAAAAAATGACCACAATAAAAATTATAGATCTTGAGAAATTATAAAACATTGTAGTAGTCTAAAATTTTTCATTTGAATTAATTTACTGCTTCAAATATGTTTTGAATAAATTATTTGCCGAGTGTCAAAAAATAGTAGGCAAAAAACTTTTTTGCCTAGTGCCAAACAAATATTTGGCAAAAAGGTTCTTTGCCGAGTGTCAAAAAACACTTGGTCGAGTGTTTGCTAAGTGCTTTTTCTTGACACTTAGCAAAGAGCTTCTTTGCTTAGTGTTTTTTTTCAAGTGATTTTTATTTAGTACTCGGAAAAGAGCTTCTTTGCATAGTGTAAAAAAACACACGGCAAACCAAAAAGCACTCTGCAAAGTCTTGATTTCGGTAGTATAGTTTGCAACAGCGACATTTTCGCATAGTGAAAACACCACGCATATCCACACTATATCTGGACTAGAAGACACTTGCCTCATAATGGCTAATAACAAATTTAAAATTGGAAATCCTGAATTTATCGCTATCTTCACAATTTACTCAAATATTCTGGCACACATGTAGAAGAACCATCAATAGTGCATATGTGAACAGCCCAAATTTTGTAGCACTGCAGGTTTATAGATGCACCCTTGTTTTCAAAACTCTTAACTATAATTTTAATGAGGTTTTCTTAAATTTACACCGAAGATGTGGTTTGCACGGGATATTTTGCCTTGTTCTACTTCAAAACATGTTCTCTAAATAAAATATATTCTCCGTCCTTAAATATACAGTCCTAATTTTATGCAAAATTCTAGATGAAGTAACCTCCGACATAGTTTTAAAACACAACATTCAATAATAGAGATCCATAGATCATTGTCTGGTCCAAATCTGACATCATTACGAGAATTCGATCCAGTAGAGGGAGTCCGCCAACGACATCGCATCCACGCTGGTGAGTTGAGCTGGAGCAAGAGAAACAAATTAGAGGAGCCAATACAAATTAAGGAAAGATATAAAAATAATGACCTTGAAGAACTTATTTTCTTTAAAATAACTACTTATACTAATGGTTAAAACAGAAGTATATACAAAAGTTGTAGACCATAGGGGAGCCTTTTGGCCCCTGCTAGCCCACTCCCCCCTGGAGTGCTACCACGCTGAGGGCATGACTAGGGAGGATCGCCTCTGCTTGATTAAGAGGAGGCGAGGAGAGTGGAG

GTAGGAGAAGATTAAGAAGAAAAGGCCGTGAGGGAGAGAGGAGAGAGAATAAGCTTATGGGGATGTTGCATTCTGTGTGAGTGAACCACGTTGAATAAAAAATTCAGATCCACAAACTAAGGATCATTGCAAGTTAGTAATACAACCCAATGGTGAACATATAACTATCGGGATGTATTATCAACTGACAGTGATAATTATTTACTGGTGTCAATTGGTTTTAAAATCTCAAAGTAATATACTTTGCTAATGAACCCCCAGTGTTACCTTTATCATTGGCAGTTTTAACTCTTTGGCAGTGATGTGCCGATAGGGGTTGTAGTTTACGGAATAGTGTGCATACTATCCATTTTTATCGGCAACATAAGTATGTTAATCCCAATATCTCCCCACTGTCAAGGAAGTGTTGCATGTGTTGCTTTATTGTAATGTGGGACGCACGTACTCCAGGCACTGTAGGGCGCGAGATGTGCGTAGCCACAGAACGTGCGGCACGCGTGGAGGCGACCGCGACGTCGCGCAGGATCAGCCCACCACCAGTGACTAAATTTAGAAAAGATAATTAGTTTCCTATTATTAGTCCTTTGTTTTAGGAGCAATTAGCTCTCATTCGGTTGGGCCAAGAACCATATATTCTTGTAACCACATATTGGTGAGATTAAGCAAGATTTGTTATCCCTGAGCATTTCCAACAACTTGGTATTTCAACATACTATCCTACCAAATTTGCTAAAAGCATAAAAATCCTCCTCCAACAACTCCTTATCTCAACTTGCTAAATTTCCCAAGTTGCTATCCAGAGGTTTCTACTGCCGAGATTTGTCAAGTTGCTATCCCAAGTTGCTATCCCGAGCGCAACTTGTTGGAGACACATATTCTTTTACCAAGTGAGCGAGTCTCATCTGTCATCCTCAATTTTACCAAGTGAGAATAGCAACTTATTGGAGACACATCCTTTTTTCTTTTGCTAAACAAATTAGCAACTTGCTATAACTCAAGATTTGACAAGTGAATTTTACCAAGTTCTTGGAGATGCTCTCCATCTCTCTCTTCTACGAACAAGCCTGCGGCGGAGGGGCAAACCTCACTGGAGAAGATGGACCGTGGCTACGAACTCCGTAGCTAGGGGCCAGCTATCCTAGAGCCTGACGCCCTTGACAACCAAGTATCACGGTCTCGTTGATCCTCATCTTCCTCAGCCGTCGCGCCAGCATCATCACCACCAGCCGCCATAAACCCTAGCCTACCACCAGCCGCCGCACACCCTAGCCCACCACCAGCCGCCACTGCTAGCACTCCTTTCTCACTGTCGTCGTCAGCCGCTTCCTGCACCGGCACCATGGGGGATGATCTCAAGGCCTCCCTCAAGGCGCTCACCAAGAACGTTGCCTTGATATAGAAGTCGACCGAGGCCAATGGCAAGGCCATCTCCGACCCCTCCATCGCTGGATCGTCCGCGTCCAGCGGTTGGCCAGGGTCTAGGGATGAACATCACCAAGATCGGCCTCCCAAACAATGGTGGCCTAAGTTTCCGCACTACAACAGCAAGAGTGACCCGCTGGTCTTCCTCAACCATGCGAGTCCTTCTTCTAGTAGTAGTGGATCATGCCAGAAGAACGCACTTGGATGGCGTCCTACAATCTCCAAGAGGGGGCACAACAGTGGTACATGCAAGTGCAGGAGGACAAGGGGACGCTGTC

GTGGCTCCATTTCAAGGAGCTCCTCAACCTCCATTTTAGACCGCCGCTGCGCTCAGCCCCCCTCTTCGAGCTCGTGTCCTGCTGGCGAATAGGAACGGTGGAGGGGTACCAAGATCGCATCCAAGCCCTCCTTCCCCACGCGGGCCATCTCGATGAAGCGCAATGTGTGCAACTGTTCACAGGTGGTTTGCTGCCTCCGCTCAATCTCCAGATCGCATCCAAGCCCTCCTTCCCCGTGCGGGCCATCGCGATGAACCCACAATCACTGGCGGCAGCCATGAGCCTTGCACGCCAGATGGAACTCTGGAGCAGTATTGCGTGTCCACGGCCAAGGCACCTAACCAGGGAGTGTAGCCATCTCTAGGATCGCGCCCTGCTGCTCCGTCCACGACCAACCCGCCAGCACCCACCGCATCGGTCAAGCGCCTAAACCAAATGGAACAGGAGGAACGGCGCCGCCTCGGACTGTTCTTCAACTATGATGAGAAGTACAGTCATGGGCATAACAAGGTTTGCAAAAGTTTGTTCTTTGAGGATAGCATGGAGGAGGACAAGGACTGCACCGAGGACACGCCAGACACGGACACGCTAGACACGGACACGTCGGTGTTCTCCCTTCATGCCGTGGAGGGTGTCGCCGTGGGCAACTCGATCCTGCTCCGGGTGCAACTAGGCGTGGCATCGCTCGTCGCCTTGGTCGACACAGGGTTGACCCATAACTTCATCGGTGAGTCCACCGCCACACGCATGGGGCTGATTGTCCAGCCACGACCACAAATGACCACCACGGTCGCCAACGGAGAAAAGGTGGCCTGCCCCAGAGTACTCCGCCACGCATCCATCGTCATCGAGGGCATGAAGTTCCACGTCAATCTGTATGTCATGCCGCTCACTGGGTACGACATTGTCCTCGGCAGCCAGTGGATGGCCAAACTAGGGTGCATTGCTGGGGACATCACCACGTGTCCTGACGTTCTAGCATTGTAGGCATCACATATGCTGGTACGGCATGCCCAACCCCGATGGACCGGCGGTCCGTGCAACAAGCTTCGAGGGCTCCCTCCTCGACAGGCTCCTCGTCTCCTTCACCAACGTCTTCGCCAAGCCCACAGGTCTACCTCCTCAACACGGCCACGACCACGCCATCATCCTCAACCCTGGCACATCATCGGTGGAGGTCCAGCTGTATCGGTACCCGGCGACCCACAAGGATAAGCTAGAGTGGCAGTGTGCCGCATTGATCAAACAACGCATTGTATGCCGCAGCAACTCGTCCTTCTCCTCGCCAGTCCTCCTCGTCAAGAAAGCGGATAGTTCATGGCGATTCTGCGTTGACTACAGGGCGCTAAATGCGCTCACCGTCAAGGACACTTTCCCCATCCTCGTCATCGATGAGCTCCTCGATGAGCTCCATGGGGCGCGCTTCTTCACCAAGCTCGACTTGTGGTCGGGTTATCAACAGTTGTGCATGTGACCAGAGGACGTCCACAAGACGGTGTTTCGCACCCATGGCAGACTCTACGAGTTCTTGGTCATGCCGTTCGGGCTGTGAAATGCTCCGGTGACATTCCAAGCGCTCATGAATGATATTTTTTGGTCCTACCTGTGCTATTTTGTTCTTGTATTCTTTGATGATATTTTGATTTATAGCAAGACATGGGGAGACCACCCTGGCCACCTCCGCACTGTCCTGTCGACCC

TGTGGCAGCATAGGTGATTCATCAAGCGCACCAAGTGTGCCTTTGGCATCGACTTCATGGCATATTTTGGCCACATCATCTCTGCCGCCGCGGTCGCCATGGATCCAGCGAAGGTGTAGGCCATCTACGACTGGCCCCGACTGTGCTCGGCACGTGCGGTACGCGGTTTCCTTAGCTTGGCGGGATACTGCCACAAATTCATCCACAACTATGGCACCATGGCTATGGCCCTCTCCGCCCTCCTAAAAAAGAAAGGGTTCTCCTGGACCAAGGTAGTGATCGCAGCGTTCGATGCCCTCAAGGGCGTGGTCTCAGCACTAATCCTCACCATGCCGAACTTCACCATGCCGTTCACCATGGAATGCGACGCCTCCTCCCACGGCTTTGGCATAGTCCTCATCCAATACGGCCACCCCATTGCCTTCTTTAGCAAGCCCGTTGCACCTCACCCCGTGCCCTCGCCGCATACGAGTGATAACTCATTGGCTTGGTGCATGCTGTCAGGCACTAGAAACTGTACCTTTGGGGTCGTCACTTCACCGTCAAGACCGACCATTATAGCCTCAAATATCTCCTAGACCAGTGGCTGGCGAGCATCCCCCAACACCATTGGGTGGGGAAGCTGCTTGGCTTTGACTTCAAGGTGGAATACAAGCCCGGAGCGACAAACACAGTGGCAGACATGTTGTCAAGCCGCGACACATCCGATGACGCCACTGTCCTCGCGTTGTCCACTCCCTGGTTCCACTTCATCGAGCGCCTTTGCCAAGCCCAGGAGAGCGACCATGCGCTGGTGGCCATTAAGGATGAGATCTACACGGGCGCGCACACTGGCCTGTGGTCCACCACCGATGGCATGGTCCAATTCATCGGATGCCAGTACATTCCCTTGGCTTCGTCGTTGCTCCAAGAGATCATGCTGGCTGTCCATGAGGAGGGACATGAAGGCATCCAGCGCACGCTCCACCGACTATGTCGTGACTTCCATTTTCCTAACATGAAGAGCCTTGTGCAGGACATGGTGCGGGCATGTGGGGTGTGCCAGCAATACAAGGTTGAGCACCTTCACCCAGCCGGGCTTCTGCTCCCACTGCTGGTGCTGCAGGGTGTGTGGACGGACATCACCTTGGACTTTGTCGAGGTGCTTCCCCATGTCTGCGGCAAGTCCGTCATCTTGACGGTGGTGGACAACTTTAGCAAGTACTGCCACTTCATCCCCTTGGCACACCCGTACTTTGCCGAATTTGTCGCTCAAGTCTTCTTTGCTTAGATCATTTGCCTCCATGGCGTTCCTCAGTCCATGTTGTCAGACCATGACCTGGTCTTCACCTCCACATTCTGGCGTGAACTGATGCGCCTAGTGGGCACCATGCTCCACATGACTACAACATTTCACCCCTAGTCAGATGGGCAGTCCCAGGCCGCCTACTGTGTCATCATCATGTACCTGTGGTGCTTGACTAGGGACTGGTTGTGCTAGAGGTTGCGGTGGCTTCCATGGGTAGAATTCATCTTCAACACCACCTACCAGACCTCGCTGAGGGACACCTCGTTCTAGGTCGTCTATGGATGTGATCCCCCTCCATTCGGTCATACAAACTGGGGGACACAAGGGTGGCTACCTTCGCAGCATGGAGGAGCGCACAGAGTTCTTGGAGGACATCTGCTACCGCCTTGACCAAGCACAGGCCACCCAGAAA

AAGAACTACGACAAGTCACACAGTGACGTTCTTATCAAGTCAGGGATTGTGTCCTCCTTTGGCTTCATCAGCACGAGCCAGCATCCTTGTCGCAGACGGTCACCGGCAAGTTGAAGCCATGATACTTCGGGCCCTACCGTATCACGGAGATGATCAACAAGGTGGCCATCCGTCTTGCTCTGCCGCCCTGCGCTCGCCTACACGATGTCTTCCACATCGCCCTTCTCAAGAAGTGGCACGGGACGCAGCCCGACGCCCCACAATTGACACTTCTAACACCACTTAAGCTAAGTTGTCATCCCTATCATGACGCCAAAATTGTGGTATAAAAACTGTCCACCAATAAAATAAATATAAGATCCACAAGCATACAAATTAAATAACATTGTAGCACTTCACCGGGAAGTATTCCAGGTATTATTATTTATATTTAGCTCAAACGAAAGAACTAAGGACTTAAACAATGAGACCAACTTACAAAACCTGAGTACTTATGAGTGATTGGGTTGCATAGTCACATACTTGGTACGAGGGTGAACCAAGATAGATATATGATATAAAGTATAAAAGCCTAGGGAATATAGAGACTAGATATAGACTACTCAGATTAATATTAGGTAGACGTCTGATTAGTTCAAGAGATAGAAAATCCGAAGTAGTTCTAATTTATCAAGTCATTCATCTACTCTAATGCTACACTATCATATATAGCACAAAAGACTCTTCATTTATGTATAAGAAACATTGCTATAAAAAGCCAAGATAGAGCTGGACTTCCTACACAACTGCGGTTCTACCTGTTCTATAACCGGAGGTTGGAATACAAAGGACTCAACAGAACTATCACTTCCATGATCTACCACGTAACTCTGCCAATACGGTGTAATTGCAGATAAACAACATCTAAGCACCATGCTTACATGACATCTATCACCTAACCCTCCAGTGAAGAGAGCTAAGCGCTTTGCAAACATATACATAAACTCATCATCAATCATAACCATATCAAATGTAGAACTAGAATAAACTAGAAACATAATAAATAGAAACATAATGATATTTAGAATAAAGTCAAAGAGAGATAAAAGATAATACCAATC

TTCGATGAAGATCCAGAAATTCTGAGCACAGGCGCCCGACTCTTCCTCTATCTAATCTAATCCTATGTAGAAGATGAAGATTAGGAGTAGCTAGTCTATCTCTAAACCCTGGCTCTAATGGCTCCCTCTAGTTCTTAGATGAGAATAAAAATATAATTATGTTTTTCTCTCTATTTTGAGCTCTCTCTAGGGGGGCAGGGCTATATTTATAGCCCCCTAGAAAGCACCACAACCCTTGGATCAAACCGACTTAAACATGCGCTTAACATTAATCCTCAAAGTCGGTAGAGGCAGGATCCGTGAGGTTGGCTCTGATTAGCCAGGCTCCCCGGGCGGCCGCCCCTGTCAGGCCTGGGCGCCCGCCCTTGCCCTATGGTAGGTGGGCCCCACCTTTCAGGTGGTGTCTTTTCAAGTCTTCTAGAGTATTCAGGAGTTGACGTTCAGGTCTTCTCTTTATGTAAATCTGACATGTGAAGCTTTTTTTTGTTATTATTCTTATAACCCCTGCAGAAACAGACATTCACCAAAATTTGTGGAAATTGTCCGTAAAAACCCTATGTCTATAGTGATGTTCAATTTTTACCCCCTTTCTTCTCCTTAGTTGACATATAATTTTGATGGTTAACAACCATCAACACCCACCTCCTCTAGCAAGCGTGCACCACGGCACGGTGGTGTGTGAGCCTGAGCGGGTCATCAAGTCACACTTGGCTCGTGGGGTGCGTCAAATCCTGGTTCAATGGAAGGGGACCTCTGCAGCTTCTGCAACATGGGAGGACATGGAGCTATTATTCACCCGCTACCCTGCACTACAGCTCTAGAGGACGAGCTGTCTCTCGACTGAGGGAGAGATGTCTTGTGGAGACGCACATAGTCCAGCTGCCGTAGGGCACGAGATGTGTGACGAGCCACGGAACACGCGGCATGCGTGGAGGTGGACCGCGACGTCAGTGGCTAAGTTTAGAAAAGATAATTAGTTTCCTATTATTAGTTCTTTGTTTTAGGAGCAATTAGCTCTTATTTAGTTGGGCCAGGGGCCATATATTCTTGTAACCGCACATTGGCGAGATTAAGCAAGATTTGTTAACCTATATGAACAAGCCTGCGACGGAGGGGCAAACCTTGCCGAAGAAGACGGACCGTGGCTATGAACTCCGTGGCCAAGGGCCAGCTATCCTATGGCCCGACGCCCTGGACATTTATCTCATTATATGTTTTAGAATTGTTTTTTAGAGATATATAAGGAATCAACCCCAGTCTTATAGCATAAAGAACGGTTATGCATATATATAATTAATATATAGTTACTTGTAGTGTAGCATTATTGCTGTCATTTGATTTGCAATTTTACATGTTGCTATATATCGGCAGGAGCTACTACAGGTGCACTCACAAGAACTACTACGGGTGCGAGGCGAAGAAGAAGGTGCAGCGCCTAGACGATGACCCGTTCACGTACGAGGTCACATACTGTGGCAACCACACCTGCCTCACCTCGACGACTCCGCTCCTCACAATCCCAGCAGGCCCCGCTACCGTGGCCTCAACAGCTGCCAACATGCTAAACAACTCCCCGACTGATTCAGCAACAGCCCTCGCCGCCGGTCATCAGGACCTCTTCATGCCGGCCGCTGAGCATCCTGCACAGGCGCTGTCCACGGCCATCCAGCTCGGCATCAGCTGGATGCCATCAACCCTCG

TTGGCTCCAGTGCCCCCGAGGGAAGCAGCTCTCAGGTGAACGTGCCCGCCTCAGGAAGGGACACCGCCGAATACCCGGTTATGGACCTCGCCGATGCCATGTTCAACTCTGGCAGCCGCGGAGGGAGCAGCATGGACGCCATCTTCCTTGCTCGTCATGATCGACGTGATACCTAGATCCATCTGATGCATCCGATGATCAGTTTCCGGTGGGGATCGATCCTTGATTCGAGTTTTGATCATCACATGACCGTAACTTCGTACAACAACCATGTAGGTGTAGGCATGGAATATATAGGTTCAAGTTTGTTCTTTGATCTTAATTTAGCCACTTAAATCGCTACATAGATTTGTTTGCGGCAAGGCCGTGCTAATAAAGGTGCTCACAGTTAATAGGTATTGAAAGAATAAGTGTACAGGACATGTGTCCGCCCCTCTTGTACATATAAAAATCAACGACGATATTTGTAATTGTATAACTCTTAGGTTCATGTTTGTTTGCAAATAAACATCGAAAATAGGATACATAATTTTGCTCGAAAGCCCTTATGGAAAAAAATTGTATGTAAAAAAGCATTAAATTCATACAGACG

>SbWRKY11

CCCTCCCCCCACCCCAGGCCGTCGGTGAGCCTGGGGCACGTGACAGCGATACGCCGGAGGCCAAGATAATCTTTGCATCGTTCTCGGTCTGGGTTCCGGACAGTTTTTATATTGCCGCGAGGTCTCCATTATTATGGAGTTTCTCTCCCGTCCCGTCCCGTCCCTTCCCTTCTCTCTGTGATGTGTTGTCATTAGTAAGCTGTTAAGCTAAAGGGCTCCTCCTCCTCTCTGCTTTGCGCTGCCCGGTGTGCTCCTGCTGTGCTGCGGTGTCTGGTCAAGTACGCTTCCGAATTAAAAGCGCTTCCGGCCTGCAACCTGCTTGCTGCCTCTCTGCTCCCACGGCCGGCCGCCTGCCCGCGTCTTCTTCCTCACCCCTCTCCCTCCTGCGGTCCCGTCCCTGCATTTCTTGATTCCTTCCTTCACCGGAACCAAGCAATGTCTCCGGCGCCGACTTCGCATCACTCGCAGATCAATTCGAGGTAAAACTAACTGCGACTGTGTTTCGCCTTGTGATCTTCGCACCTGCCTGCGCGCCGGTTCAATTTGGAGGGGGCGCCGCCTGATTTCGTTGTCTCTTGGCAGGAAGGAGAAGCGGATGAGGAAGGTGGACACCTTCGCGCCGCACAACGACGGCCACCAATGGAGGAAGTACGGCGAGAAGAAGATCAACAACACCAACTTCCCCAGGTCGGTCCGGTCCGGCGCTGATTTTACTTGTTTGTTTTTAGAAAAAAAAACCCAGATTTTACTTGTTTTGTTTCTTTTCTTCCTCGTTTGTTTTTTACTTTACCGGGGGGGAAAGGGCTTCTGCATCTGCATGTGTTCTGAGAAATCTATCCTCGCCAGCAAAGAATACGCCTTTTATCGTATAGCCAACCTCCTTTGACTTTTGCTGTTCGTTTCCTTTCCTCCCATTCTCCGCATCCAAAATCGGCGGGCCAAAATTTTCATTCAGCAGATGTTCATTCCACTCGCTCGCCGCTCACACACAGGTTCTTCTTCTTCTTCTTGTCTCCGGTGCTGCAGGTATTACTACAGATGCACGTACAAGGACAACATGAACTGCCCGGCCACGAAGCAGGTGCAGCAGAAGGACCACAGCGACCCGCCATTGTACGCGGTCACCTACTACAACGAGCACTCGTGCAACAGCGCCTTCCTCCCGCTCTCCCCCTCCGAGTTCCAGCTGCAGACCTCGTCCGGGAAGGCCGTCTCCATCTGCTTCGACTCCTCGTCCGGGGCGGCGGCGCCGCAAGAACCGCCGGCCACGGCAGCGGCCACCAATGCCAGCGGCGGCGGCGGCTCGCCGTCTTCCAGCGCGGCGGCGGTGGCGGCGGCGCGGAGAGGCACGCCGCCGGAGATCAGTAACCCGCCCGTGCTGCGGCGGTCCGAGACGTACCCGTGGGGCGCAGGCGCCGCCGGTGTCGTGGAGCAGAAGCCGGCGTCCTGCAGCACCGAGTGCCACGACGCCTTCTCGGGTGCCGCCGGCGCCGTGCCGGAGGAGGTGGTAGATGCAGGCAGATTTGGGTCTATCAGGTTCTTCCATTTTTTGTAAATTGATCAGTGGAAGACAATTGATTTTACTTCGACAAACTAATATGATACAGATGTCTTCTTTTCTTCTTCTTCTTTTTGTTCCTTTATTACATGCCGAAGCTTACTAGAGATTCGCATGTTACTTTTGTTTTATTGGTTCGAAA

TCGAAAAAATACCACTGCTAATTTCAATCCAACAGTTCATTTATTA

>SbWRKY56

TTCCTTGGTCCCAACGAGACCTTCCTGTTCAGGCCTCAATCACACAGAGCAATTGAGTATCCTGAATGGACATCAAGAGCAGCCTCATCATGCTGCCGCCCTGCAGCCATGGCTGGGAGATGATGGAGACCATGAGGAGACAGCAGGAGCTCGTGATGCAGCTCCGAGCGTTCGTCCTCCCGCTGCTCCCCGGCGTCATCGTCGATGGTACCTCGGCCGCCGAGATTGCTGTCCAGCTCTTCGACGACGTGATAGGATGCAACATAGGCGTGGTGTCTACGCTTGAAGGGTGCCTCGTGAGTACCGGAGCCAGAGGCGGATCATCGGGAGAGCCCGTAGACAACAAGTCGTTGGTGAGGAAGAATAGCTGCCATGTTACTGAAGGCGAGACGACGGATGAGCAAGCGAGGCATAGAAGCGTTGTTGGTCAAAAGAGAAGGTGTGCAGTTAGATATTGATACCTCAATATAGCTCTGCTCTTCCCTAGCTGATAATTAACTTGCTTATAATCATATTATATGCGATTATTAGAGTTGGTAAAACTAACATTTAAGATATCATGCATGATACTGTCATGCATATGTTCAGCCATGCATATATAGCCATATAGGAGGTGTTAATTAATTTGGAAGCTAGCTAGCTTAACACCTTTAATTTGCATGTCCATCAAGTTATTAATATGATCTGCTGGGCAAATTTCAACTGATCATGTAACATCCATGCATCATCAATTGATCGATTTTTAGTTCATACATACTGATCCGACATATATATGTATCTAGGAGGAAGAACGACAAGCGATCACGATCCCTTGTGACGCATGTTCCACATTATGATGGCCATCAATGGAGAAAATATGGGCAGAAGAACATCAATGGGAGGCAGCATCCTAGGTAATACATCTATCTATATGTACGTTACATTCTTTTATTATCAGCTAACAAAACAACCTACACTGTTGCGGTCAAAAAACAACCTACGTTGTTATTAGGTATAGCTTGTTTCATTAGCTGATGATCTCTAAAAGTTCAATGGACACAAAATATATAATAATCAATATATGGCATGATGACCTTGTGATCAATTGAGCAGGAGCTACTATAGATGCACCTACAGAGAACAGAACTGCTTTGCAACGAAGACTATCCAGCAACAAGAACAAATTGACAGTATCCGTCGTAGTGCCACGCCTGGTGAGGAAATTGCAAAGTACACAGTCGTGTACTACGGTGAACACACTTGCAAGGACCATAGCATCAGCATAGTCCAGCTTCCTCAACTTGTCAGCTGTATGGATCTTCAGAACATGGAAATTGCCCAAACAAGTTCAGATGTTCAAGATCCTGAGGCAGACTTGGACTTGCCAGCTTTACTAGAGGTGTTTGATAACTCTGTCATTGATTGGGAGGATATTTGGAAGATATGA

>SbWRKY53

GGGAGGGTGTGGGTGAATTCTGAAGATAATGGAGTATTTGAAATGCGCGGATGGGTTCAAGGAGGTTCAGAACGAATTCACCCATTTGGACCTCCCCTCAATCTTAATTGATTGGGACCTAGGTACTACTGCACTACAAATATAATTATATTTTTCAGCAGTTTGGTAATTGAATAATATTAACGCGGCCGCCTTCTTCATTCCACACAAAAAAAAAGAAATTAAAGAATGGTTGGGTGATGAGCTGTTTGTTTGTACGTGGATGTGTTTGAAGCAAATAAACTAAGCTTGACTGCTTGAATCTGTCACCAGAAAGTCCACTAGCTCTCCTAATTCTGAAACAAAATAATGCACGAGATGATGCGGCTACTTCGATAGCCACGATCTCAATGGCTAAAATATTGGTATCATCCAATGTTGTTAAAACGACAAAGTGTGTTTACACTGATCTGTGCTCATTGGTAGCTTTAGCGTAGTATTTAGTTTTTAGCTCAGAATGTTAGAAGGGATATTATCCATGTTTTAATCTAGAAGAAAGATGTTTAACTTTTTAATCCCAAAATAAAAACCATAGAACCAAGCAGCAGTCTCTTGTTTAGGTTAGAATATCACTAATTTTGTTGCCAATGCATGCCATGTTTAGATTTAGATGGAGATTATTTGCTCATGAACAAGTGATAGTTAATTAATAAAAAAAGGATTACAAGCTACAATAAGATGAATTTTGTAGATTTCTTCCCTATTTGGAGAAGTGAAACACACACACACACACACACACACTGCTCCATTACTAGTGCTGTTTTGCTTTAACCTTTAGGAATTATGTATCTGATTTCAGAAGAGTGTAGAAAGCAACTTGCAACTAGTTAAGTGCTTTCAATTTTATCATCATTATGGAAAAGCAGGAAAGACATATTTAAACCATGTCATTTTCAACTGTTCCTAGCTAGAGCATTTGTCTTCTGCATAAGGTCATTATATTAGCACATGGTAGCTAGTAATACAGCTCAATGAGGAGCCGTCGACAGGATTTTATAGATATATTTTCTTTTCTTTTCGGTCTTCTCTCTTGGGACTTGCTTTGAAAAAGACTACTTGGT

GAAAGTTTGTTCAAAGCGATGCACTATATTATATATAATAATAAGGTTAATTTATTTATTATGCTAGCCTGTGGCGGTGGGGATTCAGCCACCAAACCCAAGATTTTATAGTGAGGCTGTGCCTGCTTTTAAATATTTGTAAGTATAACAAATAATGTAAGACTAAGCTGAGAGTCTTCAGAAATGTAAATAAATGCAGAGCTAGCTGTCCTCTCCTGCACATGGATTTGTATTGTTAATGGCCTTCAGTCCTTCAGATTTGTTTTTTTCTTTTTTAATAACAGTCATTCAGATGTGGCTGGCTCTTTGATGCTAGCAGCCTCACTCCGTCACCTCTATCCGTTACTCATTACAGCTACTAGAATTAGTCCTGAAACTACTATAATGACATGATTTGGACACGAATTTAAATAAATAATAACAAGTTAGAAACACATCACACACGTTTTGTTATTCGGGAAAGTCGTTGTTATGTAACCAGGTTTGCTTCTTCTTTTGTATTTAAAAGCATATCTTTTTGTATTAAAAAAACTTTGCTGTCTATTATTTTTAAAAAGTTTTGCTAGGTGCCTACTGTTGAAAAGATACTATATAATATGGCTGCTGGCTATTATTTTTTATTTTAGGGATAGAAAAGATATTAGTACTCCCTCCATAAAAAAAATGTAATTCTCATTTTCCGAGAAGTCAAACAATCTAAATTTCAACAATATAGAATACAAACACTTATGATATAAAATAAAATAAGTACTATTAGATTAGTTATAGAATATTTAAAATAAATTTATTTAGAGATGACATAAATAACTATTGTTACAAACTTGATCACATTTATTAAGTTAGATTGACCGGCATAGATATTATAATTGTATTCTTTGGTGAAGGAGAGAGTATTTGTGAAGAAAAAAAAAAGAAAACAAATAAATTGATGAAGACGAAGAAGAGTGGGAGCGGCGTTAAGCGACGCGTGTTATTAACGTTGAAGAATGCTGCACCGTCGTTGTTTAATGCCTATAAATAAAGGTGGTCAGAACCGTCGTCTCCAACACCAACAACAACAACGCAACATCCAGGCCGCCGAGCCGAGCTCCCGCAAGAAA

CGCCCTCCCTCTCGCCTCCGCCGTCCGCCGTCCACGTCGCCATGGCTCTGGACTCGGTTCCTCCTTACCCCTGCGACCTGGGCTCCAGCAGGGCCGCCGCCGCCGCCAGAACCCAGCAGAGGATCAGGTACGCAGCGTCCTCTGTCGATTCCTCCTTGCCCCCCCCCCCCCCCCCCCCCCCCCCCCGCCGCGAGTTTCAGTCAGCTTCACGGTGCCTTCTGTTCCTCAATTTCTGCCTGTTCTATATATATAATCGGCAGGAAAGATGAGCGCACCTGGACCTCGGACACGTACGCGCCGTACGACGACGGGCACCAGTGGAGGAAGTACGGCGAGAAGAAGCTCTCCAACTCTCACTTCCCGAGGTATGTTCGTTCCGTTCATCCATAGCCCCTCTTGCCTACAGTTCGTTACCTCAAATTAGCGTCTTTTGTTTCAGTTATGTGTCTTAGACGGTCAATTTAGTTCTTATTACCAGATCTGGAGTGTGTTTGTGGGTCCTATTGCTGGTTCAACTGGGGGTGGTTATCCCAACTCTTCAGAATTTATTTAAGAACAAGTTATTGCCTTATTTTGACTGCAATAATTCCTCATTTTTAGTATCTTTATGTAGGTACTCCTGGCTTCTATTAGTATAAGTATATAACTGTATAAATAAAGATGAACTGAAAGAACTGTGCATACTTTTCCTATCTCTAGTCTAGTGCTAGTATAATTTTCATCTTAAATGAAGCGTGTTTGCCGGTGCAGTAGTTTTGTGGGCGTGTTCACCTCGTGATGGCACATGGAAGAAGGTCATTGATCTTGTCTTGTGCTAGACTGCTAGCTTGAGAAAATTGTTCACCAAGGCCCTGCTCGCTTTGGAGTTTGGAATACCATAGGCGGTTCAGCGGTCTGAGCGAGTGGAGAACAAATCTGAAACTGACAGTGGGTAGCAGTTGCAGACATGTTCATCTAAGCTCCAATTTTCACATATCTAAAATCAATCTAATGTTGCTACTATTCGTTGTCCGCAAAAAGAAACAAATCTCTTGCTTCTGGTCAATATATAGTCTTTGTATGTTCAGTTTGTGGGCCAAATACTTTTTATTTTCCACCCC

AAATCACATTGCCGCTTAGTGCGACTAATGTTTGAATATCAGGGGAGACAGACATATCAAATTGTGTGATTGTCTACTAATCACTCTCTGGTGGAATTGTCGGTGGACAGGTGTCGCCGTACCCTTGAATGATTGAAATAAAAACAAATGCTTGTGTAATAGAAAAGTCTTTTATGTCGTGACTACTGGAAAAGAACATTCAAAGTGGAAATCCCTTTCCTAACTGACAACTCAACTAGGCACATAAACAACTCGTAAAGTGTTACTTCAGACTTCCTCTTAACACTTCTTATATGTGAATATACAGTTCACAAGTTGGCCACCTTTAGTACCTGTCATTTGCAACTCGTTTGAAATCTAATACTTCACTAGCATACTAAAAGTTGCCTCATTTGTTTAGCGTGCATGTTGTCCAACTCAAGTCTTCAACTATTAGTAGTATCCATGCCGACAGTCTCCGGCAGTCCTTTTTCTGCTTTTGTAGAGAGCGATTGGAATGCAGTGCAGACCCCCATATATGTTGTTAGCCCATCAAGAGCATGTTAATTGATGGGGAGCTAATGTTTATTTTCTAAAAGGCAGTGGACGCAGTAGATGAAGAGGCCTCAAAAGTGTTTGTAGATGGTGTTACACCTAATTTAGGTAGACTGAATGTTGACAGAGGGGGGAAGGACATGATGCATCTTTATTTTCCAAAATACTCACCCACTTACTGAGTTAAAGGGAAGTGGACCTTTGACATATGGATGCTATTGCAGAGTCCTTCCAGAATTGTTAAGAAGAATAAGTGGTACACTAAAGTCTAAAGAAATCTTTAAAAAGGTGTTGTTCAACCTTCTGAGAGGAAATTTAATAGGACCTTGTGTTTTACTCACTAACTGTCGGTGGTCTATCTAAGTTCCAAACACTCTAATAGAGAATTAGCGAAAAACTGAGAAACTTCATTTTGACACTAAATAGATCAACTCAAAGTCCCTTACTGTCACTTTGTGGTGTCCTCTTGTTATATTATTATTAGTGAAAGACATGCAAATACTCATCTCAAAACGTAAAAAAAAAAGATGGAACAGAGTATTCTCTTACATGTGACTATTATGAATGTTAACTTCTTGGTTAAAGTATGTACACTGCATTTAGCATATTTGCAGATAATTCTTCTCTTCCCTTTTGGAGTGAACTTTTGGCACTGTCATCCTATTTTCAGGTTCTATTACAGATGCACCTACAAGAACGACATGAAGTGCCCCGCGACGAAGCAAGTCCAGCAGAAGGACACAAGTGACCCCCCATTGTTCTCTGTCACTTACTTCAACCATCACACCTGCAGCACCAGCTCAAGTGCCATAGGAAGCGCTAGAGACATCACGTCGCAGTCATCTTCTAAGAAGGCGGTATCAATCTGCTTCAGCCCACACACCGCCTCCGAACAGCCCTCATTCCTGACATCCCCGGCCATGCCACAGTCAACGATCATGCACCCTTACAGCGCCAACCAACAGTCTGACAGGAGCGCCTACGCATACCAGCAACTCCAGTGGACAGGCAGTGTGCCATCTCACGCAAGTAACGGTCCTGCTAAGATGGAAGTTGATGACTCTGCGCAGCCAAGCCCTTCATCCAGCAGCACCAGTGCTCTGTCGAGGACTCTGCTACCGATCGGTCAGTCCAGATGCATCGAGTACTTCCATTTCTTGTGATGA

ACTTCAGGGATTGAAGAAGCAGTGTTATATGCTGGGCCACTGCCTCTCGGTGCCTACTTGGAGTGGCAAATGGAGTTTGTATCTGGAGTTGGAGGCGACATTGCCATTCCGGGCCTGAAGCCTGAACTACACAGGCCGGACCGGGGCCAATATACTGATCTGCATTGCATAGAGAGATTGCCTGCTGCTTACTTCTTTACATTTGGATTCAAGCCACGTTTTTGTGCTTGAATGATGTGAAGAATGGTATTGGACTATTGGGGTTTCTTTATCTAGTAACGTACATGATATGATACGTACAGAATGGGTGCTTGACTGTTGAGATAGCTCCATTTGTTTGTTTAGAGACTGATACTAGTATGTTTCGGATTCCTGACAGCAGATTTTCAGTCTGCTACTACTGAAATGCTGAAGGTTACAGATTCTATACAGTCACAGTTTTATTAAGATGTCTTCTACAGTTTTCTGTTTCTCTATCAAAAGAACATACAGTTTAGTTTATGCCTGGATGTTGTTCAGTTCATATTCTGAACATGATGTCTTACAAATTCGTCCAAAGTTGAAGTGGAGAGTTGAGACAGGAGTATATGGTTTCAGACTTTCAGTGAGCTTATTGCAACACGTGAGCTTAGCGTACACGAATTACGAGTGGAGCTGAAACCATGGCAAAACCATCTACGTGTGCTGCTGCTGCCCTGGTTGGCAGCCGAGCTCGCACAGCTCTCGCGCCACGACTCGCCGCCAGGACGCACCGCCGCCGCCGCCTAGTCCGCGAGTTGCTTGACCGTGGATGTAGTGGCTGGCGAACCGCTTCTTCTTCCACAAAGATTCAAACAAAACTGTTCTTAGATAGAACTAAAGAAGATTTGGTGAACGCTTGCACACTCTAAGTTCAAATTCTCAACTTGACATGCATGCTTATATTTTTCTGATTTTATTTCAGTGTTGTGTTTTTAGTGGTTGACGATGTTCTCATCGATGACAAGACGTATTTAGTGACTTCGTCGATCTAGAGATGTATCAACATAGTTTTTTGGAGATGTTTATAGGGGTAAGTTTGTGTACGTGTGTTCATATAATCATATAGGTGAGTGTTTTGTGTGTATGATGACCGTTTACTTATGTAATTAGCAAAAAAAAGAAACAAGTTTGTTTTCTCCATTTCTTCTCTGCAAAAATAGGTCAGTATTGGAGAATATTGGCAACTTTTAACTGATCTTTTTTACAGTTGACCCAGGGTCCATGGACCTGGAGAAGTTCATTCAAGTTTAGTGGGCCTGAAGACCCATTTCGTGTATGCTGTTGACTTCAGAGTTCATAGACCGTCACTGGCTGTAATGTTGGGCCTGCCCATGCAAGAACGTGGGCTCAGTCTGCGCATTTGGGCTGTAGCGCTGTGGGCTAGTATCCAGTGGACGTGCGGAGCTCTGGTGGACTGGGCTTAAGATGATTTTTTCCCTCATTTTTTTTTTTGAGGGAGTTTGACCGGGGGCTGTTCGGGTCCGGCCGTTCGGACCGAATCTGTCCCCCGCCGGCTGTTTCCCGCTTTCTCGCCTGTATTGTTTCCCACTGAGACCAGGCGCGTCTAAATTAGATCTTGTTTAGTTCTAAAAAATTTTGTAAAATAAAAATAGTATCACTTTTGTTTGTATTTGACA

>SbWRKY71

AAACATTAATCGTAGCAGTCAAAGAAATGTATTTACTTGTGGGATGAAGGGAGTAGATAATAAGGAAAAAATATAGAGACATGTAAGATGATTTTTTTTTTTGGATGGGCTCGCGATCCCTGTTGGGACTGGGCTGGAAAACTAATCAGGCAGGGCGCAGGCCTCATGAGCATTGCCGCTGATCCATCACAGCCAAGCCTGGCCGTGAGTGCCAACTTCGTCTAGCAGCCCAATTGAGGCCTAAGCCCAACAAATGGTTGATGCCCATCCGGAAGCCGGCCCTAGCCAAGAACTGCATCTCGCATCCCGCCTCCTTCCCCAGCTCCGGCCAGCCAGTGGATCGGTCTCCGCCGGAACGGCCGACCAACTCGGGCTGCCGACGCGAGCGCGGCGCGATGGCGTATTGTGTGCGCGCGCCTTCTAGCTTTTCCCCTCCCGATCCGGCTGATGGCTTTCGAGATGCCTGCACCCGGCGACCGGTAATTTGGACTTGTTTTACTGCATAATTTGGCAACTTTTCCAAATTTCCATATGCACGCGTGTGCTTCGTTTCTGAGTCCTAATCTAGTAGTACAGTAGCATACTGGCATGTACATCTACCCAACACCGTTTCATTCGAATTAGCACGTTTCTGGATCGTTGCTTAAATGTATCTCGTTTGATTTGATGCTAACAATCCATCCCCATCCATTTCTCTTTTTCTCACCGACAACACCCAAGTCACTCTAGTACTATCCTATCCTATTGCAGCCACAAGTCTGCAAATATAATATTGTTGGACTGCCGCAGCAGCAGGCTCCAGGATACATTAGATTATTACTAGTACCTAATTATAATTAAGCTGCCTCTCTAGATGCTGCTTCTCCTTGCATAAACTAATTAACTAGGAATCCTCAACGAATTCAACATCTGATCTGACAGCTAAATTGTCTTCCCAACAAACACTACTTGACTCCTACACATGGGGGGTGGTGGTATATGTTATCAATCAAAGTAATATTATTCGAGTGCGGCGTTCATCGCCTTTGAGCACAAATTCTTTTCCTTACGCGGAGTCAGACGCGAGATACCATTTGCTAAAAAGGAAGGGTTAAATTCAGTGTGCGCGGTTAGAATTAGAACCCGCGATATTACATAGGCATCCAATTGTGATGTTTCCGGCGCTGATATATGGTACCAGACTACCAGGGGGGAGGAATGGTATAATATATTCCTTGGCCTGGCTTTATTTTTGTTATTTTAGCTTTATCCTGCTGTCAAAGAGATCAGAAAAGAAAGATTAGCTACACAGATCGATCAGCTGTCAAAAGATAGCACTGTTCAATTTCAATCCCAGGTAGTACACTTCTGAATTGATCTAAGCTTCCAGGAACCATCCCACCGCCCCAAGTATATATTACAGAAAACTGTTTACATAAACAGCGAGATTCTTCGCAAACAGTGCAACAGCACATGGGAGCACCCTGTACCAGCATCAATTTGCAGATATAGTGGATTAATTAGGTTGATATCGTCTTTGTTTTATGTCCCCCATTAATCTCTAATTATCCAATCAAGGGATGCTCAGTCCTTCCAGAGCATCCCTCCCTTTGTTTATGACCCTTTAGCAGCCAGGAGGATGTAGGCCAGTGTCATGTTTGGGTAAGGGCTGGTGCTTTAGCTTTGGGGCAGCTAACACATCAGTAGTCTCAGGTTAGGTT

GTTCAGTCTTTGCAAAGGGCACGAATGTAATAAAAACATAAAAAATAAAAAAACAAACAAACACACAACAACAACAACAACAACAACCCGAGCCATGGAACTGGCTTTGTATTCGAACTGTTCTATCTTGTCCTAGTTTTGCCATTCAACCTTCATGGAGTACCCGTTCCTTAAATATGGCTCAAACATTACTTATGTAAACTGTTGGTCGTTTACCGCAATTTAGATGCAAAGGGTAATTTAACAACTTCTGAAGGCTTAGTGAAGAGCAGAGAATTATATTCCAAGAGTTCTCTGTTCTCTCGATTTTCAAATATATGTGACACTTCTGATGAAACCATGCATAGTCAGGGAACATTTCTTTTTTTAGGGACTGTCGTCCATTCTTTTGAACAGGCAGTATTTTTCGTTTTCTGAAGGAAGTATTTGGGGTTCAGAGTTTGCCCATTTGTACCTCCCGTCAGCTTGATTGAGGCTAGATATGTACTACTGCATTACAGATATAAATTTTTACAACGGTTTGGTACTAATAATACAAGAGGCCTCTTCTTTATAGCATAAGAGAAGAAAGGAAAAGTGTAGTTGGGTGAGCTGTTTCTCTGTGTATTATATTGCATGTGTTTGTAGCAAATAAACTAGGATTGACAGTTTGAACCTGGTGACCAGAAAGTCCACTTGGTCGGCTGATTCGGCAAAAAAATAATCTGCGAGATGATGCGGCTACTTCTATAGCCACTATCTCAACGACTAAAATATTAGCATCGTGTAATATATCGAAGTTGATCTATGCTCATCGATAATGTAATATCAAATTTAGTGCACAAAGTTGACCTGCTAGATGATGAAATAATGAGAAAAAAATTATGTTTCATAAGAGAGAGATACCATTTATTTATTTATTTATTTATTTACCTAAAAAACATAGGCACACTAGGGAGTCCGCACATTATTATATCACTAGTTTTATTTCCAATGCCATGTTCAAGCAAAGTTTATTCGCCCACAAAAAGGAAGGGACAATAATAAACTCATTATAGGTAATTATAATAAAAAGGGAAGCCCACAAGGTGATAATTACTATAAACTAATTACAAGTTACACTAAAAGGAAAGCCCTACTGAAAAGTGAAAATCGATCACTGCCCCACTAGCTGCGGTGTTGCTTTAGGATTTGTGTATTTAAGTTTTGGAAGAGCATAGAAGCACCTCACAACCAGCCAAGAGATTTAAAATTGTATCATTATTCGAAGTTGGAAAGAAATATGTAAAGCATGTCATTTTCAACATACCTCTAGCATTTGTTTTCTGTCCATGAGGTCATTAGCAATTTAGCATGATGACTAATACTACTTAATCAGGAGATGCTGACAGGGTTAATTATCTTCTTTCATTTTATGCTTCCCTCTTGCCTCTTGGGACTGCATTGAAAATGACTAGTTGGTGAAAGTTTGTTCAAAGGGATGTACTATTATAAGTTCTTTATTTCCTCTCTATGCTAGCCTGTGCCGGTGGGGATCAACCCACCAAACCCAAGATTTTGTAATAAAGTTGTGCCTCCTTTTTATTTAACAAGTAATATAAGACCAAGATGAGTCTTGAGAAATGTAAAGAAATGCAGAGTCAGGGCTTTGAGCTAGCTGTCCTCCTGCACATGGGTTTGTGTTGATAATGGCATTTACTTAGTCAGATGTGGCGGGCTCT

TCTCCAATACTGCTTACTGATACTAACATTATTCCTGAAACTATTTGACTGACATCTAGCGATAAAAAAGGGGAATATGTAGAAACTCTTATTTTTCATGATATTCTCTCAATGTGTTTGCCCAATTTTATTGATCTCAGCCTGATTATTCTTCTGTTTTTTCTACTCATTATTGCTTTTGCATAGACAACGAAACAAAGCTTGACATATGCTCTTAGATACTCTGTATTTTGCTTTCAAGGATATTAATGCTTGTAAACTTTAATTGGTTGCAATTGCAATAATCCAATAAACAGGACGGCGCTTTGTTGTTGCCATTGAGAACAAAATGAAGGAAGAGGATGACAACCAAAAAAAAAACACTGCAGTGCCGTTAAGTGGAGTTCCAGCTAGACAGAGGCTGTGACGCGTGCTAACCTTGAAGAATGCTCCTGCACCGTCGTTGTTTAATGCTATAAATAAAGGTGGTCAGCTCTTCCAAGTCCCAGGCGCAAGCTGCCTAGTTCTGTTCTGCCAAGAAACGCCTCCTGCTCTCCTCCCTCCTCCATCATCCTCGCCATGGCTCTCGAGTCTGTTCCTACATATCTCAGCGATTTGGGATCCCACCAGGCTGCCAGAGCCCAGCAGCAAAGAATCAGGTACACTTCGTTGTAGCTTTGTTCCTAAATTCTGCTGTGAAATTTGTTCGGCTTCATCAGTTTCACTCTTGATGCCCTTTTCTTCAATTTCTGTTCTGTAAAAATAAAATTGGCAGGAAAGATGAGCGCATCTGGACCTCAGACACATATGCTCCCTACGACGACGGGCACCAGTGGAGGAAGTATGGCGAGAAGAAGCTCTCCAACTCCAACTTCCCAAGGTTTTTAGTCCCTCTTGGATTCTTCATCCCCTTGCCATAGAGTTTTATTCTCCAAATGAGTTTGTTGTTTCACTCCATGCATGCATCCCCTTTGTGTGCAAGAACCTCTCTTTACTACTACGAATTGTCAATTTGGTTATTACTATATTTGTAATGAGTTTGTAGGACCTGTTACTGGGTGAACTGCCTACTGACGGCAGTTACCACTAATCGTTGGAATTAATTTAGGAACAAGCTTTGCATGCCTTGTTTTACTGGAAGAATTCCTAGTTTTTAATACCCTTAATTTGTTCTATAATGGCTTCCAGTAGTATAATAAGTAAAGATGAACTACACATGCTTTTCCTATCTCTAACACTGTAATTTTCTTCTTAAATGAAGCATGTCATGGTGGTTTTGTAGGCACAATTCTTGACCATTCCACTGTTTTTGTTTACCTCTGATGGAGGATATATCGAATTGTGTGTTTGTCTACTAGTCACTCCTAAGTAGACAAGAAATCCTTTTCTTGTCATTATGGTTTTGCATAATCCTCTATCTGCCACTGTTAGTGGCACAGGTGGTCCCTGTATGCCATTGAGACATCAGTGGCACATAAAGAACTGCAAAATTTTACAATGGCAAATGATGAATTGCCCCTTTTTGTTACCATATATAGGTTGGTTGATTGACAGAATCAAATTGTTTTATGTAACGATTACTGGACTAGGAAAATAACCTTCACAGCAGCGATCATCCTTAATTATTGACAACTCAACTAGACAAATAAAAACTTGTAAAGTGTTACTTCCTATATGAATATAGTATAATATACGATCCACATGCCACCTTTATTACCTGC

CATTTGCAACTTGTCAAATAATCAAATCTTATACTTTCACTAGCAACTAAAAGTTCCATCATTTGCTTAGCGCTGTTGTTCTCGAACTCATTTTTCAACTATTACTAGTGTGAATGCCGACAGTCTCCGCTACCCTTTCTTTTAACTTTCGTGGAGAGTGATTGGAATGCCGATGTCTTGTTAGCCCACGGTGTCTTGTTAACCCGTTAAGAGCATGTTGACAGATCAGAACTTTTGTATATTTTCCTAAAAGGCGGTGGAGGTAGAAGATGTCTCAAAGGTGTTTATCAATTTTGTTCCACCTAATTTAGGTAGACTAAGTGTTAATAGATTGGGAAAGGACATGACTAATATCAGTAGTGGAAGACTTAGAGGTACTTATTTTATCACAAAACCAAGAAGTGAAAAGTGGGTATAGACTATTTTGGTTAGTAAGCTGTAAATTATCCTATTGTAAGCACTAAGCAGTCGGTAAAAGTATTTAGATTGCCCTCAGCACTTTTGGCCAAATAATTCACCTCTTTCCCTTCGATGTGAGATTTCAGTACATGACATCCTGTTTTCAGGTTCTATTACAGATGCACCTACAAGAACGACATGAAGTGCCCGGCTACAAAGCAAGTCCAACAGAAGGACACAAGCGACCCACCGTTGTTCTCTGTCACTTACTTCAACCATCACACCTGCAGCAGCATCTCAAATCCCATAGGAAGCACGAGAGACGTTGCCGCACAATCGGCCTCGAGCAAAGCGGTGTCAATCTGCTTCAGCCCGCATTATTCTTTCAGAGATGAGCCACAGTCACCGATTGCACATTCTTTCAGAGGCAACCAGCAGCCAGCTGAGAGGAGCGCCTATGCAACAAGCCAGTTTCAGTGGACCGCCGCATCGTCTCCGTCTCCTACCAGTAATGACAGTCCGGTTAAGATGGAGGTTGACACTTTTTCAGGAGCAAGCGCTTCTTCATCCAGCTCCAGCAGCATGGGTTCTCTCCCGAGGACGAGGACGTTGCTACCGATCGGTCAGTCCAGATGCATCGAGTACTTCCATTTCTTGTGATGAGAAGAAGGTGGCCGTTTCTTTTTTTTTCTTTTTTTTTTTTTGAGCGCCAAGTGAAGTCATATGTGGGCGTTGGAGGCAAGTAACATTGCTGTGCCGTCCCGGGCATGAACCACAGGGACCCAACCTACAGGTCCCCATAGAGATGTTCGTTCACTGTTGGCTGTTGCTTACTTGTTTACTGTCAGATTGAAGCCACATTTTTGTGCTTGAATATGATGTGAAGAATGGCACTGGCACTGGAGTTTCTTTTATCTGGTAGACTGAAGCCACATTCGTGATTGAATATGACGTGAGGAATGGCATTGGAGTTTCTTTTATAGTGATAGACTGGTTTTTGAGGTAGCACCATTTGTTTGTTTAGGGATGATTGTAGTTTGTATGGAATTCCTGACAAAAGGTATTGCCTGGTGATTCTGCTAGCTCTACTACTGAAATAGTAGCGAATTTGAAATATGTCCTGCGCTATTGTAGTTCAATGTTGAAGTGGAGCTTGCGAGTTGAGACTACAGTGGCTTCAGTGAACTTACTGCCTCGATGGCCACCTGGGTTATCCTTATATTGATACATGTGAAGAATGCAGATTGAAGTTTCTCTACCAAAGTACTAGTAGTACATTATTGTAGTAAACAGTTCATTGC

TTTAGCTCTCTGTCTCTAAATAAACAGTTTAAGTTTTGGAGACAAAAAATGACAATCTTAGCTCAATCGGATCTCTAGAGTTGGCACATGGACCAGAAGATTTCACTTTAATCATGCTTTTAATCATGTGGATTGGGACTGTTTCTCTACCAATATGTGAAAGTCTTTCGGTGTGTGGGGCCTGTTTGTGTGTTTGGGCTGTATTGTACCTCTGTGGGCTTGTAAAATGGAGAAACAAAGTCTA

>SbWRKY38

CGACATAGCTGGGTGTCTGCCCTCGCTCCCCTCCGCTTCGATTCCCACACACCTGCCGCGCAATACAGCAGTTGAGAGCGCAGGAGCTCCAAGGTAGCTAGCTAGGAGACGAGGAGGAAGAAGAAGAATAGGGTCGTCGCCGACTCCCCGCGCATGAAGAGGGAGCAGTCCTTTGAGTTTGGGGATCCCAGTGCCCAAGATGCTATGGGATCTGCTGCCTCGGAGTCGTCTTACAGCCCTCCCGGAGCTGTGTTTGGGCTCTCCCCACCGGAGTCGGCCTCGCCGCGCAGCGGCCGGCATAACAGAAGGTACACGCATAAACCCGATGAGCTGATAGATACTGTATCTTCTCAGCTCTTAATTTGCTAAACAAGTAGCATGCTTGTCTAGTGCAGTGTGGATTATCATGGCTATTAATCGGCTCCTTGCTTTCTTGCTTCTTTTGGGTCGTTTCCTTTTGTTAATTTCTCTACTTTTTGTTCTGACTGCCCTCTCCAGATCTTGATGAGCCTGCCTGCTAGTTGGTTTTAGTTCTGTGTATTGTTGCAGCGTTCTATCTTCATTTTTTTTTTTGCGAAAGAAAAGAGAGCTGCTCTATCTTCTTCATGTTCATATGTGCTACTAAATCTATCAGAATGTGTGTCTATAGATGGCCAGAGCTATATATTTCCGTCTTCAAGTTTGTGCCGATTCATAGACAAGATTTCAGGAAAGAAACAGTAACATACACTCAGAAGTCGATGCATGATCATAGGTCAATTCCCGTTTATATTAAATACAGATCGAGTACAGTTGGATAACCTCGTCGTACTATGTTTACAACTCAGACTTAATAGTTTATTTTGCTAAAAACAGTACAATACAGTGGCGTTGGAATTGGAAAGTAAGGGTCAACTACATGAATGCATATGCAACCCCATCTTCTTTCTCTCATACATCCTTCGATCCTAGCTACCAGACATGATGGCTAGCTAGTTCCATTCTTGAGTGTAACTATATTTCATGGACTGGGAAAATAACCTAATGCATGCATGATAAAGATATAAATTTGCGCATATGTGAGGATTCCATCCGCGCACTATGGTCAAAATACAGCATAATTTTTTTAAGAGATTAGAATCATCGTATAATCCTTTGTTGGGAATTCTTCATATTTCCCTTAGAAGAAGCATATTCTCCATCCCTCAGCCATGGCCATTTGTAGTGAGACTTCAAACCCCAAAGCAACCTTTTCCTATAACCTTTTCCCATAGTGGAGGGCTCCTTTGCTTAAGAGCCCTAGATAACCACTAGTACTACTAAACTTATGTGCTTTTGCACATTTTAATCATGCTTATATTGAAAAGGGCTGCACACATAGTGACCCCCTCACTATCCTCTATCATTCTTTTTTCTTTCCTTTGAACAGAAGTCTAGGATTGATTAAATTTTAGGACTTAAGCAAAAGCTACATTTCCCTTCCAAATGTGAAATAAGGAAATAGAAAGAGGTAGCACTGGATCATGTATGCAAAGTCGCCGGTTTTGATCATGCAACCGAAGGTCAGACATGAATTTATACTTCACGTGAATTTGGCATCATTGTAGGTTTCAAAATGGAAGGGTACTCCCTCCATTACAAATTATAAGTCGCTCCATCTTTTCTAGATATACAATAAATGTTATATTTTTAGACATAACATATATCTAGATGTATAGTAA

AAGTTGCGTATCTATAAAACCTAGAATAACATATAATTTAGAATAGACAGTAAGAAATATGATGACTTTGTATACATAGCCACAGTCGTGTGGAAAAATCATGGCAGCGTATGCCTGAATATGCTTGACAAGTTCATCGCATATTACGTTCATGCATTCTTCATGGATCTACCAAGGAGAACAAAAGAAAGGAAGGATACATAAATAATTAGGACCAAGAGGTGACATTAATTTTTTTCTCTTTATTATGGTCCTTGGAAGGTATTTAAATCACAATTAAACTAATGATGCCTTGGACGTAGGTCGTCATTGTACCTTACTTCCCTAATACTACTATATACAATAGTAATATCAGTCTGCTTTCTTGATACCTTATAAGGATATATATAGTAAGAAAAGCATAACTCGAACCTGTTTGCATTCGTGCCCCAATTGTTTTTTGTTTCTTCTCACTACACAATAATCTCTAGTAGGGTCATTCTTTTCAGCAACGCTGACCTCCAAATTGATCACCGTATTACTCAATTACACAGTATATTAGTTATGGCACCTACTTTATATGATCCACTTGTAGGACTTCTTTGTACAAATGAACACAAGACATAATAGAAATCTTTCACCAAGCTATAAACATTGCGATTAATTCTCTATGAGCAATCTTATCTACGGTTTCTATATATATATGGTCCATCTAGATAGAACCTTTAAAGTTTTGCTTCCTAGTGCCACATGCTATATATCTCCATTTTTGTATTGTTACTCATGTTCTTATATCTCCTATAAAACACATATGGAATTATTGATAAAGTATAATTTTTGTTATACTTATCTTATTTACCTATATAGTTTTCAAATAATGTTGCAGGAGGGATAGACCTTCATGGGTCAGACTCACGTACACACCTTATTTTGATGGTCACTTGTGGCGAAAATATGGGCAAAAGAAAATCAAGGATGCTGAGTACCCTAGGTAATTGACTTGTCAAGTCTTTTTAATTGTATTAGTTCATATGGAAAACTGCTCCATCACCACTTGTTTTGATATCATTTGATTATTATACTCATGATGGATTTAAGACACTCTAATAAGAGAACAAACATGAGCATGTCAGCTTATTTTCTTTTCAATTGATCAAACTAGTCGGTAATCTAGTTGGTCATTTATGATATTTAAGGAAAAAAACAGCTCAATTTGACGACATAAACCCATTAAATCTTCAAAGATAACTATAAAAATCTTAAACATAAGGGATAAATTCAGAAGTTACAAAAAGAGAAGTGTTTATAATCCTCTCAACTATATGGCTTATTTCTCCCAAAAGATTAATAGTTAAGCATCTAGCTCTTTATATTCACTTTAGAAAGCACATTTTGAGGTACATTATTCACTTTAGAAATGCTTTTATTGGACGTCTTGTTCACCACAATTTTATTTTTGGAACAGTGAGAATTAATCATTCAATCAAGGTTGATATCTTTGTCATGTTTCTTATTTGTTGGGCCTATCTTTCATGGTTGGAAAAAAAAATTTGGGGCAAAAAAGAGATTACATGACATTGTTTGAGCTCAAAAAGTTATACAAGATCCCAAATGTGCGGTCAATCGTACAACGAAATAATATGTCCTGGAAGTAGCCGCACAAAATCCTATCTAGCACGGTCAATGCTTAATTATTAGCATGCATGGCTCCTTACATAACC

CTTGAGTTATTAAATAAGTGTTACTCCATCTATTCTAAATTATAAGACATTTTATCTTTTCTAGATACGTAGCTTATAATACAATTAGATATGCGCTATGTCCAGTTATAGATATATATAGTAACCATAATTTATCTAGAAAAGTCAAAATAACATCTTATAATTTAGAATGAAGGGAGTAAGTTTCAGCATATCTTTCTAGTACCTCATAGTTCTTGATAAAGAAAAAGTGCATGACGCACTTCCTTGACCTCATGTCACACAACGATAGTTATATTCTTTGGTTGTACTTTAACCACCTCAATCTTCATGTACTGAGATGAAAAACTATTAATTTTTGGTCTAAATATATGTGAATTGAGGCGGATTAAAGTGCAGCCAAACATAAGGCCTAAGCTCAGCATGGATATTGTGTATCCTCATATATCGCCGCCTTTAATTTGCGCCACACAATGCTAGGTCGTCGATTAATTAGTCAACCATTGTCAACCCTATAGCAGGGGTTCAAGGATTAGGATGTTGTTGTTATCGACACTAAGCTCAATTCGAAGAGAAAGGGGAGCAGAAAGGAAGATGATGTTGCCACGGCAAGCAGGGCCATGTCCTCTCGTGATGAGGATGGCGCGGCAAGGTGGCCTTTAAGGATACAAGATGAAGCCTGATCTCATGGATAATGAATTCTTAGAGCAAAACATGAACGTGGGGGCATAGAATGGAGTAGCCATCTCGTGCTCAAATACTAGTAGATGGAGGCGGCGCGTAGGCATTGCACGGCTAGGACGAGGCTGCTAGCTGCTATATCTAGTTTATTGCTAATTTATTGCTTGATCCATATACTTCTTTAGTGGTTAACCCAAAGTTATATATTTTGATATCCCATCACGCAACCTAATCTAGCTCTATATGCAGTTACGCACCCTTTCTCCAAATAATTAGCTATAGATGGCCCTAGCTAGTTTGGATTCCTAGTAACATGCATCTTTTGTACTCAGCATCGTCATTTGGACATTAAAAAAGCACTTAATAAGCTGGCTTTGGATATGCTCGAGTAATGGCATTGAGAAGTGTACTTAGGCCAGTCTCAATGCATAGTTTCATGACACAGTTACCAAGACTATAAATTAGATAACCGAGCCACAAGAGTTTCATGGGGATGAAACTCCTCTCTCATCTTATGAAACTCCTTCATTTAATGATCTTGCCAAGTCAGCAATTTTGCTTATGTGGCACCCTATTTAATGTGCATGACACTCTTATGAAACATGCATTGAGACTAGCCTTATATACAAACTAAAGGTCAAAACGAACAAAATTAGTTGATTACATAACTCTTACATATAATGGATGGCTTGCTATAGTCTATAATAAGAAACAGTCAATATACATTGATCGATGCAGAAGATGAAAGATATTAATATGTTTCCTTTTCTAAAAAATAAAAATGCTGATGCCGCTGCAGGCTATACTTCAGATGTTCTTACCGTGAGGACAGGCAATGCCTGGCCTCTAAGCTGCTGCAGCAGAAGAACGGCGACGACCCACCACTGTACGAGGTGACCTACACGTACGAGCACACGTGCGGCGCACCGCCCGTCTCGTTCCCGGATATCGTGGCCGAGCCGCCGCCGGCCGCCAGGGAAGGTCTGGTGCTCAGGTTCGACTCCCCCGGCGGCCACGGCGGCCACGCACGGATGCAGCAG

AACGGACACTGCCAGCAGTCCACGTCTCGGAGTCCGTTCATGATGCTCAGCTTTGGTTCCAGGAGCCAGACGCACGATCAACATCCTGCCGTCTTCCGCTCTGACTTGGAGGCCGGATCATCGTCGTTTCCCACCGAGGCGCCGCCGGCGCCGCCACCACCGGCAAACGGCGACGGTGGGGACATGCTCTCGACATTGAACTCCTTCGCATATGATTTCGACAATCAAATGCACTTCGGCGATCACACGTATTTACCTCATAATAATAGTAATTACGATTATGATGATTACTGATCAGATGTGTGTAAGCGCTGCGAATCGCAAGCCTGCTCGTTACAACATGTGGCGTCCTTTGATCTTTATTATGTGAAAGTATATTTATGAGAAACTGGTATATATATATATATATATATATATAT

>SbWRKY40

ATGCAGACGCAGTCCCGCCTCGGCGGCAGCGGCAGCAGCAGCTCGGCGTCGGAGGACGAGCACGAGGCGGTGATCCGCGAGCTGACGCGGGGCCACGAGCTGACGGCGCAGCTGCGGGCGGAGGCCCTGCGCGCGCTGCGCGGGCAGGGCCAGGCCGAGGCCACCGCCGCATTCATCCTGCAGGAGGTGTCCCGCGCCTTCACCGTCTGCCTCTCCATCATGAGCTCGCCCGCCCGCGCCCCGCCGTCGACGTCCCAACCTCCGCCGCCGACGATGGAAATAATGGCGCCCGCCCTGCTGGCGCCGCCGCGCCGGAGCCGGGACGACAGCATGCCAAGAGAGTGAGTTGTCCGCTGCCCCGCCCCTGCCTGCGCAACAGACAAATGCACCAGACCAGCAGCTCCCCACCGGTGGGCGCGATGCTTAATTTGTTCTAACAAATTATTGCACGCTGATCTAATTTGTCGCTGTTGTTCGCGCGTGCATTTGGCTGGGCACAGGCAAAGGAGGACATCCTCGCCGCACTGCGATGGGTACCAGTGGAGGAAGTACGGCCAGAAGAGGATCACCAAGACGCAGTTCCCGAGGTCCATAATATCCTCTTCCTACTGTACTTGGCATAGTAGCTTAATACCAAAAATAAGAAATTAACTACTATATATATATATATATATATATATATATATATATATATATATATATATATATATAACTCCTGAAATATAATAGATGCATTTTAAGTTGCTTTAAGTCAAGCTATCTTAATTTTAACTAAATTTGTAGAGAAGAAAATTATCAATATCTACCATATCAAAGAAGTCAATACATTTCATAATGTATCTAGTGAAGCTTATTTGATGTAGTAAAATATATCCTTTTATAAATTAGAACAAAATTAGG

ATGATTTGACTTAGTATAACGGAAAATCTACTGTATTTCACAATGGAGGTAGTATATATCTGTGACCGTTCTACTCGTGGAGTGCATATATATATGCTAGTATACCAACCTAGTGTAACCCTGCATAAGCCTAAAAATAACCCTACTCCTCCAACAATTCAGCACGGCCGCTATACAAGAAAAGGGTAAGTTTGCTCTGTTTCTGCAATAAGGGTAAGATTAGCTAGTGAGCGTCACGCATCTAGAGGTAGGCAGCGTGCTAGCAACGGTGTTCCTCTCTGAATGGACGGGGGAATGGGATTGGATGTGGATGTGGTCGTGGAAAGCGACGCGTACGTGGCACCGGGGACGCAAGAGACAAGCGTAAAAAGCCGCTTGATCAATCGAGTTGACACGCACACCACCCCGGCCGTGTGTGTGTGTTCGCCTTTAAAGATTCTTGAGCGGCCAGCAGAACGAATCGCCCACACATTTGAAAACCATTCTAATAAAAAGTTTGATTAGGCCTCTAGCCCCTCTAACAAAAGCTGTTGATTAGAGATCATTCTGATGTTGACGCATGTTTTATCTTCCAAGTTTCTTTGTACATTTGTTTTTTCTTGACCAGCTTTTTTTTGTCCCCCGAAAGTAACATCTCCGCTTGGTGTACTTCTCGTGTGCGTAGACCGTGCTTTATTTACTACTATTACCACTGTGATTTACTAATTTTGCTATTACCACTGGGAGTAGTATACTCGAGGCATCTTCCTTACGTCACAACAAGATCCTCGAGTGACGTCGGATGCCGCGAAAGTCTCCTGTCTTATTAGCCGCCATGCATGTGCTGACTGAGATGGATAGGCTAGCTGATCCTGATTCCTACAAGTAATTAAGCGTGTTGTCTTATTACCTTGCTCACTTTTGTACGTACGTACGTTACGTGGTGCGCAGGTGCTACTTCAAGTGCAGCTTCCACCGCGAGCGCAACTGCCGGGCCACCAAGCAGGTGCAGCAGTGCAGCAACGACGACCCGCCCCAGTACGTCGTCATCTACTTCAACGAGCACACGTGCGACGACACGGCGGCCTGGGATCCTACTCCCACGGTGCCGCTGGACGACC

TGTCATCCGGGCTGCTGGTGGCACGCCAGGCCGGCTCGCTGCTGCTGGACGAACGCGGCGTCCAGGAGGAGCACGAGCGCCGGCTGCTCGTCTCGTCGCTCGCCTGCGTGCTGGGGGCCCAGCAGCAGCAGTCTCCTGCCGGCAGCGGAACCACCGCCGCCGTTAACGTGGGGCATGAGCAGGACCAGGAGCCGCCGCCGCCGCGGGCCCGCACGCGCGACGACGCGCCTGCGCCTGCGCCTGCGCCTGCGCCTGCGGGCGTTGACGACGACGCGCCCGGTGAGATGCCGCGCAGCATCATTGACGTGGACGTGGCGGGGCTGGATGTCATGGACTACTATGTGACGGACGCGCTGTGTTTCCGCGATTCCTACGACCTACCTAGCGACGGTTTTTCGTTTTGATGAGAGCGCAGCACATGCATCTTCTCGCGAAACCGGGTATTACGTGTCAGGCCCGAGATAGTTTAATCGGACGAGACGGCCGGGTCACTGAACCGTGATCTATAATGACTGCTACAGATATATACATAGAGCGTATGCGTACGTACCGCGTCGTCATAGAACACTACTGTAATAGTACTATATCTATCTATAACTATGTCTATACTTGCAGTAGCTTACAGCCGGGGTTTGCGTCTCTCCAGTTAAGTTTTGGTCAGGCTGGTGTGATTAATTTGCAAGTGCCTGATTTGTTAATCAGCACGTATACTTAGTATGTAGAACTATCAATTGATGCAGTTCCATTTTTTTTTGTTTCACTATTACTAGTGTGCAATGTTTTACATGTACCACTATATATGTATATTCTTAAATTTTTGTCAA

>SbWRKY39

ATGGCATTTGATCGAGACGCCAAGCTGTTCGACGTGCTCGCCAACGGTTACCATCTCAACACCCAGCTCCAGGCATTGCTCGTCGGCCGTCCCCTGAATAGCATCGGCCAGCAGGAGGCCATGGCGTTCAGCCAAGAGCTCTCGCGAGTGTTCAAGTTATCCATGTCCATGCTGAACTGTAACACAGTGACAAGATTGAGGACGGCGCCGGAGATAAGGGCCGGCGATAGCTCCGGCGTCATCATTCAGGCGGTGAAGGATAAGCGTGCAAGGTATTATGCGCTGATTACTGAAGGCGTGTGAACTGTTGTTTTTGTCATTTCAACTTTGTTTACCTTTTTTTTTCTTTTTTATGAGAAACTTAGCTTGGTTGGTTGTGGACTGTGTTTGGATTGCGTAATCACGAATTCACCATGCTGTTGCTAGGAGTGATAATGGGGAAGTGGTTACTCCCGTCAAGAAGAGTAGAGAAGATGGGGTTACTAGAAAGGAGATTACGGCCTCGCCATACAAGGATGGTTACGAGTGGCGAAAATATGGGCAAAAGAACATCCAGAACTGCAATTATGTGAGGTGAAAGTCCTATAATCCCTCTAATTCAAGAATACATGTTAAACTCTATTTTTAGAATTTCTACGAGTAAAACTTTCAAAACTTTGACCATCAATATATAATATACAATTAAATGAGATATGTTTACTTGGCGAAAAGATGTAAGCTAGGTTTACATTAGAAACTTCAAGAAATGCATTTTCATCATATTATTTATATTTATATGAAAAAAAAAATCATATTTTCAAAGAAATTGCTAGTATTATATTAATTGTGGTTTAATTCGTGGTACCTAGAAGCTAGGGCATGCATTGTCATATGCTGTAACAAGAAAATATATAAAAATATAGTCTCCATCTAGTAAACAATATTTCGCTACTCCATCTTTCTAAATTATAGTTCATTTGAACTTTAGAAAAATCCACCAACATCTATAACATTCAATTAGTTTAATTAAATTCTCAATGTAATATGGTTTGTCAATGTATTTATTTGAACATGTAGATGTGTTAGTATTTTTTAAGAAAGCTTTATTAGAATAAGAGAAGTTTGATTTGGGATAAAATTAAAACAAACTATAATTTGAAAAGGATCGAGTATGATTTAGTGGAATCTGTTATTCCAAAAATATTAGATATAATAAAAGGAACTTGTTCTCTGAAAGGTCGATCGTCATCCAAAGAGGTTGGTTCTATCCATGATCTATGAATATTTGTTCATAGATCTGGATAATGTACACTTAGGTCTTGTTTGGTTCCTCTTGCTAAATTATAGCTAGCTAAACTCTAGTCACTTTAGTAGCGAAAGTTTCAAACACATTGACTAAAAGGAGCTAAAATAATTTAGTTCTATTAGTCACCTAAGAGTAGCTAAAATAATTTTAGATAGCTAAAATTTAGTAAGGGGAACCAAACAGAGACCCGGAATTGCTGGTGTTTCTAATGAGCAACGCCCTTTGTCATGGTAAAGAGGGAATTTCATAAGAATTATGTTGGAATTATGGTGCATATATATCCTGCAAAAAGAAGTCTTTGGTAATCGTAAGCCATCTACTACTAGTACTACAAATCAAGGCACGCTTGATTACCTGCAACACATATAATGTCCAAGCCATTTCATAGTAATTAGTACACTAACTTAACCACGCG

ACTAAGTCCATTTCACTGCCGTCATCTGGAACACAATGAGATGCACGCTTGCACACAGCCGAAGACTTGACTTGCGTCGTCAACATGCATGGTCAAGCTTAACCACCATCCACCACCCCATCATCCATAGACCACAGTACAGTATGGTCCTTTGTCCTGTTTTGGAAACAATTAATGCGCGAGGTCCATGGTCATCAAGCATCAAACTTTCCTAATCGTAGCTGTGTGTAATGCGCAATCTCTGCAGGTACTACTTCAGGTGCAGCCGCGACCGGCGTTGCGAAGCGAAGAAGAAGGTGCAGCAGCAGGACGACGGCAGCGGCCGGGGCCAGCCGCTGTCGCCTCCCATGTTCGAGGTGACCTACGTGAACGAGCACACGTGCCATCTGCTCCGCGCCATTGCCAACGACGGCGACGCTGCTAGGATGGCGGCGTCGCCCCGGACCACGAACCGGTGGTCCCGCGTCCTCGGGGTCGTGGACACCGCGAGAGACGACGACCACGGCGGCGGCGTCCTGTTCAACGATCTGTCGTCGTCGTTTCCCCGCATCGGCGGCGGCGGCGGCGACGACGCCCAAGAGAACGAGACGATCGTCTCGTGCCTCGCGACTGTCATCAGCGGAGGAGCAGCGCCGTCGCCGCCCCCGTGGCCGCCAGCAGCGGCCGAAGCGGGCGCGAGCGATCATCCTGCTGCAGCGTCGTCGTACGGCGTGCCGCCGCCGATGCAGGCCTCCGGACACTCGGCGAGCGTGGCGGAGGATGGTGGTGGGACGACGACGACGACGACGACGATGATGATCGACGACATGGACACGGACTTTTGCTGGGATCCCTCGTCGTTTTGTGCGGTGGGGGAGGGAGATCAGCTGATGATGGACCACCGCGACATGCACGTGGATGTCGCCCGGCTCGCGGACACGGTGTGGCCGCGGCACACCTCCGCGGGTGCGTCTTGGCGTTGA

>SbWRKY81

AAGCTCATGTCCTGTCCAAACTTCTCAGTTCTCACCCCGCCGGCGCCGGCTCCCAGTCCCAGCCAGCTTCCACATTTTTCTTTCTTCACCTTTTGAAAAGGTGTTCCCCCCTTTGTCGACGTGCTCCAACTCCATGACGCTGTCTCCACCGCAGCCGCCGCCCTCCTCCGCCCAAGGCACGCAGCGATCCAGGAGCTCAGGAGAGGTACCCAGCTGGCGGAGCTGCTCAGGCAGCAGGTAGAGCTCATCCCGGAGCCTAACCGCCGGCAAGCTGCAGTGGTCAACGTGGGCGAAATATCCATGGCTATGGAGTCGTCGCTCTCCATTCTCCAGTCTGAGATGGAGCACCCCTTCGTCTCCGAGGTCATGGCTGCGCCCACTGCCTACTCTGACGGAGGCAGCACCAGCAGGGAAAGGAATGGTCCCGTGGCCCGTACAAGAAGGGTGAGGCACCGGCGAGGCAGAGATGGAGCTGAACTCCCGATGTGAGTAGTACAAACCTATGATTTTTACTGCGTCTTGCCCAGCGAGTATATAACTTGCATAGTTGCATATCTTTATGTGGATCTGCATATGGGAAAATGTGTTAATTTGCAGATCTGTCAAATTTATTATTTGGGCTTAAGGGAACTAATTACGTCACAGTTACTGCTGTTTACTTTCTTGCTCCATCGTCAGTTATAACTCGCTTTTCATAGAGCGTGGAGAAAAATGGGACGTACTTACTAGTTTAGGCCTAAGATTTCCAGATCTACATTACAATTTACAGAAGAAAGGGAAAAAGAGAACAATATTAATTATTAGGTGTCTCTGGATCTTATCCTATCCAAGTTTACATCTATACCATACATGTGCGCTTAATGCTCACAAAGGCGAGGATCTTTATTTCATGGTTATATAGGTAGGAAAGTTTGTAAAAGGTCATGCAGTAAAATTACATTCTGTTTCTCCTCCATCATGTTATTCAGCAAGGAGATACTGACTGAGGCACCAGAAAACGATCATTTCCACTGGAGGAAATATGGTGAAAAGAATATCCTCTATGCTGAATATCCAAGGTATATATGTATCTTTGATGGAATTTCATAATGCGCATCACCAACTCTATCTAAGTCGTCATTAGCAGATTAATTTGTTGTGAAAGATCATGATCATTCATCCCGTTTTCTACTCCATCATGACACCTTTTACAAAGTACAAATATTAATTCATATAAGTTGTCAGCTTGTCATGGATCATATTCAGTCATTTACTATGATGTTTATGCTTTGTAGTGCTAGACCGATCGATTTTTGGGATTTGCTCGGTTAGCTTCCTTCTGCTTACACGTTTTTTTTGGGGTCTTTTGCTTCTCTAAAAAAGCAACTTCTCAGCATGTCTTTGGTACATTGGCGTTTAGCTTCTATCCTTGTACGTTATGGATTTTTTTTTTAAGTCAGACCCAACAATACCTATTCCCTATTTGGCCGGGGAGTTATATATAGCTAGCTTCTGTTCCCTCAAGGATAACTCATTTTGTGTGGACTATGGAGTATAGTAGGAGCTTATGATTCCAATATTAGAGCTGGACTTGGGCAATAGCTAAACTAAGGCCATTAAATTTGTTTCAACTTGTAGATTGTAGATTGCAGCTAGCTTGTATATAGACTGCTGTAACAACATACAAGCTGTAACCCATGCACTATGTATAAGTTGAAAC

AATACAATTATTTAAGGCAGATTTATCCTTGAGAAAGCATATTTAAGGCAGAGCATTATTGAAGTTTATTTTAAACATGGTGCATGTATAGATGCATGCATTCACATACGTATGCATGCGCACAATGCACACACAAATAGTCCATGTCTGATGTCTATGAGCATTTCCAAAGAGACACAATGCTGGCTAGGGGTGGAATGTGGAAATGGCCAGACACGCATTGAATCGAATATAAATGTCACTATTTACTTTTTTTTTGTTGAGGATGAATAAGGAAATAAGGATCGAATACAACTGATTAGGTAGCTCATCTTTGTCACCATTCTTTTCAATTCGTATCTATAACTGCATGGAGAAACCCCTCAAAAAAATGAACTGCATGGATGTTCATATTATAAGTTTTAGCGAATATAGATTCAAATAATTAGGATGTCATTCATTTTTTCATCTGTCCGTAGATGTTGAGATTTAACAAACTCATGCCTATGACACCTCCGCTGAAAGAATAATAGTCGCAAATATACGTGCACCCGTTCAGATTCGGAGAGTTGACATGTACATAAAATGTCGTTAGCAGCCTGTCAACAATCTCTACTTCTAGCTTATTAATGACATACTTTAATTAATTTTCTCAATCGAAAAAAAAAGAAGTTTATTTCAAGATTTATATATGTTCATACTGTCGACCGGTTACTAATTTGCAGGTTATACTACAAGTGCGGTTACAGCGATGACCACAAGTGCCCGGCAAAGAAATACGTGCAGCAGCAAAGCAACACCTACCCTCCACTTTTCTTGGTCACCCTGATCAACGAGCATACCTGCGACACCTTGTTCCGGGATGAGCCCAGCTCAAGCAGCAGTGGTTCGCAGGTTCTCGACTTCACAAAGGCATCGCTTTCTCCTGAAGAAGACAGCAGCATGCCTGTGTCTATGCACAGATATTCGTTTTCGTATGATGGGTACTAGTCGTCTTCGTTGCCCAGGATCGATGTCGGCAGATGGATTCCAGATCATCAGGTAGATTTATCCTTGGGACCTGGGTGGTAGCGCACTCGCTCAGGCAAGCAGACTCACGCGGCAAGAGTTGTAGCTCGGCGTGCACTTGCCTTTTGATTAAGGATTTCAATGTACCTGCCAGATCCAAACCCACGCATTTAATTATTTTCTCATTTCACGGATCACACAAGCTGGTCTCGGTGGGTGTGGTTTGATGGAAGGGCAAGGTCTTTTTTCTCTTAAGAGCAAGGTCAATAATAGAGCCAAGTGCTTAGCTATAAGCCAAGTGATTAGAGCCA

>SbWRKY14

TTGCTTTGCTGTCTTGATTTAATCAGTGAGAGTGATATGTACAGTTGGTAGCGGGCATATGGTCATTATTATTAGGTGGACTGTAGGCCAAAGATCTGCTCGAAAAACTGAATTAAAATGGAACAATCTCTCGAAACTAGAGTCCTTGTTTGCGTCTACACGAGAGGTCGTCGCTCATGATCTAAGCTGATGCCCTTCACCTTCGTATTTGGTTAGTACGTACTACTTAATTTCTGTTTCAGCATATCTTTAATTTGTTTGTATAAATTTGAGCACCATGATTAATTGGTAAAGTAATCTGTTAGCATACATGATGCATGCTCGATCTTAGGCCTCATAGAGATCGATGTCAAGTATGGAGGCTCCTAGTCCCACCACCAGTCCTTTGGACGGCAGCATTTTGAAGCTTCCGGGGAAGCTGGACAGGCTACTGCATCAGCGTCATCGTTACGGATGCATCCTGCCCAAGGGCGTGGAGGATGAGATACCTCTCATCAAGGGTGATCTCGAAGAAATCATGGCCATGCTCTCAAACCTTGATGACTATGAGGCTATGATGGTTAGGTGCTGGAGGAAGGAGGTGCGCGAGCTGTCTTATGACATGGAGGACTACTTCATCGACCAGTACGAGCACACCTCAGCTGCTGAGTCGTCCTTGTCCTTGATGATGATGACTGGATCTGTTTCTCGGCGACAGCGTCGTAACAAGAGCAAGACCACTGTCTCTAGGCTTGGCGATAAGATCTTGATAGCCAATAACAAGATGAGAGAATCCAGAGTGCGAGCACAAGAGCTATTTCAACGGTACATGAGCATGTACAATCGCGACGCCGCCGCCGTTCCTGTCTCTGGTTCAAGTTCAACTAGCAGATGTCATCATTCTAATTCCACACCCCGTGGTGGAGAAGAGAAAAACCTGGATGACCACCATGCTATGAAGGAGGCTCTGGAGCTGTCGTACGACATGGAGAACTTCATCGAGCATTATGGGCTACTCACTCCCGAGTCCTTATTGGCTGCTGCTGGATCCATTTCTGTCCGTAGCCGCCGTCAGGTTACTCATCATCAGCGGCCTAGTCGTAAGAGCAAAACAATTATACTCTCTAGGCTTGGTGAAAAGCTGAGGCAGCGCCTCTGCATGGCCAACATGATCAGAGAATTCAGCTTGCGTGCACAAGAGGCACTTGGACGGTACAACACGTATAAACTTGATGCCATTTCTGCCGGCCCTGCAGCTTCCTCCATTAGATCATGTACTACTACTGATGATGGAGTTTATTCTGGCTCGTCCTGGAAGTGGAATTCCACGACGTCGACGTGCGAGGATGATGTTATTGTTGGTATCAGTGCTGCTATGGAAAATCTCCAAGAGTTGCTCCTGACGATGCATGATGAAGGGCACCAGCAGAATCTTAAGGTGGTATCCATAGTTGGGTCTGGAGGAATCGGCAAGACAACAGTTGCCATGGAACTCTACCGCAAGCTTGGACATCAGTTCGATTGTCGCGCATTTGTGCGGACTTCCCAAGAACCTGACATGAGGAGGATTTTCGTCAGCATGCTCTCTCAAATTGGCCCACACCAGCCACCTGATAATTGGACGATACACAGCTTAATTTCCACCATCAGAGCACATCTGCGAGATAAGAGGTATGGGCCGCCTGCTGCTACTGTTAATTAAATACATATACATATTTGGG

CCTTGTTTAGTTCACCCCAAAAACAAAAAAAAAAAATTCAAGTTTCCCCATCATATCAAATCTTGCGGTACATGCATGGAGCACTAAATATAAACGAAAATAAAAACTAATTGCACAGTTTACCTGTAAATCACAAGATGAATCTTTTAAGTCTACTTACTCTATAATTAAACAATGTTTGTCAAATAAAAACGAAAGTGCTACAGTTCCGAAAACTAAAAAGTTTTCGAAACTAAACAAGGCCTTGCATCATTATAAATTTTATTAGTTTGTTCGGGCCTTATTTAGTTCCAAAAAATTTAACAAAATAGATATTGTAGCATTTTCGTTTTTATTTGTCTGATCATGAACTAACTAGTCTCAAAAAATTCATCTCGTAAATTACAGATAAACTATATAATTAGTTATTTTTTTTATTTATATTTAATACTTAATATATGTACCGCAATGTTTAATGTGACGGAGAATCTAAAAATTTTTACATTTTTCTTAACTCAACATCATATCAGGAGAACATATATGATCTTGAACGGCGTGTCGCTTGGGCCCCATTTTTTCTAGGCACGGTGGTCTAAAGTTTTAGGTCCCATTTTACGGATGGGCCGGCAGAAAGCCCGCTAGCGCCGCAGCCGCCGCCTCTTCCTCCCCAATCCTGCTCCACCGCGCCCACGCAAGAGCTGCAGCCTGCAGGCGCCTCCTTGGTCATCTCTCCGTCCATGCTAGAGCCGCCATTCCTCAGTGGCTCGGCCGCGGCCCGGCCTCCTCTCGCATCGCGTCCTTTCAACGCCGTCCAGTTGCCCGATTGGATGGCGCGCGGCGCGACCTCCCCTCATCTTACCTTGCCGGCAGCGCCCAGCTTCTGCCCGCTGGTTATTCTCTCGATGGCTCGTCATTGCATTATAGTATTCTCTCGATTGTATTTACCACTTTTCTCTTGTTTTGTGATTTCCATGCTGGAAGCCTAAGCAAGTTTATTCCTGTTCTATTTTCTGCTTGAGCTGCTTGGGCTTTCGATTTAGGATTAAATCCATAATATATCGGCGTCTTCATTCTGGCCTCCTTTTTAAACCTGCTTGATAGGTTCATCATCCCGTGTAGGAGTAAGTTACAGCGCATTAGTTAGGTAAATAATGTATGCATCAAACCTGATCATTAGTCCTGGAACTGTAGTCCGAAAATTTTTCAGTAAATGTGTTTATCAGTGACCTCTGGTTTTTCTTTTCTCATATCGGTAAAGTAACCCTACTATGTATGGGTTGAATCAGAGAAATAAAATCTAGAGTCTGCTTGTGCAGTTCTTATTTCATGTTTGCCACTGATCTGAATCAAGGTTTTCACTGCACAAAAATATTTGCAGGTACTTGATTGTAGTGGAGGATTTATGTGCTACATCCACATGGGACATTGTTAAACGTGCTTTACCAGATACCAATTGTTGCAGCAGAATACTAACAACAACAGAAATTGAGGATCTAGCTCTTCAATCTTGTGACCATGACCCCAAGTATGTTTATAAGATGAAACCACTAGGTGAAGATGACTCAAGAACATTGTTTTTTAGTTCAATTTTTGGCCCTCAACATGAATGTCATTCAGAACTAAGGGAAATTTCTCATGCAATAATAAGTAAATGTGGTGGTTTGCCACTGGCTATTGTGACTGTCGCTGGTGTTTTATCAAGTAAGCCAGGCTTAGCAGATCAATGGGATTACGTCAATAAATCCATAGGGTACAGTTTATCGATAAATCCTACCAGTGAAGGGATGAAACAAGTCCTTGACCTTAGTTTCAACATTCTTCCCCAGCATTTGAAAGCATGTATATTATACACTGGTCTGTATGAAGAGGACATCATAATTTGGAAGGATGATTTAGTCAACCAGTGGATAGCTGAAGGTTTTATCGAGGCAACAGAAGGGCAAGATAAGAAAGAAATTGCCAGATCTTTTTTTGATAGGCTTATCAGTAGAAAACTGATCCTCCCTGTATGTATAAATAAAAATGGTGAGGTTTTGTCCTGCGTGGTTCACCGTATGGTGCTAAATCTTGTTATCAGATACAAGTCAGTAGAAGAGAATTTTGTCACTGCAATACATCATTCTCAAACAATCACCACACTTTCTGACAAGGTGCGTCGACTGTCTCTTCAATTTGGTAATGCAGAAGATGTGATACTCCCAATAAATATGAGACTGTCACAAGTCCGAACACTTGTCTTTTGGGGGGTCTTCAAGTGTTCGCCTACCATTGTGCTGTTTCATCTCCTTCAAGTTCTGATTCTCCATTTTTGGGGCGATAAGGATAACATCAATTTTGACCTCACTAGAATTTCAGAACTTTTCCGGCTGAGATATTTGAAGGTCACATCTAATGTTACCTTAGAACTAGGCAACAAGATTCGAGGCCTGCAATCTTTGGAAACACTTACAATAGACGCAAGAGTTAATACAGTTCCATCAGAAATTGTTTACTTGCCAAGCTTGCTGCATTTTAGTGTTCTCCCAGAGACAGACCTGCCTAATGGGATTGGCCACATGACATCGCTTCACACACTTGGATATTTTGATCTCAGTAGTAATTCAATAGAGAATGTACAGAGCCTTAGCATGCTGACCAATCTCGTAGATCTTAAGCTAACCTGTTCTACAGGTCAGCCAGAAAATATGTATAACAAAATGCAGTTCTTGCTGACCTCAATTCTTGGCAGACTCAGCAACCTCAAGTCTCTTACTCTTGTCCCAAGAGCTTCCACTAATAATGCAAAATCTACAGATGAGGCTGGTGCTACAGGCATGGCCATTTCTGGTGGCTTCAGCAGTTTGTCCTCTGCCCCAGGCCTTCTTCAGAGTCTTGAGGTTTCGCCACAGATTTGCATATTCTACTGGATCCCCAAGTGGATCGGGCAACTCCACAAGCTCCGAATCTTAAAGATTGGGCTTACAAAAATAGACAGGGATGATGTTGATGTTATCAGAGGATTGACTGCTCTTGCTGTTCTCTCCCTGTATAGCCAGACCAAGCCTGCAGCAAGGATAGTCGTTGGGAAGACTGGATTTCCAGTAATAAAATACTTCAAGTTCAAGTGCTGTGACCCTTTGCTGAAATTCGAGGAGGGTTCTATGCCTAATCTCTGTAAGCTGAAGCTTGTTTTCAATGCCAATCATGCCCATCAACATATTACAATACCTGTTGGCATCAGGTACTTGTCAAACTTGAAAGAGTTATCTGCAAAAATTGGTGGTGCTGGTTCTGATGAGTCCCACAGAAGGGCTATAGAATTGGCTTTCAGAGATGCAGTCAGGGTGCATGCTAGATGTGAGAGAGTCAGCATACAATGTGTACAGCAGATCATTGGTGGCAAGGATGATCAGTATAGCCTAGGGCGAGTGGAAGATTATGGGGATGAGGAAGACTCAGATGAACTTGTTGAAATGATGCCGGAGCATTATGGAGAAGCAGTGGATACAGATGCTGACAACAGGTCTGGATTTATCCTTGTAGCCATATAATTTGGTGTTCTTCTTCTCCTTTGTGTTCTTGCTTATTTGATTTGCATGGAAATTAAACTTCTACATTGCTGTCATCAGGTCATTGTTATCCGAATTGCATATTTTACTCCTCTTGAGGAACAAGCTCGACAACCAATGTGATGATATCACATACAGTATCCCCATCGTTGATGTCTACTCATACGACCCATGGAATCTTGAAAGTATGTTGGCACCAGAGAAAATAAGATTGGAGGATCTCCGTGCATGCCATATTTTTTTTCTTGAACACGCAAAAGAGCTGCATATCATTATATAAGAAGAAAATTGTGCATACCATATTACTAGTGTTATTATCTCTATCATTAGTATCACATCCATAGAAAGCATAAAAGTAAGTTTTAAACATTTG

TTATTCAAAACAACTTCAAATTTTGCAATAGCATCCCATCCCACAAGTTACTCCAAGAGTTAAAGTTGTCAATACTGCCTACAAGATAGTTGCATTTATCAACCTGCGAGTCCAATGCTGCTGCAAAAGTCATTTGGCACATTTCCCTTGATACTCTTTTGACATTTGGCACATTGGACGTGATACGGTGAGACTGATCCACAGGTACGGAGGTACCTACTACATGGTTATGGAATGTAAGACGGTTTAGCAACGTATGCATAAGATATCATATAATCATGTTTGAGCACATATGCAAAGTAGAATTTATTTATTTTTTCTATAGTTATTAGAAGTCTATTGTCAAGGGCCTGTTTGGATGCTAAGGACTAATTGTTAGAGAGCTAAAAGTTAGTCCTAGCACACCAAATAGGAGGGTAAATAGATATACTAATTATTAGTCTTAGTCCAATTGCTATCAAAACCATGCACCACCATCTTGCTTAGTTGCTTGCAGGTAGTGGCAGCACGCATAGGGGACTGCTGTTATTTTTCCGCCCACTACCGGAGGCAAGGGGCGTCGTTCCGGTCAAAACACCCTCAGGTTACTGGAAAGTCACAGGTCTGCCTGGGTACATCTACTCGGATGAAAGACTTGCAGTTGGGATGAAAAGAACGATGGAGTTCTACCATGACCACTTGACATCAGGTACCAAAACCAAGTGGAAGATAAAAGAGTTTACAGCATTTCAGCATGCCACAGCTGGTGAAATCTGCACGCCCATGATGGTAATTTGATTCGCGCACTCTTGATTCCATTCATTCATGAAGAAACTGCCATGCTTGTACGCCTGAACTTTTCATATATAATGATGTTATGGGTGTCCGATTCATTGTGATTTTCTAAATGATACACATCAAGCATATGTTGCCTTGCTATAGCCAAGAAGCGAGATGAGCCTGTGTCAACTGTACACGGAATTGGCAGCGAATCCCGATTGCAGTCCAGCAGGTGTCCAGTATGATGAAAAATTGTCCGTCAAACAAAATAAAATTCCAGCAGAGGAACCATCAGTAGAAAGTGACATTGTTCAAACCCGCAAACATGCTGATAAGAGGTATGCATCTTCTTACATAGCTATTGTATTTGTAATAACAACAAGTCACAAATGCTAAAGTGAGAGAGAAGATTTCAGTTGAACTTTAGGATCATAGTTATCAATACTGTTGAAATTATGTATGTACATAGAAAAAACACGTTTATATGTTGACAGAGTTTTATTGAAACAACAGTTCTGTTATCATTTTTTGAATGGTTAAATTGTATATTCTAAGACAAATATAGTTAGATTTTGATTGATATTTCTTTTCTGCGATCATATATTTTGTTCAATTTTCAATTGTACATTCTGGGGTCAATTATTGTTAAACTTACCCGTTGGAGACTGTCGATTGACCAGAATGTCTATACTATTTGTGACTTGATGCAAGTATATGGCAAGTTTCCTTGGTTGCGTTTCTATACGTGGTCACCTCTTACTGACTTTCTCCTGATCGACCTTTAACATATGCATATTTATCTTATGTTCAAAAAAAAACATATGCATATTGATCTACACTAAATTCTATGGCAAATGATATATGAGGAATTCTGACAACATGCCGTTTGAGATTAATTGCAGGAAACGTAGGGTGGACGAGACCACAAAAACTGTGGTAAC

TATTGCCAGCCCTGATGTGAACGACGGCTACACCTGGAGGAAGTACGGTTCGAAACAAATCCTGGGCTCAAACTACCCAAGGTCAGACTTATGTGTAAAAAAGCAGTTATGACTTTATTATTATAAAAAATAAAAGAAGAATAGTTATTACTCCATCAGCGGCAACACCCATGAGGAGATGGAGTGCTTGCTGATTCAATGGATCGAGCCTGATAAACTGAAATTCCCACAGAGACTACTACAAGTGCACGCAACGAAGAGGCTGCCCGGCGAGGAAGCACATGCAGCGCCGTGACGGCGAGCCAATACTCTACGACGTGTGTTACTTTGGAGAGCACAGCTGCGATCTTCAGCAGGGGCACTCCAGTGAGCAGGGATCAGAAACAAATACAATATCAGGAGGATGGGAAGCAGCAGGGCTACCATTATCGGTACCACACGCACATGGAATAAGTTCTGTGGAGGCCATGATAAGCAGCGGCAGCCTCACCGCGCGCCCCAATCCAGGTGTCTCCTCCTTGCGGCCGTCGTCCATGGTCACGAGCCAAGTAGTGATGAGCAACCCTGACGACGATGGCTACTCCTGGACAAAGTATGGCCAGAAGAACATCCTCGGAGCGAAACACCCAATGTAAGTGACGATCGACCGTGCAACTAATGCACACTCTTCCAATCCATTTCAAGTTTCCAACACATTATTCAAGTGCTGCCTCCGATCCTGCAGAAGTTACTACCGTTGTGCGCATTGGATCGCGCAGGGCTGCACCGCAACTAAACGATTGCATCGCAAGGAGGATGCCGACACGTTGGGCTTCGATGCCATCTACTACGGGCAACACACTTGTGATCAGATCGCACACTCCACTGACAATATCAGTAGCCCCTTGGGCTGTACCACCACTGGATCTACTTCCAAGCTGGGCACCGATAAAATTCATCCTGAACAAGGTGGACTTTGACTTTGGTCAACAATTAAGAGTGAAATGCACCATTAGTCTATCAACTTGTCCATCTGTGTCACTTCAGTCTATCAACTTTGAAAGTGCATTTTTTTGGTTTAAAACCTTGTTAAGTGGTACATAAATAGTTCATACCTCTTTGTTTCGTGAATAGATATGACATGATTTCGTTCGCTTGTCTTATAAG

>SbWRKY75

GAAATCTTGAAAACTTTTTACTTTTTGGGGTGAACTAAACAAGGCCTAAGTGAATGAATGAAAGAGGAACAAGTCGTCTCCCCTTCCTTCCGCTTCTGTTGCTCACAGAGGCCGGCAGGCCGCTGATGCTACCTTCCTTCTTGATCTTCTAGGTTCGTAATTCGGTATCGGTACTACTTGTTGTTTTCTGTTTGAGCATCTTTGTCTGTAAATTTAGGCACCACAGATGAGATGGGTAAGTAATGTGTAGCTGCATGCTCTTATGCCTCTGGCATGGGGATGGAGGATCCGACGGCGACCGCTTCTTTGGATGGCACCATTTCGAAGCTTCCAGGGAAGCTCGACAGGCTACTTCGTCATGGTACTCGCAGAAAAAAAAGGCTAATGCACCATGTCTGCACCCTGCCCAAGGGCGTGGTGGATGAGGTACCCCTCATCAAGGCCGATCTTGAGAAAATCTTAGCAATTTCCTCAGATCTGGAGGACGACCAGGCTATGACGGCCAGGTGCTGGAGGAAGGAGGTACGCGAGCTGTCCTACGATATGGAGGACTTCGTCGACCAATATGAGCACGCCCATGCCGTGTCCTGTTCCAGATCCATGATTCGTGGCCGCAAGATTGCTACTCAGCAGCGGCGTAAGAGCAAGATTAGTCTCCCTTGGCTCCGGGAAAAGCTGAGGCGGCGGCTGTGGATGGCCAACAAGACCAGAGAATTCAGCGCGCGCACGCAAGAGGCGCTCCAACGGCACAGCTTGTATAACCTCGACGCTGTTGCTGGCGCCTCTGCTTCTAGTAGGCGCACTTGTGCATATTCTGACCCTGCTTGGAATTCCACACCTTGTGGGGAAGAGGACGCCTATGTTGGTATCAATGATGCTATGGAAGAGTTGCTGATGATGATGCATGATGATCACGGGCACCAGAAGCTTAAGGTTGTGTCCATTGTTGGGTTCGGAGGGATTGGCAAGACAACACTTGCCACCGAGCTTTACCACAAGCTTGGGCATCAGTTCGAATGCCGGGCATTTGTTCGGACTTCCCACAAGCCTGATATGAGGAGGATTTTCATCAGCATGCTCTCCCAAGTTCGCCCACACCAGCCACCTGATAATTGGACGGTCCATAGCTTAATTTCCACCATCAGGACACATCTGCAGGATAAGAGGTGTGACTATATGTATCTTCAGTCTTCACTGCTGCACTGTTGCTAACATTAACTATATGCCTCAGTAATACTTGGGGTAGACAAAATATGTACAAGTATGAATCAGATAAAGAAAACAATACAAAGTCTGCTTAAAGGGAGAAATTCAGATTGCTCATTGTTCTTGTTTGATATTTTCCACTGATCTGACACAATGTTTTCACTGTAACAACAATATTTGCAGGTACTTGATTATAGTTGAGGATGTATGCACTGCGTCAACATGGGACATAGTTAAATACGCTTTACCAGATAGCAATTGCCGCAGCACAATATTAATAACAACAGAAATTGAGGATCTAGCTCTGCAATCTTGTGACCATGACCCCAAGTATGTTTATAAGATGAAACCTCTTGGTGAAGATAACTCGAGAAAATTATTTTTTGGCTTGGTTTTCGGCCAGCATGAATGTCCTTTGGAACTCAGGGAAATTTCTTGCAATATTATAAGTAAATGTGGTGGCTTGCCATTGGCCATTGTGACTGTTGCCAGCATTTTATCAAGTCAGCCAGGCGTACAAGGTCAATGGGATTTTGTCAATAAATCCATAGGTGAGAGTTTGTTGACAAATCCTACCTGGGAAGGGATGAAACAAGTCCTCGACCTTAGCTACAACAATCTTCCCCAGCAATTGAAAGCGTGCATCTTATACACTAGTTTGTATGAAGAGGATATCATAATTTGGAAGGATGATTTAGTTAACCAGTGGATAGCTGAAGGTTTTATCCAGACAACTGGAGGGCAAGACAAGAAACAAATTGGAAGGTCCTTTTTTGATAGGCTTATCAGTGGAAAACTGATACTCCCTGTAGATATAAATAGAAATGGTGAGGTTTTGTCCTGCGTGGTTCACCGCATGGTACTAAATCTTGTTATTAGAGACAAGTCAATGGAGGAGAACTTCGTCACTGCAATACATCACTCTCAAGCAGACACCATGCTTGCTGACAAGGTTCGTCGGCTGTCTCTTCAGTTTGGTAATGCAGAAGATGCAATACCCCCATCAAATATGAGACTGTCACACGTTCGAACACTAGCATTTTCAGGGGTCTTCAAGTGTTTGCCTTCCATTGAGCAATTTCGTCTTCTTCAAGTTCTAATTCTTCATTTCTGGGGCGATAAGGATATCATCAGTTTTGACATCACTAGAATTTCAGAACTTTTCCGGCTGAGATATTTGAAGGTCACATCCAATGTCACCTTAGAACTAGGTACCCAGATGCGATGCCTGCAATCTTTGGAAACACTTGCAATAGATGCAAGAGTAAGTGCAGTTCCATCAGATATTGTTCAGTTGCCAGGCTTACTGCATCTCCGTCTTCCTGCTGAGACAAACCTGCCAAATGGGATTGGGCACATGACATCACTTCGCACACTTGGATATTTTGATCTGAGTAGTAATTCGTTGGAGAATGTACAGAGCCTTAGCATGCTGACCAATCTCGGAGATCTCCAGCTCACCTGTTCTACAAAAGAGCCAGAAAGTCTGAATATGAAAACGCCATTCCTGCTGATGAACATGATTCTTGAGAAACTCAGCAACCTCAAGTCTCTAGCTCTGGTACCAACAAGAACTTTCAGTTATTATACAAAATCTATCGATGATGACGCTGGTGCTACCTGCATCACCATTTCTGATGGCTTCAGCACTTTGTCCTCTGCCCCAGCCCTTCTTCAGGGTTTTGAGGTTTCACCACGAATTTGCATATTCTTCTGTACGCCCAAGTGGATTGGACAACTCCACAAGCTCAGCATCTTAAAGTTTGGAGTTAGGAAAATAGACAGAGATGGTGTCGATGTCCTCAGAGGATTGCCTGCTCTTGCTGTACTCTCCCTGTATGTCCACACCAAACCTGCAGCAAGAATAGTCATTGGGAAGATCGGATTTTCAGTTATCAAGTACTTTAAGCTCAAATGCTGTGATCCTTGCTTGGAATTCGAGGAGGGTGCTATGCCTAATCTCCGTAGGCTGAAGCTTGCGTTCAATGCCTGCAATGCTGATCGACCGAGTACAATGCCTGTCGGAATCAAGTACTTGTCAGAGCTGAAAGAGGTATCTGCAAAAATCTGCGGTGCTGAGGAATCACACACAAGGACTGCCCAATTGGCATTTAGAGATGTCATCAGGGTGCATGCTGGGTGTCAGAGCGTCAATGTACAATGTGTAAAGCAGATCATTAGCAGTGAGGATGATCAGAGTAGTCAGAGTAGCATGAAAACAGCGAAATATCCACGAATTCAATATTTACCTTCCCCTGCCCAGGCCCCAGGCCGGAATCAGAACCGGCACCGGCGCTGGCCCGACAGCATCCCAAGAGAGTAAGTTTTTGGGCAGGAAATATGTGTGCAAACCACATGTGTGGTGAAAAATGAAAACTAACATAAAACACACTTCTGTAATTTGAAAATATTTGAAACTTGGCACATATATAATGCATCCATATATGAACTCATGTGCAAACGTTTGGAAGCAAATTCACTTTTGAAAATCATACGAAAATGATAAATTTCACTTGCATACGCACTACAAGACAAAATTTATGTTCTTTTTTTTTTGCCTTCTAATACACATGTTGATAAATTTTGTTTTTCGTATGGTATTTAAAAAATGATCTGTATCACAATTTTCATGTGACTATACATATATGAATAACTAACTATAGATGTGTGCGGTTTTTCAAAAAATTGAGTTAGGTATGTGTATTTTTAAATTAGTTTCTATGTTTTCATAGAAAACATGTTTTCCCATATATCCCCATTTTCCACTGTCTACCTGCCTACGCAACTCATAAATTTATTGCGGGCTCATCTAATTTGTCCCGTGCATTGCTCTCCAACAGGCAAACTACTGTGCGGCAAATGTTGACACCGACCCCATACGAGGACGGTTACCAGTGGAGGAAGTCCGGGCAGAGGATGATCAACAATGAACGTTTCCCGAGGTCAATAATCTCTCTCATCCTATCGGTACTCGTAATATGGATCGGTACCTTATGATGCATTTGGTAACGAGAAACGCTACAAAGAAAGTTTCAAGGAGAATCACTCGACCTGGTTTCTTCCGATTCTAGTTCTAATTTTAAGAATATAGCGA

GGTAGCCAAACGGTTCCACAAAGTAATCTTGAATGTGCATAGAAAAGTTCCAAACGGTCCAAGGAGGAGCACACCAGGAAAATAGATTGAATTAACAGTTCCATCGGAAAATCTGACGTGTTGTAGCCATGTATTGTAATGTCATGCATTTAGATTATGATCTTAGTAAGTTGTTAAAGTTTCATTATCTACATTTTAAGTTAAATTTTCTAGTGTTATTTTACATTTAATCAAAACTACAAAAAAAATATTCAAGATCTTCGACTACGCTCTTAGTAACTACCTACTATTATTTGGTGCTCTAGACGTTGAATTTGCTTTATCTAAGAATTACTTTTTGTATTAACATTCTCTTTATTATAAAAATAGAATGCTTGCATTTAAACAATCAATATAGTAAAATGTTCTCTTTTTTTTTCTTGTCGAAATGGATATAAGCTGAATTGGATAAGCTAGAGCTTTTCTAATAGATTATTTCTATAAGTTTTAGCTACTAATTTATTAGACATGACACAAATAATTAAGCTGCTCAATCTGTACGCACGTGCGCGCAGGTGCTACTACAGCTGCGCCTACCGCCGCGACCGCAAATGCCGGGCCATCAAGCTGGTGCAGCAGTACAACGACGGCGCGCCGCCTCTGTTCGCCGTCAAGTACTGCAACCACCACACATGCGTCCCGGAGGCAAACCCGGCACGCCGGAGCCGGGGCCGGAACAAGGAGCTGCCGCTTGGCTTCGGAACCTCTGTGGCTGGAGCTGAGCAAGGAACTCGACTGCAAATTAGAAACAGCTACAGAGGAGACTCGTCGTCGTCCGAGTCCAGCCGCGCTCCTCAATCGTCAGATATGATAGTGTTCTATGACTCGACGTCAGAGGAGACTATGTACGTATTAGGGCCGGGGTCATGGTACTAATTAAATAGCGGTTGTATAAATAGATAGCAGTGGTCTACCACTGTAACGTACTACTAGTACTGGATCAGATTTAGCAGGTTGTAGAACGGCTCTTATAATCCGTATGATGACCTACACGTAATGTGTGGACACCATGGGTTTTCTTCTTATGTTAGACATATCAGTTATTTTAGTACAGTTAAATACTCACTATTCATAGTATACATGCTTAAATTCATGTGATTATCATATGTCG

>SbWRKY37

GAAACGCGGGAGGGCATCGACGGCGGCGGCACCGACGGGAGGGGCCTCCGCACGGCGACGGCGACGCGGGAGGGTCGGAGGAGGCGGACTGGTGGTGGCCTCTCGGACACTTCCTGCGACGGAGCGGATCGTGCAAGGAGATGCAGGTTTCAGGTTTGCTCCTTGGCTTCAACCGTTTCTGCGAGCCCCAACCGTTCCATACTTTCCGACCCAAACTGTTTACTTAACAGCTTTAGGATCCCAGAATTTCAGCCCAGCTCGGTCAGCTACATCCTTGCTGAGTGGACGAGTGGTGACTGGTGAGTCCGGCTTCATGATGAATCCTGGATGCCTCATCTCCTGGTTTTGGGCCCAATCCCGCAGGCGAAAATTAGGGTGATTGAGCAATCGGTTTTTAAGGAGCTAGGGTGGTCGCGAACTCGCGATCTGAAACCAACATAGTTTTCATCGCGTATCAGGTAAGCCAAGCACATCTATAATTAATTTCATACATACATAGATATGGCGTGAAATGAGTGTCAGGCAACCATTCGTACGACTCTGACATGATTGGCAACAGATGCAAGATGTTTAGTAATAACGACAGAGAAGTGACTAAGGGTTTGTGCTTATAAACTATTCGGCAACATTTGAGTGCACTGGGGGTTACTTGTGGGCCATTGTCCAAATCAGCTTACTTCCAGAAGATTTCTTTGTCTTCCTTTTTCAAAGGGATAGAATCACTTTTCGTATCATGGCCTGTGCCATGACTCAATGACCTCATATGAACACACATGTGAATTGTGCTGGTTTTCTAAAGTGGTCTTCATTACTGGGACACGGAGTTTTATTCCAGAACTATTCCAACTGTCGGTTTGCCACTTGTTTTAGATAATTAACCGGGCAGGGCACAATGAAGTCTATGGTGCTGTGGTTGAGTAGTTGCAGTCTGAAGTACAGTGATGTACATCAACAAAAGTGGTATTGCAACGAATCCAGATCCTACTTCTGCAGACCAATTTTCAGAAAAGCTATCTGAAGGCATGATCAGGGGAGGGAACCTTTTGGACTATGAAGCTAGTGTCTGTCCCATTACTAGTTTATCACGGTGCTCACATCATCTGAAACATGTAAGAATCCCGTCACCCTCTTCCCTCTTTTACACTGCGAATATTTTAGCTGGGAAAGTATATTGTATCATTCAGATTGATGATGCTCACACTGCGTTTACTTCATTGTATTTGTTGATCAGATTTGGAAAGATTTGTGGGGAGAGTTCTGCACTACTTTTGACCTAGAATTCAATTCAAAGATGGTTAAAACAGTCACAATTGTGTATACTCATGTAAGTTGAGAAAAAGGCTTGACTGGTGCCTTGCTGTTACTTTAGTCATAAAAAAAATTGATTAATGAACATGGAATTAACGTCAATACAAACGCAATCAGCTATTTACACAAAGGCTTCAGACTAACCGTAGCTTTGTACAATAAAAAAGCGGTCAAAATGAGAATAGATAACCACTACAGGTGAACTTATGACAGCAACTTTGGATTGTTCATGAGTGGTACCTTGCGTACTGTTTTCAAAAGGTCATAGATTTTCTATCAGGTCTCACACCAACCACACACAGCATGTATCACATTTCACTTGATATATATTAATTTCAGATAGTGATCACTAGTGTTTTTTATGAGAATAGTGATCAGTAGGATTTGTTCTT

TCTTGTTAGACCATCCAAGCGTATATCTTGGTGTACTCAAGGGCTTAAAAATGCATAATGCTATCTGTCTTGTACTCTTTGATTGAGCTTTGGAAACTTCTAATGCAAGCATATGAGAATGGTACAAGATTACTAAGGTAAGATAGTGCCTTGCAAATATCCATGTAGAAGTTCAGTTCGGCAGTCTCTTTGGCTCAAAATCAAGAACTCAAAGGAACTATATTAGCTACAATACCAATGGAAGTATTGTGTGCTTCTACTCGTGTTTATAGAATGGCATTGTTTACTTATAGGTGTATATATAGCGCCTTGCACATTACTAGTAAATCTCATTACTATCTCTATGTTTTTTCTAATGTGCTGCAATCTAAAATACATAAATCTGGAGTAGACAAATCTATATGATTTTCTATAATTTACTTATACTAATTCCACCAATCTTAGGCCTCAAAAAGTATATGTGTCCTATGCAAATTTGCAATTTTTCATGCAACTACAATGAAAGTGAGCACCAAACTATATTTAAATTACAAACAAGGAAACTGATTTAACCAAAATTATGAGATTTGGGAATCCCAGAAGCTAAGCTTGGATCCTGTACATTTCAGCATGTACATATCTAGAAGTTTTAACCACTGACGAGACCTGAGAATCTAAGAGAGGAGCCTTTTCATGCTGGACTAAGAAGATGTCCATTCATCTACCCATGTGCATCCTATCAAAAGGCTACCTTACAGTTCATCACATAAGTAAGCCTCAGGTGCTGATATGTCAATCTTGTTGAGATTCAAATATGGCATAAAAATGTTATCTATTGCACCCTAAAATATGGATATATATGTGTTATTTCTGATGAATATCTGACTCACTGCAGAGGTTGTTTTTGGCAACTGGTCACAGCAGAGGCAGCAAACAGCAGTACATTAGCACCAGTCATCGTCAAACCATACCTGTGAAGTTCTCTTTATATTATGGAGCCTAAACATTGAGGACAATCTATTTCTCCAGAAACATGTAGATGTGCAAAGTGAAATGCGATAATCAAACCTGTCTTGAAGGTCTTCACTGCCGCAAGAATAAAAGACAATAATATGCTACTTGTGTCTTTCTTTTACCCTAAGAGACCTGAACAGGTGCAACCATGCATGTCACGTTGCATACATGTTGCCATGTTGGTTGAGTTCAAAAGTCCAAAACACGGTTATTAGAGTCAAGCGTAGATTTAAAATTCTGGAAGCAAGCATGCACCACCAACTCCAGGTCAAGGTTATCTGTGCATATATGAGCTACAGGCTGTGCTTGTCTGCAGCACTAGACAAGGAGAATAAAACTCATCATCTCCACTTCGCACCAGCCAAGGGCTCAGCGTTAATTGCTGAGAATCCATTGGGAGCTCTGCAATCGCAAGGTAATTTGCTGTCACCAACGCGCATCCTTGTTGAGAGGCCATATCGATCAGTTTGAAGACGTTGCAAGAGGCCAGCCATCATGGCTAAGAGGGATGACTACATGGACAGCTCTTGCGGCTGCTCTAATGGAACCCCTAAAAGGCTGGTGCAGGATTGTAGCAGTTATGCACAGGCACATGCTAAGAAGTAAGTCTAAGTCATGTTATATATACTAAACAGAGTTAAGTATGTGCACACTATATATACTAAACAGAGTTAAGTATGTGCTGCAAATGAAATTATGATAAATAT

GGAAGGAAAAATAGTTAGCTCTGTGCACTTTCAGTTATTTCTTTTGCTCCACATGATGTTCCTAGTTCTTATTACTTGTGCTATTCATTTTGTTTCTTGTTATGTTTTCAAGTTATTAACGGTACTTTCCTGAAAGTAGGAAGGTTCGCATTAGCACAAGAACTGAGTACACATACGCACCGTATCATGATGGCTATCAGTGGAGGAAATACGGACAAAAGATGATCCGGGGCAATGCATACCCAAGGTATGTCTAATTAATCATTCAATCTTTTATCTTGCATTTTCTAACAAGTGGAGTTAATAGCTACAGACTCTTGTAATATGTTAACTATGTTGAAGTCCAACAATTCATTACTTAAGAACTACTACCTAGGCTAATTAGCACTGATGCTCATCGGCTGAAATAGAACTGATCCATATACAATCAGTTAAGTCACACATGGTCCAACCAGGCCATGCTCTTTGGGTCTTACAGAAGCTTCTTAATATATAGGGGACCCAAGAAGAGCTTAAAAACGATAGAGATATAAGTGAAGATGTATCCCTTACCTAAATTAACATAGCAAGTACAAATATTGAAGCTTACTTGCTGTAAATAGTGGACGTTTACTGTAGACCTGATCAGTCTTGCCGTATTAGCTATAATACACGTGACTGATCTGGCTTGGTCAGCTTATCTTTCCAAGCCTGAAAGCTGGCTTTCTGGTCACAAACATAGTTTAATGGCAAGTGTGTTTGGGAATACAATAAAAAAATCTGGTGCATGTACATTTATGATTTATTAGGTTGCTGTCTTCCCTGTAGCAGCCGGTTCCTCTCAGGCTGTAGCGTTTACCTTTATGTTGTCGTGTAGCTAAAAGTCACATGCCAGAATGGGCATTTTTAAGAAGTGGAATAAACCCGAGAGGAAAAAGAGGAAAGAGCTAAGCACCATATGTTGAGGGAATCATTACACACTCTGCTTTGTACACTTTGTTTATTAGTGCAGAACACAAATTAGTTTCCTAATTAGCATGCAGAACCATGGATGAGATATTTAATGCTCTCCAGACCCAACTTGCTTTTGAGTAAACCACGGTTCTTCTATTTCTTTTTGTTTTTTTTTGCAATGATTGATGGTATAGTATTTTCTAAAAAGGATGATTGGCGATACATTGTACCATACTAGTGGCTCCAACTAATGGGCTGATACAACATGAATGACTTGATGTCTAGGACTAGGTTTTTATACAAATGCATAATGTGAATATAATATATTTTGAGAGAGAAAGTGTTATTAAATCGTAATAATCGAACCTATTTGTATGGGTACCGATTAGACAACTAATTATGATTAGTCATCGATTAGATTCGATTAGTCGAGTAATCATATGCTGTACAAATCTACTAAGTCGTCCTTGCGTTTGAGCGGTAACAAGATCAGCACTTGATTAGCGACGGAAGAGGAGCAGGACGTCCCAACACCGTGCATTGATCTTGCCGCTGCTTGATCAGCTTCATGGTTCGCAACCACTCCATGTCCAACAGCTGCATGTGTGCCACCTCCGGTCCATCTGCAGCATCTCTAGCTCGCTCTCATGGTTGCTGCCAGCCCATGCCAGAGACAGCTGACAGAAGCTTGAGAAGAATGAATTCTGCAGAGTCTGGCGGCGGTTTATGGATGAATGAATCTGGTGAGAGCCTGCGGTGAGCGGCTT

AGATGATGATAGCCAGCAGTGGAGCAAGCGCGAGCCGGTGCGAGGATGAGAGTTTGCGGTGCTAGCTTCGGCGGCACGAGCGTGAACTCTGGAGTCTAGTCCAGAACACAACCATATATATACTGAAAACCTAATGGATCAAACTACTTAGCCCAACTGTTCAAGCGTTGGTCCTTCCGGTTAGTCATCCACATGCCCTTGATCAGACAATTCATAATCATGATTTAGTCACTGATTAGTCGGATGACTGGGTTTCTAGTAGACCTAATCGTATCGAAGTGCCTAATCGAGGTTTAGGGTTTGGATTAGTTGTCTGATTTAATAACTATAAAGAACGGTAGCACACAAGTATGATGGATCATCGTCTACATTAATGATTTAATGCTTCTACACAGGAGTGTGCAAATAATCCTGTAGATAGATGACAAAAGGTTAAATTTGCACCCCCTGTCTTCAACCATCATTTTGATTGATAGCAAACCTAACGCTCCCACCCCCTCCCTCTCAAAGTAACTCATTGTCTAAAATGCATCGCAGATATTTTCTTTTATCACTGTTGCAGCTTATGCAAGCTCAACCAACAATTGGAGTTTGGGATTGAACATCATGAGATTTCGAGGAGAATCAATTATATTATGCAATGCTTTCAATTTGTGCATGGGGTTTTTAGCTAGGTTGATTTTTTTATGGACTTCCAAACTTTGCATTTCTGCCAAAGAGTTAGAAAACCATTGGAATCCATAGGAATGCAGTGCTATAAAGAAACCTAACACCCACAGACACGGTACTAAAATAGAAAAGGGATACTTTAGAATGGTGCAAATTCTGGCAGCAAGCTAAGGGGGGCACATTTCATATCTGACAGGTAGCAAAAGACCAAGATTAGCATATGGACGCAACCTTTTTTTTTAATAAAATAATTGAACAGTAACTAGTAGCATCGAATGTACAGATCAGCATATGTATGACTAGTGGTGTACAAACTATTTTGTGTTATGTAAATGTTATGGATGCAATTTTCTTTTGTCAAATTTATTTAGTAGTTGAATCTAGATTTAACCAGCAGCTACCGATTTTTCTTATTTTAAGCACATGTAGGAAAATGTTACTTCCTATGCCTTTGTATATACTCACTTCATTCCAAATTTTAAGTTGTTCTGACTTTTCTAGGTGCATAGCTTTATCTACACATCTAGATATATATTATGTCTAGATACATAGTAAAAGCTATGTATCTAGAAAAGCCAAAACAACTTATATTTGGAATGGAGGGAGTATCATATTAGAGGTAGTAGTGTACTATCATTATAGTATTGGTGATGTTAGATCCTCTAGTGTCTAGTGTATGCATGTGATATCCAGTACTTGGGGAAACTCCCAGCATTGGGGTATGATATCTAGAGCATGCCCCTGCATTGGAATCCCCCTTTGGAGTTTGGAGTTAATAATGCATGTCGGCATGATTAACTATTCTGAGCCAGGCAGACAACGCCACGTGTTGTCATGTGTCATGTTTTTGGCTTTTACATGCATGTTGGTGACCTCTTTTGTGAGCATTTTCCCTAGGGCATGGAAAAGTGAACTCTAAACAACTCTTGGTGTCTACGCATGCAGGTGCTACTATAGGTGCACATACCATCAGGATCATGGCTGCCCAGCAACCAAGCATGTGGAGCAAACCAATTCCCAGGATCCACCAT

TGTTCCGGGTAATCTACACAAATGAGCACACATGTTGCAGCACCCATGTCTCAGATTACATGGCTTCATCTATACACATCCAACAGATCGCCGATGCTTCTTTGAGAAAGGTAGAGGTGGAAATACCTAGCCTGACCCACTGTTTTGATGGCCACGGATTGATAAAAGAAGAGAATGATGCCATCATCTCCTCGCTCACGGCCATCAGTGATTATGATGTTGCAACATCAGATGGTGGGCACGCAGCCGTTCAAGAGGACACACCTGCTCGGATGTCCAGAAGCAGCAATGAGGCTAGCCCTTCGATTTCACCTGTACTGTTGCCGGCATCCGATAACCTGAAAACAGATTTCATTGAGCAACTGGAGCCTCAATGGTTCGAGCCTTTGGATTTGGGTTGGTTCATATAATAAGCAGACTGGTTGATCTATCATCTATTACTAAACAGTAAACACATCCATAACCATCCTGCGGTGTTTAGGACATCTGGACATGCACTGTCAGAAAATATGTACTGCTCTGTTTACTATAGCTCTCTCTATGTATATGTTTTAATTAGTTTGAAGCCATACATATTTACTGATCTGGTATATCTTCATTTTTGTGAAGTAACATGTAATGTACAAGCAGGTGTGCACATTTTATATCTTCACGGGAACAAAATTAGGGCGTGTTGGCTTTTACTAAAGCAATGCTGCTAAAGCTGCTGCTGTAGGCGTACTCTGATGAACTACTGTGTGCAGCTGATTCAAACAGGCCATTAGT

>SbWRKY51

ACCTGCTCCCTGCTCAACGACCAATAATCTATCCACGGATTGCTATCGAGAATTCCATTTCCACGCGCTCTGGTAGTCTGGTCTGGTCTGCATATATAAGCTCGTCTCGAGAGCTACCATGATATGGATGAGTTCATCAGTTGCCGCAACACAAACATACATCTATTCTGGCACTCGCTCGATCTGTATCCAGCGAGAAGAAGAGGTCGTCGATGGCTTGCGTGGCGGACCGTGAGGACGCCGTGAGGGAGGTGACGCAGGTGTACGAGCTCATCAAGCTCCAGCAGCCTCTCCTCCTCCTCCACTCGCCGCAGCACCCGCCGCCGCCGCCGTCGACGACCTGCCAGCTGGCGCAGAGCCTCCTCGCCAAGGCGCTGCGAGCCCTCAACGTCGCTCTCTCCGTCATGAAGCAGCAGCCTGCTCCAGTGACACCAATAAGCGTCATCAAAGCTGAGCCTCATCAGCTCTCGCCGCCCTGCAGCCTGGCGTCTGCTGAGTCCCAAGCCGCCATAGTACTCAGCACGGCAACAAGAGGCGCCAAAAGAAGAAGATCAACGGAAGGGAAGAAGAAGAATACCTCGTCGTCCTGGGCGACGGTAACCGCCGTGCCCTACGACGACGGCTACGAGTGGAGGAAGTACGGCGAGAAGAAGATCAACGGGACGCTCTTCACCAGGAGCTACTTCCGGTGCACCTACAAGGACGACGCCGGCTGCCTCGCCACCAAGCACGTCCAGCAGATGGACAACAACAGCGATCCGCCCATGTTCCACGTCACCTACAACAACGACCACACCTGCAACACCAGTGCCAGAGCCAACACCGGCAGCAGCAGCAACCTCGCTGCATTGCTAGCAGGCTGCTGCAACATGAAGCAAGAACCAACTGGACATGCTGCTGCTGCGGCGGCGGCGACCATGGACATGAAGCAAGAAGTCCAAGAACCGCCGCTCCTGCTGCCTGCTCTGGTGGACCTCCAGCCTTCTGCCTGTTTTCATCATGAGCAAATTCCACGGTGCCAAGAACCGCTGTTTCCTGTAAGTATGGAGCAGCAGTTCGTTTGTGGTCCTTTTAGAGACGACGATAGTGAAATCCCGTCGGCTACTGGTTCGTGCATCTCCGGCGAGACCAGCTGGGATGGGTATTCTGGACACATGGCGGCGGCTGAAGATGACCCTCTCCTCGATCTCGAGCGTTTCCTCTTCATGGACTACTAGTACTAGCTAGGCTACTAGCTGGATCTGAAACCTAATTAACAAGATCTATGAGAGTAGAAAGTATATAGTTTGATCTTCCCTTTTTTAGGGCAGTAGTGCCAATAATACAGTGTTCTGTAGGGAGGAACAGAAATCCAGTAAAATAAGAGTGCATGAGATGTAAAATTTTGTGGTCCTTAGCAAAGGCAGTGACATATTGGGGGCGGAGTCCCTAAAACTTTAGTAGAGAGACTCATGACCTTCGAATTCCTCTCCAATTTTTTGTGAT

>SbWRKY52

ACAAACAAGCATCAGTTCTCGCAGCTAGCACGTACTACTCCATATATATATAGTCCATATACATCCAGCAAGAACTGAAGAGGAGGTCGAATCGATCGATGGCTTGCGTGGGGGAGCGTGAGGCCGCGGTGAGGGAGGTGGCGCAGGTGTACGAGCTCATCAAGCTCCAGCAGCCTCTCCTCCTCCTCCACTCGCCGCAGCACCCGCCGCCGCCGTCGACGGCCAAGCTGGCGCAGAGCCTCCTCGCCAAGGCGCTGCGAGCCCTCAACGTCGCCCTCTCCGTCATGAAGCAGCAGCAGCCTGTTGTCGTCGTCAAAGCTGAGCCTCATCAGCTCTCGCCGCCCAGCCCGGCGTCTGCCAACTCCCAAGTCGCCATAGTACCCAGCACGGCAACAAGAGGCGCCAAAAGAAGAAGGTGTGTGTGCACTGCTGGATCCTTCTTCTTCCGTTATATTTTCTGAATTTCTGTGTGCAGTTTTCAGCCTCTTCGTTCGATCGCTGACTGTTGCTTCCAATTGAAACTGCGTGCTGTAGATCATCCGTAGCAATAATGGAAGGGAAGAAGAAGACCTCGTCGTCGTCCTGGGCGACGGTAACCGCCGTGCCCTACGACGACGGCTACGAGTGGAGGAAGTACGGCGAGAAGAAGATCAACGGGACGCTCTTCACCAGGAGCTACTTCCGGTGCACCTACAAGGACGACGCCGGCTGCCTCGCCACCAAGCACGTCCAGCAGAGGGACGACAACAGCGACCTGCCCATGTTCCATGTCACCTACAACAACGACCACACCTGCAACAGAGCCAAAGCTGCAGGCATAGCCAACAACGGCAGCAGCAGCAACAACCTCGCTGCATTGCTAGCAGGCTGCTGCAGCAATGGCAGTGGCAGCGGCAGCGGCAAGGGACTGACGACGATGACGACGACCAATGCTCGGCCAACCGAGCATGCTGCTGCTGCTGCTGCCATGAACATGATGAAGCAAGAACCACCGCTGCTGTTGCCTGCTCTGATCGACCTCCAGCAGCCTTCTGCCTGTTTTCCTAATGAACAAATTCCACAGTGCCAGAAAGAGCCGCTGTTTCCTACAAGTATGGAGCAGCAGTTCGTCTGTGGTGCTTTAAGAGACCATGACTCTCCTGTCGATGGCGACATCCCGTCGGCCACTGGTTCGTGCAACTCCGGCGAGACCAGCTGGTGGGATGGGTATTCTGGGGACATGGCAGCGCAGATGGCAGCTGAAGACGACCCTCTCCACGACCTCGACCGGTTCCTCCAGTGCGATAGCTTCATGGACTACTAGTAGTAACTATAGCTCTGCAACCTAAGCTAATGAACAGGGTTTATGAAAGTAGAAGTATAGTTTGATCTTCCTTTTTTGTTTTCTTATAGCTAGCTAGGGCAGTAGTTAGTAGTGCCAATAATAATGCAGTGTTTTGTAGGGAAAAGATAGCGGTTTATGTAAAATGAGATGATGTAAAATAGGATCATGCATGAGATGTAAAACAACGACGATTTGATATGTATAATGGC

>SbWRKY41

GACGTCTATAAATATTGAGTGCGGCGAGCTCACTGCCTCTGCATCTCAGTATTGCTACATACCCTCGTCTCGCCGCCCCTCCGCGGTTCAGCTCGCGGGCCACAGAGACGGACCTGGACACCAACACACCAACACCGAGTGAGCTGTGAGCCTCAAAGCCCCTCGAGCCCTCGAGAGTGTGAGGCACGACTAGCATAGCAGCACTCTAGCAGACGCATCGGTCGATCCAAGAAAGCTTAGCATCGAGCAGCGGCAGCCATGACGCTGGACACCCCGGCCGCCGTGGTGCTGGAGCTGATGACCATGGGGCAGCAGTCCGCGGCGCACCTCAGGGACCTGCTCCGGGCCTCGTCCCCCGCGGCGTCGTCGCCGCACCAGGAGCTCGCCGCCGAGATCCTCCGCTGCTGCGGCCGCGTCATCGACGCGCTCAGGGCCACCACCAACGGGCGCAAGAGGAAGGCGGCGGCGGCCGAGTACCACCAGGACGCAGCAGCAACGGGAGGCGCCACCTGGTCTCCTCCTCCTCCTCCTCCGGGTCCGCCACTCAAGAGAAGGTGCGTGGCGTGGGGCTGCCGTTGCCGTCTCCCTCCCTCTTGCACGCACAACAACAGGCTTCTCTGTTTATTTGCTTGCTGGCAACTGAAAAAGCAGCACGATGATGATGAGTATTGCTTCGCCGTTCAATTTCACAGGGCGCGCGGCGCGGAGGCGACCAGGGAGGTGACCAGCGGCACGACGGTGGACGGCTTCATCTGGAGGAAGTACGGGCAGAAGGACATCAACGGACACAAGCACCCGAGGCAAGTAGTTTCACAAGGCTCAGGCGCTCATCACCAGCTCAACTCCGTTCCGTTCCCTGGTGGCGATCGACTGACCGACCGACGCGTGCTGATCTTATTCCCCGTGGGCCTTCTGCAGGCTCTACTACCGCTGCGCGCACAAGGACCAGGGATGCAACGCGACCCGGCGGGTGCAGCAGACGCAGGACCAGCCGGCGGCGTACGAGATCGCCTACTACGGGGACCACACGTGCAAGGGCGCGGCCACCGCGTGGCAGCAGCTGGGAGCGGCGCCCGCCGTCGTCGACTTCGGCTCCAACTCCTGGGGGTCCGCGGATGCCAACAACAACGGAGGCTCGCCGGCGGCCTCCATGTCGCAGGGAGGGTGGTCGCCGTCGGCGTCGTCCGAGGTCGGGTTCGACTTCGAGGCGCTGCACGAGTGGCACGACACGGCTGCTCCTGATCCGGTGATGGAGTTCCTGGACGGCTGCTTCGGATGGGAATCCGTCCTCCAAGACAGCTCCGACTTCGGCGGGCTCCTCCTCCATGACATCGCTACGTTTCAGTAGTAGATTATTTTTAGAATAGCAGCAGCAGACCAGCGGTAGATCTGGACTGGACATGTGTTGTCGCCGTCGCTGTAGTACGTGTACTCCGTACGTGAGAACAGTCAGTCCGACCGAGCAGTAATGAGTGACAACCTACGAGGGTCACAGGCTCGCTCATCAGATGGTTGCCGTAGAGCACACTAGTAACAGTATCTTGTTTCAGGCAGAGGGTTCAGATTTTTTTAAAAAAAAAAGATATTACGTTCTCAAAAAGAGAAATGATATTGTTCGCCCGTTCAACGCTACTTTAACTGTTTTCAGTAAATAAATAATTTTTTTATAACAAATTAGCATAAGCATTAGTATAAACCAA

ATTTCAGAGAATAAAAAGGGCCAAGTGTTTTTATATTTTTATAAAAGAACTTCTACTTTCGCCATTAGAAAATGGAGCGGAGCAAAGGAACAAAGTGACGTGTACATGAGCAAGTGGCAGTCAACAGTCAGCACTGCTTCCGGTAAGACCGGCATCATGTGAAAACCAAAACGCTTCGAATTTGGACGCAGACCTGTCGCATCGGTTTTTTTTTCATTTTTTTAATGAGGGATACCTGACGTATCTTTGACAAGCTAACCAAAACTCTCTGCAAAACTCTCCTGGGCATATTTGTGCAGTGCAAAGTTCCGCCATGATCCAAATGTTAAGTTCTCTACCATGAACAAGTCAATATTAGTGACGTGGCTTTTTCGAGAAAAATAGACTCCTATATCATTAGCGGACGTTTGTAGAACACGGATTGAACTGTATATTTCAGAAAGAACATGCAAAACTTAAACTGCATACGACTAGATTGAGTGTAATAGAGCAAAACTGCATACGACTAGGGATCAACCTCACCTAATTGTTATCAACTTATCGTCCGCCTTGAATAATCGAGAGCAAAATGGAAGCTACAGAGTAATAACACTGATTGTTTTCGCCCACTTCGCTAAACATTGAACGCATCATTTAACTGTCCCCATGAAAAATAAACAACAGACAAACTGAACAGCTAACTACATGACCTTTGAAAGTCGTTTATTGATGTTGCTCAAGCCTATCATCATCTATATTACATGAATAAAGGAACAAAGGGGTACGGGACGAGGGCAGAGTCTGAATCCATGTGTCATCATGCAGACACCTTAGACGCTAGAACTGAACTTTGCCTGAATTGACGGCTGATGAAGGAAATCGAAAGCAACAGGCCATAGTCTTCTGGGTCTACGTTCCAGTGATCATCTGATTCGTCCCAGTGCT

>SbWRKY54

ACAATCCAGCTATTAGCTACTTATACGTATACATATATTGAGAAACAAACACATGATGAACATCCTTGAATCTTCCAATCTTGGCGGCTACAAAGAGGTCATCAATGAAGTTGAACACCAAAGGGCTCTCATGATGAACCTACATGACCTTGTACTACCAATACTTGATCCCTGCAGTGGGCAAGCAAAGCTCATACAACAACTCTTTGAAGAAGTATTCAGTAGCTCAGGTAAGATTATTTCTTCCCTAGAACTTGGTGATAACAGCGAGAAACAGGCCATTCTTATCAAGCATAAAGGAAAAGGAGGTAAGGATAACGTGGAGAATCACATATTGGAGGAGAACAACAAGGACCGTGGAAACAAGAGAAGGTAAGTTGTAATAGAAGCATCCTTATTTATCATATATATGCTGGGACACAAAATTTTGATTGCTTCTCTTGCTTGGTGGTGGTAGGAAGAATGCAAATCACATAAGTTCAGTTGTGACACAAACACCATACTTTGATGGATGTCATTGGAGGAAGTATGGGCAGAAGTGGATCTCCAGAGCAAAGCATTCTAGGTACATGGGAAAAAAGCATTTGCACTAATGTATGCATTTGTTATGATCTATATTGAATTTGGGTATCGGGATTCTCATCTATATGTGGATAGATTTTTGCGTAGTGCAGCTCAAAAAGGCATGCATATTACATATCTAATAGTATAACTGTAAACAACAAAATTTATATTTTCGATATATCCAAAAGAAAGGTCTAATGTCCATATTGCTAACTAGGAGCTACTATAGATGTGCCTATAGTAAAGAGCAAGGGTGTCCTGCAACTAAAACAGTACAGCAGAAGGAAAATGATGGCAATGGAACGGTGAGGTTGTTCAATGTTAACTATTATGGCCAGCACATTTGCAACAGTGATGGCATAGTTCATCCACATGTTGTTGGGGCAACACAGGACAGCATGCCAATCGTCAGTCAAAACCAAAACAGTAGCTCAGTGTTTGTCAATACCGATGTCCATGGCGTTCAGGATGAAATCTTTGAAAGCTTATTCATGGTGCCCGACATGCCAGAATATTTGACAGAGTTCGTAGATGTTGAAATGGCAAGAGCATTTGAGATTACCCCTATGAACTCGCCAATGATCCCTGAAGACATATGGGCGTGAAGAAGCGAGAGGAATCAAAGCAAAGTCCTTTTATTCAATATATGGGTGTAAATCATGAGTTCTGAAAAGGACATTGGTGTGTTACCCATTAGTCTACACTATACATCGTACAAAGGTTGTATATATGGTCTAGAATGCTGATGTACTATCTAGACTTTGCTGTGACATATATTACTGTAAAGCATGCATCATGATTGTATTACTTGTCATTCCAAGTCTGTAAAAGGACACCCTG

>SbWRKY70

CGAAGTCCAACTATTATAGCCACATATATTTGAGAAAGCATCAAATGAACATCCTTGAATCTTCCACTCATAGTGGGTGCCAAGTGGTGATCAACGAGATTGAACACCAAAGGGCTCTCATGACGGACCTACATGACCTTATCCTACCAACACTTGATCCCTGTAGTAGGCAGGATGCGCAGCAACTCTTTCAAGATATATTCAGTTCCTCAAGTAAGGTTATCTCCTTTCTCCAACTTGGTGATAACAGTAAGAAACCGGCCAATCTTATCAAATATAGAAGAAAAGGTGGTAAGAATAACGTGGAGAGTCACATGTTGGGGGACGAAGCTAAAGAAATTGGAAATAAGAGAAGGTATGTAGCAAAAGGAGCATATATATGTTTAGTTATATATCATTTACTAGGGCTTAACATATTTATCCCTACTCTTAGGAAGAATGCACAACACACAGGTTCAGTTATGACACAAGCACCACACTTTGATGGATATCAATGGAGGAAGTATGGGCAAAAGTGGATCTCCAAAGCAAAGCATTCTAGGTATGTAATAAAACATATGTGTTGATGTACTTCTTATATAATAATATTTTCTATACTGAATTCAGATTCTCATGTGTGTGGATGATTAGCTTTGTGCACATATACTGTAGCTCAAATTGCAGCCATACATATTCTCATCTATGTGAATGAATACTATTTTGCATATTTTGCCTTTTGTGTATAACAAAAAATATATATAATGAGGTCATGCTTTTTCAGGAGCTACTATAGATGTGCCAATAGTAAAGACCAAGGGTGTCTTGCAACCAAGACAGTGCAACAAAAGGAATCAGATGGCAGCACTGGAACAGTGAGGCTGTTCAATGTTGAGTATTACGGCCAGCACATTTGCAAGAAGGACGACATAATCCATCCATATGTTGTTGAGACAACAGATTATAGTGCACCAATTGCCAACTATAACCAAAGCAGTAGCAGCTCAATGTTTGTTCATAATGATGTCCTTGGAATTCACGATGAAAGCTTCGAAAACTTTTTCATGGTGCCAGGAATGCCAGAATATTTGACAGATTTCACAGATTTTGAAACGGCAGAGGCACTTGAGGTTACATCTATGATAATCTCCGAAGATATATGGGCGTAGTTAAAAGGAACTCCTTTGAAGGTGTACGTCAATCGGTTCACATGCAGACAGCCATTACTGCTTCATGAGTTGTGACAAGAAAGGGACACAAGTTTGTTACCTGTAGAGTACAGTATGTGTGATAAGCAAAGAAAGTGGATGTGCAGAGTGGACAGAAATTTATGTCTTAAAAGTTGTAAATGCTGTCCCACATGCAGAAAGTTGATGTACTAATATCTAGACTTTCCTTTGAGTGTAACTATTAACCATCCCATTCCTACACATCATGCAAAGTCGTCATAAGAGGTAAAGTGGTCCTAGAATAGTAGCTTATACTAACTTGTTAACGTTTGAGGGAGAGTACTAGAAACATGAGAAATAGTAATAATTAGCATGACAAATAATGGATGTCAGTCAAGAAAAACTCCATAGTTAGGCCCTGTTTGGTTCCCCTTGCTAAATTTTAGCTAGCTAAATTTTAGTCACTTTAGTAGCTAAAGTTCCAAACACATTGACTAAAAGAAGCTAAAATAGTTTAGTTTCATTAGTCATCCAAGAGTAGCTAAAATAATTTTA

GCTAACTAAAATTTAGAAAGGGGAACCAAACAGACCCTTAGTCACAATTTCACACATTGATATGAGAACTGTCAAAGCTTTGCTACATTCTCTATGCATACAGGGTGCAGCCTAGTAGCTATCAGGATGGTTAATTATATTCTCCCATCCCATGCCTCGTTGACTTGACTTGATCCAGCATGTGTCTGTAGTTGAACTGAATAAATAACATTTGACTGAATATATATAATTTGTGGACCGGAGGAAACATAAACATCTGGTCGATTTGGAAGCTACTTAAAATTGGGTGGTTCTGTTATGTTCTAAAATTTGGTGGCTCTGCTTTGTGT

>SbWRKY55

CTACGACTGCAGAATCTAACCATGATCTTTCCAAATCACTAGTACATGCATGAATGCATCTTGTACTAGTAATAATAGTAGTAACAGAGGAGTAGAGGACCAATGCAACCTATAAGCGCGTGGTAGCTCTGCAAAGTCTGAATCCTTGCCTGTTTTCATGTGCTCTGCACCTGCCTACGCACGCAGGCACAGCAGATTATATTACTGCGCAAGTTGGTCCATTAATTATGTTGTCATGCCGTATATAAGTAGAGCGGACTAGCGGTAACCTAATCGCCAATTCCCCATAGCCAGCCAGATGACTAGCTTCTTCTTCCTTGCTTGATTCCTTCTCTGACAATTTCCTGCAAAGAACCTGCAGCTAAGCTAATTAAGCTTAGTAGCTTAGTTTCTTCCCACATCACCAGCTCTTCAGATCCAAGAGCAAGCTAGAAGAAGAAAACTAGCAAGGATGAAGCACCAACAGTATAATAGCAGATGTCTTGCTGAATCTTCAGCTTCTGATCACCGGTCGGCGGTGAAGGAGATCGCCAGGGGGCAGTCTCTGGTGACACAGCTGCGAGCAATCGTGCTCCCTGTGCTGCAGGCCGACGAGCGCTCTGAGCTTGTCGCCCAGATGTTCCAGAACATCCTGGATTGCTCCAGCAAGGCCATGGCCGAGCTGCAGATGCATCAGTCTCGAAGTACTCGACGACCTCATGATGATGATGATGTGCTGGTGGATGACAAGAAGAGAGTAAAGAAGATTTCCTCTGTCGACTGCAAGAATGAGGAGGGTGTTACTGCTGCTAAACCCCGTCATCAGCACAAGAGAAGGTACCATGTGAGCTAATGAGTTCATTTGAAATGCTTCTTGAGGTTGCATATGATTATTAATCCAGAAATAAAATTTTAAGCAGGAGATTTGATGACTCTGTGTCACTTGAAACACCTGTGCCGCACTACGATGGCCGCCAATGGAGGAAGTATGGGCAGAAGCACATCAACAACACCAAACACTCAAGGTGAATTTATCCTTCTTATTAATTCGAAAGATATGTATTTCCTGGGTTGTTCTTTCGTTCCTTTTTTTTAAAAAAAAATCCTCCCTATTACCAAGCAATTATGCCGCATGTTGCTCCAATGATGTTAAGCAAGCTAGGTGGTAGAAATTCAGTTTGGGATGATAAAGGAAGGACACTGCCATTTTCTTTTTTTGTACTGAACTTGATCTATAGGCCGGCTTAGGCTGATCCTAGATTAGCCCATGACTTTACTGTGTTAATTAGTATATGCACAAAGTTAGATATAGTGACGCAAATTTGCAATAAGCTGTATTTGTCACCAGCTAATTGAGGTACATATGCAGGAGCTACTACAGATGCACCTACAGACAGGAACAAGGCTGCAAAGCAACTAAGACGGTGCAGCAACAAGATGACAGCAGTGGCGCCGATCATACCCTGATGTACACGGTCGTTTACTATGGCCAGCATACTTGTAAGGACAATGATGGTGCTAATTCAAGCCCTGATGACTCTGAAATAAACACTAGGAGCAGCAGCGACAGCCACTCCAGCATATCAAGCACCTGTACAGATCCTTGTGACCATCAGAATCAGACATCTCTACATGAGGATAAGCCGTTCGTCAACAAATCTGAAGAGTTGGTCACAAAGGACATGTATGAGCCATTCGAGATGACTGTGTTTGCCCCCTTG

GATTTGGATAGTTGGGAGTTGGATGCACTCCTGAGATTTGGACCCTGATAACTTAGAAACCGATGAAAAGCTAGCGACAATTAGTCGATAATTTGATATGTTTTGAAGAGAGGTATGTAATAAGGTATATATCTCATCAGGAGCTTGTGATTAGTTGTCTAGTGTTAAAAATATCATTTGCACAATGTTCAATACTAAGTATATATACGGTCAATTTTGTGTCCTTCTGTATCAAAAAGTTATATATGAAAAGAAGAGCGCGAAAGAA

>SbWRKY69

ATGGCCGAGCTGCAGCGTCATCACCAATCTGATGATGGTGCTCGAGCTCGACCAGATGATGTGCTCGTTGATGACAAGAAGAGAGTGAAGAGGAGTATCTCTGATGACTGCATCAGCAAGGAGGAGGATGTTGTTAAGCCGCGTCATAAGCAGCTCAAGAGAGGGTATGCATGCATTTTTCCAACATCAATCGATGAGTAAAACTACTGCTTTTGTTTTTTGTTTATAGTGCTTCTGTATATGTATTATGTGAGCATGGGATTAAGCTTTCTATCGATTTTGATTCTTTTATTGGCAAACAAATTTAATTTCGAGCAGGAGATTTGACGAGTCCATGTCACTTGAAACGCCCGTTCCACACTATGACGGACGGCAGTGGAGGAAATACGGGCAGAAGCACATCAACAAATCAAAACACCCAAGGTAAATTTTTCCTTCTTAATTGAAGGGACACATCTAACTCTCCTTGGTGGTGTACTTCTGACGTTCTGAGACTCCTTGATTTCTTTCTCTTTTAACACACCTGGGGATGTATGAATTAACAAGGTTCATCAGAAAGAAATTAAATCAAACACAAAATCATATATATGCATGTGAAGAATAATATCAAGGCAGACCAGATCTTGGACTCTTATTATGCGCTCACTCTTTACTAGAGCTAGCAGCGAATGAAGTTCATCAGCCATGAAAAAAAAAGAAAACAAAAATAAAACTAATCACTAAATCATGTGCATGTTCAGAAACCTAATAACGCTGCAAATCTATAATGGGTTGTACAATCACTAAATGGATTATACATATATGCAGAAATTACTACAGATGCGCCTACAGGCAGGAACAGGGCTGTAAAGCAACAAAGACGGTGCAACAGCAAGATGACAGCACAGGCACTGATCATCCCGTGATGTTCACAGTCGTCTACCACGACCAGCATACTTGCAAGGACAACAATGGCATCAACTTAGGCATCGATGACTCTGAAACAAATAGCCAATCCAGCATATCGACAATCTCTACAGATCCCTATGGCCGTGAGACACCATCCCTAGATGGCAATAAGCTGCTCGACAAATCTGCAGACTTGATCACAAGGAACAGCATGTATGAGCCAGCCGACATGACTGTATTTGAACCTTTGGATTTGGACAGTTGGGCGTTGGATGCATTCTTAAGGTTTGGAGCCTGATAAGTGATAATTCACATATATATCGATGAAAAGCTAGTGATTACCGCAATCGATGTGTCTTTGACGGCGCTGCACCTGATGTGAGAAATGTGTTGGCAATGGCCATTGAGGAAACGAGGTTATGGGGTTTGGCTAAGGGCATCACTTCTTTGATTGCACCACTCACTGTGACTAGGTCAGGCCTTGGGTGTGTGTGGTCGTTTTGTTCTTATATAAAGGCACTTTTCTATCTTGTCACCCTCTTAGGCGTGAGTGAGTGTTTGAGTCTTGGTGTTTGTTGGCTTGTTTTTCTTTGTTTGGGTTCTTTACCTTTTTTACATCTTCTTAATATAAGATACACGTACAGCTCTCCTATGTGTCGGGAAAAAAAAGACCAAGTGACAACTAATAGTAGTGCCTCACATAAAACCCCAGCAAAAACCACAACCATGCTTTGCACTAGTGTTGAGAGTTGGAGGGTGTGAAGACTGAAGAGTATTAGTACATGATGAATTACTGGTTGAAATCAATATGC

CTTTCTCAACTATAAAAGTAAACAGAATAACTGAGCAACGCCACCCTTCTATGAATCAGCTAGCATTTAAAATGCATGGGATTCCCACTTTTTTTTTGAAAATTCAGAAGAGTTGCGTTTCATTTCATTGTATTAAAGAGAA

>SbWRKY10

CGACAGCATGGTCTAACGCTGCCCTATAGGCTGGATCGATCGATGGCGTCGTCGGGCAGCGACGTGCCGGCGGCGAGGGCGGCCGTGGCGGTGAACGATCTGATCCAGGCGCGCGACGGCGCGGCGAGGCTCAGGGCCTTCCTGCTGCAGCTGGACGACCAGCGCGCGGCGTGGGCGCAGCTGCAGATCGACGGCGTCCTGACCAAGCTGTCGAGCGCGATGTCGGCGTTGGATGTAAGCGATGCCGCCGGGTCGGACGACGGTGCGAGGCCACGGCCGCAGTCGGGGAGCTCGTGCGGGAACAAGAGGAAACAAAGCTCCAGCAGAAGGTGAGCACCTCTTCATACTCTAAATTACTAGCTTTTTATACATGTACAGCTGATCTTTTGACTTGTTTCTCGTTCGTTCCAATTCGATCTGCCCATGCTTAATTATTTCGGCAGATCACAGCGTCCATCTGACAAAAAGATCACTGCTAACCTGGAAGATGGCCACGTATGGCGGAAATATGGGCAGAAAGAGATTCAAGACTCACCCTATCCAAGGTATATATGTACATACTACATACTTCGTGCTCATATGTCCAGTAATTAATTCCAGTTATATATATAGTGGTATGGCCGAACGAAAGGCCATCAAACAGAGATCACGGAACCGGAACGTGTTTGCTTTGCAGGAGCTACTACAGGTGCACGCACAAGACAGACCAGGGCTGCAGCGCCAGGAGGCAGGTCCAGCGCTGCGAGACGGACACGTCCAAGTACGTCGTTACCTACTACGGCGAGCACACGTGCCGGGACCCCTCGACGATCCCGCTCATCGACCACGCCGCCGGCGCCCTTGCCGAGCTCGACCGTGCCAACAACCTCATCAGCTTCGGCCCCAGCGGCACCAGCAACGACGCAAACGCAGCAGCCGCGGCCAGCAACGCCGGCGCCTCTTCGTCGCAGTACCTGCAGGCTATGGGCGGGAGCGCTGCTGCTGATCAGCTGTCCACGTCGTGGTGCACCAGCGACGACGTGTTCAGCTCGTCGGCCGGCTCGTTCATGCAGGTGGACCAGCTGATCGGCGCCGTCGTGGGCGGCTCCGCCGGGGTCGTGACGTCAGCGGCGGCGCCGGACCGTGGCGTCGTGCTTGGTGGCGTGGCGAGCGGCGGCAGAGGCACCGCCAGCTTCCCGACGTCTCCGAACAGCCTCGGCTTCGTGGTGGGATCGCTTGGGAGCATCGGCGGCGGCGGCGAGGACGACGACGATATGTTTAGACTGGATCCTTAG

>SbWRKY21

TTTCCTCCCAACTTCCCCCCGCGGCCACCGGCCACCAATAAAAACAAGCCGCAGCACAGCTAGCTGCTCCCTCCCCGACGACCCCATCGCTCCAGCTACGGCCGGGCAGGTGGGCATGCATGCACTTGCACCGCTGGTTTGGTAGCTAGATAGCTAGCAAGCGGGCATGGCGTCTTCCGCTGGCGGCGGCGGCCGGCGTTCACCGCCTGCTACGGCGGCGCCACGGATGGTGATGGACAGGCTGATGGAGGTGCACGAGGGCGCGACGAAGCTCCAGACCATGCTGCAGGAGTCGCCCACGCCTTCGATCGCGGCTGCGGCAGGGACCACCAGTGAGCTCAGACTGACGATTGACAGGATGCTGAGCAGCCTGTCGAGCGCCATGTCGGCTTGGAACACCACCGGCGCCGCCCAGGGACCGGGACAGGGGCGGAGGAGGAGGCGAGGCGAGGCGGCGGCCGGGTCCGGGCCGCAGCGGCGGAGCAGCACCAGGAGAAGGTGAAAAATAAAGGCTAATCAAAACCCTTAATTTTTAACTGCGCAACTAGCTAGCTCGCCGTTTTGCATGCATTATTGGGATATACTCCCTCCGTCCCAAAAAGAGTGACGTTTTTGACTTTGAGACATCGTGTTTGACCGTTCGTCTTATTCAAAAATTTTATGTAAATTACAAAATAAATTAAGCATGAGCAAAGTATCTCTAAGTCTTAACAAAATATATATTATTTATGTAAAAAATTTGAATAAGACGAATGGTCAAATAAGATGTCTGAAATTCTAAAACGACACTCTTTTTGGGACGGAGGGAGTATGCATGAGCCACTACCTATGCTGCTTACTTTTGCAATTTGAAGGATACCTAGCTAGCTGTATAAAACTTTATGTGCCAATGATAGGGAGAAACTTAATAAAACTTGCATGCGCTTTCTTCTTTCCAACAAGACTGTGATTTCACTTGTCAAATCTGAAAGTTCAAAAGGTTAATCTATCTAGGTTCAGTGCAAAAGTACTACTTGAATGCATGTTTAATTTCTATAAATAAGCATGCATCTATATATCTATATATAATTCACATGAAGTAGCGGTCCATCCAATTAAAACTTGGTGTATGATGGTTATCGATCTTTCTTTTTGTATGTGATCGGGATGATGCATATGGCAGATCGCACAGCCCCTTCGTGAAAATGGTCACTACCAGTAAGCTCGACGATGGCAAAGCATGGAGAAAATACGGTCAAAAACGTATTCATGAGTCCCCTAATCCGAGGTATGTGTTGGCCTCTAATCTTACTCAAGGCTCCTTTTGTATGTCATGCATAGGTATTACATGTCGTATACATAGTATGTCATAGTGATGACAGAATCATTGAGTATACTTTCCCCCTATACCATAATAAAGAAAAACTAAACTAAAGCTATGCATACCAAACACTTGCAACTGCATGCTGGTTATATATCCAGTTAATCATAAAGCACTATATATATATGTTGCTTTGGACCAAAAGCCTCATGCATGTACATTATTATTCATCGATAAATATAATATGGCACTACATGCATATGGTGTTTCTATATGTAGCAGTGGCACTAGTTGATCACATGACCTTTTCTAAAAAACAAAATAAAAACTAAAGAACCACTACTATAGCAGACCAGTTCTCTCTGCATCTGCATGACTGCATGCATGTACATGTGCATAT

AGGAGCTACTACAGGTGCACGCACAGGCCAGAGCAAAGGTGCATGGCGACAAGGCAGGTCCAGGCCTCCGATGCCAACCCGTCGGAGTTCATCATCAGCTACTACGGCCAGCACACCTGCCAGGACCCCTCCACCATCCCGCTCGTCATCCCAGACACCGCTCCGCCGCCGGACTGCGCGAACCTCATCAGCTTCGGAGGATGCACCACCATCGCCGCCGGCGCATCGTCGTCATCCACCACCACCACTGCTGTTCCTCCCCAACAAGCCTTAAGCTTTGATCCGACGACGACTCCGATGTTGATGTTGTCTCGCTTCGGCTACAGCTCCTCTCTGCCGGCGGCGCAGGCGCAGCAGGACTACCGCTGCGGCAGCGAGGAGGTGCTCAGCAGCAGGAGCTCGCCGGCCGCGCAGCTCGCAACCATGGTGGTGGGATCAGCGGGGACGATGGCCTCGTCGTCCACCGTGGGGTCCGCGCCGGCGGAGTACTGGCCGGGAGGGACCAGCGGCATGGCGTGTGGCCCTGGCAGCTTCCCGTCGTCTCCTAGCAGCCTCGGATTCATGACCGGCTCGCCGTTTGGTTCCTTTGGCAACGCCGGGGATGACGACCTGTTTGGCTTCGATCCCTGACAAAGGCTGATGTATGCATGCATATCCAGCCAATGCCACGTTTGCAGTTTGCATGCATGCATGCATGCACGCACGCACTACTACTTGCACTTGCACAAGTCGTACTAGTAGTAGGCTAGTAGTAGCTAGCCATGCATGACATAGTTGGTATCCACATGGATGATGTATAATGGTAATTAATACAATAAGGCAAGACCTCTAGTTCATCCTGAACAGGTTTTGCATATGTATATTTGCTGCCCACGTATATAGGCCAAAAGGATATGAATTGTATTGTAAAGGCCTTATTTTGTGCATTTGTACATATATATATGCATGACTAAATACAACGAATATGTTGTCTCGTAGGAAGTATACAAGTGAACTGAGCTCAATTGATCCTTA

>SbWRKY72

GTAGACTCGCGCGCCCCCCTCCCTCTGCTTCCATAAATGCCAGCACCTACCTCGACCCTCGAGGGGTTTCTTCCTTGCTTGCTTTGCTGCTTGCTGCTGCCTCCAGTGAGTCATCACTGAGCTGTGAGCTGTGAGCTGTGGACCTGTGGTGGTCGTGTCGTGATCTTCTTCTTCTTCATCTTCTTCTTCCCTCTTGCTGGTTCGGTTGCTGTGTGGCCATGGCAGCGGCGGCGCCGTACGCGCGGGTGATGGAGGACATGGTGAAGGGGCGGGAGTACGCGACGCAGCTGCAGGCGCTGCTCCGGGACTCGCCGGAAGCCGGCCGCCTCCTGGACCGGATCCTCCACGCCATGTCCCGCACCATCGACACGGCCAAGGCCGCCGCCGCCGAGGAGGAGGAGGCGTCCGAGGTGCAGAGCGACGTCACCTGCGCCGGCACTGCCGGCAGTAGCAAGCGGAAGGCCGCTGGTGGAGGGGACAAGAGGGCCTCCTGTCGGAAGAGGTGAGCCACAGGCCTTCGCCGCCGAGTCCTCTTCTCTTGATCTAAAAATAGTTTTTTTTTTCTTTTTTCGCGATCGGGCCAAGCACGTACAGTATGATCTAAAAAAATAGTTTGCTGATTTTGTGCAAACCACTGCCGGATGTGTGTTGTAGAGGCCAGCAAGGATCATCGGTTGTGACGAAGAACATCAAGGATTTGGAGGATGGGCACTCATGGCGAAAGTACGGACAGAAGGAGATACAAAACTCAAAGTACCCAAAGTACGTTTCTACACCTATTAGATCAATCATTGTTAATCAAATCAAATATGGCAACCAACCCAACCTATGCATATGAGATGATGATCCTTTTCTTGGCACGAATTTCTGATTCCTTTTCATTTATTTGGTCGATGATCGATGATGCTCGGCGTGCGCAGGGCCTACTTCCGGTGCACGCACAAGTACGACCAGCAGTGCGTGGCGCAGCGGCAGGTGCAGCGCCGCGACGACGACCCGGACACCTACACCGTCACCTACATCGGCATGCACACCTGCCGTGACCCGGCCACCGCCGTCGCGTCGCTCGTCGTGCACGCGGCCGGCGTCACCGGCGACGACCTCCACCACCACGCGGGGTCCCGCCTCATCAGCTTCGCCGCCGCCAACAACAACGCCAGCGCGGCGACGACCAGCACCACCACCACCGGGAACACCACCAACCAACAACTGGCGGTCCTGCAGCCCCTGAAGCTGGAGTGCGGCGGCGGCGGCGAGCAGGAGGAGGTGCTGAGCAGCCTCACCCCGGCGGGGAGCTCGGCGGCCGCGGAGGCGATGCGGAACGGGAACGCCGCGGCGGCGGCGGCGACGACGACAGGGCCGGAGCCGGACCAGGGGGACGTGACGTCTGGCCTGCAGCTGCAGCAGTTCTACGGCGCCGGCGATGACCTTGCGTACATGGCGCGCTTCAGCTACGATGACACGTTCGATCTCGAGGACATTGTTGTGTTCGGAGCTCCGGATTCGATCACGGACATTTATGCTGATGAGTAGACCAAGGAATCGGAAGAAGATGGTGGAGCAGGACAGGTTGATGGATCGACGCGTATATACTGTGCGAAATTAAAATAATGGCTGCGCGGATCGCAATTCGCAATGCATAGTTTAGTTTGTATATATGAGTAGTAGCTGTTATCTACTCTGTTGTAAGATTATGTTGT

AAGATTATGTTAAGAGTTTGTTCATTAGCTACTAGGAGCAGAACTGAAGAAGCTTTTGGTTGGCCTTCTACGAGAACAGCGGAACTTGTTACGCCTTGTGTTTTCAGTTGGGCCTTGTTTAGTTCCAGAAGAAAAAAAAATTTCGCTACACTGAAACACTTTCGTTTGTTTGTGGTAGTTATTGTCCAATCATAGACTAACTAGACTTAAAAAATTCGTCTCGTC

>SbWRKY68

TCAGCAGCAGTCAAGCCAAGCAAGTGAGCCCATTGACCTCACACTAGCAGCTAGCTAGCTAGAGGGGTAGCCAAGCCAAAGCCAAAGCCAAAAAGCTTCGTGGCCGGCCGGAGGCGGAGGAGAGAGGAGCTCCTGGTGGCGATGGCGCTACAAGTAGTAGGGAGGGAGGAGGAGCTCCTGGCGCAGCTCCGCGCGCTACTGTTCTTGCCTTCCCCGGCGGCTGCGGCGACACCAGCAGCTCCGGCGGCCGTTAAGGTGGAGTCCGCCGGCGGCGGATCGCTCATGGGCAGTGGCGGCGGTGGACGACGGCGGCGCAGACTGCAGGGGAGCAAGAGAGACCGGGACGACGACAGTAAAGCGAAGGACGAACAGAATCAGGAGGAGAGAGCAGCAGCTACAGAACCTCGCCATTATTCTCCTCCTCCCTGCAAAAGAAGGTGCGGTAGTCCATGCTATATATATGCTTGCAACCAATAGTTCCTCTGATCTCGATCTCGATCACGATGATCCATGGCTGATCTGATCGATGTCGTGCTGCTGCTGGCTGGCTGGCTGCAGGAAGAAGAAGCAGCAGAGCAAGAGCAAGTCTCTTGTGACATCAGTGCCCGATTTCGATGGGTACCAATGGAGGAAGTACGGGCAGAAGCAGATCGAAGGTGCCATGTACCCAAGGTAGTATAGTGTTGTAATAATATAATACTCTATCTATCTTCTGTCCATCTCCTATTTCTGTGATTTAGTTAGTCTGTAAGTACCTTCTTCTTACATATGCAATGAACAATAAATTAAACGGTACTACTACTATTTCTTCCTCCCTTGTACTCGAGTAGCTTGTTCGTAAAAAAAAAAACGATTTTGGGAATAATCTCGATCGAGAATGCCTTGTCCAATCAGAAAAGGAGAGATGCATGGAACGGGAAAAGATGAGAGCTATATATAGCGCTGCCTCTGCTAAAAGATGCCATTTCACTTTCCTATATACAAAATAAATCGAAGAAATACTTTGTCCACCAATCGACAGTGACACTAGCTGGATGCCTGATGCTGCTAGAGTATCAGTTTGGGTCCGTTCATCCACTGCTATAGAGTACGTGCTATGCTAGTGATAGCTAACCAAATAGTATGACTCTGCCCGTCGTGACACACGTTGTGCCTTGTCGCATGCAATTAAGCGTACCACCGACCCATCGATCGAGACAGTTTATATGCTTTCCATACTTTCATTTTTTAGGGAATTATAAGGTCACTGTTACAGTGTTACTGTCTCCAAATTCTTCTCACACAGTTTCTCCTTTTTTTTTTGTATAAAAAGGCAAAACTTTCAGTGCACTGAGTTACTTGGCGCTTCATGCATGCAGAAGTTACTACAGGTGTACACGGAGCGCCGAGCAAGGCTGCGCGGCCAAACGGACGGTGCAGCGCAACGACGACGACGGTGGCGGCGCCGCCGCCGCCCCGGAGTACACGGTGGTGTACGTGTCGGAGCACACCTGCACGGCCAACGACTCGCTGGAGGCGCCGGTCATCCTCGAGACCACCACCACCGTCGTCGCCCCTTCTAATTCTGCTGCTACTGCTAACACCACAACCTACACCGACAGCATCGTCGTTCCAACTATGTCAGATCATGGCTCTTGTTCCACCATCACCATCACCACGGGGACTGAATCTCCGGCGATCTCCGGCGACGACATCACCTG

CTGGAGCAGCACCAGCGGCGGCGCTAGCAGCAGCGATTATAATTACGCCGACGATGACTACTACTACGACTGCGGCGGGTTGTTCGGTGCCGCTGTCCATGGTGGTGGCTGGGCCACCGGACCGGCGGATGCATCGTCGTCGTCGTCGTTGTTGGAGATGGAGGACATGAACGGACCGATCCGGTCGCCGGTGCACGTTCCCGCGGTTGGTTGGACCATTATTGACCCGCTCTTGCTGCAGCTCGTTAATGAGCCTGCTGTCTGCCATTTCTAAGCTCTGCTGCTTTAATTAATTTGTTTCTGTAGCTTAGGTTGGTATTGAGATGGATGTGAACAGTTTTGACTGTACTGTACGTGTTCTAGGCTCTGTTTAATTGAATGGCTGGCCAGGCATTGTCTTCTATATGGCCCTCTCAGTGACAGTGTCTACTCGATTTGATAGATGCCCAGCTTATACGTACGTACTATTATGTATAGAGGCCTTTTACAAGGTCTGTGAGGCACCAAGAGGGGCCTCGTTAATGTCCAGCTTGTAGACACATATTTGCATGTTGGTGGAAAACTGAAAATAGATTATTATTACCCCTCCATTCCAAATTGTTCTAGTAGATACATAGACATAATGTATAACTAGATGTACAACAATGTAACTAGAAATTAAAAGATAGAACGACTTACAATTTGGAACAAAA

>SbWRKY15

CCCTCACAAACCCAGTCACCCACCTCCCCCACTTGCCTCTTCTCCTCTCCCAGCAGCAGCAGCAGCGACGCAGTCCTGCGGCACTGCCTCCAGTTTCGCAGCACCATTCCCATAGGCGGCCATCGCTGTTGATCACCCGGCGGCCCTCCTTCCTGCCATCCAAACCTGCTCTAAGCCTGCGCAACAGCAACGAACGTACAGAAGACGTCGATCGATCGATGGCCGCCAGCGTCGTCGACGGCAATGGAGGCAGCGGAGGGCTGGTGGTGACGGAGCTGGGCCACGTCAAGGAGCTAGCGAGGCAGCTGGAGGCGCAGCTGGGCGGCTCCTCGCCCGACCTCTGCAAGCACCTCGCCTCGCAGATCTCCTCCATCGCCGAGCGCTCCATCAGCCTGCTCATCACCACCTCCTCCGGCCTCGCCGGCGCCCGGAAGCGCTCCGCCGTGCCCTTCGTCAAGGGCACCAAGAAAAGGTATCCATTCCATCGAGGGAGCTTCACGATTAAGAAACAGAAACCCGTCTCTGTTTGCTGAGTGGTTCTATGTGAACTTGAATTGCAGGAAGACCATGGACAAGAAGAGGCATGAGGTGAGGGTGAGCTCGGCGGCCGGCGACCACCCGGCCGACGACGGCCACAGCTGGAGGAAGTACGGCCAGAAGGACATCCTTGGAGCCAAGCACCCAAGGTTGCCATTTGATTTGACACAGTTCCTATCTATCAAAAGATTTTTCTTTTCTTTTTTTTTCTAGACTTCAGAGCCTAATCTCATTTGCACGTGTTCTTATCGCAAGTGGAATCGAAATCATTTCCTCCTGCTTTCTTATCAACTTTTGATGGCGGGCCTCCAGTCTTGTCTCGCTGAAAAAAAAAAGTGCTGCCTCTGTTTGTTTGTGTTGTTTGCAAGTGTTTGGCTGGCCTCCACTTATTCTTTGTGCAAATCGGATTGGTGATGCTACTTTTTTGCAAATCTTGTTCTGGATGCACAAGCAAAACCTCTCCTTTTTTTCCCCCAAAAACAAACAAAGATTCTGGGCCTGAGTCTGACCTGACGTGATCCGTGCAAACATTGCAGGGGATACTACCGCTGCACGCACCGGCACTCTCAGGGATGCGCGGCGACGAAGCAAGTGCAGCGCACCGACGAGGACCCGACCTCCTTTGACGTCGTCTACCTCGGCGACCACACCTGCGTTCAGAGCCAGTGGGCGGCGGCGGCGGGCCAGGCTGCCGCGGACGCGCTGGCGCCGGAGTACAACGGCAAGCCGGGCACCAACCTGACGGTGAAGACCGAGGGGCCGACCGTGGAGCCAGCTGAGCAGCAGGTGCAGGGCTGGGACGCGCCCACGCCCTTCTGCTTCTCCTCCACGCCGGCGACGGCGACGGCGAGCTGGTGCCTCGTGCCGGAGCTCAGCCCCCCTTTCTCCGCGCCGTCCACGTCCAACAACTGGGGCGTCTCCCCGGCGACCTCGGACTCCAACCACGTCGTCTCTTTCCCGCCTTTCGAGGTCGCCGGCGACGACGTGCAGTTCGGCCGGTTCGAAGAAGTCATGTCAGCGATCGACAGAGCCGACGGCGACGGGTTCCTCGACGACCTCGACA

TTGACGTCTCAAGCTTCTTGGTGTGATCTGAGTGTAATGCAACTGATGAACACTAGGAAATGGGAATCTGAGGCAGGCAGCTGGGCGATGTGGCATTCTGGCCTCCCTTCGGTTGCTCTGCCGTTTGTCCAGGGGCAGAGGAGGGAAGAACGCAGCATGTTCCTCGTTCAGTGGCAGTGCCTGTCATAAACCTTAGAATATCAGAGTAGTGAGTGCCCCTTATGTCTTTTTTTCGGTCGAGGGTTGCAAGTAAATAGTAATTTCGTAATAGTGCTGTCAATTTTTAAGCTAAACATGTTAGTTTCTTCTTGTGAATTTAATATCTTCCAACAATTTGCGTGATATTTAATCTGATTAACCCCCCATGAATATTCGATCATGTATAAT

>SbWRKY16

AGCTCTCCCCCAAACGAAGCGTCCAGGAAAGCTGTGTTGTCCGGACCCTTTTGCATAGAGTAGTACGTGTTCCGATCCGTGTTGCCGATCCGACATGGCACAGCAGCGCCCAATCAGGCCCCAGTCGCCACTACGCTCACGCCGGTCAGCAACTTCCTATTCCCTCTCGGCTTCGCCGGCCCGGCCGGGACGCGCTTCGTTTCTCTTTAAAACGGCCCGATCGATCGAGCTCCCCGCTTCGTGTCACTCACTCACTCACCCTCCCGGCCTCCCCTCCTCCCTCGACCACCTCACCGCTGTGCTACCTTGCTCCAGTCCTATCTCGACGGCCGGCCAATATAACAACAGGAAGGGGGTATTCTGATCTCATCGTCGATCTCATGGCGACGACGGAGATGGAGATGGGCATGGGCGTCGTCGTCGGCGGCGGCAGTAACAGTAACAATGGAGGAGGCAGCAGCAGCAGCAGCGGGCTGGTGGTGACGGAGCTGAGCCACATCAAGGAGCTGGTGAGGCAGCTGGAGGTGCACCTCGGCGGCTCCCCGGACCTCTGCAAGCACCTGGCCTCGCAGATCTTCTCCCTCACCGAGCGCTCCATCGGCCTCATCACCTCATCCAACCTCGACGCCGGCGCCGCCCGCCGGAAGCGGTCCGCGAGCGACGCCGCCGGGCTCGCCTCCCCACTCTCCGCGACGCCCACCAGCGACGTCACGGACGGGCCGTTCAAGAACAACACCAAGAAGAGGTGACCACCTCACCAGTCACCACACTAATGCTTTGTTGCTTGCTCGCAAGTTTGACTCTCTGTTGAACTAAACTCGTTGCGTGCTACAATTCGCAGGAAGGTGATGGGACAGCGGAGGGAGAGGGTGAGCTCGGCCGGCGGCGAGAACCCCGTCGACGACGGCCATAGCTGGAGGAAGTATGGCCAGAAGGAGATTCTCGGAGCCAAGCACCCAAGGTACGGTTGGCAGCCCAGCCCCACCACATCTCACTCACTCAGACATGTGTTCATTTGGCTTTTCTTCAATTTCCAGAGCCCAAACAATCTGTTCAGCACGTGTGTATCTCTGTGTGTGTGTGTGTGTTCCTATCCTATCCTAACCTTCCTCCCAAGTGGAATCGAAATCATTTGCGTCTGCCTTTTGGTGTTCGGATGCTAGTGCCCGTCCTGTCTTTTCTGAAAGTTGTGGCCTTTGTTGTGTTCTTGAATAATTGGCCTCCACTTGGCACATAAGGCCATTGGATTTGGATTTGGATTTGGATTGGAACAGTGCGGCGGCTACAATCTTTCCTTGCTGCCGATAATGCTAGTCTTTGCTCCGAGTCCGAATTCACAATCAAAAAATCTCCTTTTTGGGAACTGTGGTGGTGGCTGATCTATCTCCGCCGTGCAAATTCCGCAGGGGCTACTACCGCTGCACGCACCGCCACTCGCAGGGGTGCCCGGCGACGAAGCAGGTGCAGCGCACCGACGAGGACGCCACGCTGTACGACGTCATCTACCACGGCGAGCATACCTGCGTCCACAGGCCAGCGGTGCCGGCGGAGCACAACGCGGACGCGCACGCCCACCTGCAGACCCTGAGCGCCGGCCTCACGGTGAAGACCGAGGGCCTGCCCACGGCCGCCACGCCCTTGTACCTCTCCGCCTCCACGCCCCTGGCGCCGGCGTCGACGGCGTCGGAGAACTGGGGCGT

GGTGTCGCCCGCGACCTCGGACTCCAACCACGTCGCCGCCTCCTACCTGCCGTTCGACGACGCCGAGTGGCGGGGCCACGCCGAGCTCCAGGAGGTGGTGTCCGCGCTCGTGGCCGCCAGCGCGCCGCCGCCGCCGCCGTTGCCGCCCGCCGTGGACAGCCTCGACGACCTCCTCTTCGACATCGACATCGCCAGCTACTTCGCGTGAATCGTGATCCGCGACGTGTTGTAGCTGATCAAGGGTAGTAGGGGATGTGACCGAGCCCTCTGAACACAACAGAGGAGGGAAGAGCACCGGACAGAGCTCTCTGCTTTCCTGGGTCCATGCAGATGCAGGCGTATAGTACCGTAGGAGATGATGAACAAGAGTAGAGATTGTGTCTTTTTTTTACCCCTCTTTTTTTGTTAAATCTTTTTCCTCTTCTGATGAGGCCTTTTTTTTGCTAATCATTTTCCTCTTCTGATGAGGCTTGAGAGTGCTGTGTTTGTATTGTATTATTGAATTTTTTTTTTGTGCCAAGGGAAAATAAGATCCTTCGAGCTATGTGAGAATTTTTC

>SbWRKY66

CGTCAAGCCAAGCCACTTGACGCCGATCGCTCGCCACCTCACGCAGCACAAGCCTCGAGCTGCAACGACCGACCGGACCGGACCGGCCTCCCCTGCTCCGGCTCCCGCCGAAGCCTCCCTCGATCGATCCTCGTCGAAGCAGCAATGGCTGATGCCGTGGAGAGCGGCGGGCGGGCGCTCCTGGTCTCGGAGCTGGGCCGCGTGCAGGACCTGGTGCGCCAGCTCGAGCAGCAGCTGCGCGCGCCCGCGGACGCCGCCTCCGTCGACCTCTGCCGCCGCCTCGTCCACCAGATCGTCGCGCTCACCGACCACTCCATCGGCATGCTCCGCGCGTCCCCGGCGGACCTCGCTCCCTCCCCGCCGCTGTCCGCCACCGGGAGCCCGATCAGCGGCGGCGACGCCACCTCCGACCACCACCACCACCACCCCTTCCGCGCCGCCGGCGCCAGCCCCAAGAAGCGCAAGGCCACGGCGCGCTGGACCAGCCAGCAGGTGCGCGTCAGCGCCGCGGGCGGCGGCGCCGAGGGCCCCGCCGACGACGGCCACAGCTGGCGCAAGTACGGCCAGAAGGACATCCTGGGCGCCAAGCACCCGCGGGCCTACTACCGCTGCACGCACCGCAACTCGCAGAACTGCCCGGCCACCAAGCAGGTGCAGCGCGCCGACGACCACCCCGCGCTCTTCGACGTCGTCTACCACGGCGAGCACACCTGCAGGCCGCCCGCCGCAGCTGGCGGCTCCGGCGGCGCCAAGAGGGCGCAGCAGCAGCAGCACAACCCGCACGCGCAGGCAGCGCTGCAGGGCCTCGCCGCGCGCCTCACGGTGGCCACCACCACGGCCGCCGCCGCCGCCGCCGCCGCCGCGGCGCTCCCGCCAATGACGCCCGAGAGCTGCCCCGTGCGGGGCGCCTCGTCCCCGTGGTCGCCCGTCGGCTCCGACTCCAACGGCTGCCTGCAGCACCAGGGCGTCTCGCCGTGTCCCGTGCCCGGCTACGGGGACTGGGCCCCGGAGGGCGACCTCCAGGAGGTGGTGTCATCGGCGTTCGCCGCCGTCTCGTCGGCTGCGCCCCTGCCGGTGCTCGACGACGAATTCATGTCCCTCGAGTGCTTTGCATTCGACCACAACTTCGACATTGACACCGCAATGCCAAGCCTCTACTATCCATGAGGAGAAGAGCAACTGATCGGCAGCAGCCCTGTCGTCGTCTCCATGACAGGGAGGGAGGAGCAGAGGAGCCTCTCCCTAAGAACTGTGCAGTAGAAGAAGCCAAAACTGTGCAGTGGCAGGAGGCAGCAAAGCTGTTTCATTTTGTTTTATTTTTCCTCTTTCTTTTCCCTTTATTTTTGGGGGTCAGTTCTCAACTTGCTGGGTTGGGTTTTGTTGGTTTCAGTCAGCAAGAGAAAAGTTAAAATTTACTTACGGAGCGATCAGCTCGATCTCAGAAATTATCTCCTCTTGATCTTGATATTGTTTCACTTTCATGTTTGTACACCACCAATACCTTAGAAGCAGAGCACGACAAATCTTAAAAAC

>SbWRKY31

CGGACTAAAAAAAAAAAACATCTCAGCATCTTGGGTGGCCATCAGTCCCATGACCAAAGACGAAGACCGGTCCAATTCAAAACCTTTGAATGTTATCCTAGTGGTACACAAAGGTCCTTTTAAACACCTTCGAATTTGTTTCCGCTTAGTCCAGTCAAAGTTCCTACTCTTCCATTCCACCCGACCCGACGACAACAGCAGCCAAGATACTGCTGCTGCTGCTGCCTCTCTCGTCGTCTCCGATTCCGATCCTCGGTCGGCCGCCGGCTCTCTCTATAACTATAAGAACCCCTCCTCACCCTCCCCGTTGTACGCTCTGTCACACACGCATCATCACCTACACCTCGACGACACACACACGCAGCCGCCAAGACAACTCGTATGAACAGCCAGCACTCAGGTGAGTGAGCGAGCGAGTCAGCTGCCAGCCGCAGGGAGCCGAGTGAGCTAGCTGCGAGCGACTGCGATGGAGGGGGGAGTGCCAGAAGAGAAGTGCGCCCTGGTGGCGGAGCTGGTGCAGGTGCTGGAGATGGCGAGGCAGCTCGAGACGCACATGGCGGTGGTGGTCCAGCAGCAGCAGCAGGGCGGAGGCGGTGCCGGAGGAGGGGCGGACCAGCGGTACCGGGCGCTGGTGAGCACCATGCGCGCCTCCATCGACAGGGCCGTGCACATGGCCGTGTCCTGCTGCGCCGAGGGGCGTCCGGGCACCGGGCAGCTGCCGGAGTCGCCGCCGTCAGGTGGGGATGGCAGCAGCCCGCGCAGCGGCGGGTCGGACCACGCCGGCGAGCTCCGGGGCCGTGGCAATGCGGCCGCCGGCCAGTGCAAGAAGAGGTAGGGAGCTAGTCAAAGCAAGCGCCTACTACTGTCATACTGTGTGCGATTTTTGGTTTGTGATCATATCGAACTCGAACACGATGCAGGAAGACGCTGCCCAAGTGGAGCACCCAGGTGAGGGTGAGCGCCGTGCAGGACGTGAGCCCCCTGGACGACGGCCTCAGCTGGAGGAAGTACGGGCAGAAGGACATCCTCGGCGCCAAGTACCCAAGGTCAGCGCGCGCGCCGGCGCCGGCGCCGCACAACACAACCCTTTTTCACCTCTCACCTGTTCTTGGTCATTTTTTTTTCCAATCGATTGATTGATCCCCAAGGACTCCAACCAATTCTCGCAGATCCTACTTCCGGTGCACGCACAGGCACACGCAGAGCTGCCAGGCGAGCAAGCAGGTGCAGCGCACGGACGGCGACCCGCTGCTCTTCGACGTCGTGTACCACGGCGCCCACACGTGCGCCCAGGGCGCCGCCGCGCACCCCAGCAACCAGCAGCCGGCGGTCCAGGAGCAGACGACCTCCCCGTCCCCGGGGTTCGAAGCAGGGACCGCCGTGCTGCCGTTCTCGCTCCGGCCGGCCTCTAACAAGCCGACGACGGGCGCCGACGCCGCCGCGACGAGCAGCCGTTTCGTCACGACCGGCTGTGTCAGTGTCACCGCGTCTCCTTTCCTGTCGCCGGCGACGCCAGAGAGCCAGCTGGTTAGCAGCAGCAGCAGCGGCTACGCGGTGGGCGGTGGCGGCGGCGTGGCCATGGCCGGCGTCCGGAACGTGCCTGACGTGGAGCTCGCCTCTACGACCAACTCCCCTATGGCCATGGGGGAGATGGATTTCATGTTCCCGCTGGACGCCGCCGATTTCTTGGAGCTGGGGAA

CCCGGCCAGCTATTTCTAGACGACACAAACGTACGTGCCTTCAAAGTTTGCGAAGAAATACCGATAGGTAAATACGACGAGCAAGCTTTGCTTCCTCTCCCACTGTCATGCCTCACTGATGATGTATAATCTCCCCTGGCAAGTTGTAAGTCTGCAAGGGTAGATAGGGCGGCTATATGGCACGTCACACGTCCAGCCTAGCTACGAAGCCTTTCTCCAACAATATATGTATAGTATAGTTGCCTGCTCTCTTGCGTATGGTTCGACTTTACATCTTCTTGTTAGCTACATGCACCGGCCTAATAGAGATTCATTATGAGTATTTCCACAATACACATGGTGTCCCCTTATAAACGTAGTCAAATTTAAAATAGTTTAATTTAGAATAAAGCTATAGTGCTACAGATATGACTTTCACGGCCGGATGCATCACTGATGGATTTTAAGAAATCGGCAGTAGTGATAGCACATCATTGCTAATTTTTTGCTAAAACCAGTGATAGGACCAACCGAGACACTGATATTGGGGTCTTTACTGTCGGTTCATATCACTATTGAATTTAGCCAAGAACTAGCAATTATATTAGGTCATCACTGTCGGGTTGAGCCAATAACTAACATTCCGACACTGATATTATCACTCTCGGTTTGTGGCTTGAACCGACATTAATATATTGCAAAAAAAACTTCATAACTTTCTCATATGATGCCCAATGAAGACGAACTTTATATCAAAGTTGTAGTACTTGACGAGATCTATGACTTTGTATTTAAATTTTTTTGATTTGAGATCATTTGAATATCCAAATATATACCACAATATTTTATAGTTAATATCAAAGGAATACCATATATGTTCTATAGTCATAATTGAGTGTGGAGTGGTAATTAAGGCTCGATGTAGAAGCATCCAAACACAAAAGTTGTAGATCTTAAAAGGCTATACAACTTTGTAGTTTACAACTTTGTTGTTTGAAATCGTTACGATGTCCCTCTATTTGATTCACATGATTTCATATAAGAGTTAACACTAACTAAGGCCATATGTTTTATAACCATTAGTGAGTATGCAGTGATATTTAGAAGAGACCAAAACAAAGTTATTGATACCAAAAAGTTATACAACTTTGTAGTTGACAATATTTTCATTTGAAATTGTGATAAAAATATTTTCATTTGAAATTATTTATCTAACGAAAATCATGTTTGATTTCTAAAATTTAAAATTTAAAATTTTAAAACGACCTTAGATGGAGAAATGCCAAAATAGAAGTTGTAAATCTTAAAAGGTCAATCAACTTTGTAGTTAACAACTATTTCATTTAAAATCATTTATGTAGGGAAAAATACGGTTGAGTTTTCTCAAATTTAAAATTCAAAATTTTCAAATCACCTCGGATAGAGAAATGACCAAAACCAAAGTTTTAGATCTCAACAAGTTATTAAATTTTATAGTTAATAACTTTTTTATTTAAATTCATTTAGGATCCTAAATGCTCATTCCAAAATCAAATGAACATAAAATGACTAGGACTAAAATCTCATTTGGACTCAAGCTTCTTGCAGGTGGAGTAGTTAGGGGACGAACTCCGTCTAGAGTTCGAGCGCCGGTGGCTACAAATCGCACATTTTTCGTGTGAAAAATCTTGTGACTAATGACGCTCCACATGTAGCCCCACAGTGGAGTCTCTCCTGGTGTT

AAGTGGGGTCTCTTCCCATATTAAAAAAAATCCTATTTTTTGGGCCTCTTCATCACTGTCGGTATGCCACTGTCAGTTCAAAATCTAGTAGTGTAGGTTTTGAACCGACAGTGATGTAAAGAATTGGGGTAGTGAATAACATGTTTTTTTAGACATAGGAAGTAATATTTTTCTCAGGGACCTTTCGAAAAGCAGAGAGTTTGCGCAATTCACAACAAAATAATTAAATTAATCCTAGACATCAGAGAAACCACATGAAAAAAAACTCTCACAACGCGGCTAATTAAGGTCTTGTTTAGTTCCAAAATATTTTGGACAAAATACATTTTTTTTGCTATTTTGCATTTTGGAAGTAAACATGCCCAAAATATTTTGGTTCTAAATTTTTTGCAAAATTGAATAGGGACATTGGCTTTGTTTAGTTCTAAAATTTTTTGCAAAATGGACACTGTAGCACTTTCGTTTGTATTTGACAAATATTGTCCAATAATAGAATAATTAGGCTCAAAAGATTGGTCTCGCAAATTACATATAAACTATGCAATTATTTATTTTTTATCTATATTTAATGCTTTATACATGTGCCGCAAGAT

>SbWRKY86

CGTGCTCACACACACTGCTTTGTGCCTGCAGCCTGCCTTTCTTTTCTTGCCTTCTTGGCGAAGTAGCTTAGCTGAGCTGAGCGGAGCTGACGACATGGACGGGTACGGCAGCTACGGCGGCGAGAAGAGCGCGCTGGCCTCCGAGCTGGCGCAGGTGCTGGCCATGGTCCGGGAGCTGGAGGCGCGCATGGACCAGGACCCGCTTCCGGCGGCCGCAAGGGAGCTCTGCGCCGAGCTGGCGTCGTCCGTCGACAGGTCCATCCGCATCGCCCGGTCCTGCTGCGTCGACTCGCCGGCGTCCGGGTCCGGTAGCCCCCGCAGCGACGGCGGCAATGCCGGCGCCGCCCAGTCCAAGAGGAGGCAAGCCGATCGATCCATCTTGTTTCATCAGAGTACTACTGTAACAAACATATAAGCGAGTAGCAAGCAGAGCGTGGCAATAATTCGATCGATCTGTGTGTCTACCGCGCAGGAAGGGGACGCCGTGCGTGAGGAGGCAGCTGCGGGCGGCGTCGGTGCAGGACGCGGCGGCGCTGGACGACGGGCTCAGCTGGAGGAAGTACGGGCAGAAGGACATCCTCGGCGCCAAGTACCCCAGGTCAGCACGCACACGTCACGCCCCCACACGTCACTCCCCTTGCACTGTTGCACCAGACGACTCCTTGACGAACCGACGGCGCAGATCGAGCGACGCTGACACGTGTGGAGCTGTTCCAATTATCTCTCTCTCGCAGGGCCTACTTCCGGTGCACGTACCGCCACTCGCAGGGCTGCCTCGCCACCAAGCACGTGCAGCGCGCCGACGGGGACCCGCTGCTGCACGACGTCGTGTACCACGGCGCGCACACCTGCGCACAGGCCGCGCACCCCAGCGCCCAGCAGCTGCGGCAGGAGCTCCAGCTGCAGCCCGGGCATGGCGCCCAGGAGGACCAGGCCTCCCCGCTCGCGCTGGAGACCGAGGGCCTGAGGGCTGCTCTGCTAGAGCCCATGACGCCCTACTCGTTCGCCACCGTGGCTGGTGCCGGTGCCGGTGCCAGTGCTGGCGCAGACTTCGCCGGCTGGTGCCCGCTCCTCTCGCCGACGGCCTTGGACTGGCAGTTCGAGGAGCTGTTCACCAATGCAATGGAGCCCTTTCAATGGGACCTCTATACGGCCAATTAGATGGGGGTATCTGCAGACAAAACATATATGTGCAGCACGTACTGTAGCTTGTTTTACCGATTTCCGGTATTAGTATTAGCTTCAGAGTAGACTAGAGTGTAGTATTCTTCTGCTACTATGATAACAGCTGGGTACTGTTTTGCCTTGTCCATGCAAACCATGATAACACAGCTTCAAAATGATATGAGCAACCAGACAGCCCAAGAAAATGTTGTACCCAGCTGGCTATCTGAAATTCTGAATCTAAGATAGGATCAAAGATTCAGACCAGGTAATCATGTTGAAGCACTCAGAATCAGAATGTGAATAATTATCCAAATGGAAGTTGTAGTACCATTGCCTATTACACACTGGACACTGCAACTACTCACAACAGCGTAACTGGAGACTGCAACTACACAGAATTGCAGCAGATACATTGTTTTTTTCTCAGCATATGCACAAAGTTGATTTGCCAGATTTCTTTACACCCAAAACATCATTACTAACTAATTGAGCAAAAGAAGAAATGCACGCACTCTTAGTTACTGAATGCACCA

TCAGGGGTACTGATACACCTATCTAGACAGAACTTTTTTCTTTACTGTCTTGAGAAGCTTCAGAAACCCTCCCGTTCGTGTCCTCTTCTTCCTCCAGCTTCCTCAAGGCTCCCCTCAGGCCCAAAACGATAAATAGATTGGTAAGAGTAAGCAGCGATTCCGCGCTCCCGTGCAGCCAGTCAACATTAGACAGCGAGGTTCCGTAATGAACTTT

>SbWRKY58

CATCAGATTCTCAGCCCTGGATTTATCCCTGGCTAACCGAACAATCAAATAGGAGCAAGGGGACAGGGAATGAGTGACAGCTAGGAGATCAACTAATTCCAGGCCTGGATTGATTTCTCTTCTGGATTGGATCCCCAGCTGCCGAACAAGGCCTTAGTCTTCCATGAAGTGGATCTAGTGGGGCGAGACTGGATTTCGGCCCCGCGCGGTCTCGGCCTCCGGTCGCCGCCGCTGTCTCTCGTTCATGCTGGGTTTTCTTGCCGGTACGCATCACCGTCCTCCACAGCTTCTCATATTCCGCGATCAAGTTGTTTTCAGGTTTGAGAGATGCGTCTAGACTCGACTGAGATCGATTTCATTTGTTTCTTGGAACATTTCGATGGATTTAGATTTACCCTGTTTTGTTTGCAACATATCTCTTTTTTATTGTTTTCCTCATCTTTTACGAGCCCAGTTCTTTGTTTTCTTGCTGTGCGCGACCGCGACGGGAAGAACAGCAACTTTGTGTTCCACGGCGCTGGGAAGGAACAATAGACGGACCTTTTTTTTAGTGTCAAGACTCAAGAGTGGAACCGAGTTTGGAGTTCGATTTGGACTCTGACACTCAGCGGTGGACTCCTCCTCAGACCTCAAGCCTCTCAGGGCTGCGATCCATGGAGGGTATGCCAGAAGAGAAGTGCTCCCTGGCCGCCGTAGCCGCGGAGCTGGCGCAGATACATGATATGGCGAAGCAGCTCGTGGAGCAGGTTGCGGATCCGCAGCAGGGAGGGGGAGACGGCGATGCGGCGGCCGGAGGAGGGTACCAGCGGGTCCGGGAGCTGACGAGCACCATATGCGCCAACGTAGACAAGGCCCTGCACATGCTCACGTCCAACAGCTTGGACGGAAGTCCGGCCGCGGGGCAACCGGAATCGACGCCGTCGTCAGGTGGGCATGGCAGCTCGCGCGGCGCCGTGTTGGACTCGGACCAGGCCGGTGGCGGCACCGGCAATGCGCCCGGCCAGGGCAAGGACAGGTAGGGAAGTTGATTATTGTAAGCACGCGCCGCCGTTCACTTGGTTGGCTGTGATTTTTTTCCGTTTGAGAAAACGCAGGAAGACACTGTCCAAATGGAGCACCCAAGTGAGGGTGAGCAACGCGCAGGACGCCACCTACCTCGACGACGGCTTCATCTGGAGGAAGTACGGGCAGAAGGACATCCTCGGCGCCAAGCACCCAAGGTCAGTGCCAGTACGCCACCAGTACGGCCATTTGAGCAGAGCGTCCGCACGGCCGCGATGCAGTGCTTGTGCTTGGCTATTGAATTGATTTCCAAGACGATTCTTGCAGAGGCTACTACCGGTGCACGCACCGGCACATGCAGGGCTGTCTGGCCACCAAGCAGATTCAACGCACAGATGGCGACCCGCTGCTCCTAGACGTCGTGTACATCGGCTCCCACACGTGCACCCAGCCTTGGGGCGCCGCCGCGCACCCCAACATCCAGAGCATGCTGCCGACCACGGAGCAGACGACCACCTCAGGGTCCGAATCAGGGTCCGTGCTTACCAGCGAAATTCCTGGATCGATGGCATCGAGAAAAAGAGACACGGGAGGCGAGACTCGCCTTTCAAAAACCATGGTAATATCAGTTTGTTATTAAGGATGAAACAGTACTGATAATAATATACTCTCTCCGTTCTAAATTATGAGAAGTTTTG

GCATTTTAAGATACATTCATTTACTACGTATCTAGATACAGTGCGTATCTAAGTGCTTAGCAAAAGCTATGTAATCTAGAAAAATCAAAACAGTCTTATAATTTAGAATGGATGGAGTACTTGGTACACTTGTTTGGAACATGTTTCATACTCCATAAGACGTTATGGTGGCAATTTGGTGGCTTACTGTTCGTTTTCTATACCAAACCGAGGGGGTTGAAGAGGATTAGAGAGAATTGATGTGGATTTTAATTTGTAGAGCTCCTCCAACCCCTTGGCTTTGGGGTAGAAAGGAACAGGGCCTAATTGGGGCACCCAGAGAGGCTGTTAGCAAAAGGTTGCCGACTAAATGGCCTAAGATAAGCATATGGTAACTGGTAAGAATATTTAGTGACAGTTAATTTAAATGTTATTACTTCTAAATAGAATATTCCATTTTTCCACAAGTTACTGAATCAACTGCAAACACTGTCAGCACAACTATCAGAATGCAATTTGGTTTGTTATTCTTAAAAGGGAAATCTAAGGTTATACTTGTGCCAAATTTGCAATTCCTGTCTGGGCTCTTCTTAAAAAGGCAACACCTAACTCTGCCTAGGCGCTAGGCAGGGCTACACCGTCTGGATTTTGTCTAGACTTTTCCCATGTAAAGATGTTGATTTGGGCTTGATATATGTATGAAAGGGGTCAAAGTAGAGATGTAGAAAGATGATATATACTCATGAATGTATGGTATTTTTTTATTGTTTTGATTTGCTGAAGTTTCGTTATTTAGTATAGCATTACTCTAACCACCAGCAGAAAGTACAAATGCCTAGGAGCTTGGGGGAGGCCGTCTGCCTGCATAGCGCCTAGTGCTTTTCTTTTTGAACGAAACGACGAAGTTGTGCCAATTTTATTGAATAGAGCAGAAGGCGCCTAGCGCTTTCTGAAACAATGTATCTAGGTCTAGGACACCAAGCCTGTGTTAACCATTCATTTTACTGAATTTGCGCAGATTGAGGAGCCACATTCTACTCCCTATAAGGATGTAATGGCATGGTTTTCTATGGGAAAACTAAGACAAAAAGGACGACTCAGTCATACATGTATGCAACAGGTATTCATATTTTTCAACAAGCAGGTTTATCAACTCATATTTTTCTGCAAGTGGAACTTTCCCCTTTTTTCACTTGCTTTGACATATACTTTCATTGTTTTAGGCTGGTCAGGAACTCCTTGGCAGAGACGATATTAGACAACAGGTTATGGAAAAAATACTGTTGGATAGGAATGGCGTAAACAATTGTACTGTCATTTGCATATATGGTTGGAGTGGTCTCGGCAAGACTTCACTGCTCCATGCCCTTTACAATGATCAACAATTGTTAGACGCCTTTGACAAAAGGATATGGATACAGATATCTGATAAAATAGACATATCAATGTTATTCAGGAAGATTGTTGAGTTTGCCATGAATGAGCATTGCAGCATTACAAACATCGATTTCCTTCGAGAACTGGTTGTGGAGGAAATCACAGATAAGAAATTCTTGCTTTTCTTGGATGATGCAGACATAGTAAACCAACAATTTTGGACTACCTTACTAGAAGTTCTGAACACTGGTGCCAAAGGAAGTGTTGTTGTCATGGCTACAAGGAGCTCTACTGTTGCTGCTGTTAGGAATGTCGCAACACATTCCTATTCCTTAAATCCTTTA

TCTGAAGAAAACAACCTGATGCTTCTTCAACAATATGCTGTTGTGGGTACTGATATCCAGAGCAATCCTGATTTAGCATTGATTGCCAATAGGTTCATTTCTAGGTTTAGATATAATCTACTACACTTGAAGGCCATTGGTGGCCTTCTGTGCCATACAGATACTTTTTCAGTAGAGAAGGATAAGTTTGAAGGAAGTGTTATGCCTTTATGGATTTGCCATGATGTTTTACCAGTCCATCTGAAGAGGTGTCTTGCATTATGTTCCTTGTTCCCAGAAGGTTACATCTTTGGTAAACATCACATGGTTCTCCTGTGGATATCTCATGGTTGTGTTAGGCCAGTTGAAGGGTACGAACTTGAAGACGTTGGAGTTGAATATTTCAATGAGTTGCTGTGTAGATCGTTCTTTCAGTGCTCACCTGTTCACAGTGATAAAAACGAAATGTTTGTGATGCACGAGCTTATGTACAAGGTGGTAGAGTCTGTCTCTCCTGACAAATATTTCAAGTCCGAGGACCCCGTGATCAGCATACCTGAAAATGTTTTTCACTGTTCTCTCATTACCTCACAATTTCAGACTGTTGAACTGATGCACAGAATGAAACAGTTGAAGCATCTGCAGACATTTATGGTGGTGCAACCTGAGTGGAAACCGAACAACATTTCTTTGCCTACATTAAATCTTGTAGGTTTGGATGATTTCTTTCTGAAATTCACATCCTTAGAGACACTGGATCTGAGCCATACTGAAACAGAAGAGCTTCCAGCATCCATTGCTGGCCTAAGAAACCTGCGGTACTTATCTGTCAACAGCACAAACGTCAGGGCTCTTCCATGTGAGCTGTGCAGCCTCAGCAATCTGCAGACACTGGAAGCAAAACACTGCCGCTTCCTCACTGAGCTACCTAGAGACATAAAGATGCTGGTAAAGCTGCGCCATCTTGATCTGACGAAGGAACTGGGCTATGTTGACTTGCCACATGGAATTGGAGAGCTCATCGAACTGCAGACATTGCCAGTCTTCCATGTCAGTGGTGACTCCTCATGTTGCTCCATCAGTGAGCTGGGAAGCTTGCACAATCTGAGGGGCTGCCTTTGGCTTTCCGGACTTGAAAGTGTGAAAACTGGCAGCAAGGCCAAGGAGGCTAACCTGAAGGACAAGCATTGCCTAAACGACTTGACGCTGCAATGGCACGACGATGGCATAGACATCGAAGATGAAGGCGAAGACTCAAAAGATGTGGCCGATGAGCAGGTCCTTGAAGGCCTCAAACCACATGTAAACCTCCAAGTTCTCACCATTAGAGGGTACGAAGGCAGGAGGTTTCCAGCTTGGATGCAGGGTTCTTCTCCATCCTTACCCAACCTGGTGACACTGACACTCGACAACTGCTGCAACTGCACCGAGTTCCCCACCATCGTGCAGCTGCCATCGCTCAAGTCCCTGAGCGTGCGAAAGATGTACGACGTGCAACAGCTAAGCAGCCACACAGACACACATGGCAATGGCAGCACGGCCAAGTTCCCGTCGCTGGAGCTGCTGAACCTGTGGGAGATGTACGGTCTAGAGGAGCTGTTCTCCAAAGAATCTGAAGGGGACTGCCCTCGCCTCCGCAAGGTCTGCATCAGCCGGTGCCCGGACCTGAGGAGGCTGCCCAGCGCTCGTTCTCTGACGGAGCTGGTTCTCCATTGCGGCAA

GCAGCTCCCTGACATCTCGGAGCTCGCGTCGTTGGTGTCACTGAAGATTGAAGGCTTCCACGGTACCAAGTCGTTCGGCTTGCCGGCAGCTGCGGCGCTGAGGAAGCTGGAGATCAGGTCTTGCAAGGAGCTGGCGTCGGTGGACGGGCTGTCGGCGGTGCTGACCACCGTGCAGAGGCTTAAGATAGCAGGGTGCCCCAAGCTCGTCTTGCCAGGAAGGAACCAGTAGCCTGCAGACGACCGGAACATCCACATTTTTGCTAGTGCTTGTTTGTGTTTTCTATATGGATTTATGGTACATGTGACAATTTCTGAGACTGGTCGTTTAGCAAATGAAGTGAAAGAGTTGTTCCAGCTAAAAGAAAAAACATTTTAAATCTTTCAGATATAGGGGTGTTTAGATGCTTTTGGAATG

>SbWRKY91

CTCGGTTTCATTTCCAGTTTCCCCCGGTCAAACGTGCTCAACGTCTAGAACACCACAAGTGCTGCAACCTGCAAGCAGGCCTGCAGTAGTAGATTCATGGCCTTTGAATCTCAGACAACATTCTGCTATAACTGCTACTGCTAAGCATCCTTCCTTCCTTCCTTCCTTCTCCAGAACAAGCATTGCATGCCGGCCACCTTCCTCTCGTCTTCTCCGCTGCTAGATTTCCCTCTGATCCTCCTGCATATATACCTGCCCTACTGCAAGCTTTCGTGGCCATTGTTGTTTGGTTGCCGCCATTCTTGGCCAAGAAACCACCTGTGTTCATATCGCCATTTCAGTCACCTTGTGTTTTCTCATCTGTTTCGTTCTCTGAACTTGTCATGGTCCTCCCGCTGCTTCTCCCTGCCAGCCTCGCCCTTCAAATCGATCACTGCCGTGCCGCGTCGTCGTCGCCGCCGCATCCGACCTGTTCTTCCCTGCCTCCATCACCATCGTCTCATCCTTGCTGCTGAAATACTGTTCATACAGATCACCAACCAGGTCGGCCGGTGTTACATCATCATCCAAGAATCATCACAAAGGCGCCGGTCGATCGAATCAGTGTCTGTTTGCACACTGCAGATCGAGATCGAGGCGGCACAAACAACTAACTGAACTAAGCAAGAGGTAGCCATGCAAGACGGCGCGGGAGCAGAAGCAGCAGGGAGCAGCATGCAGGCGCTGCTGGCGCTCCTCGCCGACGGCGAGGAGCAGGCGCGGCAGCTCGGGGAGATGATGGCCGACGACCCGTGGTCACGGGCGGAGCACTACAGGGGCGCGGCGCGGCGGCTGCAGTGCACGCTCGGGAAGGCGGCGGCCGTCGCCAGGGCCATCGAGGCGGCCGCGCCGGGGTCGTCGCGGGGCACCGACGACCGCTCCGACTCGCCGCGGTCGGCGGACGAGAGCTCTGGCCGGACAACGACGGAGGTGCAGGAGCGCCAGAGCATGTTCAAGAGAAGGTGCGTTGCGTTCACTTTTTTTTTCAAACTCAAAAGAAAAAACGCTGCGTACCAGTACGTGCCGCGCGCCAAAATGAGGTGCCAAACTTCGGCAATGGAAAAGTTTTGGCGGGTGCATGCATGGGTCAAATGGCAAAAAAAAAAAAAAAAAAAAATCCGAAACTGCTCGTGTGCATATTCAGACTCGAATCCGCTTAAAACTATATACATAAAAATTCAGGAAATAATAGAAGAGTAAAATACACTAACGGTCCTTTAACTTGTCGCCATGTGCCACTTTGATCAATGAACTTGCAAACGCGAAAAAGTGCCCCCTGAACTTGACGATCCGGCCCTTTAAATTCTCGTATTTGCATGTTCATAGATCAAAGTGGCACAGATCGACAAGTTCAAGGGACACTTTTTCGCGTTTACAAGTTCATGGACCAAAGTAGCACAGAGCGACAAGTTAAAGGATCGCCAGTGTATTTTACTCATAATAGAACTAGCACGAGACGCGGCCTTGTTTTTGTTTTTTTTAACAAAGCCGTGACTCCTAAGATGTTTCTTTTGTCATATTATGAACTTGAAAATTTTCACGTACAAACGATATCACCTAGATCCTACAAAGGAGTTGTCGGCTTAGCAGAAAAGTTATGGCTGAAAGTACCATTCGCTGATTTATCGTGAGAGAAAATATTGTTAAATAGCTAAAAGATT

GGACAGATATAATATTTTGAACACCTTTATGTATGTGAATTTATACAGTTTCCAACAAATTGCATCATTTTCAGCATATATGCACCCAAAGTTTGGCGACAAACCAAACATTATTATTTACTCGATTGTTGAGTTTAGACCAAATTAGGAGATAACACCTACGTAACAGTGCACAATATTTCGTGGTAACGCCACTGTCAGACAGATGGACCAGCGATACTGACATGTTCCTGTCAAATTGTTGTCTAGTACTTCAGTTGTGTGTGACAGGCTTTATTTGGTTTTTTTTCTTTTAAAAAAGAAACGGAGAGGATTAAGGGCGCGTGCAATATGCAACAAAGCTACTGTACCCTAAGTGATAGAAACATGGGTGTTGTGCCAAAAGCTATCCCATAGAAGCGTTCTACTTATGTACTCCCTCCATTTCAAATATAAGATATTTTGGTTTTTACATAATATACACTTAAATATACACTATATCTAAATATATTATAAAAATAATATATATAAGAAATCAAAATATCTTATAGTTTAGAAGAAAAGTTCTAGTAGATATATTATTGCGTTAGGATTAATATGATCATATACACAATCATGGAAGCAACGGTATGCGTGCAGTGTTCATGTTACTCCGTATCTCAGTCAGGCTGATGTATAGATACGCGTATAGCATACAGAATTATACTTTTTGAAAGAAAAAAAATCACACTGCTCTACTCTGTCATATATATACTCTCTTTGTTCCATTAAAAATATCATTTTCGTCTGTTAGTTTTCCTATTTGACCATTTATTTTATTTAATTTTTGTACAAAATAGTAAAATAAATAAATTATTGCTAAAGTATTTTATTGGAAAAACAAATTATAAAAAATAAATAATATTTATATAAAAGTTTTAAATAAGATGAACGATCAAATAAAAAATCTAAAGTTAAAAACGATATTTTTAATATGAGAGAAAGTAGACAGTAAACCTGAAAAAGATCTTGGAAAGGTACCTAACAGCATGAAGAATTAATTATCATATACTGTCTCCTGTACTACTATAGAGAGTCAGAGTGTAACAAAATGGTAGTAGCATCCTGGTCATAGTCATAGGCTACGGAACTAGTATGGGGTTGTTTGGTCAAAAAAAGGAACATGTTTTCGTGACCATAGGCATCAACAGTTCAACACTCGGTCAAATACAGAGCATTTGCAAGTCAACAAAAAAAATCTAAACATCTGACTGATCCCCCTCGTTTTCCTGGTCATTAATCTACAGGAAAGGTCTACCAAGATGGACCGCGAAATTCCGCGTGCCAGATGCAAGCTTGGATGCCACCCCGGACGACGGCTTCAGCTGGAGAAAGTACGGCCAGAAGGACATCCTCGGCGCCAAGTTTCCCAGGTAACAAACCCTCAACCATCAACATCGAAAACTTGATGATTTTTACAGATTAATATATCGGGTCTCTGTTTTGCTGAATCGCATCTCACGAGAAATTCTGCAGGAATTTCGTAGCGAGAAGCAATTCGGGCGTTCCTTAAAAATTTTGTAAAATTTTTCAGATTTCTCGTCACATCGAATCTTTAGATATATGCATGAAGTATTAAATATAGAAGAAAATAAAAACTAAAAGTGCTACTATTTTTATTTTGCAAATTTTTTTAAAGTAAACAAGGCTTCGATCGATTCATGTGATACAAGTTTCTGATT

CAGGTAGCATATGATGTTTCGTACACTGACATGTACTGCTGCTGCTGCTGTTGCATTGCAGGGGCTACTACCGGTGCACGTACCGCACCGCGCAGGCGTGCGGCGCCACGAAGCAGGTGCAGCGCTCCGACACCGACCTGTGCGTGTTCGACGTCACGTACCAGGGCGAGCACACCTGCCACCAGAAGCAGCGCGCCAGCGCCACCGTGGCCGCGGCGCCCGCGCACGGCGCCGGGAGCCAGTCGCCGCCGCCGCCGCCGCCGCTGGAGCAGCAGCAGCAGCAGCAGGACCCGAGCATGATGCAGCTGCTGAGGTTGGGCTTCAAGCGCGTCCTCAAGGTGGAGACGACGCCGGGGCTCCACGACCATGGAATTGGCCACCGCGACAGCGGCCCCGCCTCCGCGCCCGCCGCGCCCTTCTCCTTCCCCTCCGCGTCGCCGTTCCACCTCGCCGGCGAGGCGACCGACAACCCCGCCGCGGCCTTCTCGCCGCCGCCCGCGTCGAGCTACTTCCCTGCGCCGCACCCGGTGGCGGTCGACGGGAGCTTCTACGACTATGAGGCTAGTCCGGTGGCGCTCATGCGCGGGGCGGAGCCGTCGGAGCTCGGCGAGGTCGTCACCAGGGCGATCACCACCGGCCCCGCCGCGTTCGACTACTCGTCACTCTTCCACCACCAGGCTGAGCTCGACGACCCGCACCTGCCGTTCCCGCCATTTGGCGGCCCGCCCCACGGACCATACCAGTAGGAGATCGATCAGGGCCATGCCATGCTGCAATGGACGCTGAAATCATCGAGGATTTTGCACCTTATTAACACCCTAGGTAGTAGTACCTGTATACCTTATATTTTAGTTACTTGGTCTTCCTGATCCTGAACACTCGCTGTCAGTCACTTTTGTCTTGGTGAAGTAAGTACTCACTCTATTCTAAATTATATAAGATGTTTTGGTACATTAGTTTTAATAGTATGTATCTAGATATAGTGTGTATTAAAATCTATGTATCTAGAGATTAATGTCATCTTGTAATTTGGAATAGAGGGAGTAATAATTTTGAGCATCCATAGCTGTTGTAATGTAAGAGGATTATATTATTGTTGCTTATTATCAGAAGGATATACGAACTATGTTTCTCTTTTGGGTGTACTAGGAACCTTGA

>SbWRKY17

TGCTCCTCTGTCCCCCTTGAGCCCTTCGCCCGGACCTTCCCCTATAAACCACCCACGCCCTTCCATGCGGAACGCATTCGTGCCGATCCGAGTCTCCTGAGTCCATCCTCCTGACCCCCTCGGCCTCGACAGCTGCGGAGAGACAGACGCACACGCCGCGGTCATCGATGCTGCTCATGGACTCGGCGCGCCGCGCCGGCTGCTCCCCGTCCCCGGTCTGCTTGGACCTCAGCGTCGGCCTTTCGCCGTCGTCGCCGGGGAGCAGCGGCCCGGAAACGACAGCTGACACTGACGACAGGCTTGACCGTCCCGCCGCTGGCTGCAGGGTGGCATCGTCCCTGTCTGACGAGCAGGTAACTGGCTGCACTGCAGGGTGGCTTCCTTCTTCCTTTGCTAGCTCCGCGTCGTCCGGCCGGTCGTCCCTGACTCTATCGGTGGTGTGACTGACTGACTGTGACAGGCCAAGACCCTGGAGGCCAAGCTCACCCAGGTCAGCGAGGAGAACCGCCGGCTCACCGAGATGATCGCCTACCTGTACGCCAGCCAGGTCGCGCGGCAGAGCTCCAGCTCCCCCGACACCACCAGCAGGAAGAGGAGCAGGGACAGCCTGGAGCCGCCGTCGAATTCCAGCGACGGCAACGCCAACGCCAAGGCGGAGCCCGGCGACCATGCCGCCGTCGAGAGCGCCCTCAGCGACGAGGGCACGTGCAGGCGGATCAAGGTCACCAGGGTCTGCACCCGGATCGACCCCGCCGACGCCACGCTCACCGTCAAAGACGGCTACCAATGGCGAAAGTACGGCCAGAAGGTGACCCGCGACAACCCGTCCCCGAGAGCCTACTTCCGCTGCGCATACGCTCCCTCCTGCCCCGTCAAGAAGAAGGTACGTACTAGATTTCATTTCACGCATTTTGTTTTCTTCGCGGTGATCGTCGGCGATCAAGAAGAAGGCAGTCTAGTTCTAGTCTGTCGCTGATGACGACAACTCTGTTCAATCCTTGGAATGGAACAGGTGCAGAGGAGCGCGGAGGACAGCTCCTTGCTGGTGGCGACGTACGAGGGCGAGCACAACCACCCGAGCCCGACGCGCGCCGGCGAGCTCCCCAGCTCCGCCTCCGCGACGGCCAGCGGCCCCGTGCCGTGCTCCATCTCCATCAACTCCTCCGGCCCGACCATCACGCTGGACCTCACCAAGAACGGAGGGGGAGGCGGCGTGCGGGTGCTCGACGCCGCCGAGGCGCCCGACCTCAAGAAGCTGTGCCAGGAGATCGCGTCGCCGGATTTCCGGACGGCGCTCGTGGAGCAGATGGCGCGCTCGCTGACCAGCGATTCCAAGTTCACCCACGCGCTGGCTGCCGCGATCCTGCAGCAGCTGCCGGAGTACTAGTGCCTAGCGTCCCCCCTTGTTTTTAGTACTCTAGAGTGTTCGTCTTCAAAGAGCGGTTCTTTCATCATCAGGAAAGGAAAACCTTGGTAGGAACAGTTTTACGTTTACTGAGATTAGAGCCAAATCTCCCTCAAATTTATAGTCGTAATTTGAAGTTTCTTTGTTCTGGTTAGGATCTGAACTGTACTAGCACTGTACATCTGGCATGTCCATAGACCATAGTTCCATCACGAATATAAATAAGGACCACTGTCAAATATCACTACTGCTTTCTTTTATCTGAAGTTGATCCACCAAAGTATCAATGGCCAAA

ATTGCGCTACGATTTTCCCATAAAATGCATCTTCCACGA

>SbWRKY18

CCCCCAACGACTTGACTCCACCGGAAAACTTGTGACGAAGGCGGCGCACGTCAGGAGTGACGCACCGCGGTGACGACGGCGCACGGACCCCCAACTGCCCAAGGCCGGCCGTGGTCGGTCAAGTCGTCTTCGTCTTCGCCCACAAGCTAGGGCACAGGGTAAAATCGGCCGCATTCCTCGTCCCCTATATAGCGTGCCCCGCAACGTCCACGCACAAGGTTCCCGTTCCCTCGCCTTCCCGTTTTCAAGGCCGTACCGTACCCATCTTCCCTAATATAACAGCAAGACGTGAAGGTGATCGATGGCCATGGACAGCACCAACGGCGAGTGCTCGTCCCCCACCGCCAGCGCCGTCGGGCTTCTGCCGCTCTTCGGCTCGTCGCGGCCGCCGCCGGTCAGTCCACTCCACTGAATGATCCATCGCCTGGTCACTCACTGATGCCACTAGTGGCTTGGCTCTATTTTGTTTGCATATATATATATATATATATATATATATATATATATATATATATATATATATATATATATATATATATATATATATATATATATATATATATATATATATATATATATATATATATATATATATATATATATATATATATATATATATATATATATATATATATATATATATATATACCTGCAGGCGCTGTTTGTTGCTCATCTCCTCTGTTTGGTTTGATCGACAGCAGGCAGAGAGTCTGGAGGAGAAGCTGAGGCGGGTGAGCGAGGAGAACCGGAGGCTGGCCCCCGCGCTGGACGCCATACTCTCCGCCGACCGCTCCAACCACCCGCGAGCGCTCGCCACGTCGCCGCCGGCCCAGCAGCAGGGCAATGCGGCTTTGACGACGCAAGCGGCCACCGGCGTCGTCGTCACCGCGGAGCCGCGGCACAAGGTCCGCACGGTGCGCGCGCGCGCCGAGCCCGCGGACGCCGACGCCAACCACCTCAAGGACGGCTACCACTGGCGCAAGTACGGCCAGAAGGTGACGCGCGACAACCCCTACCCGAGAGCCTACTTCCGCTGCGCCTACGCTCCCTCCTGCCCCGTCAAGAAGAAGGTATGTACTATTTGCTGATTTATTACGAGAGACGAACACTTATACAATATAAGTGAATAGAGCCGGATAAATTTTACTGTTTTTTTTAATTTCTTTTAAAAAACGATGCAGGTACAAAGGAGTGCAGATGACAATTTGATGCTGGTGGCGACGTACGAGGGTGAGCACAACCATGAGCAGCATGCCCAGAGCGAATACTCCTACATCAACGACGCATCGACGACGAGCCAGCAGCAGCAGCCCCAGGCCGGCGGCTCGTCGTCGTCGACACTGCCGTGCTCCATCATCTCCATCAATTCGCTGGGCCGGACGATCACCCTTGGCCTGGCCGACCAACGGCGGCCGGGATCGAGTTCGAATGCTGAGGCGGCGGCGGTCGTCGTCGGCGAGATTGTAACGCCTGAGCTTCGAAAGGTTTTGGTGGACGAGCTCGCGAGTTTGCTCAAGAATGATCCCGAGTTCATCGAGTCGCTGGCGACCGCCGTGGCTGATAGGGTGATGGAGAGAATACCAGCAGCAGGGCACATACTCTGATGCAAGACTATATAAATTACGCTCGTGGATGGATGCAAAAATAAATGTGAATACACTTTTCATTTCGCCGTTGGAAAAACACTAATAATATGCCTA

TGTAACATAAACTTGTATACTTGCCATCACAAACTACAAATTGAAACCCACCTGACTAAAACAGTATATCTAATATGCTGTTTTAAAGGGAAGAAACAAAATAGAGTATGTGAAATATATAGTTATATTAAAATAAGGCATTATGAATCTAGGTTTTGATTTTCAAATTTTATACCAATTGCTACGCATTTTTATCCTTAAAGTTAATGAAACTATTGACTTTTCCTCAAAGAATGAAACTCCAAACTTATTTTACCTTTATTAACACTTCTTTTGTATTTTTTAAAATAAGGATTTTAGGTAGTATACTGAACTCTAAAGTACTATGATACTGTAGCAAAGTGCTCATTCAAATCTTATCATGTCGCTCAAGATCAATGGTATGTGAGATGTAAGATATAGAGCAGTGGAGCTAACCATAAGAGGTTATTATATTTAAGCCTGATATCACTACTTATTTCAAAGAAATATCAAATACCATGAGTGTAATAGTTACCTTTGCTTTGCACTGACCTCTTACAATTCACAAGATTTTTTTCTCCAAATTTCTGTAATTTGAATTGGATTATCGGTGTCATTGATATGTGGTAAGGGA

>SbWRKY45

TCCCCTCAAGCATCCCTCCTCCTCTTCTCTCTCTCCCAGTCTTCAGTTCTCTTCTCCGGCCACCGCGCTCTACAGGACCCCGGCGTAGCTTGCTCGGACACCGAGGGGAAAGGAGATTAGTTAGCCAGCAGATGGATCCATGGATCAGCAGCCAACCTTCTCTGAGCCTTGACCTGCACGTCGGCCTGCCGCCGCTCAGCCTCCACCAGGCGCCGGTGGCCGCCGTCGCCTTGGCCCGGCCCAAGGTCCTCGTCGAGGAGAACTTCCTGCCTCCAAAGAAAGAACCAGAGGTATACATATATACATATACTCCTGGACGGCTTTGATGGATTCATTCTTGCTTGACGGAGCCGAGAGTCTAACGGATTGTTTCCTTGATTTTGACGATGATCCACCCAGGTCGCGGCTCTGGAGACGGAGCTACACCGGATGAGCGAGGAGAACCGGCGGCTGACGGAGGCGCTGGCGGCGGTGGCGTCCAAGTACGAGGCGCTGCGGAGCCAGTACACGGAGATGGTGGCCGCCGCCGCCGCGGGCACGAACAACAACCCGTCGTCGACGTCGGAGGGTGGGTCCGTGTCGCCGTCGCGGAAGCGCAAGAGCGAGAGCATGGACACCGCGCCGGCGCCGCCTGCCGCCGCGCAGCAGCAGCAGCAGCACGGCACGCACCTGCACCAGCAGCAGCACCCGGGCCTCGCCGCACCGGACCAGAACGAGTGCACCTCCGGCGAGCCGTGCAAGCGCATCCGGGAGGAGTGCAAGCCCAAGGTGTCGAAGCTGTACGTGCACGCCGACCCCGCCGACCTCAGCCTGGTGGTCAAGGACGGCTACCAGTGGCGCAAGTACGGGCAGAAGGTGACCAAGGACAACCCGTGCCCGAGGGCATACTTCCGCTGCTCCTTCGCCCCGGCATGCCCCGTCAAGAAGAAGGTGCAGCGGAGCGCCGACGACACCTCCATCCTCGTCGCCACGTACGAGGGCGAGCACAACCACGGCCAGCCCCCACCGGCGGCGCCGTCGCAGGCTGCCCACGACGGCTCGGCGGCCCCGGGCGCCACCAAGAACGCCGCTGCCGTCGCGAAGCCGCCGTCGCCGCCGAGGCCGGCCGCACCGGCGCCCGCACCGGCGCCGCACCGCCCGCAGCTGCAGCTGCTGCAGCAAGAGGGTGTCGCGATGAACGTTGAGCAGCCCGTGGCCGCGGCGGCGTCGGAGATGATCCGGCGCAACCTCGCGGAGCAGATGGCGATGACGCTGACAAGGGACCCCAGCTTCAAGGCGGCGCTCGTCACCGCGCTGTCCGGCCGGATCCTCGAGCTGTCGCCTACCAAGGATTGA

>SbWRKY94

CGGAACACTCGCCGTCGCTGTCCGCCCAGCCACCAAGAGCCTTCCCACCAAACTTGCAGCACGCCACTGACGCACATGTGCCTCCTCCTCCTCCTCCGCTGCGTCCTCGCCTATATATTCGTCTTCCTCGAGAGACTGCCCCTCTCATCCATGCACAGGCGCTCTTCAGCTCCTCCCTTCCTTGGCTTCCACTACCAGCCGTGCACGCCTCTGATCTGTTCTTGACGTAGTACGCGATTGAGGGGCTAACGATTCTTGTGGGGACGACGGCGGCGGCGGACATGGACCCGTGGGTCGGGCACCAGCCTTCCCTGAGCCTCGACCTCAACGTCGGCCTGCTTCCTACGGCGAGGCCGGCGGTTCCGGCGAAGTCGACCAAGGTCTTGGTCCAGGAGAACTTCATGGCCGTCAAGAAAGACAACCGAGAGGTACCGTACTCGTTCGATCCATCGCTGGATTTACAAACGTGGGTATGGTACAGGCGTAAGCGTGAGCACTTTGCTGATTGGGGATCGTCGATCGGAACTTGCTGGTCATATTACAGGTCGAGAAGCTCGAGGCGGAGCTCCGGCGCGTCGGCGAGGAGAACAGGCGGCTGAGCGAGATGCTGCGGGCGGTGGTGGCCAAGTACACGGAGCTGAAGGGCCAGGTCGACGACATGGTGGTGGCCACCGCCAACCACACTGGGTCCTCCACGTCGGAGGGCGGCTCCGCGGCCTCGCCGTCCAGGAAGCGGATCCGGAGCGCCGGCGACAACAGCCTCGACACCGCCGCCCAACACCACCACAGACGCAAGCCGTCGCCGCCGTTGGCGGCTGCCGTCGCGGCACACGACCAGACGGAGTGCACGTCGGCCGCCGTCAGCGTCACCGCCGCCGCCTTCCGGCGCGCCGTGCGGGAGGAGTGCCGGCCCAAGGTGTCCCGGCGATACGTCCACGCCGACCCCGCTGACCTCAGCCTCGTAAGTAACGGGATCGGCCGGGATGCCGTAATTAACCATCGCTTTTTTTTTTGCGCGCCGAATTTACGGTCAATTAACTCTCGTGCTGCTTTTTTAAACAAAAACAAACACAGGTGGTGAAGGACGGGTACCAATGGCGGAAGTACGGGCAGAAGGTGACCAAGGACAACCCGTGCCCGCGCGCCTACTACCGGTGCTCCTTCGCGCCGTCGTGCCCGGTGAAGAAGAAGGTGCAGCGCAGCGCGGACGACAGCACCGTCCTGGTGGCCACGTACGAGGGCGAGCACAACCACGGCCAGCCGCCGCAGCACGACGGAGGCAGGGCCGCGAGGTCGACGGCGACGGCGCAGGCGCAGGTGGCGAGCGAGGCCGCCGTGCGACCGGTGGCGGCACCACTGCCGCTGCAGCACCCGCACCAGCAGCAGAAGCAGAAGCAGAAGCAAGAGGCGGCGACGACGGTGCCGTCGTCGGAGGTGGCGAGGAAGAACCTCGCGGAGCACATGGCGGTGACCCTGACCAGGGATCCCGGGTTCAAGGCGGCGCTAGTCAGCGCGCTCTCCGGCCGGATCCTCGAGCTCTCCCCGACCAGGGATTAACTAGCAGTAGACGAACATGATGATGAATTAACGAGTTAGTCCGTAGACAGCGAATTTATTCAGAATCGTAGCTCAGAATTTCGCCACCAAGCGATCGAAGAAGTGAATTCGAGCTGCTTATTGCGTGTGCGTTCGAATATCAA

CTGGCGTGCGGACCATTCAAGTTGCTAATTCAAGCACGTACGTACTTACTCCATTCAAGATTCAAGCTACATGATATGATGCAGCCTGTGGTTAATTAACGGACACGGTATATATAATTATATACATATACCAGTAGTATACCTGACCTCCACGTACGTGTGCATATATGTCACCGGACGCTTGGATTTGGATGCTAATCTGCTAATGCATGGATTATCGAAGAGATCAAGATCGAGAGAGCGACGACCGAGTTTCTGTACGGCACCGGCGCGGGGTACGTAGAAGTAGAAGATCGATGATACTCCGTTCATATCATAAACAAAGTTGTTTTGTACAAGGTTTGAATCA

>SbWRKY22

CGAATCAGATCGGACTTGGGACGATCTCATCCCCGTCCCCCACCCACACACAACCATTCTTGTTGTTGCTTGCATGGACAAGGCCCACCTCGGAGTCGGAGGGGGTCTGTTAGCTCTCGACGCGTCGCCGCGGCCCCTTGGCTTCCTCTCCCCCACGGCGTTCCACAGAGCAAGAACAACAGCCATGGAGGCCGCCGACGACGGAAACGGAACACCCCCGCCAGGCAGCAGGGTCCGTAGGTCCGTCGAGGTGGACTTCTTCTCCGACCAGAAGATCGCTGCCGATGCAGCTAACAACAACACCTGCGGCAGGACCACCGTCTCGCCGGGATCGGGATCAGGAGCCAGCTGCCTCGCCATCAAGAAGGAGGATCTCACCATTAACGTACGTCCTTGTTACACGTACTGCTGCGCTGCCTTGTGTCTTAATTTACTTTCTTTTTTTTTTCATTTCTTCATTGCATTAGAGATCGAGCGAGTACATCTTTTTTTTTCCCTCCCTCTCATTCGATTCGATTCCCCCCTTGTGTCTTTTAATTCCCTGACGACTGACGCCGATGACCGGCCCATGCGCGCATGCAGCTCCTTCCCGGCACCGGCAGCAACGCCAACGACGACGAGGCGGCCACCCGGCTCCGGCTGCTGGACCAAGACAAGCAGAGCAGGAACACCAACGAGGTTGTTGTTGTTGTTGATTGATTGATTGCAAGCCGATCGAGCGTCGTCGATGCATGCCATGTACAGTAGTTAGTACTATATAATAGCGGATCTGTCCGCCGCAAGTCAAGTTAGACTTTCTTTAATTTGCAGATGCAGGCGGAGCTTGCGCGCATGAACGACGAGAACCAGCGGCTCCGCGGGATGCTGACCCAGGTCACCAGCAGCTACCAGGCGCTCCAGATGCATCTCGTGGCGCTCATGCAGGCGCGGGCCGGCGGCCAGGCCCAGCTGATGCTGCCTCCTGTGGCCCAGGCGCTGCCGCCGACGACCGACGGCGCGGCTGCTGCTGTCATGCCATTGCCGAGACAGTTCCTCGGCCTAGGACCGGCTGCTGCTGCCGAGGAGACGTCCAACTCGTCCACGGAGGTGGGAAGCCCGCGGCGGTCGTCGTCCACCGGCGGCAACAGGCGGGCGGAGCGCGGCGACAGCCCGGACGCGTCAACAAGGCAGCAGCAGGTAGCGCAGCAGCAGCAGGAGGCGAGCATGAGGAAGGCGCGCGTCTCGGTGCGCGCGCGATCCGAAGCGCCCATCGTAAGTCGTCTGTACTCTGCTCTTCGGAGTTGTTTTCTTTCTTTCTTTTCTTTCTTTCTTTCTTTCTTTCGGCATGACTAATTGCTTCGACGGCCACGCAGATCGCCGACGGCTGCCAGTGGCGCAAGTACGGGCAGAAGATGGCCAAGGGCAACCCATGCCCGCGAGCCTACTACCGCTGCACCATGGCCAACGGCTGCCCCGTGCGGAAGCAGGTGAGAAGAGGGAGAAGGACCCAGTCAACGGCTGCTGCTCGCATCGCATTTATTTGCGTGTTTTGACCGGACCTCTCGGACCTTGGACCGGGATGCTCCTTTCCTTTGCTTTGTACATGCATGCATGCATGCTGACTTTGACTGACCAGCTGGCACACGCAACGCACGCAGGTTCAGCGCTGCGCCGACGACCGCTCCATCCTCATCACCACCTACGAGGGCACCCACAACCAC

CCGCTCCCGCCCGCCGCCATGGCCATGGCCTCCACCACCTCCGCCGCAGCCTCCATGCTGCTGTCCGGCTCCATGCCCAGCGGGGACATGATGACCTCCAACTTCCTGGCGCGGGCCGTGCTGCCGTGCTCCTCCAGCATGGCCACCATCTCCGCCTCCGCGCCGTTCCCCACCGTCACGCTCGACCTCACACACGGCCCGCCCGCTGCTGCACGACCGCAGCCGCACTTCCAAGTCCCGCTGCCACCGCACCAGCAGGTCCAGCAGCAGCACCACCACCTGCAGGCGGCCGCGCTCTACAACGCCCACCAGTCTTCGTCCAAGTTCTCCGGCCTGCACATGTCATCGTCATCGACATCGGACAATAACAACAATGTTGGCACTAGTAGCAGAGCCGCCGTGGCAGCGGCTGACGCGCCGCCGCACATGGACACCGTCACTGCGGCGGCGGCCGCCATCACCGCGGACCCCAACTTCACGGTGGCGCTGGCGGCGGCCATCACGTCCATCATAGGCGGCGGCGGAGGTCATCCAATTCCAATTGCAATTCATCATGGGCAAGGGCAAGGGCAAGAGCAGGGGCAGCAGCAGGCCCCGACGAGTAACAGCAACGCCAACAACAACAACAATGCGGTGGTGACGAGCAGCAGCAACAACACGGCGACCAGCAACAGCGAGACCCAGTGAACATAACATATATGCGTACATACATATATAGGCATACATACAACCTGTGCAAGGTCGCCTCAGCTCAGCTTTTTAGCTCAAATGATTTCTTAGCTTGCATTTTTTTTCTTCTGTAACTTGTTTCCCAGATCAGTGGCACGCTTTTGTTATTTCTCTGCGGCAAAATGAAGTTGTATTGATTCATTATTTTCCTAAGGCTTTGTTTACTTCCAAAAATTTTTGCAAAATAAGAATAGTAACACTTTCGTTTGTATTTGATAAATATTATCCAATTATGGACTAACTAGGCTCAAAAGATTCGTCTCGTCAATTCCGACTAAACTGTACAAATCTTACGACACATGTATGGAACATTAAATATAAATGAAACAAGGCAAATCTTTTAAGCCTAGTTATTTCATGGTTGAACA

>SbWRKY85

CGCCACGTGTGCGCCCTCCCGCTCCTTCTTATACGTCCCCGTTCCCTCCCCTCCACCGATCTCCCTCGTCTCCGTCCCCGGCCGTCCACCCCCGCTCTCCCTCCTCGCCCGTCGTCGTCGTCGTTACGTCATCATGGAGTCCTCCTACCTGGGAAAGCGCAGGCTGAACGGCGGCGCCGATAGGGAGACGACGACGGCCAGGGCGCCGGCTGCGTCGTCGTTCCTCCCGGCCGCCGCGATGGGGTACGAGTACGGTGACGCGGCGGAGGCGGCGGACCACCACCACCTGCCTCGCCGGGTGGCGGCGGGCGAGATGGACTTCTTCAAGAAGGAGAGGAAGGACGCCGCCGCCGCCGCCGCCTTGGCCGCCTTTGTGCCGTCGTCGTCGGACGAGCACGGCATCAAGGAAGACGACCTCACCATCAACGTACGTTAATAACCCCGCTTTGCTCGCGAATCGACGAATTCAAATTCATTTTGGCGTTTCCCTCCGTCGTCCATCCCATCCGATCGATCCACTAGTCTGTCGGATCGGCGGTTTTCTACTTTTTCTGCTTGACGCGCGTGGCTGTGGCTCGGTTTTTCAGATGGGTCTGCACCACGTCAGCGGGAGGAAGAGCAGCATCAGGAGCGAGGAGTCCAGCGTCGACGACGGCGTCTCCTCCAACGGCGTGGATCACAGGGAGACCAAAGCTGAGGTGAGGGAAAAATATCACCAACGAGCCGTGTGATCCATGCATTTTTGCCTATCTAGCTAGTTTCTACAGTACTTTATTGTGAATATACACACGTACCATATCACTCACTGCCTGTTCTTGGAAATTAAACTTCGATTTCATCTGATGCGCCAATTCTGCATGCAAGTTTAGATGGTGTTTGGGACTGCTCCACGAACTCTGCTTCATAAACTTCGTCGTGGAGCAGCTCCACAAAAAACTGGAGTTCGTGGAGTACCTCTTTAGGTGCTCTCACAACTCCACCCTTTTTTTTCTCGAACTGAGTGCGTAGAACCGTTTGGCTAAAAAACGTAGAGCGGAGCTGAAAAACGTGGAGCAGAGCAGTCCCAAACACCCCCTTAGTCAAACCTTAACTGGCCTCCCATGTAAGGCCTGGTTTAGTTTGCAAAATTTTTGGTTTTTGGCTACTGTAGCACTTTCGTTTTTATTTGACAAACATTGTTCAATCACGGAGTAACTAGGCTCAAAAGATTCATCTCACAAATTACAGGTAAACCGTGTAATTAGTTTTTATTTTCATCTATATTTAATGCTCCATGCATGCGACCAAAGATTTGATGTGACGGGAAATCTTGAAAATTTTTGCGAACTACCCCATGTAAATAGCTACGACTAGTCCACTACCAGACAGACACTGTACATACTAGATAGTAGAGAGAAACAAACAAAAAATGAAAAAAACTTGCTTATTCATTTTGTTTGTTTAGTCTCTCTTGTTATTACAGGATATTAGTATTAAATAATGTGTATTTATATGACCAGCTGGCACTGGCAAAATCCGAGCTTGGGCGCCTGAACGAAGAGAACAAGCAGCTCAAGGACATGCTCAGCAGGATGACCATCAAGTTCAACGCCTTCCAGGTGCAGATGCCGGTCTACACTACACTGATGCAGCAGCAGCAGCAAAGGACCAATAACCATCAAGCCCTTCTCCGCGGAGCTCCAGGCCATGAGGTAAACCAC

TGCCACACCAAAACACATGCATCGATGTAAGCGAGGCAACGTCTCTGACCGATCGAGACTGTTGTGATTGTGACTTGCTGCGATCGTATCATGTTACCAGCTGATGAACGTCGATCCGGAGACGAAGGATCATCAGGAGGGGAGCGGCGGCAGCCACCTGCTCCCACGGCAGTTCATCAGCAGCCTCGGCACCGCCCCCGACGACCCGCTGCGCTCCGTGGGCTCGGACGCAATGCACGGCGGCGGAAACAGCTCGGGGTCGTCCACCAGCAACGCGGAACCGCCGCCGCCGCAACCCTTGGACTACTGCCCTGGCAACGGGCTCATGGTCAGCAGCAAGGAGATGATGCCGCTGCCGGCGTTCGAGCACGGCCACCAGCAGCCGCAGCAGCACCTCGCCCACGAGATGGGTAGCAGCAGCCGGGCGGATGAGCCGCCGCAGCCGCACCACCTGGCGGCGGCGCAGCAGGGCTGGCTCTCCAACAAGGTGCATAAGTTCCTCCCCTCCAAGGGCCCCGAGCCCGTCCCCGAGGCCGCCACCATGCGCAAGGCCCGCGTCTCCGTCCGAGCCCGCTCCGAGGCACCCATGGTAGGCTTGGCTGCCAGTGCTTCTGAATCTGAGTTCTGACTGTGGACTGCTACTGAGTCGTTGTTGCTGAAATGTTTGTTCTTCTTGATGGTATGCAGATCAATGATGGGTGCCAATGGAGGAAGTACGGGCAGAAGATGGCCAAGGGCAACCCGTGCCCCCGCGCATACTACCGCTGCACCATGGCCGCCGGCTGCCCCGTCCGCAAGCAGGTATGCTGTGCTGCTGAACGATGGGTCGATCGAGCCAGAAGTTGGGCACGTTGTTGATCATGCATGTGCGTGATGGCAGGTCCAGAGATGCGCCGAGGACAGGACGGTGGTGATCACGACGTACGAGGGGCACCACAACCACCCGCTTCCCCCGGCGGCGATGCCGATGGCGTCGACGACCGCGGCGGCGGCGTCCATGCTGCTGTCCGGGTCGATGCCGAGCGCGGACGGCGGCAGCCTGATGGCGGGGTCCAACTTCCTGGCGCGCGCCGTGCTGCCGTGCTCCTCCAACGTCGCCACCATCTCGGCGTCGGCGCCGTTCCCCACCGTGACGCTGGACCTCACGCAGCCGCCACCGGGAGCCGCCTCCGCCTCCGCCTCCGCGTTCGCGCAGCCGCCGGCGTCGGCGCCGGCGCAGGCACGGGCGACGGGGACGGAGCCCTCCCAGCTCCAGGCCGCGCTCGCCGACGCCGCGGGCCGGCCGATGCCGCTGACGACGCAGCTGTTCGGGCAGAAGCTGTACGACCCCTCCTCCAAGGCCCCCGCCGCGCAGGCGGACGCGGCGGGCGACACAGTCAGCGCGGCGGCCGTGATCGCGTCCGACCCCAACTTCACCGCGATGCTCGCGGCGGCGATCAAGTCGTACATAGGCAGCAGCGGCAGCGGCAGCAATGGCGCCGGCGGGAGCAGCGGGACGACCGTGCTGCCGCCGGCGGGGGCGAGCAGCGCCGGCGACAGCAGCAGAGACGACAAGGTCGGGGAGCAAGGGAGCTGACGTGATCGCCTAGCGATATGATCGTCCGTTTGCTTGCTTGCTCTTTGCTCGCCCGATTCCAGTTTTGTTCCGCTCTAGTTTGCTTCAGTTTAGTTAATTGTCTCTTGCGTAGTTTAGTTCCCTC

AAAATTTTGCGTAGTTGAGAGTTTGCTTCAGTTTCGTCTGGTAGGAGACGAAAATTTTGCTATTTCTTTTTACTCCCCTGGGGCGATTTTTATGGCAGCAAGTGGAGTACCAATTTTGTTCTCCTACAACTTTTAATTACAGATTCAGCATCTCTAGGAGTCTTTATAAAGCGCACTATCTAAATTATCGTTTAGATAGTCATTTATATAAATTGTTTCTATATATTTTTATTCTCCAACTGTTTTTCTATATCTTGCTTGTACTTTAGAAAGTTATTCTCATTTTTTATCTTTAGCTAACGAGAAATATAAAATATGGGATGACTATGTTTACATAACCAACTGAAGAGTTTGATGGTAGATATTTTTTTTATTAAAATCTCTAGTACTTGGAGTTAGAAATGATTTATAGAGTCTCTTAGAGTTGCTCTCAATCCTGTGGAAGAAAAGTAGATGCTGTCAGCTGTCACCATTTTGTTCTTTCGGGGCAAGCAATGTTAGTTAGCGAGAAAACTGAGATTGCTTGAATCAAAACTGAAACATTGCGTCCCATGTGTAACAGACTTGATGAAGACTGCCGCCATCTCTTTTTTTAACTGTAAAGGAGCTCGGGGGTGTTGGCGGACAATCTCAAAAAAAAAAAAAAGTGTTGGCGGACGCTGCACTCGAGGAGTTGCGTTGCAGCTTAGCAAATTGTAAGTCAGGGAAGGAAGTACACTAAAGATATGGGCAATGCAATCGGATCTTCAACTGAAAATCGTGGTTTGACTGTGGAGATGGTGGACGA

>SbWRKY26

TTGACCCTAATTGACACAAGTAACCATAAAGCCCAAACCTTCTCCGTCCTTTCCATGGAGACGAGGCCGGAGCGCCACCGCCACGACCACGTCCACGAGCAGCAAGCCCAGGAGGGGGAGGAGGACGACGCAGCAGCAGCAGTCATGGAACACGGCGCTGCTCTCTCGCTGCAACGCGGAGCCACCTTGTTTGGGCGCCGGCGTCAGCACGATGAGGAGGCCGACCGCCGCCGCCGGGGCGAGATCCGGGAGGTGGACTTCTTCTCGAGGGACTCCGGAGCCCGGGGCCAGGACGACGGCGGCGGGCGCGGGGTGCCCGGAGGCGGACGCGACGACGTCAACGTGAGTCTCCCGGCCGATCTCGACCATCAGCTGCTGCCGCGGCTTTTGTGCGGATTGTCTGAAATTAAGCGCGCGGTTGTATGATGATCAATTCCCCTTTGTTTTGCCCACCGTTTGAATTTATCGGGTGTAGTGGTTGCTGTGCAGATCGGGCTAGACCTGCTGACCACCGCCACCGCTGCCACGACAAGCGCCGCCGGCGAGGAAATGATGGCGGTCAAGAATCAAAAGGTTTGTTGGTTCATTTTCGTTCATATATATATCTATGCGTGCTTCTTCTCTGAAGATCTCCTCCCGTGTCTTTGGCCATTTCTTGATTGGATCTTGCATTCCTATGTGTGTAGATAGAGGCGTCAGCTGTGGAAGTGGAGCTCAGGCGGGTGGTGGAGGAGAACCGGCGGCTGCGTGGCATGCTTGAGGAGCTCAACCGGAGCTATGGCGCGCTGTACCAACAGCTTCTCCAGGTCACGCAGCACCGACAGCATCCTGCTGATCTCATGATCAACAGATCATCACTGGCTCATGTAAGTGCTATTTTTCATCCGATTATATGGAAGTACTACTTGCATCTGTATCACTATCTCTCCCGTGTATCTTCTTGAGTACGCTAGCTGTCAAAATCATTTTTTTTTCAACCTTATTTCCTTTAATTGTGCCTGGATTATTGTTGATATGATTTCGACGTTGAGCGAGTTTCATATTCCTAATTCAATTTTTCAATCTTATTTCATTTTTATTGCCTGGATCGAATTGTTGATTTCAACGTGGAAAATAGTTTCATATTTATTCCAACAGTACAGCCGATTACTTTCCGTCCAATTCTTGTGCTTTAATCAGGCTTACTCTTCCATAAATCAGGCAACAAGCTAGACTTGTAAGTTTTCTATAGATATAGGTACCAACATTTGAACAATTATTTCGTGTGTGGCCTATTTAATCTATATTTGACTTTGAGAATCAAATCGAAGATCAAGGAAAATAATTTGTTTGTCATCTTAATGTAAGTGCCTTAAGCTTGTCTGAACTTGTGGAATTGTTACTACCACGGCCTTAGACGATCCAGTTGAGTCGGCATCAAAATTGTGCAATTTTTAAATTCGCTTTTTGTCCACGTATATTAGACCTTGCTACAGAAATATTTTTGCTGCCGAGAGATTCTTCAAGATGAAGATTCGTGCAATAATAATGTACAGGCCACAGCTAAATTTGCGTGCATGCACGCACATCCATGTAAGATCCTCGTCAAATCGTGTCACATGAGTTTATGAATGCCTCGGTAGTTACTTAATTTGATTCTTTCATCATATAAAAAAGTATCCTAGCAGATAATATAATCAAGTACTAATATGATAAAAACA

TAGGGGCATGGGAACAGCGGATAGTATAGTCACTCTCAATCTGTACATTACTAATTTCTTCCGAAACTTTCGGAGTGATAGTATCAGACCAATTAATTTCATCCAAACTGAGCACTGAATTAGCTAGCATATTCACCTCAAAACGATTTGATATATATGCACACGGCGTCTAATTTGGATGTTTTTTTTTTGCATACAGTGACACTAATTATGCATACACAGCTACTGGTACATCTTGATCTCTCATGTCTGTAGTCTGTACTAGTAGTAGTTAGCATGTGCTCGTCGATCTCATGCACGAACGCTTTCAGTTGCTTCCTCGCTTTAAATTTCCTCTTTGACTAAACAAATGACACGTACGTGTCCATCACCACCAAATCTGGATGCAGACCCACCTGACGACCACCGCGGCCTCGCACAACACGTCGTCCACTCGGCAGCTGTTGGAGGCGCGCGCGTCCTCTACTGCTATGGCGCAGCCGCATGCCGTCGCCGCTGGAGGGGACGACGAGGCTAGCGACGGAGCCGAGGAGGCGTCGCCTTCGCTGAGCAATGGAGGAAACAACAACGACGACGCCGATGGCAAGAGGAAAACCTCGCCGGATAGGACAGCACCACCGAGAGAGAATGGCGGCGAGCAGGCGTCGTCGGAGCTGCCCGGCCGGAAGGCAAGGGTGTCCGTGCGCGCGCGATCTGAGGCACCAATGGTAACAATTTGATTCCTGGCTATGTTTTTTTTTCACTGTTTAGATATTCCTGATATATATGTTGATCTGCTTAATTGATTATATGAGAAAGAGGTGTTCAGAAACATTTAGAACACAGTATGGGGGTACAGTACGTACGTGCTGTAACAACCACGCGTACCTAATTTGTATAGAGGGAAGAGGCTGCAAAAGTTTTAGCGCTGTTTCTCATTCATTTCTCATATGAACATTGTAATCGTTTTCTTTTTTAGAAATAGGTTTCAGACTGACCAGAGGGCACACAAACTCAAATTATGAAACATTGTGTTCAATTACACATCTACTGACATGCATGTTGGATATTTAGTAACAAGACTTACCATTTGTCCACCTCTCTGCATAGTTATGGTTATTTCATCCAATGCACATAAACTTTAATTTGTCATGATTTAATTTGCTACTGGTACGTGCTCCAATATGTATATGACATTAATTATTTTTTGGTATGCTTGCATTTTGTCTAACTATAGATTAGTGATGGGTGTCAATGGAGGAAGTACGGGCAGAAGATGGCCAAAGGTAACCCATGCCCGAGGGCATACTACCGGTGCACAATGGCTGTGGCATGCCCCGTCAGGAAGCAGGTAATACATGCATAACCTAACCATATATGTCATATCTTGTGATAATATTACATGCATATATGCCATTTCCATACCAATTTCACTCTCATAGGTTTGTTTTGTTGAGAAACTTTAACATATATGTTTGAATTACCGTACCTGCTATATATATACTATCATCTTTCTAATTTGAGTATCTGGTGTTTATATATACATTATCATAGCAACAGTTTGGTATTGTCTCATTTTTTAAAAAAAAAGAACTAATTAAAATGGCATGTAACTTTAACCATTTTAGCTCAAAGAATTGTGAATCATCAGGTCCAACGTAATATGATAACTAGCTTGTATGGTACACAATCATAGCATGATTCTACATGAACCAAAATGC

ATGCTACTGCTAACATCTCTCAAAAACCAATATAATATAAAGAACTATTTTCTGAGTCAAGTAGAGTCCTCTACTTTTGCATATATGCATATAAGTACCTATAATTTGTGACATGTTTATTTCTAACTCGATATGTGAATTGACTGAACAGGAGATCATGCATATGTTGACTTAAGGCCTTGTTTACTTCCACCCTAAAATCTAAATTTTTTTAAAATTTCCCATCACATCGAATCTTTAGACGTATGCATGAGTATTAAATATAGACAAAAATAAAAACTAAGTGCACAGTTTGGTCGGAATTTACGAAACGAATTTTTTGAGCCTAGTTTAGTCCATGGTTGGACAATAATTACCACAAACAAACGAAAATGTTATAGTGTTACTAAACACGGCCTTATTACAGTTATAGCTTTCTCTAAGATATACATACTACTCAATAGGTAACATGAACATGAAGCACTCCATGCTAATGTCAACATACATATACCCTTCGGAATATAAGCTGCACGTACACGTGCATATACTTCCAATCCCAAAGCATATATTATATATTACCGCTTACCTTCTTCCATCTATCTCTAGCTGTATATCAAAGCAGGCCATACACACACGTCTACAAACTGTATAATATGGTCCATTATATTGGGCGACCATCTCTAGCTATAGTTGCAAGCCAACACATTATTTTCAGCGCAGATGAGACGCGGATAGCTCACCGAACAGTAGCTGCGATATTCTGAAAAAGCCAAAGACAGAACTTCACACGCTTGCATGTACTCTATATGACTTACGTGACGGCATGCAATGCATTAAAGCTAGCTCTAGCGCAGCTGGTGGTAATAGTAATACTGGTTTCATGCATGTGTCATGATTAATTGTACATGGATGCATGCAGGTGCAACGGTGCGCCGAGGACAAGACAATTCTGGTCACGACTTACGAGGGACACCACAACCACCCGCTGCCGCCGGCGGCCACCACCATGGCGAACACCACGTCCGCCGCGGCGGCCATGCTGCTCTCCGGCCCGGCCACCAGCCGCGACGGCGCTGCCGCGGCGCTCCTCGGCCACCCGGCGCTGTTCCACCACTCCAGCAGCATCCCCTACGCGTCCACCATGGCCACGCTCTCTGCCTCCGCGCCGTTCCCCACCATCACCCTCGACCTCACCCAGGCGCCCGGCGGCGTTGCCGGGAGCGGCGGCGGCGGCCTGCTGCCTCACGGGCTCGGGCTCCATCGCCCACCCGGCGGGATCCACCCTGTGACGGCGGTCCCGGCGATGCCGTTCCCGGTGCCATCGCCGCTGGCTTCGATGTTCCTTCCACAGCGAGCGCCCACGGGGCCGCCGATGCCCACCGGGCTGCAGGTCGCGCGGCAGCAGCAGTCGGTGATGATGGAGACGGTGACAGCAGCCATCGCGGCCGACCCCAACTTCACCACGGCGCTTGCGGCGGCCATCTCGTCGGTCATGGCAGGCGGAGCAGCGCACCAGGCTCAGCCTACTCCACGTGGGAGTAATATCATCGGCATCGCCGCCGGGGATCAGGCCAACGGCAGTGCGGGTGCTGCTGCCATTGCAGGTCCGACGGCAGCCGGAGCACATGCAGCGTCTGCCGGGTCACCTCGTTTCGCGACGCAGTCCTGCACCACGTCAACTTGAGGTAATTTTAGGAGTGCAGAGTTTTACTGTCAGAGAT

CATCGATCTGCACTACGACTATTTGTAAAACTGATGAGTGTATTATATTTGATATGCACATAAATGAGATATACCGTACCATACTCGTTGTACTATGTACATGTACAGATGAAAATTTGGAATATACAGTAATTTTTGCTGAAATAGAATATTGTATTTTCGAAGGAAA

>SbWRKY76

TCTTCATTCCTTCTTCTCCTCTCTGTAGTAGCTTGCATAGTTGGACATCTCTCTCTCTCCTCTTTTTTTCTCTCTCTAGCTCGTACGGCACTGCAGGTCGTCACAAGCTGTATATAGCCGTCTCTTCGTCTCCATTACCTGGGTTGATACCCTCTCGCTCCAACCCCACCATCCATGGACTTGGTGCCGAAGCAGCAGCAGCAGCAGCGGAGCAAGGAGAAGCAAGAGGAGGAAGAGGAGATGATGATGGCCTTGGCCGAGCACGGGGACAGGCCGCAGGCGGCGGCTTTCGGTCACGGTGGCGGCGGAGGCGGCGGGCGCAGGAGCGAGATCAAGGAGGTGGACTTCTTCTCCACCGCCGGCGGTGCCGCTCGCCGCAGGACCGACGACGACGACGATGGGGATCGAGAGGAGGCCGCCGCAGGAGCACTGGCACGCGGTTGCCATAACACCACGGTCAACGTAAGTCTCCCGTCGAGATCGAGATCGAGATCGAGATTGCGAGCTTGATTGAGATCGATTAATTGATGTCTGTGGCTGTGCAGCCTGTGCTTGCAATTAAGATATATATATAGCCATTATAGTATATACACTTTAATTTACTTGATCATCCTTTGTGCTGTTGTGCGCGCGTGTACGTTCTTTAATCTGTATATACATATATACATGTTTCGTGCCGGCGCAGACTGCACTTGACCTGCTGACCACCAGGGCGGCGGCGGCGGCGGCGACGCCAGCAGCAGTCGACGGCGGCGAGGGTACGGCGAGTGGTCGTGATACAGAGGTACCAATAGTAAGCTTCGTCTGATCCTGTTCATTAATTCTTCCACACGTTCAGAACTTATATTGATCCGGCCTCTTACGAACACACATGCATGTGATCGATGGAGTAGGTGGATGTGGCGGCGACGGCGGCGGTGGAGGGGGAGCTCCGGCAGGCCAGCGAGGAGAACCGGCGGCTGCGGCGGATGCTGGAGGAGCTCACCCGCAGCTACGGCGCACTCTACCATCAGCTCATCCAAGCCCAGGCTCAGCAGCAGCAGCAACAGGTATGATAAACTAGACTAGATAGCTAATTAATTGGGTGCCTAGCTTGATTCTTCGTGTTCAAACTTCAAACTTTGTTGAAAAGAAATGGCTACGCGTCTTAATTGGAGCCTACTACATAGTCAACGTTGTACTTGTCTTGTTATCAAAAAGATATGATTTAAAGATCTCCTGAATATCTCTGTGTTTACTATTATATATAATCGAAGAGAAAAAAAAAACTCCTATCCTACCTCGTGAATGGAGCAGCCAGCAAAGCGAGACTACTACTCATACTTTATTCACTTCATGCACACAAATTTTTAAATTGATCGTTCCTACAAACCCGCTGATAGAATATTACATTTAAATTATTGAGTATATATAAATAGGCTAATTATTAATTCCAATCTGACACCCTGCCTATCTATTTGACTCATATTTTCTTTTCTGGATCCATTTGATTCCTCAAATTAGGTCGGTCTTGTTCTAATATATGTATTCTCCTTTATATATGTGTGTGTAATTAACCAAGAAATGTCATATATAACGATGTTAGACCAATTAATGATTGATGGAATTTTAGAAATTATACATTTTAAAAGCTAGCATTTCCTCAAGTTAGACCCAAATATAATGCATTGCATACCCAGCAGATCCCAAGACATATGTGA

ACAACACAACTATTTTCTCTTCAGTGGTTCAGATGGGCACCTCATTTAATTTCCGTGACCACTTGCTATAGTGTTTACCGTATTGAATTGAAATGCAAGCTCCTTTCTATTGAGATTCTTGTGCTCAACTATCAATTAATAAGCATTAACATCCCCAAGAAAAATTTGGACACCTGATGATTTTAGGTGGCTAATCAAATCTATGTACGTCGATTATCGCCCATGCATTTGCATTGATAATTTGGATTGGAAAGAACTGTCTCCATCCATGTGTGCACCCTCAAAAGCCTATACTATATACAGAGTACATATATAACTGTGCTATAGAACCTAATATATATGTCTTAGAATCAGACTCCATATATCATCGATAGATACGAGTTGTTCTAACTCGTATGATTATGAGTCGTAAGTCTTGAGGAGCAAGTGAATGGTATTTTAAGAAATAGTAAAAGACGGAAGAAAGAATTAGGTAAGTATAGATGTAGTGCATAGATATAGATATAGATGTATATAAGTAGATCTCGCCATCGAAAGCAGCTTGCCGTGCAAACACAAACTAAGCTTGACAGCAAAGCCTCACTTAATTACTAACAGTAGTTAGCATGTTCATTCATGTCAACGTTACATTGGGGGACACATTGCACTGCCCCCATATGCATGCATTTCCGAATTCCGGGTGCTTTCATACGCACCATTTTCTTACTCTTATAACTAACTCTTGTCTACGTGCTGCTGTCAAGCAATTAATTAGCACGAGTAGTTCATGGCAAGTGATACGATGGTCGATGGATCAATCGATGCAGGCCTGCAGTGGCGGCGCAGCGAACCCGATGCTACCGGCGGCAACGACGACGGGAGTACAGTTCATGGACCACGCCGGCCGCGTTGCTCCGGCGATAGCAGGAGAAGCGGCCCCGCCGGCGTTCAGCGGTGACAGGGGCGATTCAGACGATGGCAGCGGAGGAAATGGCGGCGAAGCGGATCAAAACGATGGGATGAAGACGCCTGAGCGCGGCGAGAACGTCGACCGGTCACCGGCGGCGGCGGCGGAGGCGCCTTTGCGGCGGGCAAGAGTGTCCGTGCGCGCACGGTCCGAGGCCCCAATGGTACGTACTAAGATTTGCAGATACAGATTTATGTTGCTGCTGTCAGCAAATAGAATACATGTATATATGAGTGTGGATTGAAAAGACTGAAATTCACATGCACTCTTTCTCAAATTTTGAACGAATCTTTGTTTGGGATATGTTCATATTCATGTGTGTGTAGATCAGCGATGGATGCCAATGGAGGAAGTATGGGCAGAAGATGGCCAAGGGTAACCCATGCCCCAGAGCTTACTACCGCTGCACAATGGCCACAGGATGCCCAGTCAGGAAGCAGGTATATATTGCACTAACAATCTATTATCTATGCATATCGCGGTTGCTACTTCGTAAATACTCTCTACATTTCAAATTATAATTTTTTTTAGCTTTTATAGATATATTGTACATAACATTTCTTACGCGCGCGTTTTATGTTTAGATGCATATTAAAAGTTATGTATATAAAAAAGAAACTCATGTTTTAAATAACGAGCTAAGGCCAAGGTGTTACACCGTAATTAGCGTACTTTAGCGGTAAAATAGTAGCAAAATTGTACATAAACGTAAATGGTGGTATAATTTTTTTCAAGCTATTTCGAACTATAGCGG

CAGAAATAGCGGGCTATTTTTTTCTACGAAAGAAACCATAACAATTTGCAATTTAGAACGAAAGACAGAATCGTTACGATACTGTGCATTCTTCCGCTGGCTTATATTCAAAAGGACTGGTCCATCACATGCTAGGACAGGTTTTTCCTAGCTATTGGTCAAGGAAATGCCAAACAAAAGGCTGCGTGTACTTGATTTGAATTGAAGGGGGACTACTATACTTACTACGACCTGTAGGTATATAATGCAACATGTATGCACTTAGCTAATAAACAAGCAAATGATCGCCATGGTGTATTATTATATGCCGTGGTCCGAATGTCTGATACGTTGCGTGCTCCACAAATTCAAACACATGTGTAGTACTTTCTTTGCATTTCAATATTTGGATATATATAGTCAAATATGGCATTTGAAAACGCACACACATACACCCAACAAACCAGCTGCAAGTACATTTGAATATACCTTTGCAACCCCTGATGACGACATTATCCTATGATAGAGATGACATTTTAATGTGAACATACTATATGCTAGTGTTGCAGCCTGCACGCTTGCATACGTTATTGATTTGAACCTACCTAGCTATATATCTCCCCATCTTTGCCTTACCTGTCCCAATGAACATTTTATAAATAGATCAAACTGTTCACAGATTGTGACATCTCACCAGACGGCTTTAAAATATTTTCCCTTACGTGTTTGTTGCCTTTAACAACTTTATTATTTAATAATGCTGCTAGTTTTTAGAGATCAGATGCCTGCATCTGCATGCATGCAGCATGAAAGAAATGAGCATACGTCATCAGTAACTAATGGGCACACACCGACACACGTGTGCGTGCGATGCATATATATATATATATATGTATATATGTGCGCAGGTACAACGGTGCGCGGAGGACAAGGCGGTGCTGATCACCACGTACGAGGGCACGCACAACCACCAGCTGCCACCGGCGGCGGCCGCGATGGCCAAGACGACCTCCGCCGCGGCGGCCATGCTCCTCTCGGGCCCGGCCGTCAGCCGCGACGTGGGCGCGCTCTTCGCCGGCCACCACGTCGCCGCCCCGGCGCCGCTATTCCAGTACCACCACCCCTACGCGTCCGCCATGGCCGGCGCCACGCTGTCCGCCTCCGCGCCGTTCCCGACCATCACCCTCGACCTCACGCACGCGCCGTCGCCGGGGGCGGCGGCGGCGGCTGCGGCTGCCGCGGGCCTGCTCCAGCAGCGCCAGCTGATGCCGCCGCCTGTCCCCACGATGACGCCGTTCCCGATGTACGGCTTCACTGCTGCCGCTGGGCACAGGCCGGTGCCGCCGCCACAGCCACCGGCGGCGACGACGCTGTTTGGTTTGGACGGCAGCAACCGGTCGGCGCTGGAGACCATGACTGCTGCGATCACTAACGACCCCAACTTCACCACGGTCGTGGCGGCCGCCCTCTCGACGATCATGGCGGGAGGCGCAGAGCCACCGGTTCCCCGGAGCGGCGCCGCTGACGCCGGAGATGGTAGCAACGGTAGCGTTGGCATTGAGCCTGCCACGGCGGCGGCGGCCGGAGCACGTGAGAATGCATTGCATGCACTTTTACAAAGACTTCATGACAGCCGGCAATGACATTATTGGAATCAAGCTAGCTCATGCACCTCTCGATCCATCTGTGGATACATCCATATACACATGCATCGGCTTTGT

AGTTTGTACACGTAATGCATGTATACCATAGAGCCCTCTTATATACGTTGTAGAGTTGTAATTTTGTAAATGGATTATAATTGAGATTTTTATAGAGGCAATTTGATTTTTCACACCTTCTGTACATTCTTTCTGCAGTTAGTTGGGGAAACAGCACTAGCTACACGTTTCACAATCTATTACAGTACTATGCATAAGCGCATAATCCAACCATTATTAATTAGATGTCCTAAAGGAAGAGCGTCCACGTTTCACAATCTATTCCAGTACTATACATAACAATGCTATATACCACCATATAGCACTACGAAATATTAAAGGATTTTAGAAGACATTCATCCCTATTAACAGAGGTGTGCATTCTGTGGATCCGCCTCTGTAAATAGTTAAGCCGGCTCCAAGCAAATGTCCGCCTTTTTTGTTAATAACTTGTAGAGGCATATGACAGGCTCCAAGCAAATGTCCGCCTTTTGTTAATAACTTTTACAGGCATGGTATTC

TAAATTAACTGCTTCTGTTAATTGACTGTTACCAAATTGTTAATTCACTGAGACAACTTCTAATAAGATGCCTGCCTTAAAAATGACTACTAACAGATACCGGCGTTCTAAGTGTCCGCCTCTAGAAATTGATAGAGGCCGCACGCTTAAGAAGGCCTGCCCCTGCAAATAGTTGTATGAAAAAATTCGTAACTTTTTTATCTGAACTCAAATAAAAACAAATTTTATACCAGAAATTGTAGTGCTCGATGTGATATACTCCCTCCATCCATTTCGGAGTCATTTTAGGGTCATGCGCTAAATTACCAATGAAAAAATACCCAAGGAGAAATTAATTCGGCTAGTGAAAGGAGCTGTATGTAGACGGTTGAATTAATTAGACCAATTAGAGGAGCTGCATGTACTTAGAAAAAAGTGAAAATAGTGGTCCCTAGCTTAGATGACTCCGACTCTTGGAGAATTTGTTTCCGATAAGATGACTCCGATAAATGGATGGAGGGAGTACAACTTTGTAGTTGCTAAGTTTTTTATTTGAAACTATTTTGTAGAGTACCAAAAATTTATAACTACAGTTGTAGTTGATCACATGCTCTATTCACCTTAATAGTCAAATGGTAAACAAAGGTTGACTATGTCATTTACGGTCTAAAATGTCCATTAGATGGTTGCCATTTAACCATCTAAAATGGTCCAAATGGAATAGAGTTAAGTGTGATTAAATGGGCTAAATAGTTGCTAATTCATCATTTAGTCAATAGGAGAACTATAACTAACACATTGGCACTCAACTCATTAGCATCACAAATATAAGTTAGAGTGTGATTTAGTTTCCTTTCCAAAATATTTTATTTGCTAGGTTACATATTTTTTTACATACATAAATTTGAATACTTTCTTGTATATTTGAATTTTACATACATAATATGTATATTTAATGGTACTTTACAAGACCATTGAACTCTGTTTAAATGCAGACTAAACAGGTCTGGATGACCGACTATTTATCTTTTGACGTTGAGAATATATTTCATCTCATATGTCTATTGTGCATCTTTTCCCTAGGGCAGAGGATGTTTTCCCCCTAAGGGTTTAGGATAAAGTGTGACCGACAAAATGGACCCAAT

>SbWRKY24

GCTCCGCATAGAGAGGTACGGCCAGGACAAGCCATTGCGTGCCTTGCCGAAAGCTGAGCGGAGCTAGCTAGCTGCTGCTCGATCGAGAAGGGAGGGGTAGGCCGGCCGTTGCGGCTAGCTAGCTAGCTTGGTTGGTTGGTTAAGTTGCCATGGAGGTCGCCGTCGAAAGGCCGCCGCCGGCGCCGCAGGTGAAGGCGGAGGAGAAGAGACCCGACGCCAAGCCAGAGATCGCCGCCCGGCCACCGGTATGAACTGCATGACCTGTTCGATCCTTAATTTAATTTCGCTTTTCTTCAACGCAAAACTAGCTAGAGAGATCTGACGAGACAGAGCATGAACTTTAATTAATTTATTTTGTGGATGGATGCAGATGGTAGGAAGCGCTCTTCCGATAGTCTTCGAAAGCTTTCCATCGACGCAAAGAGACGCGGCCGGCGGCATCAACGTCAAGCAGGAGGAACGCAGGCTGGAGGCGGCCAGGGCGGAGATGGGCGAGGTGAGGGAGGAGAACGAGCGCCTCAAGTCGATGCTGTCCCGCATCGTCAGCCAGTACCAGTCCCTGCAGATGCACTTCCTCGACGTCGTCAAGGTGCAGGAGCAAGCATCGTCTGCAGCCAAGGTGGCCGAGAAGAAGCTCCCCGTCGCGCCGGCGCCGGCGCCGAATCCCGGCACCGACGACGACGGCCCCGACGACCTCGTCTCCCTGAGCCTCGGCACCAGGGCAAATAGCGGTGGCGCACCCCGCCGCAAGGGGCACGAGAGGTCGTCGTCCTCGTCCGGCACCGCTGAGACAACCACCGCCGCCGACGCTGATGACCAAGGCCACCACCAGCTCTCCCTCGGTCTTGGTTTCGCGCGCGGAAACGGGCTGCCGTCGTCCACCACGACGGCCACGGACGACGACAAGGCGAGCCACGCGTCCACGGCGCCCGTCCTGAACCTGACCTCCGACAGCAGCGGCAGTGCCGACGACAACGACGACGCCAAGCCTGCCCTGGCGGCCGCGGGCACAGCCCGTAAGAGCCCGAGCGCCGGTGCCGGTGCCGGAGACAGATCAGCTGACGATGAGGTGCAGCAGCAGGCCAAGAAGGCTAGGGTTTCCGTCAGGGTCAAATGCGACACTCCCACGGTACGTACGTTACAAGTGTGCTACTATGCTTATGAAAATTGTTCCAAATAAACACAAATTAGCATTGTTTGAGCTCTAGCTAAACGCAACTTAGTGTTAGACCTACCCTAACAATTGTTTTGTGTCATGATAATCATCAGATGCCCGATGGCTGCCAATGGCGCAAGTACGGGCAGAAGATCTCCAAGGGGAACCCGTGCCCGCGCGCCTACTACCGCTGCACGGTGGCGGCGCACTGCCCGGTGAGAAAGCAGGTGCAGCGGTGCGCGGAGGACACGTCGATCCTGATCACCACGTACGAGGGCGCGCACAATCACCCGCTAACGCCGGCGGCGACGGCCATGGCGTCCACGACCTCCGCGGCGGTGGCCATGCTCACCTCGGGCTCCACCACCTCCGCCGCCTCGGCCTCGCTCGTCCACGGCCACGGCCACCCGCTGCCGGCCGCCGCCGGGCTGTTCGGCCCCACCACCATGGTCTCCACCGCCGCGTCCTGCCCCACCATCACGCTCGACCTCACCTCCCCCGCCGCGCCGCACTCCCTCATGCACTCCTCGCCCTACGCCGCCGC

CGCAGCAGCAGCAGCAGCAGCGGGGTTCGAGTCCAAGGCGTTCCCGGCGGCGTGGAGCAACGGGTACCTGGCGTACGGCGGCGCCCACCCGTCCTACTACTCCAAGAGCTCGACGTCGCCGGCTCTGGGGCACCTGTTCGGTGGAAGCCTGGGCGTGCCATCGAGACCGGAACAGCTGTACGCCCAGTCGTACCTGCAGAGAGCCAGCAGCCTGGGCGGCGGCCATGGCGCGGTGGCGCCGGCGGCCGTCACGGACACGCTCGCGAAGGCGATCACGTCCGATCCGAGCTTCCAGTCCGCCCTGGCCGCGGCGATCACGTCCGTCATGGGCCGCGGCGGGGCCGCTGCTGCCCAGAAGTGATCAGGAATTCAGGATCCGCGGCGCAGGCATGCACCATGCGGAAGATCGATTGGCAAGGGCAAATCGCCGTTTTTGAGCCCAGCTGCCATGCATGCATGCATGGATTAGTGGTTCATGTGTAATTAATAAGTGCATCGTGGTTATTTTACTTTGTTCATTGGAGTAGTTTTTGGAGTGTTCGTAATTGGCTGGGGAAATGGTTGGTGATCACAATATGTCAATGTAACAGGGATCATCAAATTGATCGATCTATGTACATAGCACAGACGAGTCAAGAATTGTACGTATTTATGCACCATGGATAGAATGAAAGGAAGTTTTGATGGATCAGGTTGTTCG

>SbWRKY90

CTTTTATGCCAAAGTCCAAATAGGAGAGAGAGACAAGAGGTTGTAGCCTGCCTTTATAAGCTATAGACTGCTGCAGGAACAAAGAGATTGGAAGAGAGAGAACTGGAGCTAGAGGGAAGTTAGAGAAGAAAGGAGGGATTAGACCCTTGAAAGTTAGCTAGCTTCTGTCAAAGAGGAGAGAGATGGTTAGAGGGGAGAAGCGTTCTGGTAGCCATGAAGAGCAGTTAACAAGCAAGGTTTGGTATTTTTCTCGTTTTGTTTGTCTGTGATTTTCTCTCTTTCGGATTATTATTGATTTATTGTTCTTGATCTATATATGTAAAGGGACTTCCTTCAGAATTGCAGTTTTCCTGGAATATATAGATATGATTGGAACTAGGATTAGGGAGACATATTCAGCAGCAAATTCTGATGTCGAAATCAATCAGGTGTCCAACTAAATATTAGAAACCACCAATTGTGGTGCATATGTAAATAACACACAAAAAAACATGTGGAAAAACTAAACTGGAATGGTCTCTGCCCCATGTATAGTTGGTCCTCAAGATATTTATCCATGGCTTTCTTTGCTGTTCTTACAAAAACAAATTTCCATGTTACTATTGCAGATCAAAGATGATGATCATATCCTGGATGGCAACTTGTTTAAGTCCTTACACGAATCAAGTTCAAGAAAAGAGGTGCCTAACTATTAGTATAAACATATATATATTCAATAATTTAATCCATTAACCATGTATGAAAGCAATTCGTGCATGGCTAAACATGACTACATGAGAGGATAAAACCAGTCCATTTTCCATCACCTATTTCAGAAGTCTAAGTACCTTATCCAGAAGGTTTTTTTCCCTGGAATTTTCACGAGAAGAGAAACCATAGTTTCTTCATGAGTTCTGTGTGTAGTTTTACCCTGTTCTTATTGTCTACTGCAGTATTCTGATCAGCCGTTTTTTTATGCCTTTACTAGGCAAGCTCCTCAAGCCTAAGGGAAAGATCTGAAACGGACGAAGCATCAAATCAAACTTCAGCTAATCATAACAAGGTCAGGATGCATTCTGATTATATCAATAATCTTCCGAGGTTTATACAAAGACAAAAGTCCATTATTATCATATGTGACTTGGTTAAGGAATGTCTACAGATCTCATAGTTGTGCATTTGTGTCTTCAGGTTGATAAAGATAAACTTGCATCCACAAGGGCAGAGATGGGTGAGGTAAGAGAAGAGAACAAGAGGTTAAAAACTATGTTGTCACGCATTGTAGAGGACTACCGATCTCTTCAATTGCACTTCCATGACGTTCTTCAAAAAGGACAAGCCAAGAAGCTTGCTGACCCCTCGACCATCATGCCCACCGGCATCGAGGAGCCTGAATTCGTCTCACTGAGCCTCGGCACGACCACAAGCATGCACAGGAAGGAAGACAAGAATAGTGCTGCTGAAGGAAAAGGGAGAGAAGACTTCATGAGTATTAAAGAAGAAGGCCTGTCACTTGGACTGTCAGCCTGCAAAGATGGTGCAACTAATAACAATGTAAAGATCCAGCCCGAAGTGATGACCTTGAGCCCTGAAGTTAGTTCCGAGGATGCCAAGGATGATGCCATGGAGGCAGCAGATCAGCAGTGGCCGCCAAGCAAAGCAGAGAAGAGCTTGAGGAACGTTGGCACGGGGCCTGAGGATGACATAGGTCCACTGCCACA

GGCCAAGAAGGCAAGGGTGTCTGTAAGAGCAAGGTGTGATGCACCAACGGTACGTAGACATATATATTCCATCTGAGAGAGATGTATAATGCCTGCTTGATATGTATAGGAAGTAGCTAATAATACACATGGTGATCAGATATACAACTGAACAACATGCAAATATATGCTTTGGTTGTTGATCAGATGAATGATGGATGCCAATGGAGAAAGTATGGGCAGAAGATAGCCAAGGGGAACCCATGCCCCCGTGCCTACTACCGGTGTACAGTGGGAGCAGGATGCCCCGTCAGAAAACAGGTACATAATGCATGGTTTACATCAAACAATACTGTTCGATTCTCATCTTTGAATCTACACATGTTCAGTTCAAAGGTAAAATGCTTCTGTCTCTGAACATAGAAAACCCAAACACACTAAACCCAAGTCTCCTTTACACGGCTATAATTTATTTATTCTTCATCAGTTGAAAACAGAAGGGACTTTATTTAAGATGCACATAATATAGTTGCTATAGATTGACTTGGGGAAGTTGGGTTGGATAACCGAGTACTGAATCTAGGCCCCGAACCTAATTAACAAAGCTGCTGGCTGCACTATAGAAGTACTAGAGAACGTGGGTTTGGAGTCAATGGAATGGATGGCCCGAAGTCTGTTAGTTTGAACAATCATTGCTTGCAGTCTACGAGGCAATTGAAATCGCAAGTGATGGGTTGACAACTTGACATGGACCCATGTGTAAACTAATAAACAAATGCTATCACTTACTATTAGAGCTCTGAAATCCGAATGATGTGATCCAACCGCTTCCGAACCCCAGTTTACTCTATGCATTCTACTAATCTACCAATACCACTACTAACAGTTGGCAATAGACGAGCTAAGTTGAACACTAGGTCTTAATTCACCCATAAAATTGATCACAATTCAGAACCACCACGCCAAACAGAATTCAACAAAAAACACGCATCTAATCGAACTCGTGAACTAAGGAATCGTACATTCGCACTGGCGCTCATCACGGATCGAAATTAAGGTCAGAAGCACGAATGCACGATAGATTGGAGATCGGGGACTCGGGGGCTTACCAGATCGAGAAGATACGGGGAGGAGGCCGCGAGCGCGGCGAGGGAGCGCTAGGGAAGGCGACGCGCAGCCCGAGGCGTCCGCTTCCCACTCCTCTCCGTCGCGGCGGAGGCGGAACCCCGAGCCCCCGAGGAGGAGTGCTCCCGCCGCCCGCGCATGGGGCGCTCCCGGCGTCGCCGGGCTTCCGGTTCGCGCGCTTGCTTACTGGACCTGGTGGACTGGTGGTCCCTCCCATGCGCCCATGGCGAATTGGCGACGGCCAGTTGAATTTGAAGAGAAATGGGCCTTGGGAAACTAGTGTTCGGAGAGAGCTGGACTGGTTCGCAGCATATTCAGGCTAGAGAGGCCCGGTTCCATAGTACTAGTATCTTAAAAAAAAAGGCGAAGTCCATTTCTTCTCGGCCTCGGCCCCAACTACTTAGCTATATACTCCCTTCATCCCAAAATTGTAAGTCACTGTAAGAATCTTAGAGAGTCAAAACAGTTTGACCAAATCTATATGATAGGATAATAATATTTATAATATTAGCTAAGTATCATTAGGTTCTTTGTTACTTATATTTTCATAGTATATCTATTTGATGTCACAAAAATTTGTAAGTTTTTCTATAATT

TTGGTCAAACTTGAGATGCTTTGACTCTCCAAGATTCTTAGAATGACTTACAATTTGGGGTGGATGGAGTACTTCTCGATACGATTTTCAAATTTAACTCTCTATATAAAAAGAATATCTCTACCATATTGAGGATAAAGGTCTTATTTGGCACGATTCCTCTTCAAGTGAAGTTAGAGACACAAATTGTTGAAATAAGCCCTAAGTGATCCCTTTCCCTATAACAAGTGTTGAAAGAGGATCAAATGCAAAAGGCAAGCTCTAGCAAATCGCCTTCCCTATTTTTTATCAATTGAGAAAACCACCCCCTTAGTCCCTCAAAGAAAGGGGTAGCGATTCAATCCCCTTTCCTTTCATTCTCTACATGTGCCACCGACCGAGCTTGCCATGCCACAAGCAGGGCGCACCACCTCTCTAAGTCAAGGTTACTACGAGCAAACCACACCACTGGCCCGAGATGAAAGAGAAGGAAAGTAGTTGGTCGACTTGTCTTCCACTATCGCTGCCTTAGCTTCTTCTCATCTCCTTCATGGTGATCCGCGTTTTCTAACCTCACATGCCCTCTCTCTCTCTCTCTCTCTCTCTCTCTCTCTCTCTCTCTCTCTCTCTCTCTCTCTCTCTCTCTCTCTCTCTCTCGGCAGCGATGGGGGATGAGGTGGTGGGAGCGGCTGGTAGTGGCGATGGGAGGTGTCGGTGGTGGAATGTTATAAGATGGATGTTGTTATATCACACATTTAGGTTCAAGGTTTTTTCTCTTATACGAATTTGAATTTTGCTTCTCTCCACACCTCATCGTGAGAGCTTCTTTTCACCTCTGGTAAGGTTAGAGTTTGGGAGTACTTATGTGCTCCCACAGGCCTCGAGGGAAAAGTGGTGGAGGTGTCATGGAAGGGTGGGGGCACGAGGTTGATGAGCCTGGTTGGATGGGCTGGAGGTGAGAGGCCACTACAACTCAAAAATTTAGCTAGTGAGTGGATATAACAAGAGACGTTGAAAAAGATGGTAGTCATCCATTGGCATAGATATTTAAGAAAGAAGGATGAACTGAAGAGAAATGTTGGATCTAATGTCACATATTTAAAATTTGGTGTAGGGGCCTTTAAATGCTTGAGTGTCACCGACCTAAGAGCATCTCCAAGAGCTTGGCTAAAATTAGTAGCCAAATTCTAATGTTTAGCCATTTTGTAAAATAGACAACTTCTTAAAAAAGAAGAGGTCCACAACAGCCTTGCTATCAGATAGCTAGTGTCAGTGGTTGGCTATATATGGCCAACGAAAATCAAGAAATAACAAACTTTCTATTTTACAAAACCAAATAGCACATCTATTGGAGCCTATTTTTCTACTATACAAGTTCTAAAAAGCCTTCATAATAAGATAGCAAAGCTGTTGGAGTTGCTCTAACCTCTATAAAAATATACGTAGTAGATCTTTTCATGATTGTTTTCATGGTTCCAAGCATCTAGGATGTGTTTACTTCTAACCTAGGCAAAAGTGCTTGGGTCAGTCTACTTCCCAGGCCATCGATTGACTGACGGCTAGATCAACCTGTCTGGGTAAGCACCTTTAGGCCACGCTACGAACCAAACACGCCCCTAATGTATTGGATCTTTGAGAGTTAAAATCAGAAATAGGAAAGATAACCACAAAGAACCCTGAACATTATTTTCTATATAAGCCATAACACAAATTTTGAATTT

AAAGCTCAACTTGTATAGTCACAAAAAAAAAATCCCTAGTGAATTGGCATAGTTCTTGTGGATCCAATAAGTGCATGTGCACATGGTATTCGATCAGGACCCGGGAGATTGATATGCTGCTTTTATTTTGAATTTGTTTATATTATACTATCAGTAGTAGGAAACTAGCCAAATGCTGCTTGGTTGTGGACCCACTAATTTCCTGCAAGCAAGTCACCATGAATTTTCATGCATGAGCATGACACACCGTGCGTGTAACATTTGCAGGTGCAGAGGTGCGCGGACGACATGTCGATCCTGATCACCACGTACGAGGGCACGCACAACCACCCGCTCTCCGCCTCCGCCACCGCCATGGCCACCACCACCTCCGCCGCGGCGTCCATGCTCACCTCGGGCTCCTCTACCTCCCTCCGCTTCCCCGCCGCCTCGCCGGCCGCCGCCGGCCTCAGCTTCGGCTTCCCTCCGGCGGCGGCGCACGACCCCTCCAAACATTTCTTCCTCCCGAACGGCGGCGCCGCGTCCATCACCTCCACGCCGTCCTACCCGACCATCACGCTCGACCTCACCTCGCCGGCGGCCACCTCGCAGGCATTCTCTCTGGGCAACAGGTTCTCGTCGAGCTTGGTTCACGGCGGCGCTAGGTACCATCATCCCACGAGCCTCTCCTTCTCCAACTCCGGGCCCAGCGCGCTGTCCGGCGCTGCATGGCCGGCGGCCGGTGGTGCTGGGTACCTGAGCTACGGGTCACCAGCAGCCTCGTTGTTCAACGGTGGCGCTGCACTGAGCAGCATCAACGGAAGGCAACAAGGCGGGGAATTCCCCGTGCTCTACCAGCCGCAGCAGAAGGCGTCGGCGGCGGCGAGCGGGAGCGCACCGGAGGGCGTGCTCACCGACACGATAGCGAAGGTGATCACGTCGGACCCGAGCTTCCAAACGGTGCTGGCAGCCGCCATCACGTCGTACGTCGGCACGCAGGGAGGTAACAGATCGTCGGCGGGAGGAGAGGGCGGGAGCCAGCTGCAGGGGCTCAAGTGGGGACAGCACCTCGGCCTGGGGCCGTCGCCATCAAGCCCGGGTGCGGCGTGCTCGTCGGCGCTGCTGGCACGGTCATCGTCGACGACGGCAGCAGCGGTGGTGGAGCAGGGTTCCAATGGGCACCGGTCGTTCTTGCAACCATCGCTGGGCTTGTCAGGTTCTCACAGCACCTCCACCTCTCCTGTGGAGAACAGGGAGCACTGATCGTAAAGATTCTGAATGTATTTAGGCCACTTCATTCAAATGTTTTTTTTCCCTTAATCTGGTTATTTCAGCAGCATTTGGTGTATATATCCTGAGGATAAGTGTCACTTTATAGAGGATAGATATCCTGACTCTCACTGTAAGGTAAAGAAATTGTTTTGATTCACACAACACACATGTAAAACAAAGGATTGAATTTATCTTTGTTCAGTCTTTCCTTCCCTGATGAGTTAGCAAAATGTGAGCTGATGATGATACATCAACACAGTATTGAATTATAATCAAATAGCTGCCTTTTCCAGAATGAGGATCTATCTACAGATTTTTTTTTCCTAAGTTACATAAATAAGATCTCACGGTGTATAGGATCACCAAATGACTCCAGGAATTCACGGTATGGTAAGTGTACACATTTGGTGGCTTGTGCTGGAATGCCAAACTATACCGTTCCGAACAGT

AGTACCATGTTGTTACAGACTAAGAGCAGCATGGATGCACCAAAATAATCTTCTTGTGTAACTCAAGCATCATGATGATTGTTGACGGCTTCAAGTGTGAACAATGTTAGCTTTAACTTTCACTTAGAGATGACCCTCATAAATCGACTCACAGAACTAAACACTCCCATGACATTGTTCATCAAGGATGGTGCCTCTTCAGATTGCTCATCGACACCACCAAATATTC

>SbWRKY28

TGCAGGCTGCATATGCACAAAAGAGCAAAGCACAAGAGAAGTTACAAAGTGCTAGCTAGCTATATATATATATAGCTGTTCATGGAGCTCAAGACTTTTGTTTTCAAGGAGCTGATGAGAGGCGCAGCTGACAAGGTACTTAAAACTGCCAATATACCCATGTTTTCTCATTTTCTCTCTACAAACTTCACGTAGGAGTTGAGCTTTGTTCATGTTACTGAAAAATATATATGTGCATGTGTCCAGATGAAGTTGGGCGACGAACCGGCCGCCGGGCCATTTCTGTCACTTAGCCTCGGGCCGGCGAACGCGATCGGCGCAAGTCTCCGCACGAGAGGAGGGGAGGCGATCAATGCATCGCAGGCGCCACCGCACGCCGCCAGCAACGCCGACGACGGGATCGGTTTGGCCCTCGGGTTGCGCTGCGACAGCGATGGCGGCGGCGAGCCGGTCCTCGCCGCCGTCGTCGGCTCTGCTGCTGGCACCAAGCGGCAGAGGGTGGCAATACTAAGCGACGACAGCGGCGGCAATAGAGGAAGCAATAAGGCGCTGCGACTGCCGGCGCTGCTGGCACCACCGCAGCAGCAGAGGCCGGCGGGCCGCGTCACCTTCAGGGCGCGGTGCAGCGCGGCCACGGTGAACGACGGGTGCCAGTGGCGGAAGTACGGCCAGAAGGTGGCCAAGGGCAACCCGTGCCCGCGCGCCTACTACCGCTGCACGGGCGCGCCCGACTGCCCCGTCAGGAAGAAGGTGCAGCGGTGCGCGCACGACGCGGCCGTGCTGGTCACCACGTACGACGGCGCCCACAACCACCCGCTCTCGCCCTACGCCGCCGCCATGGCATCCGCCATGCTCGCCTCGTCGTCGTCCTCCTCATCATCTGCTACCGCCGCCAGCTGCGACGACGACGCGCCGCGGCTGGCCTTCCCCATCTCCGTGCTGCCACCGGCGCCGCAACGCTATTCGTGCAGCCGCGACGTGGCCATAGGCGCCGGTCCGCCGCCGGCTGCGCCTGCGGCGACGAGTCACGGCGACCTCGTCCCGATGGCTAACATCATGCAGAAGGCGGTCGGGGACCCCAACTTCCGCGCGGCGGTGATGGCCGCCGTCGCCAGCTACGTCGGTGAGCAGTGCGGTGGAAGAAATATCTCGACGACATCTTCACCTTGCCACCTCCCTGCTAATAACTGATGCATGGGCTCACAGATACTGCGTGAGGATAGGGAACAAAAAAATTGACTTGTACATTTATCCCATTCGATTTAATTTGTTCTTTGATTACCAGTAACCTACTTCAGGTTGTAACAACATTATTTTCATTTGCAGTTCTGAATTTGTCTTGTAATTAATAACAATTACTTTTCCGATGGAAATTTTCATGTTGGTATGAATTATATATAACATGAATCTCTTTTGGCATTAATTTATGTGATACTAATAAATTTGTCATCTACCTCAGTGAATTATGTTTTAGATTCATGAAATTTGTTAGGTTCTTCGT

>SbWRKY50

TAGACACCCGGACATTGACTTAGATGCCTCGGGAGAAGCAGCATTGGGATTTAAGACCCTTCCTCCTTTTATTAGCTGTACAAACGGCAGAGAGACAAAGGTACACAAGCTAACTAGATAGAGGTCAAGAGCATGTCATCCAAGAAGAAGAGGGCCGCGATAGATCTCTCCCTAGAGGTAGAGAGGAGTGACGAAGACCATGGCAGCGGCGGCAGAGGCAAAGGCGATCGCCGCCGGGGCAAGGATGATGGTGAGGTGGACAAGAAAGAAGAACAGTTCAAGGAGCAAGGCGAGGAACCCAAAGAGGAAACCGGCGAGGAGGAGAAGGTGGTAGTTGAAGTGGTTGTAGACCAAGGAGGAGACGGCACCAAGGAGATCAAATATAGGACTCAACAAGGAGAGGAGATGGAGGACGACAAGCAATCGCCAGCGGATGCCCACGGCGACGGGGAGAGCGATGGGGCCGAAGCTCGTGCGCAGGACAAGCACGTGGTAGAGGCCGCCGGCAACGGCGACGGCGGGGACGATAGCTACACCACCATGGTGCAAGACGAGGTAAGCACATACACTAGCTCGAGGCTGCTGCTAGCTAGATGTACTATATATAGTGTTGCTAATTGAGCTTTGCAGCTAATAAGTAGCTAGTGTTAATTCATCTTGTTTAATTCCTCCAGCAATATAATGACATATGTGTGTGTAATATGTCTTCAGGTAAGTGCGATGCAGGAGGAGATGGAGAAGATGAAGGAGGAGAACCGGATGCTCCGGCGAGTCGTCGACCGAACCGTGCGTGACTACTACGAGCTGCAGAAGAAGGTAGAAGCCTGCTATCAGCAGCAACAGGCAGATGAGCCTAAGGTAATAGAGGAGTAGCTAATAATCCTACGCTAATCGATTAATCTTATCTAGCTAGCTAGGGTGTTATCCAATTGTAGCCTACGATTCTACCCCATGTTCCATCATCTAGCTAGCGCTGATCTCGTGCCGCTTTTAAAATTCTTCCACGTGACACCATAATTACCATGTCAATTTGATTCGTGGTTTTATTTGATTTCTAATTCGATGCGCGCGCAGGAGCCCGAGGTGTTCCTTTCCCTCGGTGCCACCGCCGCCGGGACTGGCGGCGCCTTCCCGGAGCCGAAGCGGAAGGAGCGGCAAGCAGCGCGGCGGCCGTCTGTGGGGAGCGATGACACCGACGACGACGACGCCAAGGAGGACCTTGGGCTGTCCCTTAGCCTGAGAGCGTCGTCGTACGAGGAAGAGAAGCTAGAAGCGGGGCACGACGACGTGGAAGGTGCCTCCGTGGTCGGCGCCGACGACGGCAAGGCGAAGGGCTACACGCTGCTGGAGAGTAGCAAGCTCGGGGCACCGGCGGCCGGGATCACGAGCCAGAGTGTCAACCCGGCCAACCGCAAAACTAGGGTTTCCGTGCGCGTCCGATGCCAAGGCCCCACTGTAAGACGCATCTCACTGTTCGAGTTCTTCCAGCGCATGATTTCACTGTCAAATTGATGATGACGAGTGATCTGGTTCGCGTGCATGCATGCAGATGAACGACGGGTGCCAGTGGAGAAAGTACGGGCAGAAGGTCGCCAAGGGCAACCCGTGCCCGAGAGCCTACTACCGGTGCACCGTCGCGCCGGGCTGCCCGGTGCGCAAGCAGGTAACCAAACATTTGCCTCGCCACTTCAGCGCATGCA

TTGCAGTCTCTGCCACTAAATTTCGTCGCACCGGCCGGCTGCGCTTGGCCGGAATTATATATGTGTGTGCATGCATCGGTGCATGGAGTGATGGTTACTGATTCGTGATGGTGTGCGCGCTCGATTTGCAGGTGCAACGATGCCTAGAGGACATGTCGATCCTGGTGACGACGTACGAGGGTACGCACAACCACCCGCTCCCCGTCGGCGCCACCGCCATGGCCTCCACCACCTCCGCCGCGGCCACATTCATGCTGCTCTCCAGCACGACCTCTTCCTCCTCCATCTCCGAGGCCGGCGGCGGCTCAGCGGCGCCTCCCTACCTCAGCACTCCATACCTGCTCAACTCCACCTCCCACCACTCCGCCGCTTCGCCGCTGCTCAGCGCACCACCGTCGTCGTCAATGCCCAGCAGTACCCCTGGCGCGGCCAGTGGCGTGCAGCATCTCAACATGTTCGGGCATTCGTCGTCCATGCTAGCTCAGCAGGCGCCACATTTCGGCAGCAACAGCAAGTACCCATGGTCATCGGATCCTTTGCAGGGCATGGGTGGTGGTGGTGGTCTGCCAGCAGGGAGCAAGAGGCCGTTTTGGAGCACCGGCGGCGACGAGAAGACGGCGACATTGCCGGACAATGTCGGCGCAGTCATGGCAGACCCAAGTAAGTTCTCCGTGGCGATCGCGGCCGCGATCAACAGCTATATGGGGAAGGACGGGCAGGTGGTGGGCGGCAAGGACGGGGAGAGTAGCAGCAGCAAGAGTAGTAACAAGTGGGGGGTGGTCGAATCACTTCCACCTCCATGACGACTACTAGTAATCAGTAGTGTAAGGCACCACCGAGAGACTAGCTAATTGTGGACAAATTAAGGGGTTTTGCTTTCTTCATAATTTGTTGTGTGTGTAAACAAATCAAGATAGGGGGCAGGCAAACACAAATTGGAGGGATTTTTTGGGCCATTGGTTGTAATCATTTTTCAGGTTTGCTCCATTGGAACAGATCAACTGCAATTGTTGTTAACTGTAAACTGCAATATCTACTGTTTTATTATCACG

>SbWRKY3

GGTTAGAATTTTTCTCTCTCCATCTCTCATCATCAATATAGTAAATAAAATAGAACACATAGACAGAAGAGATAGAAGACTATTCTTTATTCTCAAATAAATATTATTGATTACAACATATATTTTCCATGTATTACGAATATTTAGAGCTTAGTAAGAGCCCACGTGTAAACAGAACATGATTAGGATTCCTTCTTCCTTTTTATTTTAGAATAGAGTCCGCATGTATTACAAGTTCACAACAAAAGCAAAATACTAGTGATTAATTCATAGGTTGAAGTGTGTGAAGAACCCCGTTGGTCTCTCGCTGCCGGGCCTCGTTTGTATACTCGTAAACTTTTATCCTCATAATAAAATACGGTAATAAAATACCGGTGAATAACTCGATCGTGGAAAAAAAAGGTTGAAGTGTGCGATGGTGATGTTCCATCGCGTGTGCCCAGAGCTTGGATGAGGACTGGCTCCGGTAAATTTTAGAGTTTAATTAGTTCAGATTTTCAGGTAATCTTTAGGTTTCAGTAAACACATTTTGATGCCAAAGCTCCTAAATCTAAATGACTCCTAGAGAAGTCTGCTCGGAGCTCTTCAAAAAAAAAAAAAAAGTCTATGCTCGGAACCAATGGGGAGCCTTGGTCAACGTTGTTTGAGACCGTGTGCCCGACGATGCCACTCGTAATGGTCCGCCACAATTAGCGCTACAACTCGCAAATCGTAGATGAGCCTCGCTCGTAAACAATGCAGCTTTAGTTGTAGACAGCATGCGGTCTCTGTGACAGCTAAAACTGAAAGCTAGCCTGGCTATCCATTTGGCAATTTTTGAGATAATCTCAATTCTCAACCGCGTGGCATATAGCTAGACTACAGTTTAGAGACTTTTGAAATACGTCTCATTGGTAGAGGCAATCGAAGACGAATCAAGTTTAGAATTCAGAGACAGTTCGGAGTTCATTCAGACAGCCGGTCGCGCTTCGGCAGCCAGGCGTTGCACTTGACAGCAACGGTGCAACCCAGCGGCCGCCAGCGCAGCCCTCAATCTCTCTCGGGCTCTCGGCCTGCCTCTCTATTTGTCGCTGGTCTGTTCTCTGGTCGGCCGCCGTCCCTGTCATTGCCACCGCCAAGTCTTCCTTGGCTTCTCCAGAGAAGCAAGAAAGGTCGCGGCAATGGACGACCGCCGCGGGCGCCGCGACGCCATGGGCCAGAGGCCGTTCGCGTCTGCTGCTCAGGGACAGGAAAGGGTGTTCGACGGTGGAGGCGGTGGCGGTGGCGGCGGCCCTGGGCCAGCGTTTGGCGGTGAGTTTGATCAGGGATCGTCGTCCCTCATGGCTCTTCTCGGAGCTGGTGGTGCAGTCAGCTCCCAGCCACCGCCGCCGACGTGGGGCGTCGAGGAGGTGACAGCGGCGCCTGCCATTAACCTGGTGCCTCAATCATTATTCTCCATGGTAAACTCCGTGTCTTTAATTTATATTCTTTCTTCTCAGTTTGATGCATGTGTGTTCATTGCAAGTCAGTCAAGTTCCTCTTTGAATCTGAGTTCATTTTATCGTCGTATATGTGTTCATTTGACAATCACGGTATATTTCTACTGCTATCTTGGTCCAATAACTACGATCAGAACCATGTCTTCTGTCAGATGTAGTTGATTCATATGCTCTTTAGTCACTGTTACAAAATCTAAAAGATGCAATGATCTGGCGTTCTAT

TCTAATTACTGGCTAAGGGATTGATTTGCCCATCATTCGTTGGAGTTTTCTAAAGAATTAGCTGGCCAGGGATACACAGCAGGCTGCAGTTGTACAAGTACTTGGCAATGAACTTTCAGCTTTGCACGTTCTAGCGTGTTGGATTATTAATCTACAAATACAAATTAAAGCCTCCAATCTCGGGTTCTACTCCTAAATGCCAACTGGCACTTGCGAGAATTTTATCATCCTTTCATGACTGGTAACACAACGTAAACATGCAATTACGAATTATCACATGCATATTGCAGTACAATTGACAAATGACAATGATACCTCAAAGTTCACAACAAGGCTGCATGTTTCCCCTCCATGCAGGCGAACTACGCGCCACCGCCGCCGTCCTACCAGCAACCCACCTCGTTCGCCCCATCGCCGCTGGGCGGCAGAGTGGATCCATACCCGCCGTACCTTGTCGCGGACCAGCCGCCGCAATGGCCTCCTCCCCGACCGGCGGCTGCTGATTCCTCCATGCCGCACTCCAACTTCACCGTCTTCTTCCCCAGGAATCCATACGACCATGACATGCAGCTGCGAGCGACCGCGCTCTTCGGCGGCAGCAGCGGCTTGCACGCGCACGCCCTGCCGCCGCCGCCGCCAGCCATCGAGCAGCCGGCGAAGGACGGTTACAGCTGGCGCAAGTACGGGCAGAAGCAGCTCAAGGACGCCGAGTCGCCGCGGAGCTACTACAAGTGCACCCGCGACGGGTGCCCCGTCAAGAAGGTCGTGGAGCGCTCCTTCGACGGGTTCATCAAGGAGATCACCTACAAGGGCCGCCACAACCACCCGCGCCCCCAGGAGCGCGGCCTCGCCGGCGGCGGGAACGATGCCCTCGCCGCCGCCGAGGAGGACGTGGACGGCCCCAGCGACGACGACGACGATGACGTCGACGGGGCTCCCGGCCGGTGAGTTATTCTCTGCTTGGTCCCAGTACATTATAAGGCGCCGCGCGCGCAGACAATGTGAACCGTGGCAGTCTGGCGATTGTTTGGTCAGGGCCGCGGACGGCGTTGTGGCCGGGCAGAGGGTGGTGAAGAAGCCCAAGATCATCCTCCAAACGCCGAGCGAGGTGGATCTCCTGGACGACGGCTACCGGTGGCGCAAGTACGGGCAGAAGGTGGTCAAGGGCAACCACCGGCCAAGGTGAAAAATTCACTTCGACTATCTGATCCTGAACATTTTGTGACGTGCACCAACCATTGTGAGCTCTGATGGTGCTCAGTGCATACTCACTGGTCTGCTCAATGCTGTGCAGGAGCTACTACAAGTGCATCGCCGACAAGTGCAACGTGCGCAAGCAGATCGAGAGGGCGTCCACCGACCCCAGGTGCGTCCTGACGACGTACACCGGCCGCCACAACCACGACCCACCGGGCCAAGGCAACGAAGCCGCCGCCGCCACCGTCGCCGCAGGCGGCTCTTCTGCTGATCCGGGCCCTCCGTCCAGGAACACGGCTAGTGGAAGTGGGGCGTTTCAGGAGAACTGGGGGGCTCGGCAGCTGAAGGAGGAGTGCTAGCCGGGAAATGATTGGACTTGGCCATTGGCCTCATATGTGGAGTGTGGCTTTCCTGGGTGTGAATAATAGAGGCTGGATCATTGATTCTTTTGGGCAGGTGTGTGTCTGCCGGCTAATTGTTATTCGATTAGATTTCAACTGTTG

TAGAGGAGCTGACATGTTGATTTCGGCTAAACATGTGCTTGTGCTGCATGGTATATATGTACATTTGTTTTCTCTTTATTTATTTCTTTTATTTTCACTTTTTTTTCTCATGGGAAAACTTCTAGCTGAAGAATGGAAGGAACTCGTGTGGTGATGTAACTGAACCATTAGTAATCGTCAACTGAAAACACAGACATGTATTTCCTTTCAAAGAGAAAAAAAACAGACATGTATATACATCTTCAGAGTTCATAACGAAGAT

>SbWRKY9

ATGGCGGCCCGCGAGGCCTCCGCCGCGGCGCCGGCCCCGGGCCCCGCAGGCGACGGGCCGAGCCGCCCGCCGCGGCCCACGCTCGCCCTGCCGCCGCGCTCCGCCGTCGAGTCCCTCTTCGCCTCCTCCGGCGCCTCCTCTGCCGGCGCCGCCGCCGAGACCAGCCCCGGCCCGCTCACCCTCGCCGCCGCGCTCTTCCCCGATGGCGCGCCCTCCCCGGCTTTCCACGGCTCCTTCACCCAGCTCCTCGTCGGCGCCATAGGCTCCCCGGCCGCCGTACCCTCGCCGCCCTCTCCGTTCGCCGTCCCGCCGGGGCTCAGCCCGGCCACGCTCCTTGGCTCCCCCGGCCTCTTCTCTCCCACGGTATTCCCTCAACCCCCGTCTACGAATCGGACCACCTCACGCCCTGCTCTCCGCTGGCTTGCTGCCTGCCTGCCTCGCTTATCCAGTTGTGCTTGAGGGTTTTTTCTTGCCTGCCGCGTCACGAGTCATCACTCATGAGTAGTACCACTGTACCAGTACCATTTAATCGAAACCGCAGGGAATATGCTTGGTACTACTATTACTGCTTCCGTAATAGTGTTGCGGATTCGTGAGGGGAAATACGGCGTGGAGGGTGCCTACCTTGGACCCGCCCTGGGAGATCTGCCCCAGGAGTGGAAAGGTCAAGCTCGATTACTAAACTAACTCGTGCCACTACTCCCTCCGTCCCTAGCTATTTTTAGGAGAAAATTTTGTCCACAAATACTGCTATTTCTACTAGCATACTCAGTCCACCAGCCCATTTAATTTCTCCTCGGAACTTCCATAGTCCACCAGCTCATTTAATTTCTTCTAGGGAATAGTACTTTGACGCATGGTAATTGTTTGAGCCTGTTTAGTTGGCATTAAATGCAGCTCGTTGGAACTAGAGAACCATGACTTAACGTGCATGATTAAATGATGGAACCCAAATTAAGTGAGGGTAGAATGGTCTTTTGTTTGCCACACTAATTTGTCTGAAATCTGCTAGAAATAGCGTATTTGGGGACGGAGGGAGTACTTTGGAATGTTGTGCGTTACTGTTCTTGATGTGAGACATAGTGTCAATGATCTCTCTGGACTGGACTTTAGCAATTTGGTTTTTTGGGATTACGCTCATATACTTCTGTTCCTTGTATAGTTGATGTCGGTGGAAAAGTCCTGTTCTCCACTTGGTTAGCATGAAAATTATAATATCTCATTTAGGAGAAGTTATGGAAATGGAACCTCAGTAGTTACGCATATTTCAAAACACCATTATTAGATATATAAACTTTGAAGTAGGGATAAATTGAAGATTGTCAAATGCTATATAGGCTTGTTTGACACAACTCAACTTCACTAGTAAAGCTGTTTTTTTAGAAAATAGCTTCATGAGCAACTTTCTAGGTGAAGCTGAGCTGTTTTGGAAAAAGTGTTTGGTAAAATAGCTTCACCAACTACTTCATGCATAGATGAGAGAGAGAAATGAGGGAGTGCAGTAAGCTACTTTTTTCAGCTTCATCCCAACTCATATCTTTGTGAGAGAAGAAAAACAGCTTCACCCATGAAGCTGTTTTAAAAATAAGTGTTTGGCAAAAAAACAGCTCACAACAGCTCATGAAGTTGTTGTGAGCTGTACCAAACAGGCCCTTACATTAAGAGACCAACTTATGCAGCTAAAAACTTTTCCTTCTACT

GCTTGGGCTCTCCACAATGTGTGCCTTGAGTGATGCCCAAAATAGAGGGAGAGGGTTGGGGTATCACCCCTAACGCCGCGCCCGCTGGTGCCATCTATGTAAAAAAAAAAGTTTCCATGCTGCTGTCCCAACAGACACTCACTGTGATGGCACAAGGAGAGCTTGAGGACGTTCAGCCCAACGTGGAGGGGCGGCAGCCAGAGTCATTGGTGGCGTGGAGTTCAAGCGCGAGTTGGTCGTAGGTATCGTCATCGTGGTGCACAACTGCAGATCGATGTTGAGATGGCCATAGAGCCTTGACGTTGGGCGGCTCAAAGCTGCTGTCTAAGAGCTTCAGGTCGCGTTCCCTAGCATGGCCATCTAGGCCACCGTCCCCGCGAAAGGTGGCGCTGCCTTGTTAGAATCTGATACCCCTAGTTATGCGCAGTAGCTCAGATCTTTGAAAGGGTATTGCCTGTATGACACAAGTTTGCACTGTATCAAGAACGACGTAGACGAATCTTGTAGAAAACCCTAGAAGATGATGTTGAAGCAACCAAATACAAGAATCTAGTGCAATAGAGCTGGCTATCCTAATTCTCTTTAGCACTTCCAACTTCTCCTTACAAACCTTCCATGAGACATTGAGACCCTTGTGTGGGTTTGGTTGATTCCTGTGGCCTTCACCCCTTGTTTATATAGCCCTCTCCAAGACAAATACATGAAAGTAGGAGTCCTGTTCTGGTGGAAATTCAGAAACAGCCTGCAGTAGTGGTGAACCTGACTGCCTTTCATGGACAGTTGGTTGAAGAGCAGAGCTGGTGCTTGTGAGGAAGATGAGAGGAGTTTTTGCTTCTATTTTGTTTTTATTTAGCACATGTTTCCTGCCAGTGACACCTACATGTTGCAACCTTTGAAGCACAATACAAGTTTTTCATCGTGGGGCCTGACTTTTTTTTCCAACTTCATGTCCATGCTGTGGAAGGCCTTCAAGGCCATATAAAACTAAAACTTATTGGAATCAACTTAACAAAAGAGCAAGCTTTCTTTTGTTTAATTTTTTGAGGGAAAGCAAGCTTCTTAGTCTTGGCTTATGGGGATAAACCATAATTGCAATACATAAGCTTATGTTGCTCAGTAAGCATGCATTGCATTTTAAAACCGCAATACTATTTTGGTGATGGGAAATAAATTCCAAATGTGTTTTGCACACTCCAATTTTCTCTTTCCCAAGAACTTCAAAATTTGGATGCTGTGCATGAAACTGAGTGTCTAAAGCTCCACATGCTTTGCTCTCATTCATTTGATGCCGTCACATCACTGTCATATAGTTTTATTTTACAATTGTGTCAGCAAGCAGAATAGATGGCAAAAAATTTTTGTTTGCTAATCTCAAGGATAGAGATATTTGAGCTTGGGTAGAAAAGTAAACATTTTAGGTATATCCATTTTTCAAGTTTTTGATGCTGCTGCTATAGACTTGAAACAATGTCATAGAAATACTGTGTTGTAGTAGAGCACACAGTTTGAAGTGTTTGTTGCCAGTAATTGTGTTATGGTGAAAATTATAGTGATCTACAAACTTCGATAAGCACATTGTTACAGATCTGTTTCATGCCAAACATTTTCAGGTTACTCCTGTTTAATGTCTTTGAATGTCTGTTTTTTACTGTTTTTGCTAACAATTAGGTATTACATTTACAAATAAATATGTTAGTGCT

TTGGATTAAGTGCTATTATATTTTTTTCTGCAGGGGAGTTTTGAGATGTCTCATCAACAAGCCTTAGCACAAGTAACAGCACAAGCAGTCCATTCTCAGTACAATATGATAAATCACGCAGATTACGCTATCCCTTTTTCGTCTACAACAACACCAGCTTTGATCACAGCACAGCATGCCAATTCTTCTGCCAATGTGACATCAGCACAGGAGAAACCAGCTCTGCCGTCACATACAGGTAATAGCAAAATTGAATCAAATGAGGTTTCACAAGGACTCAAACCTTCTGCACCCACTTTTGATAAACCTGCTGACGATGGGTACAACTGGCGGAAGTATGGCCAGAAGGCAGTTAAGGGTGGCGAGTATCCGAGGAGTTACTACAAATGTACCCACGCGAGTTGTCCAGTTAAGAAAAAAGTGGAGCGCTCAGCAGAAGGATACATCACTCAAATAATTTATAGAGGTCAGCACAACCACCAGCGACCTCCAAAAAGGAGATCCAAAGATGGTGGTGGTTTACTAAATGAAGCAGATGATTTCCATGAGAATGAAGACACTTCAACTAGATCAGAACCTGGTTCTCAAGATCACTCTGGAAAACATGAGGGGTCAAATGATGGCATAGCAGGGCCTTCAGTGTCAAGAAGGGGAGAGGGACATGAGCAATTGTCAGGCTCAAGTGATAGTGATGAGGAGCGAGATGATGAACAAAGGGCTGGCAATGGAGATCCTGGCTATGCAAATGCAAACAGAAGGTGGTTTTTGTCATGAACATTTTTTAATATGTTTTTCAAGATTCGATCTATAATTGCTAGTTTTATTGAGCAGACATGTGCCAACTCCAGCTCAAAGGATAATTGTGCAAACAAACAGCGAGGTTGATCTTCTGGATGATGGCTATCGATGGCGCAAGTATGGACAGAAAGTGGTAAAAGGGAATCCCCATCCGAGGTAACCACTTTTTTTACTTTGGCTTGTTGTCTGTCTTATTTGACTGGATCTAGTCTGCTGTCAGTTAGAACTTGCTTTGAAAACAACATTTTTTTTAATTGGTTAAATTCTGAATGGTGACATTCATCTGTGATCCGTTCATGATTCGTTACCGGAATGTAATTGTCGATGCATGATAATACCAGTGTTCTGCTAGTCCTTAAATGATTACCAACATTGCAGATGAATACACAGATATGATATAGGAATATACATGCTCTTTCTATATGGTGGTTTAACAAATGGTTTGGTGTCCTGTCATTTTTATTATATTACATCATAATACAGCTGGTGTACAAATGTAATTTGTTCTTTAGTGTGAAATCATAGGCTGTGCGCCATACAGTAACATACCTGCTTTTATTGCATCTAGTATATACTATATACGAGGATTTGCTTCCTTAGTCTTCTTGAAAAGAAAAAAAAAGAATGCATATTAAAGAATTACTAGCTCTTATCAGTTATCAGTAGGGGTAAATCGTAATGAATTTGTTAATTTTTATTTTAAGAATTCCTATACAGAACCCACAAGTTATGGAACTATAACAGCCTTCCATGAATCCACCCACTGTTTTAGTAACAAATGAGACCATTTGATACTCAGTAAAGCCCAAACATGTACATCACTGCTCAATGGTGCAACTCTTAGGGACTGGCACAATGTCACATCAAGCCTGCATCTTTTGAAAGTTAATCTCAATGAACCT

GACGATGTTGCAGTTGCTGAGAAAAACTGACACTATTTCTCTTATCTTTGCTACATTTACCTTTAGTCCATTTCTTTGATGTTCAGTTAGAACCTTTCAGATTCCTTTTTGTTTGGGACCAGAAAGGATCTTTCAGAAAGTGACAAGCTTTAGTCCCTCTGATCCAAGAGATTTCATTTTAGTACCTGTGTTATAAAACAATTTTAGCTTTGGCCATCAGTAAAAACTACTTTTCACGATATTCTTTTATTTAAATGCATTTCACCCTGGAGACCGTGCCAATGAATTTTATTTGGCTACTTGCCCCTCTCAGTTGACTGAAATTAGAGAATATTTCAGTGAACAGCTTGAGCTAGTTGGATTCAAATCGAACGTGCCATGCTCTTTCTTCAACATCTAGCGCCTCCTCACTCTATCTTCAAACAAATCAGCACTGATGAGCTCCACTCCCTGCCTTGTTGACTGAATTTGCTGCAGGACATATTATTTTTGCACTGTTG

AAATTTTGTCATATATTGTCTATTCTCCTATTGAAGTTGAACTGGAAGTTTTTCTAGTTCCTTGGTAAAAAGTGGACAGAATAAACTAGGTTTATGGGCTCAGTGACTTGTTCAATGTGCTGATTTAGCTCTCTTAACAAACCTTGTGCATAAAATTCTGATAGGAATATATGTTGATGTACTTATGTAACTACTTTGCTCAAAGTATCAAATTTCATGTTCAAATTCATCACCAAATAGGAATTACAAATAAGTTCTGACAAATACCAAACAGGAGGAGAAAGAAGAATAAAAACATTTACAGTTTATGCTAACTTATCACTTTTACTCTTCGAGCATTTTTCGAGTTCTTTTATTTGGTGAGGTTTGTGCATGTTCGCTAAATGGGGCTTGTGGAAGGTAGACACTAGCTGGAAGGCCTGGACTTTTTAGTGCTCTCTCCAATCCCAAATATAATTCCATTTAGCCTTTTTCCTAAATGCAGCAGATAAGTCAAATGACTCCAATGCATCTTTTTGCTTCATTCTTCCATGTCTGTTCTCTACTTAAATGAGCAATAATCAGTGCAATACACTTGCCACCCATCTTAACTCTAGCAATGATTGATGGCCAATATAGTCATCATACCTCTCACCTTAATCCTTCTTTACACATACTTGTTAACAATAACAATTCACAATTAAAATATGGCTTGACCAATAGCAGTGAACAATTCACACTGGTTCTATCTGATGATGATTTGTTTGGGGGAATTTGCACGGAGAAACAATTTTAGTTCTATTCATCCTTCACCTATATACTTTGTTTGCACTATGTAGGAGTTACTACAAATGCACCTATCAAGGGTGTGATGTGAAGAAGCATATTGAAAGATCATCCCAAGACCCAAAGGCCGTCATAACAACATACGAAGGGAAGCACAGCCATGATGTTCCAGCAGCCAGGAACAGTAGCCATGCTGCTGCCAATGCAAATTGTTCATCTTCCACCAGTGTACCACACAGGGTCCAGAGTTCAGCGTCCAGTAGTCGCAGAGTGGCAGACTTACAAAGCACATCCTCAGCTTCTTCTATGCTACTAAAAGAGGAAAACGAAATAACATAAGAGTTTTGGAGTGTGTAGCAGGGTAAATTATCTCTGTTCTCTCATCCATTTCCTAGGTGTGCTGCTTCACGCTCTGCGCCGCAGCAAAATTGGCATTGTAAGCTCCAGTCTTTGGTTTTAGTCAAGAGCGAGCTCTTTCGTTTGTGCCCTTCCAAAAGCCTGCACACCCAGTTTGGCTGCGTGATGTATGTTATGATGTGATTACGCCCATCGGTTGTAAATGCTATCAGTCGTTCCCCTGGAGAACTATAGTAGATGACAGCCTTCATCGGCGCAAGCATATATACTCGATTTAAAACCTTTAACTGAGATATGTTTGTAGATACACGGCTTCCTTCCTGGTCTTTGTAAGTGCTATTTCGACAGGATATACAAAGTT

>SbWRKY73

CCTTCCTTCCTTCCCTTTCCTTCCCCCTCTCCGAACTCCGAAAGCCAACGAGTTGAGAGACGAGAGACACACAAACACTGCAGAGGAGAGGAAGAGAGGAGCCGAAGTCAGAGTCTGCTCACCCAACCCAAAAAAACCTCCACAAACCAAAGTCAACGCCGGAAACCAAGAAGAAGAAGAAGAGGGGAATTCTCTCTCTCTCTCTCTCTCTCTAGTCTTCCTTCCTCTCTCGCCTCCGCCGCTTGTCCGCGACCATGTCCGCGCGCCCGCCGCCGCCGCCGCGCCCGCGCCTGGCGCTGCCACCGCGCTCGGCGGCGGAGTCGCTCTTCACGGGCGCCGGCGACGCCAGCCCGGGCCCGCTCACGCTCGCCTCAGCGCTCTTCCCCTCCTCTGACAGCGACGGCGGCGGAGGCGGAGGCGGAGGCGCCAACTCCTCCTCCGGGGCCGCGACGACCTTCACGCAGCTCCTCACCGGCTCCCTCGCGCCGCCTCCGCAGCAGCAGCATGAGGCGGAGAGGGGACGAGGAGGAGGCGGGGTCGCCAGGGCCGGCCCGGCGCTCTCGGTGGCTCCGCCGGCGTCGGCGTCCGCTGGCGCGTCCGTCTTCACCGTGCCCCCCGGCCTCAGCCCCTCCGGTTTGCTCGACTCCCCTGGGCTGCTCTTCTCGCCCGCCATGGTACGTCGTGACACCGGCTGCAACTTCTTGCTGGAAGAAAATATTAGCTTTTGTTTGTTTCAGGCGCGAAAAAACAGATTTTTTTTTTGGATCTTGCTTGCGAGCTGATAAAATTGTTAAAACCGTATGTATGCTATAATCGCCTTTTTTTGTCGTATTTATAGTGTTGCACTACGGTAGCGTTACTGCGAGATATTAAGAGAGACAAGAGTGTTATTGTTTCTATTTTTAACACAAAAAGCGGTTTTTTATGTTCCTTATTTTCATAGTTCATAGATTGTTGTGAAACATGTTCCATTTCTTTCGCTTGTGACATGGCTTTAATCTGGGTTCATCAATCTGTTCATCTACGGGATTTACTTTTGGATGATGCTAAAAAAACAGGAAAGAATTTTTCAAGCAACATGGGAATCTGGAACTTCTGAAAGGTGCCACTTTTGCAGTTCCGTCAGTGCTTTTAGTGAGATGGTTACATATAATGTAGTACTGATCATGTAGATCAGGCCCATATGTTTGTCCCGAAACGCATAAAGCCACCTTACTATATTTGTGTGTGGCACTTCAGGGCACTGAATAATATCGGCCTACTAAGATAGATAGGTGATGCTTCTTGATCTTTTAGAAATGCCAATTGCCAAGTGGTGATACGGTACATTGCATTGCATTAACATAATTGTACTACTGCCAGTGTGATGGTTGCAAGTTGGCACACTATCTTGATACTGATAGGACAAACTTTCCTGTAAAACAAATGTTACTTGCTTCTTGCTTTTGGTGTTCTATCTATTCATAGAGTAATTTGGAAGCATGTTGCTCATTTTGATGCGTTTATATATACTAGCCTCCAATTTGCTCTGCTCTGCTCTGCAGCAGAAGTTGTAAAAAAAAACTCATAGTTTGTTTGCATTGAATCAATGTTCCATATTCACAATATCTGGTAGGTATATAGCAATTTGTAGGCTAAACTTGTAGCTGGACTTGTAATTTTGTTATATAAATCCTGAACTAAGGAAACCAGTATAAATG

GGGCACAGAGTTGACCAGTTGATGGTAAGAACAGTCAAACTAACGTAAATTTTCATTCAATATTTCCTACCTAAGCCGTGTCCATTTCTGGAGCTTCTGCTTTAGTCCTGAAGAAATAGAGTGATTTTAAATTTTCTGATGCTACCTATTCAATACGTCTACAGGGGGGTTTTGGAATGTCGCACCAGCAGGCTCTGGCTCAGGTGACGGCCCAAGCAACCCATTCTCCACTCAGAATGTTTGATCACCTTGAACAGCCATCTTTCTCCACAGCTGCTACAACGTCGGGAGCTCTACAGCATATAAATTCTGCAGCCAGTATGGCAGGAATTTCAGATATGACAATGGCAACAGCAAACAATGAGAATGCATCATTTCAGTCTGCTGAGGCATCTCAGAGGTATCAAGTTAATGCCCCTGTTGATAAGCCTGCTGATGATGGCTATAACTGGCGGAAATATGGTCAGAAGGTGGTAAAGGGCAGTGATTGTCCAAGAAGCTATTACAAATGTACTCATCCCAGTTGTCCGGTCAAGAAAAAGGTAGAGCACGCAGAGGATGGTCAGATATCTGAGATCATATACAAAGGCAAACACAATCACCAACGTCCACCAAATAAGCGGGCAAAAGACGGCAACTCTTCAGCAGCTGATCAAAATGAACAATCTAATGACACCACATCTGGCTTGTCAGGTGCCAAGAGAGATCAGGACAATATATATGGGATGTCTGAGCAAGCATCTGGTTTAAGTGATGGAGATGATATGGATGATGGTGAATCAAGGCCACGCGAAGCGGATGATGCTGATAATGAGAGCAAAAGAAGGTAAGTTTAGCTATCAATGTAACTCCACTATGATCAGATTGTTGTTTCATAAAATTAATTCCTTTAATGCTCAGGAATATACAAATTTCTTCACAAAGGACCTTGTCAGAGCCTAAGATTATTGTTCAAACAACCAGTGAGGTTGATCTTTTGGATGATGGTTATAGATGGCGCAAGTATGGACAGAAGGTAGTCAAAGGGAATCCTCATCCAAGGTATGCTGTGTCCTCTTCAGTGTGATCTGCATCATTTTTATACCTTCTCGCTCTTTTTGGAAAGTGTGTATGGAATGCAAGGGGTAGCAACATTAAGATTTGTCTGTCTCTGCATTAATGTCACAATAATATATTCAAAGTTATTTTGTGGAGAATAAATGTTACCAAACTCATGCCATCTATTGGATAATATTACCGTTCTTACTTTCACTAGTCACTACTATATGTACAATCTTTCACTTCAGCCTTATGAAGTTATATATTTTGGTTTCATTTTCATGTCTCTTATAATCTGCTGGAAATCCTGTAAAAAATAAAAGGAATATGGTAGGAATTGCATATGTGTGGCCTTGAACTATTCATCGATAGTCCATACACATTTCGATATTCTCTAATTCATGCATCAGTACAACTTGCAAGCAGTGTTATCAAGTCGTCCGATTAATCGCGATTAGTCGCAGAGTCGATATTCTGGACTTGACTAGGGCGGTTAGTCATCCTACTCGATATCCTGGACTAAAACTAGGGGCAACAACTTGATTAGGCTGATTAGCGATTAGTTTTCTGGTTGTCCGATTAGTCGGACGACTAGGGGGTGGACAACCAGAAACATAATGGAATTGCTGGGCATCTTAGGACATGGGCTGCATCTGGGATGG

CTTAGGCCACTTGGGCTCATGTTTTTCAACCTAATTTGCAACCCTACATTCAGCCACTACCCTAGAGTTATCTTCATGCTTGATTTGAACTCTAGAACATATATACTATAGTATATTAGTATATATTTGGTATATTAGTCCTGATATACATACGACGACTAGGACTGACCAGGCTGACTAGTCAGCCTGGTTGACAGACTAATCATGACTAGTCACCTGGTCGCTCCCATGGCGACTAGCCGACTAGGATTGATTAGACGACTTGATAACGCTGCTTGCTTGCAAAGTACCTTTTTTCTTCCAGTAACAGTGAAATGCTAGTGTGATGCCCTGGCTTGTGTTATTGATGGAGCATGTTAGCTTAGTTAATGGTTCCTTGAAGCATGTCTTGTTAAAATATCATGTGTTTCTCTCCCTGAATGAGTTGGATATGATGGGTGCATGTCTAAATATTTCATTTAAAAGGAACCTACCGTTTATGCCAAAGTAATGTAGCCACTAAACATCCGTGTCTAGTTAATCAATTGCTTTTGTTTAATGTGTCATCCAGAACTACTTTAATGGTTCCATCAACAGACTTCTGAAATAGCTTCTAGCTCAAAAAAGGAAAAAGAAAAACCTCAGATTGCTTGGCTTGTGCTTGCACTGTGTGGCTTATTGGTGGAGAAGAGTTAATAAGGTTTATCCCGTTCCTATCAAATGATGGTAATATGTCCACACTGATCAATGACACACGATATGTTGCATGAACGGCGGTGTCAATGGCAAGAGGACACTGCCATTCTTGGAAACTGCACCAGGGGTTTGAATGCCCTGGAGGCCCCATGTCGGGAGCGATGCCTCCGTGATGGAGTGCATACCACCAACATCCCTTACAGCAGGAGCGCTGCCTGAGTCTACCCTCATCCCCTTCTGTGGGCGGCTGCAGCTGCAGCCTGCAGCCATCTAGCTGGCTGCTCATGTCAGAATGCACGCACGGGCTGATGGACAGATTGGTCAGAGATGCTATTATCATGGACTGTGGTTTCTATGATGATATTAATGAGCGTCTTAGACTTCTTTATGCTGTCCTAAAGCATTGCCATTCTCTTTAAACATTTGAAATCTAGAATTCTAGATACTAAGTAAAGCCTCAGAGTGTCAGGCGTCATCTGTGTAATTAACCTTAGATCACATATTCCATCTGAACCATTCTATTGTTGTCATGAATTTAATGGCATTTTATCATTTCATGGCTAGGCTAGGCTAATGAGAAGTCAACCATTCAATGCCAGAAGTGAATGCATTGTTAACCGCCATAGACTGTTTGGTGGTGAAGTTTGAAAGAAAGGGTAGACTAGTAGGATAATGAAGAAAAACTGAGTAGCTATAATCTGAGCCTCATAAAATATTTCTAGAAACGATGGGTTAGCTAGAGGTTAGATTATTCATCGAAACTATCTTAACTGGGGGGCAAAGGTTGTTGGATGAAGCTATGAGAGGACCGCCTGCATGATATACCTCTCTATGCTGCATTAAATAAAACCATGGCATGTACTTTTACCTTACTGTTTTACTATTCACTCATTATCTAATGATACACCTCTCTACCTGTAGGAGTTACTACAAATGCACATTTGCTGGATGCAATGTCAGGAAGCACATTGAGAGGGCTTCGTCAGACCCTAAGGCTGTCATAACAACCTATGAAGGAAAACATA

ACCATGAACCACCGGTTGGTCGGGGCAACAACCAGAATGCAGGAATATCACAACAGAGAGGGCAGAACAACATATCTAGTAATCAAGCTTCACTTCCGAGACCAGACTTCAGCAACACTAACCAGATGCCGCTAGGGATCTTGCAGTTCAAGAGCGAGCAATAGTAACAGAACCTTGCTTACTGTTGTTTGGGTGTCGTTCTTTTGGCAAATAGGATGTCTGCAATTCGTACAGTCATATGGCATCAAAGTTTCTTAAAAGTCTGATAATTGAAATGGTATCAGTTTACAGTTTTTCCCACAGATACACAGGATAGCTGATACGGCACCATCATTTGTAGTTTGTACATGTTATTAAGTTCAAGCGTGCAGTATATGCATATGCTTATGATGTTTTGTAGATGGATCAGTGGTATACATGTTTATCTTCTGATTTTATTCCCAACATCCAACAAGATCTTGTATGTATTGTAATAAGCTTGAAAATATAAGTGAATGTACAGATGTTTTTCAGTTACGTTTTACTTGTAATTTGTAACTTTCAAGATATTCTAGTTTTCTATGTCCTTTCCTTTTCTTCCTTCCAAATTTGGTTTCCTTAGTCTTCTATGATCACTATTATCAACTATCAAGAATACGTTGAAGGTACTACAAAAGCACCATTTTGCATCTGGCTGCAAACTACCCTGGATGTCTAAGCCGGTAGGAGTAGGACTGCCTGTACAATTCTTATTCAGTTCTGGTGCTGCTATTTTCGTCAGACCAATTCCAAAGCTATGTAAATTCAATCTGAGAAAAAAAAACATTTTACTTTTGCTTCGATTTTCTTGTACTAAATAGTAAATTGAAACTTGGTTTTTTTTTGTGAGGAAATTGAAACTCAGTGTCTAGCTACAAGTACAGTGATTCATGTGCGAAGGCTAACTATCTCAGATCGACAATTGGCATGCCTGCCCCACCGTTATATGAGGTACAATAGTTTTTTTTTCTCAACCTTGGCACTTCCTCTGTTTCTAGCCTAACGGCAGTACGTTTTTATTAGAAAATGGAAAAGGTGTCCGGCCTCTACGACCGTGCAGATATCCAAAATAAATATATATGTAACCTATGTACCTAAGCATATTTTTTCATTCCTATGTCGTGGACACTATTATCACTGCAAAAATTTCATGCATATCACTGTCAAGATCAGCTAGAATGTCATATCTAATGTTGCTGCATCTGCTCCGAATCTCAGGTACAATTAGGTTGGTTGAGCACGAACTTTTTGAAGGAAAAAAGAATGCCATTAGGCATTAGGTCATAACCTCCATGAATGATTTTCAGAGTGAAATTTGACAAGGACATTTGATCCTAA

>SbWRKY19

CGGTCAAACCGCCTTTCTCTCTCCTACTCTTCTCCCTCCCCCCTTCCCCGGCTCTCCCTCTTCTCTCTCTAAAAAAAGTAGCGCCCGGAGAACCGAGAGGCTGGACAAAGAAAGGCGGAAAGAGGGAGCAAAATCTTGCTTTGTTGGGAGTTTCTTTCCTCCGGTTCCGACTCCTTTGTTCTCCATAGGGTTCTTGGGTTGGTGGAATGGAAGCTCAGCAGCTCCATTTGTGCCCCCGAGATCTTCGTCGTCGTTCCCCTCGTGTGTAGTAGCCTTTTGGTAAGGTTGGTGGAAGCAGGGCCAGGCACACAAGAACATCGATAAAGGGAGTCCCCCGTTTTTTAGCTCTTCGTGTTCTTGTAGGAGGAATTTCCGTTCACATGATCCGTGCCTGTACCTGACGGCCTTGTCGCGTTCTGCTGCTTCGCTTTCGGGGAGAAGAGGACTCGACTCAAATCATCTGGTGTCGGAGACGTCGGCCTTCCCAGTTCAGGTGCCAGTTGCAAGTCCTCTCCTGTTCTATTTTGATGACTGTAGCTTGTCTTGCATCTGTGTTTATGTTTATGATGAAGGCGGATAGGTCGGCATAGAATTTTGCTTTTGTTCTTCAGAGCTGTTGAGGTTTGATGAGATCGTTTGGGGATCGCAAAGTTCGCGTCTTTGGATTTTATTTTTCTTATATCAGTTGCGGCGATTCAGTGCTTTCTTGGGGGGAAGTTTGCCGCTTGGTTTGCAATTTACTTGCTTGGGCTGTAATATCGGTTCTTCATTCTGCATGCGAATGTGTGTTTTCGGTCACTCGTTAATGAATCTTCTGGAAAATTTGGTAAAGTTGCAGTTTACTTTAGGTGATTTGGCAACTCTTCAACTCCTGTATTTGACTTGTGTGTCATCTCTATTTATTTTAGTTGACGTTTGTTTACTGGATTTAGTTGGTCTGTGTGCTACCGGCCATATACGGTTTATTTGGTTTTTGTGTTCTGCCTTTTGACTAATTTCTTCTGCTATATGATCCCGATTGGTTTTTAACTTTTGTTTTCCTCGCCATGTAAAGAGAAAAAGAAAATGATTTTTTTTACTCAATTTTACTACCGGGCAACCTAATGTTTCAACATGACTAACGTGATACGTATTACACGGCACTCGTTTCAGTTGCGAGAGATTTCTGGCATGGCCGGCGCAAGTAACCATGGATCCCTCATGGACGAATGGTTGCCGCCCCCTACACCAAGCCCAAGAACACTCATGTCAAGCTTCCTGAATGAAGAATTCAGCTCCGAGCCATTCTCTGGTTTTTTCAGTGAACATGGCACCAACAAGCCCCATGATCAATCCGAAAAGAGCAGAGAAGTTGTGAATTCGAGCGAGGAGGTCCCTGCTCATGCTGTCAATGATCCATTTCAAAAGGGTTTCTCCCTGAAGCCAAATTTGTTCAGTGCTAATCATAAATCAAACTCCAATGGTGGTTTGGCGGAGCGCAGGGCTGCGAGAGCAGGTTTCAGCGTCGCAAAAATTGATACTTCTCGAGTTGGTTCATCAGCAGTTATTCGATCTCCTGTGTCAATTCCACCAGGTCTAAGTCCAACTACACTTCTTGAGTCTCCTGTGTTTCTCTACAATAAAATGGTATGTGGTATTTCCTTAATTGGTTTCTTGTGCTCTCAAGATTTGTATGTTACACTATTATCCAAGGTTGAAAT

AAAGTCATAACTTGAGCAAGCACTGAAGACTTTCATATTGCAGTTTTCAGACATTGAATTTGGTCCAGTATAGAAGAACATTGATTGGTGCCTACTGTATAGAACATTATTTTCTGCAGTATACTTATGCATGTGACTACAGCACCGATTGCACTGTGCAAAACATTGTCATATTAGAAATATAGTCCTCTGATTGGTGTCTTACAAGCTTGGCTTCTGTTTTGATTCTATAGCTGCTAATTTGGACAAACACTTACGAATTCATCTCTGTGCCTGCCCTTTAATATTTGCCATGCGGACTTAACTGAATTGGTTATGATATATTGCAGGCACAACCTTCTCCAACCACTGGCACGTTGCCATTTTTGATGGCTACGAATGATAAGTCAACAATACCACCAGCTGCCAAGATAACTGAAGATTCTCCATTTGATAATGATGTGTTTTCTTTCCAACCCCACTTAGGTTCTGAAGCAACAGGTTTCTCTACTGCAGAAAAGGTGATTTCCTTGATTGTTAGTGTCTACATATATTTTCCCTCCTTTTTGCAGTGCAGTGTTATAGTACTAATTGGCTTCCTTCTAATGCCAACCTCACCACCAAAATATCCTCTTTTTGTTTTTTTGTATAAAATACCTTTTAATTGATTCATGTGCCTGAAAGAAGTTTTGATCTACAGGACTATGGCGCCTATCAGCAAAAGCAGTCCTTGTCGAATATTCATCAGCAGGAATCCAGTCTTCAGTCAAGCTTTACAGCAGTCAAGGATAACACTAGTGCAACAATTGTTAAAGCGAAGACGTCTAGCTCCATGTTCAGTGATAGTCACTATTCAGCTGACCAACAGCAAGCTGACGAGACAAATATAAAGGTGCAAGGCAAAGGTGTCGAGGCTAGATCAGCTGCTTTTCTTCCTGTATCAGCGCATAGTGATGCATCTCTCTTGGAGTCTCAAGATGCAGTTGATGTCTCGTCAACACTGTCTAATGAAGAGGAGAGGGCAACGCATGGTACTGTTTCTATAGAGTGTGATGGTGATGAAGATGAGACTGAATCCAAAAGAAGGTTAATATTGTCCATCAGTTAACCACTTAAGACTTGATGCAGACATTGTACTGAATTCACTGATACTCTTTTTTTTCAGGAAGCTGGAACTAGATGCTTTAGGAGCTACTGCTATCACTACTACCTCCACCACCAGTACCATTGACATGGGGCCTGGAGCCTCAAGAGCTGTCCGGGAGCCTAGGGTTGTTGTTCAGACCACAAGTGAGGTAGACATTCTTGATGACGGTTATCGGTGGCGCAAGTATGGGCAGAAGGTTGTTAAGGGCAATCCAAATCCAAGGTCACACTTTCACTACCATTTCTTACACTGATTAACTGTATTCCTCCATCCCCTGAAATTAGTAACATTGATTCACCTGTCTGCAGGAGCTACTACAAATGTACACACCCAGGATGTTCAGTGCGCAAGCATGTGGAAAGAGCATCACATGATCTGAAATCAGTCATCACAACATATGAGGGAAAGCACAACCATGAAGTTCCAGCAGCCAGAAATAGTGGGCAAGGCAGTTCTGGTTCCGGCAGTGCTCCATCTGCACCACAAGCTGGTGGTTCTCACCGTAGGCAAGAATCAGCACAAGCCAGCTTTGCTCACTTCGGCACGACCAGTCCTTTCGGCTCCTTCGGTCTCC

CACCGAGCAGACAGTTGGGACCAACAACTGGCAATTTCCGCTTCGGGATGGTTCCGCCGGGCATGACGATCCCAATGCCCTCTCTAGGATCACTTGCCCCTACAAAGATGGTAGGAAGTTCATCAAGCATGCAGGGGTACCCAGGGCTTATGATGCCAGGAGAGCCCAAGGTGGAGCCTGTCTCGCAACCCCTCTTCCCAATGGCAAATGCATCTCCACCAGCTTACCAACAGATGCTGAGCAGGCCTCCTTTTGGTCATCAGATGTAAATAAATAGGAAAGAGAAAAAGGATAGATTTGGTTCAGCTTGTATACATGATAGCGACACTGCAACATGGCTTTGTTCTAGTGTTCATGGATCGTCCAATTCTTAGCTTTATTTTATTTTTCTGCTCACGCATTTCTTTGAAGCAAAGAATGCTGAGGATGATAACCTGTCCTGCTGTTGTAATCAGGGAAAGGATACCTTCGTCTTTTTGAGACTATCGAAATTGGAAAACGATCAATCAAAATTTCTGGTTGTGAATACAAATCTGTTGAAC

>SbWRKY67

CAGCTCTGCAACTCGGCGAGCCTATCGCCTGCTGCAACGAATCCCTCCAGCTTCTCTTTCTCTGCCGCCGTTCCCTTCGCCTCCTCTCGGCCAACCGCGCTTGTCTCCTCCTCCTCCTCCTCTCTTCCATCTCCTCTCCCTTTCCTGGGAAAAAAAACTGCTTGCTCCCCATAAATATCAGCAAGCCACACACACAGAAAGAGAGAGAGAGGTGAATCCAATTCAGTCGTCTCGGAACAAATTCTTCTTCGTGGGGGGGCAAGAACCTGATTTCCACTGCCCCTGACAAGGTTTTTGCCGCCGTGGACGGTAGGTTCTTGCTGTGGTCATTTGGTGAGACAATTGCGGGGTGAAAAGGAGCCGGAAAAAGCCGTAGAAAGACGGCAATTTTTGTCCATAGTCAGAGGCCTGATTGTTCTGTTCTTATCCCCGCGGGCGAGCGATTTGACCAGCTTGCCGTCTCCGCAGCAGGCCATCCATCCACCGCCATTCGCACATCCGCCGCCTCGCCGCCTCTTCCTGTCCCGTCGCTTTGCTTTGTCAAGGAACAGCCGTATCGTTGCCGTCGACCGCTCCAGGCCTCCAGCCACCTCAGGTGCCCATTTCAATGCCGTGTCGCGTTTAATTCCTCCGTGTTTTTAAAAGTTTGTGGTAAAGGCCGCGCCTTTGTCATCGAAGCCGACGATTTGTGGCTTGTTCCTTGGATTGTTTGATTCGTGTGTCGAGTTCGAGGTTTATTTGGTTCATATAAAAAACTGCAAAATTGAGATGCTAGTTGCCTGTGGTTTGTGTAAGGGAATTGTGTTCGTGGATCGTTTTGCAAGAATTGGAGACCTGACGTTCTTTTCATTTTTACACCGTGGTTGTTGGCATGCTTGAGTCGAACTGCATCTTGCAGTGGTGTGCTTGGATTCAGTTAAAATGTCAGTGATTTAAGGTCAGCTGGTTTGGCTGTAATTTAGTTGCACATAGGACTACGAATTCGTTCAGTAGGCATGAAAGAACTCATGTTTATATGTTTACTCTGTATTTTCGTGTGCATGAACTCCATACCTTAAATAATCTCCTGGGTTTAGCAGTAAAGTAGTCTACTCTCAATT

AAGTTACTTTACTGTTGCTGGATAGTATGTGCAAAGGTTGCCCCATTACCTGTCCATATCCTGAGCTCAAGTGTCTGTATATTAAATGACTGCTTCGATCTTCAGTTAGCTGTTCTCATTTCCAGCTGTGCTATCATGTGCCATGTCTAGCAATATCATTTGCTTTTTCCTATGCACAGTTGATAGAAATACTGCACTGGCTTGATCTTATTAACACGATTGTTTTGTGTTTCAACTAACATAATGGTTTTGCTTTTAAGGTTTAAGTCTGAGTTGCTTCCCTTATTGCTGGGACAGTCAACCGCAGCGCATTCATGGAGGATTGGATGCTTCCCTCACCCAGCCCAAGAACACTGATGCCAAGCTTCTTCAACGAAGAATTCAGCTCTGCTCCCTTCTCCAACATCTTCAGTGATGATAGAAGCAACAAACCCCTGGATGAAATCGAGAAGAGTAAAACTTTCATTGGCTCGAGTGCTCAAGAAACCTCTCAAGATACGAAAGACCATCCACAGACTGAATCAAACCTTTTCAGCGCAAATCAGAAATCAACCTCTCCAGGAGGTCTTGCAGAGAGGATGGCCGCAAGAGCGGGTTTTGGTGTCCTGAAAATTGACACGTCCCGTGTCAGTTCATCTGGTGCACCAATCCGGTCTCCGGTGACAATTCCACCTGGTGTGAGCCCAAGGGAACTTCTTGAGTCACCGGTTTTCCTTCCAAATGCCACTGTAAGTGTCTGTATTCATTGTGAGATTTTTGTCTGGGTGATACTGTGAAGGATAGGCTGGCCAGTAGCTTTGAACTATCTTCTAACCTGATAATCTAATTGGTTGTTTTTTTACCACTTTCCAATGTCTTATTGCGAAGGTGGAATTTTCGAAGATGAAAAAAAGGTGAACCAATCTTTATTGTCTGAACATTCCGTAACTGTTTATATCTCTGCAGTCGCAACCTTCTCCTACCACTGGTAAATTGCCATTCCTGATGCCTAATAATTTTAAATCAACAATGCCGTCCGTCCCCGAGAAGTCTGAAGATCACTCACATGAAGATTCTGCGTTTTCCTTCCAGCCCATTTTGAGGTCTAAACCATCAACCTTATGGACTGCAGAGAAGGTAATTTGCCTGATCACATATTTATTGCCATTGCTATTTCTTATAATTGCATTTCCTTTTTTTTTGACTAATTCAATCTGCAAAAGCATATCTAGCAGTTAATAATGCACTTATTTATTTTTTTTAGATAAAGGATAGTTCATACCCGGCTTCATCTACCAACTGGTAGAATCAGACCAATTATTACAAAGTTTAGCCCATAGGCTGGCATATGAGCCATAAAGTGATCTTAAAACGAAGTACAGTTTTTTGACTAAGCGAAACCAATAGACCTATTTGACAACCACCCCTTGGAAGCAAAGAAGTGCAAAGCTGATGTCTCCAGCAACAGCTGATCCTGATGATCATCACGCCGCTGCAGTCTTGCTCATTGACGGAGCCAGTGGGTTCCCCTGAAAAGAACCTGCAAAAAAGATTTTGGTTTACATTTGTCAAAAACCACCTCGTTTCTTGTTATCCAAACTGCCCAACACAAAGCTGATGCTGCAGTCAATAACAACGAATTATGTTTGTTACCACCCATCTTAGACCACCTGTTAAACAAGTCATCTATACTCTGAGGAGGTGGTATTCCAAACATTAAA

TGTACAGCGCGCCACAAGAATTTGGCATAAAAACAATCGAAAAAGAGATGTTGAATACTTTCTGGTGAGTGACATAAACTACACCTCGTATCGCCATTCCAATTTCGTCGAGCAAGGTTGTCTTTAGTTAAAGTCACACCTTTTTTTAGATACCACAAAAAAATTTTTATTCTTGTAGGAATTTTAATTTGCCATATTTCCTGTGACACTCTAACCCCATTGTTAATCAACGCATCATACATAGATTTGACGGAAAAAATTCCTGATGATTGTAAAGACCACACAAACACAACTGTACCCTCCTGTAAGCTCACATCATGTAAAGAGGCAACAATTCTGTGCCAATCCCTTAGGTTTCTGCCCACTAGATTCCGACAAAAAGAAATATTTAGTGGGATTGAGCTAAGTACTTTTGCTACAGAATCTTGTTTCCTTCTTACTATGTTGAACAACCCAGGGAATTTGTCCTTAAGTGGTTGATTGCCTAGCCAAGTGTCAAC

CCAGAATCGTGTTTGTGTTCCGTCTTTCACCTTGAAAGAACCAAGACTGAAAAAAGTGTCTTTAACATTCATCAGACTTTTCCAAAAATGAGAGTCCGTTGTTTTTTTGACGCAGCTCGCTACAGTTTTGTCTTTGACATATTTATTCCTCAAGAGTTCCTGCCACATACCATCTTCATTTAACAATTTAAAAAGCCATTTACTCAATAAACATTTATTTTGTAAATCTAGATTTTGTATTCCTAAGCCACCTTGTACTTTTGGCTGGCACAAAATTTCCCATTTAACTAATCTATATTTTCGTTTGTGTTGGTCATTCTGCCAATAAAATCTTGATCTGTAACATTCAATTTTTTCTAGCACCCCTCTTGGGATTTCAAAAAAAGACATCATAAACATCGGTAGACTAGAAAGCACCGAGTTTATTAAAACTAAACGGCCTCCTGTCGAGAGCAATTTTCCTTTCCAACCACTGAGTCTATTTTCAATTCTATTCTCAATTATTTGCCAATCTTTATTGCTTAATTTTCTAGTATGCATCGGTATGCCTAAATAACGAAAGGGGTATGTCCCAATATCACATCCAAATAGTTGTGAATAGAACATTTCTTGCTCTTTTGCTTTACCAAAACAAAAGATTTCACTTTTATGAAAGTTTATTTTAAGTCCTGATAGTTGCTCGAAGGTAATAAGAACCAGTTTCATATTTACTGCCTTCTCAACATCATGGCTCATAAAAATCACTGTGTCATCTGCGTACTGGAGGATAGACAAACCATCTTGAATAAGATGAGGGATGACCCCTTCTACTAGACCTGCTTCCTTTGCTCTAGCAATCAAAATGGCCAACATATCCACCACTATATTAAATAGTATCGGGGACATTGGATCTCCTTGTCGTAAACCTTTGTTTGTTTGGAAGTAGGATCCTAGATGATCGTTAACCTTGATATTAACATTCCCGCCTTGCGTGAACTGCTCAACCCAGGTACGAAACTTTTGTGAGAACCCTTTCATCCTTAGAGTTTGTTGTAGGAAGCTCCATTTTACCTTATCATATGCCTTTTCAAAATCTATTTTAAAAATGATCCCGTCCTGTTTTTTTGAATGTAACTCATGTATTGTTTCATGGAGAATTACCGCTCCCTCCATGATATTTCTTCCTGGAAGAAAGGCTGTTTGAGTTGGTCTAATGATCTTTTGGGCAATAGTATTCAGTCTATTGGTTGCTACTTTTGTGAAGATTTTGAAAGAGACATTCAACAAACAGATTGGTCTGTATTGCTGAATCACTTTCGCATCCTTCGTTTTTGGTACAAGGATTATTGTTCCAAAGTTTAAACTTTGTAAAGGCAAATGCCCCTGATGAAATTCCTTGAATAGAGCCATCAGATCAACTTTTATTAGATTCCAGAAAACCTGATAAAACTCAGGAGGAAAGCCATCTGGGCCAGGTGCCTAGTTATGTTCCATTTGGAAAATCGCAGCCCTTACTTCAGATTCAGAAAAATCATCTATCAAGTACTCATTTTCCAGTACAGATACCTGAGGGATATCCTCAATCTGTTGCTCATTGAGGAATATAGGAGAGCTTTCCGATGGGCCAAACAAATTCTTATAATAGTTAGTAATATGCTTTTTGAGTTGAGCATCACCACTAATTGTATTGTTTCCATCCTCCAACTGGAAAATATGAGTTT

TTCGGTGTCTACCATTTGCCAACAGATGATAGTATTTAGTATTTGCATCCCCTTCCAACAGATGTTTCACCTTAGCCCTTTGGTACCATTTAAGTTCCTCCTCTCGAAGTAATTCAGCTAGCCTTTCATTTAAGACATGTTTTAAATTTAATTCCATTTCATCAAGCAAAGAACTTTCTGCCTTTTTATCTAATTCGTCAAGTTTATTTAATAACATTTGTTTTTCTTTTCTATATGCACCACTTATATTTTTAGCCCATCCTCTGAGATATTGGCGTAACCTTCTGATCTTAGCTTGCCACCTCTCTAAAGGAGTGCCTTCAACCAGCACACTTTCCCACACATCCCGTACCAAGTCACAAAATCCCTCCCTCAACAACCAACCCAATTCAAATTTAAATTGAGATTGGTAAGATGAGGATGCATTATTTGTGCTTAATAACAACGGAGTGTGGTCAGATATCTCTCTCGATAGAGCATGTACAGTGGTATGCGGGTATTTGGATTCAAACTCTGTACAAACCAAAATTCTATCTAGCTTCTCAAAAGTCTGGTTTTGTAAGTGGTTGGCCCAAGTAAAATTTCTACCTGACATTTCTATTTCTCTGAGATTCAAAGAATCAATTACCGCATTAAAAAGAAAAGGCCATCTATCATTGTAACTAACATTATTTTTCTCGTCAGGACTACGAATAATGTTAAAATCACCACCTATAACAATAGGTAGGGTGTCTTTTGAACATATTCGTACTGCTTCAGATAAGAATTTAGATTTCAGATCCACTTGAGCCGGCCCATAAACTGAAATCAGGTTAAACTTAAAATCGTCTTCCTTATGTCGAACCTTGAACCGAATAGAGAAGTCACCTTCCTCAATTTCACCAATATCAAAAGAAACCAAATTTATTCCTAGAAGCATGCCACCAGACCGACCCTTTGGCCCCATGCAATGCCACAAAAAATCTCTACCAGCGCAAATATTATTCAAGGTGGATTGCGAAAAATTGGCTCTGCCGGTTTCCGATAAAGCAATAAAATCTAGGCATTTTTCTTTTGTTAAGTCAGATAAAAATCTATACTTCTTAGTATCAGCAAAACCGTTACAATTCCAAAATATTCCTTTCATCTAGATTGCTTTGTATTATTTTTTTTCCTATTCTTTGAGCGTGCCTTTGTTTTTTGTGATACAGGGGTCTGTAAACACAATGAGTCACAGCCCCCATCACCTAGGCCTTCAGAAATTTCTGAGCAAATCAAATTCAAAGTTTGTAACTCCAGATTATCTTCTAAAGAACAAACTGAAGAGGCAACATCCAACACACAACTATTCCCAGCAGCTTGCTGGTCAACCTTTTCCAGTCTAAGAAATTCTAAATCTCTAAAAGTATTTATAGAGTGCGACACTTCTTGCTCATTATTACCAAGAACCACCCCTAAAGAATTAGCTCTAGCTAAAAAAGCAGAATCATCAAGAGTACTGAAAGAGAGAGGTTGAGATCCTTTACCTTTGCCTGAAGAAGAATCCAAATTCCTTCGGGCTTTCAATTTTGTTGCCTTCTCCAACGAGTCTTGATCCGAAGTTGACGCATTCCTTTTACTAGCTCTCACTGGCGAAGCTGTCCCACTGTTTTGAACCCGAGACTTACTGAAAAGGCCAGAGTCGTCCTTGGTAGGCATCAGATCAGCCATCCGATGTGCGG

ATCCATCTGATGTCACCCCACTTGGAGGAAGTATTATCATAGGTTTTTTACTAGACTTCACTGCTGCTGAGTTGGAACCGGGCTGCTGTCCACTTGACACGCCTGGACCAGGTGGCAGCTGATCAGAAGCATTAGCGTTATCAGTAGATGGATTCTCAAACTTCTTGCCATCTTCATCCATATTTTCAGGCTCTTTATCCTCTGCAGGTTTATCTTCATCCATATTAGTATCCATATCAATTACCTCTGGCTCACCGACGGTCATATCCGTCTCAACACGAAATTGTAGCTCATAAACAAAATCCCCAATAACTATATCCACTAGGTCTGGGATAAGATCAGGGTCAAGTACGGCTACTTTTATTCTTGACCTTCCAAATTTCTTTGTGAATTTCATGTCAACGGCACGAGATACACCTAAAATTGAGCCAATTGCCCAAAGAATAGGAAATTCTCGCAGTTCTTTTGGTAAGCCACGAAACTGAACCCAAACCTTGTCAATCTCATATTTATAAACTTCATTTTCAACTCCTTTTTCAAAACGAATTTTCCCTTTCACTGTCTTTGTATCCATAGGACCCCAGTTGACCATGTGATTCAACAATTCAGATGATGGGAAATTAGTGATAAAGACATTTGTGCCCTTCTCCTCAATTACCCACTTGTTTTTGCCTGGCAGAAGTTTTTCAAGTTCCACTGCCAATTGATCAGCAGTAAGAGAACCCTCCAAAACACGCACCATGGCTGATTTTTCCTCGGAATTTATCTTTGGATTTTCAGCGTGTGGAATGAAATAAAAGCCCAAACCTTCTACAGCATAACCACACGGAATTGCAGTAGTTTGCATGTTCTTCAAATTCGGACAAACCTTTGTAACATGGTCTCCAAAGCAAATATCACAGCTAAGAGTTGCAGTGCAAACAGATAAAGTGTGACCTTTAGTATGGCACCGATAACAATAAGGTTTACCCTTGCTTTTGTGTGCTCCCTGAAGAGATGACTCTGGCACTTCATTTTGCATTTTTGGGACAATTTTAATTTGTGGGACAGTAGCCAATGACATAGTTTCGGAGGTAGCTGGCACCGTAGTCGTTGTTGTCGTTGCTGTTGTCTGGAACGCATCAGTTCCCGCCACCTGAGTGTCGCCCTTGTGTCCTGTTGCCGGGGCGGCAGCAGAACCAAGCCCAGACTGTGTGCCTACTGCCGATGTCTCGGCCTGCACATTTGCAGCCGAAGCCCCCGTGAGCGTCCCTCCTGCCGAGGTCACCAGAGACGAACCAGGAGGTGCACCGTGACCCGCATGGTTCCCAGATGGAGTTGCGCCATGTCCCGCCTGGTTCACCACCACCTTTGCCTCGTGCTGACGCATCCTCGCCTCGAATGCACTTATTTATTGCCATTGCTATTTCTTATAATTACATATTCTTTTTTTCTACTAATTCAATCTGCAAAAGCATATCTAGCAGTTAATAATGCACTTGCCTTGATGAATTTGCACTGTCAGGGATCAAGTGTTGTTCACCAAACCCAGTCCTTAACGAAGGATAGTCAGGGGTTAAATGTTCATGCTAACCCAACTGCGACCAAGCATGAAACTGAGGAAAATCTAGTTAAACCTAAGACATGTGATTCCATGTTTGATGAACAGAGCGAGGAAATCCAAAATGGGGAAGATTCTTCAGCTCCAGACACTGGCACCG

CCGATGATGGATATTTTTTGAGGGTAAACAGGAGGGGCATGCCCCTACTGGATGATGGATATAACTGGAGAAAATATGGAGAAAAGCAAGTGAAAAAAAGTGAACACCCAAGGAGCTACTACAAATGCACTCACCCAAAATGTCCTGTCAAGAAAATGGTGGAGCGTTCTCTAGAAGGTCATATAACGGAGATAGTCTACAGAGGTTCTCATAGTCACCCACTGCCTCTTCCCAACAGCCGGCCAAGTGTCCCTTTGTCGCATTTCAATGATTCAGAAGCTGATGGTAACTTCAGTTCCAAGCCTGGGCCTGGCTATGACTCGTCAACTTCACAGGGAATTGCTCCAAAAGGTCAGTTCCAAGATGTGCACAGCGGAGCTCTTGAAACAAAACTGTCTGGCTCTCTTACTACAACTGAGATTGCTGACACGTCTGTTATGGAGTCTATGGATGTCTCATCAACACTCTCCTCCAATGAGAAGGGTGATAGGGCCATGAATGGTGCCGTTCCTTCAACCAATGACATGAACGAAGACGAGACTGAATCTAAAAGAAGGTTTGGACCTTGTTACCAACCAAGTACATAATTATAACTACCATACTAGTCAACGAATTCTCATTTGTTCCTTTGACTCTTCTCCAGGAAAATGGAGGTTTCTGTCGCTAGTAACACTGCCAACATTGTCACTGATATGGCAGCTATGGCATCAAGGACTGCCCGGGAGCCTCGGATTGTTGTGCAAACAACAAGTGAGGTCGACATCCTTGACGATGGCTACCGCTGGCGCAAGTATGGACAGAAAGTTGTGAAAGGCAACCCAAACCCAAGGTCAGTCTTGGTGCCATTTCCGTCTCGCCCATCCTTTTATCAGGAAACCAAATGGAAATTTAGCCATTTCAATTGTTCAATACACACCCTTCTATTTGCATGCAGGAGCTACTACAAATGCACTTATGCTGGCTGCTCAGTGCGCAAACATGTGGAGAGGGCCTCAAATGATCTCAAGTCCGTGATCACAACATATGAGGGGAGACACAACCATGAAGTTCCAGCTGCCAGGAACAGCAATGGGCATCCGAGCTATGGCTCCAGTGCTGCACCACAGGGTAGCAGTCTTCACCGGAGGCCAGAGCCTCCACAATTCAGCATGCCTCACGCTGCTGCCGCCGCTGCCTACGGTTCACTTTGTCTCCCACCACAACTCAATGCAGCTTCAGGGGGTTTCTCCTTCGGAATGCTCCCCCCTGCAATGGCAATGGCAATTCCAGTACCATCTCTGGGTAACTTCATGCCAGCGCAGATGCCTGGCCATGGATCACCAATGCAGGGTTGTTCAGGCCTTATGCTGCCGAGAGGTGAGGAGAAGGTGAACCCAGAGCAGCAGTCCAGATTGCCGGTAGCGAATGGGAATGCGGCAGCAACTTACCAGCAGCTCATGGGAAGGTGGCCTCAGGGTCATCAGATGTAAATACTCCAGCAGGCACAAAGATGAAACGATAGGGTTTGTTTTTTGCTTGTGTACATACACAATGAAATTAGGTTTCTTCTACCGTAGCTCCTTTGTTTGCTTTCAGGCTTTAGTAAGATGATTCTGATAATAGTGAGGGAGAGGGAAAGGGGAAGGGATGAACTTGGGTGATCAGGCTTTGCTGTTGTGTTCATTGAATTATTCAGACTAAGGTCCTGGAGTAGTAACTAG

CTTCAGCGGGTGTTTAGTTCCCCATAAAATACAAAATATCAACTATTTTGGAATTATGGAATGCAAAATGTCAAAATTTTC

>SbWRKY59

CGTTTCTCTCTCCACACCCTCCCGTAGTCCCGTCTCGAGTCCCAACGGATCCTTCCGCACATCCACCCTTCTCTTGGTCAAAGCGCTCTCCTCCTCCTACGCCCTCACCCTGCTCTCTCTCTCTCTCTCTCTCTCTCTCTCTCTCTCTCTCTCTCTCTCTCTGCTTCTGTTCCTTCCTCCGCCACCCTTTTATTTTTTACTCTTCTCTCTCCAGAGTAAAACAATTGTGCTGGTGAGGACGATGAGAGAGAAAGAGAGAGAGGGAGCCGCCGAGTAATGGCAGGAGGAACCTTCTTTTCCTGACTCCCTTACCTGTGGCTGCACCCCATCATTTGTCCCTGATCCTTGTGCTTCGCTCCTGTTGCGCTTTTCTTGGGCCATCGGTGGACTGAGCCGTCGCCAATAAGGAAGGAGAAGGCTTCCCCATTCCCACAACCGGGGGTGGAGCCGGAGCGCCTCCTCGCCACTGGCCTCCTCCGGCGATCTCTTCTTCCTCCAGTCACAGGTTCGTCGTGTTCCATCCCCATTCCATCCCCCTCGGTGGCTCTGAAAACTCTGTTTCTCGGCCTGCCATTTGTCGCGACGGGATTTGGCTGTATCCTATGGAGCCCCCTCGCGTGTTTTCTTGTTGTGCGTGGCCTGAATTTGTCCGTTCTTATGAGGAAGATCCTTCTGCCATTCCATTCCGTCCCGATGTGTTTTGTTTCCGCGATTTGGCCATTTTTCGTGCTGACGGTTCACTCCGTTTCGCGTAAAGGCGGAAGGTTTGCGCCGATCGACCAGTTGGGGAATGGCATTGGATATTGGTTTTCCCTGTCTGATCTTTGGTTGGTTTTCCCTCTTCCTCCATCCCTGTGTTTCTCGCGGAATCACTGCCGCATCCATCTCCTGCGGATTTCGCAATGAAATGCTCTGGCGAAGGCTGAGCCGGGCCATTCAATTGTAATCTTCTACGCCGTTTTGGGTCATCAGCATGTTCTTGGATGTTTCTGAATTTCTTTTTCCTCATCACGCCTGGTGTTGTCTTCGAGTAGATGTTTCATCATTGTCATCATAAAAAAAAATCTGAATCTTCTTACCTGAAATAGGAGTATCATAAGGGAAAGCTGTTCCCTGTTGTTATGCGTATACTCCGTATGCCGGTATGCGCTTTGAGCTCCGAGCCACAGCCCACAGGCAGAGGCAAAGGAAAGCACGCAACGATTGGGGTCCATGCCATTTCTTTTTCCTGCTCTTGAAGTGAATGAGATGGAACAGCCACGTTCCATTTTGTATTTCTTTTTGGTTTTCTATTCATTCATTCGTGGGGGAGTGTGCCGGTCGCATCGACCTAACCTTTCGTGTTACACCACTATTAAATAAATTCCTTAAACAAACACCGCTATCATGAGTTGTCGTTTACTGACATTGACGCGGTTTTGGTTTTGGAATCAGCCTCACAGAGAATTTTTGAGTTTGTGAAGACAAGCTCCAAGTCTGCGATTGATATTTAGAATAGGAAAATGGAGTCTCTGATTGAAAATACTCCTATATAAGTTGCTGTTCTTCCAGTTGGAGAAGACTGCCATCTTTTGAGAAATCTGTGGTTGTTGATGCATTTGGATTATGGTGCTTACTCATGCGAACCTAACAAATTTCCATCTATTGTTTCCTTTTTTTTTTCAATTGGACAATGTTTCTGCATATCTGTTCACCAAAAC

TATTATCACTACCGTTGTCAGATTCCTGTTGTCTCTGAATATTGTCATTGTCTACCTCTGTAACATTGAAGCTAATGTTTAGCCAAAACATTTACTACTGCTGAGATATAACTATTTTTCACCCCACTAGGTTGATCAATCCAAGCTGAGATGGAAGGCCATGTAGCAATGGAGTGGAAGGATCCTAAACCAGGCCCAGAATCCTTGATGGGCTTCCAAACCAGAGGTGTTCGACCAGACACCGTAGGGGGGCACAGCAACGAGGACGCTAAACCCGGATTTGAGAAGCATGGCTTCTCAGTCGACATAAGCTCGCCTCAGGAGGAAGGTCGGTCACTTCCACTCACCCCGCAGTTTGGCCAGAAGACCAGTCCTGGCAGCAGCCTCGCCGAGAGGATGCAGGCAAGAGCTGGATTCAAGGTGCCGAAGCTCAACATGCCTTTCAGCACAGCAGCTGGAGCTGATAATTCAGTACCAGGAGCCCCATCGCCCTACCTCACGATCCCGCCTGGCTTGAGCCCAGCAACGCTGCTCGAGTCACCAGTCTTCGTTTCTAATGCCATGGTATGTGCTGTTCTCTAGGTTATGATAGTGTGTGAACGCGTACCAGAATTACTATTGCTTTCGCCTATAGATGCTAAACTGGCAGTTCAAGAATGCTTGCTTTATTGAACTTTTGACCTCTTCGGGGGAGTTCGTGAAAGCTCTACTTTGGTTCCATGATCAGTTCTGCTTTGTTTTCTAGCATCCAGTCTCTTCTTTCCAAACATGTTTCTGTTTGTTTTCTCAAAAGCAGAAAATTGGTATGATATCATTCAAATATTTCAAAGTTTCCTCGAAAGCAGAAATATGATGACCAATCTCTTTTATTTCCATAGTAATAGAAGTAGCAATGTACACCCCACATTTCAGATCCAAACTTTTCTCCCATGTACTACATGGAAATCCAATATAATAGCTCTTAACGTTGGCGAAGCTGGGATAGGAAATTCACGTGTTTGCTCTGTTCACATTCATATATGTAATTGGACTAAAATGTGTACAATGTCTTCAGAGGTATCAACTAGGGAAATTATTTTTCTACACTCTGATACTGAAGCTATTTCTTACCTGACAACAATGTTTTTGGCTTCCTTGCAATTCTTCAGGGCCAACCCTCGCCAACCACGGGGAAGCTATTCATGTCTGGTAGCACAAATGACAATGATCCTATTAGATTTGGAGGTCCTCCAGTTGGAGATGGCCCTGATGCTTTTTCTTTCAAGCCCTTAGATCTGAAATCCTCACACTACACTGCTGAAGCAATGAAGGTTAGTAACATTTGAATGGTTCGTGAGTTATTCTTGATGAAATTTGCTTGCGCGCTAATGATAGAAGGCTAGTAACTTTTCGATAGTTCATAGGTTATTCCTGAAAAAAATTGCTTCCGTGCTAATGATAGAAGGTTAGTAACATCTGAATAGTTCATAGGTTACTCCTGATAACATTTGCTTGTGTGCTAATGATAGAAGATTAGTAACATTTGAATAATTCGTTGGTTACTCCTGATGACATTGGCTTGCGTGCTAATGATAGAAGGCTAGTAACATTTGGACAGTTCATAGTTTATTCCTGATATGACATTTGCTTCTGTGCTAATGATAAAAGTATCTGTAAACTTAGAAAGTATTTGCATGAACATGCAGTTGGCAGCCATATTT

GTTTCTGAAATTTTACCCGAAATATAGATGAGAAATCGGTTAAATCAAAAGAGTTTTGCTCAGGAAGTCTTTTATCTGTTACCCTACCTGTAACAGATTAGTTCATGTCTAATCTTTAGTAACACGAGTTATAGCAAAGTGGCTTGTAATGTCGGTACTGATGTTTTGCCCCATATTGTTCATAGTACTAATTGGAGAAAGACAAGGGACCAGCTAGATTCTGCTAGAGCTAGATAATTTATAAGTTCAATAAAATAATTCTCGCTGACAAAACTAAACACTACTTTAGTTGTCCTTCAGTTCAAACTAAACAGATAAGAAAATCATCCCGCTTTTATGTTTCAAAAATGTGAATGAACCATTTTTACTTTCCTTGTTCCTGATCCAATACTTGTTTGATTTCACTTCCTTACGAGTTAATTCATATTTGCTCCACCAGGAACAAAATACGCAGGTTTCTGTCAAGACTAAAACTAAGACTCAGCCAGTACAAGAAGCTAATTTGCTGGGCCAACTGAACCAGCAGAATCACAATGTGCAGACCAATATGAACATCGGCGGTCCTCATGACTCCAAGCTCAGCCGTCTTGCATCTGGCACCGGTGCCTGCAACGAACACGTATCACCCCCGGATTACGGCCAGACAGCTGAGGAAGGTGGTGATGCGAGGGAAGACTACCCGCCGGCAATGGCCGCCGCTACAGCGCCAGCAGAGGACGGGTACAGCTGGAGGAAGTACGGGCAGAAGCAGGTGAAGCACAGCGAGTACCCGCGGAGCTACTTCAAGTGCACGCATCCCAACTGCCAGGTGAAGAAGAAGGTGGAGCGCTCGCACGAGGGGCACATCACGGAGATCATCTACAAGGGCGCGCACAACCACCCGAAGCCCACGCCGAGCCGCCGGCCAGGCGTCCAGGTCCAGCCCGTGCACCCGTTTGGCGACGCCGGCGGTGCGCAGCAGGCGGACGCCGCGGCCGACAACAACCTGGGGTCACAGTCACAGCAGGCAAACGCTGCAGCGGAAGCCAACCACCAGCCGTGGCGTGCGGGCGTTCAGGATGGCATGGACGCCGCGACGTCGTCGCCCTCGGTTCCCGGTGAGCTCTGTGACTCGTCGGCGTCGATGCAGCAGGTCGAGTACGCACCCCGAGGGTTTGGGTCCCCCGAGGGCGCGGATGTGACGTCTGCTCCGTCTGACGAGGTGGACGGCGGCGACAGGGTGACTCTTGGCAGCATGTCCCACGCCGGTGCCGACGCGGAGGGCGACGAACTGGAATCTAAACGAAGGTCAGCAGTCCTGTGCATTGCTTGCTACTCATGCTCGATCCAGAGCTGACCGTCTGGTTATTTCAGGAAGGTGGAGGCATATGCCATGGATATGAGCACTGCGTCGAGAGCTATTCGCGAGCCTCGCGTGGTGATCCAGACGACGAGCGAGGTGGACATCCTCGACGACGGCTACCGCTGGCGCAAGTACGGGCAGAAGGTCGTCAAGGGCAACCCGAATCCAAGGTTTGTCTGAAGCAACAAAAGTTTTCTTTTTTCAGAGCTCGAAGATGAGTGGGCATTTGCTCAGCGTTTACTGCTATGGGGTCTCACTTGGTGGTGTGTCGCGTTCGTTCAGGAGCTACTACAAGTGCACGCACCCGGGGTGCACGGTGCGGAAGCACGTGGAGCGCGCGTCGCACGACCTCAAGTCC

GTGATCACCACGTACGAGGGCAAGCACAACCACGAGGTCCCCGCGGCGAGGAACAGCGGCGGCCACCCGAGCACCGCGGCGGCGGCGACAGGCGCGGGTGGTGGTGGTCCGCGGAGGCCGGAGCACACGTCGTCGGTGCACGACGGCCTGATGATGAGGCACCTGGGCGGCTGCGGCGTGCCGTTCGGCCTGCCGCTGCAGCCGCCTAGCAGGGACCCGCTGGCGCCGATGGGCAACTACCCGACGTACCCGTTCACTGCCCTCGGCGGCGGCGGTGGCAGCGGCGGCGACGGCGGCCTCACGTCGCTGCCGAGCTTGCCGATGGCCACGGGGAACCTCAGCGCCGTGGAGGGGCTGAAGCTCCCGATGCTGGCAACCTCGTCGCCCCTGCACCACCAGCACCCGCTGCTGAGGCACCGGCAGGCGATGCAGGCCGCCGCCCTCGCCGCGGCGCCCATGGCGCAAGTGAAGGTCGAAGACAACGTCGCCGCCGGCGTTACCGCTGCCCCGTCGGTGTACCAGCAGATGGTGCGCAGCGGGCTGCGGCTGGGCCACCAGATGTAGATAATAAAAGCGGGGACGATTAGAGTGGGTCTCAAGTTATTAGTTTCAAATTCTTATTACCAGTCGTATTAGGATGAGTTCGTCATAAGTATAATAGCGTGGAAACAAACGCCCCTTCGATTTGTTTTTGTTTTTAACCTTCCGTTCGGCTTCATTCTTTTCGCCAGCAACAATTTATTTACTGCCCCTGTTCATTTGAGATTCAGTTTTTGTTCAGCAGCTGGCATCTGGTCATTTGGTATTCAATGTTTATTATTTGAGAAATGACCAAGAGTTTCTGGGCTTTGAACTTGGCATCAGCCCAGAAAGCACTTTAT

>SbWRKY20

ACGCTGCCCCCGCCACCAGACGGGGCCACTTTCCCCAAACTGCCCTTCCCACCACCCCCTCCAAAACCACCACCTAGCTCCGCTCCCTGTCTTCGTCCCCCTTCACTCTCCGCTCCCCACATCCCGGCACCGCCAATGGCCGATTCGCCAAACCCTAGCTCCGGGGACCTCCCCGCCGGCGCTGGGGGCTCGACGGAGAAGCCGGTCCTGGCGGATCGACGTGTGGCTGCGCTCGCCGGCGCCGGCGCCAGGTACAAGGCCATGTCCCCCGCTCGGCTGCCGATCTCGCGGGAGCCGTGCCTCACCATCCCCGCCGGCTTCAGCCCCGGCGCTCTCCTCGAGTCCCCCGTCCTCCTCAACAACTTCAAGGTAATTTACCGCGCCTCCCTGTAACTGTCGCCCGCGCCACTCACTGCTCCCGCGGGATCTGCGAATTCCCGGGGCCTCCATGTCACGCCGAGGTTGATTCGGGACGGGGGAAAGAGGGATTCCGATGCGCATTAGCTTTATGGCCTAGTTTATGAGACATTTGCGCCGGTGGGATTGGCTTGTGGCGCCGGAATATCGCGCTTTTGCCCCGCGCTTTCGCATTTCCGGTGGTGATATGCGGCTTCTGGAGCTGCCTTTCCCGCTGGGTTCTCGGGGATATGGTTGTTTTTGCCCGGCTCCTCCTTTTGGGGCGCCCCTGCGCCATCTATTTGGTGCGGGGTGATGGGGAGAGCTGTTGCTTGTGCGTCTCGGGGAATTATTCTGTCCGGCACACACTTGGTGGCTCAGAGAACAAATCATCGCTTGGCATTTCTCTTGTGGGGGCTTTAAAGGGCCTTATTTAAGGAAATGTCTCCTCAGCCTCGGGTGCTATGGTACTGGTCTCGAGTGCTAAGGGATGGTACACTTCCATAAAGAAAACACCAAACTGCTGGGGGAAGGAAAGAGCGAATTTTGCAGGCTTGCTTGGACTATGGATGACCATTTCGTTGCTATTGTTTCTTGAGATTGGTTTAAGTGCCGTTTCTGGTTTTCATTCTTCTTTCTGACTACCCACAACCCAAAATAAGTGAGGATAGGATGCTTGATTACAGATTCATAATTATTGCTCTGTTCCAGTTTGTTGTTACTGTTGACATTCTGTTCTGTTCATTAGATGCTTGGCAAATATATTCTTCTGACTAAAGTAAAAATTAATGAAGTTAGTATTCATTTCAGATTTTACTGGTGGTTCTAATAAACCTTTAAGGGCTTAACTAATACAATATTGTCATAACAGAAACACTATGAACAAGTTGTGTGAACGTTGTTCTACACAATTTGATATTCAATTATTCATATAGATATAGTTTATCCTGCTGGACAGATAATCAATAAGCATTTGTTTCTATTTAGGTTGAACCGTCTCCCACAACTGGTACTCTGAGCATGGCTGCAATCATCAACAAGAGCACTCATCGAGACATACTGCCTTCGCCTAGGGATAATTCAGCTGGTAGTGGCCAGGAAGATGGAGGCTCTCGGGATTTTGAATTCAAGCCTCATCTCAATTCTCAATTGGCGGTATGGAACCATTATTGGAATGATTATTTTTGTAATTCTCTAGCACCATAGAACACACATCTTGAGGGTCTTGTTTATATGTGAGCTACCCCAAAAAAAAACTTTATCTTGTATAGCAGCAAAGGGAGTTTAAGCATTTTGTTTGTATG

ATATCACTAAAATATAATGTAGAAATTATCTTTAAGCTCTGAGGAGGGAAGTTATATTTCTGATGTACTTTACACCACTTCCTTTTAGCAGCATGATGCTCACTTATTGTTCAACCCGTATCTTATTAGTATTCAGAGATACAGCTAATTGGTTTCAGTGTTTTACATTTTAGATAGGATCATAGGAGCTATATATTGCATTCTGATTCATGGAATCATTTTTTTTTTGGTTCTTGTTTCGTATGAAAAAAAACACCCTCCTTTCTACTTTGTTCTACTACCTCTGTTCCGTAATAACAGGTGTATTCTCGTTTTTCATTTTGTCCATAATACTGTGTGGGCTGGAGTATCGAGGGAGACACTTAATTAGACCACTCAAAACTGAAACGTCTCATGCATGCGGGTATCCCAGCGCCGAAACGTTACATGCATTAATGGCACTTTTGGTCCATTAAATGAAGATGTGGATGCAAACGTGGCCCGTATACTGCTGGGCAGAAACGGAACGACGGTTAGCCTTCGTGCCCGGGGACACAAACGAGTATGCGAGTTCATACAATTTGCATGTGATTGAAGTTTGTCCTTGGTATCTGCAAACAGACAGCCTTGCCACGACTGGTAAAAGGGAACGGAGGGAGTAGCACTTATGTGCATGTTTCTTTGACTAAAATAGTAATTACCAATTTACCAACACCTTGATCTGTTACCGTTCTTTAATAGCTGAGTCATAACCGGTTACCTAGTGCCCCCCCCCCCCCCCCCCCCCCCCCGGGCTGTTTATTTTGGTAGTTCCTGAGTGAATTCTGGCATGAAGTTAATTTGAACTCTTGACTGTTATATTGGAATCCGACTACCCCAGTTTTGTTGGGTCTAGTCATGTTGTCACTAGTCTAAGTTTTTCTTCTTGGATTTGCTAGAAATAAAAAGAAGGTGACACTGTTTATTATCATGGTTTGGGAAAAAAATCCCTTTTGTGCCTTTGTGTATACCACAATTTCAAGTCTGAATGAGTTATGGTTCTGTTACATTTTGGCTTGATAACCTATAAAATGCTACACTGGAAAATGGATTGATTTAGGAATATATGTTATGATTTAGTGTTAGGACAGTTCATAGTGTGCTTACACATTTACAACAAGCCTGTGAAATTAGAATATAAATAGGAAAATCTGCAGGAATTTCATAGGAATAGTCCACGACTGAAGTGGTGTGGATGTTTCCTTATAAAATTTCCATGGCATTCCTGTTGCAGTGCTGAAAGGATTACTCTTTTTGTGGCTTCAGTTATTATCAACAAAATAGGGTGTTCTAACTTCAACATCGAATGAAATTGATATACGTATATTTTGTGCTGGGTCTTAACTAGTCTATTCTCATATGTGTAACAGTTTTCTTTTTATATAACTGATTAGTTTTTATGGAAAAAACTTCAGTATTTATTGTGATATACCCATATGGGGCTTCTGCGTTCTTATTCTTGTTGACTTACACTGCAAATAGTTTTGTATTCCATGACATTCATAGTGCTCACATGCAGGCTCCTGCGGTCAACAATCAAAATCGTCATGACACCCCTATGCAAAATCATAGCTCAAATCATGCCTCACCATCTAGCAATTTGATGACTGAAAATAAACCTCTCTGCTCACGGGAATCGAGTCACACAGCAAATGTTTCAAGTGCTCCAAACCAACCTGTTT

CAATAGTTTGTCCATCTGACAATATGCCTGCTGAAGTTGGTACATCAGAGATGCACCAGATAAATAGCTCTGAAAATGCTGCCCAGGAGGCACAAACTGAAAATGTAGCTGAAAAATCAGCAGAGGATGGCTATAATTGGCGCAAATATGGACAGAAGCATGTCAAGGGAAGTGAAAATCCTAGAAGTTATTACAAGTGCACGCATCCTAATTGTGAAGTTAAAAAGCTATTAGAGCGTTCGCTTGATGGTCAGATTACTGAAGTTGTTTATAAAGGGCGTCATAATCATCCTAAGCCCCAACCGAATAGAAGGTTAGCTGCTGGTGCAGTTCCTTCAAGCCAAGGTGAAGAAAGATATGATGGTGTGGCACCTATTGAAGGTGAGTGTGCCATGTTTTTCTGTAATTACATTTTTCCATTGCATTGCAATTTCGACTTGAGCATACATATATCTAGTTTGCATTATGAAGTATTTTCTTGCTCATTTTGCATGTCTGTTTCAATTGCTTTTGTAACAACTTTAGTTCCTGAGTCTTCCAAAATCTTGAGAAGGAATGATTTGAACATACAATGATCATTTGATACTAAGTTTCTTAATGTTTACCTGTGACATTTTGTTGGGTCTGAGAATTCCTGTTAATATGCAGACAAGCCTTCAAATATTTATTCCAACCTCTGTAACCAAGTACATTCAGCTGGCATGATTGATACTGTTCCGGGTCCAGCTAGTGATGATGATGTTGATGCTGGAGGTGGAAGGCCCTACCCTGGGGATGACGCTAACGATGATGATGATTTGGACTCGAAACGCAGGTAAGACATGGTTTGTACTTTGGATTCAATATGCCCTAAAGGCTCTCCCCTGTCAATTGCTAATCCTGTACTCCACAACTGTGTGACTAGGAAAATGGAATCTGCTGGTATCGACGCTGCTTTGATGGGTAAACCGAATCGCGAGCCCCGTGTTGTTGTACAAACTGTTAGTGAAGTTGATATCTTGGATGATGGGTATCGCTGGCGCAAATATGGGCAGAAAGTAGTGAAAGGAAACCCCAACCCACGGTCAGTACTCATTTTGTTCATTATTTTTGCCACTATTATGTTTTCCTTCCTTTTGTGTTTGACATTCCAAGAGAATTTGAACCATTATCGGATAAAGTCGGGTTACATTATTTGGACTATAAACTAAGAAATGTTTGTAATCAGCATATCTTATTGCAATTGATCATTTGCATACCTATTGATTTTTGGCCCTTTCTAAAAAGAATTGTTATTACTATACTGGTTACTTGTGCCTTCTAGAAACTTCAGTCCAGAGTGGAAATGACCAAGAAACAACAAAATTTTCTACTTTAACTCTGATAAGCTGAAAGGTTGGCTTTCAACAAATTAGGAGACAGTTCTGGTAAAGATGTATGGTGGAAAGATGCAAAAACTGCTATGGACATCTATCTTAAATATGGAGAAGGAAGCCTTCTTATTAAGATCTGTCCATGTGTCCCTTCAAGCCTTCTTATTATTCTGTTGATTTGTGCTGCCATTTTGTACTGTTTGGCACCTGATTTGGGCAGATACTAGCTAATACTATTATGATGTGTTCTATTCCCTTCGCATAGATTGCATCTAACTGCACAAGAATTGAAGTTATGAGATATATGTAAAGTGTATAAGGAATATGAGGTGCTTCACATTTTGTTTA

TACCATCATACAAGTATCTGTTGATTTATTTATATACCCTAGCCTATCACCCTACCCTGCAAAAGTACATTTGATGTAGAAAATAAGCATTGTCTTGCACTGTTTTCCAAATATATGTAGTTTGCATAAGCAAAGTGTATGTTTTTAATTAATGGCATGAAGTCAAGGCAATCGGTTGGTTGGGATTTGTCTTCTACGCTTGGTTTGAAGCCTTTCATGTAGTTTAATTTTGCTGGGAAGTAAAACTTGAAAGAACTTACTCTGAATTGGGTTGCTAGCTAGGCATTTGGACTAATTCGTGTTGGTTTGATACCTACGGTTGCCAAAATTTTGATGGCACTGTCCTGGTGATAAGATTGGCAGGCCTCTAGTTTTCTGCACTTTCTTTTTTAAAAAAGACTTATTAGGCAGTGCATTCATATGGAAAAACATATTCACTTCTGTTCTTGATTGCAGGAGTTACTACAAATGCACACATACAGGATGCCCGGTCAGGAAGCATGTTGAGAGAGCATCACATGACCCGAAGTCAGTGATCACAACATATGAAGGAAAACATAACCATGAAGTCCCTGCTTCCAGGAATGCAAGCCATGAGATGTCCACAGCTCCCATGAAGCCTGTGGTGCATCCTATTAACAGCAACATGCCAGGCCTTGGTGGCATGATGAGAGCATGTGATGCCAGGGCCTTCACCAATCAATATTCTCAGGCAGCTGAAAGTGACACCATCAGCCTTGACCTTGGTGTAGGTATCAGCCCTAACCACAGCGATGCAACAAACCAAATGCAGCCCTCAGTTCCAGAACCTATGCAGTATCAAATGCAACACATGGCTCCTGTTTATGGTAGCATGGGACTTCCAGGAATGCCTGTGGCAGCAGTACCTGGAAATGCAGCTAGCAGTATTTACGGTTCCAGAGACGAAAAAGGAAATGAAGGGTTTACTTTCAAAGCCACACCTTTGGACCGATCAGCTAACTTTTACAGTAGTGCTGGTAACTTAGTGATGGGTCCATGAGTGTCTTGCTGATGGTTATACCTCCATGAATCACATTACTGTCGTCATGAAAATCTCTTCAGAAGATGCATCCGCACACAATTGGAGGCGGTCAAGGTGTACCTCCGGAGCTGCAGCGATGACACATGATGTCTTTGCTGCTTGGATGTACTCGCTGTATTGAACGCTGCAGCTCAACATTCGTTGTACAGCAAAACCAGTTATGATTAATTAGATTATGATAATTTGGTTATGTAAACTTCTTTCTGTACTGGAATATGGGATAGAACCAAAGAGCCATCTGGTGGCAAAGCTTTGTTATCTCCTGCATATGAACGATGCTAGTTTGATATTCATATATGAAATGAAATATATCATT

>SbWRKY42

CGGCAGCTCCGAATTACCTCCTCCTCTCCCACCTCATCATCGCCTTCCTCCACGGTGCCGGCCAGCCAGGACGTACACGCCGGACACGACGTGACAGCCACCCCGAACACACACACACACACACACACACACGACCGCCGGTCTCGTCGCAGCGCAGAGAAGCCGCAGGTTTGATCGTTCGCCGATGACCACCTCGTCGTCAGGGAGCATCGAAGCACCGGCGGCGAGCTCGAGGCCCGGCTCGTTCTCGTTCGCGAGCACGAGCTTCACGGACATGCTGGGAGGATCCGCGGATGCGGCGGCCGGCGGGGCGTCGAGGTACAAGGCCATGACCCCGCCGTCCCTGCCGCTGACGCCGTCGTCCTTCTTCAGCAACATCCCCGGCGGCCTCAACCCCGCCGACTTCCTCGACTCGCCGGCCCTCTTGAGCTCCAGTGTAAGCAAGCACCGACGAACTCCGTGCAGCGGCGCGCACTGCCGTCCACAAGTTCCTGGAATGCTAATGCGCTTTTGCACGTTTCAGATCTTCCCCTCGCCGACGACGAACGCATTCGCGTCGCAGCAGTTCAGCTGGCTGACGACGCCGGGCGCGGAGCAAGGCGTCAAGGAGGAGCAGAGGCAGTCCTACCCGGACTTCTCGTTCCAGACAGCGCCGACGACCCAAGAGGCCGTGCGGACGACGACGACCTTCCAGCCACCGATTCCAGCGGCCCCACTGGTGAGATCAGCATCCATCATCTGTGTCATCACCCTCTCGTGGGCTCTTTGCTGACTTTTTTGTCCAAAAGAGACAACTACACCACTAAAAAAAAAACAGAACTGCTGTCCGGCATAGTATCCTAGACTATTTTCTTTAATAAGCACACAAATATAAAATAACACTTGTGGTTAGCATCATGTTATGATCATGATCTTTTAATCTTTTGTTTGTACTGTAATTGTCAATGATAAAATTATCCTAATATATCCCTTGCAACACTTCAGGGTGAAGAGGCGTATAGAAGTCAGCAGCAGCAGCAGCAGCCATGGGGCTACCAGCAGCAGCAGCAGCAGCCTGCAGGCATGGACGCGGGATCCAGCCAGGCTGCCTACGGCGGGGCGTTCCAGGCAGGCTCGTCGGACGCCGGCGCGATGGCGCCGCACGTGCCGGCGAGCGGCGGGTACAGCCACCAGGCGCAGAGGCGGTCGTCGGACGACGGGTACAACTGGCGCAAGTACGGGCAGAAGCAGGTGAAGGGGAGCGAGAACCCGCGCAGCTACTACAAGTGCACCTTCCCGAGCTGCCCCACCAAGAAGAAGGTGGAGCGGTCGCTGGACGGCCAGATCACCGAGATCGTGTACAAGGGCACGCACAACCACGCCAAGCCGCAGAACACGCGCAGGAACTCCGGCGCCGCCGCGCAGCTGCTGCAGGGCGGCGACGCGTCCGAGCACTCGTTCGGCGGCACGCCCGTCGCCACGCCCGAGAACTCCTCGGCGTCGTTCGGGGACGACGAGGTCGGCGTGGGCTCGCCGCGCGCCGCCAACGCCGCCGGCGACGAGTTCGACGAGGACGAGCCGGATTCCAAGAGATGGTGAGTGCTTTGATGATGACACAGTTTTCCTTTTGCGCTAAGTTGCATGCTTGGAACAATCGAACAGGTGGAATTGAATTCCTGTTGTTTTTTGTCAGGAGGAAAGACGGTGACGGCGAGGGTATC

TCCATGGCCGGCAACCGGACGGTGCGTGAGCCGAGGGTCGTTGTCCAGACCATGAGCGACATCGACATCCTCGATGACGGCTATCGGTGGAGGAAGTACGGGCAGAAGGTGGTGAAGGGAAATCCAAACCCAAGGTAAAAAAAAAAAAATCCCTCTCCGTGGAAAAGAATTCAAAGCTTACTGCAAGTGCAAGTGTGAACTTTTTTTGCTCATGGTGAATGTTTTTCTTGTGACTGCAGGAGCTACTACAAGTGCACGACGGCCGGGTGCCCGGTGCGCAAGCATGTGGAGCGCGCGTCGCACGACCTGCGCGCCGTGATCACCACGTACGAGGGCAAGCACAACCACGACGTGCCCGCGGCGCGTGGCAGCGCCGCACTCTACCGGCCCGCGCCTCCGCCGCCGCCGTCCGCGGACAACGCCGGCCACTACCTCGCGGCGCAGCCGGGCATGGCGTACCAGACGGGGCAGCAGCAGTACGGGTTCGGCGGTCAGGGCTCGTTCGGCCTCAGCGGCGGCGCCGGCGCGCCGGCGCAGAGCAGCGGCAGCTTCGCGTTCTCCTCCGCCGGGTTCGACAACCCGATGGGTTCGTACATGAGCCAGCACCAGCAGCAGCAGAGGCAGAACGACGCCATGCACGCGTCGAGGGCCAAGGAGGAGCCCCGTGACGACATGTCGTTTTTCCCGCAGTCGATGCTCTACACTGACTGAAGGACTGCATCTGCAGAACATGGCAAGACTACATGACGTGATGTCCATAAAACTGTTTCTTTGCATAGGGTTGTTGTTGTTGTTGTGTCTAGGATAGGGTTGAACAAATTTCTTTATTTGTTTGGAGGCCCCCTCCATGTTGGCAGAGACATGCTTGCCGCAAGGGTGCCTCCCCTTAACAGGTGGAAGGGGAAACAAAGGATTTAGGATACTAGAGTCTAGGACACATGGCAATTAGACAAAAGGGTAAAGAAGTTTTGTTACTCATTATTTGTTTTCACACACATGAAAACTGCTGTAGTCAAAATTGAACTGAATTTTGTGTCATAGAGCATTTATCAAGATTCAAACTGGTCCGTCTC

>SbWRKY80

ACCGCCACTCCACCGCTGGCTCACGCCGTCACCGCGACCCGCACCGGCCAACCATGGGGGCCCACGGGCCTGCAGGACGCGCTCCCGTCCCGCCATTGGTGGGTGAGGAGGATCTTCGAGCGGGGGAAGTGTGGAATGGCCGCTGCGCAGCCGCACGCCAGTCAGACGCCCGGGGAGCGCTGACTGGTCAATGGTCAAACTTTCGAACGTCTATATAGGCCGCAGACGGGTCGGTCTCGACTCTCGACATCACTTCACCATCCTATACGCCGTCGCCCGCCCGGCTGCTTCTCGTTCCATTCGTCTTCGTCTTGCCTCCCTCCGGCAGCCTCCGACGACCCTCCTCTCCTCGCCTGCGATGACCTCGACGCCGGGGAGCTTCGGCGGAACGCTGGCGGCTAACTCTGGGCCGGTCGCGCTCTCGTTCCCGACCACCTCCTTCGCCAACTTCCTAGGCGGCGGTGGCTCCTCAGCTTCTAGCAGCGGAGCGGCGGACAACGGGGGAGTCGGGCTGTCCAAGTTCAAGGCCATGACCCCGCCTTCCCTCCCGCTGTCGTCGTCGCACCCGCCGGCGTCGCCGGCGTCGTACCTCCACGCCTTCTCCGGCATCCTCGACTCGCCGATCCTCCTCACTCCCAGCGTAAGTTAGCACGCACGGAGCCGCCGGCGTGCATGCATGAACGTGCGTTGTTGCGTCGAGCATGTTTGTTTGCCGCCTGTCAGTTGCTAACGCGAAGGTTTTGCTGATGTTTCAGCTATTCCCGTCGCCGACGACGGGCGCGATCCCGTCAGAGCCCTTCAACTGGATGGGGACGTCGGAGAGCCTAAGCGGCAGCGTAAAGACCGAGCAGCAGCAGTACACCGACTTCACGTTCCAGACGGCGGCGTCCGCACCGCCGGCGACGTCGACGTCGACGATGACCGGTGCCTCGCACTCGGCGTCCTATCTGCAGTCATCAGTGCTGATGGCGCCGTTGGTAAGCGATATGATTGACATCATGAACTAGTACTTCACGGTAGAATATCTGAATACAGAGGCAGCAAGCAAACTCCAGTCAAGAAAGTCCACGCACTCGTCTCTAGCAGTTTGCCTGTAGCTTGTAGCGCCTGTAGTACTGTAGTAGTACTAATGTGTGTCCTGTTTTAAGATGCTGACATGTTCTTTTTTTAACTTTATTTGGATAATTTGGACATCTCTCATGACGCCAGATGTTGATCTCATGCATGCATGCCCCCAACTGTTGCTTTCAATGATCACAAATCTACTATAGCACTAATTCACGATAGTCAATAATGTCGAGCGATTCTCAAGACCGAGTAGAGCAAAGTCCATTTTAATTCGAAAATCATTATATTGTTTAAATCATACGTATTTGATGTCTAAAAATAGCGCTGCACTGTTATATATATGTAGGGACGAGTAGGAGACTCGTACAACGGCGGCGAGTTGCAGCAGCAGCAGCAGCAGCCGCCATGGGCCTACCAGGAACCGTGTACGCAATTCGAGGCGCCGGCGGCGGCGCAGCCTGACAACAGCATGCTCGGGAACGGCGGCTACGGCGGGGCTCCCGGGCCGGCGGTATCCGGCTGCTTCCGCGAGCAGAGCCAGAGCAACCGGCCGTCGTCGGACGACGGGTACAACTGGCGCAAGTACGGGCAGAAGAATATGAAGGGGAGCGAGAACCCGCGCAGCTACTACA

AGTGCAGCTTCCCGGGCTGCCCCACCAAGAAGAAGGTGGAGCGGTCGCCGGACGGGCAGGTCACGGAGATCGTGTACAAGGGCGCGCACAACCACCCGAAGCCGCAGAGCACCCGCCGGAGCGCAAGCTCGGCGCCGGCGCCGGCGTCGCACGTGCTGCAGAGCGTCGGCGACGCCGTGCCCGAGCATTCCTTCGGCGCGCTGTCCGGCACACCCGTGGCGACGCCCGAGAACTCGTCGGGGTCATTCGGCGGCGACGACGAGATCAACGGCGTCAGCTCGCGGCTGGCCGGTAACTTCGCCGGCGCCGACGATCTCGACGACGATGAACCCGACTCCAAGAGATGGTGAGGCACCGAACTTTCGAACGAACACGCAGTCTGTTCAGTGCGTGTTGCAGCATGGGTGTTAACGTGTTACGGTTCCTTCCCAGGAGGAAAGATGGTGGCGACGGCGACGGAGGGGTCTCGTTGTCCGGCAACAACCGGACGGTGCGGGAGCCGAGGGTCGTCGTGCAAACGATGAGCGACATCGACGTCCTCGACGACGGCTACCGGTGGCGCAAGTACGGGCAGAAGGTCGTCAAGGGCAACCCGAACCCGAGGTACGTACTATACTGCTGGCTGCTGCGACGACGGAAGCGTTCGAGTTTCGACGCGTGTGAGCTCACATGCATGCATGCACGTCTTGCAGGAGCTACTACAAGTGCACGACTGCAGGGTGCCCAGTGCGGAAGCACGTGGAGCGTGCGTGCCACGACACGCGCGCGGTGGTCACCACGTACGAGGGCAAGCACAACCACGACGTGCCGCCGGCGCGCGGCAGCAGCGCCTCGCTCTACCACCGCGCCGCGCTGGCGGCGCATCAGATGCCGCAGCAGGCCGGCGGCGGGAGCTGCTACCAGCAGCAGCAGCAGCATGGCGGCCTCGTCCGGACCGCCGATGGGTTCGGCTTCGGGGCCAGCGGCGGCCTGCACGGCGGCGCGCCGATGATGCAGGCCGCGGAGAGCGGCTTCGCCTTGTCCGGGTTCGGCCACCCGGCGGGCACGGCGGCGTACTCTTACACGAGCCACCAGCAGCAGCAGACGACGACGACGAACGAGGCGATGTACTACGCCAAGGACGAGCCACGAGACGACATGTTTTTTGAGCAGCCGCTCCTGTTCTGACCGGATGAACCACACTCACTGCCGCATCGATTGGCAGGACATGCTCGCACTACTTGTGATTCTTTTTTGACTCGACACAGATTATGTACACAATCTATAGTGGTTTTGTTGATTTGTTTGGAGGCCCCAACTTTGCGGGCATCATCTGTAGACAGCCAGGGTAGCCTTCTGTAACATGTACAAGAAACGTAGGACACATCACATTATTGCTGTATAGTAGGAAGTAAGGCCATGGGGAAATGGTTTTTCAGCAGTTTGACTGCATTTTTTTTTCAAAAGGAAATTGTTGTAAGTGCTTAGATCTTAATCGATAAAGTGTTCAGTTCATGTATCTTAGTACAGTCAAAAAT

>SbWRKY79

ACTCCCGTCCGCCTCCGGCCCTCTCCCCCTCATCTCCTCTCCTCACGCTAGCTGTGACCCCATCTCTGCCCTCGTCTCCTCCACTGCTCGGCTGCTCTGCGTCGCTCGCCATGGCGTCCTCGACGGGGAGCTTGGAGCACGGCGGGTTCACGTTCACGCCGCCGCCCTTCATCACCTCGTTCACGGAGCTACTCTCCGGGACAGGGGACATGCTAGGAGGAGCCGGCGGCGCCGACCAGGAGCGGTCGCCGAGGGGGCTGTTCCACCGCGGCGCCAGGGGCGGCGTGGGCGTGCCCAAGTTCAAGTCCGCGCAGCCGCCCAGCCTGCCCATCTCATCGCCGCCGCCGATGTCGCCGTCCTCCTACTTCGCCATCCCGGCCGGCCTCAGCCCCGCCGAGCTGCTCGACTCGCCCGTCCTGCTCCACTCTTCCTCCAACATCTTGGCGTCTCCCACCACCGGCGCCATCCCGGCGCAGAGGTTCGACTGGAAGCAGGCCGCCGATCTCATCGCGTCTCAGTCTCATCAGCAAGACGACACCCGGGCTGCCGCCGCCGCCGGCGGCTTCAACGACTTCTCCTTCCACACGGCCACCACCTCCAACGCCATGCCCGCGCAGACCACGTCCTTCCCTTCCTTCAAGGTACGAAGAATTGCTTGTTACTAGCACAGGCTAATTAAGCTACGAAATAAGTATTTACCAGTACACTAGGCCAGTAGCTGAGAGTCTGAATCGATCTGTGTGTGGTCGTGCAGCAGGAACAGCAGCAGCAAGTCGAAGCGGCAGCGACGACCAATAAGCAGAGCGCCGTCGTGGCGTCGAGCAACAACAAGCAGGGGAGCAGCGGCGGCGGGAACAGCAGCAACACGAAGCTGGAGGACGGTTACAACTGGCGCAAGTACGGGCAGAAGCAGGTGAAAGGGAGCGAGAACCCGCGCAGCTACTACAAGTGCACGTACCACAGCTGCTCCATGAAGAAGAAGGTGGAGCGGTCCCTGGCCGACGGCCGCATCACGCAGATCGTCTACAAGGGCGCGCACAACCACCCCAAGCCGCTGTCCACGCGCCGCAACTCGTCCTCCGGTGGGGTCGTCGCGGCGGGGGAGGAGCAGCAGGCCGCCGCAAACAGCCTCTCCGCCGCCGCCGCCGCGGCGGGTGGCTGTGGGCCGGAGCACTCCGGCGCCACCGCCGAGAACTCGTCCGTCACCTTCGGCGACGACGAGGCGGAGAACGCGTCGCACCGGAGCGACGGCGACGAGCCCGACGCCAAGCGCTGGTACGAAATTCATTCGCTACTCATTCGCGGTTGTATATATGGTATTTTTCTTGTGTTGGTTCGTGGAACTCAAACTCATCATATATATATATATATATACATGTGCTTGATCGATCAGGAAGCAGGAGGATGGCGAGAACGAGGGCAGCTCTGGCGGCGCCGGCGGCAAGCCGGTGCGCGAGCCCCGGCTGGTGGTGCAGACGCTGAGCGACATCGACATCCTGGACGACGGGTTCCGGTGGCGCAAGTACGGGCAGAAAGTGGTGAAGGGGAACCCGAACCCGCGGAGCTACTACAAGTGCACCACGGTGGGTTGCCCCGTGCGGAAGCACGTGGAGCGCGCGTCCCACGACACGCGCGCCGTGATCACCACTTACGAGGGCAAGCACAACCACGACGTGCCCGTGGGCCGCGGCGCCGCGAGC

CGCGCGGCGGCGGCGGCGGCGGTGGCGCCGACGATGGGAGCCTTGATGGCCGCCGGCGGCCATCAGCAGCAGCAGCAGCCCTACACGCTGGAGATGCTGAGCGGCGGAGTAGGCGGTGGCGCCGCATACGGCGGCGGCTACGCGGCCAAGGACGAGCCGCGGGACGACCTGTTCGTCGACTCGCTCCTGTGCTAGTGAGGGGATATGCGGAGTCGTCGGTCCACACCAATCTTGGCGCGCGCGGCGGCGTCTGTAGCCTGCTGTACGTGTACGGCGGGTGTACGTATACACGGTGGCGTACACGCTCGCCTAGCTTCGCACTCAGATACGTACAGAATACACACAACACGTACGCTGTATACACTGGATCGTAGTAGGTGTATTTAGCTTAGTTTAGGAAATGGAAATTTGTTGATTCGTTGTTGGAGGCCTTTCTGCCACGCGCGCACTGCACGGTGCCCTTACATCTCGTATGCGGTATGCCTACGCATACGGTTCTG

TGTCTTTTTGTAGCGGCGGTGCCGCCTGTGTGAAAAGGCCATACGTATATCAAATATATAGTGAGACAAAGTCAATAAAGCTGAACAAAGATTTTGCGTTATTTCATCATCTCTTCTAGCAGTGATTGTGAATTACCAAAATAAAACAAAGAAGAGTAAAGTCCATGCATATGTCAAACGATACAAAAACCACTTTTTTGTTTTGTTCTTGGGCCGTGGTTAGTTCCTTATGAAAAAAATTTCGCGACACTGGAGCATTTTTGTTTGTTTATGGTAATTATTGTTCAACCATGGACTAACTAGGCTCAAAAGATTCGTGTCGTAAATTTTGACCAAACTGTGTAATTAAATTTTATTTTTATCTATATTTAATATTCTATGCATACGTCTAAAGATTCGATTTGATAGGAAATTTTAAAAAAATTTGGTTTTTTGGTTGAAACTAAACAAGGCCTTGATTCTATTTTTGAGTCGATGGAGGAATTCGATCACGGGATACCGAACTAGTACTACCAAGACCTTGTTTAGTTGGCAAAATTTTTAGTTTTTGGCTACTGTAACATTTTCGTTTTTATTTGACAAACATTGTCCAATCATGGAGTAAGCAGGCTCAAAAGATTCATCTCACAAATTACAGATAAACTGTGTAATTAGTTTTTATTTTCGTCTATATTTAATATTGTATACATACGATCAAAGATTCGATGTGATGTAGAATCTTAAAAAATTTTACGAACTAAATAAGACCCAAGTTAAGAAAAAAAAGATTGTTTGGGTTTTTGGTGACATGTGGCTACCATGCATTTTGATTTTATATATGTGACGTAAGAGGTGACATGCATGCTGGGCCGGGGAAGCTAGCTCAGCTGCTCATCCTTATCGCAATGAGCAGCGCAATAATCTATCTAATCTAGTACAGTAGTTGCATCGCTCGGGCACATTCGTTGAACCCAGGTGGAGCAGCTGGCACGTGCCTCATCAACTTGTGTATCAAGAAGGCGACGTGCGCGTCATCGCGTCTCCTCTCACCTTTTTAAAGTACAGTACAGGCTAGCTGTTGACGATCCATCACGCTAATAATAATCAATACGGTCGTGTTCCTACAAAATGAACACAGTTTACGGGCTGATTGATTGATTGTGCGTTGCCGCCTAACTGAACTCTTTTTGTAATATATATAAACTCTTTTTTATCTGTTCAGTATATATATGGCTATGTGCTAGCCTCAAGTGGAGACACGATCTCCTGCGTGGTTGCGTACCATGATTGGGTTGCCGACCTCCCAGCGCCCCAGCTCCATCTGTAGCATCTTCTTAGCTGGACGATGCACAACCGACAAATTATCGCGTTCTCACACTAGATTTTTGCTCAAAAGAGCATCGGTTGGTTTGTTCTATCAAGTGCGGCAGCCGCGGAGCGCGCACTTTAAGTTTCAGCGACGGCTAGGGGCGCGCGCACGGGCTCAAAAAAAAAAAACGCTCACTAGCGGAGCGCGGCGGCTAGGGTTCGTCGCCAATCATGGGGCGCCGGCCGCAAAAATTAAATGGACGGGACGAGCTAGGAACTTACAAGTTGTTTAGTTTAAGAAATTAAACCATTCTAGATGAGATTGTGTATTATGATTCATGAGCCAATTCCTCAGTTTTGGTAAATAACACCACTTCTCCCACCGTAAGTTTTGTATGCTTACCTAGTTATTA

TCTTGTTGGTAATTTTTGTGTGCTTCATAAATATGTTACGAAAAAATATAAAATGTTAGTGTTTTTATCACCAACTATAATTATGAGGTATGTGTTGGATGTTGAAAGTAGCATATATGACACAGCAGAGAGATTAAGGGCATGTTTGGATTCCCTTTCTCTAAACTTTAGAGCTCTAAAAGGTTTAGAGCATTTTAGCTCACTTTTGAGCTCTAAAGCTCTAATGTAAGGTGAGCTAAAGTTTAAAGTTCATTCTAGACTATTTGTTTAGAGCTTTAGCTCTAAGGTTTATATAACACTCCAAAATTTTTAATTTTGGATTGTTATTAGAAAACACTAAATTGAATAAGGATTTAAATTATTTTTTCTAAAATATTCCAAGATTCATTTAATTATTGAATTCATAAGGAGAGAAAAGTGTGGAATTTTAAAACTTATATACAAAAAAGTGAAGGGTGTTTTGTTTTGTTATTGTGTGCTTTGTGCCCTAGTTTTATTTGGTTTGTCTTAGAAAAATGAATTTCAAATTTTTCTAGCGAGCACGTTAGGTCCTTTCGATTTTCGGAAAGATAAAACACTAAAAGTCCTTCTTGAAAACTAATTTATATTTGAGTTTAAAAAAATAAATAAAAAGGTGTTATTAGAAAATATGTTTTTGTTAACAAAAGTAGTGAAGAAAATAAGTCCTAAAAATAATCTTAAATTAAGAAATTAAATTTTGGTTTATAAAGGTGTTGACAAATTTGGTTTAAACCCTTTTGAATAAAAAAAGCTTTTCCTTTCGCTTGGGCCGTCACCCTTTCATTTTGGCCCAACCCTGCAGCAGCTACAGCCTAGCCCCCCTCCTTTCTCCCCC

>SbWRKY65

AAAAGCTCCCCTGCCTCTTCCCCTTCTCACCCGGCTCTCGAACTATCCAGCCTTCCAAACCCTAGTTTTCCCCTCCATGGCCGACGGCGATCCGGCGCCGGCGCTGGCACTGGCCAACGAGAAGCTCCCTGCCCCGGCGGCGGCTGACGTGGATGAGACGCGCCCGGCGCCACCGCTGGAGCCCTCCCGGGGTCCGGACGAGGAGAAGCGCCCGCTCGAAGAGGAAGAGGCGGAGGTGGAGGCGCACCCGCCGCGGGAACCAACCGGGGCGCCGCCAGTAGATCGCTTGGGGATGGAGGTGGTGGCGGCGGCAGAAGCGGACATGAAGGCGAACGAAGTGGAGAAGGAGAGGGGGGATCGGGCGAAGGAGAAGCGGGAGAAGGATAAGGGGAAGGGGAAGGAGGGGAAGGAGAAGGAGAAGGTGGAGGAAGAGGCGAAGCTGAAAGTGACTGCGGTGGTGAAGGTGGAGGGGACGGAGAAGGAGGTCAAGGTGACCCGGCCGCCAGCTGGGGCCAGTGCGGAGACGCCCATCCTAGCGGTGCCGGTGGTGGCCGTGCCTTGCTTCATTGCGCCTCCAGGGTTTGCGGTGAGTCTGCCAATCCTCTCTCTCACTAAATATATTGATGAGCCCTGGATGAGGTTTCGAGCCACATATTTCTAAGTTGATTCATTCCTGAACAAAGTGGTAATTTCATACCCTAGTCAGCCGATCATAGCCGGTGCTAAATTGTTGTTTGATATCACCTTTGATGGCCAATTAGCCAGTGCTAGAAATGTTGCCTGAGTTTTTGGGTTGCATTTATGCACACCTTGATATTTTTGCTTGATTAGAATTACAAAATCTTTGTAGTATACAATCTAGAGGATTGACATGCCATCACTCATTTTAAGGGCAGCTGCATACTGAAATTTGCCCCCTCTGTGGTTAATTCACATGTGACTTATGTAGCTTGTTTATGTTCTAGGCTTTGTACTGAATCAGAATTATAATAAATTAGAAATTTAGAACGCATTGACCTATGTATTTATAAAATATAGCAATTGAAGTTCCATAATATTCTTAGGTTTGTGCGTGTACCATAATTAAGGCTGGTGTTGCAATTTTATTGCAGTTCCATAGTTGATAGTTTATTGCTCGAGAAGAGTGGTCTACTTGCAGGATATTCAGTCTTCCGTATAACTTGAATTCGAAATGTTCCTTTTTGGGGGTGGGTTTGGTCAGTAGCTCCATTCAGATGACACACATGATGGCCAGATATGCTGCAGGAGTGCAGGATAACCAGTTAAACCACTCAACTATTGGCAATGCATTGTTTTTGAACATGAGTAACATTTGCAAAGCAACCCCTCTAATTTGATTTTTGTTACATTATGTCATGTTTACACATGAGCACATTGAGCGAAGTAACACATGATGTTGACTAAATAATTACAACAAGTTCCATTGGAGTTCTTAATAGTGTAACATAGCAGCAATCCAGGCAGTGTCTAAAAAACGGGTGCACTCAGCTATACAAAGAATGGGTGCAAAAATCATAATCTATGCCAGATTATTCCCTGATTATACCATGCAATCACAACTAGGTTGGGTGGGTCGGCCCATAACCGGTTACCGAACCTTAGAACCTGACTCCAGGCACTAAGGTGCTGTCGGACCACTGTTCTATTTCATGTATGTTAAAGGGCTGGATTAATCTCAT

TCAACATATTACAGTATCTTGGATCTCATGGTTTTTACTTTAGGGTATTCTTTCACCCAAATTTGAGATTGGAAGATATCTGTCATTTACTTCTACTAGTAAATGGCCCATGTGTTTTTATGGCCACAAACAAAATACCAATTATTCTAAAAAAAATATTATACAAAACAGGCAAAAGCAAGATGGGGTGGCCAATAAATCAAAATTACACATGAGAAAAATACAATCTAACTCATGCTAGATGTACTTTCAAAGACAACTATCACCGAGCCTTGAATTAATTCTTCGGCTGTGTATTGACCCCTTACAACTGAAGCCTCAATGTGATGTGAGATTGGAATTGAAATTGAAGAGGCATACAAGACAACTATATTACGGAGTGTTGATGATACCTTGTTGCTCTTTTTAGTGGTAGAGATAGGTTATGAAGCTTTCTTATATAGTCCCTCTGTTCTTTTGTATATGACATTATTTAGTTCAATTTTGAACTAGCCAGCATCATATGAAAGAGCGGAAGGAGTAGGATCTTACTATAAATACACTTTATGCATTGTTTTCGTGTGTTGTATGGTCTATCATATCTTCCTCAGTTCTATATAGTGGATTCTTTTATTGCATTACTTAGATCTGTAAAAATCTTTTTCTTGAATATTAGGGCCAGTTTGCAATGACTCATCAAGCAGCTTTGGCGAGTGTTACAGCACAGGCACAAATGCACTTGCAGTCACCAACTTCATCTGCATGTTCAGAAGTGCCATCAAGTCCATTTTATATGACACCAAGATCTCTAGTGCCACTTCAGCAATCACCATCAGTAACTGAAGGAAATATTTGTAAACCAATTGCTGACAAGTCATTTTCATCTGATTCAAAATCACACCATGTTGTTGTAAATATGGTAGCTGATGGCTTCAACTGGAGGAAATATGGTCAGAAGCAGGTGAAAAGTAGTGATAATTCCAGGAGCTACTATAGATGCACAAACTCAGGTTGTCTGGCTAAAAAGAAGGTTGAGCATTTCCCTGATGGCCGTGTTGTGGAAATCATTTATAGAGGAGCTCACAATCATGAACCACCACAAAAGACCAGATTTGCAAAAGAAAGAGTGACTCCAATTGGTGTTCCCTCTGGAGGCGAAACCTTGAGACTTGTGAACACAGAAATTGTAGAATCCAGTACCCCTACATGTAAATTGGAACAGAGTGCCATCTCAGAAACTTCTGAGCAGCATCTCTTTTGCTCAAGTGACTGTGAAGGGGATGCTGGTAACAAATCTGAAAATGAACATCCTAGTGCAGAACCCCTGCCAAAGCGAAGGTCATTGTACTGTAGGACTATTTGATTCATTCATATCTATATTGATTAGTTGTTCTTACCTTTTGTATCCTCTATTGTTGTTCTTTGAACAGGACACTTGAGACCACAGCGCCTAACTTAACGCCAGTTCTCAGAACAGTTAGAGAGCAAAAGATTATTGTGCAGGCTGGGAAGATGAGTGATGGATACAGATGGCGCAAGTATGGGCAGAAAATCGTGAAGGGAAATCCAAACCCCAGGTGGGAAGCCGCTCACTTTGTTGTCAGCACATGACAATCACAGAGCAATTTCACACTGTAGAACTTAATAACAATATTGTCTACTGTTATTTCTGATCCTGAAAGAGGCTTATTATCTCCTCTTATGGCTCATTATTATTTTCA

TTTAACCTAGCAATACTAAATGGTTGGTTTTTGTTCTAATCTCAACATCTGGGTCATGTATTTGTCCATTAGGCTTGTGGTTAGTAGGGTGGTTTCGTACATTAGGTGAGCTTTATGTCCCTGATGCTTAAATTGACCATGAACATTACATCTGATGGTGCTTTTAGAAACTAAATTTCTGATGCAACAATGAGATATTGTCGTGTTCTCATGACTAGTCTTCAAGCATTTTTTTTTTAAGTTTTATTATAATATAGTAGCACATCTGCAAAACTATTATTTTATGTGGTCCCGTGAAGGTTGTAGCAGAACCTACAAACTTGGTTTATGAGATGGAATCAGTATTGCAATTAGCTATTCTTGGTGAAAGTTTACCATCTTTGATGTAGAGTCCCTTTCAAGATACAGTAAATTTTACAGAACTACAACTTCTGGGGCTAGTTTCATAGAAATATAACTGTTTGTGCACTTAGCAGCACAAGACTATGAACTTTGTATGGTGAAAACTGTAAAACCGAGAAAATGTAAGAAGTGAAAGGGCCTTTAGCAACTGGTAAGTATATTTCATGTGCCGAATTGTTTTACATTTGTTTGGTCCTGTGCCTATGCAGTGCGCTTATCACCCACAAGGCCTTGGTGTTTAGGATGCACAGAGTAATTAATCCCACATATCCAACAAGTTATAGGCTATTGATATGGGTACTAATGCAATGATGGCAATATCTTAAACTATGTTACTGATTACAAAATTTGTAAAGTTTGGCTGCAGTCACCCTTGTTTTCATTTGATCAAAGTAGATCAAAGTAACAAGCTTTTTATTAAAATAAACCATTAACTCTATACAATGTGTTAGTTCTTACTTAAGAACCCTAGTTGTCTAATATCACCTAACAAAAGGGTGTAATGCTGAGTGCTCCCCTAGTGAACCATTCCATCCAAAAGAGATTGAGGTCTTCATCCCTTAACCCTTAGTGATGTTGTACGCTACACCTTTAAATGTGATATGTTGCTACTTCTTACATTTTGTATTTAGGTAGTCCCCCCCCCCAACCCCCAAAAAAAAACTCTGCCCTGGGGAACAGCCTCCCCGTGGCGTTATATTGAAAAGCCTCAACCACAAGGTTGAGAAACCCCCTGAGTTCTACCTTAGCCCTATAGAGCTGCACTGTAACATAGGCTGACCACGACCTCCTGGGCTGGTGACCACTGCAACTCAACTACCCCCCTGGAAGTTGGGTCAGTGCCCAGTCACTATTTGTTTGGGTTGCCAGCAAGGTCATCTTTTTGGTGCCACCAGGATTTGAATCCTGGGTGGTGTCTCCCTCATCTAGGGTATAATACTACAATGTGACTGGTTGACTAGTAGCATCATTGGAACTGTGGAAGTTGTTAGTAGATGGACAGTGGGGGCCTAGTGTGGAACCTCTGTCATGCACACAATGTTCAATTAACCCAAAAGTGATACTGTCCTGTTTAGTGGTGCTGTTGCTTTGTGTGGTTGCAGATTGAGTCATTTAGTCATTTGGTCCAATTTGTTTTTGTTTCTTTTGGTACACTGGTGTGTATATTGGATCAAAGTTCCCCACTTATCTTCGTTGTTACAGTTGTCTTCTATAAATAGCTTCCATGTTTTCAGAATACAATGTACGTTCCTTTGCAAGCAATGTTGCTATGCTATCTAAATTCTCACTAGCACTA

TATCAATGGTTGCCAATTCCCAACAAAAACTTGTCAGGGAATGAAGATATAAGTGGAGCTGATATCTTAAATGTCATTTTTCATGTGCATAAAACAAATACTTTCAAGCTTTGCAGTTCAAAATTGTTTGTTACATCTGCCCATTTGTTCTGTCTAGGTGCTGTTTTAATTAATTTGCTTTCTAAATTCTGAATCAACCTCTGCAGGAGTTATTACCGCTGCACGCATGGTGGATGCCCTGTCCGTAAGCATGTGGAAAAGGCACCAGATGATGTTAACAATATTGTTGTAACTTATGAAGGTAAACACAATCACGATGAACCATTCAGAAGCAGCAGCATACCAGTTTCTGCAATCAGTCCGTCAGCAACAACCACTGAGCAACCCAACACATCAACTACATCAGATGAGAAACCTCCAACCATTACTCAGAAGGATGCCAACAGTGAGTCTGATAAGGAGACAACTTTGGAATTTGGAGGTGAGAAGGCACTTGAATCTGCACAAACACTGCTCAGCATCAAAACTAACTCCGATGACATGAAGAACTCTGTCCTGAAAGAAACTTCTGCTGCAGTACAAGTCCAAAACAGTTGAGCATGCTGAGATAGGAGGTGATTCGATTGACATCTTAGGTTGTCATATTTATTGGTTCAGTGATATGTTATTGTGTTAACTTGTGTATGAGGTGAGGCTTTGGTGTTCGGCGTTCCGTGGAGCACTGGGGAACATTTGAGGAAGCGTTAGGTTCAGGAAGCCATGACTGGTCGTCTGCCTGTATAGATTCCCATTCCCTAGTGTGTATAGGTACTGTATTTTACATGAGGCTCAGCAACAAGGAGACCAGATAACATGTGTGGGATGGTTCTTTAGAACCTTGTGTGCTGTATTGTTCTAGTTCGATTTATTGCATAGCTGATATGAGATGTGATTTATTTATATATCGGTATAACATCGGAATACACATTATGCATGCTGTCCTATTTGCAGTTTGTAATAGCTGCATCATTGTCTTGTCTCCAGAGTGTCTGCTCACATAAATTCTCTGGAACTGTGTACAACGCAATGGATTGATGTTGGCAGCCGCTACTTTTATGCCAATGCGAAGCTGGGAGCGGGCTCGAAGTCCTTCACCGTATTCTTCACTGCGTTGATGTCGATGTCGATCAGCTTCCCCGTGGCCGACGAGATGACGGTGCAACTGCTCTTATCCTCCACGTACGGCAGGAACAGCTTCGCCTTCTCTGCCGACGAGAACAAGGCCACACTGGC

>SbWRKY87

CGACGAGTACGACGCTGTGGTGGCGGTGAAGCAAGAAATGGTTGTCCAGCTGAGTGACAGCCGCCGTGACGCCGACGCCGATGGTCAGATGGCCGGCGCGGCGGCCGTCACGCCGGCCAACTCATCGGTGCTGTCGTCATCCAGCTGCGAGGCGGGAGCGGATGCCAACGACGACGACGAGGAGCCGTCGCGCCGGCGGTGCGGCAAGAAAGGTAGGATCGAGGGAGAGGAGGAGCAGGAGGGAGAAGGAGAAGCTGACGATGATGCAGCCGATCGGAACTGCAAGTAAGGCCTTGTTTAGTTTCGAAAAATTTTGAGAAATCGACACTTTAGCACTTTCGTTTGTATTTAACAAATATTATCTAATTATGGACTAACTAGGCTCAAAAGATTCGTCTCGTTAATTTCGACTAAACTGTGCAATTAGTTTTTATTTTCGTTTATATTTAATACTCCATGCATGTGTCTAAAAATTCGATGTGACGGGGAATATAAAAAAATTTGCAAAATTTTCAGAGAACTAAACAAGGCCTAAGTCTAGAATTATATACCTGTTGCAAAACTAGCTACACACTATATATGCTTAAATTAAAATTTGATGTAAATCTTCTTGTTGATTATAGTAGTAACAAGCTTAGTTTATAAACAATCAAGGGAATAAGGAAAAATCGCTTTCAAGTGTTAGATATTTTTCTTCTTAAATAATTCTTTTTTGCTTAAATAAAACAAAGATCCCACCGTGTGTATACCGGTCCGGTGTGAACACAAAGGGATTAGGGTTTGCCACCTAACTTGACCGACAAACAAGTCCTAAAAAAACTCTTTTAACCAACAAATAAGCTCACTCTTGGCAATTTACAAATAAGCTCACTCTTGGCAATTTATTTAACCTTTTTTTTATAAAAAAACATGGTCGAGTAAAAACTCTTTTAACCAACAAATAAGCCCACTCTTGGCAATTTACAAATAATCTCACTTGGCAATTTATTTTTTTTTAAAAAAGAACATGGTCGAATTAAGTCCCCTGTCCATGAACATATATTTTTATTTAGCGTATGGTCTCCAAATCAATTAAGCTTTGGCTATTATTTTCTTACATATGTATGGAGTATATATAATTTATATATTTGAATATTTGATGTTTTAGGTACGGTGTCTATTTTTTCATACAACAATCTTTGATAACCAACTATGGAAATTAAAGGATAATTTATAAGATACAATTTATAGTTCTTTTCTACAGTCAAAGAACAACAAAATATTAATAGCATCATAAATCTCGTGATACTTATTGTTCATCATAAATATAAATATCAGTAGCTTTTTATATGCTTAAGATTGTTTGACTTCTCGATAAATGAGGTTTAGGACGAGTAGCTTTTATACGCAAACTAGGTAACTATACCTCAATGCATAGTTTCATAGTACAGTTACCAAGACTATAAACTAGGTAACCGAGTCACATGAGTTTCATGGGGATGAAACTCTTCTCTCATCTGATGAAACTCCTTCATTTAATGACCCTGCCAAGTCAGTAATTTTGCTTATGTGACACCCTATTTAATATGCATGACACTATCATGAAACACGCATTGAGACTAGCCTAACTCCATCAAGTTCTTCATCCATCTTGGATGAATCAATTTTCATAGTTCTCTTAAGTCAAACTTTTTTAGTCAAGTTTAACCAAATTTGTACAG

AAGAGCATCAGCATCTATGAAATCAAATTTGTACCATAAAATATATTTTTTATAACGTGCTCGTTTGGTTTTATAAATATTAAATTTGATCAAACTAACATAAAAAGATTTAACTTAAAACAACTCTAAAATCTGATTTATTTAGAGACAGTATTTTTCATCCATTATTCGTCCTATCCCATTCATGAGGTACCGGTCCTGATGACGACTTTGCAGGCAGTACGTACTAGTACTAGTACACTAGTTCCATGGACCAATTTGTGGTCCGCTGCGTTGCTTCCTTCCATCTTGTCGTATCGTACGTACAGTACTCTATACTGCAGTTGAGAGTGCGACCTGCCGCACAGCTACCCCTGTCGTCCTTGATGATGTGTACATTTGGGCACGCGTACGTGTTTATAATTCATTTCACAATTTCACATCACATTTCAGGAGCAGCAAGGAGAATAAGAAAAGGAGAGGCGAGAAGAAGGCGCGGGAGCCCCGTGTGGCGTTCATGACCAAGAGCGAGGTCGACCACCTCGAGGACGGCTACCGCTGGCGCAAGTACGGCCAGAAGGCCGTCAAGAACAGCACATACCCAAGGTATCTTTAGTCAAACACGTGCATCGCACAGTTTCCTTGTTTTCTCTGTTCAAACGAAACAAGACCCAGAGAATCCAGAGAGTCCAGACCAACGTGTGTCATGACCACGAAAACCATGTGTGTAGTTTAGTCGTCGCATATGCACCATATGCATGCTACACGCAGTTATTTAGTACAGTATACAAACCAGCTGCACGCTGTTCATGCACACGCTCAGGAACGACACAATATATAATAATGCGTGCATACATATGCAGGAGCTACTACCGGTGCACGACGGCGCGGTGCGGGGTGAAGAAGCGGGTGGAGCGGTCGCAGCAGGACCCGTCCACGGTGATCACCACGTACGAGGGGCAGCACACGCACCCGAGCCCCATCGACCTCCTCAGGAGAGGAGGAGGCGCCGCCGCCCTCATGCGCTCCGCCGCCGTTGCCGGCGGCTTTCGCCGCCCCGACGACCTGCTCAAGATCGACGATTACGCTGGCACGCCGATCGGCTTCCTGCCGCTTCTCCCTCCCGGTGGCATCGGTGCTGGTGGTGGTGGCCGCCTGCTGCATCACCGAGCTCGTTCTTCGCAGCTGGCCGCCGTGGACGCGTATGGCGGCATGCTGGAGCTGGACTTCATCCCTTCCATTCCGCGATGAGCCATGAGAGCATTATGTCGCTCACCTTCATTTCCAGGCCAGCCAACTAGCGAGCGTACATATATACGGAGTATTTGTCACTTTGAGTCGAATTCTAAAGAGTATTATTTACTGGAACTGGAATCAAAGTGTTTGCTTACAAAACCCCAAGTCTTGTAAAAGTTGCACCGGCCAGTGATCTTTTGGTTTGGCCTTGTTTAGTTCCGAAAAATTTTGGGAAATGGACACTGTAGCACTTTCGTTTGTATTTGATAAATATTGTCCAATCATAGACTAACTAGACTCAAAAGATTCGTCTC

>SbWRKY88

CCACCGCACAAGCACAACATCTCCCTCTCACTCCTCTCTACCTTCTCTCTCTAAATTAAAGCGGCGAAGA

CTTGCACACAAGTTCAACGCTAGCTATCACCCACAGTCGTCCCCAGCTACCTCCTCTCCATCTCCCCAGT

CCCCATTGCACGCTTGACTACCTCCTCCATCCCCGCAGACTCCGGTCGATCACCATGTCTGGAGGAGCAA

CAGGAGTTGGAGGAGGCAGCGGCTACGGGGGCTTCTACCACGGGGACGATCCTGCCACCTCCGACCAGCT

CATCACAGCCTTCGACAACGACGGCGGCGGCGGCTTCTTCTTCCAGCAGACGGTCTCGCCGCCGTGCGCC

GGGGAAGTGGACGGCGGCACGGCGCCGTACGCGAGCATCGCGGACTACCTGCAGGGATTCCTGGACCCCG

CGGGCCTGGCCGCGCACTTCGGCAGCGACGACGCGCCGCCGCCGTGCCGGTTGGGCGGCGGCGCCGACGA

CGAGTACGACGCTGTGGTGGCGGTGAAGCAAGAAATGGTTGTCCAGCTGAGTGACAGCCGCCGTGACGCC

GACGCCGATGGTCAGATGGCCGGCGCGGCGGCCGTCACGCCGGCCAACTCATCGGTGCTGTCGTCATCCA

GCTGCGAGGCGGGAGCGGATGCCAACGACGACGACGAGGAGCCGTCGCGCCGGCGGTGCGGCAAGAAAGG

TAGGATCGAGGGAGAGGAGGAGCAGGAGGGAGAAGGAGAAGCTGACGATGATGCAGCCGATCGGAACTGC

AAGAGCAGCAAGGAGAATAAGAAAAGGAGAGGCGAGAAGAAGGCGCGGGAGCCCCGTGTGGCGTTCATGA

CCAAGAGCGAGGTCGACCACCTCGAGGACGGCTACCGCTGGCGCAAGTACGGCCAGAAGGCCGTCAAGAA

CAGCACATACCCAAGGAGCTACTACCGGTGCACGACGGCGCGGTGCGGGGTGAAGAAGCGGGTGGAGCGG

TCGCAGCAGGACCCGTCCACGGTGATCACCACGTACGAGGGGCAGCACACGCACCCGAGCCCCATCGACC

TCCTCAGGAGAGGAGGAGGCGCCGCCGCCCTCATGCGCTCCGCCGCCGTTGCCGGCGGCTTTCGCCGCCC

CGACGACCTGCTCAAGATCGACGATTACGCTGGCACGCCGATCGGCTTCCTGCCGCTTCTCCCTCCCGGT

GGCATCGGTGCTGGTGGTGGTGGCCGCCTGCTGCATCACCGAGCTCGTTCTTCGCAGCTGGCCGCCGTGG

ACGCGTATGGCGGCATGCTGGAGCTGGACTTCATCCCTTCCATTCCGCGATGAGCCATGAGAGCATTATG

TCGCTCACCTTCATTTCCAGGCCAGCCAACTAGCGAGCGTACATATATACGGAGTATTTGTCACTTTGAG

TCGAATTCTAAAGAGTATTATTTACTGGAACTGGAATCAAAGTGTTTGCTTACAAAACCCCAAGTCTTGT

AAAAGTTGCACCGGCCAGTGATCTTTTGGTTTGGCCTTGTTTAGTTCCGAAAAATTTTGGGAAATGGACA

CTGTAGCACTTTCGTTTGTATT

>SbWRKY30

CGCTCCAAAAAAGTCGTATCTTGCGCCTATCCTCGCACGCTTTTGCACCCCAGGAAAAACGAAACATAAATAAATCAAAAGGGAGCTGCTGGCTGGGACTGGGAGCTTGACAGACCTGCTTCTGAAAAGCTCAAACCCTCCTCACTTCCACGCCTCTCCTTCCCTTTCCACACTTAGCTCCCCAGCAGACTCCCCCACTCCCCATGTCTTCTGGTGGAGGAGGAAGCAGTGGCGGAGGAGGAGATCACCATGGCGTCTACCACCAGCATGGCCACGGCCACCTCGCCCGTGCCGATGCCGGCGCCGAGTACGTGTTCCACAGCAACGACATGGAGAGCTTCTTCTTCAATCAGCCGGCGGCGTCGGCGGGCGTCGATGGATCAGGCAGCAGAACTACTGGCGCCGCCGACGAGCTCATGCCGCCCTACTCCAGCATCACGGACTACCTGCAGGGGTTCCTGCAGGACCCCTCCGGGCTAGCTCGGCACCTCGACGCGCCGTGTCTTCCCGCGGAGGACGCCCCGCTCAAGCACGAGCTGTCTGTCGATGTGAGCCACGACAGCCAGGGCACCAGCGGCGCGCCCGGAGGAGAAGGGGCGGCGATGCACACGCCGAACTCGTCGGTGTCTCTCTCGTCAAGCGACCGGGAGGGGGAGGGCGGCCAGCAGCCTCGTCGGTGCAAGAAGGGCAGGCCCAAGGCGGAGGATGCGGAGGGGGATGAGAAAGAGCAGGAAGATGGGGAGAATTCCTCCAAAGCGTACGTTCTCGATCTTTAGTTTAATGCTACTAATCTGTGTGTTCAACTGTTCATCATCTCGAATTGTATTTGCATCTCTCTTCATCAGGTTCAGGAGTGCTTCTCTATGCTCTCTCTCTCTCTTGGTGGGGGGGGGGGGATCAGGAGTGTTTCTCTTCTTGGTGCATGATCGATAGATCTAGTCCTACTGTTACCTAAATGAACAAAAATTTTCCTAGTTAATTAGGTTTATATGTCTATATGTATACTACACGGTGTAAACATTAGGGTTTGTTGCGATTGCTTCTCTAGGGTTCTTAGATCATCAAGAATTCAGATCGATCCACACCAGCTCCAGATTCATAGTACTATAGCTTAGCTACGCTCTAGCTTTCCCTCTTCTTTAACTTCTCTTTCCGTTCCTTTTTTGGTCCCGTTTCCAGTATGACTTTCTAGATCATTTGGTACTATATCCAATTCAATTTGGCAACGTACTCTCCAGAAGAGATCTTCCTTTAAATTAAAAAAATAAACAATTTGTGTGCTGCAGCTTCAATTTTTGACCAACTAGTAGCTATATCTCTGAAGAGAAAATACAAAGATTTCTTTTCCCCTTTTTTAGAATAGATTTTAATTAAAGATCATAAATGATATGTACCGAAAGCACCAAACAGACACCACAGATCTAGAGTATCTTGTGAAAAACACAATCTGGATATGACGTGCTCGATCCCAACTCCGAATAGCTCGCTAGCTAATCTATACTCGCGCTACACGCACGTCACTATATATAGACTACTACAGCACTTGTGTTCGAATAATTGGCGCTAATAGTCCACGTGACATGAAATATCTACTTCATCTAGCTCCAGCTAACTAGCATTTAGTTCATATCATCGCACTGGATTGGCTTCTGATATATATATATATATATATGCATATATGTATTCTCATAACAGGAACA

AATCCAAGAAGAAGGCTGAGAAGAGGCAGAGGCAGCCTCGCGTTGCCTTCCTCACCAAGAGCGAGGTGGATCACCTTGAGGACGGCTACCGGTGGCGCAAATACGGCCAGAAGGCCGTCAAGAACAGTCCTTACCCAAGGTCTGTATACTTTTCTTTTTTCTCATGACAGTTTCACTTGTTCTTGGTTCCCCCATCTATGGTCATTATAATTGCATGCTAGCTATCACACATCTATATCTAGCTATCGTATATGCTAATTATAGGAGCAAGTAAACAAGGTACCTAATGCTTTCTACAACTTGTATGTAAATTCTCACCAAGATCGAGTATATACATGATGCATGTACCTTATTAGAACTTGTCATATATATTCAAATCCGATCTCCGGCCAAACAATGCATGCAGGAGCTACTACCGTTGCACGACGCCCAAGTGCGGCGTGAAGAAGCGCGTGGAGCGGTCGTACCAGGACCCATCGACGGTGATCACGACGTACGAGGGGCAGCACACGCACCACAGCCCCGCCAGCCTCCGCGCCGGCGGCGCGCATCTCTTCATGTCAAACGCGCACGGCGGGCTGCCGCCGCACCTGATGCCGTCCAGCTTCGGCCGCCCGGACCTGATGAGCATGATGCACCCCGCCATGGGCGCAAACCCTAGCATGTTCCTGCCAAGTATGCCTCCTCCTCACATGTCAACACCATCTCCTGCTCCTCCTCTTCAGCAGCACCACTTCACTGACTACGCCCTCTTGCAAGACCTGTTCCCTTCCACAATGCCCAACAACCCATAACTAACTGCTACACAATTAGCTAGCCTAGATCGACGGCATGAGATGCTGTCGAAATACTGCGCCAAATGTGATCACCTGATCGATGTGATGACCTTGTGCTTGGGCCTGGTCTAATGGAGAGGGAACAACAGTTATCATTGATTATTAATCTGCTTCCATCTTAAACTTTTGTTTCATTTCTTCCCCTTCCGAACTCTCCGCTGCGCGGATTGCCTGGGAGGTACTAGAGATGGAACGGAGTACTTTATAACTATATCACTAGAGCGGCAACATTTATTTATGTACGCCTCTAGACTTCTTGTACAT

GCATGTACTAGTGTATATCATGTGTTCCTCATTGACTCATTTGGGAGAAAAAAATTAATGAAGGAGTATTTCATTTCTCTAGTTCCCGTTTGAAATTAAAGAATATGCATGCCCCCCTTTAATTTAAAGAACTCAGAAGAGATGTCACCTCCACTTCTGCTTGTTTTCCTACCATGGGTGCTTGTTGGATGGATGCATGTGTACAGGCATGGTGCTTCTTTTGTCCATGTGTAATGTTGGTTTATATATAAGGTCCCATTTGAAATGCATATTTTTTTTTTCATGTTTTCTGCATTTTCTTGTGAAAATAAACTGATTTTAGCATGATATTTGAAATTCTTACATTCCAAAAGGCTCTAAAAAGGAATTCCGTGCTTTGGTGTACGCCGTATAGCGATCCAAGCATTTTTTTAGACTAGACCAAGCAAATTCGTTCATGGATTTTTTGTTGTTTCAGTGGTAGGGCAACTGAGAAAATACTTTGGTTATTGTCGTTGGGATTGATGGTCTCAGCCTCCATGTCTTATTTATCTATGCTAGCTGTGACCTTTCTCATACAGTATGAGAGAGGCTGTGATTTTAATTGATTTTGTTTGGTACATTCCTATCTCAATGCAAGTACATCAACAATATGTTGAGGTGTCGCTGTGAATTTTTATTACAAATCATGCTAGCTGTATCCTGTTATAACTGTATTGAATGAATAGTTAATTTGTGTTCCCCCAGTTTATACAAATTTATGATGCCCTAAATTCTATTTCGTGCATATTCAAGCACAGGGGGACAGGTTTGTAAAGAATTGAAAGTACTGGCCAGTTTCGTACATGCATTATATTTTCCTCTAGTCAAATTAAAATATATATAGTTGGTACCTTTTGATCTAGTAGAATAATAGTAGATCCAAACATGCATGTAGTCATTGTATATTATTTGTACATGTACTCATTGTATATGGTGTTAGTTTGCTCTTTATCACTCTCTAGTAGACGCATATAGTCATAATTTCATTTTAAATAGTACTCAATTAACACATTCACAATTGTTGACGGCGCTGGCTTATAATCCAATGTATATGTGTGCGACCACACTTTTGAATTGTGTGGACCAGGGTGAAAAAAGGTTCAAAATTTCGTTGAAATCTCTTTCAAATTCCATGTAATTTTGAAGGTGGCTGAAACAAAAAAAAATCCGCAATTTTGTATTATAACAACACACATTTATCTTTGTTGTAAATATATACCTACAAACTCAAATTTTCTATATTGTTTGTGTTGCACTTTCTTCTACTCAATAGATGTGTAGTCATAAATCATGTTAATATTTTGTTTAGAACTTTTTTTAAAAAAAATCATACTTCATATTTAAGTCTCTAAAAAAATTGGTGAAATTGGACTGAATTTACGTCAAGTTTTGGGGTAACTGAAAAAATTGTAATACTGAAATTGAAAACCCTACTATGGACGATTGCCGTCTTGGATCGGAGTACACATGCATGCATAATAATAAGTTACAGACGCACAATTTCTCTGAAGCCAATATACTTATATACAGTATGTCTTATCTCTCTCCCTCTTCCATGGTAAAGTTTAGTTGCACCTTTTTTCTGTTTCAGTTTTCCTCTTTGGTGTACCTGCTGAATTCCATAGTTTTTGGTAGCCGTCAACACTCAAGTGCCATGTCCTTTGATTCCCTCTGCCGT

CATTTTTGACCAACAACCCCCCTCCCCCCCCCCCCCCCCCTCCTCCTCTACGTACACACACCCCTGACCCTGAAAGCTAGGGTACAAATTAGGTAGTCTATGCATCCAATTGACCATGTGTTGATGATGAAAGAGCGGAATTGTAGGTAGGTAGCCTAGGGAAATTGACAAAGAAAAATAAGTCAATCCAGCGTTTATTTTAAAATGTTTGATGAAAGTTTGAAAACAATATTATATCATCGTCTCGACCACACACATACAACAACCATGGAGGCATGGAAACACACATACATGTTCCAACTGAAGACATATATTCAAAGCAAGCGAGAAAACTACTGGTAGCTTGTGCGTAATTAATTATCATCATATATTGAACACCACAACTACAGTGTATTTGGCAAAATTGTTTGTCTAGTAGCGGAATCATGTGCAAAGTGCGCGTGTTCTAATATAAAATATCAGGTCCAACATGTATGTTTTCGATTAAACGCACACACGCCTTCTAAACGTTGTGACGATTATGAGTCCACACACGGGCCAGGTATCTTATACGTACCCAGCTAATCTCATAATTTAACTGTTGCGAGCGTTGAAGCCATCCCGTTTAGACTGCTAATTTTTTTGTAAGCTACAAGCCTGCAATAGCTAGATGAAGATGGCTAATAGTATTTTACTATTTATATAAGGTGGTTGACTAGTCAAAATTAGTGATATATAAGCTGGACTGTAGAAACTTGATTATTTAGACCGGCTAGAGTTTTTTTTTCAGCAGCTAATAACTTCTTCACGCATACGTCATTATGTATGGCCTTCATAAGCTGTACTTGACATTATCAGCGTTGCCGGCCCATGCTGATATGATTGCAGGTAGGGCCGCCGTGATGGTAATACTATTAATGTAAATGCAAAGAGTATGAAGGATTCATCAATATGTACGCTCTAATTAGTAAACATGATTGTTATATAGTGTAGTCATTGGCCACACTCATCTGTATACGACGGATCGGATGGCCAGAGAAGAACGGTTCTTTTTGTCTTTTTTTTTTTCTCGAGACAGATGCTCGCCCGTGTTCAGCTAAAACTAAAGACTAACGCGGCATTTTATTGAGATCCTAGGGGCACGGTTTCGTTTCGCCCCAAATTATATATATATAATGTGAACATTACTAGATACATCATAGAAGATCACATATATACTAAGAGTATCTCCAACGGTTTGGCATTCTTGTTTTGCATTTGTGTTTTTTTGCCAAAAAGCTAAAAATCACCCCTCCAACGGTACGGTTTTGTAAACAGAGTTGTCATTTTAGTAAACTTGGCATTTGAGAGGCTCCCCTTGGCATATTTGTCAGTTGGGTTTTGGCTTGGCAAACACGTCCGCCCGGTTTTCCTGCATCGATCCCGCGCGTGTCCAGGTCTCTTTGGCGTCGTTTACTTCGTCGCTGGGCCTCCTCGCGCCAAATAGAGTTCGCGCCACCATTGTGTTGCCACGAATGAACAGAAGGATCGAGTCAAGGAAGATATCATGGCATCATTCCTGGCCTCTTCGCGTCGATCTCGAGCGACGCGACACCTGATATACATCGACACGAGCAGTGCATCGACGCAGCTGACCGATCTCCATCGATCAAAGCCCGGTACGTCCCTCCCTGTAGATCTCCTTTGACGCGATGATGTGGTAGCTACTCAAGGAATACCTT

CCTGTAGGGTTCATGGCAGTGCCATGTGACATGTAGGGTTCATGGTAGTGCAGTATGTAGAGTTCATGGTAGCGTAGTAGTTCTGCAGCACTCGAGGATTGGCAAGGGCCTGCTTGATCATTAGTGTCACCTCCACGCGTGCGAAGGAGAAGACGCACGAAGAAAAGTTGCGCGCGCGGCAGAAACGCGCGAAAGGATTCGGCCGGGTCCACTTTAGGTAATTGTCAAGTAAAAAAATACTAAACCTTTGAAGATGGCCTATTTTTTTCCTTTGCATTTTGAATTGAGAGTTGACAAACAACAATAAATGCCAAACCAAAATTGCCAAACCATTAGAGATGCTCTAAGTGAACATTAGTAATACACCATAGAACATCACTACATACATTATAGAACATCACATATATATTAAGCGAACATCACTAATAGAACACCACATGTGTACTGAAGTGGACATCACTAATACACCGTAGAACATCACATGTATACTGCGAGAATATCCTATATATATTACGCAGACATCGCAAAATAAATCACAAAATATTACATGTATACCACATGAACATAAAAAAAGCATCACAGAACATCAAAATGTATATCACGTGAACACAAAGGCATAAAAACATCGCATATATGTTTACTACATAAACATAAAAAATAACATAGAATATCAC

>SbWRKY32

ATAACTCCAGGGGAGGGGGCGGGAAATGGACATGTCGTTTGGGTCTAATCAATTAGTCCTACTAGTAGTTTCTGTCACAAAAAGCTGGATCAATTAAATAAAACGCCAACCCCAAAAGCAGCGCTGCTGGTAAGAGAGAATGAGAGAAAAACAAAAAATCCTCCCACCCAACCAACCATCCATCCATCGTCCATCTCCATCTTCTAGCAGTATATCTGTATGTGCATCATCTTCCTACACACACTCTTTCGCGCGCGCACCACGTACACAAAAACACGCCCGGCCATCCCTTTCGTTCCCTTGCCTCACTACGCCCCCACTACCATATAGCCGTATACGCTCTCGATCGCTTCCCAGGCCGTCTTGATCTCTCCCCTCCCTCGCCCTCGCCCTCGCCCTTGATAAGTGAGCACACCAAGCGGCAAGAAGCCAAGGCAAACCACAGTCGCAGCACCCAGCAGCTAGCGGCGCGCCGATGTCGTCGGGAGACTTCCACTTCCACGACGAGCTGGCGTCGCTGTTCGCGCAGCGGCCGGCGGCGCCGGGAGAGATGATGATGGCGCAGCAGCAGCAGCAGCAGCAGCAGGCGCCGGCGTCGTGGTTCGCGGACTACCTGCACGGCGCGGGCGTCCCAGGGATGGGCGGGATGGACTACGACCTGCTGTGCCGCGCGCTGGACCTGCCGCTGCCGGGGGACGACGTCGTCAAGAGGGAGCTGCTGGTGGTGGACACAGGCGGGGGAGGGGGAGGCTTAGGCTTCGCCGCGCCCACGCCCAGCGGGGGCGGCACGGCGCCGGTCACGCCCAACACGACGTCGTCCATGTCCTCCTCGTCTAGCGAGGCGGCGGGTGGCGGCGCCGCCGGAGGAGGAGGAGGAGGAAGCTTTGGTGGTGCCGGGGAGGAGGACTCGCCGCATCAGGGGAGGTGCAAGAAGGAGGAAGGGGATGGGGAGGAGAGCAAGGCGCTCGACAAGGGGGAGGAGGACGCTGACAAGGGCAAGAAAGGGTGAGTAGAGCTTGGCCTGGAATTCTTCTCGAGATTATAGACATCGACCAAAATCCTGTGTTATTTGCTTCCTTTTTTTTCCAACTTCTTGAAAGATCTTTCCGCTAATCATCGGTTTTCATGAGACTATCTAGATAGTGCTCGATCTTTTGGGTGATCCTGGATCAGGCGTCAGTTTCTGTGTCTTGGATTCTGCTTCCTTCCATTCCCCTGCTGGTCAATCAGTCATTCATTCCTCTTCTGTTCCTTGTTCCTTGTCCCCATTTGTTCTTCTTTTTTTAAAAATAAAAAATAAATAAGGTAGCGGCAAGGCAAAGGTGTGAACTTGGGCTAGGGTTTCACACTCGCATGTCACAAGAAAACAAACACGGAAAAAGTCGGTCGGTGTATTGTATAGCCTAATCTTCTAGCTAGTTGCATCCTCCTCTCCTCTTCCGTTCCCCTTTTGCTTTATGGATTTCATGCTAGCTACGCGCTAGCTAGCATGAACATGCATCTTCTTCCATTTGGCCAAGATTTGCATCCTCAGCTGATTCTATACATGTTTTCTTTTTGTTCCTTTTACTCTTTTGGTTCACCACTTCACCCCCCTTTTACCAAGATCATACCCCCCCCCCAAAAAAAAAAAAAAAAAACCCAAAGATCTCTTGAGATGAGAATGGCCCGGCCTAAAAGCTATAGCCCCCCTTGCAGAC

CAAGAGATATTCCTCCCCTCTCTATAGGGATTTGTTACAGCCCCCTTTCTGATGCAGGCAATCTAGCAGCATATGGCACAAATTTTGGTACAAATCTGCCGGCACAGCAGAAAAGGGGTGTGTGCACACAGCACAGCCACAGCAGGCAGTGTGTGAACCTGTTAAAACCATGTGCTTACGACTCTAGTCTAGCTGATTATTCGCACTACACCCAGCCAACCACCGCGTTTGAACGGGATCCCATCATCCCATCCCAGCCCAGCCGCGCGGCCGGCCACGCACCTCGCGTATCGTATCTTAGTTTGAACGAAATCTGCATCACTTTCCGCTGACCCGGCCGGCTAGCTGTCCGGTGGACGTACGGGAAGACGAACTGATGAGATCGATGCCCACGCCGGCTCTTGAATTGTTGCAGGTCACCGGCGGCGGCGAAGGGCAAGGGCAAGGGCGAGAAGCGGCAGCGGCAGCCGCGCTTCGCCTTCATGACCAAGAGCGAGGTCGACCACCTCGAGGACGGCTACCGGTGGCGCAAGTACGGCCAGAAGGCCGTCAAGAACAGCCCATACCCCAGGTACGTACCAGTCTTCCTACGCATACATACATACATACATGCACAGCAAGTAGTATATACAGTTACCCTACTGCGCACTGCACTGCACGCACCTCTCTCCCTTATCCATGTCTGCATGAGCTAATGGTGCAACCGCGCGTGGTGTGATGCATGGCGCGTGCGCGCAGGAGCTACTACCGGTGCACGACGCAGAAGTGCCCCGTGAAGAAGCGGGTGGAGCGGTCGTACCAGGACCCGGCGGTGGTGATCACCACGTACGAGGGCAAGCACACGCACCCGATCCCGGCCACGCTCCGCGGCAGCACCCACCTCCTCGCCGCGCAGTTGCACGGCGGTCACCACCACCACCACCACCTCGGCGGCGCCTTCCCGCCGCCGGCCCCGCTCCCGCAGCAGATGGCGGGCGCGCCGTTCGGGCGGGCAGGCGGCGGAGGCGGAGGCGTCATCGACATGCTGGGGCTCCTGCCCCCGCGCAACAACAACCACGCCGCCATGCCGCCGGCGATAGGCCTCGCGTCGTCCCGCGGCATGAGCGGCGGCGGTCCGATGAGCACCGTGGCAGGCGCGACGGCAGCGACGGCCGCCGCCACCACTACCTCGTCGTCGTCTCCTCCCTCGCTCCAGATGCAGCACTTCATGGCGCAGGACTTCGGGCTCCTGCAGGACATGCTGCCGTCCTTCGTCCACGGCAATGGCGGCAACGTCCAGCCCTGAATTCGATCAATTGGTCATCACCGCCACAAGCAGCAAGCTAATGAGGAAGGAAGCTAGCTACTTAGATAAAGAGATAGTACTTAATTTGGTTCTTGTTAATGCCCTTAACCTCTTTAATTTTTCCTTTTTTTTCACCGATTTGCTCCTCTAGATGGATCATATCGGTTCTTGATTTGTTTTTTCCTAATACTGCTGTGCTGCGAGACACATGCCGCTACAGAGTTTTCTGGCGCCGGCCGGGTCTCGTACCGTTTCTTATGATGATGATCTAGTAGCTAGTCCTTGCAGGTAGTACTAGCGGTGTTCTTGTACATGTACCTAATCAACTCAACCCCTCAGCTCATGCATTGCACTCTATCTGCTGCAGTACTGGCCATGAGCTATGATAATTTTATGCTAGCTCCTCTTTGCACGC

ACTCCTCCTGTCCATCTCCATTAATACAACACTACTATATATGTGCACATGTTTGTCAGCCTCATTGCATTGCTCAAACCAGAGGGCCATGCATGCATGGAACAGAATTTATTTTCAATTTTCTTCTTCAAAATCGCTTCTGCAGGAAGCAGGATATATGATATGTCGTCCTTAATCTCTTCTTGATATATATATATATGATGGCAGCAAGATGATGCACGTACTGATGTATGCATGGATCGATCGTTCCACCATGCATGTGTTTGCTAGCTAATAACATATTGTCATGCATGATGGGACTGTTCGTGCGTGGTTGGTACAAAGAGTAGTTATGGAAAAGCAGTGGGCGTCATTTTGCTCCTTGTTTTCGTGCTTGCACGTACATACTGCTATAGTGGCCTGGTCAGGTCGTTCCAAGAGCTAGTACTAGTACTACTCATGCATATAGTGATGTGAACCTGCCTACCTAGAACTAGAATGGCCGTGTGTGGAGCGTATATGATTGATTTTGATCAAGGATTTGGACGCACATGCTGCACGTACATTTCAACAAGCTAGCGCGCCAGATCGAGA

>SbWRKY84

AGCCCTCTCACCCAACCACCCACCCATCCATCTCCTTCTCCTGATCCTTTTCCCCGCACACACGATCACACTCTACAAAGCTAGCTTCCTTGCTTACACACCTCTCCCTTTCCTCACCTTCTCTCTACTGCGGCCGCCGCCGCTAGCTGGAGCGAGAGACAGCACCCGCGCGCCGCTCAAGGTCGTCACCGGCAGCTGGATCGGAGGCTTGATCTGAGGAAGGAAGTAGCCATAGCAGCAGCCGCTCCTGCGGGCCGATCCATCTCCATCCATGTCGGGCGCGAGGCATGAGCACCACCTGTCCGGTGACTTCCAGTTCCACGACGAGCTGGCGTCGCTGTTCGCGCATCAGCGGCCCGACGCGGCGCCGATGGCGCAGCCGTGGTTCATGGACTACCTGCACGCGACCGCGGCGGCGGCGTCGCCGCTGGACTGCGACGCCTTCGTGGGGGACTTCATCGACGTGCCGGCGGTGGCCGCGGACGAGGTAGTCAAGAGGGAGCTCGTGATGGTGGATACTGCGGCTGCCGGCAGCGGCGGCGGGACGCCGACGCCGACGACGGCGCCGCTCACTCCCAACAGCATGTCGATGTCGTCCACGTCCAGCGAGGCTTGCGGCGCCGGGGCCGGAGCGGGCGAGGAATCGGCGGCTGGTAAGTGCAAGAAGGAGGACGGCGAGGAGGAGGGGCTCGAGAGCAAGGACGACGGATCGGCGGCGGGCAAGGGAGACGGTGGGGAAGGAGAGGAGAAGAACAAGAAAGGGTGAGTTTGTTCGTCGTCTGATCAGATTTCCATCACCCTCTTTATCATCAGAAAATCTATTTGTCCCCGGATCTTGGAGTTATTTTTATTAGATCTATATATAAGCCTCATATATAAATACCAATTTAACTAGTTAAGGTGTTTGATGATTTGCCAAGCTTTTGATAGGTTTATCTGGACTAATTCCTCTCTGGCCCGATCTTGCTTGTTCACTTGTGTGAATCCCTTCTCACACATGCATAACATAATACTAGTGTTCCTTTTCGTCTACAGCTTTGATTTGAACAAGGTGTGAACCCTGCTAGGGTTTCTCATTTAGTATACGCTGCATGCTGCATGCCCAAGAACAGAAAACAAAAAAGAACTTGATCTTTAGGGTACACGTTCTATACCAAAGTTTTCTGTTTGTTTCAAACATCTTTTTTGCTTCATTCACAAATCGGCTTTGCTTTATTTCCTCCTTGATTTCGCGTCTTCCTTGTGTCTTGGCATCATCTACCTTCAATTTGACTGCACCAACTGCCTGCTGTTTTCGTAGTTAATCACACCCGCCCGGAAAGGATTTAAGATTATTATTAGCTGACGATCGAATCGGTCGAATCAGTAGCCTCTCTGTGTTGGCCATAAATTGCCCAACCGATCCATAAATTTCGTCGAGACTTGCTTTTGCAACTTCCCCTTTTGCTGTGACGATGACGGATCTCCAAATCATCCCAGCAGATCGATCAAAACTCAAGAACACTACTGCAGGTGAGTTTCAGTGTGAACCCTGTCAATCTGAAGCCATGTATAAGTGGGCGCCGGACGACCGTACATACGTAGCTACTCTTCCACGACACCCATGCATGTTTGAATGGACTAATAGTTAGGCCTTGTTTAGTTCCGAAAAGTGAAAAGTTTTCGGTACTGTAGCACTTTCGTTTGTTTATGACAAATAT

TATCTAATTATAGACTAACTAGGATCAAAAGATTCGTCTCGTGATTTACAGATAAACTGTGTAATTAGTTTTTATTTTCGTTTATATTTAATATTCCATGCATGTGCCACAAGATTCGATGTGACGGAGAATCTTAAAAACTTTTTGGTTTTCATGGTGAACTAAACAAGGCCCTAGCTGATATATAATCTTAATTTTGACCACCAGTTTGTACCATTATGAAGTATGAACGCAGCATGTAGCACACCAAACGCATGCATTCAAATGAACACTACAGCTAGAGCTACCGCCATGCATGCATGTTTGAATAATAATATATAGATTGATCAATAATGCAAGTTGGCATGTGTGTATGATCGTCTGCAGGGCGGCCAACAAGGGCAAGGGCAAGGGCGAGAAGCGGCCCCGGCAGCCGCGGTTCGCGTTCATGACCAAGAGCGAGGTCGACCACCTCGAGGACGGCTACAGGTGGCGCAAGTACGGCCAGAAAGCTGTCAAGAACAGCCCATTTCCGAGGTACGTACACAGAAGCTCTAGCTATCCACGAATAATTAGGGTTCTCACGAGGAGCGAGAGAGAGCATGGTGACTGACTGACTGACACGGGGAGTACAAATGTACAATGCATGGGCAGGAGCTACTACCGGTGCACGACGCAGAAGTGCCCGGTGAAGAAGCGGGTGGAGCGGTCGTACCAGGACGCGGCGGTGGTGATCACCACGTACGAGGGCAAGCACACGCACCCCATCCCCGCCACGCTGCGCGGGAGCTCGCACCTCCTCGCCGCCGCGCACCACCACCCGATGGGCGGCCTCCACCACGTGCACCCGCACTTCCGGATGGCGCCGCCGCCGCCGCCGGCGGCGCTCGGCGGCTTCAGGCCCGGCGGCGGCGCCAACGCCTTCGACGCGCTCGGACTCGGACTCCTGCAGCCGCCGTCGTCGTCGCAGCAGCAGCAGCAGGGCCACCATCACCACCACGGCGCCGCCGCCATGCAGCAGCTGGCCGTCAGCGGCGGCGCCGCCGGAGTGCAGCAGGTGAATGCTGCCGCCGCCATGGCGAGCCACGCGGCGCTGCCAGACGATGGTGACCAGCACGGCTTGGCTGCCATCGCAGGTGCGGCTGGTACTACGACGGCGGCAACTACTGCCGCCAGTGCTCCGCTCCGGATGCAGCACTTCATGGCGCAAGACTATGCTGGGCTCCTGCAGGACATGTTTCCATCCTTCGTTCACAGCGACGATGATGGCCACCATCACCACCATTGATGTGACGAGTGATGACTGAGTTTTTCTGCTTTAAACGATCGATGGTGACGATGATGGTCGATCGATCGGGAGCTAGTAGCCAGAGAGATAGCTGCTAGAGATTTGGTTGCTCTTAACTTTTTTGGTTCTTCGTGGTATCGTTGTACGATGATGTGATCTGAGGTAGCTAGTAGTATAGCATCATCATCAGTAGTATAGGACGGGCACCTATCCGGTACTAGCTAGCTAGCTAGGAGTTTGTTGTACATGTACCTGATTCCTAATGAGCCCATCAAGCTCTGCACTTAATTTCATTAAGCTGGGGGCATGCATGCAGTACTGTACTGTTGAGCAGTCGCAGTATTCTGGCACGTTACCCGTCTATTCTAATTATGCCATTTACTTCCTCTAATTCATGATGATGACAGACACATCACATATATATGATCGTGTG

TTGTTCATGCATAGGCTATAGCTAGTCAATTTCTGATGGGTCTGTTTCCTCAAAGAAAATTCTGATGGATCTACATCATCTGCATATGCCATGAGCCTGATCTCAGTATATATATATGGATAATTGAAACATGTGTGCTGCTCAAATTATTCTAGCTAGCTTATGGAGTAGTAATAGTACCAGAGAAGAACTAAAAATGAAA

>SbWRKY4

CCCAGTCCCCAGACCCAGCAGCACGCCGCTACAAAGGCACGCGAGCACAAGCAAAGCAAACCCTCGCTCGCAGACCTCTAGCGTAGCGAGCTTCTTGCTATCCAAGCCAGTGACACACAAGTGACAAAGAGAGTACTCGCTCGATCGCGCGACGCGCCGCGCAGCTAGCAAGCTACGGAGCTAAGAGATCGCATCGGTGCTAGCTTCCCATGGCCGGCGCCGCCGGCGACAGATCGGAGGACGTCGGCGCCGACTGGCCATTCGGTGGCGGCGCCGCCGACGCCTTCACGGAGTACTCGTCCGTGTTCGCGGAGCTCGGCTGGCCGGGCGGCCTTCTAGCCAGCGGGGAGCTCCCGGTGCTGGATCTGCCCGACCCAGCAGCACCGCTGCCGTCGTCGTCGCAGCTGCTGTCCATAGAGCCGTCGGAGGACCCGGCGCCCGCCCGGTCGGGCGACGCCGGCGCGTCGTCGAGCTCGAGCGGGGACGGCGACGGCGCCGCGCCGGGCAACGACGACGACGATCGGAAGGCGGCGCCGGCCGCCGAGGCAGCGTAAGTGCATGCATGCGCGGTAGCTTCTGCCCCTAAAGGTAGCATACGCCGGCACTTGGCCGGCGCTTAGCTCTCCGGTGAGTGGCAGGGCTGTAATTTCGGAGCACCACCGCACGAAAGCGAGCGAGGCTCCCCAACGCGTTTCCGTTTCTACTACGTGCACCGCGCGCGCCGCGCCCGGGACGGGAGGGGCAATCTCGTCAAATACCGGCGGCATGTCCACGCAGATCCGCGATTTGCGTGCGCGGATTGTTCCAAGTTTCACGCGGTTTCCTTTGTTTTTTTTTCTTCTCGTCTATCTACTCCCCCTTTTTTTTAACATATGCTGCATTTCCGTAGATTATCTGTAGACGACCGGTATTTCCGTGTACTGTTTACGTGTTCGTATGCATATGCACGGGTATTTATTTGTTTTGCTCGGATGCAGTTTGTGCTTGTGCATGATCTTGGTGTCAAGTAGATCCAAACGGATTCATTTCAAAGTTGCATGTGGTCAAACACTAATGGACATAAAGTTGTTCATCGAAGAAAAGGGAAAAGTGTACATACATGTATCATGTATGTCTACGGACGGAGTATGTGTCTATGTATTCTAGCAGTGATGGATGGATGGATGGATGGGTGGGCGGGGTGGACGGGTGGCTGTGTGTGTTTCTCCATGCCCGCTACCATTTTTCTCGCTGATCTGCATATTATCGTCAGCAACGTTGGCGGCGAGCGGCGTCCGGTGGGCCGGGGGTGCGGCCGGCGTGGTCACGTCGTCCGCCGCCCTGGTCCTGGTCGGTCCTGCCGTGATCGGCCGACCAATTTCCTTGTCTTACCTACCTTTGCCATGTTCAGTGCGCATTAAATCTTGCACTCCAGCCTACGCAGCATTAACTGCTTGCAGGCATGGATGGATCCAAACATACTGTTCCTGTCATAGCTGCTCATAGATCTGGCCACTATCACTATGAACTGCCGCCAGTACATGACATGCTTTATAGAAGGAGTACTCTTTCATTAGCATTTTTAGTTTTTCTTTTTGACGAAAACAAACACAAAAGTTTTCTGATACAGCTACTTGGACTTGGATTTGCATACGCAGGGGTAGGAAGCCGGCGGCGGCGACGGCGAAGAAGGGGCAGAAGCGGCCGCGACAGCCGCGGTT

CGCGTTCATGACAAAGAGCGAGATCGATCACCTCGAGGACGGCTACAGATGGAGGAAGTATGGCCAGAAAGCTGTCAAGAACAGCCCTTTCCCAAGGTTGATTTGCATTGCCAGCTTTATAGACTAACTTGAGTGTGCTACACTACACTGGTACTTTATTAGCTACATATCTTGCATTGTCTAGCTAATTTTATGTGCACACATACGTATATACGCATATCATCGCTGTCAGCCATTCAGCGGTGTTTTTCTCTCATAATAAATCAACCAACAGTACTTTATGTCATAGCTTATCAGTCAAAAGAACAAGGCGTTACCTTAGATTTCACTCTTGTCCTCGCTCTCATCCAAATGATACTGCATTGCTTGTGTTTCAATTCTTCTGTCAACAGGTAAATCACTCATATATTGTTCATGCTAAAATGACCCCCCTGCTGTTTGTGGTTTTGCTAGTTCGCTCTATACTACCATGACTTTTAATTTTACCAAGAATATGAAGTTAAGAAGAAATCTACATAATACTTTTAACGCATACATGAACATATGTATTTTTTTTGCATCACGGCATGCAATTAATTATAACTAACACAAGAGAGTCGAATCAAATTGGGCTAAATTTCTTGAGCAGGAGTTACTACAGATGCACCAACAGCAAATGCACGGTGAAGAAGCGCGTGGAGCGGTCCTCCACCGATCCCTCCGTGGTCATCACCACCTACGAGGGCCAGCACTGCCACCACATTGGTCCATTCCAACGCGGCGGCGGCGGCGGCGGCGGAGGCGCAGCCACGGCGCGCTACCACAGCGCGGCGGCCGTGGCACTAGCGGAGCAAATGTCTTCATCGTCGTCGTTCATCCCAGCGCGGCAGCTCTACAGCTTGCCGCCGTTGCACCCACCACAGAGCTCCCTGTCCTCAGAAGCCGTCGTTAGCTCGGCGGCGACTACATCTTTTCATCAGCATGTTAACGACGGCGACGAGCTGCGGCAGGCTAGCTACAGCTCGAGGGTGTCCATGGCGCAGTCGCCATCAACTCCATCGTCGGTGCCTCCGGCCATTTCAGTTGAGAAGGCCGGGCTACTGGACGATATGGTGCCCCATGGTGTGAGGCATGGAACACCATGATGATGCCAATATGCATGGTATATACTAGTTTCATAATCATGTTGTTTTAGTTTTATTCATGGCATATATTTCGGCCGAGATTCCATTTAGCTGAAGTTGCATAATTAAATTTGTGCGCTTTGCTTCTACTAACAAATATTTGCTATATATATGTCCTTAGTAGCTAACCTCAACTTTAATTTGATATATTGAACCACTTCATGATTTTTTTGCATACCCTTTTTTTTGCATAAGTATCTTTGCAATTTATGTTACCTTTATTTTTTAATGAAAGTATATGTTGGCAAGATACAAAAATCTGTTTGTGCATTACTACCCAGGTTTACAATATAAAATCGATATTCATATACCATCAGTAGCTAGGCAGTAATTTCCCTGGTTTTTGCATATGTATAGCTGGATGCATGCCTACCTTATAATTGATTTCATGGAGAAAATGCACGGCAAGAGCTATAGCATAGTGGTGTGTACGTAGTACGTTGCAGGAGCAATTGGCACTTCATGCATGCTAGCTGCGACACATATACATACATATATGAAGTATTCATGTCTGCTAGTACACTTGTGAGGGGACTC

AATGGATTAAAATCTTTTTCAAGGACATAATTAAACTGTAGTGCACAAGTACTAGAGTAGTTGGCAGACTAAAGCACATGTGTACAAGTACAGAGCATATATACATGTATACATGTGATACTCCCAAGTTTAGATTCTAGTAGGGAAGTTGTATAAAAGTTATTTTCTCTGTACATACCTTTTTATTACTTTTCTATTACATTTCCACTTGCCCCGGCTAGCTTTGACTTGTCAGTTCCCCTTCCCCACATGCTCATAGCTGAAAGCTTAGCTAGCACACACATGACACACATGACACATTAGTGGATTGAAAATGCAGTGCATACATAAGTAATGCCTTATGTTCCTTTCAATTGGAATCCTCTATAAAATCGAGGCGTGTAGTATATCAGAAAGGAAAGACGCGTGTTTTTGCGTTGTTCATACTTGTGCACATGGAGGACCATTTCCTATCTTTTATCCTTCAGCATATAGAGTGACTTGACTTGACTTGACCTGGAGTCTGATCGATCTAAATTCAGGTTCTCACTTTTTTCAGACTATCTGAAGTCACCTCTCGCAGGTCGGCACTACCAGGAAGGCAGAGATGAAGTGATCAACCAGCTAGCTAGGGTTTTCTCCTGCTGGATTCATCAGTAGTTAAATTTATATCTACATCATCTTGTAGCTAGAGAGTATGTATATATACTCTGCTCATTTGTCCCAGATTTGCTCATGGTCAATGGCACTCTCCAATGGACCTGCAGGTGGATGCATGTATAACAGTACTTCTACTTTTCATGGCGAATTTTTTATGCCAGATTAACTTATGTCTAGTTATTACATGGCGCTAGCTTCTGGTCAATGGCTTAATTAA

>SbWRKY12

GCGTCGACGCCGACGCGTGCCGTGTCGGTGGACGGCGGCGGCGCGTCGTCGGCCAGCTCCACCGACGACGGTGCCGCCGCCGCCGCCGCCGCGCAGGAAGAGGAGGAGGAGGACGACGACGACGACGGGGCGCCGGCGGCCGCCGCAGCCACCGAGGCAGCGTAAGTAGTTGCTTAGTGCTATATATTCCGCCGATCTTGTTCATGTCCATCTCCTCCTCCTCCTCCGACAGTCCGATCCCTCGCCAAATTAATTAATAAACTCGTGCGCATTTCACCTGATGATCCATCTTTCTCTCTCATATTCTTACTTTTTATTCAGGATTTAGTTTCACTTCAAACCTGATTTGTTTGTTTTGGTGTTTGCATTTGTTGTTACGTGCATCAAGCTATCAAAAGTTGCACATTTCACCTCTTTCTCCTTTTTTCCCCCTTCAATTCATTTTCTTTTTAGCACTCGTTTCAACCTTTGATTTGCTTCATCTTATTGCTACTGCTTCCAACCATTCTAAATTGCTTTTATTATGCAGTAAGATATACACTATATTTAGATGCATAATAAAATAGACGGCTTGTAATTTATTTAATTTAAAGTGGATGGAACATGAATTACTGATGGATGGCCTCTGCGCAGCGGTTGCAACGGAGGGGCAGTTGGGTAAATTTGCGCGGGAAGAGAGGGAGAGAGAGGGATGGATGGATGGATGGATGGGGACGGAACGGAACGGAACGGAATTGAAGTGAAGCGGACATTGCTCCGCGATTCTCGTGCGTCACAGCAAATCTATATAGCCACTACTCCTTTCACTATCCTACACAGTATATCCTATACAGTAATAATAATAATAAAATAAATAAACACAAGAGCCACGCGGCGCCTTCTCCTCCTCCTTCCTATTCTTACTACCTCATGCACCTTACCTTCGGTTTTATTTCTTTTGCTTTTTTATCTCCTTTTCCATCTTCACTACAACTACAAAAAACATAACTTATATCAAACAAAAATATATAATAGTCCATGCCTGTGATTTTTCTCCCATTTATTACTTGAGGAAAAAAAAACATGTATGTTCTCAAAAACAAAAAAGGGAAAAAAAACTTGTACGTATAATTTAAAATAAAGTGGAGAGAGTAGTACTTGTCACGTCATAATAATAATAATAAACATATATACATAGATAAGACACTGATTGTCAAGTCATGTTCTCCAGTCTAGTCGACAGAATGCAACCGCACGATGGACGATTCATCATATATAAATATATAATTATATACTAGCAGCATGATGCACAGCTAGCTAGCCATGTAAGGTCGCTCGGTGTTGCACGTACGTAACGTACCTTCATCTCATCACATGCATGCAACAAGCGCAAAGGCGGCCGGTCACCGGCCGTGCATATACCCTCAGCACGCAGCACATGGCGTCTTCACCGTACGTTAATTACCTTCGTTCACGTCACAAATAATAGAGTTACTTAATTATTAGTGATCATATGTAAAAATATGGTTGCAGGAGCAAGCCGCCGGCGCCGGGGAAGACGACGAAGTCGTCGGCGGCGGGGCAGAAGCGGGCGCGGCAGCCGCGGTTCGCGTTCATGACCAAGAGCGACGTGGACCACCTGGAGGACGGATACCGATGGAGGAAGTACGGACAGAAGGCCGTCAAGAACAGCCCCTTCCCCAGGTAATATAAGGCCTTGT

TTAGATCTAATTTTTTTTTAAATTTTGACACTATAGTATTTTCGTTTTTATTTGATAAACATTGTTCAATCATAGAGTAATGACGGAGAATCTTAAATTTTTTTTTGATTTTTGGGTGAAGCCTAATACACCCATGCATGCAATGTCTATGTATATATTTCCACTTCTCTCTCTCTCAGTTGTCTGAATTTCCTTTTTCCCGCTGTTCAGATAACATACAATTTTATCATTGTTGTTTGATCATACTATGATAGAAATGATTTTTGCTCATGGTCAAAAACAATTTTCATCGGGGCTGCTTTATCCGTCCCTAAACAGACATTTTCAGGGTCCATTTTATTATTAAAAAAATCTGCCACTAAAACACTACTACAGGAATCTTTTAATTATGAGGCGGTCAGAAATGGTTTTTCAAGGTAGATAAAAAACAAACGCCTCGGAGAAAAAGTCATGGTAAAGAGTAGGCTTTCACATTTGGGCAAACTGCTCGCCTCAGTAAATGAAATTTACAAAATAAAATAAATCTAGCCTGCGAGCCCAATGGCCTACTGAGCCTGCCCGTCGGGATCTGTCGCTACCGCCCGGAGCACCGCCGCTGGATCCAGTCGGCCTCCCGCAGCCGAATTCAAGCGCACGCCGCCTCATCGGGCTGCCCGCCACCGCATCCACCCTCTCGGTCGCCCGACACCGGATTTGCGCTCGCCCTCCACCGTTGCCCGCTACCCTCCACTGGGGGAGGAGGTGGCACTCCCCTCCACACCGTCACCAGCAACCGGACTACACTCGATGTAGTAGGAGGCGAAGGGGCGCCGGATCTATGGTGGTGGCGCCGGATTTGGGGTGGTGGAGGAGCGCCATCCTTGGGGAAGAAGGGGAAGGGTTGCCGGATCTAGGGTGGTGGAGGCAGCGACATCGAGTGTTGGGGAGGAAGGCAACCACGTCGGGTGCTGAGAGAGAGAGAGAGAGAGAGAGAGAGGAATGAGGAGATTAGGGTTTCCCCATTTATATACAACCACCTATATCCGGTTGAGATGAGTCTTTCTTGGGCTCGGTCATATCAGTTAATGATTTACCAAGACCGTTATTTTATAATCCGTGAATAACCGAGGTGGGCTACCAGGCTGCCCGCCTAGGAGCTCCAAACTGAAACGCCGCGGAAAAGATTTTCTGTATAGTAAAAATATGTTTTTAGGGGAAGATTGCTTTAAGAAAACTAATTCTGAAAATAGAAAAATAAATGTATAGCTAGTATACCGAGACTGCATGCACCATATACTTGATTTCTCTCTCTCTCCATATACACGTAGAATTTATAGTCCAATAATGGCATATGTATATATCCAGCAGCATTCAATTTGTACCTTTGTATGAATGAAATAATGAATCACACCAAGCATTCAAATATGTGCGTGGATCACTTAAAACTGTTACGGCTATTCAATATCACACGGATTGCAATTAAGTATAATACAAAGTACAGCTCTCCTACACTTAATGCCTACTTTACTCTAGGTTTAATTTATAATCATTTTGGTACTTTTAAACAAATAAAATAAAATTGCAACTGATCTGATCTGATTAGAATGCTGGCAAACATATAGCAACAATATAATTAAAAGGAACAGCTGATGATACATGCATCTGCATCTGAATCTCTGAAAATTGAACTCTAAAAGTCAAGTTATGGATGATGTACCACT

GTGTACCTGCTGCAGGAGCTACTACCGGTGCACCAACAGCAAGTGCACGGTGAAGAAGCGGGTGGAGCGGTCCTCCGACGACCCCTCCGTCGTCGTCACCACCTACGAGGGCCAGCACTGCCACCACACCGTCGCCTTCCCGCGCGCCCACCACCTCCACGCCGCCCTCGCCGCCGCCGGCCACCACCACATGCCATTCAATTTCTCTGCCGCCGCGCACCACCACCACCTCTACGGCACCACCAGCGGCGTCGTCACCGACCACGGCCACCTGCCGCCGCTGCTTCTCCCGACGACACCCGCGCCTCAGCACAACGCTCTCAACGACAGCGACAACAACGGCTCGCCGCTCGCCTGCAGGACGTCGACGACGACGTCGTCGCTGCTAAGGCCACTCGACTGCAACCACCAGGAGCTTCTGCTGGCGGCGGCAGCGAGCTACCCCTTGTCGTCCTCAGCTGCGATGTCGTCGATGCCAGTGCCTTCCATGTCAACGACGACGACGACATCGTTGCCGCCGCCGGCTAGCAGTGCCGTCGACAAGGGGCTTCTTGACGACATGGTGCCGCCGGCGATGAGGCATGGATAGTTAATTGATTACTTTAATTAATTCGATGGATCCATGCATCAGTTTATTTGTTGCCTATATTATTAATCTCTGATTGGACAGACAGATCAGACAAGAAGAATTGATTGTAAACATATACATATATAGGTATATATATAGAGTGGTGGTGTCAAATGTCAACTGTCTAGCAATAGCAGTCGGCAGCCTTGGATAAAATTTTAGTTACACAAGGTTATATATTGTTCCAACCAAGAGCTCTACTGATTAATATATGTTGCAGTTATTAAGCATGTCACATAAATCGGTTTGGCTTCTCTATCTATAATTTGGAACTGAGTGACTAATGACTGTGCACCTGATA

>SbWRKY25

GTCACCCTTTGCATGCTCTCCACAGCTGCTCTACCACCGCTTTCACAAAATTATTTCATTTTTTGTCCCTAATAGCTCCCAAGTTCCCCAACAACAACAAGATCGAAGCAGTTTATATCACACACGGACACACCTCACTTTGCTCCGAGATCGATCGATCCTCTAGCTACCGCTACTCCTGGGGAAAGCAAAGCAAAGCAAAAGCCAGAGAGAGAGACGACTGAGAGACAAAATAGAAAGGAGAAGAAAGGGATCGAGGACCATGTTCCCGTCGCCAGGGAGGACGGTGATGGCGCTGGGCCACGGTGGCCAGCACATGACCTCGTCGTCCACCGCCGGAGCCGCCGGCGGCATGGCGGCCGCCTCGTCGTCGTCCACCCCCACCATAACCTTCGCGTTCCAACCGTCCCCTCCGCCGACGAGCGGCCTCGCCCTCGCCCACCATGGCGTGCTAGGTTACGGCTCCTCTTCCCTTCTCCTGGACCACCACCACCACCCAACAACCACCACCACCTCCTCGGCGGCCTCTTCCTCGCACGCGGCTTCGTCCATCACCCTCCACCACCACCTCCATGGCCATGCAGCAGCAGCAGCGCCGCACGCTTCCCTTTCCCCTCCCACGAGGGCGTCGCCTCCTCCTCACCCATGGTAAGCTCGGCTCGCTAGCTCATCATCAGATCTCTTTTCTCTCCCTCCGATCCCCGGCGCCGCCTACTTCTCATCCTCCATGTCAATTAATTCGATGGATTAGGTCGACGACGACGACGGCCTGCGAAGAAGCAGGAGGGCCGGCGCCGGCGCATGATCGCCAAGCAGGCCAACAAGGCGGGAGGCCGCCGAGGGGGAAGGGAGCTGCTGCGGTGATCAGCGAGGGGTCGGCGGCGGCGGCGCTGGGGGTGGGCGCCGTGAGGATGAAGAAGGCGGGCGGCGGCGGAGGAGGAGGAGGAGGGAAGGCGCGGCGGAAGGTGCGCGAGCCGCGGTTCTGCTTCAAGACGATGAGCGACGTCGACGTGCTCGACGACGGCTACAAGTGGCGCAAGTACGGCCAGAAGGTCGTCAAGAACACGCAGCACCCAAGGTATATATGTACCTCCTCTCCTCTCCTCGATCTCGCTCTCATCATCATCATCATCATACGCATGCATGTTGGGTTTGTGCACGGACGTCGAGGACGCACTAGCTAGCTATAGATGCATGGCATGCAGGTTCTAGCACATTAATTATTACATTTGCTCGCTCCCGATCAGCGATAATTCACGTACATCTCCGTTCCATGCCATGCCATGGCTTGTTAACGATTACATGCACATGCATAATTGCACTGCTCTTTAGTTTCAAATCTGTCAAGCACGTTGTTGATTCCATGAGATCACCCTCTCTTCAATTCCTTTGCAAAGTCGTCATCGATATATATTTTGAAGATTTTGGGATGCTTTTGCATTTGTGAGTCGTGTGAGATCAGCTCAAATTAACTTACTATAAGCATTATATATGTCCGTTCCCGTTCTAGATATATTCATGTAGCATGGAGTTAGTTTTCATTAATTTGCTCCTAAGACTCCTAGGCAATTAAGCTAATCACCCCCCCCCACACACACACACAATTCATAAAAAAGGTAATTAATCTCCCTGAAGGCCTGAAGAATGGTTTTGTTAATTAATTACACCAGCTATAAATTAAACTTCACATATTCTTTGGC

CAAGGATATATATAATAGTTTAAGACTAATTTGGTTTTGTATATACAACTAAATTAGTTTCCTTCTAAACTGATCCACATTAATATAAGCAACAATTACTGGATTTAATATGCCTTGACAATATGAGCGACACAAAAAAAATCTTAAATATAAAACAAGTAGAGTGGGGAAGAGAAATTCTGTGCACCATCTACTTGCAGTGTTAGATTGAAGTTACATTAAAATAGAACCAACTAATAATCTATATTTACCGTTCCTTTTGTTTACTAGGGAGACTGGCTTGCTAGTGTTAACTCAAATGTGAATATTACTCTAATTCTTGTGGTTTTTCTGATAAAGTGTAATGTGATAAAAGGTTCTTATAGCCGGATATTGTTTTATCCCTTGTCTAAAAAAATATGTCATAATGCAAACACCACATACCTCCTTTGTGCCTTTTCGACTTTGATAAAAATTCTCTTTTATCTTACCTTCTCCGGTAAGAGAGATTACAACAATGAATTTACAATTTATTCACGGGATGGTGTGCCAAAACTTTTATGGACAAATGATACCAAGTATAATCATTCACAAATATATCTTTACTAGCACAAAAAAAATTGTTGATTTCGCATGTGCTCTAGCAAACACTCAGAAATACTTTTTTGCCGCGCCAAAGGTACAATTTCTGCTATTGAATCTAGTCAAACAACTTTGATCATATATGCAACAAAACATATGGCACCGTATCTTTTGATCCTAGTTAAAGAATAAGATTAATTAAAATAAAATGTATTCTTATTATTTATCATGATCGACGATGCTTTTATTTATGTAAGTAGTTATTTCTATGGACAACAAGTGTGACCCGAATTGTGATTGGAACCATGTTATATTTTGAAACAACTGCATAATATATTTATGCAGCGACGGAATTAGAAGGATCATGAACTTATTTTGTTGAAAACCCACCAGATTCATGAATTCATTTTGTGTGATTATTTGCAGTAATTTTATTCTGTCAATATATTCGGATATTTGTTTCTTGGGAAGTAGTGATAGTATGTAATTTTGTATACGATAGTAATTTAGCGCCGATCAATCATTTGTTCTCAATGGCCATTTTCATCAAAAGAACAATATCAGTATTTACATATATTTCTACATTAACTAAGAACCAATCGAACACTAAGGTGCAAGATACATTGCAATGTAACATTATTAGTTCATCATCAGCTATATGGCTAGTTAATAATAATCGATCATAACATATTTTTCTAACTATTGCTCGAATGTATTTATGATATGGCTGTGCTTTGAATTGTTCAGTGCAATTTCTTAGCATAATACCAGCCTAGGGTTGTACCGCCAAAAAAAAAGGAGAGAAGAAGCAAAATGTTAAAACCTGAATATCCACATAAAGCTTATTTTGTAAATACAGAGTTCAGTGCACTTCTGCAAGAAGCAAACGAGAGAGCATTTTCACAAGCCTTTCCACTCTCCCACAGTTGCTTTTTCCTAAAGTGCTAGTCATTGGATCTGAAAAGGAAAAAGTTCTTGCAAAGCTGGTCCTAAGATGACTTTGTTGTGTGTCTGGTCCCCTTCCTGTTTATCCCATATCTAATTAACTAAATTCAATTCCCCTAATATATATCATGTAGTTTCTCTCATTAATTAAGGATAAGATATTTTGCTCAGAGTAAAAGTAACCATCTGTTGGT

GGTGACATTCGTGTGCCTGATATTTATTTTATTTAATGAAGGTATAACATATATCAGATAATATATATATATATATATATATATATATATATATATATATATATATATATATATATATATATATGCTAATAATAAGCTTAAGGATCACCAGTACATTTCATGTGGGTCCTGAATATTGGCTCTTGCATTGTGCCCCAGTGCATGTAGAGAGAATGACGTGCATCCTAACAAATAGTAGTAGCAGTAGTAGGTTGAATACTCATTGCACCATCTAACCCACTAACGTCCACTCGTCCATGCCTACTACTGTAGGCAACGGCGACTCTAAAAAATTTTGCAAAATTTTTCAGATTCCCCGTCACATCGAATCTTTAAACACATGCATAAAATATTAAATATAGACAAAAATAAAAACTAATTGTACAGTTTGGTCGAAATTGACGAGACGAATCTTTTTAGCCTAGTTAGTCCATGATTGGATAATATTTGTCAAATACAAACGAAAGAGCTACAGTGTCGATTTTGCAAAATATTTTGGAACTAAACAAGTCCCAAGTATATATGGACGAAATCACATTTTGTTGAGAGTGCAAGTATATTAAATAAAAAATTTTATTGTAGTTATTCTAAAAACAAATCACTTGTTGCTTATTAGGAGTGGACACGTACGTACTTGTAGTCACAAGTAGTAGTAGGAAGTTAGGCCCTGTTTAGTTCGCAAAATTTTTCAAGATTCTCCATCACATCGAATCTTTGATCGTATGCATGGAGCATTAAATATAGACGAAAATAAAAACTAATTGCAGAGTTTACCTGTAACTTGTGAGATGAATCTTTTGAGCCTAGTTACTCTGTGATTGGACAATGTTTGTCAAATAAAAACGAAAGTGCTACAGTAGCCTAAAACCCAAATTTTTGCAAACTAAACAAGGCCTTAGTCACAAGAAGTAGAGATGACATGACAATATATGACATGATCTATATCTTCTCACGTGAGCAGAGCGAATTAACCAAGCTGATCCAAATGATGAAACCACCACACGTACCTAGAAGCGTGTGTCGTGTGTATGCGTGTGAATCCGATCGATCCCTGAATTGGCTATCATGGCAGGCAACGCCAAGCATTGACCCACCCACGCTGCAGCAGACATGAGCCCAGCATCCAGTCCAATCTGATTAGCCATCTGCAATGATGCCACTAAGAAATAACCCTCCTCCCCTACCTCGGTGTCTGAATCACACCATACCCATGCCCCTTTTTTTCCAATCAGTTGGCTAATCTTGGCAATCATCCATCTTTTTTAAAAAAAAAACTCCATAAATAATAAAAAAAACTCAACGGGACGGTTAAGATTGTCATCATAGCTTTCAAATTAAGAAGAAAATGTCTTCTCGTCCGGCGAGAAAACCCATAAACTCCATCTATACACGATGGTCTTTCTGTCGTCCAGTAAGAATTAGGTTCAGAGGGCTCTAGTACTTTAGTACGAGGATGGGTCTAAAATTTGCTCTCCCTGAAATTCGAACTCAGGAGATGCCGTCGGAGTGTCTAAACCAACTGATCTAGCATCATTTGGCAACTCCGTAATAAATAATAAAAAAAGAGCATATACATATACATAAATAATGGATCACACTCCGCAAAATTGTGAAATAATATATAACATGTAGTTTTTTTACAAAAAGGAAAATATTACAAT

AGTCTTTATTAGTTTCTCCCGTGTGGGATGAAGTGGCTGGCCGGCCTCGGTAGGAGAGAATCTTTACTGTTTGGTAAAGGGCAAGATGATGATGAAAAGGTGGGTTTGAGCTTTGCAAATTGGGCCCCAAGTTTTTTTGTTCTTGTAATTATTACCGCATCTTTATCAATCTACTAGTATGGCCCTTAGGCTGAGTGAAATGATGCTACCTGGGTAGCCCGGAATATTATTATTATTACTCACTCAGATCAGTATCTCTACATTTTCCTTGTTGATGTGTCAGGCCATGATTGAGATTTCCAATCGGTGTACTTTTAACTACCAGCTACTAGCAGGAGTTGTACATAGGAGCAGTACCTCTGTATAACTATATTAACAATTATTAATTATATGATGATAATTATACTAGTAAAAGTGCTAAGTGCAAACTAAAATAGAGCAGTACTAGCTAGTTGTAGAGTTGTAGTGAACAAACTATTAACACGAGCGATCTACAGTCTACACTAGCTTGTCTACTGTGTCGTGTTGTGCCGTGTCCCATGCACATGCATGTGTCCATGTGTGTAAAGAAAAGTTAGCAGTGCTCAAAGCAAATATCCATGAGAGCTGTTAGCTAGGTTAGGTCAATTAGACAAGCTAGCTACTATGCACGCCTTTAAGATTGAATTTATATTAACGTTGCAGCAAAGCTACATGTCTGGAATTAATTTTCAAATATCGAAAATTATGGTGATGGTCGTAAGAGATATAAATTGATCTAGGCTTAATAAGTCTTTTTTTTGCGAGTAGGCTTAAGTCTATTTGTTGGCTATACTAGCACATTTTGTCACAGTTTATGTGGCGACCGTTTGATTTAAGGCCAAGCTTGTTGCGGCTCTCTCGGGGCTCCGTCTCCTCAACTGTGTAGCAGGAGCCGAGAAGTCGCAAGAAGCTAGCTCTATAGTTTGTTTGGCAGAGCTCTTCTCCGGCTCGGGCTCTAGTTTTTGGAGAAGCCGGCCACCCTCTCGTCGTTAGCAGCAAATTAAATGACTAATCAAACACCATACCATCACAGTTGATCTATCGTCTATATATACAAATGCATGTATATATAATTGATTTGCATCCATTTTACTATTAGTACGTAGAGTACTTGAATTTATGATATGATGCATGCAGGAGCTACTACCGTTGCACGCAGGACAACTGTAGGGTGAAGAAGCGGGTGGAGCGGCTAGCTGAGGACCCTCGCATGGTGATCACCACCTACGAGGGCCGCCATGTCCACTCCCCGTCCCGCGACGATGACGACGACGCTGCGCGCGCCAACGCCGAGATGAGCTTCATCTGGTAGTAGCCTGCAAATCCGGCCATCTCAATGTCATCGTCGTCGTCGTCGCCAGTAAAGTTTCGCCAGCCAACCAGCACAGCTGATCAATCCTGAATTATATATCCATCCATGCAATCATCGTGTGTGTCTAGCCATTGCAAATGTTGATCGCTGGTACGTAGGATGTAAACTGCACATGCTCCTACTGTTGTAGTTAATTAGCTAAGTAGCGCGCATCAAGGTTATGGTGGACCAAATAATATATATAGCTAGCTGAAACGTGTTCTTCTAGAATTAGCAATGTTTATTGCACTGCCTAGTAATACTTGCTACTTTAAATTTAAAAGGTTAATTAATAAGTTTGTTGATGATATGATGCTAGGTTGTTCTT

CCACCTTCACTAGTTAGGTTAAGGTGTCATCAGTAGATATGGCCATATATATGGGTATATCTGCTGTGAGCAACTTGTGGAATCTTCTGTGTAGAGGATAGACATCCTCGACAATATATATAATAATGTGTATGAATGTATAATGC

>SbWRKY49

TACCCATAGTACTCGATCCTCTCAAGCGGCCATATAGCATAGCCCACACCACACCACTAACCCCAAAGCATCATCGTCATCGTCAACCTTATCGTGCACAGTTATTGACACCCGAAAGGGGCAGAGATCCATTCCGATCCATCCTGCGAATTTTCTGCCCCGCCCAACCAAGAACCAGATCATCATCAATCGAGATCGCTAGGGCATATATGCAGGCATATATGGAGGGAGGCCAGTTGAGTGCTTGCCTTCCTGGCTTCCTTGTGCCGGATCACTACGCCTTCCCTCTTCCTCTCCCGCTACAACTTCCTAGCAGCCAAAACAAGCTTTTCCAGATGCCGTTTGTAGTTGACCAGGAAGCAGAGACCGAAAACCATGGCGGCGGCGGGATGCTCTCCTCCGACCATTGTGGACTATACCCGCTGCCGGCACTGCCCTTCGGCAGCTGCTCCGGTGCCGCCGGCGCCGCAACAGCGTGCGGTGGGAAGCCTACGGCCGGTTTCATGCCCAGTGCTATTGTCGCTGAGGAGGTGAGGTGATCTAGCTCAGCTTGAAGCTTTTCTGTCAGCGTCCTTTCTTGCTTGTTGTTCCTTATGAAAGCTTTCGTTCGTTCTTCCGCAGGTCTGCACCTCGGTGACTACTAAATTAGGTTGCAACGACAGTAATGGCACATGGTATGCTCTATCTCTTTGTGATATTTCTTGAATAAACCTTTGGCTATATATAGTTAATTAGGGTTTGATGGTGTGTGCAAGCTAAGTGGGCGTGTGATTGTGCAGGTGGAAGGGTTCGGCAGCTACAACGATAGCGGAGAGAGGGAAGATGAAGGTGAGGAGGAAGATGAGGGAACCGAGGTTTTGCTTCCAGACCAGAAGCGACGTGGATGTACTGGATGATGGCTACAAGTGGAGGAAGTATGGGCAGAAGGTTGTCAAGAACAGCCTCCATCCAAGGTATCTACAATCCACATCATATTCCATGAATTAATTTGTGTAGAGGTTGTTATCCATGACTAGCTACACACTCCATGCATGCGCCGTGGCATGCTTTTCTTTTGTTTATGAATATGTGGAAGCTAGCAGATTAACAAGTAAACCTTAGATGATTAATTTTATGTGTTAATGATCTACAACCCCTAGTCGTCTAGTTGTTGTATCGTTGTGCTCAGTCTAAGCTTTCTTCTTTTTAATAATATAATCGGCAGCTCTCTCCTGCTTGATTTGTTTTTTTTAAAAATGATCTACTCCTATAAGCAGTACCTTGCCTCTTGAAAGATGCCTGATAGATGGTATATATATAGAGGGTCAATGTAGCTGGTGTGGTGGTGATCGATTAACAAAAGTCCTGGTAAACCTTATTTAATTTGAGACACAGTAGTAAAGCCCGTTCGCGAAAAAAAATTTAATTTGAGACACAGTAGTATGTTAACTAATTTGTAATGAACCACATGTGTGCAGTGTGTGCTAGCTGCTGGTTTTGCAATTGCTTCCAGTTCATGTATCTATTAGCTAGTAGCCCCCATGCGGCATATGTGAAACTAAAAATATATAGAGAACTGGGGAGTTTTTGTGTCTTTTTTTTAGGGAAGAGTTTGTATGTTTTATTCACTCCAAACGCATGCTAGAATTCGAGTTACATTTCATCTCTGTTTTTGCAGAATTATACAACTATATATATAGGAGTAATAATGATCTCTTTTA

CACGGTTACAAACACATTTATGCTAATAACAACTACTTGTTTATAAATTATAATTCTGCCTTCCGGTTTTCTTTCTCTACTAGAATTAATTGAAGTGGTACGGTATTAGACTTCTTAATTGAGCATATAGCGGATTGTATCATGAGTGAAATTATTGGAAGGTACCATACGTTGAAGAAGATAACCAAAGAGTTGGGAAAGGAAAAAGATGTGTCAGTGCATATTAATGGCGCTGATCGATCGAGTTAGTTCTGAACCGTGTATAGGTGGGATAGACACATAGATAGTGGATCAGAAGTTCAGTCTTTCAGATCATAGTGTCCCATGCCTGGTCTACCGTAGGCTACAGCAAACAGAACGAACCAAGTGTGTGCACATGCACATGCATACACAGAAGGTGTATCAGAAGTGTCCCACTGTCTCTAAGATCCTAGCTTGTGTAGTTGTGTTTGTTCAAAGATAAGATGTACACAAGTATAGCTAGCTAGATCAAGCTAACATATCTAAGAGATCTACATATGCACCACGATGATAATCATTTAATTTGGTCTAACCTACAAATGCACATGGTCTGAATACTACAATCTTGATATATTGCCATAAGAAATGCCTTAACAAAGTTAACACTTACATACACAGAATTAATTAAGTTGATTTGTACTGTATGAACTACCTCTGGTCACTTGATGCTTTGAACAATATCCAATCATCGTTCTTTCTAAACTTTGACTGTGTATAGTTTCCATTGTATTCAGTTTGCACGCGTAAAAATCATGTATAGCTTCATCTTTAAAAAGTAACTTTGATTATCCCATACATTTTTTAGATTACAACTGCTTTCTTTTGTTTGAAATTACAAGACATCATATAATTTGTTCAAATAGACAAATTAGTGGCCAAAGTTTCACCTCAAAGAGTGACAACATGATTTTTTTAAGCATTAGTCAGAGCATACTATATCACACTTCTCTTGTGCAAAGAGACATCGGGCCTGTTTAGATCGGCTAAGCCTAATTGTTAGCTGCATCCAAAAATTAGCATGCTAACTATATATTAGAAAGTGCATATATATATATATATATATATATATATATATATATATATATATATATATATATATATATATATAACCTCAGCTAATAGGTGCAGACGGCATTGATACATCCCTAGCAATGTACTATTAGCTCTCTTGTTAATTAGACGACTAGTCATCCCTAATAATAATGTTCATACATGACAATAACATACTTCACATGCAAACCACTATATGTCATATTTCAAGGTATAGGAGTATGTATATATGAGTATTCCTAATTAAACTACTCCCTCCATTCCAAATTATAAGAGTTTTGTTTTTTCTAAATATATTATGAGTATTTCTAAACTACTCCCTCTGTTCTAAATTATATTGTGATACCTGCGTCTAACGCAGAAACGTTTGCTTAGATCTACTTAGGTGAGATGCTCACGAGAGATCGTACACACCGCATAAGAGACTTGATGTTTGGTGCCATAGGCTTCGGCCAAATCTTGTTATTATAAGAGCTTTGTTTTTTCTAGATACATAGCTATATCTGATTAACGCCGCCGCCGCCGCCGCGCACCTTGTTTGGCGCTTGGCATGTGCACATTGTGGCAGGAGCTATTTCCGGTGCACTCACAGCAACTGCCGCGTGAAGAAACGGGTGGAGCGGCTGTCG

ACGGACTGCCGCATGGTGATGACCACGTACGAGGGCCGCCACACGCACTCTCCCTGCAGCGACGACGCTTCCTCCGCCGACCACACCGATTGCTTCACCTCCTTCTGATGATCAATCTGTCTATCTGTCCCACGACCGCATATATACATCGACGACGCTAGCTGTAAGTTCTGATGAATTGAAGCAGCAAGGTGATCGAGGGCAATATATATATTGATGATCTCGATCTTCAGTTTTGCCGGATGATATATATGGATCGGATGACACAGAACGTGATCGATGAACCCGGCCGTCTCATGCATGCGTACTACTCATGTCCGTATATATGTAATCCGGTCGATGTGGTATGATCATCCGCCAACCGTACCCGTGCAAATGATCCATCCGTCGATGTAAGCGGTCATGTAGTATTATATCAAATAAAGCACACTTTTTTTTTCACTTAGAACGATTTGTACTACCGAGGGACGGAGCAAAAAGAGTATTGTTTTAGAATTTTAGATTTAGATATTTTATTTGACCATTCGTCTTATTTAAAAAATTTATATAAATATTATATATATTG

>SbWRKY60

CTAAAAAGACAGAAATCTTCCTCGGCATCTCCCATTCTCACGACACTCCTCATGCCCCCAATCCCACCTTGATCCTGATAGATCCATCGATCAATTGATCACCACCACAAGCCACACCTGCACACGGTCGATCTTGTTATATGCATGCGTGCATGGAGGGGAGCAGCCAGCTGTTGGAGACCTGCCTTCCTGCTAGTAGCCTCTACGCGCTCAGTCCGCATCATCCTCTTCTTGCCCCGCTGCCGAACCAGCACAAGCTTCTGCAGATGCCGTTGGTCCAGGAGCAGGCTGCTGCGAATAATCATGGCGTGATGCTCTATTCGGACCACCACCACCACGGCGGCGGCCTCCTGTACCCGCTGCTTCTTCCCGGCATCCCGTTCTGCCCCTTCTCCGCCGCCGCCGACGCCGCCACCTGCGATAAGACCACCACCACCGGCGGCTTCGCGGCGCTCGATGCCGGCGAGGTAAATGAAGCGACTCCCGTGATCCCGTCTCGTCCACCCAGCTGGTTAAATGACTTGCTGTTGCTGATCTGGGCTGTTCCTTGATCGATCCTTGTAATCGCTCGGTTTCCGCAGGCGGGCACCTCAGTGGCGAAAGCCGCCGGCGAGATCGCTAGTACCACCACCACATGCAACGGCCCAAGTTCCTGCAATTGGTACGTATGACTATGAGCTCACCGTACCATAGCCTACTTGTTGATTCTTGCACGCTTGGGAGCAATAGTGGAGTAGCGTGGTTGACAGATTGTTAGGGTTTCTAGTGCGGGGCGGAGGGGGTCGTTCGCGCTGATGAACTGATTGGGATCACATCTACTACATCTGTTGATGAGGTGAGCACGCAGGTGGAAGGGCCCGGCGGCGGCGGGGGAGAAAGGCGGACGGATGAAGGTGAGGAGGAAGATGAGGGAACCCAGGTTCTGCTTCCAGACCAGGAGCGACGTGGATGTGCTGGACGACGGCTACAAGTGGAGGAAGTACGGCCAGAAGGTTGTCAAGAACAGCCTCCATCCAAGGTACGTATAGCAGCAATATATATCATATATGACCATCCTAACCCCTAATGCCTCTTTCGGAACATGAATTTTCCACACTGCTACTTTAGGAATTGAGTGGTTTTTTTAATAAAATTCCTACTACAGATTAAGCTAAACATACCGTTTTCTACACAAACAACCCAAACTCAGTTCATGGGAGAGCCTGAAAGGTGCTCTTCTCCAGCTGATGCAGGATCTTGTTTCCTCTCGATCGATGCTAGGATTTTTTTTTTCCGGTAACATGCTTAGCACGTGTTTCATTAAGAGGAGTCCTCGATGCTAGGAAGGGTCAACACCTTAAATCTTACATGTATTATGCGTGAATTAATTTCGTACATACTCCCTCCGTCCCATACTCTCTCCGTCCCTGAAAGCTTCAATTATTAGAATCCGTGTCAGTCAAACTTGACCAACTTTATCGAAAAAAGTATCAATATGTATAACATAAAATGAGTATCTTTTGAAAATATATTCAATGATAAATCTAATAGTATTAATTTGGTATTATAAATCTTAGCTTTTTTTTCCTGTAAATTTAGTCAAAGTTTTAAAAGTTTGACTTAGAACAATTCTAGAAGTTGCACCTTTCATGTACGGAGGGAGTACTCAGCATTGTTTGGTCAGCTGTGTTATCACTTTATAAGGCTATAATTTGTCAAAT

TCTGAAATCAGCTAAACAGTGAAGATTGAAATCAAACCAAGAACTTAGGTTGACATCCATAATTTTTTAAAATTTATACAACATGTACATATTTTCTTTAACTTTTTAGACTGGCCATACGGAAAATTGGTTGTTTTTTTTTTTGCAAATAAAATTTGCATTCTATATGAGCAAGCAATTTTATATTCATGTCTATATGGGAAAGTGGGAACCATGCATATTTCACATTTTCAAGATGGAGAAAAGGGCGGTAATCGCAAAGATTGATGCCTAGTTTCACTTAACGTAGGAAAGATTGATGCCAATATGGGCATTATTTATGATATTGTTCAACAAGCTTATCCATGTGAAAGATCAAGGTTGCTAATAATATATATCATATGGAATAGTTTCCAAAGAGTGAGTGTCGTCCAAATTGACGTGTTCTTAGGAATAGAAATAGTGTGCTTGGCGATACTTTTATTTGTGTTTACAAGACAAAACTCTTGAGTTTTGTGACAAATATGCAACATATTATCATGAACCAGAATGAGTACCAAATTATAGCTCAGGACATCTGGGGTCTATAATAATTAAGTCACTTAATTTATGGCTAAGTTTTGGTGCATCATCAGCGATAGATGATGTCTAAAAGGCAGCTCTGCTCTGTTGATTTTATTTAAGTGTTTCTCGTTTGTTTCACCTAAAAATGTCATGTGCATCCACCAAAATGTTGGCGCAGGAGTCACACACCAAGTAACCTTTTTTCCAAAGGAGGAGAGTCACAATGGAATACTAGAAAAATGATAGGACAGATGCATTGTTGCTGGAACTCTCAAAACACCAGTTTAAAGGATCTATTAAGAGCATCTCCAACAACTTGGTAAAATTCACTTGTCAAATCTTGAGTTATAGCAAGTTGCTAATTTGTTTAGCAAAAGAAAAAAAGATGTGTCTCCAATAAGTTTCTATTCTCACTTGGTAAAAATAGAGGATGACAGATGAGACCCGCTCACTTGGTAAAAGAATATGTGTCTCCAACAAGTTGCGCTCGTGATAGCAACTTGGGATAGCAACTTGACAAATCTCGGCAGTAGAAACCTCTGGATAGCAACTTGGGAAATTTAGCAAGTTGAGATAAGGAGTTGTTGGAGGAGGATTTTTATGCTTTTAGCAAATTTGGTAGGATAGCATGTTGAAATACCAAGTTGTTGAAGATGCTTTACATTAAAAAAACGATCTATAAATATATCTTTTAATTTTCCTCCTTGCTTGGTCTAAGTTCTGATCATGCACCATCTACGGCATGTCAGCATGCACTAGTAAAACATATCATCATTCACGATCTGTAAAAATAGGACACACGCATGAACAGGAACGAGTGCATATGCATGCGCTGGGACAAATGCCAAATGGTGCACACATATTCACAGCGCAATTTCTTCAGGCACTCAATGTGCCGTGGAAAGGCAAGCCGGCCTGTCCATGGTTGTTGGCCCGTAGTCTCTCTTTGAATTTTGTTCAGGCTACATGTTTTCACTTGTAAGTGTTTCCTTTAGGTAGAGGGTATAGAGGCTGAATCTATGCCTGTTCATATGTTTTCATCTCATGAGAATGCTTTTGAAAAGTGCCAGAATACAGTCACGCCCATGCACGTACACTACCAGCCAATTAAAATTCACAGAAAATACAAATAGTAGCCTAGTGTACAATCAAAACTC

CACACATTCAACCTACCTTGTAAAATTATCGTTCTGAAAGAATTCTTTTTTCCTGAGTTCTCATGCATAATTGAAAGAAAGGTTTTTAAAATCATGATTCTTGGTAAGATTTAATTCAATTTTGGTAAACTTAGGTGGATTATTGGCATTGTTCCAATCCACCTAGCCTTCAATAATATTTATCAAAACCCAATCACTTCAAAGGTCATCTGTATCAATATTATGTATCGTAAGTCTAATGATCTTATGAAATTTGGGATACATGGTGGGAATGGTATCATTTCGGCTTGGTAGGATTGTAGTATTGTAAGATATAAGATTCTAAGATCACAGGATCCTGACAACCTTGATTGAAACCACATATTTGGCTCCCAAAAGTTGATAACTTGTGACTATTTCCGTCAGATACCCAGCATGGTGAAATATTCATCAAGCATTTTTTTTGTGGTTATACTCCCATCCCAAAAGAATGCAATTCTAGGTTTGGGATGAGTCAAACTTATTAAGTTTGACCAATTTTATAGAAAATTGTTCCAACATTTATGTATTCAAGTAGGTTTACTATTTTATAATCAATCTAGTGATATTTGGTATCATAAATGTTAGTATTTTTTATAGTCTTGGTTAAATTTGAAATTAGAACTTCTTGGGAAGTGAGAATTGTATAATTTTTTTGGGGACGGAGTAAGTACTACGAAATTAGTCTATCATAGTATCATCACACTGAAAAATCTATCAGTAAACCATTATATTTCCACCAGAAGCATGCACTAGATTAATTTGAACAGAAAAAGAACCTGGATATATTCAGTTTGACAGTGCTTACTAATGCTTGGATCGATATTCAGAGGCCAGTTAAGGAGCTCTTCCTCTAACTTGGTGCAGCTAGATTCATATACAAGCACGGATTGCTCTACATGTTGATATTATATTTATGAGTTCAAAGCTTTTTAAACTTCACAATTTGTGGTTTCAGTTCGCTGGTCAGCGCCTTCTCTAAATTATCACTATGCATGCGTACATCCAGGAGCTACTACCGGTGCACCCACAGCAACTGCCGCGTGAAGAAGCGAGTGGAGAGGCTGTCGGAGGACTGCCGC

ATGGTGATCACCACCTACGAGGGCCGCCACACGCACTCCCCCTGCAGCGACGACGCCGACGCCGCCGCCGGCGACCACACTGGCAGCTGCGCTTTCACGTCGCTCTAGAGATCGATCGACGCGGGCGTGCAAAGCTTAATCTAGCTAGCTGCCGCCCATATTATATTGATTTATACATGCAGTGCATGGATCGATCGATCGAGCTGTTTGCCCTGGGGAAAGCCTGATAATGCAAAGCTCTGTGCATCAGAACACGAAGCTATTAGCAGCAGTAGTGCTGTATGTAGAGACGATGGATCGATGCGCCAATATATCCATGCATGCATGCAGGTTTGTAGAAATTAAGTAACCAATCGATGGATCAGCAGTAGCTGCAAGGTCCATCCTATTCTCTACTGGAGACTTTTGTTACATGCATGATGCGTCAGAACATACATCTGATGCTGTTGGAACGGGGTACGCGTGCCTGCTTAAAGCCTTTAGACATGGCTGCCATGCATGTAAGGCACAGAAAGCGAGGCCGGGCGGCCGGGCCACCTTGGTGGCTCGCTGCTGTGGCCTGTAGGCAGTACACACATTGGTAATCACATGGGGTAATTGTGCGAAGAAGGCAGCTCGTTGCTCATCATGCGTGCAGCTGCTAATGATTGCGGAACTGCACCCCAGCAGTTGCTGCGAGCAGGCTGCAAGTAAACGAACATGAGCTCTCAGACAAACTTCCATTCACATGCTCATGCTCTACTGATGCTCGTGTATGTCTGACTGAAGAATGCATCGGGAAGGTAGAACTTGGACACTCCTATTCGCGGGTCGGGAACGCAGCAGCTTGTACATAAATACTAGCACAAAAAGATGATAGATCCCTTGTCTGAAATTGTTACCACAAGAAGAAATATGAGTTTCAACACGAATATATCGTTGTGATAGAAATAGAAAATGTTACGAAATTAAGTTGTTGACAAGGATGGGTACCTTTATGATGAACATGATTTTTTTTTGTCACAGAAAATACAACAGCATGAATGGGTACCTTACGGTGGACACGAACTTCTGTCACAAAAAAATACAATAGAGCCCATGACGATTATATAGATTGGTGATGATAAAAAACTATTCATTCGGAATTGTTCTTATTATTAATTAATTTACTATACACCTATATGACCAATGCACATATGTCACAGATTAACTGGTAGTGTTCTGAGACCACCTAAAGAGATATTTCACAAACTCGATTTTTTTTTGAGCTGGTCTATGGTGGAGTGGTTGTGGAGCAGTTCCAAGTTTTTTTTTTGGCAACTGTCGAAATAAATAAATAAGTTATAGATACGTTGGGCCAACACTTACTGGCCCAATTTTCCTGGCCTCCAAGTTGGCCCATCTCGTATGACCCCGCTACACACTCTTAGCCCGGCAGCCCGCCTGTGCCGG

>SbWRKY35

GGCTTTGCGCGTGCACGTTTACCCAACTAAATCTCCTCTCTCATCTCCTGGTGCCTTCTCGTTCACTCACACTTCAGCTAGCTCTGCAAGGACCGTACGTTGTCCCTGTGATCTGGTGATGAGTTGCATCCGATCCTTCTATAAATCACAAGACAATGTTGTCTCAACTCTCAAAGGCGCCTACACACGTGACGACGTCGGCACGTCCCACTAGCTAGAAGAAGCAAACGCAGGCAGTAATGGAGAACCAGCATCTCCAAGGAGACGAGTCGTCGTCGTCGCACGCGCTCCCCAGCTTCCCCTACTTCGCCGTGCCGTCGCCGCCGTATGCGCCGCCGCCGCCATCGGAGGACCAGCACAGTACCCTCATCACAGCGCTCCAGCAGCAACCGTCGTCGTCGGCGTGCAATAACGACGACCTTCCTCCTCTGGGTCTGGGGCCTGATCAGCTAGCGGCGGTGGCGGCGCCGATGATTCTCCCGCCAATGGTGGACTGGTCGGCGCTGCTCCAGCAGGCCAGCTTGATGGGGCCCCAGCTCGTGCCGGGACTACTGCAGCAAGTACCGCCCCTGGAGCCGTTGGACCAGAGCGGGGAGAACGACGGCGGCGACGCAGGGAGTAGCAGTAGTAGCAAGGAGAAGGTGGTGGCGAAGGGCGGCGGCGGCGCGGGGAGGTCTGGGAAGAAGAAGGCGAGCAGGCCGCGGTTCGCGTTCCAGACGAGGAGCGTCAACGACATCCTGGACGACGGCTACCGGTGGAGGAAGTACGGGCAGAAGGCCGTCAAGAACAGCGAGCACCCCAGGTTCGATTTCTCCATGTTTTCGTTTTTCAATCATTTCATGCATGCATGCATTGCATAGCCGCCGACCTTGATCGGGGTCGGGCGCATGCATGCATGGCGCATCCATGCACACACGTGGATCAATTATTGCATTCATAAATTGCATATAGGAAGGACTTGTTTGGCACTGCTCAACTAGTAAAGCTGTTTTTTTAGAAAATAGCTTCATGAGTGACTTTCTAGATGAAACTGAGCTGTTTTGGAAAAAGTGTTTGGCAAAATAGCTTCACCAACTCCTTCATACATAGTTGAGAGAGAGAAATGAGGGAGAATGTGTAATAAGCTACTTTTTTCCAGCTTCATCCAACTTATATTTTTGTGAGAGAAGGGGAAAAACAGCTTCACCTATAAAGCTGTTTTGGAAATAAGTGTTTGGCAAAAAAAAACAACTCACAACAGGTCACGAAGCTGTTGTGAGCTGTACCAAACAGGCCCGAACTTGCTGAAATATTTGTTTGCACTGCACATGTGTGCATGCATGTAACGAATGCGTTTCGACGGGCATGCATGTTTGCATATATGCATGCATTCAAGTGTCATCAGGTCATCAGTTGCATCATCTCTAATTCCATGCAAATTAGTACAATTTAGAAAACAAATTAATGGAGGTTCAACGCTGCCGAACTAATTTGCATGGCAGCATGGGTACGGTCGCGCGCGCGCGCGCGTGTCAGTGTGTGCGCTAAGCCGTGTGTGTAATTCGTGTCTATTTAAACCCCCGCCTAGTATTGTTGTTCGCCAAATTTCTATCTACACATCTACTTGCTTGTCCGCATGCATATTAAAATGACAACGTACACATGTGGTTCATCTATAGCTAGTCGATGGCCTGGAGAGACACTACTTGTGATGCCATGTA

CTTCCTCCATTTTAAATTATTATTGCTTGCTTTTGCTTTGTACTAAGTCAAACTTCTTAAACTTTAGCTAGGTTATAGAAAAATCTATTATGGTCTATAAATTTAATTTTAATAAAATTCTGGGGTGGGATAAAACCATTGTTCCACCAAAAAAGAAAGATCTACAAATTCAAATAAATGGCCTATCAAGACATACTCACGTAGGTTTAATGGAACTATTTTGGTATCATAGATGTTGTTGAGCATTTTCCCATAAACTTGACTAAAGTTCAAGAAGTTTGATGTAGTATAAAGCTAAAATTAACTATAATTTCAAAAGATTTGGTTTTCATAGAGGAAAGAACTTAATGGAACTAATTGGGTATCATATATATAGATGTTGTTGAGCATTTTCCTATAAACTTGATTAAAGTTCAAGAAGTTTGATGTAGCATAAAGCTAAAATTTCAAAAGAAGAAAGTATTAGTGGTTTTCATAGAGGGAAGAATTTAATGGAACTAATTTAGTATCATAGGTGCTGTTGAGCATTTTCCTATAAACTTGATTAAAGTTCAATAAGTTTGATGTAGCATAAAGCTAAAATTAATAATTTTTAAAGAAGAAAGTATTAATGGTTTTCATAGAGCAATAGATCATGCAACTTATGCATGCCATGTTGGTTAAAAAAATGTGTGCATACCATTAGTACATAAACGCTTTTTTAATGGTTTTCATAGAGGAATAGAGCATGCAACATATGCATGCCATTAGTACATAAACATTTTTTTTACAATGCCTCCCATTTTTTTCATCGGCGGTTCATAAAGGACAGACACCAATAGAAACAAAAGTATGAGCACATTACTACGAACTGTCAGTGTAAACATATCTATACCAGTGACTGGCTTAAGGGTCTGGCAGTGTAAATCTTAGATTTATGCTACCGACAGCCTTAAGAAAACTACCAGTGGAAAATAGAATATATATTGCTGGTAGCTTTAAGAAAACCGCCAGCATAGGTATGTTTGCATTGATAGTTGTCTTAAGCTAGTTACTAGTACAAATACTTTGATTTTTTTAAACAGTCTTGGATGGATAATCCAAACCAAAGTTATATATAGTGTATGAAAAGATCTCAAACTTTATCCTGTTTATTTTTTATTTAAATCATTAAATACAATAAAGCTACTACCGGTGTTGATACATGCACGGGGAAGCTATATGTTGATACATGATGTACACAGAGTCGAGTCACTTGAGTAAAAATCATTTTATCTAAAACGATTTGAAAACTAACGGCCGCCGCTGCTGATACATGCATGCATGCAGGAGCTACTACCGGTGCACCCACCATACGTGCAATGTGAAGAAACAGGTGCAGCGCCTGGCCAAGGACACGAGCATCGTGGTCACTACTTACGAGGGCGTCCATAACCACCCCTGCGAGAAGCTCATGGAAGCGCTCAGCCCCATCCTCAAGCAGCTCCAGTTCCTCTCGCAGTTCTAACCGAAGCTGCCTCTGGTCATTATTATGTACAATAATACATATACACAGGCACGGCCACCAAAAGGTCAAGCAGACAAACATAATAATATATATACTCCTAATGCACAACTACATATGTGATATGTGTACTGTAGATAGTATTACACGAGAGAGTACAAACGTAAAGTGTAAAGTAACCAACTGGAAGGGGGAAGAAAAGGAGAAAAAAATGTGC

CTGCCTCCCCAATACTGCTGATCTCGCATGTATATGTCATATATACCTTGTTTATAAAGTTTACAAATATGTATACTGATAAGGTGGGTG

>SbWRKY82

ATGGAAAACTCGCCGCTCCATGGAGTCATCCGGCAACCAAACACTCCGCCGCTGGCTACGGGTACGTGCCTTGCCCCGCTGCCGCCGGCCGTAGCCGCGCCACAGCCGCCGGAGCAGCATGCGTGCAGCAGCGATGCGACGACGACGTCCCTCGTCCCGGGCGCGGCCACGATGATGAGTTGCCCGCCGGCGGCCGTGGACTGGGCGTCGCTGCTTCTCCCGCGCGCGCCGGGGACGCTGCACGTCGGGACGACGCCGCCGCCGGTCGCGAGCGAAGTCGAGAGCGGCGGTAGTAGCGCTGTGACGGTGGCCGGGAGCAGTGCTAGTGCGACGGCGGCGGGAGAGGGAGATAATAATTATAAGGCCGGAAAAGCTGGGAAGGCCGGCGGCGGCGGGAGGGGGAAGAAGAAGGCGAGCCGGCCACGGTTCGCGTTCCAGACGCGGAGCGACAACGACGTCCTCGACGACGGCTACCGGTGGAGGAAGTACGGGCAGAAGGCCGTCAAGAACAGCGCATTTCCAAGGTGTCTTTGTTTTGTTCGATCGTCTCCATTGATCATGCTTGCATGCATGATTGCAAGTTTGCAACTGCATTGGCGCTGCTTAATAAGAAAACTTGGAAATAATTAGTGCAATGTGTTTCATATGCATGCAGGAGCTACTACCGGTGCACGCACCACACGTGCGACGTGAAGAAGCAGGTGCAACGGCTGGCCAAGGACACGAGCATCGTGGTGACCACGTACGAGGGCGTGCACAACCACCCGTGCGAGAAGCTCATGGAGGCACTCAGCCCCATCCTCAAGCAGCTCCAGCTCCTCTCGCAACTCCAGTCTTGCACTAATCAACTCCTCTGA

>SbWRKY57

TGAGCCAGAAATAAAGTAGCAGACGCTGCCCCGGCAGGTTAGCACATTACCATTATACTAACCCTCTATATAACCCTGGTTGTAACTTGTAAAACTCTTTCAGCCTCCCCTCTCCCTCTCCCTCCCTCAGAAAAGCTCTCTAGCTAGTTTCCGCGCGCCCGGCCGGCACGGCGGCGCAAACTAGAGCCATGGAGAATTATCACATGCTCTTTGGGGCGGCGTCCACGCACGCCTCATCTGCTGCCACCCCCAGCTCGTACAACTTCATGGCCACTGCTGCTGGAACCAGCGGCGGCGGCGGCGGCGGCTTCCACGACCACGATCGAGGCCAGCGCAGCAGCGGGCACGGCGGCGGCGGATCGTCGTCGTCCTTCTTCGCGGAGCTGTCGTCCAACAACGACGACTCCAAGGAATATGGTGGTGCATCTAGCCCTCCAGGTCCAGCTGCCGGTAGTGGCCGTGGGGAGTCGTCCGTGGGGCCTGCGGCGGCCGCCGGCGAGGTGGACAGGCCGCCCAAGAGGAAGGGGGAGAAGAAGGAGCGCCGGCCACGGTACGCCTTCCAGACGCGCAGCCAGGTCGACATCCTCGACGACGGCTACCGGTGGAGGAAGTACGGACAGAAGGCAGTCAAGAACAACAACTTCCCAAGGTTAGTTCGTTGCTGCACTGCACACACGATTCATATATATATATATACATATATATGTATATATATATATATATAGTACAGTCAACGATCAAATTCTTCCTCGTATAAATATAGTACGATCAAAATGTCTGACGATGTTTCTCATGATCAATGACTACTATGCATGCCGATCCATTTTGTGATTGATCGATCGATCGGCCTGCGATTTTGATTTTCTGTTGCTGCTCGGGGATTTATTTTGATTATTATTACTGGTTTAATCTGGTGTGCGATTTGCTTGTTTTTTTTTTAAAAAAATGTTAGTATAGGGTTGGGCCGCAGAAGGTAGGTGAGAGGTGAGATGGAGTCCCAGTGAATCTTCCAAGCTCTGCTAGTGGTTGCTTGCTTATACATAGGATAAGGACACATCTCATCTCTCGTCCATCCTCATCATCTAGATGAAAACGACTCCTAGTTTTCATTCACATGTTTTTGCTGGTGGGCATCGATCATTCAATAAATAGGAGCCGATGGCGATCGAATTTGACCGGAAGTTATTTCGTCCACCCACCGGTTTAACCGCAGCATATATTCTATTAATTTCACTCTTGGATTTATCGATGCTTCGTTAGCTATTAGCTCAAGCTAATTCTTCCGTCGCTATCTTTTATATGCTTCAGAGAACAAAAAGTGGTTTCTTTGGACATCGAAAGGATAAGGGCACTACCTCTTTTTCCCCAATTTTTGGGCCATAAACTTCACAGATTCAGCATCAACTAACTGTTGCTGGAGGTTCCTGAACGCTCCAAAGACTTCAAAAATTTTACACAAGAGTAAATTCGATGCAGAAAAATAACTAACTATATATATGGAGTAAAGAGAAATGGCCAAAAGTAAATCTACTAAAACACCGTGTCTGATAAAATGCAATGATGTAGGAAATTAGCGTGTTCTGTTTTTCTTTAAAAAATTACAGCATCAAAAGGTGCCACTCCAAAAGCACTTGGTGCCTTCCTTTCTTCTTAAATTAATTAGGCTTCATGGACATGATTCTACGATATTTATTTCCTCT

CTTTCTTTGCTCGATTCCTTTTGTTCTTCTACAAAAACTTTGCTCAGACAAGCATGTAGCCGATATGTACTCTACGTTGAAACGTTAAGTAAGAACAGCATATGCCTATACACATCATATGCATCGAGGATAGATGGAATGCATGCTTTTGATCATGTCTGTGCCAGCCATCTATGCTAGCTATAGCAAAACACTACCAACTGAGAAAGCCATATATATGTACAGTTACCTCATGAACAAAATTTGATGCATACACATATAATAGTATTATTACACTTTTAAATTCTTATTCATCACTTCCATTAGCTGTCGATTGGAGGGTATTTTTTTTCATGACGAACATAGATAAGATTACAACTGTGGCTTTAAAACTATATGGACAAATTTAACATTTTGCACTGGTACGACTTGAGCATATATCATGCCAAAGTAACAGTAATGGTATGTGGTATGAGTTTGGAAAAAGAGATATATACTGAAACTTCTTAATTTCCAAATTGAATTTGGCGTTCTTTTAATGAAAGAATAAGAATATATATAATAACTATCATTGAGGTATATATATCTTATATCTACAAAATTCTGCAACCCCTGGGCATAATATCTCACAGATCAATACTTATATACATTTTGTAAACAAAAAAAAAGTCCCTTTTACTTGTTACATATTCACAGAGTTAGGCATGCCAATAATACATTCCTCTATCTTATTTCTACGTGCACCAACATAATGAAAACTTAAAAAGACCAATTAAGGAGCAGCAATTAAACAGTACATTGTTCATCGAAACGAAGACATATATTAGACATTACCACAACTTGAAATTGCAGATAACTTATAAATTCTTTTTACTGAGAAATGATTGCATGCTTCTCCACCAAATATATATACTGACATACTTAATTAGCCAAGCAACAATGTAGAAGCTAGATCCATACACATATAATAAACCAAACATGTTCAACTTGTGTCCACCCGTTTGAACATACTCCTATACATATGCATCGATATACAGGCATATATATATATCTCTCTTTCACGCACATTTTCACTTTGTTTGTTGCTATTACTACTTTCTTAATTCATGCACCCGGAATAATTAATGAACTAATCCTTATTCTTTTCGCACGTATTTATGGATGCAGAAGCTACTACAGATGCACTCACCAAGGGTGCAACGTGAAGAAGCAGGTGCAGCGGCTGTCAAGGGATGAGGGCGTGGTGGTGACCACATACGAGGGCACCCATACACACCCCATTGAGAAGTCTAATGACAACTTCGAGCACATACTCACCCAGATGCAGATCTACTCCGGCATGGGATCAACCTTTAGCAGTAGTAGCCACAACATGTTTCACTGAATGAGCCGTCCAAAATCACTTCAAGCATCCGTGCACCGTCCAAACTAAGAAACAAAATCGATCCTTAGATTTTATTAATTTGTACATTAAGTCTTAGATGTTTAAGAGATAAATAAGCGTGATTCGGTAGCTAGATGCAGCCATGCAGGTATTATACTCACGCAATGGTTCTTTTGCTGTTGATCTTCTTTATTTTTAAATTGCAATTGTAGGGCAGGGATAGAGACTGATAAACACCCTCAGAAATATAAACATGCTGGATTACATTGCAATTGAAAATCTGATACCTCTCAGACCAGGGCATCACCTTAAGTGTACTT

>SbWRKY23

ACGTGCAGACGCTTCTGAGAATCGAACGACGACGAACCTTCTGCGTGCGCGAGCTGCCGCTGTGTGAAACATCCATGAAGAAGCCTAATGGCTAGCCCTGCTCGTTCCTCACTTCGCGATCGCTTCAGAGACTTGAGCTCTCATCGCCTCATCTCATCATCAGCTTCAGCTAGCTAGCACCACAGACGACGATGTGCCAGCGTCGTTCGGGCGCTAGTATAAATTGCCGAGCTTGACACGGTGATGGAATATATATCTATCCCCGTCCCGTCACACGCAGCGCAACGGCAGCTATAGCAGCAGCAGCACCGCTGGTTCGCTCCACAGTCGCGCCGGTGGTGTGCCGTGTGCGTGCGTCTACTCTACATGGCGGCATCGCTAGGACTCGCCCACGACGCCAGCTGCTACGCCGCCTACCCGCCGGCCGCCGCCGCTGCCTCCTCGTACTTCCCATCACCACCACCACCCGGCGACCTCGTGGCGGAGTTCCCACCGACCGCCGCCGCCACGGCCATGGCTGATGACTACTACTACTACTACTTCCAGTTCGGCGAGGAGATGGGCGGCGCTCGCGCTCCCGGCTGCGGCGGCGGCTACTGCTCGCCGCCGGCGCCGGCGTTCGACAATGGCATGAGCCTGCTGTAAGTTTCCGTACGCGGATATAACTACTGTGGATGGGGTATATATACGTACATGTAGCTTCTTGCGTGCGATAATTAATGTTCGCTGTCGCCGTCGTCGGTTGTGGCAGCTATGCTCATCCCATCAGTTGCGTGGTTTGATCGGATTCAGGAGCTATGGCGGCGTCGACGGCGACGGGAGGAGGCCGATGAGCGGACCAGCTGCTGGCACTGGTGGGAACGGCGGCGGCGGCCGGCCGCCGGCGTCACGGATCGGGTTCCGGACGAGGTCGGAGGTGGACGTGCTGGACGACGGCTTCAAGTGGCGCAAGTACGGCAAGAAGGCGGTCAAGAGCAGCCCCAACCCGAGGTACAGCACAATACAATACGACTCCGGCGATCACTTCAGTTCCATTCCATGGTCACGCTCATGCATGCATGCATGACGTGCTGATCGCCGCCGCCGCGCCGCGCCGTGCGTGCAGGAACTACTACCGGTGCTCGTCGGAGGGCTGCGGCGTCAAGAAGCGCGTGGAGCGGGACAGCGACGACCCGCGCTACGTCATCACCACCTACGACGGCGTCCACAACCACGCCGCGCCGGGCGCCGCCTACCTCTGCCCGCCGCCGCCGCGCGGCGCAACCGCAACCGCAGCAGCGCCCTGCTTCTCGTCTCCATGCTCGGGCTCGGCGTCGGCGGCGCTGGTGGCAGCACCCAGCTGGAGCGGCGCTTTTGACGCGTGGGAGGCGCAGCTGGCGGCGGCGGCGGCTCACTCGTCGGAGTCGTCGTACTGATCCCTCATCAGTTACACGCACGTACGGATCTTGTAGCTAGAATGTTCACGAACGCGTGGAGATCGAGACGATCGATCCCTGCTTTTTTTTGGGTTTGGAGTTTGTACGTACCATACTGTCATTACAGTTAGACATACATACACCGAGTCTATTTCTGATTAGCTGAGCGTTCCCTTTGATTCAATAATACAGGTCCACGTATATATCTCGCTGTACTGAATACTGACAAGCTGAGCTGCCAGACTTTCCTAGGGAAAAAAAATCTATGATGAATAACAACAGTCA

GACTTTCTGAAACTGATATATATTGTGTCATATTTTCAGGGAACTGATAAGATCAACAGCATATACACTAGCAAAACAGAGTCATATTTTGCGAAGTGTATAATGCAAAAATATAAGTTAGAGCATGAAACTCCAATCATCCCTTAGTCTGTTGTCCTCGTCTTCTCTTATGAGCAATTTTCAAATATGGTAGAGTTGCTTGACTTGCATTGGTTCAGATAAGAGTTTTCAGGTGCTGTCATCTGTGACCAATGTTTTAGTACCATAGCGAAAATGAAAAGAAACGAAAACCCTATGTCGGGAGCTGGTAAAATCGGCAACATGTATTTTGGAAAGTACATATATTGCAAACAGAGAG

>SbWRKY77

CCCGGCACGCCAAAAATATTATTCAGATCAGCAGGCACATTAACTGATACACAAGCAAGGCGGCTAGCTACAGCTAGCTGGTGCGTGCAGTGCATGCAAATGGCGGCTTCCCTGGGACTGAACCCTGAAGCTCTCTTCGCTTCATACTCGTCTGCCTACTCCTCCTCCTCGCCGTTCGTGTCCGACTACGCGGCGAGCTTCCCGGCGGCCGTCGACTCCGCCACGGCCTTCTCCGCGGAGCTCGATGACCTTCACCACTTCGACTACTCACCGGCGCCGATCTTCACAGCTGTCGGAGCCGGGGCTGGCGGCGATCGCAACGAGAAGATGATGATGTATGTATATATATATATATATATATTTATGCACAGAAAATTATTAGGGTGTGATACTAGTTATTAGCTAGCTGTTATGTTGGTGCCTCTGTTGCTTATTAATTATAGTTTGCTTTGGTGATTTTATTCATCAGGTGGTGTGAGGGCGGTGGTGATGAAAAGAGACTCAGAAGCAGTGGAAGGATCGGGTTCAGAACGAGATCAGAGGTGGAGATCTTGGACGATGGATTCAAATGGAGGAAGTATGGGAAGAAGGCTGTCAAGAACAGCCCAAATCCAAGGTACACATATATATCTGTGCAGATTTCATTCCTAAAAAAAAAATCTGAGCAGATTTCAGTCTCTCTAAATAATAACTAATCTCGGGTTTGAGTGCCACCGAACAGTTAAATTCCCAGAACAAAGCTCAGAAAGCAAAGAAATGAATCCTCGCAAAACAAAACAAAACAAAAAGAGCAAAGAAATGAAATATGTCTATTAATTACTCGATGCTGATATAGCTTGTGCATGCGCACTTGTGCAGGAACTACTACCGCTGCTCGTCGGAGGGCTGCGGCGTGAAGAAGCGGGTGGAGAGGGACCGCGACGACCCCCGCTACGTCATCACCACCTACGACGGCGTCCACAACCACGCCAGTCCCGGAGCCGCCGCCATCATCCAGTACGGCGGCGGCGGCGGCAATAGCGGCTTCTACAGCCCGCCGCACAGTGGCTCGCCGTCGGCTGCCTCATACTCGGGCTCCTTCGTCTTCTGACTTTTCCGGGCCTTGACCTTGACCCAGAGTGATTTACCTTTACCCCAGTCGTAGGATGAGAAGAGTGTTCAGAGAGATCAGTTCAGCGGCCGGCTAAATTCCGACCTGCTTGTACATTTTTACACACTTGTGCAGTACAAGTAATAATATTTAAGCTAGAAGCAGTGTACTCTACTTTATTTACCTCAATTATTGGTTACCTCTTGTGATGATGCATCATCAATCATCACTAGGATAATTGATGACAAACATGTTTTTCACTTAATTAGTTTCTTTGTGTACACGACGTTTGTAATATGCCGTATAGCTGAAAGGTTGCTGATTTAGCTTGGCTTCTCTTGTTCGGTATTTGATACCATGATCAACAATTATGCTGAACAAATGCATTGTA

>SbWRKY33

GCCCCGCGCGCGCGCGCGCGCCCACGCAACCAAAATCCAACGACGACGGCGTCTGAGAATCTCAGCTCGCTCATCGCCCGTCGCCCGTGCTATAAAAGCGAGCGCTTCGGCGCGCATCTGACTACAGTTACTCGCAGCTGGCCCCCCGGTTGGCGGCCCATGCACATGGCGCTGTCGTCCCGCAGCTCGTTCGCCGCCGCCGACGTCCTCCTCCCAGCAGCCATGGCGTATCGTCAGCCGTGCAGCGGCGGCGGCGGCGGCCCTGCTACCTCCAGCTACTTCGGTTCTCGGCCCGCGGCCCCTTTTTTCCCGTTCGGTACGGCGGCGCAGCTGGACGTCTTCGAGTGCCTGTCGGACGAAGGCGGCGCCGTCCCGGCACCACCGGCAGCTGTACCTGGTGCGTTCGCGACGCCGCCGCCGCCGCTGCCGCTTATGCCGGCCGAGCGCGTCGTCCCGGACGCTGCTGCAGGCTATAGTAGTCATGCTAGGTGAGTTTCATGTCCAGACTCCAGACTCGTGTTTCCAGCAGAGACGTGGTGCACTTTTGTTCGAAAAAAAGAAAACAAAAACAACAACACTTCTGCTGCCTTTTCTCGTGAGCCAAAACGTGCACGTATATCATCAACTGGTTCGTGAACCTGGACGACGTGTATTTCCAGGAGTGCGGCGGCGGCGGCGGCGGGTGAGGGGCCGCCGAGGAGGACGGACAGAATTGCGTTCCGGGTGAGGTCGGACGACGAGGAGGTACTCGATGACGGCTACAAATGGAGGAAGTACGGCAAGAAGTCCGTCAAGAACAGCCCGAATCCGAGGTAAATTTTTACAGTATTTCGTAGTTTGTGGCGCAAAGTGCTACTTTAACACTGATAGTTTAATTTGTAAATCCAAAAGTGAGAGATGAATGATCATGAACTAATTAACAACGATGTGTACTACATATGCATGCTGGGGACTGAACCTTGCAACGACTTGAATTGATTTGCAGGAACTACTACCGGTGCTCGACGGAGGGCTGCAGCGTCAAGAAAAGGGTAGAGCGAGACAAGGACGACCAGAGATATGTGGTGACCATGTACGAGGGAGTGCACAACCATGTGAGTCCTGGTACCATCTATTACGCCACCCAGGACGCCGCCTCCGGACGCTTCTTCGTCGCCGGGATGCATCAGCCAGGCCACTGATCAACCAACCAGAGATCAGAGCATGTTATTAAGTAATTATTAGTGTAAGAGATAGATACACACTGCACACTGACTATCTTTAGGGTAGAGACAAACCTACTCAAGATATATAGAAACTGACGTGACATGTCGCTTATAGCTTGTCATATCTCTCTCTATCGGTCCCTTCTGGTTTAACGTGGGGCTAGATGATTTTGTGCTGCTACTAGGAGGCTAATTTTAAGAGGAAAATGGTATATGTGTTCATTTGAAATGTGCTACAAA

>SbWRKY83

GCCCCTGCTATAAGTGGCACGCCGGGCGGGCAAGCGGCGGCGCGCGGCGACCCCCGCAGCAGCTCCGAACCTTCTCCCAGCCATCCAGCCCAGGAAGCTCACCGGGAAGCCGGTGCAAAACCCCCCCAAGATCACATCCCCGCCATCCCCCCGCTACAAATCCCCCGGTCGACGTCCCCCACTCCCCCATGCGCACCGGCAGCTCACCGATCCCCGGAGCCGCTACTACTATAAATACAAAGCTTCCCCGGCGGTGGTCTAGCCGGCCATGGCGGCAGTCGGAGCGCGCCCAGTGCTGTACCACCACCCGGCGCCGGCGGGCGACGCCGCCTCCATGTCCTCCTACTTCTCCCAGGGAGGCAGCTCCACCACCTCCAGCTCCGCGTCCGCCAGCTTCTCCGCCGCGCTCGCGCCGACGACGACCACGCTCGCCGAGCAGTTCGACATCTCCGAGTTCCTCTTCGACGACGCGGGAGTCGCCGGCGCGCCAGGCGTGTTCGCCGATGGCTCGGCCCCCGTCGTCGTGTCGGATGCCGCCGCCGCTGCTGGTGGTGGTGCAATCAGCGCAGCCGCTGGGTGAGTGGCGTGCGTGCGTTCGTTCGTGCGCGTCGATTCTCTCGACGCTTGCTTAATTGGCACCTGTTTATGCTTGGGGCTTTCGTTCATTTGGTCTGAGACTCTGAACTGAACCGTATCCGCCGGGCTGTTTGCGCTGGGCATTTCAGGAGCGCGGCTGCGGCGGCGGAGGCCGTGCCGGAGCGGCCGCGGACGGAGCGGATCGCGTTCCGGACGAGGTCGGAGATCGAGATCCTGGACGACGGCTACAAGTGGAGGAAGTACGGCAAGAAGTCCGTCAAGAACAGTCCCAACCCAAGGTATGGTATATAATTGCGTATTTGTGTATCACAATTACACATACTGTACTATTCACCAAGAACACCACACAATTAGTACACGCATTTCGCGATCTGGCTGCAGCCATCTGGATGCCGGATGGATCGACGATGTTGCTGGCTGCTGAATGGCTGATGACTCACTCACTTCACACATGACATGCTTGTGCTCCTCGCCCGCGCAGGAACTACTACCGGTGCTCGACGGAAGGGTGCAACGTGAAGAAGCGGGTGGAGCGGGACAGGGACGACCCCAGCTACGTGGTGACGACATACGAGGGGACGCACAACCACGTCAGCCCCAGCACGGTGTACTACGCCAGCCAGGACGCCGCCTCCGGCCGCTTCTTCGTCGCCGGCACGCAGCCGCCGGGCTCCCTCAACTAATCCCGATGGCCCACCAGCGTCGCGACGTTCCGCTCCGGTCCACCGCACAGTGTTTCGATTCCCAGATGGCAGCTAGCTGAGCTAGCTACACTACTACTAGTAGGATAGGCCGATACCCGATAGGTGATCAGCTGGGTTTCCCGAGGTGGATTTCTCGTTTGGGTTTCTAGCACAGCTCCTCTCTCTTTTCCCCTCCGGTCCTTTTCGTGTTCCGATCGTGGAAAGGGCCCGGCGGCTGGCTGTTGCGACTTGCGATCGAATAGTATGTAGTGGTGCTGCTGCTGTTCATGCTGGGGTCAAGAGGTTTAATTTCCCCATTGCCTTGAGCTCAGCTAAATTAGTCCGGGAAATGTAGCAGATACTGCATTGACCGTGCAATAAGATGTACAGAATCTGATACTTATATTAGAATACATCTTT

TGTTTGCAGCGCAACGGTGCAAGTGTGGAATGTGCGTGTAAGATTGCATATACTCC

>SbWRKY27

CGCGCGTGCATGTCTTTATTGGCGTCTTCACTTGCTGCCGTTGACGGCCTGCCCAGGTCCGCTCGGGCAGGATAAGAATAAGAGGCACCCTTCTCCCGCTTGCTCGTTTACCTCTTGGTGTTGCCGAGCTGTGACATGGACACTGGCACACGCGATTGATCGGTTGCTTTGCCGGCCGCTTCTCCGGGGGACCGGACGGGGCGATCGATCCACCGACCCAATGTCGTCGTCCTACTCGTCACTCCTCTCACCGAGCCGCGCCCACGCGGACGATCATCGGGTTCTTCTCGGCGGCGATGCCGACGACGACGACATGGCGGCCGTGTCAAGCTACCTCTCACTCGACGACATCGTAGATGATGTCGTCGGCGGGGAGTGGTACCGCCCGCTGGCGGAGGAGTCGTCGTCGGCGGCGGCGGCTGAGTTGCAGCCGGAGCCACTCTTGTTTGCCACGCTGCAAGCGGAGGATGGCTACTGCGTCAGTGGCAGCGGCGGCGAGCAGAGCTCAGCGGCACTAGCCAACGACAACCACGACAGGATCGACCTGACGCAGTAAGCTTTTGATTGTTGATCAGTAAGCTGCCCGAGATGAGATGATCCTCTCTGTTAATTTTATTTAATTTCCTGACAAAGTAACTAAACGTTTGCTAATTTGCTGAATACATGGAAAAGGGACGGAGGCTCGAGGAGGCTCCTGAGGAGCGAGCACGGCAAGATCGCCTTCAAGACGCGGTCGGACGTGGACGTGCTGGACGACGGGTACCGGTGGCGCAAGTACGGCAAGAAGCTGGTCAAGAACAGCCCAAACCCGAGGTATATATTACTATATTATATCTATCGACATGTATATTACGTATCTTTTACTCTGTTCAATTATCGTCTCTCTGATGAACATGAACATTTCATCATCCAATTAATTTGCTCGGGCCAAATCAGGAACTACTACCGGTGCTCCAGCGAGGGGTGCCGCGTGAAGAAGCGGGTGGAGCGGGAACGGGACGACGCGCGCTTCGTCATCACCACCTACGACGGCGTCCACAACCACCCGGCCGCCGCGCCGCCACGGTCGCCGGCCTATCGCCTCGGGGAGCCGCCGCACGGGCACCACGTCTAGGAGCCAGGCAACCAGCGTCCAGCGGCGGCGGCACCGCGGCCGTCGATCTCTTGGCGCGTCTAGAGCTCTGTTCTTTAAACCCACTCTTTAGATCATCAGCCTTTCTAAAACTCACTCATTATTTCCTTTTATTTTTTTTTAAAAAACACCACTCTTTTAAATCCTTGTTGAAGAGCTATTCTAATATAAAAATCACTCTCTATCTCTCTTCGCACTTTAACAGCTTCTCTATATTTTATTAGAAACTTTTCATAGCCAGCTCCGTTGTCTATGTTTGACAATGAGAAACCAGAAATATCAGATGGACATGTTTGAAGACTTATAATATTGAAGAAGTTGTTAGTGTTTTTTAACAAAAATTCCTATTTTTATTAATATGGAAAGATACTTTTGGATTTGCTGTAGTTTCTCCTATCGATGCACGATTTGTTTATCACGATGGTGATTCATTATTGCTGAATCTTCTACTCAGCTATATGCTATTAATGTTCTACTTCTTCTAGTTCAGTTTCTAAGACATCTTCTAAATTACATTTTTGACTATTTATATATTTTATATTACATTATTTATAGTTATAAATTTATA

ATTATATATTAGATAATACATTTGATTA

>SbWRKY63

TCAAGAATCATCTTTTTTTATGGTTCCAATCATATATAAAAAAAGGGGAAAGCTTTTGGAGCAATGTACGTAGTTTTTGTTCTCCTGGAAAACTCTCTTTATGGATCCTCTTATGCATTATGTTGGGTATCAAGGAAAGGTGGATAAAACATTCAGAGAATGTAGACAACAATTCTTAGAACAACCAGAATTGATGAAAATTGTTATGGTCCTTGAGTTTGGTCAGTTGTCTTATACAGCATGCTATTTATGTTTAGAATGCATTATCATTACCATCACACCAAGATTTGTTTACATGTGATTTGTCAATCTCAACATACCAATGCAAGGATCTGTTGTCAATGTCTCTGTGGTGCCTGACTGCTTGGGGTATGAGGCTATGAACTGCATCTGTGCTCCTGTCTGCTTATGAACTTTAGCTCACTGCATATAAGAAATTTGATTTTCTTTTTCAGCTTGGATTTTCACTATCCTCGAGCAAGTAAAGTAGCTCTGCATACTTTATTAATAAATGTTTTTGTTATTAACCCCCAAATATAGTCAATATGCTCTAGCATACAAGTTTTAGTTGTCCACTGCGTCACCTAAATTTGTTGGGTTGGTCAGAGTTTGTCTCAAACAAGGATGGACAGGAGGAGGATGGGATCAGAGTCTGATACATCACTAAATGCTGACCATTGGAATATATCTACTCCCCTGCTTCTAGCCCATACCTAACAGTCTTACATGCATGTGCCCTAACTACCTCCCTCTTTCTCCTCTGCTCAACAACCTTGCTGCCTTTGCAATTGCATTGCTTCCACCAGTCTATACAGCTAGAGGACTCAGGTGATCACCTATGCGAACCCTCTTGTTCTTCCATTACTCTCTTTACTTGCTCAGTTCGTTGCCTATGTGAATGGCCTTCCTCGGATTACTATAATTTGGTTGACTTATTGTGGGAGCTTGAAATTTGCACTACGACATAGTTCAGTTTGTATATGAAGTATTTGTCTACTACTCTCTTAAGTTCCATGTGCATTTGTTTTCTTTTTCTTTCTCTCTCTGCCCCTACAAACAAGCAAATGAGCTACTGCTATGATTACAAAATCACAAATTGTCCCCTGTTTTCAGGTCAGGGAAGATGCAACCTCATGAGAAGGTTGCTGTTTTGAAGCCTGTGGCCTCCAGGCCTTTCTCCAGGTTCAGGCCCTTTCCGAATGTTCTGCAAGACTTCAATGCCAATGGTTCCCCAACAATCACCGTCCCAGAGGAGACTGAACTAATCAGGCCAAAAGCCACTCGATCTGCATCCCTACTGGGTAATCTTCCAACACAGATAGCAGCAACAATAGTAAGTCTAACAGTTCTCTACTTTGGAAAGACGCTTCATGTATTTTAATAATGGCAAGATTTTTGTGTGTGTTTGAAGTGGAGCCTTCTAAATTCAAGTATTTGTCTATCGTCTATTCGATTATTGAACTATCCAGGGTATCATCATGAAATCTTGTTTCAACTGTGCTAAACTTAACTTGCAGGATGCTGGGTCAGATGCCATATCTGAGGAGGTGGAAGCCAATGCAGAGCATTTGACGTGTTGTGACCATGTAACAGCATGCCAAGCTGCCAGGCGGAATGGTGTGCGCAGCCGTCTGTCACTCGATGGGTACAACTGGAGGAAATATGGGCAAAAGAAAGTGAAAGGCAGCGAGTTCCCACGG

AGCTACTACAAGTGCACTCACCCTAGCTGCCCTGTGAAGAGGAAGGTGGAGACGACAATAGATGGCCGGATCGCTGAAATTGTGTACAGTGGCGAACACAACCACCTGAAGCCAGGCAAGCCTTGTCTCCCCAGGAAGCCATTGTCGTCGACAAGCACAGAGGTTGTGGTGTGCGACATGCGTGGTACTGACGACACGATGAGGGAGTAGAAACGACGAAGACGATTAGAAGGTCAGATGCTTTGTTCCAGTATCTGGAGGCAGTTGCATTTTCTTGGACGAATTTGGAAACGATAGTTAGCTTGCTGGTTGATAGAAAAAGTACTGTCTTGTTCCAGGACATCAGTGGGTATCATAGAATGGCAAAAAGCAGAGTAGCTGTTGTCGATGTACATATATGAATCGATATGCATCTGATGCCACTAGGGTGCTGCTGAAACTGCCCTTTTCAATTCAGTTGCTGGTGTTTCTTCTCTGTATTTTTCTGCGGTGAATAATGGATCTAGTGGGAGGGAGGAGGGAGTGCTCATCAGTTGAGTTTTGACACAGCGATGCAAGAATGCGTACCGTGAACCATGTGGATAAATGGAATAGAATAGAATGTTCATTTGGTAGCTTACATGGTGTCCTCGCCGTCTAACTGTGCTGACTTTTTGGTAGAGTTCGTTGAGAAAAGTCGACAAATCAAACGCAAGCTAACACTCTTTGTTCGTGCAGGCGCGTTGTGGACAATTAAGGAATGAAGTGGCTTGTTTAACAAGAAGGTGCTTGCGTCCCCAATGGTGATCATTTACAAGACTCTAATGTTGATCAAGTCGTGGCTCCCTAAAGCGAAATTGGAACCCATGGCGGAAGAGATGATAAACCTGATCTCGTCAAGCGCACATTAAGTCGTATCTCTTGTAGTTCAGTTAGTCTTTTGCTCTTTGAGGGGAGTTATATCGTTTGTTGGTCAGTTGAACTATCGCTGAGAGAAAGTCGTTGTCTGTCAAATTTCTCTCGTTCTCGTTGTTGCTACTATGTAAACTAGTAATCCCAAGTAGCGTGACAAGGCAATCGCCGACCGAACAACGGAGCAGCAGTATGACCCATTTGCCACAGGAGAGCGGGGAGATGATGGCGGGGCCGAGAGGGTGATATAAACAACTCTGTTGAATTCGTATTAACCGTCCGCCACAAAGAAACTAATACTTCACGCACAACTTGTGTACAACCGCCCGCCACCAACAAGTGATCCTTCACTCACTTAGCTAACTTGTTCGTTTGGCTCATAAGCCATGACGAAAAGTATTGTTGGCTGATTTGTTATAAGAGAAAAACACTACTAAATGACTGGTGGATTTGGCTGATAAGCTCAAGCGAACAACATACTTTACTTTATTTTATTTTATATACAACTCCAGGTCACTCTTACAATTGCCATACCAACTGTTGGATTCATCATTCATACAGAATCAATGGCAATTGCATGCTCTCTGATCCTCTAGCCTACCCTCGTCTCAGAACCTGATAACTTGCTTTGGATGGCTTCCATGTTTGCCCAGGGCCTTCTCCAGAGCCAAGCTGAACTCACCG

>SbWRKY44

TGATCCAACACACAAACACACACACACACACACAGATATATACGGTAGCAGTAATCTCTTGCATGCACACCGTGTCCCAAGTCCAACTAGGAATGAATTGAGATATTAGCTAGCTCAATAATAAGGGCATGTCGTGTCAAATGAAAGGGCATCAACTGCTGCTTCAGATGACTACTTTCACTTGCACTGCACCACTCCATCCCCTGAGGGTATATTGGGAATGAAATTATCATGTTGAAAAAGTTGCATTAATTGTTATTGTTATATATATACACCCTCCCTTGGAGCGGGTTGGTGATGAGTGAAGTCGTCACTACTGCATGCATCTCTGTGCATACACATCATCATCATCATCAGAGAGTGAGCGAGAGAGAGAGAGCGAGCGAGAGAGAGAGACACCAATGGCTTCTTCTTGCATGCATGCTTTGCTTGTGTAGGCACTACTACCTAGCTCTATATATGTCTCTCTCTCTCGCTGCTCGCTCTCTTTGCTTGTGTAGCTAGCTAGCTAGCCACTATAGGCAGGCATCATCCTTTCCAGCTGGTCAGCCATCAGCCAAGCATGGAGGAGGACCTGCTTCTTCTGGAAGCATCAGTCGCTGTGCCGCCGCTTGCTGAGAGCATCAATAAGTACTGCTGGCCCAACGGCAGCGATTTCGCGACGACGGAGGAGCTGATGATGATGAGTGACCTCGTCGACGAGGCGGCCTTGTCGTCGTCGCCGGTGCAGCAGCAGGAGGAGGAGGAGCCTCGTCGCCAGCGGGAGTCGATGCTCAACAAGCTCATCTCCACAGTCTACTCCGGACCCACCATCAGCGACATAGAGAGCGCGCTCTCTTTCACCGGCGCCGACCAGGCCGCCGCCGTGGATGCTCACATCTACAACTCTGCCGGCCCAGTGTACGTCCGTCGTCGTATGAATTCGTGTATATGTATATAGTAATTGTAAATATTAAATACTGTAGAACATAAAGTGGTACCGTCTTCTACTATAGGTAAATTGATCGATCACCAAAATGCAATTAGATATATATATATAGATGCATGTTGTTCTTCATTCATATACATCGATCATTCATCATCACCTGATCGTTACAGATGCTCTCCATAACCGATTGATCCTATATATCAATTAATTAATTATGGCATGTAGCGTTTTCTCCCCGGAGAAGGTGCTGAGCAAGATGGAGAACAAGTACACGCTCAAGATCAAGACCTGTGGGAACGGCCTTGCAGAGGATGGATACAAATGGAGGAAATACGGCCAGAAATCCATCAAGAACAGCCCCAACCCAAGGTATAGAGAGATACCCTTTTTAATATATATATATATATATATATATATATATATATATATATATATATATATATATATATATATATATATATATATATATCTGTATGCATGTAATAATAGAAATTAGTTTGGCGATCGATCGGCCGGAGATTTAAAAGAAAAAGTACTAGAAGAGGTAGCTAGCTAAGGTCGTCGCTACATATGATACTACTATATATATGTATGTATGTATGTATATATGATAGATAAGTGTGAGGAGGGGCGACGGTTGATGCATGAATAATACAGTATATATGAGTAGTCTGGCCAGTGATGATGAACCAAGGAACGAAGCAAGTGAGCCAAAAAGGGCAGTGCTTGCTAGCTGTGCACTGTCCTACGTACCTAGCTAGCTACTAGCTA

CCTTTCCCAACTGAAAATTCCCAAGACAACAACATGTACACCCTCCAGTCCAAACGCACAGTTGCTAGCTAGCTTGCCAATGCAATGAATCGAACCATGCAACACCTAGCTGTGCTAGCTTGCAGTATATATTTGTGTGTCTGTCTACTTATATCGCATGAGCATGTGATCAATGTTATTAGCTGATTAGAATTGAATTCAGACTCGCATGCATGCAAATTAAGGTAATAAGGTAGGTGTAGAATTGATTACATGACATGAACTGATGAGCTGATCGATCGTCGAACGCTTTATTGTTGTGTGATGCAAATGTGCAATGCGATTGCGTGCAGGAGCTACTACCGGTGCACGAACCCTCGGTGCAACGCCAAGAAGCAGGTGGAGCGGTCGACGGAGGAGGCGGACACGCTGGTCGTCACCTACGAGGGCCTCCACCTGCACTACACCTACTCGCACTTCCTCCAGCCCCAGCCCCAGCCCCAGCCCCAGCAGCCCAAAAAGCCCAAGCTTGGAGGCCCACCACCACAACCACAGCCCATCATCATGCTGGAGGACCTCGACGGCCCGGCCCAACAAGATATCACCACCTGCCCACTGGACGCTACCGCCATGGCTCCAGCACCACCACCAGCTGCTTTGTGCTATCTTGACGATATGTTCCAAAAGCCTGCCTTTTTCGAGGAGGAGCTGCAGCAGCAGCACATCACCAATGGCGGGTTGCTGGAAGACATGGTGCCGCTGCTGGTCCGGCGACCCTGCAGCAGCACAGGGGCCACCACCACCACCACTGGCAGCAGCACCACCTCGTCGTCGCCGCAGCTGGCACCGTCACCGGACCTTTCCACATCATCTTCTGTCTCATGGAACCCCACCTCTCCTTACATCGACATGGCCATTCTCTCCAACATTTTCTAGCTAGTCTCTATCTATCTCTATATATACAATAGATAATTTATATATTTAATTTGTGCTGTGTTTGTTGTGTAGTGATATATACTACTAGTATGTATGTAGATGCAGTATATTCTAGCAGCTGCACTAGTGTGTATGTCCTCAGGGGCTATTGTACGTCTGTTAATAAATTGTTTGTGCTATCTAGTAGTATATGAGAATGGGATCTCATTATATATATGTTATTATATGTATCTAAATAAATATTATACCGCATCATGA

>SbWRKY1

TAGCAGCTAATTAGCTTAGCTTCATCATGGAGTAGTATATGCAGCTCATGTGAAAGAAAGCAGGCCGGAGAGAGAAGTTATTATGGGCAGCTGACAGTATATATCATCCAGTGTGTGTGCGTGTGTATGACTGTGTAAGTGTGTATGTCCATTGACTGCTAGCTAGCTGCAACGAACGCCAACCACACAGGGGTGAGTTCAGGGCTGTTGGCTAGCTAGCTAGGTAGTAATAGAGCGAGCTGCTAGTGGAAAGTAGAAAGAGTGAGCACACCACTATGGACATAGCGGAGGAGGCCGTCGCCGGCACCGCAGCTCAGGGCGACCTCGCCGAGGTTGTGGCCCGTGCAGGTGCAATGGCCATGGCCGCCGCGCCGAGTCACCACCGGCGGCCACCCTCTCCCTCTCCTGCAGCAGCAGATCACAGTCACGTCATGTCGAGGGCTGCCGGCCAGATCATGGCCATCCCTCCTGCTTGTTACGACGAGGAGCAGGAGCTACGGCCTGCTGCTGCTTGCGGTGACGCGGTGATGTTCGACGTGCCGTCGTCTATGGTCGTCGATCCATACCATCAGCTGTCTTCGTCGGCGGCCACGGCGCCGCCGCACGGGCACGGCGGGTACTGGCTGCCGCCACAACAGATCTCCCAGCAGGCCTGCTATGGCCTCGACGTGGCCATGGGAGGCGCTGCCGCCGCCGATGCCGACGGCGACGAGCCCATGATGATGAGGATCTCTCCAGTCACTCCGCCGCCGCCTTCTCATCATCAAATCATGAAGAGGTTAATTGACTGGAAAGCTAAGCTAGTGCTCTCTATATTTCGTGGCTTGATTTCTTTAATTTTTGGATCTTCTTTAACTGTTGTCTCCTTCTTCTTCTTTTCTCGATCGATGCATCCTTTTCAACTAACTCCTAAGATCTCGCATGATTGATTTTCCATGCCCCTCTCTAACAATCTATCTATCTATCTATCCCTTGGCTTTTTTACTGTTATTTCGTGATAATTCATGGCTGTTTTGCTCTCTGATTTGCGCTACTCATTGCTGCAGTTTATCCACTGCACGATGACCCTTTCTCAGCTGCCGTTTACTTCTGTGGATGCGTATTTAGTTACCTGTAGGCTATCAGTCAAAACATGCTTTGCATTGGGATGATGTGACTCAAAGCTGATTGCCTGATTTCAATTCGACTGTTCAGTTTTGACAAAACGGCGCACAGTTGTATTAACCCCTTTGTCTCTGCATAGGCATATATATATAGACACAATATTTCTTTGGGAGGGATTTGGTCATTCAGAGTACAAATAAGCTAGTTGACTTTAAAATTCATTCTTCGTCATTTGATGCTGGTTGGAATATTTTTTTCATCTGCAAAAATTCTATTCCTTCCTCATTATTGCTTCTTCCGGCCTAGTATTTCTTTTTTTTTTTTCTTTTGAGAGAGGTGCTCCGGCGAGCTAGCAGGCACTTAGATATCCTCTCCGTGTGGTTTGTCAGGTTATTTCGTGTTGATCTCTAATAAACTAAGTTTGCCAAATTAATCAGATCGAGCGTTTATTTCCTTTTGTCTCTTAATTAGCTTGTGCATCATATATAGTTCCAAATTATACTCTTGGAACTTCATACATGACTCTAAAATTAAATAAATGCAGAACATTTTGCGTATTTGCAGCAGAAAAAATGAGGTGAAGAAGGTGGTGTGTA

TCCCGGCGCTACCTCCGGCGAGCAGCCGGCCAGGAGGAGGAGAGGTGATTCCCTCTGATCTTTGGGCGTGGAGGAAATACGGCCAGAAGCCCATCAAGGGCTCGCCTTACCCAAGGTGCCTGCCCACCTTAGTATTTTTTTTCTTTTAATTTGTTCTTGCCAATGCAAATAACAAACAAATTAACACTTAATAAGGATTCAATACAATATATAATTAATACTTATCAACATCAGATCTCTTCCTTTGCCTTCTTTTTCTGAAAAGGATTGCAGTCTCTTTTTGTTGCACAGGTACACACGGTGTAGAGAGATGTAACATAAAAAAAATTAATGTTTCCATTTCATTAATAATGTGCACTGCTCTTATGGGCAGCTATACCATAGCAAGTCAACAGAAACCATGCATGGTCATCTTCGATACAGACCGCTCCAATATTCCCTGATAAGAACTGAATTAATTGGTCCAGCAAATAGGCATTTAGATATTGTTTCAGAGAAAAAACATATTAATTCATTCATATATAGCTCCCTCTTGATCTAATTATAAGTACTTACTTTCTACTTTACCTTCTCAACTATAATATACTACAAGAAATAGTTAGATGTATATATAAAGAATCTATCGTGCATGTTTATATACTTCTTACACGAACAAACATCCTCGATTAGGTTGATCACCCCTTCACTTAAGGTCGCCAATCGCCACAAAGAGACTATACGTAGGCAATTAACCCAGGCACACTTCGTCTTAAAGTGGGCTTCATATAGGGCAAATTGGCAGATGAGACTAACAATAGTAAGATGGCAATCTCTGTTGATTTCATCATCATTCTTTTTATCAGTTACATCCATCTATTTTCTCTATTTATTATGCATCAAATTAAGATAAGTTTATGTATTTAATATGCAACTAATTAAATTCTGGTGTTTCTAAAAGATTCAGTCCAAGTTTATGGGCCAATGTCCAATTGCTAATTGGAAATGTATGTGGCAGAAAAGATATATCTTTGCACTTTTAATAGGCCAATTATATGTGTGCCTCTAGTTTGTTGTGTATATATATGTCTCATTTGGATGCCATAGTTTGCTTCCACCAATTTGTCTAATTGTGCAAAGTATGTAAAACTAAGGACAGCCATATTAGCTGTGGACTTATTCTGTTTAGCTGAAACCAAGAATATATAACCAGAACCAGAAACTCTCTTGGCTGCCTAGGTTTCCTATACTCTTGTATATATATGGTCAAATCCATACTGTTTCCAGTCAGAAGTGGTCGTGATTCATACATTCAGACTTGCTTCTTCTAGTTTAGTTTAACCCCATGTACGTGTATATTTTAATATGCGAGTCAACAGAAATTTCAAATGCAATTATTATAGTGAAAGTAGATCTTCCTAACAAATTAGTTTGTCCATATATATTCCATAAACTTACTGTAATTGTTTTAGAAGTCAAATATGTTTTTACATTTTTAATGTTCCTTCACTAATACTACTCTACATTTTTTCAAAGGTATTGACAGATATCCAAATAAATAACGAAAATACTCCATTACAATAACTTTAATTAATGGTGACATAAGTATTCTAGCAACAAATTACTTAGGCTAGTAAAGCTTCCATATATCAATCTACATGCCTGAGCTGTAATATTTGTATGTTTTCATGATTTTACCTCATATCCTATGCCACCTAGAATTT

CATCAAATCCTTCATATATTAATTATAGATCCTACATACTTGATACAGAAGCACAAATTATATTAATTATGTTCTAGTATATAGAGGCGGTAAGATTATCGATATGTGTATATATATTGTTTCAAATCTAAAATAATAATAGCCAATCCCTTATCCCAATACTCATTAATCCACTGCTGGGAAGGTTTTATCTAAAGTATATTATTTCCCAATGCATGTATTTGATTAAGTGAAACTTTATTTTATTTTACAGAGGTTACTACAGATGCAGCAGCTCAAAGGGATGTATGGCGAGGAAGCAAGTAGAGCGCAGCCGCAGTGACCCGAATATGCTAGTGATTACCTACACGGCAGAACACAACCACCCTTGGCCAATGCAACGAAATGTACTTGCTGGGTACTCCCGGCCTCACACTCACATGTCCAACTGCAAGAAGAAGAACAGCTGCAGGGTTGAACCGACGAGTTCATGGCCAACATCATCATCGTCGTCATCATCATCAAAGAATGCCAACTACTTCGAGCATAATGTAGTTCCCAGTAGCAACATCGAGTGTCAACAAATGACAAACATGATGGAAGATAATGCAGCTGGGTATGTTGCCTATGCCATCGATACTCTTGATGAGGAGGGTGTTGCGATGCATCAGCCGATCAATCGCAATAGCATCCAACCTTCAGATGAAGTTTTCGCAGAGTTGGAGGAATTGGAGCCTAGTAATAATCCTGTGAATGCAAACATCTACTCTAGGGGGGTAAGTTATGAGTGGCAAAAGTTCTAATCTATCAGGCTCAAGGTATAAGGAAGGCTACAATACATATGGCATAAATGCTTTGATGAAAAATAGGTTACAATTATGCACACATTTTACTTAATTAATTGCTGTGACAGATCATAATATATACCTTTGTGCTTATGATTAATTCAAAGCTAAGATATATATTTAAAGAATGAGAAAATAAATATACGAGTTTAAGATCTTGTTGTCTACATAATATAAAAAATTCCACAATTCTGCATGGACTGTATTTATTACTAGCACCCAATGTTATTCTAAATGGTCGGTCATCCTTTGTTTAGTGCTTAGGCCATTTAGACAGTGTTTAAACAGAGTTTAACAATCCAAATGGTTTTATATAGTAACACTAAAATATACATAGTAATTATGTATGTAAAAGTCAACAAATGTGCTAGAATGTATAAATATTTACGTATGTCAAAACAGTATTCTGTCAAATATTTTTTTAGAAGAAAACTAAATCTACTTCACCTCATATACTTATGATAATAATTAGATGAGTGCCATTGTGATAGTTGCAATCCTTCTATTGATTAAATGATGAATTAAATTGTTGTTTATCTCGTTTAATCACATTTAACTTGATCTTGTTTAGACCGCTTAGAAGGGATTTAATGCTCTAAATAGTTCAACCGTTTAATATATGCACCGTTTAGGACCTAAACAAATGAGTGGCCTTTTTTGCCATTTAAACATTTAAATGGATTAAACCAATGTTTAGGCAAAAGATTGCATCATGATCTTTTTATCAAAACTAGTTCTTTTTTGACATTTCCATGTTGACCAACAAATAGCTGCAGTCAACAAAACATGCATCCATTTATCATTAGGTAGCCATTTGTGCAACCATGAACTTCATTGTTGAATGGAATTAGGGAACAGTTTGCACCAATCAAGCTG

ACTATGAAGCCCCTAGTTAGATTTGACAACTTTGCATTCAAAGAAAAGATGACTAGCTATTTCATTCTCATTGCAAAAATAGCATGTGGGATGGACCTTCCATTTCCTTTTCATCATGTTATCTTTGGTGAATATGACATCATTCTCGAATAGCCACAAGAAGTATTTAGTTTTTGGTCAAATTTTGGCTTTCTAAATGTGTTTATGAGAAGCACCAAATTCCATTTGAGTTAATTATTCGTACATAGACCTCACTATGAACAAGCTAGATCTTGTCCATTTCCACATTATATTGTCTCTAGAATCCACCAAATTCAAAGTTGAGACATGACTCATAATAAAGTTTCATTGATCCCTCAAGAAACCAAGAAGTTGCCTTCCAAATTTCAAATTTTCATTTCTAATTTTTATAGTTATATATTGAAATGTATTTCTCTTCACAAAGATCAAATGACAAGATAATGTTGGCTCAAGGGTGTCTCACCCCACCAGATATTCATCCAGAGAGAAGTCTCTTCAAAAATAAGTTTTACCCCTAATCATTTTATAAACAAAGATCAGAGGCTTATACGGTGCTGACTTGGTCTTGATGGTCAATGGACATGGAACCAATGAGAGATCTATTAGGGTTGTGGTAGTGGGATTGTAGTATGGATAGGGTGGCTAATGGGGGACTTATTGTATTAGCAGGGTTTGTGCTAAAGATTGGGGGTGGTGATCATCATGTGGTTAATTAGTGATAGCTAGGGTGGTGCATATCTAGGAAAATAAGGTTATAATGGACGAAACGAACTATGAGAAAATGAATTAGGGAGAAGTAATTGGGTGCTCAGTTTTGTGGGTTTTCCCTTCACATATGTGCATGCCTGATAAAATCCTAGGGTATGCCATCGCCGACTCAAACCATCCAATGGATCAGCTCTACGCTTGCACAACTACTAATTAACGAAGGCAGACAGACCTAGTGATCACCTTCATAAATGCCTAGGATACCTTAGAAAAATTATGCCTCTAAAAATAATTTATGAAGCGGGTGTCCTAAGACGACTGCCTCTGGAAATCATCGATTAATGGAGGCGCGCGCCTTAGGACGCCCATCTCCATAAATCGATTTTTAAAGACTGGCATCTTAAGATGCCCATCTCTGAAAATCGCTATAATAGAAACAATAGTTCTATGTGTGCGTCTTTAAGAAATAATTTTCAAAGACGGGGTCTGATGATGACGATAATTGGTTCAGGATGGACGAGCTAAGGCCGGAGACAAAGGGGGTGAAGGAACATCCACACTAGCAATAATTGGGGCTTGGGGTTTGGGGAGGTGGGAGCATCCATGCATGACTACTTATTAGGTGGAATGTAGGAGAGTAGAATCAGACAAATACTAAGAAAAAATGCACCTATTGATTTCATGAGCCTTTGGCTACATAGTATGCATGGTAGTACATATGGTGGTGACTCAATTATATTGTGGCCTTTGTATTACTGTTTGTTGGGAGGAAAAGCTTGATAATCAACCATATGCATGGTACGTTCCAAGGAAAGGAAACATACCCTATATATATATATATATATATAGACCCGCGTGTAGTTACTCTCATGTCTATATTTCTAGATTTAGACTGTATATAACCGGACGAAATGTATATAGACGAAGTATATGATCATACTAGACCGTCTAGGGTGATATATAAGTTAAGT

ATGCATACATACATAGACTATGTGGTTATACTTATTGTATGTAATATAATAGTGGCATTTTAGTAATATATTAAAAACATGAATTTCTTTTAAAAAAAATTTCACGTGGTATGATCTAAAGTATCTTAATATGAGTATATAAATATACTCAAATCAATAAACAAGTATGAACACATACATAATGTAGTTTATTGAAGATGAGAGTAACTACACGTACGTGATGTATCAGGTGATAACATGATGAAAAAAATTAGGCTGCAGGGGATAGACCGTGAGCTATTTTTGCTTAAGGTAGAGGACTTCTCACCTCAGCCCGAGAACCCCGAACCCCATGCCGCGCCCGCACATAGGTGCCCGTTACCCGATGAGAGAGTCGGTCTATTTACCTATACTTTGGAAACACGGGACAGATGAGGGTTTTTTTTTCACACTGCTGACCGTAAATTTACCCGACCAAGGTTTGAAATTTGGTCACGGGGAATGCCATCCGGAGGCTTGACCAGCTAGTCTAGCAACCGTAGGTGTGATAACATGATGAGATTTGGTCCAATAATGAAATTGAAATGTTAAGTCAAGGCGTACCATGTTATTAATAAATTTAGTGTGGAAGATAGGGTCGGGGTTCTAGGAGCTGAAGAATGAGGTTTTTTATCGGCACCAATCATGTATTACAAGGTTCTATGGATACAAGCAGAAACAACAAAGTAACAAAAAAACACAGACAGCCTTTTGGCTTGTACTATCTAGCAAGAGCTGCAGCGCCCAATCCTAAATTAGCCATGGAGCCACACTACTCAAAAGCTTTTGTCTGCTGCGTCAAGAGAGGAGTAAAAATATAATCAGGGCAAGCAGAACGAAGATCTATATATGTGTTGTATTGCCTGTGTTGTGTGATGATCTACTGATCTCTGAATCTAGCGGCCAACCGACATGAAGATCTCTGGTAGACCCAGCTCGCGATTGAGCGCCAGTGCAGAGGAACTGGAGTACATGCATACCATTGATATGATGAGGAGTAAAAGGTTACTAACATGCATGCAGTAATAGGACACATCATGTTGTTCCTCAGTCGATCATGGACGGCGACGATATGATGTGACAAGTCAAGGAGAAGAGAAGAGAGCGAGGCAGAAGAAATAGAAATGGAGCGCACACAGACCAGCACAGTGTGCTTTTTCTCCGGTATCCATCGATCCAAATAGTAGACCTACTTCATAACTGTTCACTCGTCAACTCATTTTTGACTTGTTATTTAGCAGATTAATTAACTGGCACAATGGATTGTAAAGTTAATCCGTGCAAATAGCTCTAATCTTATTTTTTTTTTCATCGTAACCATTAAGGTTTCTTTTACTCAAAGCAATTAACTACATCCACTTACGCGAACACTATGGCCTTGTTTAGTTCCAAAATATTTTGCAAAATGTGAATAGTACCAATTTCGTTTGTATTTGACAAATATTGTCTAATCATGGACTAACTAGGCTTAAAAGATTCGTCTCG

>SbWRKY8

TAGCTTGGATCGATGACGATGGTACGTGCTGAACCTGTCTGTGGCACTACAGTGCCGTTGATTTGGACCGTGCAGTCGAGTGCACGCATGCATTTTGCTCTATGCTCCTAGCTAGGGCTGCAGATCATCACTGGCTAGCTCAATCGTACTACAGTGCTCGATAACGCTGGCTCCATTTATATATCTGTGCTGTGTTGCCTCAGCCTGACACGGACACTTTATTTCCATGCAAGTTAGATCGATTCTTGTGAGTAACTCTTTCGCCTCCTTGAATTTCTGTCGGCAACACACAGATCCTGAATTCAGATTTTGATCGACCCACAATATATAATAGATTGTAACATCAGTAGTATCATGTGCGACTTCTTTTGGCTGTCGCCGGCCGATCAAGCAGGCGACCTCTCGGACGTTGTCCGGGCGAGCCTGCAGCCACCGCCGCCGCCGCCTCACCACCACCGACTGCCGCCGCCGGAGGAGGAAGAGGGGTTGCTGCTGCTGCAAGCCCGAGTGAACTTCGATGGCGACGATCACGATGAAGCGCGCTCGCAGCAGCTGGTGCATGGCAATGGCAGCATGAGGCTCATGCTGGGCAGCAACGGCAGTGGCGGCTGCGCTTGTGATCATGCTGCTGCCCTGTGTCCCCAGCACCATCCAGAGGCAGAGCGACTGATTCCTCAGCCGCCCATGTCCGGGCCGCAGCCACAGTTGTGCGCTGCTTCCAGCTTCGTCGTCGAGAGGGACGATGATGATGCTCCCGTTCTGGAGGAGCATGTCCTCGACATGGCGACGGCCCCTCACCCTCATCCTCATACCTCGGCAATCAAGCGAAGGTTCGTTCTTCGTTCTTAGCTAGTTATATATATCATACAGTATATATTTTGTTTACTTTGCATAAACATAATTGCGTTGCATTGCATTACATTACGTATTGATTTGATGCATGCGTTGTGTGCGTGCACGGTACGGTGTCGTCTTATAGGAAGAGCCAGACGAAGAAGGTGGTGTGCATCCCTGCGCCGGTGGCAGCGCCGCCACCGGGGGTGGGCGGGCGGCCGAGCACGAGCGGCGAGGTGGTGCCGTCGGACCTGTGGGCGTGGAGGAAGTACGGGCAGAAGCCGATCAAGGGGTCGCCGTACCCGAGGGGGTACTACCGCTGCAGCAGCTCCAAAGGCTGCTCCGCCCGCAAGCAGGTGGAGCGCAGCCGCACCGACCCTTCCATGCTCGTCATCACCTACACCTCCGACCACAACCACCCATGGCCGACGCAGCGCAACGCGCTCGCTGGCTCGACCAGGCCGGCCTTCTCCTCCTCGTCGGCGGCCAGGAGTCATCACCATCACCACCATCACCATCACTCCGCGGCTGCCGTTCCGGATACGACACTGGCCCACCGTCACGCCAGCAACGTCGCCGTCGCCGACAATGCGACGCCAGGGTGGTCCATCATTAGCGCCAGTGTTCATCACCAGCTGCTCAAGCAGGAGGTGGTCGACGTGGATAACCACCCCATCAGCAAGCAGCCGGCGCAGGACGCTGCAGCTGACCGTGACTGCTTGGACATGTTTGCTGATATGGACGGCGCCCTCGACGTCCTGTGCGCCTCCAACTTCCACCCAAAAAAGCAGCAACAGCAGGTCACTGCTGCTCAGCACTTGGAGAAGCTGCCGGAGGAAGAAGACGAGCACTTGCTGCTGGGTCCG

GATCCTTTCAGCTTCAGCTTCTTGGACTGGGTTGGCGCTTCATTTGGAGTTGGAGAGACAGCAGCAGATAACGGTGATCACAGTTAGCCGGCCTAATAATCCATCATCACTACTACTATTAATCTAGCTATAACTGATCTACATCCAGCCATCCAGCTAGTCTCTCTTTAATTTCGTAATCAAATTAAGCGCGCCAACAGATCTATGTTGATATTATATTGTTATAAAGTTTTTAACTAGCTAGTCTCTTTTGAGTATATTCAAGTAGATAGTATTTCACTCTATGAGCAATCTTTATTTTCTGTTGAGATTGTGCCTTTTCATTTTATTTTAACTGAAAGGATCAATGATATGTTACCAAGAGGAAAGAAGGCCCTGTTGTCAGGTGTTCTACTAACTTCGTGGGCGATTATTGTTAAATATACATGCA

>SbWRKY48

CGATCCGCCACCCCCTCACAAAAAAATTTTTACCGCGGGCGCGCGCGCGCGGGGCAACGCCATTACTATAAGAGTCATTACTATAAGAGTCAATAATGTGCTTAGCTATAAGACAAATGATTAGAGCCAAGTTATACAATAAGTTATCTTATATTTGTCTCTAAAATATCTTCTATTTCCTATTTTTTCTCACAACACTTGCTATTTTGGCCTACCACTACCTATGCATTCGTACCCAGCTTGGTGCTTAGTGTTTACATAAGAGCCAAGCTATCATCTCTCCCCTCCTCTTCTCTCATCTACATCAGCATTAGCTTGGCTATAAGCTCATTATTGTACCTTAACAGTGTCCCGTCTGATTTACCGCGGCTCTGATCTGATCCGTGCTGTGTTCCGCGCATCGAGAACACTAGCAGTCCGGGAAATGAAGTCAGCGACCACGGAGGAAAAAAGAGCACTAGCACTCCCAGTTCCCTCTCTCCTCCCTCGCTTCGCTGCTGCCACTAGTATTTTGTCCGCTCCCAGCCAGCCCTTACTGTGCCTGCCATGCCTGCCCCTCCCCTCCCTCACAGTAGTCCCTACTCGCCTCCTCGCCAGGCCCCCGGCCAGCCCTTCCCTTCCCTTCCCTTCTCTCTCTCCTCTCTTCTGTCCTTTTTCTCTCGTGTCCTAGTAAAGAAAAGACAGTTTGATTGCACAGCTAGCCCATGCTCTTATCATCCGCTTCCCCCTCGCCGGCTCTCCCTCCTCTCTCTCACACACACACAAACAAACAAGCCTGGATCGAGCGAATTCAGCCGAGCGAGCTTCAGAAACCCTTGCGGGCAAAAGCTACTAGCAAGCTCTCTCTCAACCCTCCACTTTCTTCATCTATATAACCCTAAATTTGTTTGCTTTCCTTCGGTTCTTGCGAATTGCCAAGAACAAAAGAGCGAGCTCTCTCCTGTTACAAAGACTAGCTTGGTGAGACCGAGAGGAGTACCAGGTAGACTATCAGCTAGACTGCTAGAGCGAGCGAGCTAGCTAGCTAGCCATGTGTGACTACTTCCTGCAAAGGATGGAGGGCGACCAGCATCACCAGGCCGGGGACCTCACGGACGTCGTCCGAGCCGGCGGCGCGATGCATCAGCAGGGAGCTATCGCGGAGCTCTCCTCCTCCACGGCCACGGGGTGGCAGCTCCCGGCCGAGCCAGCTCCAGCTGGGCCCGGCCTCTTCCTGCCACCGCAGCCGTCGTCGTCGGATGGCGGCGACGGCTTCGCGGACGCCTTCGCCGGCCTCCCGGACCCGTTCGCCAGCGACTTCGTCCGCGCCTCTTCCTCCTCCGGCGGCGGCCCCGTCCCTGCTGCCGACTTCTTCGACTTCGAGGCGCCTGCCGCTGCTGTCGGTGGTGGCGCCAGGAGAGGCGGAGGCGGCGTACTGGTGGATAGCGGCGGAGGCGGAGTGGTGGTGGAGAGGGGGGTGCCGCAGATGCCCGCGCTGTCGCCGAGGGAGATACGGCCGTACCCGGTGACGATGATTGGCGGCGACACCGTGAAGATCGGCGTGCCGACGATGATGCCCGGGCTGGCGGTTGGGCCGGCCTGTGCGTTCGACGCCATCGCCGGGTTGCAGATGCCGTCGCCGCACGGCGGCGGGATCAAGCGCAGGTTCGTTCGGCACCACACGCGATCGCTGCCAGATCTAAGCAGCTTTTGATCATTGT

CATTCATGAAGCAAGCCCTTCATTGATTTCTTCTCCATGGAGTGTTTGAATTTTGCATGGATTTTATTTCTTGCGCTAGAGTCACAGCTCCAGATTTGTAATTCAGGGAAAATGGTGCTAATCGCTTTTCCTGCTCCCCACCTGCTCTTTTCGTCATTCTGCATCTTCCAAGAAAGCTAAAGAGAGTAAAACATCGACAACCAATTTTTTGTTTCTAACAAGTTAAAAATAGCAAGATCAAATGTTCTAGAATCTAGATACACAAAAAATGGTTAGCTGTTGGTGAAATCTTCTTGAGTAAACAAAAACAACTAGCATCTCTACTCAGTTTAAAAAAACAAATACACTCTATAGCTACTAGAATTTCAGCTAAATTAATTAATTAATTATTAGTATGGCGCCAGAGCAACTAGGCCTATTTTCTTGAGCTCAACTTGCTGCTTCCTTGCAAACCCTAGTTTTATCATTTTTCGCAAGATGCATTTTTTTGAGATGATCATTCACGCATATAACAATATGCGATTGCTCAAAAACTAAATTTATATTAATAATCTCTGATATGATCTATATATGGCACTAGTTGCTTATTGGTGCTTAATTTATTAATTAATTTGTGTTTGATATGCAGGAAGAACCAGGCAAGGAAGGTGGTCTGCATCCCAGCACCTGCAGCCGCGGGAGGAAGGACCACTGGGGAGGTTGTTCCTTCTGATCTCTGGGCTTGGAGGAAGTATGGACAGAAGCCTATCAAAGGATCACCCTACCCAAGGTATTGCGAAGACTGATCGACCTCTCTTCTAGTTCATGTATGCATTTGAATATAGTCATACTAAATGTTCATAAATTAATCATTATATATGGTGGTCATATATGTAATCATAATAATATAAGAACATTAGTACTCCCGACCCATTGATCTGATATACCATCTCCTCAATCAAGAGGAGACACACACGGATTCCCTATTTTTTTCCTAGTACTATTGTACTACTTGCTGGTTAGTTGTAGAAGCTAGCACATGATAAGGTAGCAGCTAGCAGCTGATGCTATATATACAAATTGCAATTGGCAAGAAGTGCAACATTTAATTGAACTCTATCATGCATGGAATTTCTAAATAACGACTGTGAAGTTCAAGAACTCAGCTGTATTTATTGCCTTGTCCTCCTTTAATTTGAACCATATCATAGAATTTTCAATGTCCACAGCTATACTACACACTTAAAAAAATAATATTGAGTTGATTCAATAGGGATATTTCTTATATCATGTCGCCACACAAAAACATCACTAGATGTTAGAAACAGATAAATCGGTGCATACGAAAAATGTGTGAAGTCGCATAAATAAGCCAAGAAAAGTTAAATGAATCGAGCTCCTAAATTTGCTTTGTGCACAATATATTAATCGATAATTGTTTTGCTTTTTTATTCGGTCCATATATAAGGTGGCCTCCCAAGGGCGGCCTACTTGTATTTACCTTCTATATGTCAATAGAAATGGTGCACAATCATGCCCCGTTCATTCAAAAAGAAGAACAATTGACAATTGTATCACATTTTGTCTGCAAACTTTGCCTTGCTGTGTAAAAGTCAGAACTTAAAACCATGTTGATTTTGCACAAAACCCTTCTTATTCAGTGATAATACTTTTCATTATCAGGCATACCTCACCAACAAATCAATTCAAATTTTCTATAA

AAGATTGTTAGCCGACCTATTATATTTTTGGTTGATCTATAGTGTTTGGAGCATTATATATGGTTCCTTCACTTCAAGGTGATTCTTAAGTACTTTAATTACTTTTGTGATTTTTTTCATACATAGATAGTCTCAGATATGCAAATAAATATCACCAACATCCTGTTAAAATATATTTGTTATATATAGTCAGGAGTATGGTCTACCTAAGCAAAATACGTCATTAGCTCTGCAAAAAACGTTTTGAGGTGGTTCTAGCTGTTTATATACCTTCTAAAAATTTATGCTGCCGACATAAGTTAAAAATGTATTTTGTCAAGAAAATCTTGGCAAACTATTTGTCCATATATATGGTACAAAGCTCTAATTTCTTGTGACATATATAAGAAGCAGCAGTTATGCTGTGCTTACAATTAACTTCATGCAAATTAATATATATGCACTTACTGGTTAATTAAGTTCTGAAAAAAAAATTGTACATTGTTCAGAGGGTACTACAGATGCAGCAGCTCTAAAGGATGCCCGGCGCGGAAGCAGGTGGAGCGCAGCCGGACCGACCCAAGTCTGCTGGTCATCACCTACAACTCGGAGCACAACCACCCTTGGCCGACGCAGCGGAACGCGCTCGCCGGCTCAACACGCTCTCACCACGCCAAGAACAGCAAGAACAACCCCTCGCAGCACAACCTGCAGAAGCCAGACCTTAAAGCCGAACCTGAGCATCATCAGGCCTCGGCGGCAGTAGTCCCTACTGGCTGCGCCACCACCGCGACGACCGCAGCCACCAGCACCACCACCACAGCGACCACGAGCACCACCAGCAACAGCACTCCTCCGCCGGCGACGATGGCAGTGAAAGAGGAGGCAATGGTGGGGTCGGAAATGGAGAAAGGGATGGACCATGACGCTTCTGTCTTGCTGGATCACGGTGATCTCATGCAGCAGATGTTCAGCCAGAGCTACTACAGGCCGATGATACCGGAGGCCGGCGGCGGCGGTGGCGGCCACCACGCCGATGACTTCTTCGCTGATCTCGCCGAGCTGGAGTCGGATCCCATGAGCCTCATCTTCCCAGGTGGTGGTGATCCTGGAAAGGAGAAGGAAATGATGCCCAACAAGAGCTTGGGCGCCGATCCATTATTCGGCATGCTAGATTGGGGTGCCACTAATAATGGTGTTGCTACTTCTGCAGGGAGTTCATTTGAGCAAGACGAGAGTGGTTGGTGACATTGTTTAGCTTGCATTGCATCTATATATGGTCAAACGCAAGCATGCATATATGCACGCAACACATTCGATCTCTAGTAGGACAATAACCATTGCATTAATTAAGATGGAAAGAGGTAGAAAAGGATAAGATGAGGGCTAGCTACTTTTGTTTCTGTGTCCCCCTCTTTTATATATAGTAGTTTCTTCTTTCGTTTTATTTATTTATTTATTTATTCTGAGTGATGACTTAGAATAAATACGAGCAAAAATCGCACTAGAAATCGTTTTTTCTCATTCTTCTTTGTTTCTGTGGGGGCTATTTTTGTTTTCTATATATGTCCTGTTCTTTTTTAGTAAGTAATTTCTAATTCGTTTTATTTATCACTCTAATTGATGTGTTAGATCATGCGTTTTTAATCGT

>SbWRKY61

AAAAACCACACAACCCATCACTCTCGCCCTCCTGTCCCTCTCTTGATATGCCTGTCTCTCTCTCTCTCTCTCTCTCTCTCTCGTCGTCGTCTCCCCTCACATACTTCTTTATTGTCCTCTCTTGGAAGCCAACTCAAGTTACCCATCTCTCTCCCCTCTCCTCTTCCTCTCCTTGTGTGGTGTATTCTCTTGCTCCTCTCCTGTTGCTTGCTTGGTGTAAAGAAAAGATAGCTCACTTTCCACAGCCCCGGGCGGCCCATCTCTCACATCCTGCTTCCCCTCCACCCTACCTCCTTTTCTGCCCCCCCACAAAACCAAAACAAGCTTGCTAGATTGCTTGCTACTAGCTTACCTCCAAGCAGATCGAGCCAACCTGCAACTTGCTTCTTCAAGAGATCGCGCGCTGTCCTGCTGCCCTAGCCTTCTTCGTTTCGTTCTCCTTCGTGCCAACACATACATGCATGCACCCAAGCTGCTGCTGCTGCTGCTTCCTGTGCATCACACGGCTCTTCTTGGCTAGCTAGCCTTTTTAGCTAGCTTGGTGTGCCAGTGCTGATTGCAAAGGAGCGAGACGGGAGAATTATTGAGCTAGCGGTTGGCATCAGCCGCGCGCATCCACTATAGAGGCAGGCAGCGACCAGCGAGCGCAAGGGATCGATCGGCAAAGGGCGCCCTAGCTAGCTAGCTCTAGCTGCCGCGCGCCACAGCCATGTGCGACTACTTCCTTCCGAGGATGGAGGGCGACCAGGCCGGCGGCGGCGGCGGCGACCTTACCGACATCGTCCGGTCCGGCGGGGCCATCCCTGGCAACGCCGCTGAGATGTCGTCCACGGCCGCCGCGGACGAGTGGCAGCTCCAGGGGGACCCGATGCTCTTCCCGCCGCTTCCCTCGTCCACGACGTCGGAAGCAGCAGCCTGCTCCGCCGGCGGGGGCACAGGTGCCGACGTCTTCGGCGCCGACCCTTTCTCGGGGCTCGTGGACCCGTTTAGTACCGACTACTCCTCAGGCGCCGACTTCTTGGACGCCATGCCGGACGCGATGGCCAAGGTCGGCTTCGACACGGCTATCTGTGGCGGCAGCGGCAGCGGCTGCGGAGGAGGCGGAGCAGGCGGAGGCGGCCAGCTGATAGACATGAGCCGGAAGCAGCCTCTCTTGCCGCGGGGGGTGCAGATGCCGGCGCTTGGAGTGCTGGCGCCGAGGATGGTGTTGCCGTCGCCGTTGTCGTCGCCGAGGGAGATACGACCGTACCCGCCGTTAGCCGGCGACATGGTCAAGCTCGGGATCACGGCCGGGCAGGTGGCCGGGTGCGCCATCGACGCAGCCGTCGTCGGCATGCAGATGTCTTCGCCTCGCTCCGCGGGCGGGATCAAGCGCAGGTTGGGCAGATCCGCTTGGATCCAACACATCTGCTAGCTTCGCTGCATCATCTTATCTTGCATGTACATATATACAGGGCGTGTGTGTTTGCTAGCTATAGCTCGTGATGAATTTTAGGGAAAGTTCTGCGATCTCTTTTTTTCTGTTCCTTAAGCTTCTCCTCCTTTTGTGCTCTGCAGTCTGCACTGCACATCCACCCATGGAAAAAATAAGAATTGAGGGAGGATTCCAAGGATTCCTCGGGCTGTGCCAGAAATTGGCAAGGTCGTAGGTGTTGCCTACAGCTCGAAATTTTAGGCAAGAGAGTCATCATGAACACATG

GTGTGATCGGTTGGGATATTTCTGAATCGGCAAACGATGCCTACTCATAGGGCTATGTGCATCCAGACCTAGCTGGTTTTCTCCCCCCGGAAAGGCCGGCTTTTGAGTCTCCAAAGCTGATGATTTGTGAAACCCTAGTAATAAGCTTCAATTACTTAAAAAAAACCCAAACTTTGCCTCAGGAGATTATGCGTTATGCGTGCACTTGCAGTTCTGTAAATTTATCACGTGATTACAGCTGTTCATCAAATATTAACACTTGCCGGAAATTCATCTGTGCAGGAAGAACCAGGCAAGGAAGGTGGTATGCATCCCGGCGCCGACAGCAGCTGGGGGAAGACCAACTGGAGAGGTGGTTCCTTCTGATCTCTGGGCCTGGAGGAAGTACGGCCAGAAGCCTATCAAGGGTTCTCCTTATCCAAGGTATGTGTTAATTTGGCACGCATCCACCTTTTAATTTAGCTGGGATTATGATTCAAGACAAATGGGCAATGTGGCTCTATATATTAAGCTTAAGCTGAACAATCTGCTAGCTGGTGACAAATTGGAGGTGAAGCTAGCTAAGCCTAGCTTTACGAATTGCTGCAAGAGTAAACAAAACTATCGTCTCTATATATGGTCCATATAGCTATATATAGAATTCTCAGCTCCACGATTCTTAATTTGTGTCTCCATGATCGATGCAGTTTGAATTAATTAGCTGAAAGATAATTTAATGGTTACCTATCTAGCTAAGCTAGATGTATGGGGGATGCTTCTGATTCTCATGTGTCCAATATATAATTACTAGTTTTTTTATCACTCTATATATGATACATTTTGGAATTTTTTGGAGTGCACAGCGTCGTCCTTATTATTACAGATACGGTTACATGATCGATCAGCAACAGATAACTAACTGTTATGTACCTAAAATACCGGTTAATGTATGATCCATGTTCCAAGGCATATACACACATTTTCAATATAATTTTTAATATTTGTTCCAGATTTTAATTATATATTCTATGCCAAGTGTAGGATGCTAATTACAATCGTCTGGATTGATTTTTAAGATCTGTCTGTACAGTTTTCAAATACATGTGATATGTCAGGTTATCTTCCCTAGATTAAAGTGGAAACAATGTAAAACTATGGCTTTTATTTAGCAAATAAAGCGTATATATAAACGGAACTATCGGCATATATATGAAGTGTGATTTACCTTATATATATACATATGTATATATATGCTGGTTGCAGTATATCACAGCAAGTGATGAAAAGGTGCATGCTTGCTAACTTTAATAATGTTCTGCTTGTCCCATGAAATGCACGATATATCTAGAGGGTACTACAGATGCAGCAGCTCAAAAGGGTGCTCAGCACGGAAGCAAGTGGAACGCAGTCGGACTGACCCAAACATGCTCGTCATCACCTACACCTCTGAGCACAACCATCCATGGCCGACTCAGCGCAACGCCCTGGCCGGGTCAACTCGGAATCACCACGGCAAGAACAGCGGCGGCAGCTCAGGTTCCAAGAGCTCACAGAACGAGAAGCAACAGCAACAGCAGCAACCAAACAACGTCAAGGAAGAGCCCAAGGATCCGGCGGCCACGACGACAACCACTAGCACGATTACTACCACAACTACTAGTACTTCTCCGGCAGCGGTTGTGAAAGAGGAGACGCTGGCAGCTGGATCATCGTCAGAGGCA

CTGGGGCAACAAGTCATGGATACTACAGCGCTAGCAGTAGTAGACCACAACATTGAACTCATGGACCAGGTGTTCGGCGAGAGCTACAAGCCGATGATACCGGAGGCCGGCCACTCTGACGACTTCTTCTCCGACCTCGCGGAGCTGGAGTCAGATCCCATGAGCCTAATCTTCTCCAAAGAGTACATGGAAGCGAAACCCAGCAGCGGTGGTGATCGTGGCCACCATCAGGAGAAAGCAATGTCCAAGGACTTGGATCCATTGTTTGACATGCTAGATTGGTCTACTAATTCCTCATCAGCTGGGAGCCCATTTGAGCAAGGAAAGAGAGGCTAATCCGATCCATTGGCTTATGTGCACACATACATTTGATCAGCAGCTAATAATGATAACCAGAAATTAAAATTACCACAAGATCAGCAAGGAAATTAAGTTGAGAGAGTTTTCCTTCTTTTTTTTTCGCTTTCTTCTTTGCTTCTTATTACCATTGTGTGGTGAGAGAGATTGGATTTTTTTGTTCTTAGATATGAACCTTATTTTTTAAGGTTTGTGTCAGGTTGCTTGGTGTACTTATATGCATGAGATGAATAGCCCCCTGATGAGGATTCTTCTCTCTCTCTCTCTCGTGTAGTAGAGGACTCTCCATTATTGGCATGGCATAGCAGAGTTTATGAACGTTGTCTAACATTCTATGTGCTTGAAGAAGAGGACATAAAAAATGGAGGGCCGGGCCAGGGGTGATGTGAATGTGATAGATGGAGGAATAGAAGAAAGAGAGACCCACAACCTTGAAGGTGATGTGACAAAAAGCAGGGGCGCCGTTGCAACGGAAGATGTTGTTACACCTCATGCTTTTTCAGCTTTTGCATGCAGCTGGAGCTGGGGCCTGGGTGCCCCTGTGCATGTTTGGATTCTCAGA

>SbWRKY29

TACTAGGGATGCAAATCCTGGCGCGATCTCTCCTTTTTTTTTTTATCCCTGCCGCTGCTGCTCTGCCGCCGTGCCCACCACTTGCTGGCAGCGGGTTGACTTCGCATCGCATCCCAATCCCATCGCAGAAGATCGGGCCTGCCGCCTGCGGGTAGAAGAGTCCCCCGCGCCGTCCCGTGCCGCGTCTCTGCAGACCGACGGCGGCCCCCCACCGGCCTGCGCGTCCGAATACTCCTACACATACGTATACGCGTATGGAGTATATATGCGCGCGTGCGGGGGGAGGGCCCTGCTGGACTGTCAGTGCCAAAAGTCAACCCACCCCTTCCTCCTTCTCCAGTCCCCCGCATACGGGCAGGAGAGGAGCAGCACGCACGCGTCCCCTCTGTGCTCGTCAAAGGCTCCAGCGCGGAGAGCCCGGTCGTGGTTCATGCGCGGCGTGCTGCTGCGCATGGCGGAGCACTTCAACGACTGGGACCTGCAGGCCGTCGTCAGGAGCTGCGGCAGCGTCGCCGCGCACCCGGACCCCGCGGCGCCGAGGGCGGAGCCAGACGCAGCGCCGCCGGAGCCGACGACGACCACGCCCGTGGCGGCGCCGCCGGAGCGTCCGCCCGGCGCGCGTGCCACGCCGCCGACGCCGGTGGCTGTGCCTGTGCCTGTGCCTGTCCGGGGACAGGAGCAGCGGGCGCCGCCGCCCGTGGCGGCCAAGGCCGCGGCGCTCCTGTACGACCTCGAGTACCTGGATTTGGATCACAAGCCGTTCCTGATGCCGGTCGTCGCGCCGTCGCCGCGCGCGGGGGACAACGGGCGCGGCGAGCGCGAGGTGATGATCTCCTTCCCAGCGGGGGCCGCGTCCACGTCCGGGATGCAGCAGAGGGCGTCGCCGCCAGGCCGGAAGCCAGGCGCGCGCACGCCGCGGCCGAAAAGAAGGTACCGTACCGATGCTGTTGCTGTGGCTCAAACTCCATCCATCATCGTGTGTACTAGCTAGCATAGAACCATACCGTGAAGATGCCGACACAGATTTAGTGCAAGATTCCTTTTTTAGGCTTAATTTTGCGTGCATGATTTGGTTAGAGAACTAGAATGAAGTGCTGCACGACTGCGATGGCTTATTGCTTTCCTTTTTGTCTTCTCCATGGTTGTGGTATGATGACAGCAAGAAGAGCCAGCTGAAGAAGGTGGTGCGCGAGATGCCGGTGGCCGACGGCGGCTCGTCGTCGTCGGACCCGTGGGCGTGGCGCAAGTATGGCCAAAAGCCCATCAAGGGCTCGCCTTATCCACGGTAACTGCCTTTGACCCTCGCCTTGCATACTTTCCACGCGTTGCACTCACTGCTTGCTCGCCCGGTATAGCTAGCTAGCCTTATCGTCGATCGTTGACTGTCTGCAGAGCACTCAACTTGGTAGGATCCCAACACCTTAATTATTGCCACCTTTTTTAAAAAAAAAATTATTATAGCAAAGAAGAAAAAGGGGGGAAACGCCATCCTTGAGGCTTCACATAGATGTCCTAGTTGGGAGCTTTGATGATAGTTCAGCCACAAGAATAAAACGCCATGCCACTTGGGCATCATCAAGCAGCTTTGGTCGCACTGCACGTCACGTGTACATTTTGTAGTACTACAAGACAATTCGGTAGCCGTACTGCGTGCTCGCAGTTGACCTTGTGGCTGCTGCTGCTGACCCGCAGGCTCAACG

AAGAACGAACTAATGCCCTGTTGCGATGGGATATCTGTGACCGCAGGGGGTACTACAAGTGCAGCAGCATGAAGGGGTGCATGGCGCGGAAGCTGGTGGAGCGCAGCCCGGCCAAGCCCGGGGTCCTCATCGTCACGTACATGGCCGAGCACTGCCACCCCGTGCCCACGCAGCTCAACGCGCTCGCGGGCACTACTCGCCACAAGACGTCGTCGTCCGGCGCGGCCTCCTCTCCCAAGAGCCACGAGCAGGGCCAGGCGGTCGAGAAAGCGGCCGGCCGTGGCGCCGGCGACCGCGAGCATGGTAACAACGAGACGTCGTCATCAATGGCCGGGGAGTTTGGCGGCGAGGAGATAGCGGTAGCCATCGACGACGACGAGTTCTGGCCTGCGGGGATGGACCTGGACGAGCTCTTGGCGCCCGTGGACGACGACTTCGATTTCGAGCACGTCGTGGAGGAGGAAGATGGCGTGCTGGGACGGCGGCTCTCCCTCTAGGTCGGCGCCGTAACTTCAGAACCTTGAATTGCAATAGAGGACGAAAAGATCTCGCCTCGAAGAGAAGCTATGTATAGGCGTCTTTTTTGTGCGCGTTTCTGACAAACAGGATCCCGAAAACTAACGGGCTCAATTTCCGTTGTACTTGCGTAATCTCCGAGAAGTCGGTGCGCATTGATTTTTGTACTCCTAAAATTCAGGCGCACCTTGGCTGTACCGAGCACATGGACCAGGCAACAGACCTGGAACCAAGTAAAACGCCG

>SbWRKY89

CTCTCTCGGCTCGTCGCTCCGTCGCCCGCCGCACGGCACGGCACTACACATCCACTGCAGTCTGCACGCGCCATATCTATCTATCTATCTATCGATAATATATATAATAATACATGCTACTGTGATGACATACACACGCCATGCCAAAGTCAACCGTCTCCGTATATATGAACCCTCTCCCGGTCTCCCCCCACCTTGGCACACATACACACGCACGATCCATCGCCATGGACTGCTCCAACGACTGGGACCTGCAGGCGCTCGTGCGGAGCTGCGGCGGCGGCGGAACGGCAGCCGCAGCAGCGTGCAACAGCGGAGCGGCGCCGACGGCGACGAGGGGAGGGTACGACGCACCTTCACGGGAAGCTGCTGATGATGCTAGCGTCGTCGTCGGCGGCGGCGGCCGTGTGGTGGCAACGGCGGCGGCGGGACAGGAATTCCTCGGGCAGCCGGTGGCGGCGTGGCGGCGCAACCTCGACTACTTGGACCTCGTGGACCACGAGCTGCTGCGCATGCCCTTCTCCATCACGCCATCGTCGTCTCGAGAGACAACGTCTGGTGGGGCGCCTGGGCAGCAGATGATCCGGCAGCCCAGGAGGCAGCCCGGCCGCAAGCCGGGGGTTCGCACTCCCAGGGCCAAGAGAAGGTACAATACATACGAATACGACTTGTCGACTTCCATTTCATTTGCGGTTCATTAGCATGATTTGTTTGCATGCATGATCGACGAACTGAATGATATTGATGCATGCACACGCATGCTTTGTATCAGCAAGAAGAGGCAGGTGAAGAAAGTGGTGTGCGAGGTGCCGGCGGCCGGCGGCGGCGTCTCCTCGGACCTCTGGGCATGGCGCAAGTACGGCCAGAAGCCTATCAAGGGCTCACCCTATCCACGGTATATACGTGTATTGTTTGTTTCCACATGTACAGTCCATGCATAAACATCTAGAAATATGATAGCTAGCTAGGTTGCATGATGCATCTGGAATTCTAATCCAACTTTTGTGCATCCATATCATCGTCAGGGGATACTACAAGTGCAGCAGCCTCAAGAGCTGCATGGCCAGGAAGCTGGTGGAGCGCAGCCCGGCCAAACCCGGGGTGCTCGTCGTCACCTACATCGCCGACCACTGCCACGCGGTGCCCACCATGCTCAACGCACTGGCCGGCACCACGCGGAACAGGCCGGCGGCGGAGTCCCCCGACGATGGTGACCACCATCACCACCAAGAACACCATGACCACGAGACGTCGGACGGGGCGCCGGCGGCGTCCGCCGATAACAAGCTCGACGACGACGGGGCCGACGCCGCGTCCACCATGACCGTGGAAGAGAACGACGCGTGGCTGGTGGATCATATGGCGCTGGAGGACGACGTCGTCGACGGCGACTGCCCATTCGATGATTTCTTGTGGCCGTTCGATGACGACTTGGATCAGTTTCTCGACGTCGACGGCGGCGGCGTTCTTGGACGCCGGCTGTCGCTGTAGCTGGGGACATGCCGCCCGGGCAACTGATGGATCATCGTTAAGCTATATATTCATTGTTGACTACTGCAAATGGATTGCATATATCTTCTATGCTCTATCTGTAGATGTAGATAATCTACATCTCCTTCAGCTGGGACCTGGGAGACAGGTTGGCGTGTAGTAAGCGGAATGCATTGCGCCTTGCAGGTGCAGCGCCGCAATTAGACGAA

AATGCAGACCGTGTACATTTGACCGAGTTCTGCTGATGTTATGGTGTTTTTGTCAACAATCTCTCTCTCGTCCTTGATGGAAGAATAAATTGATTTATTTATTGTTACACAAGCACGCACGACACGGCCATGATGTTTGAATTGTTTATATATCCAAGTTCGTGGATGTGGCGACATGTAT

>SbWRKY46

AGGGGAAAAAAAAAACCCAGAGCATAGACCAGTAGCCCTCACCCCTTTTTAGGACAGGATCCAATGCCTTTGCCCACCACACTCTCTCTCTCTCTCCTTTTACTCTTCCCATAGTCTCCTCACTCTTCCAAAACAATCAATAAACTAGCAAAAGTCAGCGCCTCCCCCTTATCTCCTCCTCCGCCCGTTTCCTCCAGCTAGCCCCCAAGAACCCAACCACTCCGCGTCGTCGTCGTCGTCTGCTGTGCTTTTCCCAAATGGAGGAGGAGCGCTGCTTCAACAACTGGGATCTGGACGCCGTCGTGCGCCTGGGCTGCCGCCGCCGCCTCTCCCCGCCGGGCCAGCCCGACCCGTTCGCGTCGTTTCTGCCTCCGCCGCCGCCGTCGCCGCCGCACAAGGAGAAGCCCGTGGTGCCAGCGCCGGCCGCCAAGGAGCCAGAGCCATATGCGGCGTGGCGCTTCCCTGACCTCGGTGCAGCTGGCGGCGGGCAAGACGGCGACGAGCTCCTCAGGGCCCTGCTAGCCGCCCCCCCGCCTCCCCCGCCTCAGCCTCTGCCAACGCCAACTCCGCTGCCTCCCCCGCCGCCACAGCAGCAACGGCAGCCGGCTGTGGCGGCGGTGGACGTGCCGCTACCCCAGGCGCGTCCCGCTCCGGCGAGGGCGCAGCCGAGCGGACGGCAGGTGCCCGGTGGCGTGCCAAGATCCAAGAGAAGGTACGCTTCCCTGCTCGAGCCCTCGACACGGCGGCGCTTCATTGACTTGTTTCTTTCTTGGGGAGGTTGAAAGCTCGGGTATTTTGCAAGCTAGCTGCTGACGCTGTTTTCCGTGCACTCCGCAGGAAGAACCAAGTGAAGAAGGTGGTCTGCCATGTTCCGGCGGACGGCTCGTCGTCGGACGTGTGGGCGTGGCGCAAGTACGGCCAGAAGCCCATCAAGGGCTCTCCCTACCCAAGGTCAGTAGTCAGCTAGAACGATCCCCTTGTCTCCCTGTCAGCTTCTTGCCGGCTCGTCGTCGGAGGAGGAGTCCGATCTCACCGGCGGCTCGGCTGCTCTGTTTCTTGAACGAACAGGGGATACTACCGGTGCAGCAGCTCCAAGGGGTGCGCGGCGCGGAAGCAGGTGGAGCGAAGCCGCGCGGACCCCAACACCTTCATCCTCACCTACACCGGCGAGCACAACCACGCGGCGCCGACCCACCGGAACTCGCTCGCCGGCACCACCCGCCACAAGTTCCCCGCCTCGGCGACGCCTCAGCCGCCGCCGCCGTCCGTCGTGGTGGGCGGCGCCGGCGCCGGAGCCGGAGCCGCCCCCGGCGACGCGCAGCACCAGCACCAGCAGCCGAGCCCGAGCCCGACGTCGACGTCGACCGCGGGGCTCTCGCCCACGACGCCGCTGCGCACGCCGTCCATGGAGGAGGACGACGAGGAGGAGGAGGACGAGCTGCTGGTGGAGGACATGGAGATGGCCGGCGAGGACGAGCTCCTGTTCCTCAACCCCGACGCCGACGCCGGGGCACCCATGTCCTCGCTCTTCGACGTCGTCGACGAGCCCTTCCTGAGCTCCCCCTGGGTGACAGCCACCAGCAGCGCCGGCGAGCCAGCCACAGGGGCAGCCGGCGCCGGGAGCTGA

>SbWRKY92

ATGGACGACGGCCGCGACGGCAACAACTGGGACCTGAACGCCGTACTGCGCTCCGGCTGCCATGGTCCCATGCCGCCGCCGCCGCCGCCGCCGACAAGGACGGCCAACAACCCCGTCGCACGGTACGCCCCGCCACCGCCAGCTCAGCCGAGTTATGCCTTCACTGTCTTGGCCGGCGGCCTCGGCCATCAGGCCATCTCTGTGCTTCCTCAGCCGCAGGCGCTGGATCAGGATCCGCCCCACGCTGCAGGGCGGGGTCCGAGCCTGGATCTGCCGCTGCTGCCGGAGCCGGACTACACCGCAGCCGTCGGCAATACACCTGCGCCACTGAACCCACCGTGGCCAAGAAACGAAATTCCGGTGCCGTCCGTCCAGCAGCGACCAGCCGACAAACACAAGACGCCGCCTTCCAGCGGCTGTGACGCTGCAGAAGGGTCCAGTCGATCCAAGAAAAGGTACGACTAAATCTTGACTCTCGTAACTATTTATTTTTTTAAAAAGGAGACATTCATGGAAAGTGCCCATCCTATATATGTTTAATCACGTGTGTACGTCGTTGCTTTTGGTCGTGGCGGTACGCAGGGACAACAGGACAACCAAAGAGAGCAAGGTGGTTCTGGTGCTAGCCGAGGATCCGACGCCACCAGACTCGTGGGCGTGGCGCAAGTACGGGCAGAAGTCGATCAAGGACACCCCGTACCATCGCAGCTACTATCGATGCAGCACCGACAAGAAGTGCAAGGCGCGGAAGCATGTGCAACGCTGCCTCACCCAAAGCTTCCTGGCCGTCTCCTACATCGGCGAGCACAGCCACCCGATGCCGCTGGCCCGCAACGGCCAAGCCGGCACCACTCACCAGAAGCCGCCGCCGCGGCAGCCTACGTCGCCGTTCATCAGGACCCCCGCCAAGGAGGACCAGCCGCATCATCAGGCTCCAGCTCCGCCGCCGGCCACGTCGTCGTCGCCATTCGCCATGATCTCCGCCGCGAAGCAGACTCCGGCCCCGCCGGCGTCCGCGTCGCCCTCGCTGCAGTCTGTTTCGGCGATGCCCCTCGGGATCAAACACGTGGAGCAGCCGCCGCAGGCTTCGGCTCCGGCTCCGGCTCCGGCTCTGGCACCGTCGGCGGCGTCGGCGTCGCCCATGATGTCCCCTACCACGCTGTTGCGTCCGCCGTACGCGGACGTTTACAAGGAGGGGGACGACAGCCACAGCCACCCCATTCCATTCGTCTGCAACTGCCTCGTCTGCACGACCCGCCAGAAGACGCCGCTTCTGGTGCCTCCGTCGTCATCGGCCATCGAGACCCGTTCCTGGGTGAGCCAACCGGCTCTGCTGGTACGCGTGCCCTATTCGCTTTCACCCAACACCACAATGCCGCCGTCGATCGCCATTGCTTAATATCTTAGCCGTGCAGGAACAGCAGCAGACTCCAGCTCTACCGGCGTCCGCATCGGCCTCGCTATCATCAAACACGCCGGTATCGCCGCTGCTCAGCTTCCAAGCCGAGGAGCGGCCAACTCAGGTCCCGGCACCGGCTCTGCCACCGTCTGTATCGACGCTGATGTCGCCTAGCGCGCTGTTGCCCCCGCCGTCTGTGGAGTTCGACAACGAGAAGGACGATGACGCCGTCGCCGTCAGAATGCTGCTCAATGACATGGACATGACTCCCGAGGATGCACTGAAGTTTGTTAACCCTGAA

GAAGAGCCCCTGGATGGCGTTGGCGACGACCTTCTGATCCCGACGCCTGAAGAGCTAGCACCGTTCTACTACGGTGATGAGGAAAACATGCTATACCCGATGGCTTCTGAGCCCGCATCAGGAGGCAGCCGCAACACTAAGGCATGA

>SbWRKY93

AAACACTCTATTTATCTTGCTACCACCCTGGATTTCTTCTCTCTCCCACCTCTCTTTGACATTTTCAACCAAAACGCATGCCAGCATCGGCGCCACGCCACCCCGTTGATCCTTTTTAACTTTGTTCAAGTTGTCGTACTGTCCGTACAACCCAACCACCCCCTCTCTCCCACCCGCACCCCCACCCCCAAGCAAGTCAACGTCTTCTAGCTCTCTCCTCCTCCATTAGCCACGAACTCAAGAAGCTAGGCTATGGAGGGCGATCTGCGTTGGTGCTGTGGTAGCAGCAGCAACGACTGGGACCTACACGCCGTGGTGCGCCTCGCCAGCTGCAGTGGTGGCAGCCGCAGCCGCGTCACCTCGCCGTCGCCGTGGGCCTCGGACGAATCATTCTCCTGCCTGCCCCCGCCGCCGCAGTCACAGAAGGACGAGGTGACGACGGACGCCGCAGCGTTGCAGCAGCCCCTGATCAGCCCTGCCGTCGACGACCTCTGCGGCCTGCAGCAGGCCTTCTTGGCCGCCACGCCGCAGCCAAGAAGCGAAGCGCCGCCGCCGCAGCCGCCGGCGAAACCACGAACTTCCTACCGCAATAACGACGGCGGCGTTGGCGGCGGACCGACACGATCCAAGAGAAAGTATGTATCAGCCGTGCGCCCATCCAAATGAAATCTCTATTTTCGATTATAACCTCGCGCGCGAAATACTAGCATCTAGGAACGACGTACTCCAGCTCTTTAGATAGGCATAGCATAGGATCTCACACGTACGCCTGTATTCGTAGGAAGAAGAAGAGCCAGGTGACCAGCAAGGAGGTGACGCGGGTGCCTGTGGGCACGTCTGCGGACCCCTGGGCGTGGCGCAAGTACGGGCAGAAGCCGATCAAGGGGTCGCCGTACCCGCGCGGCTACTACCGGTGCAGCACCGACAAGGACTGCAGGGCGCGGAAGCAGGTGGAGCGCTGCCGCACCGACGCCTCTACCCTCATCGTCAGCTACACCGGCGAGCACAGCCACCCAGTCCCACTCCACCGCAACGCCCTCGCCGGCACCACGCGCAACAAGCCGCAGCCAGCACCGTCCACCTCCCCAGCCGAGCAGCCTCCAGCTGCGTCGCCGATCGTGGGAGTGGAGTACGAGGAGGACGACACAGTCGCCGCCAGTGTGCTGCTCGAGGATGCCGAGACGGAGGGAGAGGAAGACGTGCTGTCGTTGTTCCTCGAGCTCGCTCCGAGTCCCAGCAACGGCAGTGGCTCCAAGGACGTCATGGTATCCACGGAGCTCCACAATGGCAGAGGCTCGCAGAAAGTCGTGGCACTCTCGAAGCTCCACGAGTTCCGACATCCAGCAACGACGAGCAGCAGCAGAACGAGCGATGGCTTAGGGGCGGCTCCGGCGGCCATGAACGTGACCCACGAGAACTGCCCTTTCTCAGGACTCCGACTCACGACGTAGGAGGCTGTGCAGCGGTGGCGGCGGCGACCAACTGGGGCTGGCTTGTGCTGCATACATCGATCTCGAAGCACACTGCACACAGCACAGGCGCACACAGAATGACAGAATGCTGTTAATCGTCTTGGCTTATTGCGGCAGGATGTACAACCATTCTGAACTCTAGTCAGCATCAAAAGTGTCGCTTTTTACTAGTGTTATAAGTGCTTTTTAATTTAGCTAGTTTGTGTTAAGTGCATGTATAATGTATAA

TTATAGAATAATCAACCTAAAAACCAATTGTTTTTTAGACTTTTGTATTCTTAATTTATTTGTCCTTTAATCAATAAGATCTTCTTGATAGCCCACTCCTTTTTCTGGAATTAATTTCCTTTGTGCTATATACAGATACAGGTCAAATTAAGCAGGTGCAGTAAGGAAGTCCAAGTATATATATTTGGATGATAGCCTACTACACACTACGATATCGGATCTCCGGGGATCAATCCAAAAGCAAGGAAAACAATGTCTATCAGGGCCGTGTTTAGTTCCGAAATTTTTATTTTTATTTGACAAATATTGTCCAATCATG

>SbWRKY2

AAGACATCTGGGCCGTCCACCTTACCGACGGCCCTGATCATCACCCTCGTTGACTTTGCAAAATTTTATTCCGATCGTGCTCCACCTCTCACTCCCAAATGGAATATCATATACTCACCAGAATTACTTCCTGCTCAAGTGAAGGAGAAGGACCCAATGAGACTGCCTCCTCACATATGCTCTTGCTCTCCCTGTTTAAGATCACACAAAGATCTTGCAAAGACTCTTTCCAACGCCACCACCATTGCTGCAATTCTCGCAACCTTTCTACCTCTCCACTCAATCATAGTCATAACATAGCACCTGGAGAAATTTCATGTGCAAATCTAAGATTGATTAGTGGTTGCAATCTCACTGGGTAATTCAATCGAGGCTACCAGTGTGCTACCTACTAGCACTCGGATTTGCCGGCGGTGCAAGTTTTCTGATGAAAGCCATGGAGGTAGTGGAGGAAGCCAACCGGGCCGCCGTGGAGAGCTGCAAGAAGCTCGTCGCCGTGCTCTCGCTCTCCGGTGCCGACGCGTTCCGGCCTTTGCCCGTGGCCGCGGAGACCGACGAGGCGGTCGCCCGGTTCGGCAAGGTGGTCGCCGTCCTGAGCGACAGGCTCGGCCATGCCAGAGCAAGGGTTGCTGGCAAGAGGAGCCCACCGGCGCCTCCCGTCGATGCGAGCTGCCTCTTGGAGTACCACCCGTCGCTGGCAGTGGCACCGCGACACACTACCAACGGCGGCCATCTGCTGGTCAGTGCTACTTCTCCTCCTCCTCCTCCGCCGCCGCCGCCGACGACGACGACGTCGCTGCTTGCTAGCATGACGATGCGAAGCGCTGCGGAGCCGATGACGATGAGAAGCCAAAAGGCGGAGGTAGTGGCGCCGGCGGTGCTGGTGTCGCCACCTTGTGCCAGCAACGTGACACTGACGCCGGCCCCGGCCAAGAAGTTTGACAGGAGCATGTTCCTCGAGACGTCGCTGCTAGAGTTAAACAACTCTTGCAGCGTGCCTCCATCTTCGTCGCCGGCCATGGCGGTGCAGAAGAGCAGCCCGAAAGTCGCCGCGCCCAATCCGTGCACCAGCACCCCCCACATCCAGTTGCAGCCCACCACCCAGTTCCAGCCACCGCCACAGCAGCAGGCGGCGAAGAAGCAGAAGAGCTTCCAGTTCGACCAGACGCCGAGCGGCGAGCAGTTCCACATCGAGGTCCCTGTGCCGCTGCCCCGTGGCGCCGCCCCCGCCGCGAAGGAGGTGATCAGCTTCAGCTTCGACAACAACTCGGTGTGCACGTCGTCGGCGGCCACGTCCTTCTTCACGTCCATCAGCAGTCAGCTGATCAGCATGTCCGACGCCGCGACGAGCTCCGCCGCCAGGCCCGCCACGGCGAAGAAGATGTGCGGCAAGGGAGTGGAGGACGGCGGCGGCGGCGTCAGATGCCATTGCCCGAAGAAGAAGTGAGTGGCAGCCATCTGCATTATCCTTGTTGCAATGCAAGGTGGCTAATGGATGATGGGAAATCTGTGCATGCAGGAAGCCGAGGGAGAAGAGGGTGGTGAGGGTGCCGGCGATCAGCGACAAGAACGCCGACATCCCGGCGGACAACTACTCTTGGAGGAAGTATGGGCAGAAACCCATCAAAGGCTCTCCTCACCCAAGGTGCGTTGCATCTTGAGACCATTTCGTTCTCTTCACGCCATTGCTTTATTAGTTA

TAATGTCCATTAATCTGCCATGCCATCTTGTTTGTGCTACTCTCTGCTACACTGCCATTCTTTGATCATTATTCAGAAAAATTGCAGTTTTGTACGACTGAAAAATTCTGAACGAGAGCAGGAGTACTGATGAACATTTGATATGCAGGGGATACTACAGGTGCAGCAGCAAGAAGGACTGCCCGGCGAGGAAGCACGTGGAGCGGTGCCGCAGCGACGCCGCCATGCTGATCGTCACCTACGAGAACGACCACAACCACGCGCAGCCGCTCGACCCCTCCGTGCTCACCGCCGCCAACGCGGAACCTTGAGAGCCCATCGGACGGAGACGGAGACGGTGCCGGAGTCTCTGCCAACGTCGTGCAGGAGCGACGAATTCTTTTCTTATTTCCAAACAAGATGTACAGTGGTGACAATTTGATTGCGTTTCACTGCTCCCGTTTCTCCATGTCTTCTGTTTAGACGAAATTCATTCAGATGAGGCCTCTAGTTTAATTTGTGCATGCCAAAGAGTGTAATCCTACTAGTTGATGTTTCACTTTCATCCGCCATTCAGAATTCACGTGTCACCACCTCGTTTCG

>SbWRKY36

CGAGGCAAGGCGCCAAACCAACAACAAAAATATCGCGTACGCTCACGCCTGCAGCCGCCCGCTGGTGCCGCCCGGCTGCCAGTATGGATATGGCTCGCTCGCCCTGCGTGGGCTGCTGCTGCCCTGGGCGCCCCGGTGCTCCCCGATGACACCAGCCAGCATCGTTCGCATAAAAAAAGGCGCCCACTAGCGCGCGCACCCCCGCATATACAGACACGCCGCGCATCGCGAGCGAGCGAGAGAGAGAGAGAAAGAGAGGAGGGATCATTGAAAGCCGCAGCGCCCATCATCGATCCGCCCCCGGCCCGTCTGTTCGCCACCATTTCCTTCACCCCCTTCTTCTTCAATCCCCTCCATCATTCGCTCCAATACTCCGCCCCTCCCCCCCGCCCGCTTTCTCCACCATCAGGGCGAAAATTTTATGCGCGCTTTGGGAAAGCGCGAGTTAGTTTTGCTTTGAAGCTGCCTGCCTTTTTATCCATCCGGGGCTGCGGGCGTCGGGAGAAGCACAGGTTCGAGTCCGGCTCCGTGTACTCCGGCCGGGCGTGGGAACCGAGGCGGCAGCGGCGGCAAGAGAGAGCTAGAGAGGGGGATCTGATCGATGTCGTCGGCCGAGGACGGCTACTGCAGCTCGGATTCGCCGCGGGCGGAATCCCCCGACGAGCCGCTGCTGCCTGCGGCGGTGGCGGACGCGGACGCGGAGTCCCCTCGCGCCGCCGGGTCCGGGATGAACAAGCGCGAGCGCGACCTCAGCGACCTCCCCGCCTCGCCGTCGTCGCCGCTGCCGCCAGCTAAGCGCAGGTACGGACAGGATTCATGTGTCGTGTCTCATCACGCGTGCCTGCGCAGCGAGTATTCTGCGCGGCGCGCGCCCGTGACCCTGCTGCTGCGCTGGTACGGGAATCTAGAAATCAGAGCTGCAAGCAAAGATGGCTGATTTTGGCTCTTGTCTCTTTCTGTGCGAGCAAGCAGCCGGAGATCGGTGGAGAAGCGGGTCGTGTCGGTGCCGCTGGCCGAGTGCGGGGACCGGCCCAGAGGGGCCACCGGGGAGGGGCCGCCGCCGTCGGACTCGTGGGCGTGGCGCAAGTATGGGCAGAAGCCCATCAAGGGCTCCCCCTACCCACGGTACGTACCTAGCTGCTGGCCTTCTGCTCATTCATGTGCGCAACCTAATCAGCATCTGCATGCTAGATTCCCGATTAGCAGCCTGGTAACCTTGCTTTCTGCTTGTGTTTGTGCAGTGGGTACTACCGTTGCAGCAGCTCCAAGGGGTGCCCGGCGAGGAAGCAGGTGGAGCGCAGCCGCGCGGACCCCACCGTGCTGCTCGTCACCTACACCTTCGACCACAACCACGAGGCGCCGCAGCCGAAGAGCAGCAGCTGCCACCAGCAAGGCAAGCCGTCCACGCGGCCGCCGGCGCCGAAGCCTGAGCCCGTGGTCGAGCAGGATGAGCTTGGTCCGGAGCATGAGCTGGCAGAAACAGAAGTGCCAGAGCAGCAGGAGCCGGTGGAAGAGGAGCAGGAGCAGAAGGTCGTCCCAGGTCTGGCCGGGCCGGAAGCGGAAGCGGAAGCGGAACCAACCGCAACGGTGGCGCCGGCGGCAGCCGAGGAGGACGAAAGCTTCGACTTTGGGTGGTTCGACCAGTACCCGACGTGGCACCGTTCGGCGCTGTACGCGCCGCTGCTGCCGCCGGAGGAGTG

GGAGCGGGAGCTGCAGGGGGAAGACGCCCTGTTCGCGGGGCTCGGCGAGCTGCCCGAGTGCGCCGTCGTGTTCGGGCGACGCCGCGAGCTCAGCCTGGCCACCACCGCGCCGTGCTCCTGATGCTGATGGGCTTTCGACTATTTTTACATTTGTTTTCTTTTGCTCCCTTTTGGCACTAGTACATAGTAGCTCTGATCGATGCACGGGGTTTATCTGTTCAAGGCCGGCTGCTGCTGCTGCTGCTGCTGCCTCCCCGTGTTGATTTGGTGCGCGCGCGCGCGTTCTTGTTCGTTCGTGCGTTCGCCGTCCAAAGGTTGTCCCGTTTGCGATGTGATTCCAACCAGAAAGCAGCGAAAGGTTTGGC

>SbWRKY34

GCTCGTCTGCTGTTACTACGCACCAACCACTCCAATGCATAATACATATGCGTACGTTAATTAATTCCCGATCCATCCAAGGAATAAGCAAAGCAACCGCTGACAGATGAGGGACGATGATGGATCATCAAGAGACGGACATGGATGGACATGGGGATGGGATAGTTGGGGAGACGTCGCCGTCGCACGCTCGCGCTCGTCGTCTCAAGCAGCTTGTCTGGCTGTCTCTCCTCTTTGTTTAACTTCGCGCCCCGCCCGCCGTCTCCAAGCCTAGCTCAGCTCAGCCAGCGTGCGCGCCCACAAAACCCCACGTCCTCCCCGCCGGCCAATCACACTCACACCGCACGAACCCAGGACGTCGCGACACCGTCGGTCACCGCGACGTACGTAGACGCAGAGCCTCGTGGTAGCTAGTTTAGCTAGCTCACTTCCACACCGCACCGCGCGCCGTGTTGTTGCCTTCTTTTATATCCCGCGGGGATGCGCCTGCCTCGGGGCAGCTGGTGACATCGGATTCATTCGGATCAGGCCAAGAACTGCTCCGGCAAACCTGCCGCGCCCCGCCCGTTGTCTTGTCTGCTGTTTCCGATCGACAACGCCGCGGGCGCGCTTTCTTCTCAAATGGACGCCGAGTGGAGCGACGGCGCGGCGGCGGCGTCACCGCCGACGGTGTCCGGCGGCGAGAGCAAGCCTGGTGCTGCTGGTGCCGTCTCGTCGTCGGCTGATTGCCCAGGGTCGCCGCCGGTGTCGCCTGCGCCACCGTCGACGACGTCGCCAGCTGCTGCTGCTGCTGCTGCCGGGAGCGGGAGGAGGCGGTCGGCGAACAAGCGGGTGGTGACCGTGCCGCTGGCAGACGTCAGCGGGCCTCGGCCTAAGGGCGTCGGCGAGGGCAACACGCCCACGGACTCGTGGGCGTGGCGGAAGTACGGCCAGAAGCCCATCAAGGGCTCGCCTTTTCCGAGGTACACACCACACGTACTAGAAGCAATCGTAATCAATCATGCATCCATGGTTACATGGCCGCCCCGTGTGCTTCGAGTTCTTGTGGACGCATCACGTATTATTAGTCTAGATTTTTGTCGAGTAGTTTTGCTGATCTGGATTTCATTTGGTTTTTGGGGTGCGGCGGGGCAGGGCTTACTACAGGTGCAGCAGCTCCAAGGGGTGCCCGGCGAGGAAGCAGGTGGAGCGGAGCCGGGCCGAGCCGGACAAGGTGATCGTCACCTACTCGTTCGAGCACAGCCACTCCGACGCCGTGGCGAGGGCGCAACAGAACCGCCAGCAGGCCTCGAAGCCAAAGGCGGTCCAGCGGCAGCCAGTCCCGCCGGAGCCGGCGGCGGAGTCCCCGTCATCCGGAAGCTACGACGTCGCTGCTGCCACGGTTTGCGGTGCCGGCGCGCCTGCTGCTGCTGCAGCCGGGACCGAGGTCGGCGGCGCGGCGTCCGTCGAGGTGCGCGACGAGTTCAGGTGGCTCTACGACGGCGTGTCCGTCACCTCCTCGGCGTCGCCCTCGGACGTCGAGGCCGCGGACGAGATGCTGTACGGGGCGATGTTCTTCGGCGCCGCCGCCGCCCCGCCCGCCGCGCCCCTCCCCGACGAGTTCGTCGGCGACGTCGGCGGGCTGTTCGACTACGGGGAAGGAGGCGGCGAGGAGGACGCCATGTTCGCGGGTCTCGGCGAGCTGCCCGAGTGCGCCATGG

TGTTCCGGCGGCACGCCGGCGACGGGCTTTCGGTGGCCGGCGGGGTGAAGTGAAGGTCGAGCAGCCGGCCGGCGGAGAGCACAGCCGCAGGCCTGGTGGCCTGACATGTGGGGCAGGCTGGCCGGACTCCAATAAAAACCAGATAAACTCATGTAGAGCTCCTTGATCTATCTACTTTTGGCTTGTTGTTCTCTTCCCTCTTGTCATCACGTAGCCTTTTTCTTTCATCCTTCTCTGATTGGATGTTTATACACAGACACAGACACGGTAGCACATATAAAAACTTGAGGCATTTTACATTTGCATAGAGTTGATTCGGTGGGCTGGCTGGCATGAGGGGTTTGAAAAGGACACACAGATCTTTTCTTGTCAATGGTTTGGTTGAAGCATTTTGTTTTCTGTGCAAGAGCACGAGAGCCAGACAGAGAGAAGCTCAAACAATCAAACCAACGTTTTCTAGCTAGGTCACTGACATGTGGTACAGGCCGGACACAGACTCCAATAGAACCAGATAAACTCATTTGAGCTCCTCTTAATCTATCTACTTTTGAGTTTTGGCTGCGTGTTCTCTTCCCAGTTGTCCCGTAGCTTTTCCTTCATCCTTCTTTGATTGGTTGTTTATATATTCTACAGTACACGGAAACGGTAGCACGTAGTAGAAGAACAAAGTTAAGGCATTTTTGCAGTTGCACAGATCGAGTTGATTTGAAGTCGCAGAAAAAAAAAAGATCAAGTTGATT

>SbWRKY5

AAAAGCGAGGGCACTGCCTGCGCTTCGCTGCTCTCCCCGCTGAGCGAGGGCTGCGCCAGGCACCCTACCAGTATTTGTTTGTTTATATTATATATATATATATAAATCGTCGCTCACTCCACTCTGCCGAGCGCCGGGCCCTGGACTCACATACCACTTCGCACTTCCGCAGACGGACCACGGGCGGGGGGGGGCTGGGCTGCTGCTCCTTTGGCTTGGAACCAGGCTACCAGCCGAGGGGAGAGCGAGACGAGAGCGTCCCTGTCCTCCTCCAGCAGGCCTCCACCCTCCCCGTCCCCGAGCTCGTCGTCGCCACACCAGGCGAGGCCGCGGGGCTTCGCTACTCGGAGAGGTTTGCGACCTTCTTCTCCTGCTCCTTCTCTGGTCGGTTTTTTGCATGCGCGTACGTGCTCTGCTGCTCCCTCCCCTGCCGATTTGGTGCCGAGCTTTAGCCTGGTGCCCATGGATGGGGGGCGCGAAGCAGCCGATTGCTTGCTGGCGTAGGGTTTCCCATCTTTGATATTTTTCAGTGGTTGTATTTTACGGCGGAGGGGGGAGAGGGAGAAGAGCAGCAGGGTGGTGGTGGTGGTGGTGGCGGCGATGGCTCCTGGCCTTTTTTTCCCCCTGATAGCGGCGTCGCTTAGGTCTCAGGTGTTTGTTGGCTTGGCTTGTCATGTTGCCTAGTTTTAGTACTGATTTGGACCATTTAAGCGCCATGTTCCCGTGCTCGAATGCCCAATTTTAGCTTACCCGGTCGCCGAAATGCCGCCTTCATGTGCCTCATCATTGTGGGAATCTGCTGCTACCTTCTTTTTTTTTATTCAGGCTGCAAATCTAGGGAGGCTTGCAAATCTTTTATGGCATGGAATGATTAGAGCCACCGCGCGCGTATCAACCTTTGTCACTGTATTTTACTTTTGTGTACGGAGCGGAATGCTTGTGCTGTCACCGCAAGAGATTATATTGCTCTGTGTGCTGCTCAGGCAGTGGTTTGATTTCAGTCAGTGATTGTTTGGTTGCAGGGAGGGCGGAATGGGGCCGTCGTCCATTCAGGAGATGGAGGAGGCCCGAAGGACCGCAGTGCAGAGCTGCCACCGGGTGCTGGCGCTCCTCTCCAACCCGCACGGCCAGCTCGTCCCCAGCAAGGACCTCATGGCTGCCACCGGGGAGGCCGTCGCCAAGTTCGGCTCCCTGACGGCCAAGCTCACCAACTCCAACTCCAACTCCAACGGCAATGGCCTGCAGCTGCAGGGCCACGCTAGGGTCAGGAAGATCAAGAAGCCCCTGCCCATCTTCGACAGCAACCTCTTCCTCGAGAGCTCTGCGGTGGCCGCCGCCGCCGCCGCCACTGTGGCCAAGACGCCCAGCCCGAGCCCGATCACTGGCCTCCAGCTGTTCCCGAGGTACCACCAGATGGAGGGCTCGTCGTCTAAGGATCCTGTCAGGATCCCTACCCAGTTCCCCAAGAGGTTACTGCTAGAGAACCCGGCTGCCGGTCTGGAGGGGCTGCCGTCCAAGGCCCCTCCGGTCCAGATGGTCCAGCCGGTGTCGGTTGCGCCTCCTGCAGGGACGCCTACCCCGGCATTGCCCGCTGCTCACCTTCATTTCCTCCAGCAGAACCAGAGCTACCAGAGGTTTCAGCTCATGCATCAGATGAAGATTCAGAACGAGATGATGAAGAGGAGCAATCTTGGTGATCA

GGGTGGTAGCTTAAGCGGTGGTGGTGGTGGTAAGGGTGTGAATCTCAAGTTTGATAGCTCGAATTGCACAGCGTCATCGTCTCGCTCCTTCCTTTCGTCTCTGAGCATGGAAGGGACTCTTGCGAGTTTGGATGGAAGCCGGGCCAGCAGGCCATTCCAGCTAGTTAGTGGCTCTCAGACATCTAGCACACCGGAGATGGGCCTGGTGCATAGGAAAAGGTGCGCTGGTAGGGAGGATGGGGGTGGTCGGTGCACTACCGGGAGCCGGTGCCATTGTTCAAAGAAAAGGTTGGAATTCTTGGTTATAAACTGATAGGTGACTACTAGCATTATGGTTTTTAAATTTGTTTCTAAAAATCATATTTTTCTATGTAATTTCAGGAAGCTTAGGATAAGGAGGTCCATCAAGGTCCCTGCAATAAGCAACAAGGTTGCAGACATCCCAGCTGATGAGTTCTCGTGGAGGAAGTATGGGCAGAAGCCAATTAAGGGATCCCCACATCCTAGGTACTGAATACTTGTCCATCTGTGACACTTTTAATCTTTTTTGTGTGAATTTGAAGTGTTTCTTACTAGAGTATATGCGGTCCATATTTAATGATATCTGCTTATATCGATTTGGCACTTGCTAATTTATAGTGTAGTTGTACCCACTGACTTGATGCTATTTTAGTTGCATACGAGTATACGACTACCATTTCTGTTGAGTTCAAAGTTAGTATTTCTCCTTTCCATATTCTGGAATGCATGATCTCGCATGGAAGCACGCACCACAAAGGATTACTGATATGAAAAATCTGTCTGAACAAGTGTTCCCCCTACTCTTTAGATGCTGCTTATAGCATTGGGTCGTTTGGCTTCTAATCAAGTTATTCAGTTTTGTTTCGTATTTTACAATAAGCATACTGCTTACTGAGCATACATGTGGTTTGAAAGAAACCTGTTCTAATTATGGTGGCACACCTGTTGCAATCCGAGGAAGGTTACAACTGGCTACCACTTTTGCAACACAGATCACTGTCTTGTACCTTTGTCAAATATGCAGATCACAAATACTCACAAGCTCTCTGCCTTGCAGGGGTTATTACAAGTGTAGCAGCGTGAGAGGGTGCCCCGCGAGGAAGCATGTCGAGAGGTGCGTGGACGACCCCTCGATGCTGATTGTTACCTATGAAGGTGACCACAACCACAACCGAGTTCTAGCCCAACCAGCCTGATCTTTCAGGCTATCAATTCAAGATCGCTTCTATAGTCACCCTCCTTCCCCTAGAGGAAGGGAGGACAACAACAACAAGAAGAAGAGCCTAGTGTCTGTACATATTTTCTGCTTCTAACAGTTGGGGGGAGAGAGAGTCAGTTAGCTAGCTCTTTCAGAAGAATCAAATCAATGGTGACAGAAGGAAACTTATCGCGTCGATGGAGAAAGGGAGAGTGCCTCATCAAATCAAATACTCTAGTTTTGTAGTTTCGATTTCTCTTTTGTGTTCATCATCCTGTGTTAGGGCCCCATCCTGTGGTTGCAGTGTAGTAACATTGAGCTGCAGTTGTATGTCTGAGAAATGGTGTTAGGGGGATTGGATCGGAGAAGAAGGCATAAGGCAGGAAAGTGCCTGTAAAAGTGGCTACACCTAGGAACAGAATGTACAATCGTCTTCAGCCCAGTGGTACAATCTTGT

>SbWRKY7

TTTTTTCTCTCTTCGTTCACTCTCTCTGCGTCCGGTCTTCTTCGTCCCCTTCTTCGCCTTCACACCTTCTGCCATTTGCGATTTACCATCTCGTGGGGCGACGACGACCGCCGCCCTCCCCTCCGGCCACCGGTCTCGGTCGCGGCTCCCCTGGATTCGCGGCACCGCGTCCGTCTCCGGATCGTCTCGGAGGTGAGCTCGTGGCTTGGGTGGTGATTCTTTTTTTTGTGGGTCGTCGTGCGGCGGTTTGGTGATTGCCTGCCTTTGCCTGGTCGAGTTCCTTCCTGGCGTGCGAATCTTATGGATTATTATTATTAGATAGAGGTGTTCCGTCCGTGCAGTGGGGTTCTTGTGTCCTTCTTGATGCGTCTGCACGCAAGAAATCGCTCGCTGCCCCCCCGGATCGTTGCTTGCCGCGGCCGTCGACGTCGGTGGTCGGTCTCGGTGTCCGCGCCCGCCGCTTCTGGGAGGGATTTCTTGCGGAGACGGCTCCCCCCCGTGATCCGCTCACGAGCTGCTTTCTTGGCTCTATAAGCTTTTCCCGATGCCTGTTGCGTGGTGTACTGGTGTTACGATAAAAGAAGAGCTGAGCTTCTAAGCTTTTCTTAGGATCTGAGTGAATTTTTTTTCCCTGCTGTCCAGATCTGCTGCTGAGGGAGCTGCATATAATCTGAGGTGCTCCTGAAGTGTTCAGAAGAGGTTCCTGTTGTATTTATTTTTGTTGGTGCGAGTGTAATGGAGGAAGTGGAGGAGGCCAACAGGGAAGCCGTGGAGAGCTGCCACAGGGTGCTCGCCTTGCTCTCGCAGCCGCATGACCCCGCACAGGTCAGGAGCATAGCTCTGGGCACGGACGAAGCATGCGCCAAGTTTAGGAAGGTGGTCTCCCTGCTCAGCAATGGAGGAGTGGGAGTAGGAGAAGCCGGACCATCAGGCGCAAGTGGAAGCGGAAGCCATCCGAGAGCTAAGCTTGTTAGCAGAAGACAGAATCCAGGGTTCTTAACTCAGAAAGGCTTCCTGGATAGCAACACCCCGGTTGTGGTGTTGAACAGCGCCCATCCTTCTCCTGCCTCTGCGCAGGTGTATCCTAGAACTGCTGGAGCTCTGGATGCGCAGGGCGTGCATCCCCTCGGAGGACCGCCTAAGCTGGTCCAGCCTTTATCTGCGCATTTTCAGTTTGGCAATGTGTCGTCACGGTATCAGTTCCAGAATCAGCAGCAGCAGCAGCAGAAGTTGCAGGCTGAGATGTTCAAGAGAAGCAACAGTGGGGTTAACTTGAAGTTTGAGAGCACCAGTGGCACGGGGACAATGTCATCGGCGAGGTCCTTCTTGTCGTCTTTGAGCATGGATGGTAGTGTGGCTAGCCTGGATGGCAAGTCATCGTCGTTCCATTTGATCGGTGGGCCTGCAATGAGCGATCCGGTGAACGTGCAGCAGGCCCCAAGGAGGCGATGCACGGGTCGTGGGGAGGATGGGACTGGCAAGTGCGCTGTGACAGGGAGGTGCCATTGTTCAAAGAGAAGGTACATCTGCTACCCCTGTGCTTACTATTACTATTTTAAGTTCAAGATTTTTAATATATCATCACTGTTCTTTTTTATTTCAGTAGGAAGTTGCGGGTAAAGAGGTCGATTAAGGTTCCCGCCATTAGTAATAAGATTGCTGATATACCTCCGGATGAATACTCGTGGAGGAAGTATGGG

CAGAAGCCAATTAAGGGTTCCCCTCATCCTAGGTATGGACTGAAGTATGCCTAGTCTAATCTTTCTTGCATGCATGTGGTTTATTAAATAGTGCTTTAGAATAGCTGTGATGATTCCACAATTAATGCCAACAAAACTCATCAGTTTTGTCAGCAAAGACGTCATCATTTGCAAAGTGATAGTGTGGTACAAGTTCCATGACGTACTCAAGTGCCTTCTTGATAAATAAATGAACTGATGATAATCAGATGCTATCTAACATTTGTTTTCTGTACTCAAGGTGCTTCGTTATTGAAGAGGCTATGACATTAAATTATGGTTCCATATATGATGTTGCTCTTTCTTTTGTGTGCACTTTATGGAAATGGATCAATCATGTTGCTTAAAGTGGGCACATTTTTTAAATTGAGCCAAGATTCATTATCTCCGAACCCCCCTCACCATGTCAGTTGCCCACCTTGTCTGGAGCTTTAATATCTTGATAGCAAGTTACATATTGGTAGCAAGCACACAAATATAGTTGTTCCTATGAATGATACACATCTTTGTCTCCATTTAGCTAACACTAGATATGGGAACACTTTGTGTGTTTCTATTCTTATCATAGGCTCATAGCTACATGTTGCTATCTTGATGTTGGCATAAGACCAACTCATTGCTTTCTTGAATAAAATGCAGGGGTTACTACAAATGCAGTAGTGTGAGGGGCTGCCCAGCTAGGAAGCATGTTGAACGGTGTGTAGATGATTCATCAATGCTCATTGTGACATATGAGGGCGAGCACAACCACACCAGAATGCCAACTCAGTCAGCACAGGTTTAGAGAACTCATCATCACTGCTGCACCTCCTTCAGGAAGTGCCACCAACACGGCCTTTGTTGGCAATGGACGGACTGCGCCGGCCGGTCAGGTTAACAATGATTGTGTGGAGACATTTTTAGTAGCTGTCGTTGAGATGTTTGGCCCTTCTTACAGCCACATGATTTGTAGCGAGAATGGAAGTGGGTGAGCACTAGATTTTCTTCTCCCTTATGGAGGTTATCACAATGATGATGACGAGGAAGAACACGCCATTAGATAGGACAGCTGAGCTTTTGCTCAGGATTAGTGTGAAGAGGTGGAAGGGAAGATAAAAGTTGGCGGGAATGAGACAGTTGTGTAAATTTAGTAAAAAAAAAGGGAGTAAAACTAGAGCTGCTCTTCTTGGGCTATCCCGATTGGCTGATTCCCTCACTCACTCACCTGTATCAAGAATAGTGTCAACTGAAAACAAGTGCACAGAGGCTGTCTTTTGCTGTTATAGCAATTCATTCATATTCATTCATGGTCCAATAATCAGCGGTGCTACCTTCTCTATCACGGTTCTAAAATTTATCTTTGTTGTTTAGCATATGAATTCCTTTATCAGCATATGTAGTGTTAGAGAGTGCCACAGTGGTAGACAATTATTGGTTGAATTACCTTGTAATAGTATCCGAAACATCACTTTCATGAAAAGTTGCGTGCAAATAGTATAAAGT

>SbWRKY74

CTCCGCCGCGCGCTTCCCCCCATTCCTCTCTCTTCCTGCTCCTGCTCCTGCTCCTGCTCTCATCCATCCTCCCTCCTCGATCCCCTCCACGCCGGCCCTGCCGGACCGCGCCGCCGCCGCCGGCCGTTCTCCCCCGCGCCGTGCCTGTGGTTCTGGAGCGGGCGGATTCGTTCGGTGCGTTCTTGGCCCGCCGCCGCCTGTCCGTGTGCCGGAGCCTCGTGCTGCGCGCGGCGTGTTGCCTCCTCCTCCTCCTCCAGTCCAGGTGCGCAATCCGGTACCGTGCTCTTTCCATGAGCCGAGGATTGGAATAATCCACCCTTCCTGAATCCTGATGGGCCGTTTCTTTCGTTCCTGTGCAGTTTGCTTCCGAGCCCTTTTGAGATTCCCCGGAGATCTGCTTAATCCGTGCGGGCTCTCTGGTTCCGATGTGCAGACGCTGCTGCCCGTGAGCGCTGGCAGGAGCCTGAGGTACGTCCGAGGTGGAAGAAGAGGAGTTCGTGGCTGGACGAAAGAACAATCCTTTCTTTGTCCACTCGGCGGCCGCCGCTGCTAGCTTCTCCATTCCAGGTCGGTCGGCTTCCAGTCCAGCCAGCCATGGAGGAGGTGGAGGTGGCCAACAGGGCCGCGGTGGAGAGCTGCCACCGGGTGCTGGCCTTGCTCTCGCAGCAGCAGGACCCGGCCCTGCTCAAGAGCATAGCTTCAGAGACGGCCGAAGCCTGCGCCAAGTTCAGGAAAGTAGCCGCCCTCCTCGGCAGTGGCAGTGGCGGTGGCGGCGGCTGCGGCCATGCTAGAGGCAGGTTCTCCAGACGAGTCCGGCCTATGGGTCTCGTGAACCAGAAGAGTCCCTTGGGGAGCGGCAGTGGTGGCGGCGGCAGCCCGCTGGAGATGATGCCCAGCACCGCTGCTGCTGCTGCTGCGGTGGCGGCTCCGTCTCCATCGACTAGCTACGCACAAATGCGAGCTCGGCTTAACGGTGTGCCAGACTCACGAGGGCTGGATTTGGCCTGCTCCAGCAGCAAGAGTGGCGGCCCTCATCCGTTCGGAGCCCCCAAGCTGGTCCAGCCGCTTTCTGTGCAGTTCCAGATTGGGAATGTTGCGCATAGGTACCCGTTCCACCAGCAGCCCCCGTCGCGGCAGAAGCTGCAGGCCGAGATGTTCAAGAGGAGCAACAGTGGGATCAGCCTCAAGTTCGAGAGCCCCAGCCCCAGTGGCGGCGCTGCTGGCACGATGTCGTCTGCGAGATCATTCATGTCGTCCTTGAGCATGGATGGGAGCATGGCTAGCTTGGATGGGAAGCGGCCATTCCATTTGGTTGGCACCCCGGTGGCGAGCGACCCAGCTGATGCCCACCGCGCACCCAAACGGCGGTGCACGGGTAGAGGGGAGGATGGAAGAGGCAAGTGTGCCACTACCGGCAGGTGCCATTGCTCAAAGAGAAGGTAAGTTAGTGTTCTCAGGGCCATTCTTATGCTTGTTGTTATGTCTTGTTGGGTGCAATATTAACTCCCTTGAATCGTGCTTGCTCTGTTTGACAGGAAACTGCGGATTAAGAGATCAATTAAAGTGCCAGCCATTAGCAACAAAATCGCTGATATACCTCCTGATGAGTACTCGTGGCGCAAGTACGGGCAAAAGCCAATTAAGGGTTCCCCCCACCCGAGGTATAATATGAACTGAACAAGAAATATTCAACTAACATG

AAAATGTGTATCAAATCCTTTTCAGTCATAATGATTGATGCATTATTCCCCTTGTTGCACCATGAGAGGTGGCATATCTGCATCAGGCTCTCAGGGTGCCCCTTTATCTGTAGAATAATTGTTGGTTGATCCTCCTCTCCACTTTACCTTTTCCTTCCTCCCAAGTGCTTCTGCAACTAAGGCATGTCGATAACATAGTAGCTAGCGCAATTAAATATTTAGTTAGTGGATAATCTCAGGGTTCAACTCTTTTTGTGCCCATTTACTGAGAAAGCAATGATCTATCATGGGGGACCTAAAGTAGGTAAATTATTATTGCTACATGAATTTGAATGCCATGTTAGGCTATCTACATCCTAATCTTGATTGCGCTCATTCCCCTACCCCATATCAACCTTTAATGTCTGCCCGAGATGATAGGAAAATGTAGCTATACCATGAAGGCAAGCACATGGGGCTGATTGTTCCCCCCAATTTCACATATTCTCATATTCTCTTTGACTTCAATCCAATGCAGGGGTTACTACAAATGCAGCAGCGTCAGGGGCTGCCCGGCAAGGAAGCATGTTGAGCGATGCGTAGATGACCCAGCGATGCTAATCGTGACATACGAAGGCGAGCACAACCATAACCAGTTGCCAGCACAGGCTGCCCAGACCTAGAGGATCAGTAAACTGTCGCTCCATTCAGGAAGCGCCTGGGCCATGCCTTTGCTGAAGGATGACCTAGCTGGTCATGTTAGGATCCGAGGCCTTTGAATTTCGAATGCAGCAGCGATGAACATGGATCCATTTGAGAATTTGATGAGCTGCTTGGCCCCTCTTCTTGAGCTACACGCTGTTACAGATGCTATTGTGGGGACTGCTGCCAAGTTGTTCATCTTTCTTGTGAGCTGTGGCATGTAGTATGATTGAAAGATGGTGCAGGAAGGAAAATGCGGCGTCTGATAGCGACCGGTGAGCTTTGTCGCTGAGAGTCTGAAGGGTTGAAAGCAAAGATTGACAAAAGGAAGTGAACAACCGCGCATTGTTGTAAAGGGTGGATTAGGATGGCAGGGTTGGCTTTTAGCGTGCACAAGTTCGCTTGCCATGACTTGCCTGTGTATGCTGCTTTTTTGCTTTTCTGAAACCTCTTTTTGTTGACAAAAAAAAAAAGGAGAGAGGGAGGAGGTCCGTACTGACCGCTGCAAGCTAGGACCATTTGGATTTTCCAGGTGTAAGCATTCCAGAAGGAAAGGTGCAAAGCTGTTGTTGGTGTGATTTGCCACCCCCAGTACTATTGCTCTTGTGTAAAGCATTACGTGTTAATGATTTCCCCATCCATCTGTGCCACTCTGCAATCTTGAGAGGTCATCTAGAAGATGAATGAATGCTCT

>SbWRKY47

ATGATCACCATCGATGATCTGCTGAGGAGCTGTGGCGGCGACAGCGGCAGTATTCCAGTTCCTAGCAGCGACGACGGGCGGCAGATGCTTGCGATGGGCGACCACCACCAGCTGACGGTGTCCAGGATCCGCACGGCTGTGTCCATGCTCAACCGCCGCACGGGCCACGCGCGCTTCCGCCGCGGCCCGGTCGTCGCGGAGCAGCATGCATCGTCGGACCACCAGCTGCACCCGCCGGCGGAGAGCGCGCCCCGGTGTCGTGGCGCTGGACTTCGTCAATAAGGCTTGCGAGGCGAGGTTCAGCGCGTCGGCCTCGGGGACCAGCTCGTCGCTGCCGTCGAGCCTCACAGTCACAGCCGGCGAAGGGAGCGTCTCCAACGGCCGCGCTCAGCCTCAGGGTCAGTACCCCTTCCAGCCTGTGAGCGGCGGCGGCGGCAGCGACGGCCACTCTGCCAGGAAGCCGCTGCCGCTGGCGGTATCCATGCAGCAGCAGCAGCATGCCTCCCCCGATCACTCCGCCCCTGCAGGCACCGCGCTTAAAAACGGCAAGTGCCACGACCGCGCGCGCTCCGAGAACGACGCCGGCGGCAAGACGCACGGCCACCGCTGCCACTGCTCTAAGAAACGGTACATATACATGCTCATCAAACGCCCGCTGCCATTGGTACCAATGATCGGCTCCATAATTTGGTGAGTCGTAATACGTAGTACTATTTCTGACGAGTTGTGAAACGACGACGGATGGCATGCAGGAAATCGCGCGTGAAGCGGACGGTCCGCGTGCCGGCGATCAGCTCCCGGAACGCGGACATCCCGGCGGATGACTACTCGTGGCGCAAGTACGGCCAGAAGCCCATCAAGGGATCGCCGTATCCCCGGTAAGAAACGAGCTTGTAAACCGTCTGATCAATCCTTGATCCTTGTTCCTCCCAACCCAGCGATCGAGAGGCTGCGACTGACCATGATATGTGCGACTGACCATGATATGTGTACAGCGGCTACTACAAGTGCAGCACGGTGCGCGGGTGCCCGGCGCGGAAGCACGTGGAGCGAGACCCCGGCGAGCCGGCGATGCTTATCGTCACCTACGAGGGCGACCACCGCCACGACGACCAGCAGCAGGAACGGTCGGCCGGCGGCGCGCAGACGGATCACACGACGACGTCCAGTTGA

>SbWRKY62

ACCTCCCAACCCAATCTCCTCAGACTCAAGAGAGCCCCAAGCCAAGATCTGCGCGGGCAAGTCACCGGCGAAGCACCGGCTCCCATGGCCGTGGACCTGATGGGCTGCTACGCCCCGCGCCGCGCCGACGACCAGCTCGCCATCCAGGAGGCGGCGGCGGAAAGTCTCCGCAGCCTGGAGCTCCTGGTGTCGTCCCTGTCCACCCAGGCCGGCGCGCCGCACAGGGCCGCTCATCACCTGCAGCAGCAGCAGCCGTTCGGCGAGATCGCCGACCAGGCCGTCTCCAAGTTCCGCAAGGTCATCTCCATCCTGGACCGCACCGGCCACGCCCGCTTCCGCCGCGGGCCCGTCGAGTCCCCGCCGCGGGCGGCCGCGGCGCCTCCGGTCCCCGCTCCCGCTCCGGCTCTCTCCCTGGCTCCGTTGGCTCACGTGGCGCCCGTCAGCGCGGCGCAGCCGGCGCCGGCTTCCCAGCCGCCGCAGAGCCTGACGCTGGACTTCACGAAGCCTAACCTGACCATGTCGGGCGCCACGTCCGTCACCTCCACGTCGTTCTTCTCCTCCGTCACGGCCGGCGAGGGCAGCGTCTCCAAGGGCCGGAGCCTGATGTCCTCCGGCAAGCCGCCGCTGTCCGGCCACAAGCGGAAGCCCTGCGCCGGCGCGCACTCCGAGGCCACCACCAACGGCGGCCGCTGCCACTGCTCCAAGAGAAGGTAACGCCGGCCGATGTCCAAATTAAAGGCTTCCTCAAAAACACCACTTGCTGTTTGTAGCTTGTCTTCTAACCCACGGTACTTTTTTTATTTTTTGGCAGGAAAAACCGCGTGAAGAGGACCATCAGAGTGCCGGCGATCAGCTCGAAGATCGCCGACATCCCGCCGGACGAGTACTCGTGGAGGAAGTACGGCCAGAAGCCCATCAAGGGCTCCCCTTACCCACGGTAAATTTCCTCCACCGAGAGCCGGAATCGCTCGTCTCGTCTCCTCCGTCGTCGCGCAAGGAATCTGACTGTTTTCTGGTCATGTGGTCCACAGGGGCTACTACAAGTGCAGCACTGTGCGGGGATGCCCGGCGAGGAAGCACGTGGAGCGCGCCACCGACGACCCGGCCATGCTGGTGGTGACCTACGAGGGCGAGCACCGCCACACGCCCGGAGCGGCCGGGCCCAGCCCCCTGGCGACCGCGTCTCCGGTGGCCGCCGCTGTCTCCGCCGGCAACGGCCATGTCTAGTCGTTCCAAAGCTAGGATTAGCTTCTCTCTTCTTTTTTGAGCTGCTTGCCCCCCGATCTGATGTCCGTGTAAAAAAAGGAACAAGGTTGTAGAAAGAGGGAGTGCACACCTTCGATGCCGCAAAAACTCTCAAACTTGATGAATCGCATATCTCCGATTGGTGCTCGCTCACGCTTCCTGTTTTTTTGTTTTGTTGTTGTTCTGAGAAAATACAATAGCCCTCAAAATCGGACGAAAATCGCGACCGCTGACCTGCAGATCTGAACATCTGACGCAGCTGTAGACTGTAGCTGCGACTGTAAAATAATTGTAT

>SbWRKY64

ATTGACTTTGCACAGCCCCGGCGGGCAGCTCGTCGCCTAGCATTTGGTTGGTCCTCTCCTCTCGCTCAGCTCCTCCCCTCCCCACTTCCATTTCCACCTATAAAACCCACCCCCCACCACCACCTGTTCCCACCTCACAATCCCCACAACCCAAGAAAGCAGCAGCAGCAGCAGCACCACCACCACCACCACCGCGCGGATCTGACCTCCAACTCCAAGCAGCAGCAGAGCAGAGCAGGGGCAAGCCATGGCGGTGGACCTGATGTCGTCCTGCGGCGGCCGGGCCGGGGCGTACGAGCAGCTGGCGTTCCAGGAGGCGGCCGCGGCGGGGCTGCGCAGCCTGGAGCTGCTGGCTTCGTCGCTGTCGTCCCCGTGCGGAGCGGGGCAGCGGGCCGAGTCGCCGCCGCTCGGGCAGATCGCGGACCAGGCCGTGTCCCGCTTCCGCCGCGTCATCAACCTGCTGGACCGCACGGGGCACGCCCGCTTCCGCCGCGCGCCCGTCGCCGCGGTGGAGACGGAGACGACGCTCCAGGCCGCGGTGGAGGAGCCGCAGCCGCCTCAGAAGAAGGCCGCCCTGACGCTGGACTTCACCAAGCCGGTCCCGGTCCCGGCGGCGGCGGCGACCAAGCCGGCAGCGCCGGCGCCGGCGCCGGCCGTGTCCGGTACCTCGACCTCGTTCCTGTCGTCCGTGACCGCGGGCGGCGGCGGCGAAGGGAGCGTGTCCAAGGGGTGCAGTCTGGCCGTGTCCTCCGGCAAGCCTCCCCTCCCGAAGCGCAAGCTCCCGTGCCCCGCCTCCGCTCCCCAGCAGGCGCAGGCGCACCAGCACCAGCACCAGCACCAGCACCTCGCGGAGTCGTCCGCCGGGCGTTGCCACTGCTCGAAGAAGAAGCGGAGCCGTCAGGGCCTGTCCCGGCGCACGGTGCGCGTGCCCGCGGCAGCCGCGGCCGCAGGAGCGCCGGGGTCCCACGTGCCGGCGTCGTCGGACATCCCCGCCGACGACTACTCGTGGCGCAAGTACGGGCAGAAGCCCATCAAGGGGTCCCCTTACCCGCGCGGCTACTACCGCTGCAGCAGCGCCAAGGGCTGCCCCGCGCGGAAGCACGTGGAGCGCGCCGCCGACGACCCGGCCATGCTCGTCGTCACCTACGAGGGCGACCACCGCCACGACGCCGCCGCCGCCGCCGTCCGCGCCCGCGCGGCCTGAAGATGATCGTTTGCAGGCGGTGGCAGCTCTAGTCAATCATCAGTGCTCGTAGTAGTAGTTGTAGTCCGCTCTTGTTTGATTTGTTCTTTGGATTAATTAAATTAGTGGTAGATCTCCTCAAGCTTTGCTTGCTTCTGTAAAGAAATTAAAGAGCTTCTAAACTTGAATTCGCCATGAATTTTCTCCAAGCTTTTTTACAGATATACAGTATATATATATATATATATTATATAACGCGTGAATGAATTTCTCGGA
