# Supplementary material for: Genome-wide Identification of WRKY transcription factor family members in sorghum (Sorghum bicolor (L.) moench)
Source: PLoS One. 2020 Aug 17;15(8):e0236651. doi: 10.1371/journal.pone.0236651 (PMC7430707; doi:10.1371/journal.pone.0236651)
Supplement: S4 File — (DOCX) [file pone.0236651.s004.docx]

>SbWRKY13

ATGGCGCCGGTGCAGAGTGCTCTGGCGGCGACGTGGACGCCGTCCTGTCGGACATCACTGATTCACTGTGTCAAGCGATCACTTCTCTGCGGATCCGCACCGGTGGGCAGCCAGCGACCGCCGGCATCGCCGCCAACAGGAGCCCAGCCGGTGCCGGCGGCAGGAGATCAGCAGCGCCAAGGAGAACTTCTCAACGGGCCAGTGCTACAGAATGGAGTTCATGATTCGTACACATGGAGGAAATACGGGCAGAAGGAAATTCTGGGCGCCAGATTTCCAAGGAGTTACTACAAATGTGGCCGCCGGCCGGGCTGCCCCGCGAAGAAGCACGTGCAGCAATGCGACGCGGATCCGTCCAAGCTGGAGGTCACCTACTTGGAGGCACACACGTGCGATGATCCACCACCGTCGTCGTCCCATGCTGTTCCAGATCCGACGGCCGGCTCCGACGCTCTGCTCGTACCACCAGTCCCGACCGTTCCGTTTCCATCAGCTCAGTGCTACGGCGGCGGCCGACCGTCGCCGCCGCCGCTGCCGCCGTACCAGGTGCCGTACGCCGCGACGACGCTCGGCTCCAACGTCCTGACGCTGACGGCCACCGGTGTTCTTCTGCCTAGTGCAAGCTACGACCCTGTTCCGGATGTCACGGACTGCACGCCGTCGTTGGAGCAGGAGCAAGACCATGATCTGCTTCACATACCTTCGCCGGCTTGTTCACAGTCAGAGCTGCTGCCGATGGAGGCTGCCAAGCTTTCACCGCACGCGCACGGGCTGCCTCTGTCGTTGGAGCACACGCTGGATTGCGACTTTGCTGTACCCGAGCTTTAA

>SbWRKY6

ATGGATTCAAGCTCACAGCCCGGCGCAATAGGCAGCGGAGGAGGAGGAGAGAGAAACCAAAGGGAGGAGGACGAGGCGGCGGCGGCGGCGGCGGCAGAGGCCGGCTACGGCAGGCAGCTGGTGATGCCCGAGGACGGGTACGAGTGGAAGAAGTACGGCCAGAAGTTCATCAAGAACATCCAGAAAATCAGGAGCTACTTCCGGTGTCGGCACAAGCTGTGCGGCGCCAAGAAGAAGGTGGAGTGGCACCCGCGGGACCCCAGCGGCGACCTCCGCATCGTCTACGAGGGCGCGCACCAGCACGGCGCCCCGGCGGCGGCGGCTCCTCCCGGTCCCGGCGGCCAGCATCACGGCGGCGGCGCCTCCGACTTCAACAGATACGAGCTGGGCGCGCAGTACTTCGGCGGGGCCGGCCGGTCGCATTGA

>SbWRKY43

ATGGGAGATGGGGACTATGGGCTGCACCCTGAACCCGGCGCCGCCGACGTGGCCGTCTGGCCCGGCGAGCTCGACGAGCAGCTCATAACCGAGCTCCTCAGCGACGACAGCCTCCTCCTGGGCACCCTGCCGCCGCCGCAGCAGGTCCCCGCCGGCGACGACCCGGAGGAGCAGCACTGCTCGCGTGACACAGGCGGCGCGTCATCTGCTCCCGCCGCGCCGTGCATCAGCGGCGGCGGCACCGCTGCCGAGCACCGGGAGCTGCTTCCGCAGCCGGAGGCGGTGAGCAGGGCCCTGTGCTCGGTGTACACCGGCCCGACGATCCGGGACATCGAGAAAGCGCTGTCGACAACCAGGCCGTACCCTTGGAGTTGGAGCAGCAGCCGCTACAGCCCAACAATGCATCTTGGCAGGTTGGGAGCGCTGAGTCGAGCGCCGGAGAAGTACACTACGAAGGTGAGGAGCTGCGGCGGCAAGACGCCGAGCGACGGGTACAAGTGGAGGAAGTACGGGCAGAAGTCCATCAAGAACAACCCCCATCCCAGGAGCTACTACAAGTGCACGAGCTCCCGGTGCGGCGCGAAGAAGCACGTGGAGAAATCCACGGAAGACCCGGAGATGCTGATGGTCACCTACGAGGGTCCGCACCTGCACGGCCCGCAGCCGCTCTTCCCGCGCCGGCAGTGGCTGTCGATTGACCTGTCCGGCGCTGCGGCGGCGGCGGCTGCGGCCTCGAAGACGAAGCAGCAGCAGGCCAGGGTCTCCTCCTCCTCCTCCCCGGCCGCGTCAGCCGCGCTCGCAACAAGCGACGACGGGGGCGGCGGCTGGCCACCGAGCCAGCAAACGACGACGCGCGGGCGCGACACCGAGGCAGCGCGAGGAGGGCCCACGGCTGCTGCAGGCGAGACGGCGACGCCCGGGCCGCCTCAGATCGGACGCGCGGGTGACGCTGTGTCGACGCAGCCGCGCCTGGTGCTGACCGCGGACTCGTGCGACGACGGCTCGGCCGCCTCCGTGCCGCCGCCGTGGGCTTCTGCGGCGTTTCCTCACTGTGACTCGCCGCCTATGATGACCTGGTAA

>SbWRKY78

ATGGACGACGTGCTGCGGCAGATCGACGAGGGGTTCCGCCTCGCCAGGGATCTGATGGAGGAGCTCCCCGCGGCCCAGAACGAGCGGACCTACCTCGCCGACCGCTGTCACGGCATTGTCCAAGCCTACGTCGCGGCCATCCGCATGCTTCACCCGCACGGCGGCACGGAAGACACGGCATCGTCCCCACCGCCGCTTCGGCCGCCTCATCCTCCTTCTCCTCACTTCGGCGGCGATGGTAGTGGAAGCGGCCAGCACGACCACGAGATCCCTCAGCTGGACCTCCTCCGCCCGTTCCTCGGCGGTGCACCTTCTCCTTCGGCGCCGTCGTCGTTCCCGCACAACCTCGGCCGCCTGCTGGCAGAGTCATCGTTCATCAACACCACGCCCGTGGTTGACGCGTTCGGCGCTGGCACATCGTCAGGCGGCCCGGTGAGACGGCAGGCTTCGTCGTCACGGTCGTCACCGCCGGTCCAGCTGCGGCAGCAGCACAGACGAAGGAGAGAGAATGGTGAGAGGATGACAATTATGGTTCCGGTCCAGCGGACGGGCAACACAGATCTGCCACCGGACGACGGCTACACGTGGCGCAAGTACGGACAGAAGGATATACTGGGATCAAGATTTCCAAGGAGCTACTACAGGTGCACTCACAAGAACTACTACGGGTGCGAGGCGAAGAAGAAGGTGCAGCGCCTAGACGATGACCCGTTCACGTACGAGGTCACATACTGTGGCAACCACACCTGCCTCACCTCGACGACTCCGCTCCTCACAATCCCAGCAGGCCCCGCTACCGTGGCCTCAACAGCTGCCAACATGCTAAACAACTCCCCGACTGATTCAGCAACAGCCCTCGCCGCCGGTCATCAGGACCTCTTCATGCCGGCCGCTGAGCATCCTGCACAGGCGCTGTCCACGGCCATCCAGCTCGGCATCAGCTGGATGCCATCAACCCTCGTTGGCTCCAGTGCCCCCGAGGGAAGCAGCTCTCAGGTGAACGTGCCCGCCTCAGGAAGGGACACCGCCGAATACCCGGTTATGGACCTCGCCGATGCCATGTTCAACTCTGGCAGCCGCGGAGGGAGCAGCATGGACGCCATCTTCCTTGCTCGTCATGATCGACGTGATACCTAG

>SbWRKY11

ATGTCTCCGGCGCCGACTTCGCATCACTCGCAGATCAATTCGAGGAAGGAGAAGCGGATGAGGAAGGTGGACACCTTCGCGCCGCACAACGACGGCCACCAATGGAGGAAGTACGGCGAGAAGAAGATCAACAACACCAACTTCCCCAGGTATTACTACAGATGCACGTACAAGGACAACATGAACTGCCCGGCCACGAAGCAGGTGCAGCAGAAGGACCACAGCGACCCGCCATTGTACGCGGTCACCTACTACAACGAGCACTCGTGCAACAGCGCCTTCCTCCCGCTCTCCCCCTCCGAGTTCCAGCTGCAGACCTCGTCCGGGAAGGCCGTCTCCATCTGCTTCGACTCCTCGTCCGGGGCGGCGGCGCCGCAAGAACCGCCGGCCACGGCAGCGGCCACCAATGCCAGCGGCGGCGGCGGCTCGCCGTCTTCCAGCGCGGCGGCGGTGGCGGCGGCGCGGAGAGGCACGCCGCCGGAGATCAGTAACCCGCCCGTGCTGCGGCGGTCCGAGACGTACCCGTGGGGCGCAGGCGCCGCCGGTGTCGTGGAGCAGAAGCCGGCGTCCTGCAGCACCGAGTGCCACGACGCCTTCTCGGGTGCCGCCGGCGCCGTGCCGGAGGAGGTGGTAGATGCAGGCAGATTTGGGTCTATCAGGTTCTTCCATTTTTTGTAA

>SbWRKY56

ATGGACATCAAGAGCAGCCTCATCATGCTGCCGCCCTGCAGCCATGGCTGGGAGATGATGGAGACCATGAGGAGACAGCAGGAGCTCGTGATGCAGCTCCGAGCGTTCGTCCTCCCGCTGCTCCCCGGCGTCATCGTCGATGGTACCTCGGCCGCCGAGATTGCTGTCCAGCTCTTCGACGACGTGATAGGATGCAACATAGGCGTGGTGTCTACGCTTGAAGGGTGCCTCGTGAGTACCGGAGCCAGAGGCGGATCATCGGGAGAGCCCGTAGACAACAAGTCGTTGGTGAGGAAGAATAGCTGCCATGTTACTGAAGGCGAGACGACGGATGAGCAAGCGAGGCATAGAAGCGTTGTTGGTCAAAAGAGAAGGAGGAAGAACGACAAGCGATCACGATCCCTTGTGACGCATGTTCCACATTATGATGGCCATCAATGGAGAAAATATGGGCAGAAGAACATCAATGGGAGGCAGCATCCTAGGAGCTACTATAGATGCACCTACAGAGAACAGAACTGCTTTGCAACGAAGACTATCCAGCAACAAGAACAAATTGACAGTATCCGTCGTAGTGCCACGCCTGGTGAGGAAATTGCAAAGTACACAGTCGTGTACTACGGTGAACACACTTGCAAGGACCATAGCATCAGCATAGTCCAGCTTCCTCAACTTGTCAGCTGTATGGATCTTCAGAACATGGAAATTGCCCAAACAAGTTCAGATGTTCAAGATCCTGAGGCAGACTTGGACTTGCCAGCTTTACTAGAGGTGTTTGATAACTCTGTCATTGATTGGGAGGATATTTGGAAGATATGA

>SbWRKY53

ATGGCTCTGGACTCGGTTCCTCCTTACCCCTGCGACCTGGGCTCCAGCAGGGCCGCCGCCGCCGCCAGAACCCAGCAGAGGATCAGGAAAGATGAGCGCACCTGGACCTCGGACACGTACGCGCCGTACGACGACGGGCACCAGTGGAGGAAGTACGGCGAGAAGAAGCTCTCCAACTCTCACTTCCCGAGGTTCTATTACAGATGCACCTACAAGAACGACATGAAGTGCCCCGCGACGAAGCAAGTCCAGCAGAAGGACACAAGTGACCCCCCATTGTTCTCTGTCACTTACTTCAACCATCACACCTGCAGCACCAGCTCAAGTGCCATAGGAAGCGCTAGAGACATCACGTCGCAGTCATCTTCTAAGAAGGCGGTATCAATCTGCTTCAGCCCACACACCGCCTCCGAACAGCCCTCATTCCTGACATCCCCGGCCATGCCACAGTCAACGATCATGCACCCTTACAGCGCCAACCAACAGTCTGACAGGAGCGCCTACGCATACCAGCAACTCCAGTGGACAGGCAGTGTGCCATCTCACGCAAGTAACGGTCCTGCTAAGATGGAAGTTGATGACTCTGCGCAGCCAAGCCCTTCATCCAGCAGCACCAGTGCTCTGTCGAGGACTCTGCTACCGATCGGTCAGTCCAGATGCATCGAGTACTTCCATTTCTTGTGA

>SbWRKY71

ATGGCTCTCGAGTCTGTTCCTACATATCTCAGCGATTTGGGATCCCACCAGGCTGCCAGAGCCCAGCAGCAAAGAATCAGGAAAGATGAGCGCATCTGGACCTCAGACACATATGCTCCCTACGACGACGGGCACCAGTGGAGGAAGTATGGCGAGAAGAAGCTCTCCAACTCCAACTTCCCAAGGTTCTATTACAGATGCACCTACAAGAACGACATGAAGTGCCCGGCTACAAAGCAAGTCCAACAGAAGGACACAAGCGACCCACCGTTGTTCTCTGTCACTTACTTCAACCATCACACCTGCAGCAGCATCTCAAATCCCATAGGAAGCACGAGAGACGTTGCCGCACAATCGGCCTCGAGCAAAGCGGTGTCAATCTGCTTCAGCCCGCATTATTCTTTCAGAGATGAGCCACAGTCACCGATTGCACATTCTTTCAGAGGCAACCAGCAGCCAGCTGAGAGGAGCGCCTATGCAACAAGCCAGTTTCAGTGGACCGCCGCATCGTCTCCGTCTCCTACCAGTAATGACAGTCCGGTTAAGATGGAGGTTGACACTTTTTCAGGAGCAAGCGCTTCTTCATCCAGCTCCAGCAGCATGGGTTCTCTCCCGAGGACGAGGACGTTGCTACCGATCGGTCAGTCCAGATGCATCGAGTACTTCCATTTCTTGTGA

>SbWRKY38

ATGAAGAGGGAGCAGTCCTTTGAGTTTGGGGATCCCAGTGCCCAAGATGCTATGGGATCTGCTGCCTCGGAGTCGTCTTACAGCCCTCCCGGAGCTGTGTTTGGGCTCTCCCCACCGGAGTCGGCCTCGCCGCGCAGCGGCCGGCATAACAGAAGGAGGGATAGACCTTCATGGGTCAGACTCACGTACACACCTTATTTTGATGGTCACTTGTGGCGAAAATATGGGCAAAAGAAAATCAAGGATGCTGAGTACCCTAGGCTATACTTCAGATGTTCTTACCGTGAGGACAGGCAATGCCTGGCCTCTAAGCTGCTGCAGCAGAAGAACGGCGACGACCCACCACTGTACGAGGTGACCTACACGTACGAGCACACGTGCGGCGCACCGCCCGTCTCGTTCCCGGATATCGTGGCCGAGCCGCCGCCGGCCGCCAGGGAAGGTCTGGTGCTCAGGTTCGACTCCCCCGGCGGCCACGGCGGCCACGCACGGATGCAGCAGAACGGACACTGCCAGCAGTCCACGTCTCGGAGTCCGTTCATGATGCTCAGCTTTGGTTCCAGGAGCCAGACGCACGATCAACATCCTGCCGTCTTCCGCTCTGACTTGGAGGCCGGATCATCGTCGTTTCCCACCGAGGCGCCGCCGGCGCCGCCACCACCGGCAAACGGCGACGGTGGGGACATGCTCTCGACATTGAACTCCTTCGCATATGATTTCGACAATCAAATGCACTTCGGCGATCACACGTATTTACCTCATAATAATAGTAATTACGATTATGATGATTACTGA

>SbWRKY40

ATGCAGACGCAGTCCCGCCTCGGCGGCAGCGGCAGCAGCAGCTCGGCGTCGGAGGACGAGCACGAGGCGGTGATCCGCGAGCTGACGCGGGGCCACGAGCTGACGGCGCAGCTGCGGGCGGAGGCCCTGCGCGCGCTGCGCGGGCAGGGCCAGGCCGAGGCCACCGCCGCATTCATCCTGCAGGAGGTGTCCCGCGCCTTCACCGTCTGCCTCTCCATCATGAGCTCGCCCGCCCGCGCCCCGCCGTCGACGTCCCAACCTCCGCCGCCGACGATGGAAATAATGGCGCCCGCCCTGCTGGCGCCGCCGCGCCGGAGCCGGGACGACAGCATGCCAAGAGAGCAAAGGAGGACATCCTCGCCGCACTGCGATGGGTACCAGTGGAGGAAGTACGGCCAGAAGAGGATCACCAAGACGCAGTTCCCGAGGTGCTACTTCAAGTGCAGCTTCCACCGCGAGCGCAACTGCCGGGCCACCAAGCAGGTGCAGCAGTGCAGCAACGACGACCCGCCCCAGTACGTCGTCATCTACTTCAACGAGCACACGTGCGACGACACGGCGGCCTGGGATCCTACTCCCACGGTGCCGCTGGACGACCTGTCATCCGGGCTGCTGGTGGCACGCCAGGCCGGCTCGCTGCTGCTGGACGAACGCGGCGTCCAGGAGGAGCACGAGCGCCGGCTGCTCGTCTCGTCGCTCGCCTGCGTGCTGGGGGCCCAGCAGCAGCAGTCTCCTGCCGGCAGCGGAACCACCGCCGCCGTTAACGTGGGGCATGAGCAGGACCAGGAGCCGCCGCCGCCGCGGGCCCGCACGCGCGACGACGCGCCTGCGCCTGCGCCTGCGCCTGCGCCTGCGGGCGTTGACGACGACGCGCCCGGTGAGATGCCGCGCAGCATCATTGACGTGGACGTGGCGGGGCTGGATGTCATGGACTACTATGTGACGGACGCGCTGTGTTTCCGCGATTCCTACGACCTACCTAGCGACGGTTTTTCGTTTTGA

>SbWRKY39

ATGGCATTTGATCGAGACGCCAAGCTGTTCGACGTGCTCGCCAACGGTTACCATCTCAACACCCAGCTCCAGGCATTGCTCGTCGGCCGTCCCCTGAATAGCATCGGCCAGCAGGAGGCCATGGCGTTCAGCCAAGAGCTCTCGCGAGTGTTCAAGTTATCCATGTCCATGCTGAACTGTAACACAGTGACAAGATTGAGGACGGCGCCGGAGATAAGGGCCGGCGATAGCTCCGGCGTCATCATTCAGGCGGTGAAGGATAAGCGTGCAAGGAGTGATAATGGGGAAGTGGTTACTCCCGTCAAGAAGAGTAGAGAAGATGGGGTTACTAGAAAGGAGATTACGGCCTCGCCATACAAGGATGGTTACGAGTGGCGAAAATATGGGCAAAAGAACATCCAGAACTGCAATTATGTGAGGTACTACTTCAGGTGCAGCCGCGACCGGCGTTGCGAAGCGAAGAAGAAGGTGCAGCAGCAGGACGACGGCAGCGGCCGGGGCCAGCCGCTGTCGCCTCCCATGTTCGAGGTGACCTACGTGAACGAGCACACGTGCCATCTGCTCCGCGCCATTGCCAACGACGGCGACGCTGCTAGGATGGCGGCGTCGCCCCGGACCACGAACCGGTGGTCCCGCGTCCTCGGGGTCGTGGACACCGCGAGAGACGACGACCACGGCGGCGGCGTCCTGTTCAACGATCTGTCGTCGTCGTTTCCCCGCATCGGCGGCGGCGGCGGCGACGACGCCCAAGAGAACGAGACGATCGTCTCGTGCCTCGCGACTGTCATCAGCGGAGGAGCAGCGCCGTCGCCGCCCCCGTGGCCGCCAGCAGCGGCCGAAGCGGGCGCGAGCGATCATCCTGCTGCAGCGTCGTCGTACGGCGTGCCGCCGCCGATGCAGGCCTCCGGACACTCGGCGAGCGTGGCGGAGGATGGTGGTGGGACGACGACGACGACGACGACGATGATGATCGACGACATGGACACGGACTTTTGCTGGGATCCCTCGTCGTTTTGTGCGGTGGGGGAGGGAGATCAGCTGATGATGGACCACCGCGACATGCACGTGGATGTCGCCCGGCTCGCGGACACGGTGTGGCCGCGGCACACCTCCGCGGGTGCGTCTTGGCGTTGA

>SbWRKY81

ATGACGCTGTCTCCACCGCAGCCGCCGCCCTCCTCCAGCCCAAGGCACGCAGCGATCCAGGAGCTCAGGAGAGGTACCCAGCTGGCGGAGCTGCTCAGGCAGCAGGTAGAGCTCATCCCGGAGCCTAACCGCCGGCAAGCTGCAGTGGTCAACGTGGGCGAAATATCCATGGCTATGGAGTCGTCGCTCTCCATTCTCCAGTCTGAGATGGAGCACCCCTTCGTCTCCGAGGTCATGGCTGCGCCCACTGCCTACTCTGACGGAGGCAGCACCAGCAGGGAAAGGAATGGTCCCGTGGCCCGTACAAGAAGGGTGAGGCACCGGCGAGGCAGAGATGGAGCTGAACTCCCGATCAAGGAGATACTGACTGAGGCACCAGAAAACGATCATTTCCACTGGAGGAAATATGGTGAAAAGAATATCCTCTATGCTGAATATCCAAGGTTATACTACAAGTGCGGTTACAGCGATGACCACAAGTGCCCGGCAAAGAAATACGTGCAGCAGCAAAGCAACACCTACCCTCCACTTTTCTTGGTCACCCTGATCAACGAGCATACCTGCGACACCTTGTTCCGGGATGAGCCCAGCTCAAGCAGCAGTGGTTCGCAGGTTCTCGACTTCACAAAGGCATCGCTTTCTCCTGAAGAAGACAGCAGCATGCCTGTGTCTATGCACAGATATTCGTTTTCGTATGATGGGTACTAG

>SbWRKY14

ATGTCAAGTATGGAGGCTCCTAGTCCCACCACCAGTCCTTTGGACGGCAGCATTTTGAAGCTTCCGGGGAAGCTGGACAGGCTACTGCATCAGCGTCATCGTTACGGATGCATCCTGCCCAAGGGCGTGGAGGATGAGATACCTCTCATCAAGGGTGATCTCGAAGAAATCATGGCCATGCTCTCAAACCTTGATGACTATGAGGCTATGATGGTTAGGTGCTGGAGGAAGGAGGTGCGCGAGCTGTCTTATGACATGGAGGACTACTTCATCGACCAGTACGAGCACACCTCAGCTGCTGAGTCGTCCTTGTCCTTGATGATGATGACTGGATCTGTTTCTCGGCGACAGCGTCGTAACAAGAGCAAGACCACTGTCTCTAGGCTTGGCGATAAGATCTTGATAGCCAATAACAAGATGAGAGAATCCAGAGTGCGAGCACAAGAGCTATTTCAACGGTACATGAGCATGTACAATCGCGACGCCGCCGCCGTTCCTGTCTCTGGTTCAAGTTCAACTAGCAGATGTCATCATTCTAATTCCACACCCCGTGGTGGAGAAGAGAAAAACCTGGATGACCACCATGCTATGAAGGAGGCTCTGGAGCTGTCGTACGACATGGAGAACTTCATCGAGCATTATGGGCTACTCACTCCCGAGTCCTTATTGGCTGCTGCTGGATCCATTTCTGTCCGTAGCCGCCGTCAGGTTACTCATCATCAGCGGCCTAGTCGTAAGAGCAAAACAATTATACTCTCTAGGCTTGGTGAAAAGCTGAGGCAGCGCCTCTGCATGGCCAACATGATCAGAGAATTCAGCTTGCGTGCACAAGAGGCACTTGGACGGTACAACACGTATAAACTTGATGCCATTTCTGCCGGCCCTGCAGCTTCCTCCATTAGATCATGTACTACTACTGATGATGGAGTTTATTCTGGCTCGTCCTGGAAGTGGAATTCCACGACGTCGACGTGCGAGGATGATGTTATTGTTGGTATCAGTGCTGCTATGGAAAATCTCCAAGAGTTGCTCCTGACGATGCATGATGAAGGGCACCAGCAGAATCTTAAGGTGGTATCCATAGTTGGGTCTGGAGGAATCGGCAAGACAACAGTTGCCATGGAACTCTACCGCAAGCTTGGACATCAGTTCGATTGTCGCGCATTTGTGCGGACTTCCCAAGAACCTGACATGAGGAGGATTTTCGTCAGCATGCTCTCTCAAATTGGCCCACACCAGCCACCTGATAATTGGACGATACACAGCTTAATTTCCACCATCAGAGCACATCTGCGAGATAAGAGGTACTTGATTGTAGTGGAGGATTTATGTGCTACATCCACATGGGACATTGTTAAACGTGCTTTACCAGATACCAATTGTTGCAGCAGAATACTAACAACAACAGAAATTGAGGATCTAGCTCTTCAATCTTGTGACCATGACCCCAAGTATGTTTATAAGATGAAACCACTAGGTGAAGATGACTCAAGAACATTGTTTTTTAGTTCAATTTTTGGCCCTCAACATGAATGTCATTCAGAACTAAGGGAAATTTCTCATGCAATAATAAGTAAATGTGGTGGTTTGCCACTGGCTATTGTGACTGTCGCTGGTGTTTTATCAAGTAAGCCAGGCTTAGCAGATCAATGGGATTACGTCAATAAATCCATAGGGTACAGTTTATCGATAAATCCTAC

CAGTGAAGGGATGAAACAAGTCCTTGACCTTAGTTTCAACATTCTTCCCCAGCATTTGAAAGCATGTATATTATACACTGGTCTGTATGAAGAGGACATCATAATTTGGAAGGATGATTTAGTCAACCAGTGGATAGCTGAAGGTTTTATCGAGGCAACAGAAGGGCAAGATAAGAAAGAAATTGCCAGATCTTTTTTTGATAGGCTTATCAGTAGAAAACTGATCCTCCCTGTATGTATAAATAAAAATGGTGAGGTTTTGTCCTGCGTGGTTCACCGTATGGTGCTAAATCTTGTTATCAGATACAAGTCAGTAGAAGAGAATTTTGTCACTGCAATACATCATTCTCAAACAATCACCACACTTTCTGACAAGGTGCGTCGACTGTCTCTTCAATTTGGTAATGCAGAAGATGTGATACTCCCAATAAATATGAGACTGTCACAAGTCCGAACACTTGTCTTTTGGGGGGTCTTCAAGTGTTCGCCTACCATTGTGCTGTTTCATCTCCTTCAAGTTCTGATTCTCCATTTTTGGGGCGATAAGGATAACATCAATTTTGACCTCACTAGAATTTCAGAACTTTTCCGGCTGAGATATTTGAAGGTCACATCTAATGTTACCTTAGAACTAGGCAACAAGATTCGAGGCCTGCAATCTTTGGAAACACTTACAATAGACGCAAGAGTTAATACAGTTCCATCAGAAATTGTTTACTTGCCAAGCTTGCTGCATTTTAGTGTTCTCCCAGAGACAGACCTGCCTAATGGGATTGGCCACATGACATCGCTTCACACACTTGGATATTTTGATCTCAGTAGTAATTCAATAGAGAATGTACAGAGCCTTAGCATGCTGACCAATCTCGTAGATCTTAAGCTAACCTGTTCTACAGGTCAGCCAGAAAATATGTATAACAAAATGCAGTTCTTGCTGACCTCAATTCTTGGCAGACTCAGCAACCTCAAGTCTCTTACTCTTGTCCCAAGAGCTTCCACTAATAATGCAAAATCTACAGATGAGGCTGGTGCTACAGGCATGGCCATTTCTGGTGGCTTCAGCAGTTTGTCCTCTGCCCCAGGCCTTCTTCAGAGTCTTGAGGTTTCGCCACAGATTTGCATATTCTACTGGATCCCCAAGTGGATCGGGCAACTCCACAAGCTCCGAATCTTAAAGATTGGGCTTACAAAAATAGACAGGGATGATGTTGATGTTATCAGAGGATTGACTGCTCTTGCTGTTCTCTCCCTGTATAGCCAGACCAAGCCTGCAGCAAGGATAGTCGTTGGGAAGACTGGATTTCCAGTAATAAAATACTTCAAGTTCAAGTGCTGTGACCCTTTGCTGAAATTCGAGGAGGGTTCTATGCCTAATCTCTGTAAGCTGAAGCTTGTTTTCAATGCCAATCATGCCCATCAACATATTACAATACCTGTTGGCATCAGGTACTTGTCAAACTTGAAAGAGTTATCTGCAAAAATTGGTGGTGCTGGTTCTGATGAGTCCCACAGAAGGGCTATAGAATTGGCTTTCAGAGATGCAGTCAGGGTGCATGCTAGATGTGAGAGAGTCAGCATACAATGTGTACAGCAGATCATT

GGTGGCAAGGATGATCAGTATAGCCTAGGGCGAGTGGAAGATTATGGGGATGAGGAAGACTCAGATGAACTTGTTGAAATGATGCCGGAGCATTATGGAGAAGCAGTGGATACAGATGCTGACAACAGGTCATTGTTATCCGAATTGCATATTTTACTCCTCTTGAGGAACAAGCTCGACAACCAATGTGATGATATCACATACAGTATCCCCATCGTTGATGTCTACTCATACGACCCATGGAATCTTGAAAGTAGTGGCAGCACGCATAGGGGACTGCTGTTATTTTTCCGCCCACTACCGGAGGCAAGGGGCGTCGTTCCGGTCAAAACACCCTCAGGTTACTGGAAAGTCACAGGTCTGCCTGGGTACATCTACTCGGATGAAAGACTTGCAGTTGGGATGAAAAGAACGATGGAGTTCTACCATGACCACTTGACATCAGGTACCAAAACCAAGTGGAAGATAAAAGAGTTTACAGCATTTCAGCATGCCACAGCTGGTGAAATCTGCACGCCCATGATGCCAAGAAGCGAGATGAGCCTGTGTCAACTGTACACGGAATTGGCAGCGAATCCCGATTGCAGTCCAGCAGGTGTCCAGTATGATGAAAAATTGTCCGTCAAACAAAATAAAATTCCAGCAGAGGAACCATCAGTAGAAAGTGACATTGTTCAAACCCGCAAACATGCTGATAAGAGGAAACGTAGGGTGGACGAGACCACAAAAACTGTGGTAACTATTGCCAGCCCTGATGTGAACGACGGCTACACCTGGAGGAAGTACGGTTCGAAACAAATCCTGGGCTCAAACTACCCAAGAGACTACTACAAGTGCACGCAACGAAGAGGCTGCCCGGCGAGGAAGCACATGCAGCGCCGTGACGGCGAGCCAATACTCTACGACGTGTGTTACTTTGGAGAGCACAGCTGCGATCTTCAGCAGGGGCACTCCAGTGAGCAGGGATCAGAAACAAATACAATATCAGGAGGATGGGAAGCAGCAGGGCTACCATTATCGGTACCACACGCACATGGAATAAGTTCTGTGGAGGCCATGATAAGCAGCGGCAGCCTCACCGCGCGCCCCAATCCAGGTGTCTCCTCCTTGCGGCCGTCGTCCATGGTCACGAGCCAAGTAGTGATGAGCAACCCTGACGACGATGGCTACTCCTGGACAAAGTATGGCCAGAAGAACATCCTCGGAGCGAAACACCCAATAAGTTACTACCGTTGTGCGCATTGGATCGCGCAGGGCTGCACCGCAACTAAACGATTGCATCGCAAGGAGGATGCCGACACGTTGGGCTTCGATGCCATCTACTACGGGCAACACACTTGTGATCAGATCGCACACTCCACTGACAATATCAGTAGCCCCTTGGGCTGTACCACCACTGGATCTACTTCCAAGCTGGGCACCGATAAAATTCATCCTGAACAAGGTGGACTTTGA

>SBWRKY75

ATGGGGATGGAGGATCCGACGGCGACCGCTTCTTTGGATGGCACCATTTCGAAGCTTCCAGGGAAGCTCGACAGGCTACTTCGTCATGGTACTCGCAGAAAAAAAAGGCTAATGCACCATGTCTGCACCCTGCCCAAGGGCGTGGTGGATGAGGTACCCCTCATCAAGGCCGATCTTGAGAAAATCTTAGCAATTTCCTCAGATCTGGAGGACGACCAGGCTATGACGGCCAGGTGCTGGAGGAAGGAGGTACGCGAGCTGTCCTACGATATGGAGGACTTCGTCGACCAATATGAGCACGCCCATGCCGTGTCCTGTTCCAGATCCATGATTCGTGGCCGCAAGATTGCTACTCAGCAGCGGCGTAAGAGCAAGATTAGTCTCCCTTGGCTCCGGGAAAAGCTGAGGCGGCGGCTGTGGATGGCCAACAAGACCAGAGAATTCAGCGCGCGCACGCAAGAGGCGCTCCAACGGCACAGCTTGTATAACCTCGACGCTGTTGCTGGCGCCTCTGCTTCTAGTAGGCGCACTTGTGCATATTCTGACCCTGCTTGGAATTCCACACCTTGTGGGGAAGAGGACGCCTATGTTGGTATCAATGATGCTATGGAAGAGTTGCTGATGATGATGCATGATGATCACGGGCACCAGAAGCTTAAGGTTGTGTCCATTGTTGGGTTCGGAGGGATTGGCAAGACAACACTTGCCACCGAGCTTTACCACAAGCTTGGGCATCAGTTCGAATGCCGGGCATTTGTTCGGACTTCCCACAAGCCTGATATGAGGAGGATTTTCATCAGCATGCTCTCCCAAGTTCGCCCACACCAGCCACCTGATAATTGGACGGTCCATAGCTTAATTTCCACCATCAGGACACATCTGCAGGATAAGAGGTACTTGATTATAGTTGAGGATGTATGCACTGCGTCAACATGGGACATAGTTAAATACGCTTTACCAGATAGCAATTGCCGCAGCACAATATTAATAACAACAGAAATTGAGGATCTAGCTCTGCAATCTTGTGACCATGACCCCAAGTATGTTTATAAGATGAAACCTCTTGGTGAAGATAACTCGAGAAAATTATTTTTTGGCTTGGTTTTCGGCCAGCATGAATGTCCTTTGGAACTCAGGGAAATTTCTTGCAATATTATAAGTAAATGTGGTGGCTTGCCATTGGCCATTGTGACTGTTGCCAGCATTTTATCAAGTCAGCCAGGCGTACAAGGTCAATGGGATTTTGTCAATAAATCCATAGGTGAGAGTTTGTTGACAAATCCTACCTGGGAAGGGATGAAACAAGTCCTCGACCTTAGCTACAACAATCTTCCCCAGCAATTGAAAGCGTGCATCTTATACACTAGTTTGTATGAAGAGGATATCATAATTTGGAAGGATGATTTAGTTAACCAGTGGATAGCTGAAGGTTTTATCCAGACAACTGGAGGGCAAGACAAGAAACAAATTGGAAGGTCCTTTTTTGATAGGCTTATCAGTGGAAAACTGATACTCCCTGTAGATATAAATAGAAATGGTGAGGTTTTGTCCTGCGTGGTTCACCGCATGGTACTAAATCTTGTTATTAGAGACAAGTCAATGGAGGAGAACTTCGTCACTGCAATACATCACTCTCAAGCAGACACCATGCTTGCTGACAAGGTTCGTCGGCTGTCTCTTCAGTTTGGTAATGCAGAAGA

TGCAATACCCCCATCAAATATGAGACTGTCACACGTTCGAACACTAGCATTTTCAGGGGTCTTCAAGTGTTTGCCTTCCATTGAGCAATTTCGTCTTCTTCAAGTTCTAATTCTTCATTTCTGGGGCGATAAGGATATCATCAGTTTTGACATCACTAGAATTTCAGAACTTTTCCGGCTGAGATATTTGAAGGTCACATCCAATGTCACCTTAGAACTAGGTACCCAGATGCGATGCCTGCAATCTTTGGAAACACTTGCAATAGATGCAAGAGTAAGTGCAGTTCCATCAGATATTGTTCAGTTGCCAGGCTTACTGCATCTCCGTCTTCCTGCTGAGACAAACCTGCCAAATGGGATTGGGCACATGACATCACTTCGCACACTTGGATATTTTGATCTGAGTAGTAATTCGTTGGAGAATGTACAGAGCCTTAGCATGCTGACCAATCTCGGAGATCTCCAGCTCACCTGTTCTACAAAAGAGCCAGAAAGTCTGAATATGAAAACGCCATTCCTGCTGATGAACATGATTCTTGAGAAACTCAGCAACCTCAAGTCTCTAGCTCTGGTACCAACAAGAACTTTCAGTTATTATACAAAATCTATCGATGATGACGCTGGTGCTACCTGCATCACCATTTCTGATGGCTTCAGCACTTTGTCCTCTGCCCCAGCCCTTCTTCAGGGTTTTGAGGTTTCACCACGAATTTGCATATTCTTCTGTACGCCCAAGTGGATTGGACAACTCCACAAGCTCAGCATCTTAAAGTTTGGAGTTAGGAAAATAGACAGAGATGGTGTCGATGTCCTCAGAGGATTGCCTGCTCTTGCTGTACTCTCCCTGTATGTCCACACCAAACCTGCAGCAAGAATAGTCATTGGGAAGATCGGATTTTCAGTTATCAAGTACTTTAAGCTCAAATGCTGTGATCCTTGCTTGGAATTCGAGGAGGGTGCTATGCCTAATCTCCGTAGGCTGAAGCTTGCGTTCAATGCCTGCAATGCTGATCGACCGAGTACAATGCCTGTCGGAATCAAGTACTTGTCAGAGCTGAAAGAGGTATCTGCAAAAATCTGCGGTGCTGAGGAATCACACACAAGGACTGCCCAATTGGCATTTAGAGATGTCATCAGGGTGCATGCTGGGTGTCAGAGCGTCAATGTACAATGTGTAAAGCAGATCATTAGCAGTGAGGATGATCAGAGTAGTCAGAGTAGCATGAAAACAGCGAAATATCCACGAATTCAATATTTACCTTCCCCTGCCCAGGCCCCAGGCCGGAATCAGAACCGGCACCGGCGCTGGCCCGACAGCATCCCAAGAGAGCAAACTACTGTGCGGCAAATGTTGACACCGACCCCATACGAGGACGGTTACCAGTGGAGGAAGTCCGGGCAGAGGATGATCAACAATGAACGTTTCCCGAGGTGCTACTACAGCTGCGCCTACCGCCGCGACCGCAAATGCCGGGCCATCAAGCTGGTGCAGCAGTACAACGACGGCGCGCCGCCTCTGTTCGCCGTCAAGTACTGCAACCACCACACATGCGTCCCGGAGGCAAACCCGGCACGCCGGAGCCGGGGCCGGAACAAGGAGCTGCCGCTTGGCTTCGGAACCTCTGTGGCTGGAGCTGAGCAAGGAACTCGACTGCAAATTAGAAACAGCTACAGAGGAGACTCGTCGTCGTCCGAGTCCA

GCCGCGCTCCTCAATCGTCAGATATGATAGTGTTCTATGACTCGACGTCAGAGGAGACTATGTACGTATTAGGGCCGGGGTCATGGTACTAA

>SbWRKY37

ATGGCTAAGAGGGATGACTACATGGACAGCTCTTGCGGCTGCTCTAATGGAACCCCTAAAAGGCTGGTGCAGGATTGTAGCAGTTATGCACAGGCACATGCTAAGAAGAAGGTTCGCATTAGCACAAGAACTGAGTACACATACGCACCGTATCATGATGGCTATCAGTGGAGGAAATACGGACAAAAGATGATCCGGGGCAATGCATACCCAAGGTGCTACTATAGGTGCACATACCATCAGGATCATGGCTGCCCAGCAACCAAGCATGTGGAGCAAACCAATTCCCAGGATCCACCATTGTTCCGGGTAATCTACACAAATGAGCACACATGTTGCAGCACCCATGTCTCAGATTACATGGCTTCATCTATACACATCCAACAGATCGCCGATGCTTCTTTGAGAAAGGTAGAGGTGGAAATACCTAGCCTGACCCACTGTTTTGATGGCCACGGATTGATAAAAGAAGAGAATGATGCCATCATCTCCTCGCTCACGGCCATCAGTGATTATGATGTTGCAACATCAGATGGTGGGCACGCAGCCGTTCAAGAGGACACACCTGCTCGGATGTCCAGAAGCAGCAATGAGGCTAGCCCTTCGATTTCACCTGTACTGTTGCCGGCATCCGATAACCTGAAAACAGATTTCATTGAGCAACTGGAGCCTCAATGGTTCGAGCCTTTGGATTTGGGTTGGTTCATATAA

>SbWRKY51

ATGGCTTGCGTGGCGGACCGTGAGGACGCCGTGAGGGAGGTGACGCAGGTGTACGAGCTCATCAAGCTCCAGCAGCCTCTCCTCCTCCTCCACTCGCCGCAGCACCCGCCGCCGCCGCCGTCGACGACCTGCCAGCTGGCGCAGAGCCTCCTCGCCAAGGCGCTGCGAGCCCTCAACGTCGCTCTCTCCGTCATGAAGCAGCAGCCTGCTCCAGTGACACCAATAAGCGTCATCAAAGCTGAGCCTCATCAGCTCTCGCCGCCCTGCAGCCTGGCGTCTGCTGAGTCCCAAGCCGCCATAGTACTCAGCACGGCAACAAGAGGCGCCAAAAGAAGAAGATCAACGGAAGGGAAGAAGAAGAATACCTCGTCGTCCTGGGCGACGGTAACCGCCGTGCCCTACGACGACGGCTACGAGTGGAGGAAGTACGGCGAGAAGAAGATCAACGGGACGCTCTTCACCAGGAGCTACTTCCGGTGCACCTACAAGGACGACGCCGGCTGCCTCGCCACCAAGCACGTCCAGCAGATGGACAACAACAGCGATCCGCCCATGTTCCACGTCACCTACAACAACGACCACACCTGCAACACCAGTGCCAGAGCCAACACCGGCAGCAGCAGCAACCTCGCTGCATTGCTAGCAGGCTGCTGCAACATGAAGCAAGAACCAACTGGACATGCTGCTGCTGCGGCGGCGGCGACCATGGACATGAAGCAAGAAGTCCAAGAACCGCCGCTCCTGCTGCCTGCTCTGGTGGACCTCCAGCCTTCTGCCTGTTTTCATCATGAGCAAATTCCACGGTGCCAAGAACCGCTGTTTCCTGTAAGTATGGAGCAGCAGTTCGTTTGTGGTCCTTTTAGAGACGACGATAGTGAAATCCCGTCGGCTACTGGTTCGTGCATCTCCGGCGAGACCAGCTGGGATGGGTATTCTGGACACATGGCGGCGGCTGAAGATGACCCTCTCCTCGATCTCGAGCGTTTCCTCTTCATGGACTACTAG

>SbWRKY52

ATGGCTTGCGTGGGGGAGCGTGAGGCCGCGGTGAGGGAGGTGGCGCAGGTGTACGAGCTCATCAAGCTCCAGCAGCCTCTCCTCCTCCTCCACTCGCCGCAGCACCCGCCGCCGCCGTCGACGGCCAAGCTGGCGCAGAGCCTCCTCGCCAAGGCGCTGCGAGCCCTCAACGTCGCCCTCTCCGTCATGAAGCAGCAGCAGCCTGTTGTCGTCGTCAAAGCTGAGCCTCATCAGCTCTCGCCGCCCAGCCCGGCGTCTGCCAACTCCCAAGTCGCCATAGTACCCAGCACGGCAACAAGAGGCGCCAAAAGAAGAAGATCATCCGTAGCAATAATGGAAGGGAAGAAGAAGACCTCGTCGTCGTCCTGGGCGACGGTAACCGCCGTGCCCTACGACGACGGCTACGAGTGGAGGAAGTACGGCGAGAAGAAGATCAACGGGACGCTCTTCACCAGGAGCTACTTCCGGTGCACCTACAAGGACGACGCCGGCTGCCTCGCCACCAAGCACGTCCAGCAGAGGGACGACAACAGCGACCTGCCCATGTTCCATGTCACCTACAACAACGACCACACCTGCAACAGAGCCAAAGCTGCAGGCATAGCCAACAACGGCAGCAGCAGCAACAACCTCGCTGCATTGCTAGCAGGCTGCTGCAGCAATGGCAGTGGCAGCGGCAGCGGCAAGGGACTGACGACGATGACGACGACCAATGCTCGGCCAACCGAGCATGCTGCTGCTGCTGCTGCCATGAACATGATGAAGCAAGAACCACCGCTGCTGTTGCCTGCTCTGATCGACCTCCAGCAGCCTTCTGCCTGTTTTCCTAATGAACAAATTCCACAGTGCCAGAAAGAGCCGCTGTTTCCTACAAGTATGGAGCAGCAGTTCGTCTGTGGTGCTTTAAGAGACCATGACTCTCCTGTCGATGGCGACATCCCGTCGGCCACTGGTTCGTGCAACTCCGGCGAGACCAGCTGGTGGGATGGGTATTCTGGGGACATGGCAGCGCAGATGGCAGCTGAAGACGACCCTCTCCACGACCTCGACCGGTTCCTCCAGTGCGATAGCTTCATGGACTACTAG

>SbWRKY41

ATGACGCTGGACACCCCGGCCGCCGTGGTGCTGGAGCTGATGACCATGGGGCAGCAGTCCGCGGCGCACCTCAGGGACCTGCTCCGGGCCTCGTCCCCCGCGGCGTCGTCGCCGCACCAGGAGCTCGCCGCCGAGATCCTCCGCTGCTGCGGCCGCGTCATCGACGCGCTCAGGGCCACCACCAACGGGCGCAAGAGGAAGGCGGCGGCGGCCGAGTACCACCAGGACGCAGCAGCAACGGGAGGCGCCACCTGGTCTCCTCCTCCTCCTCCTCCGGGTCCGCCACTCAAGAGAAGGGCGCGCGGCGCGGAGGCGACCAGGGAGGTGACCAGCGGCACGACGGTGGACGGCTTCATCTGGAGGAAGTACGGGCAGAAGGACATCAACGGACACAAGCACCCGAGGCTCTACTACCGCTGCGCGCACAAGGACCAGGGATGCAACGCGACCCGGCGGGTGCAGCAGACGCAGGACCAGCCGGCGGCGTACGAGATCGCCTACTACGGGGACCACACGTGCAAGGGCGCGGCCACCGCGTGGCAGCAGCTGGGAGCGGCGCCCGCCGTCGTCGACTTCGGCTCCAACTCCTGGGGGTCCGCGGATGCCAACAACAACGGAGGCTCGCCGGCGGCCTCCATGTCGCAGGGAGGGTGGTCGCCGTCGGCGTCGTCCGAGGTCGGGTTCGACTTCGAGGCGCTGCACGAGTGGCACGACACGGCTGCTCCTGATCCGGTGATGGAGTTCCTGGACGGCTGCTTCGGATGGGAATCCGTCCTCCAAGACAGCTCCGACTTCGGCGGGCTCCTCCTCCATGACATCGCTACGTTTCAGTAG

>SbWRKY54

ATGATGAACATCCTTGAATCTTCCAATCTTGGCGGCTACAAAGAGGTCATCAATGAAGTTGAACACCAAAGGGCTCTCATGATGAACCTACATGACCTTGTACTACCAATACTTGATCCCTGCAGTGGGCAAGCAAAGCTCATACAACAACTCTTTGAAGAAGTATTCAGTAGCTCAGGTAAGATTATTTCTTCCCTAGAACTTGGTGATAACAGCGAGAAACAGGCCATTCTTATCAAGCATAAAGGAAAAGGAGGTAAGGATAACGTGGAGAATCACATATTGGAGGAGAACAACAAGGACCGTGGAAACAAGAGAAGGAAGAATGCAAATCACATAAGTTCAGTTGTGACACAAACACCATACTTTGATGGATGTCATTGGAGGAAGTATGGGCAGAAGTGGATCTCCAGAGCAAAGCATTCTAGGAGCTACTATAGATGTGCCTATAGTAAAGAGCAAGGGTGTCCTGCAACTAAAACAGTACAGCAGAAGGAAAATGATGGCAATGGAACGGTGAGGTTGTTCAATGTTAACTATTATGGCCAGCACATTTGCAACAGTGATGGCATAGTTCATCCACATGTTGTTGGGGCAACACAGGACAGCATGCCAATCGTCAGTCAAAACCAAAACAGTAGCTCAGTGTTTGTCAATACCGATGTCCATGGCGTTCAGGATGAAATCTTTGAAAGCTTATTCATGGTGCCCGACATGCCAGAATATTTGACAGAGTTCGTAGATGTTGAAATGGCAAGAGCATTTGAGATTACCCCTATGAACTCGCCAATGATCCCTGAAGACATATGGGCGTGA

>SbWRKY70

ATGAACATCCTTGAATCTTCCACTCATAGTGGGTGCCAAGTGGTGATCAACGAGATTGAACACCAAAGGGCTCTCATGACGGACCTACATGACCTTATCCTACCAACACTTGATCCCTGTAGTAGGCAGGATGCGCAGCAACTCTTTCAAGATATATTCAGTTCCTCAAGTAAGGTTATCTCCTTTCTCCAACTTGGTGATAACAGTAAGAAACCGGCCAATCTTATCAAATATAGAAGAAAAGGTGGTAAGAATAACGTGGAGAGTCACATGTTGGGGGACGAAGCTAAAGAAATTGGAAATAAGAGAAGGAAGAATGCACAACACACAGGTTCAGTTATGACACAAGCACCACACTTTGATGGATATCAATGGAGGAAGTATGGGCAAAAGTGGATCTCCAAAGCAAAGCATTCTAGGAGCTACTATAGATGTGCCAATAGTAAAGACCAAGGGTGTCTTGCAACCAAGACAGTGCAACAAAAGGAATCAGATGGCAGCACTGGAACAGTGAGGCTGTTCAATGTTGAGTATTACGGCCAGCACATTTGCAAGAAGGACGACATAATCCATCCATATGTTGTTGAGACAACAGATTATAGTGCACCAATTGCCAACTATAACCAAAGCAGTAGCAGCTCAATGTTTGTTCATAATGATGTCCTTGGAATTCACGATGAAAGCTTCGAAAACTTTTTCATGGTGCCAGGAATGCCAGAATATTTGACAGATTTCACAGATTTTGAAACGGCAGAGGCACTTGAGGTTACATCTATGATAATCTCCGAAGATATATGGGCGTAG

>SbWRKY55

ATGAAGCACCAACAGTATAATAGCAGATGTCTTGCTGAATCTTCAGCTTCTGATCACCGGTCGGCGGTGAAGGAGATCGCCAGGGGGCAGTCTCTGGTGACACAGCTGCGAGCAATCGTGCTCCCTGTGCTGCAGGCCGACGAGCGCTCTGAGCTTGTCGCCCAGATGTTCCAGAACATCCTGGATTGCTCCAGCAAGGCCATGGCCGAGCTGCAGATGCATCAGTCTCGAAGTACTCGACGACCTCATGATGATGATGATGTGCTGGTGGATGACAAGAAGAGAGTAAAGAAGATTTCCTCTGTCGACTGCAAGAATGAGGAGGGTGTTACTGCTGCTAAACCCCGTCATCAGCACAAGAGAAGGAGATTTGATGACTCTGTGTCACTTGAAACACCTGTGCCGCACTACGATGGCCGCCAATGGAGGAAGTATGGGCAGAAGCACATCAACAACACCAAACACTCAAGGAGCTACTACAGATGCACCTACAGACAGGAACAAGGCTGCAAAGCAACTAAGACGGTGCAGCAACAAGATGACAGCAGTGGCGCCGATCATACCCTGATGTACACGGTCGTTTACTATGGCCAGCATACTTGTAAGGACAATGATGGTGCTAATTCAAGCCCTGATGACTCTGAAATAAACACTAGGAGCAGCAGCGACAGCCACTCCAGCATATCAAGCACCTGTACAGATCCTTGTGACCATCAGAATCAGACATCTCTACATGAGGATAAGCCGTTCGTCAACAAATCTGAAGAGTTGGTCACAAAGGACATGTATGAGCCATTCGAGATGACTGTGTTTGCCCCCTTGGATTTGGATAGTTGGGAGTTGGATGCACTCCTGAGATTTGGACCCTGA

>SbWRKY69

ATGGCCGAGCTGCAGCGTCATCACCAATCTGATGATGGTGCTCGAGCTCGACCAGATGATGTGCTCGTTGATGACAAGAAGAGAGTGAAGAGGAGTATCTCTGATGACTGCATCAGCAAGGAGGAGGATGTTGTTAAGCCGCGTCATAAGCAGCTCAAGAGAGGGAGATTTGACGAGTCCATGTCACTTGAAACGCCCGTTCCACACTATGACGGACGGCAGTGGAGGAAATACGGGCAGAAGCACATCAACAAATCAAAACACCCAAGAAATTACTACAGATGCGCCTACAGGCAGGAACAGGGCTGTAAAGCAACAAAGACGGTGCAACAGCAAGATGACAGCACAGGCACTGATCATCCCGTGATGTTCACAGTCGTCTACCACGACCAGCATACTTGCAAGGACAACAATGGCATCAACTTAGGCATCGATGACTCTGAAACAAATAGCCAATCCAGCATATCGACAATCTCTACAGATCCCTATGGCCGTGAGACACCATCCCTAGATGGCAATAAGCTGCTCGACAAATCTGCAGACTTGATCACAAGGAACAGCATGTATGAGCCAGCCGACATGACTGTATTTGAACCTTTGGATTTGGACAGTTGGGCGTTGGATGCATTCTTAAGGTTTGGAGCCTGA

>SbWRKY10

ATGGCGTCGTCGGGCAGCGACGTGCCGGCGGCGAGGGCGGCCGTGGCGGTGAACGATCTGATCCAGGCGCGCGACGGCGCGGCGAGGCTCAGGGCCTTCCTGCTGCAGCTGGACGACCAGCGCGCGGCGTGGGCGCAGCTGCAGATCGACGGCGTCCTGACCAAGCTGTCGAGCGCGATGTCGGCGTTGGATGTAAGCGATGCCGCCGGGTCGGACGACGGTGCGAGGCCACGGCCGCAGTCGGGGAGCTCGTGCGGGAACAAGAGGAAACAAAGCTCCAGCAGAAGATCACAGCGTCCATCTGACAAAAAGATCACTGCTAACCTGGAAGATGGCCACGTATGGCGGAAATATGGGCAGAAAGAGATTCAAGACTCACCCTATCCAAGGAGCTACTACAGGTGCACGCACAAGACAGACCAGGGCTGCAGCGCCAGGAGGCAGGTCCAGCGCTGCGAGACGGACACGTCCAAGTACGTCGTTACCTACTACGGCGAGCACACGTGCCGGGACCCCTCGACGATCCCGCTCATCGACCACGCCGCCGGCGCCCTTGCCGAGCTCGACCGTGCCAACAACCTCATCAGCTTCGGCCCCAGCGGCACCAGCAACGACGCAAACGCAGCAGCCGCGGCCAGCAACGCCGGCGCCTCTTCGTCGCAGTACCTGCAGGCTATGGGCGGGAGCGCTGCTGCTGATCAGCTGTCCACGTCGTGGTGCACCAGCGACGACGTGTTCAGCTCGTCGGCCGGCTCGTTCATGCAGGTGGACCAGCTGATCGGCGCCGTCGTGGGCGGCTCCGCCGGGGTCGTGACGTCAGCGGCGGCGCCGGACCGTGGCGTCGTGCTTGGTGGCGTGGCGAGCGGCGGCAGAGGCACCGCCAGCTTCCCGACGTCTCCGAACAGCCTCGGCTTCGTGGTGGGATCGCTTGGGAGCATCGGCGGCGGCGGCGAGGACGACGACGATATGTTTAGACTGGATCCTTAG

>SbWRKY21

ATGGCGTCTTCCGCTGGCGGCGGCGGCCGGCGTTCACCGCCTGCTACGGCGGCGCCACGGATGGTGATGGACAGGCTGATGGAGGTGCACGAGGGCGCGACGAAGCTCCAGACCATGCTGCAGGAGTCGCCCACGCCTTCGATCGCGGCTGCGGCAGGGACCACCAGTGAGCTCAGACTGACGATTGACAGGATGCTGAGCAGCCTGTCGAGCGCCATGTCGGCTTGGAACACCACCGGCGCCGCCCAGGGACCGGGACAGGGGCGGAGGAGGAGGCGAGGCGAGGCGGCGGCCGGGTCCGGGCCGCAGCGGCGGAGCAGCACCAGGAGAAGATCGCACAGCCCCTTCGTGAAAATGGTCACTACCAGTAAGCTCGACGATGGCAAAGCATGGAGAAAATACGGTCAAAAACGTATTCATGAGTCCCCTAATCCGAGGAGCTACTACAGGTGCACGCACAGGCCAGAGCAAAGGTGCATGGCGACAAGGCAGGTCCAGGCCTCCGATGCCAACCCGTCGGAGTTCATCATCAGCTACTACGGCCAGCACACCTGCCAGGACCCCTCCACCATCCCGCTCGTCATCCCAGACACCGCTCCGCCGCCGGACTGCGCGAACCTCATCAGCTTCGGAGGATGCACCACCATCGCCGCCGGCGCATCGTCGTCATCCACCACCACCACTGCTGTTCCTCCCCAACAAGCCTTAAGCTTTGATCCGACGACGACTCCGATGTTGATGTTGTCTCGCTTCGGCTACAGCTCCTCTCTGCCGGCGGCGCAGGCGCAGCAGGACTACCGCTGCGGCAGCGAGGAGGTGCTCAGCAGCAGGAGCTCGCCGGCCGCGCAGCTCGCAACCATGGTGGTGGGATCAGCGGGGACGATGGCCTCGTCGTCCACCGTGGGGTCCGCGCCGGCGGAGTACTGGCCGGGAGGGACCAGCGGCATGGCGTGTGGCCCTGGCAGCTTCCCGTCGTCTCCTAGCAGCCTCGGATTCATGACCGGCTCGCCGTTTGGTTCCTTTGGCAACGCCGGGGATGACGACCTGTTTGGCTTCGATCCCTGA

>SbWRKY72

ATGGCAGCGGCGGCGCCGTACGCGCGGGTGATGGAGGACATGGTGAAGGGGCGGGAGTACGCGACGCAGCTGCAGGCGCTGCTCCGGGACTCGCCGGAAGCCGGCCGCCTCCTGGACCGGATCCTCCACGCCATGTCCCGCACCATCGACACGGCCAAGGCCGCCGCCGCCGAGGAGGAGGAGGCGTCCGAGGTGCAGAGCGACGTCACCTGCGCCGGCACTGCCGGCAGTAGCAAGCGGAAGGCCGCTGGTGGAGGGGACAAGAGGGCCTCCTGTCGGAAGAGAGGCCAGCAAGGATCATCGGTTGTGACGAAGAACATCAAGGATTTGGAGGATGGGCACTCATGGCGAAAGTACGGACAGAAGGAGATACAAAACTCAAAGTACCCAAAGGCCTACTTCCGGTGCACGCACAAGTACGACCAGCAGTGCGTGGCGCAGCGGCAGGTGCAGCGCCGCGACGACGACCCGGACACCTACACCGTCACCTACATCGGCATGCACACCTGCCGTGACCCGGCCACCGCCGTCGCGTCGCTCGTCGTGCACGCGGCCGGCGTCACCGGCGACGACCTCCACCACCACGCGGGGTCCCGCCTCATCAGCTTCGCCGCCGCCAACAACAACGCCAGCGCGGCGACGACCAGCACCACCACCACCGGGAACACCACCAACCAACAACTGGCGGTCCTGCAGCCCCTGAAGCTGGAGTGCGGCGGCGGCGGCGAGCAGGAGGAGGTGCTGAGCAGCCTCACCCCGGCGGGGAGCTCGGCGGCCGCGGAGGCGATGCGGAACGGGAACGCCGCGGCGGCGGCGGCGACGACGACAGGGCCGGAGCCGGACCAGGGGGACGTGACGTCTGGCCTGCAGCTGCAGCAGTTCTACGGCGCCGGCGATGACCTTGCGTACATGGCGCGCTTCAGCTACGATGACACGTTCGATCTCGAGGACATTGTTGTGTTCGGAGCTCCGGATTCGATCACGGACATTTATGCTGATGAGTAG

>SbWRKY68

ATGGCGCTACAAGTAGTAGGGAGGGAGGAGGAGCTCCTGGCGCAGCTCCGCGCGCTACTGTTCTTGCCTTCCCCGGCGGCTGCGGCGACACCAGCAGCTCCGGCGGCCGTTAAGGTGGAGTCCGCCGGCGGCGGATCGCTCATGGGCAGTGGCGGCGGTGGACGACGGCGGCGCAGACTGCAGGGGAGCAAGAGAGACCGGGACGACGACAGTAAAGCGAAGGACGAACAGAATCAGGAGGAGAGAGCAGCAGCTACAGAACCTCGCCATTATTCTCCTCCTCCCTGCAAAAGAAGGAAGAAGAAGCAGCAGAGCAAGAGCAAGTCTCTTGTGACATCAGTGCCCGATTTCGATGGGTACCAATGGAGGAAGTACGGGCAGAAGCAGATCGAAGGTGCCATGTACCCAAGAAGTTACTACAGGTGTACACGGAGCGCCGAGCAAGGCTGCGCGGCCAAACGGACGGTGCAGCGCAACGACGACGACGGTGGCGGCGCCGCCGCCGCCCCGGAGTACACGGTGGTGTACGTGTCGGAGCACACCTGCACGGCCAACGACTCGCTGGAGGCGCCGGTCATCCTCGAGACCACCACCACCGTCGTCGCCCCTTCTAATTCTGCTGCTACTGCTAACACCACAACCTACACCGACAGCATCGTCGTTCCAACTATGTCAGATCATGGCTCTTGTTCCACCATCACCATCACCACGGGGACTGAATCTCCGGCGATCTCCGGCGACGACATCACCTGCTGGAGCAGCACCAGCGGCGGCGCTAGCAGCAGCGATTATAATTACGCCGACGATGACTACTACTACGACTGCGGCGGGTTGTTCGGTGCCGCTGTCCATGGTGGTGGCTGGGCCACCGGACCGGCGGATGCATCGTCGTCGTCGTCGTTGTTGGAGATGGAGGACATGAACGGACCGATCCGGTCGCCGGTGCACGTTCCCGCGGTTGGTTGGACCATTATTGACCCGCTCTTGCTGCAGCTCGTTAATGAGCCTGCTGTCTGCCATTTCTAA

>SbWRKY15

ATGGCCGCCAGCGTCGTCGACGGCAATGGAGGCAGCGGAGGGCTGGTGGTGACGGAGCTGGGCCACGTCAAGGAGCTAGCGAGGCAGCTGGAGGCGCAGCTGGGCGGCTCCTCGCCCGACCTCTGCAAGCACCTCGCCTCGCAGATCTCCTCCATCGCCGAGCGCTCCATCAGCCTGCTCATCACCACCTCCTCCGGCCTCGCCGGCGCCCGGAAGCGCTCCGCCGTGCCCTTCGTCAAGGGCACCAAGAAAAGGAAGACCATGGACAAGAAGAGGCATGAGGTGAGGGTGAGCTCGGCGGCCGGCGACCACCCGGCCGACGACGGCCACAGCTGGAGGAAGTACGGCCAGAAGGACATCCTTGGAGCCAAGCACCCAAGGGGATACTACCGCTGCACGCACCGGCACTCTCAGGGATGCGCGGCGACGAAGCAAGTGCAGCGCACCGACGAGGACCCGACCTCCTTTGACGTCGTCTACCTCGGCGACCACACCTGCGTTCAGAGCCAGTGGGCGGCGGCGGCGGGCCAGGCTGCCGCGGACGCGCTGGCGCCGGAGTACAACGGCAAGCCGGGCACCAACCTGACGGTGAAGACCGAGGGGCCGACCGTGGAGCCAGCTGAGCAGCAGGTGCAGGGCTGGGACGCGCCCACGCCCTTCTGCTTCTCCTCCACGCCGGCGACGGCGACGGCGAGCTGGTGCCTCGTGCCGGAGCTCAGCCCCCCTTTCTCCGCGCCGTCCACGTCCAACAACTGGGGCGTCTCCCCGGCGACCTCGGACTCCAACCACGTCGTCTCTTTCCCGCCTTTCGAGGTCGCCGGCGACGACGTGCAGTTCGGCCGGTTCGAAGAAGTCATGTCAGCGATCGACAGAGCCGACGGCGACGGGTTCCTCGACGACCTCGACATTGACGTCTCAAGCTTCTTGGTGTGA

>SbWRKY16

ATGGCGACGACGGAGATGGAGATGGGCATGGGCGTCGTCGTCGGCGGCGGCAGTAACAGTAACAATGGAGGAGGCAGCAGCAGCAGCAGCGGGCTGGTGGTGACGGAGCTGAGCCACATCAAGGAGCTGGTGAGGCAGCTGGAGGTGCACCTCGGCGGCTCCCCGGACCTCTGCAAGCACCTGGCCTCGCAGATCTTCTCCCTCACCGAGCGCTCCATCGGCCTCATCACCTCATCCAACCTCGACGCCGGCGCCGCCCGCCGGAAGCGGTCCGCGAGCGACGCCGCCGGGCTCGCCTCCCCACTCTCCGCGACGCCCACCAGCGACGTCACGGACGGGCCGTTCAAGAACAACACCAAGAAGAGGAAGGTGATGGGACAGCGGAGGGAGAGGGTGAGCTCGGCCGGCGGCGAGAACCCCGTCGACGACGGCCATAGCTGGAGGAAGTATGGCCAGAAGGAGATTCTCGGAGCCAAGCACCCAAGGGGCTACTACCGCTGCACGCACCGCCACTCGCAGGGGTGCCCGGCGACGAAGCAGGTGCAGCGCACCGACGAGGACGCCACGCTGTACGACGTCATCTACCACGGCGAGCATACCTGCGTCCACAGGCCAGCGGTGCCGGCGGAGCACAACGCGGACGCGCACGCCCACCTGCAGACCCTGAGCGCCGGCCTCACGGTGAAGACCGAGGGCCTGCCCACGGCCGCCACGCCCTTGTACCTCTCCGCCTCCACGCCCCTGGCGCCGGCGTCGACGGCGTCGGAGAACTGGGGCGTGGTGTCGCCCGCGACCTCGGACTCCAACCACGTCGCCGCCTCCTACCTGCCGTTCGACGACGCCGAGTGGCGGGGCCACGCCGAGCTCCAGGAGGTGGTGTCCGCGCTCGTGGCCGCCAGCGCGCCGCCGCCGCCGCCGTTGCCGCCCGCCGTGGACAGCCTCGACGACCTCCTCTTCGACATCGACATCGCCAGCTACTTCGCGTGA

>SbWRKY66

ATGGCTGATGCCGTGGAGAGCGGCGGGCGGGCGCTCCTGGTCTCGGAGCTGGGCCGCGTGCAGGACCTGGTGCGCCAGCTCGAGCAGCAGCTGCGCGCGCCCGCGGACGCCGCCTCCGTCGACCTCTGCCGCCGCCTCGTCCACCAGATCGTCGCGCTCACCGACCACTCCATCGGCATGCTCCGCGCGTCCCCGGCGGACCTCGCTCCCTCCCCGCCGCTGTCCGCCACCGGGAGCCCGATCAGCGGCGGCGACGCCACCTCCGACCACCACCACCACCACCCCTTCCGCGCCGCCGGCGCCAGCCCCAAGAAGCGCAAGGCCACGGCGCGCTGGACCAGCCAGCAGGTGCGCGTCAGCGCCGCGGGCGGCGGCGCCGAGGGCCCCGCCGACGACGGCCACAGCTGGCGCAAGTACGGCCAGAAGGACATCCTGGGCGCCAAGCACCCGCGGGCCTACTACCGCTGCACGCACCGCAACTCGCAGAACTGCCCGGCCACCAAGCAGGTGCAGCGCGCCGACGACCACCCCGCGCTCTTCGACGTCGTCTACCACGGCGAGCACACCTGCAGGCCGCCCGCCGCAGCTGGCGGCTCCGGCGGCGCCAAGAGGGCGCAGCAGCAGCAGCACAACCCGCACGCGCAGGCAGCGCTGCAGGGCCTCGCCGCGCGCCTCACGGTGGCCACCACCACGGCCGCCGCCGCCGCCGCCGCCGCCGCGGCGCTCCCGCCAATGACGCCCGAGAGCTGCCCCGTGCGGGGCGCCTCGTCCCCGTGGTCGCCCGTCGGCTCCGACTCCAACGGCTGCCTGCAGCACCAGGGCGTCTCGCCGTGTCCCGTGCCCGGCTACGGGGACTGGGCCCCGGAGGGCGACCTCCAGGAGGTGGTGTCATCGGCGTTCGCCGCCGTCTCGTCGGCTGCGCCCCTGCCGGTGCTCGACGACGAATTCATGTCCCTCGAGTGCTTTGCATTCGACCACAACTTCGACATTGACACCGCAATGCCAAGCCTCTACTATCCATGA

>SbWRKY31

ATGGAGGGGGGAGTGCCAGAAGAGAAGTGCGCCCTGGTGGCGGAGCTGGTGCAGGTGCTGGAGATGGCGAGGCAGCTCGAGACGCACATGGCGGTGGTGGTCCAGCAGCAGCAGCAGGGCGGAGGCGGTGCCGGAGGAGGGGCGGACCAGCGGTACCGGGCGCTGGTGAGCACCATGCGCGCCTCCATCGACAGGGCCGTGCACATGGCCGTGTCCTGCTGCGCCGAGGGGCGTCCGGGCACCGGGCAGCTGCCGGAGTCGCCGCCGTCAGGTGGGGATGGCAGCAGCCCGCGCAGCGGCGGGTCGGACCACGCCGGCGAGCTCCGGGGCCGTGGCAATGCGGCCGCCGGCCAGTGCAAGAAGAGGAAGACGCTGCCCAAGTGGAGCACCCAGGTGAGGGTGAGCGCCGTGCAGGACGTGAGCCCCCTGGACGACGGCCTCAGCTGGAGGAAGTACGGGCAGAAGGACATCCTCGGCGCCAAGTACCCAAGATCCTACTTCCGGTGCACGCACAGGCACACGCAGAGCTGCCAGGCGAGCAAGCAGGTGCAGCGCACGGACGGCGACCCGCTGCTCTTCGACGTCGTGTACCACGGCGCCCACACGTGCGCCCAGGGCGCCGCCGCGCACCCCAGCAACCAGCAGCCGGCGGTCCAGGAGCAGACGACCTCCCCGTCCCCGGGGTTCGAAGCAGGGACCGCCGTGCTGCCGTTCTCGCTCCGGCCGGCCTCTAACAAGCCGACGACGGGCGCCGACGCCGCCGCGACGAGCAGCCGTTTCGTCACGACCGGCTGTGTCAGTGTCACCGCGTCTCCTTTCCTGTCGCCGGCGACGCCAGAGAGCCAGCTGGTTAGCAGCAGCAGCAGCGGCTACGCGGTGGGCGGTGGCGGCGGCGTGGCCATGGCCGGCGTCCGGAACGTGCCTGACGTGGAGCTCGCCTCTACGACCAACTCCCCTATGGCCATGGGGGAGATGGATTTCATGTTCCCGCTGGACGCCGCCGATTTCTTGGAGCTGGGGAACCCGGCCAGCTATTTCTAG

>SbWRKY86

ATGGACGGGTACGGCAGCTACGGCGGCGAGAAGAGCGCGCTGGCCTCCGAGCTGGCGCAGGTGCTGGCCATGGTCCGGGAGCTGGAGGCGCGCATGGACCAGGACCCGCTTCCGGCGGCCGCAAGGGAGCTCTGCGCCGAGCTGGCGTCGTCCGTCGACAGGTCCATCCGCATCGCCCGGTCCTGCTGCGTCGACTCGCCGGCGTCCGGGTCCGGTAGCCCCCGCAGCGACGGCGGCAATGCCGGCGCCGCCCAGTCCAAGAGGAGGAAGGGGACGCCGTGCGTGAGGAGGCAGCTGCGGGCGGCGTCGGTGCAGGACGCGGCGGCGCTGGACGACGGGCTCAGCTGGAGGAAGTACGGGCAGAAGGACATCCTCGGCGCCAAGTACCCCAGGGCCTACTTCCGGTGCACGTACCGCCACTCGCAGGGCTGCCTCGCCACCAAGCACGTGCAGCGCGCCGACGGGGACCCGCTGCTGCACGACGTCGTGTACCACGGCGCGCACACCTGCGCACAGGCCGCGCACCCCAGCGCCCAGCAGCTGCGGCAGGAGCTCCAGCTGCAGCCCGGGCATGGCGCCCAGGAGGACCAGGCCTCCCCGCTCGCGCTGGAGACCGAGGGCCTGAGGGCTGCTCTGCTAGAGCCCATGACGCCCTACTCGTTCGCCACCGTGGCTGGTGCCGGTGCCGGTGCCAGTGCTGGCGCAGACTTCGCCGGCTGGTGCCCGCTCCTCTCGCCGACGGCCTTGGACTGGCAGTTCGAGGAGCTGTTCACCAATGCAATGGAGCCCTTTCAATGGGACCTCTATACGGCCAATTAG

>SbWRKY58

ATGGAGGGTATGCCAGAAGAGAAGTGCTCCCTGGCCGCCGTAGCCGCGGAGCTGGCGCAGATACATGATATGGCGAAGCAGCTCGTGGAGCAGGTTGCGGATCCGCAGCAGGGAGGGGGAGACGGCGATGCGGCGGCCGGAGGAGGGTACCAGCGGGTCCGGGAGCTGACGAGCACCATATGCGCCAACGTAGACAAGGCCCTGCACATGCTCACGTCCAACAGCTTGGACGGAAGTCCGGCCGCGGGGCAACCGGAATCGACGCCGTCGTCAGGTGGGCATGGCAGCTCGCGCGGCGCCGTGTTGGACTCGGACCAGGCCGGTGGCGGCACCGGCAATGCGCCCGGCCAGGGCAAGGACAGGAAGACACTGTCCAAATGGAGCACCCAAGTGAGGGTGAGCAACGCGCAGGACGCCACCTACCTCGACGACGGCTTCATCTGGAGGAAGTACGGGCAGAAGGACATCCTCGGCGCCAAGCACCCAAGAGGCTACTACCGGTGCACGCACCGGCACATGCAGGGCTGTCTGGCCACCAAGCAGATTCAACGCACAGATGGCGACCCGCTGCTCCTAGACGTCGTGTACATCGGCTCCCACACGTGCACCCAGCCTTGGGGCGCCGCCGCGCACCCCAACATCCAGAGCATGCTGCCGACCACGGAGCAGACGACCACCTCAGGGTCCGAATCAGGGTCCGTGCTTACCAGCGAAATTCCTGGATCGATGGCATCGAGAAAAAGAGACACGGGAGGCGAGACTCGCCTTTCAAAAACCATGATTGAGGAGCCACATTCTACTCCCTATAAGGATGTAATGGCATGGTTTTCTATGGGAAAACTAAGACAAAAAGGACGACTCAGTCATACATGTATGCAACAGGCTGGTCAGGAACTCCTTGGCAGAGACGATATTAGACAACAGGTTATGGAAAAAATACTGTTGGATAGGAATGGCGTAAACAATTGTACTGTCATTTGCATATATGGTTGGAGTGGTCTCGGCAAGACTTCACTGCTCCATGCCCTTTACAATGATCAACAATTGTTAGACGCCTTTGACAAAAGGATATGGATACAGATATCTGATAAAATAGACATATCAATGTTATTCAGGAAGATTGTTGAGTTTGCCATGAATGAGCATTGCAGCATTACAAACATCGATTTCCTTCGAGAACTGGTTGTGGAGGAAATCACAGATAAGAAATTCTTGCTTTTCTTGGATGATGCAGACATAGTAAACCAACAATTTTGGACTACCTTACTAGAAGTTCTGAACACTGGTGCCAAAGGAAGTGTTGTTGTCATGGCTACAAGGAGCTCTACTGTTGCTGCTGTTAGGAATGTCGCAACACATTCCTATTCCTTAAATCCTTTATCTGAAGAAAACAACCTGATGCTTCTTCAACAATATGCTGTTGTGGGTACTGATATCCAGAGCAATCCTGATTTAGCATTGATTGCCAATAGGTTCATTTCTAGGTTTAGATATAATCTACTACACTTGAAGGCCATTGGTGGCCTTCTGTGCCATACAGATACTTTTTCAGTAGAGAAGGATAAGTTTGAAGGAAGTGTTATGCCTTTATGGATTTGCCATGATGTTTTACCAGTCCATCTGAAGAGGTGTCTTGCATTATGTTCCTTGTTCCCAGAAGGTTACATCTTTGGTAAACATCACATGGTTCTCCTGTGGAT

ATCTCATGGTTGTGTTAGGCCAGTTGAAGGGTACGAACTTGAAGACGTTGGAGTTGAATATTTCAATGAGTTGCTGTGTAGATCGTTCTTTCAGTGCTCACCTGTTCACAGTGATAAAAACGAAATGTTTGTGATGCACGAGCTTATGTACAAGGTGGTAGAGTCTGTCTCTCCTGACAAATATTTCAAGTCCGAGGACCCCGTGATCAGCATACCTGAAAATGTTTTTCACTGTTCTCTCATTACCTCACAATTTCAGACTGTTGAACTGATGCACAGAATGAAACAGTTGAAGCATCTGCAGACATTTATGGTGGTGCAACCTGAGTGGAAACCGAACAACATTTCTTTGCCTACATTAAATCTTGTAGGTTTGGATGATTTCTTTCTGAAATTCACATCCTTAGAGACACTGGATCTGAGCCATACTGAAACAGAAGAGCTTCCAGCATCCATTGCTGGCCTAAGAAACCTGCGGTACTTATCTGTCAACAGCACAAACGTCAGGGCTCTTCCATGTGAGCTGTGCAGCCTCAGCAATCTGCAGACACTGGAAGCAAAACACTGCCGCTTCCTCACTGAGCTACCTAGAGACATAAAGATGCTGGTAAAGCTGCGCCATCTTGATCTGACGAAGGAACTGGGCTATGTTGACTTGCCACATGGAATTGGAGAGCTCATCGAACTGCAGACATTGCCAGTCTTCCATGTCAGTGGTGACTCCTCATGTTGCTCCATCAGTGAGCTGGGAAGCTTGCACAATCTGAGGGGCTGCCTTTGGCTTTCCGGACTTGAAAGTGTGAAAACTGGCAGCAAGGCCAAGGAGGCTAACCTGAAGGACAAGCATTGCCTAAACGACTTGACGCTGCAATGGCACGACGATGGCATAGACATCGAAGATGAAGGCGAAGACTCAAAAGATGTGGCCGATGAGCAGGTCCTTGAAGGCCTCAAACCACATGTAAACCTCCAAGTTCTCACCATTAGAGGGTACGAAGGCAGGAGGTTTCCAGCTTGGATGCAGGGTTCTTCTCCATCCTTACCCAACCTGGTGACACTGACACTCGACAACTGCTGCAACTGCACCGAGTTCCCCACCATCGTGCAGCTGCCATCGCTCAAGTCCCTGAGCGTGCGAAAGATGTACGACGTGCAACAGCTAAGCAGCCACACAGACACACATGGCAATGGCAGCACGGCCAAGTTCCCGTCGCTGGAGCTGCTGAACCTGTGGGAGATGTACGGTCTAGAGGAGCTGTTCTCCAAAGAATCTGAAGGGGACTGCCCTCGCCTCCGCAAGGTCTGCATCAGCCGGTGCCCGGACCTGAGGAGGCTGCCCAGCGCTCGTTCTCTGACGGAGCTGGTTCTCCATTGCGGCAAGCAGCTCCCTGACATCTCGGAGCTCGCGTCGTTGGTGTCACTGAAGATTGAAGGCTTCCACGGTACCAAGTCGTTCGGCTTGCCGGCAGCTGCGGCGCTGAGGAAGCTGGAGATCAGGTCTTGCAAGGAGCTGGCGTCGGTGGACGGGCTGTCGGCGGTGCTGACCACCGTGCAGAGGCTTAAGATAGCAGGGTGCCCCAAGCTCGTCTTGCCAGGAAGGAACCAGTAG

>SbWRKY91

ATGCAAGACGGCGCGGGAGCAGAAGCAGCAGGGAGCAGCATGCAGGCGCTGCTGGCGCTCCTCGCCGACGGCGAGGAGCAGGCGCGGCAGCTCGGGGAGATGATGGCCGACGACCCGTGGTCACGGGCGGAGCACTACAGGGGCGCGGCGCGGCGGCTGCAGTGCACGCTCGGGAAGGCGGCGGCCGTCGCCAGGGCCATCGAGGCGGCCGCGCCGGGGTCGTCGCGGGGCACCGACGACCGCTCCGACTCGCCGCGGTCGGCGGACGAGAGCTCTGGCCGGACAACGACGGAGGTGCAGGAGCGCCAGAGCATGTTCAAGAGAAGGAAAGGTCTACCAAGATGGACCGCGAAATTCCGCGTGCCAGATGCAAGCTTGGATGCCACCCCGGACGACGGCTTCAGCTGGAGAAAGTACGGCCAGAAGGACATCCTCGGCGCCAAGTTTCCCAGGGGCTACTACCGGTGCACGTACCGCACCGCGCAGGCGTGCGGCGCCACGAAGCAGGTGCAGCGCTCCGACACCGACCTGTGCGTGTTCGACGTCACGTACCAGGGCGAGCACACCTGCCACCAGAAGCAGCGCGCCAGCGCCACCGTGGCCGCGGCGCCCGCGCACGGCGCCGGGAGCCAGTCGCCGCCGCCGCCGCCGCCGCTGGAGCAGCAGCAGCAGCAGCAGGACCCGAGCATGATGCAGCTGCTGAGGTTGGGCTTCAAGCGCGTCCTCAAGGTGGAGACGACGCCGGGGCTCCACGACCATGGAATTGGCCACCGCGACAGCGGCCCCGCCTCCGCGCCCGCCGCGCCCTTCTCCTTCCCCTCCGCGTCGCCGTTCCACCTCGCCGGCGAGGCGACCGACAACCCCGCCGCGGCCTTCTCGCCGCCGCCCGCGTCGAGCTACTTCCCTGCGCCGCACCCGGTGGCGGTCGACGGGAGCTTCTACGACTATGAGGCTAGTCCGGTGGCGCTCATGCGCGGGGCGGAGCCGTCGGAGCTCGGCGAGGTCGTCACCAGGGCGATCACCACCGGCCCCGCCGCGTTCGACTACTCGTCACTCTTCCACCACCAGGCTGAGCTCGACGACCCGCACCTGCCGTTCCCGCCATTTGGCGGCCCGCCCCACGGACCATACCAGTAG

>SbWRKY17

ATGCTGCTCATGGACTCGGCGCGCCGCGCCGGCTGCTCCCCGTCCCCGGTCTGCTTGGACCTCAGCGTCGGCCTTTCGCCGTCGTCGCCGGGGAGCAGCGGCCCGGAAACGACAGCTGACACTGACGACAGGCTTGACCGTCCCGCCGCTGGCTGCAGGGTGGCATCGTCCCTGTCTGACGAGCAGGCCAAGACCCTGGAGGCCAAGCTCACCCAGGTCAGCGAGGAGAACCGCCGGCTCACCGAGATGATCGCCTACCTGTACGCCAGCCAGGTCGCGCGGCAGAGCTCCAGCTCCCCCGACACCACCAGCAGGAAGAGGAGCAGGGACAGCCTGGAGCCGCCGTCGAATTCCAGCGACGGCAACGCCAACGCCAAGGCGGAGCCCGGCGACCATGCCGCCGTCGAGAGCGCCCTCAGCGACGAGGGCACGTGCAGGCGGATCAAGGTCACCAGGGTCTGCACCCGGATCGACCCCGCCGACGCCACGCTCACCGTCAAAGACGGCTACCAATGGCGAAAGTACGGCCAGAAGGTGACCCGCGACAACCCGTCCCCGAGAGCCTACTTCCGCTGCGCATACGCTCCCTCCTGCCCCGTCAAGAAGAAGGTGCAGAGGAGCGCGGAGGACAGCTCCTTGCTGGTGGCGACGTACGAGGGCGAGCACAACCACCCGAGCCCGACGCGCGCCGGCGAGCTCCCCAGCTCCGCCTCCGCGACGGCCAGCGGCCCCGTGCCGTGCTCCATCTCCATCAACTCCTCCGGCCCGACCATCACGCTGGACCTCACCAAGAACGGAGGGGGAGGCGGCGTGCGGGTGCTCGACGCCGCCGAGGCGCCCGACCTCAAGAAGCTGTGCCAGGAGATCGCGTCGCCGGATTTCCGGACGGCGCTCGTGGAGCAGATGGCGCGCTCGCTGACCAGCGATTCCAAGTTCACCCACGCGCTGGCTGCCGCGATCCTGCAGCAGCTGCCGGAGTACTAG

>SbWRKY18

ATGGCCATGGACAGCACCAACGGCGAGTGCTCGTCCCCCACCGCCAGCGCCGTCGGGCTTCTGCCGCTCTTCGGCTCGTCGCGGCCGCCGCCGCAGGCAGAGAGTCTGGAGGAGAAGCTGAGGCGGGTGAGCGAGGAGAACCGGAGGCTGGCCCCCGCGCTGGACGCCATACTCTCCGCCGACCGCTCCAACCACCCGCGAGCGCTCGCCACGTCGCCGCCGGCCCAGCAGCAGGGCAATGCGGCTTTGACGACGCAAGCGGCCACCGGCGTCGTCGTCACCGCGGAGCCGCGGCACAAGGTCCGCACGGTGCGCGCGCGCGCCGAGCCCGCGGACGCCGACGCCAACCACCTCAAGGACGGCTACCACTGGCGCAAGTACGGCCAGAAGGTGACGCGCGACAACCCCTACCCGAGAGCCTACTTCCGCTGCGCCTACGCTCCCTCCTGCCCCGTCAAGAAGAAGGTACAAAGGAGTGCAGATGACAATTTGATGCTGGTGGCGACGTACGAGGGTGAGCACAACCATGAGCAGCATGCCCAGAGCGAATACTCCTACATCAACGACGCATCGACGACGAGCCAGCAGCAGCAGCCCCAGGCCGGCGGCTCGTCGTCGTCGACACTGCCGTGCTCCATCATCTCCATCAATTCGCTGGGCCGGACGATCACCCTTGGCCTGGCCGACCAACGGCGGCCGGGATCGAGTTCGAATGCTGAGGCGGCGGCGGTCGTCGTCGGCGAGATTGTAACGCCTGAGCTTCGAAAGGTTTTGGTGGACGAGCTCGCGAGTTTGCTCAAGAATGATCCCGAGTTCATCGAGTCGCTGGCGACCGCCGTGGCTGATAGGGTGATGGAGAGAATACCAGCAGCAGGGCACATACTCTGA

>SbWRKY45

ATGGATCCATGGATCAGCAGCCAACCTTCTCTGAGCCTTGACCTGCACGTCGGCCTGCCGCCGCTCAGCCTCCACCAGGCGCCGGTGGCCGCCGTCGCCTTGGCCCGGCCCAAGGTCCTCGTCGAGGAGAACTTCCTGCCTCCAAAGAAAGAACCAGAGGTCGCGGCTCTGGAGACGGAGCTACACCGGATGAGCGAGGAGAACCGGCGGCTGACGGAGGCGCTGGCGGCGGTGGCGTCCAAGTACGAGGCGCTGCGGAGCCAGTACACGGAGATGGTGGCCGCCGCCGCCGCGGGCACGAACAACAACCCGTCGTCGACGTCGGAGGGTGGGTCCGTGTCGCCGTCGCGGAAGCGCAAGAGCGAGAGCATGGACACCGCGCCGGCGCCGCCTGCCGCCGCGCAGCAGCAGCAGCAGCACGGCACGCACCTGCACCAGCAGCAGCACCCGGGCCTCGCCGCACCGGACCAGAACGAGTGCACCTCCGGCGAGCCGTGCAAGCGCATCCGGGAGGAGTGCAAGCCCAAGGTGTCGAAGCTGTACGTGCACGCCGACCCCGCCGACCTCAGCCTGGTGGTCAAGGACGGCTACCAGTGGCGCAAGTACGGGCAGAAGGTGACCAAGGACAACCCGTGCCCGAGGGCATACTTCCGCTGCTCCTTCGCCCCGGCATGCCCCGTCAAGAAGAAGGTGCAGCGGAGCGCCGACGACACCTCCATCCTCGTCGCCACGTACGAGGGCGAGCACAACCACGGCCAGCCCCCACCGGCGGCGCCGTCGCAGGCTGCCCACGACGGCTCGGCGGCCCCGGGCGCCACCAAGAACGCCGCTGCCGTCGCGAAGCCGCCGTCGCCGCCGAGGCCGGCCGCACCGGCGCCCGCACCGGCGCCGCACCGCCCGCAGCTGCAGCTGCTGCAGCAAGAGGGTGTCGCGATGAACGTTGAGCAGCCCGTGGCCGCGGCGGCGTCGGAGATGATCCGGCGCAACCTCGCGGAGCAGATGGCGATGACGCTGACAAGGGACCCCAGCTTCAAGGCGGCGCTCGTCACCGCGCTGTCCGGCCGGATCCTCGAGCTGTCGCCTACCAAGGATTGA

>SbWRKY94

ATGGACCCGTGGGTCGGGCACCAGCCTTCCCTGAGCCTCGACCTCAACGTCGGCCTGCTTCCTACGGCGAGGCCGGCGGTTCCGGCGAAGTCGACCAAGGTCTTGGTCCAGGAGAACTTCATGGCCGTCAAGAAAGACAACCGAGAGGTCGAGAAGCTCGAGGCGGAGCTCCGGCGCGTCGGCGAGGAGAACAGGCGGCTGAGCGAGATGCTGCGGGCGGTGGTGGCCAAGTACACGGAGCTGAAGGGCCAGGTCGACGACATGGTGGTGGCCACCGCCAACCACACTGGGTCCTCCACGTCGGAGGGCGGCTCCGCGGCCTCGCCGTCCAGGAAGCGGATCCGGAGCGCCGGCGACAACAGCCTCGACACCGCCGCCCAACACCACCACAGACGCAAGCCGTCGCCGCCGTTGGCGGCTGCCGTCGCGGCACACGACCAGACGGAGTGCACGTCGGCCGCCGTCAGCGTCACCGCCGCCGCCTTCCGGCGCGCCGTGCGGGAGGAGTGCCGGCCCAAGGTGTCCCGGCGATACGTCCACGCCGACCCCGCTGACCTCAGCCTCGTGGTGAAGGACGGGTACCAATGGCGGAAGTACGGGCAGAAGGTGACCAAGGACAACCCGTGCCCGCGCGCCTACTACCGGTGCTCCTTCGCGCCGTCGTGCCCGGTGAAGAAGAAGGTGCAGCGCAGCGCGGACGACAGCACCGTCCTGGTGGCCACGTACGAGGGCGAGCACAACCACGGCCAGCCGCCGCAGCACGACGGAGGCAGGGCCGCGAGGTCGACGGCGACGGCGCAGGCGCAGGTGGCGAGCGAGGCCGCCGTGCGACCGGTGGCGGCACCACTGCCGCTGCAGCACCCGCACCAGCAGCAGAAGCAGAAGCAGAAGCAAGAGGCGGCGACGACGGTGCCGTCGTCGGAGGTGGCGAGGAAGAACCTCGCGGAGCACATGGCGGTGACCCTGACCAGGGATCCCGGGTTCAAGGCGGCGCTAGTCAGCGCGCTCTCCGGCCGGATCCTCGAGCTCTCCCCGACCAGGGATTAA

>SbWRKY22

ATGGACAAGGCCCACCTCGGAGTCGGAGGGGGTCTGTTAGCTCTCGACGCGTCGCCGCGGCCCCTTGGCTTCCTCTCCCCCACGGCGTTCCACAGAGCAAGAACAACAGCCATGGAGGCCGCCGACGACGGAAACGGAACACCCCCGCCAGGCAGCAGGGTCCGTAGGTCCGTCGAGGTGGACTTCTTCTCCGACCAGAAGATCGCTGCCGATGCAGCTAACAACAACACCTGCGGCAGGACCACCGTCTCGCCGGGATCGGGATCAGGAGCCAGCTGCCTCGCCATCAAGAAGGAGGATCTCACCATTAACCTCCTTCCCGGCACCGGCAGCAACGCCAACGACGACGAGGCGGCCACCCGGCTCCGGCTGCTGGACCAAGACAAGCAGAGCAGGAACACCAACGAGATGCAGGCGGAGCTTGCGCGCATGAACGACGAGAACCAGCGGCTCCGCGGGATGCTGACCCAGGTCACCAGCAGCTACCAGGCGCTCCAGAT

GCATCTCGTGGCGCTCATGCAGGCGCGGGCCGGCGGCCAGGCCCAGCTGATGCTGCCTCCTGTGGCCCAGGCGCTGCCGCCGACGACCGACGGCGCGGCTGCTGCTGTCATGCCATTGCCGAGACAGTTCCTCGGCCTAGGACCGGCTGCTGCTGCCGAGGAGACGTCCAACTCGTCCACGGAGGTGGGAAGCCCGCGGCGGTCGTCGTCCACCGGCGGCAACAGGCGGGCGGAGCGCGGCGACAGCCCGGACGCGTCAACAAGGCAGCAGCAGGTAGCGCAGCAGCAGCAGGAGGCGAGCATGAGGAAGGCGCGCGTCTCGGTGCGCGCGCGATCCGAAGCGCCCATCATCGCCGACGGCTGCCAGTGGCGCAAGTACGGGCAGAAGATGGCCAAGGGCAACCCATGCCCGCGAGCCTACTACCGCTGCACCATGGCCAACGGCTGCCCCGTGCGGAAGCAGGTTCAGCGCTGCGCCGACGACCGCTCCATCCTCATCACCACCTACGAGGGCACCCACAACCACCCGCTCCCGCCCGCCGCCATGGCCATGGCCTCCACCACCTCCGCCGCAGCCTCCATGCTGCTGTCCGGCTCCATGCCCAGCGGGGACATGATGACCTCCAACTTCCTGGCGCGGGCCGTGCTGCCGTGCTCCTCCAGCATGGCCACCATCTCCGCCTCCGCGCCGTTCCCCACCGTCACGCTCGACCTCACACACGGCCCGCCCGCTGCTGCACGACCGCAGCCGCACTTCCAAGTCCCGCTGCCACCGCACCAGCAGGTCCAGCAGCAGCACCACCACCTGCAGGCGGCCGCGCTCTACAACGCCCACCAGTCTTCGTCCAAGTTCTCCGGCCTGCACATGTCATCGTCATCGACATCGGACAATAACAACAATGTTGGCACTAGTAGCAGAGCCGCCGTGGCAGCGGCTGACGCGCCGCCGCACATGGACACCGTCACTGCGGCGGCGGCCGCCATCACCGCGGACCCCAACTTCACGGTGGCGCTGGCGGCGGCCATCACGTCCATCATAGGCGGCGGCGGAGGTCATCCAATTCCAATTGCAATTCATCATGGGCAAGGGCAAGGGCAAGAGCAGGGGCAGCAGCAGGCCCCGACGAGTAACAGCAACGCCAACAACAACAACAATGCGGTGGTGACGAGCAGCAGCAACAACACGGCGACCAGCAACAGCGAGACCCAGTGA

>SbWRKY85

ATGGAGTCCTCCTACCTGGGAAAGCGCAGGCTGAACGGCGGCGCCGATAGGGAGACGACGACGGCCAGGGCGCCGGCTGCGTCGTCGTTCCTCCCGGCCGCCGCGATGGGGTACGAGTACGGTGACGCGGCGGAGGCGGCGGACCACCACCACCTGCCTCGCCGGGTGGCGGCGGGCGAGATGGACTTCTTCAAGAAGGAGAGGAAGGACGCCGCCGCCGCCGCCGCCTTGGCCGCCTTTGTGCCGTCGTCGTCGGACGAGCACGGCATCAAGGAAGACGACCTCACCATCAACATGGGTCTGCACCACGTCAGCGGGAGGAAGAGCAGCATCAGGAGCGAGGAGTCCAGCGTCGACGACGGCGTCTCCTCCAACGGCGTGGATCACAGGGAGACCAAAGCTGAGCTGGCACTGGCAAAATCCGAGCTTGGGCGCCTGAACGAAGAGAACAAGCAGCTCAAGGACATGCTCAGCAGGATGACCATCAAGTTCAACGCCTTCCAGGTGCAGATGCCGGTCTACACTACACTGATGCAGCAGCAGCAGCAAAGGACCAATAACCATCAAGCCCTTCTCCGCGGAGCTCCAGGCCATGAGCTGATGAACGTCGATCCGGAGACGAAGGATCATCAGGAGGGGAGCGGCGGCAGCCACCTGCTCCCACGGCAGTTCATCAGCAGCCTCGGCACCGCCCCCGACGACCCGCTGCGCTCCGTGGGCTCGGACGCAATGCACGGCGGCGGAAACAGCTCGGGGTCGTCCACCAGCAACGCGGAACCGCCGCCGCCGCAACCCTTGGACTACTGCCCTGGCAACGGGCTCATGGTCAGCAGCAAGGAGATGATGCCGCTGCCGGCGTTCGAGCACGGCCACCAGCAGCCGCAGCAGCACCTCGCCCACGAGATGGGTAGCAGCAGCCGGGCGGATGAGCCGCCGCAGCCGCACCACCTGGCGGCGGCGCAGCAGGGCTGGCTCTCCAACAAGGTGCATAAGTTCCTCCCCTCCAAGGGCCCCGAGCCCGTCCCCGAGGCCGCCACCATGCGCAAGGCCCGCGTCTCCGTCCGAGCCCGCTCCGAGGCACCCATGATCAATGATGGGTGCCAATGGAGGAAGTACGGGCAGAAGATGGCCAAGGGCAACCCGTGCCCCCGCGCATACTACCGCTGCACCATGGCCGCCGGCTGCCCCGTCCGCAAGCAGGTCCAGAGATGCGCCGAGGACAGGACGGTGGTGATCACGACGTACGAGGGGCACCACAACCACCCGCTTCCCCCGGCGGCGATGCCGATGGCGTCGACGACCGCGGCGGCGGCGTCCATGCTGCTGTCCGGGTCGATGCCGAGCGCGGACGGCGGCAGCCTGATGGCGGGGTCCAACTTCCTGGCGCGCGCCGTGCTGCCGTGCTCCTCCAACGTCGCCACCATCTCGGCGTCGGCGCCGTTCCCCACCGTGACGCTGGACCTCACGCAGCCGCCACCGGGAGCCGCCTCCGCCTCCGCCTCCGCGTTCGCGCAGCCGCCGGCGTCGGCGCCGGCGCAGGCACGGGCGACGGGGACGGAGCCCTCCCAGCTCCAGGCCGCGCTCGCCGACGCCGCGGGCCGGCCGATGCCGCTGACGACGCAGCTGTTCGGGCAGAAGCTGTACGACCCCTCCTCCAAGGCCCCCGCCGCGCAGGCGGACGCGGCGGGCGACACAGTCAG

CGCGGCGGCCGTGATCGCGTCCGACCCCAACTTCACCGCGATGCTCGCGGCGGCGATCAAGTCGTACATAGGCAGCAGCGGCAGCGGCAGCAATGGCGCCGGCGGGAGCAGCGGGACGACCGTGCTGCCGCCGGCGGGGGCGAGCAGCGCCGGCGACAGCAGCAGAGACGACAAGGTCGGGGAGCAAGGGAGCTGA

>SbWRKY26

ATGGAGACGAGGCCGGAGCGCCACCGCCACGACCACGTCCACGAGCAGCAAGCCCAGGAGGGGGAGGAGGACGACGCAGCAGCAGCAGTCATGGAACACGGCGCTGCTCTCTCGCTGCAACGCGGAGCCACCTTGTTTGGGCGCCGGCGTCAGCACGATGAGGAGGCCGACCGCCGCCGCCGGGGCGAGATCCGGGAGGTGGACTTCTTCTCGAGGGACTCCGGAGCCCGGGGCCAGGACGACGGCGGCGGGCGCGGGGTGCCCGGAGGCGGACGCGACGACGTCAACATCGGGCTAGACCTGCTGACCACCGCCACCGCTGCCACGACAAGCGCCGCCGGCGAGGAAATGATGGCGGTCAAGAATCAAAAGATAGAGGCGTCAGCTGTGGAAGTGGAGCTCAGGCGGGTGGTGGAGGAGAACCGGCGGCTGCGTGGCATGCTTGAGGAGCTCAACCGGAGCTATGGCGCGCTGTACCAACAGCTTCTCCAGGTCACGCAGCACCGACAGCATCCTGCTGATCTCATGATCAACAGATCATCACTGGCTCATACCCACCTGACGACCACCGCGGCCTCGCACAACACGTCGTCCACTCGGCAGCTGTTGGAGGCGCGCGCGTCCTCTACTGCTATGGCGCAGCCGCATGCCGTCGCCGCTGGAGGGGACGACGAGGCTAGCGACGGAGCCGAGGAGGCGTCGCCTTCGCTGAGCAATGGAGGAAACAACAACGACGACGCCGATGGCAAGAGGAAAACCTCGCCGGATAGGACAGCACCACCGAGAGAGAATGGCGGCGAGCAGGCGTCGTCGGAGCTGCCCGGCCGGAAGGCAAGGGTGTCCGTGCGCGCGCGATCTGAGGCACCAATGATTAGTGATGGGTGTCAATGGAGGAAGTACGGGCAGAAGATGGCCAAAGGTAACCCATGCCCGAGGGCATACTACCGGTGCACAATGGCTGTGGCATGCCCCGTCAGGAAGCAGGTGCAACGGTGCGCCGAGGACAAGACAATTCTGGTCACGACTTACGAGGGACACCACAACCACCCGCTGCCGCCGGCGGCCACCACCATGGCGAACACCACGTCCGCCGCGGCGGCCATGCTGCTCTCCGGCCCGGCCACCAGCCGCGACGGCGCTGCCGCGGCGCTCCTCGGCCACCCGGCGCTGTTCCACCACTCCAGCAGCATCCCCTACGCGTCCACCATGGCCACGCTCTCTGCCTCCGCGCCGTTCCCCACCATCACCCTCGACCTCACCCAGGCGCCCGGCGGCGTTGCCGGGAGCGGCGGCGGCGGCCTGCTGCCTCACGGGCTCGGGCTCCATCGCCCACCCGGCGGGATCCACCCTGTGACGGCGGTCCCGGCGATGCCGTTCCCGGTGCCATCGCCGCTGGCTTCGATGTTCCTTCCACAGCGAGCGCCCACGGGGCCGCCGATGCCCACCGGGCTGCAGGTCGCGCGGCAGCAGCAGTCGGTGATGATGGAGACGGTGACAGCAGCCATCGCGGCCGACCCCAACTTCACCACGGCGCTTGCGGCGGCCATCTCGTCGGTCATGGCAGGCGGAGCAGCGCACCAGGCTCAGCCTACTCCACGTGGGAGTAATATCATCGGCATCGCCGCCGGGGATCAGGCCAACGGCAGTGCGGGTGCTGCTGCCATTGCAGGTCCGACGGCAGCCGGAGCACATGCAGCGTC

TGCCGGGTCACCTCGTTTCGCGACGCAGTCCTGCACCACGTCAACTTGA

>SbWRKY76

ATGGACTTGGTGCCGAAGCAGCAGCAGCAGCAGCGGAGCAAGGAGAAGCAAGAGGAGGAAGAGGAGATGATGATGGCCTTGGCCGAGCACGGGGACAGGCCGCAGGCGGCGGCTTTCGGTCACGGTGGCGGCGGAGGCGGCGGGCGCAGGAGCGAGATCAAGGAGGTGGACTTCTTCTCCACCGCCGGCGGTGCCGCTCGCCGCAGGACCGACGACGACGACGATGGGGATCGAGAGGAGGCCGCCGCAGGAGCACTGGCACGCGGTTGCCATAACACCACGGTCAACACTGCACTTGACCTGCTGACCACCAGGGCGGCGGCGGCGGCGGCGACGCCAGCAGCAGTCGACGGCGGCGAGGGTACGGCGAGTGGTCGTGATACAGAGGTACCAATAGTGGATGTGGCGGCGACGGCGGCGGTGGAGGGGGAGCTCCGGCAGGCCAGCGAGGAGAACCGGCGGCTGCGGCGGATGCTGGAGGAGCTCACCCGCAGCTACGGCGCACTCTACCATCAGCTCATCCAAGCCCAGGCTCAGCAGCAGCAGCAACAGGCCTGCAGTGGCGGCGCAGCGAACCCGATGCTACCGGCGGCAACGACGACGGGAGTACAGTTCATGGACCACGCCGGCCGCGTTGCTCCGGCGATAGCAGGAGAAGCGGCCCCGCCGGCGTTCAGCGGTGACAGGGGCGATTCAGACGATGGCAGCGGAGGAAATGGCGGCGAAGCGGATCAAAACGATGGGATGAAGACGCCTGAGCGCGGCGAGAACGTCGACCGGTCACCGGCGGCGGCGGCGGAGGCGCCTTTGCGGCGGGCAAGAGTGTCCGTGCGCGCACGGTCCGAGGCCCCAATGATCAGCGATGGATGCCAATGGAGGAAGTATGGGCAGAAGATGGCCAAGGGTAACCCATGCCCCAGAGCTTACTACCGCTGCACAATGGCCACAGGATGCCCAGTCAGGAAGCAGGTACAACGGTGCGCGGAGGACAAGGCGGTGCTGATCACCACGTACGAGGGCACGCACAACCACCAGCTGCCACCGGCGGCGGCCGCGATGGCCAAGACGACCTCCGCCGCGGCGGCCATGCTCCTCTCGGGCCCGGCCGTCAGCCGCGACGTGGGCGCGCTCTTCGCCGGCCACCACGTCGCCGCCCCGGCGCCGCTATTCCAGTACCACCACCCCTACGCGTCCGCCATGGCCGGCGCCACGCTGTCCGCCTCCGCGCCGTTCCCGACCATCACCCTCGACCTCACGCACGCGCCGTCGCCGGGGGCGGCGGCGGCGGCTGCGGCTGCCGCGGGCCTGCTCCAGCAGCGCCAGCTGATGCCGCCGCCTGTCCCCACGATGACGCCGTTCCCGATGTACGGCTTCACTGCTGCCGCTGGGCACAGGCCGGTGCCGCCGCCACAGCCACCGGCGGCGACGACGCTGTTTGGTTTGGACGGCAGCAACCGGTCGGCGCTGGAGACCATGACTGCTGCGATCACTAACGACCCCAACTTCACCACGGTCGTGGCGGCCGCCCTCTCGACGATCATGGCGGGAGGCGCAGAGCCACCGGTTCCCCGGAGCGGCGCCGCTGACGCCGGAGATGGTAGCAACGGTAGCGTTGGCATTGAGCCTGCCACGGCGGCGGCGGCCGGAGCACGTGAGAATGCATTGCATGCACTTTTACAAAGACTTCATGACAGCCGGCA

ATGA

>SbWRKY24

ATGGAGGTCGCCGTCGAAAGGCCGCCGCCGGCGCCGCAGGTGAAGGCGGAGGAGAAGAGACCCGACGCCAAGCCAGAGATCGCCGCCCGGCCACCGATGGTAGGAAGCGCTCTTCCGATAGTCTTCGAAAGCTTTCCATCGACGCAAAGAGACGCGGCCGGCGGCATCAACGTCAAGCAGGAGGAACGCAGGCTGGAGGCGGCCAGGGCGGAGATGGGCGAGGTGAGGGAGGAGAACGAGCGCCTCAAGTCGATGCTGTCCCGCATCGTCAGCCAGTACCAGTCCCTGCAGATGCACTTCCTCGACGTCGTCAAGGTGCAGGAGCAAGCATCGTCTGCAGCCAAGGTGGCCGAGAAGAAGCTCCCCGTCGCGCCGGCGCCGGCGCCGAATCCCGGCACCGACGACGACGGCCCCGACGACCTCGTCTCCCTGAGCCTCGGCACCAGGGCAAATAGCGGTGGCGCACCCCGCCGCAAGGGGCACGAGAGGTCGTCGTCCTCGTCCGGCACCGCTGAGACAACCACCGCCGCCGACGCTGATGACCAAGGCCACCACCAGCTCTCCCTCGGTCTTGGTTTCGCGCGCGGAAACGGGCTGCCGTCGTCCACCACGACGGCCACGGACGACGACAAGGCGAGCCACGCGTCCACGGCGCCCGTCCTGAACCTGACCTCCGACAGCAGCGGCAGTGCCGACGACAACGACGACGCCAAGCCTGCCCTGGCGGCCGCGGGCACAGCCCGTAAGAGCCCGAGCGCCGGTGCCGGTGCCGGAGACAGATCAGCTGACGATGAGGTGCAGCAGCAGGCCAAGAAGGCTAGGGTTTCCGTCAGGGTCAAATGCGACACTCCCACGATGCCCGATGGCTGCCAATGGCGCAAGTACGGGCAGAAGATCTCCAAGGGGAACCCGTGCCCGCGCGCCTACTACCGCTGCACGGTGGCGGCGCACTGCCCGGTGAGAAAGCAGGTGCAGCGGTGCGCGGAGGACACGTCGATCCTGATCACCACGTACGAGGGCGCGCACAATCACCCGCTAACGCCGGCGGCGACGGCCATGGCGTCCACGACCTCCGCGGCGGTGGCCATGCTCACCTCGGGCTCCACCACCTCCGCCGCCTCGGCCTCGCTCGTCCACGGCCACGGCCACCCGCTGCCGGCCGCCGCCGGGCTGTTCGGCCCCACCACCATGGTCTCCACCGCCGCGTCCTGCCCCACCATCACGCTCGACCTCACCTCCCCCGCCGCGCCGCACTCCCTCATGCACTCCTCGCCCTACGCCGCCGCCGCAGCAGCAGCAGCAGCAGCGGGGTTCGAGTCCAAGGCGTTCCCGGCGGCGTGGAGCAACGGGTACCTGGCGTACGGCGGCGCCCACCCGTCCTACTACTCCAAGAGCTCGACGTCGCCGGCTCTGGGGCACCTGTTCGGTGGAAGCCTGGGCGTGCCATCGAGACCGGAACAGCTGTACGCCCAGTCGTACCTGCAGAGAGCCAGCAGCCTGGGCGGCGGCCATGGCGCGGTGGCGCCGGCGGCCGTCACGGACACGCTCGCGAAGGCGATCACGTCCGATCCGAGCTTCCAGTCCGCCCTGGCCGCGGCGATCACGTCCGTCATGGGCCGCGGCGGGGCCGCTGCTGCCCAGAAGTGA

>SbWRKY90

ATGATTGGAACTAGGATTAGGGAGACATATTCAGCAGCAAATTCTGATGTCGAAATCAATCAGATCAAAGATGATGATCATATCCTGGATGGCAACTTGTTTAAGTCCTTACACGAATCAAGTTCAAGAAAAGAGGCAAGCTCCTCAAGCCTAAGGGAAAGATCTGAAACGGACGAAGCATCAAATCAAACTTCAGCTAATCATAACAAGGTTGATAAAGATAAACTTGCATCCACAAGGGCAGAGATGGGTGAGGTAAGAGAAGAGAACAAGAGGTTAAAAACTATGTTGTCACGCATTGTAGAGGACTACCGATCTCTTCAATTGCACTTCCATGACGTTCTTCAAAAAGGACAAGCCAAGAAGCTTGCTGACCCCTCGACCATCATGCCCACCGGCATCGAGGAGCCTGAATTCGTCTCACTGAGCCTCGGCACGACCACAAGCATGCACAGGAAGGAAGACAAGAATAGTGCTGCTGAAGGAAAAGGGAGAGAAGACTTCATGAGTATTAAAGAAGAAGGCCTGTCACTTGGACTGTCAGCCTGCAAAGATGGTGCAACTAATAACAATGTAAAGATCCAGCCCGAAGTGATGACCTTGAGCCCTGAAGTTAGTTCCGAGGATGCCAAGGATGATGCCATGGAGGCAGCAGATCAGCAGTGGCCGCCAAGCAAAGCAGAGAAGAGCTTGAGGAACGTTGGCACGGGGCCTGAGGATGACATAGGTCCACTGCCACAGGCCAAGAAGGCAAGGGTGTCTGTAAGAGCAAGGTGTGATGCACCAACGATGAATGATGGATGCCAATGGAGAAAGTATGGGCAGAAGATAGCCAAGGGGAACCCATGCCCCCGTGCCTACTACCGGTGTACAGTGGGAGCAGGATGCCCCGTCAGAAAACAGGTGCAGAGGTGCGCGGACGACATGTCGATCCTGATCACCACGTACGAGGGCACGCACAACCACCCGCTCTCCGCCTCCGCCCCGCCATGGCCACCACCACCTCCGCCGCGGCGTCCATGCTCACCTCGGGCTCCTCTACCTCCCTCCGCTTCCCCGCCGCCTCGCCGGCCGCCGCCGGCCTCAGCTTCGGCTTCCC

TCCGGCGGCGGCGCACGACCCCTCCAAACATTTCTTCCTCCCGAACGGCGGCGCCGCGTCCATCACCTCCACGCCGTCCTACCCGACCATCACGCTCGACCTCACCTCGCCGGCGGCCACCTCGCAGGCATTCTCTCTGGGCAACAGGTTCTCGTCGAGCTTGGTTCACGGCGGCGCTAGGTACCATCATCCCACGAGCCTCTCCTTCTCCAACTCCGGGCCCAGCGCGCTGTCCGGCGCTGCATGGCCGGCGGCCGGTGGTGCTGGGTACCTGAGCTACGGGTCACCAGCAGCCTCGTTGTTCAACGGTGGCGCTGCACTGAGCAGCATCAACGGAAGGCAACAAGGCGGGGAATTCCCCGTGCTCTACCAGCCGCAGCAGAAGGCGTCGGCGGCGGCGAGCGGGAGCGCACCGGAGGGCGTGCTCACCGACACGATAGCGAAGGTGATCACGTCGGACCCGAGCTTCCAAACGGTGCTGGCAGCCGCCATCACGTCGTACGTCGGCACGCAGGGAGGTAACAGATCGTCGGCGGGAGGAGAGGGCGGGAGCCAGCTGCAGGGGCTCAAGTGGGGACAGCACCTCGGCCTGGGGCCGTCGCCATCAAGCCCGGGTGCGGCGTGCTCGTCGGCGCTGCTGGCACGGTCATCGTCGACGACGGCAGCAGCGGTGGTGGAGCAGGGTTCCAATGGGCACCGGTCGTTCTTGCAACCATCGCTGGGCTTGTCAGGTTCTCACAGCACCTCCACCTCTCCTGTGGAGAACAGGGAGCACTGA

>SbWRKY28

ATGGAGCTCAAGACTTTTGTTTTCAAGGAGCTGATGAGAGGCGCAGCTGACAAGATGAAGTTGGGCGACGAACCGGCCGCCGGGCCATTTCTGTCACTTAGCCTCGGGCCGGCGAACGCGATCGGCGCAAGTCTCCGCACGAGAGGAGGGGAGGCGATCAATGCATCGCAGGCGCCACCGCACGCCGCCAGCAACGCCGACGACGGGATCGGTTTGGCCCTCGGGTTGCGCTGCGACAGCGATGGCGGCGGCGAGCCGGTCCTCGCCGCCGTCGTCGGCTCTGCTGCTGGCACCAAGCGGCAGAGGGTGGCAATACTAAGCGACGACAGCGGCGGCAATAGAGGAAGCAATAAGGCGCTGCGACTGCCGGCGCTGCTGGCACCACCGCAGCAGCAGAGGCCGGCGGGCCGCGTCACCTTCAGGGCGCGGTGCAGCGCGGCCACGGTGAACGACGGGTGCCAGTGGCGGAAGTACGGCCAGAAGGTGGCCAAGGGCAACCCGTGCCCGCGCGCCTACTACCGCTGCACGGGCGCGCCCGACTGCCCCGTCAGGAAGAAGGTGCAGCGGTGCGCGCACGACGCGGCCGTGCTGGTCACCACGTACGACGGCGCCCACAACCACCCGCTCTCGCCCTACGCCGCCGCCATGGCATCCGCCATGCTCGCCTCGTCGTCGTCCTCCTCATCATCTGCTACCGCCGCCAGCTGCGACGACGACGCGCCGCGGCTGGCCTTCCCCATCTCCGTGCTGCCACCGGCGCCGCAACGCTATTCGTGCAGCCGCGACGTGGCCATAGGCGCCGGTCCGCCGCCGGCTGCGCCTGCGGCGACGAGTCACGGCGACCTCGTCCCGATGGCTAACATCATGCAGAAGGCGGTCGGGGACCCCAACTTCCGCGCGGCGGTGATGGCCGCCGTCGCCAGCTACGTCGGTGAGCAGTGCGGTGGAAGAAATATCTCGACGACATCTTCACCTTGCCACCTCCCTGCTAATAACTGA

>SbWRKY50

ATGTCATCCAAGAAGAAGAGGGCCGCGATAGATCTCTCCCTAGAGGTAGAGAGGAGTGACGAAGACCATGGCAGCGGCGGCAGAGGCAAAGGCGATCGCCGCCGGGGCAAGGATGATGGTGAGGTGGACAAGAAAGAAGAACAGTTCAAGGAGCAAGGCGAGGAACCCAAAGAGGAAACCGGCGAGGAGGAGAAGGTGGTAGTTGAAGTGGTTGTAGACCAAGGAGGAGACGGCACCAAGGAGATCAAATATAGGACTCAACAAGGAGAGGAGATGGAGGACGACAAGCAATCGCCAGCGGATGCCCACGGCGACGGGGAGAGCGATGGGGCCGAAGCTCGTGCGCAGGACAAGCACGTGGTAGAGGCCGCCGGCAACGGCGACGGCGGGGACGATAGCTACACCACCATGGTGCAAGACGAGGTAAGTGCGATGCAGGAGGAGATGGAGAAGATGAAGGAGGAGAACCGGATGCTCCGGCGAGTCGTCGACCGAACCGTGCGTGACTACTACGAGCTGCAGAAGAAGGTAGAAGCCTGCTATCAGCAGCAACAGGCAGATGAGCCTAAGGAGCCCGAGGTGTTCCTTTCCCTCGGTGCCACCGCCGCCGGGACTGGCGGCGCCTTCCCGGAGCCGAAGCGGAAGGAGCGGCAAGCAGCGCGGCGGCCGTCTGTGGGGAGCGATGACACCGACGACGACGACGCCAAGGAGGACCTTGGGCTGTCCCTTAGCCTGAGAGCGTCGTCGTACGAGGAAGAGAAGCTAGAAGCGGGGCACGACGACGTGGAAGGTGCCTCCGTGGTCGGCGCCGACGACGGCAAGGCGAAGGGCTACACGCTGCTGGAGAGTAGCAAGCTCGGGGCACCGGCGGCCGGGATCACGAGCCAGAGTGTCAACCCGGCCAACCGCAAAACTAGGGTTTCCGTGCGCGTCCGATGCCAAGGCCCCACTATGAACGACGGGTGCCAGTGGAGAAAGTACGGGCAGAAGGTCGCCAAGGGCAACCCGTGCCCGAGAGCCTACTACCGGTGCACCGTCGCGCCGGGCTGCCCGGTGCGCAAGCAGGTGCAACGATGCCTAGAGGACATGTCGATCCTGGTGACGACGTACGAGGGTACGCACAACCACCCGCTCCCCGTCGGCGCCACCGCCATGGCCTCCACCACCTCCGCCGCGGCCACATTCATGCTGCTCTCCAGCACGACCTCTTCCTCCTCCATCTCCGAGGCCGGCGGCGGCTCAGCGGCGCCTCCCTACCTCAGCACTCCATACCTGCTCAACTCCACCTCCCACCACTCCGCCGCTTCGCCGCTGCTCAGCGCACCACCGTCGTCGTCAATGCCCAGCAGTACCCCTGGCGCGGCCAGTGGCGTGCAGCATCTCAACATGTTCGGGCATTCGTCGTCCATGCTAGCTCAGCAGGCGCCACATTTCGGCAGCAACAGCAAGTACCCATGGTCATCGGATCCTTTGCAGGGCATGGGTGGTGGTGGTGGTCTGCCAGCAGGGAGCAAGAGGCCGTTTTGGAGCACCGGCGGCGACGAGAAGACGGCGACATTGCCGGACAATGTCGGCGCAGTCATGGCAGACCCAAGTAAGTTCTCCGTGGCGATCGCGGCCGCGATCAACAGCTATATGGGGAAGGACGGGCAGGTGGTGGGCGGCAAGGACGGGGAGAGTAGCAGCAGCAAGAGTAGTAA

CAAGTGGGGGGTGGTCGAATCACTTCCACCTCCATGA

>SbWRKY3

ATGGACGACCGCCGCGGGCGCCGCGACGCCATGGGCCAGAGGCCGTTCGCGTCTGCTGCTCAGGGACAGGAAAGGGTGTTCGACGGTGGAGGCGGTGGCGGTGGCGGCGGCCCTGGGCCAGCGTTTGGCGGTGAGTTTGATCAGGGATCGTCGTCCCTCATGGCTCTTCTCGGAGCTGGTGGTGCAGTCAGCTCCCAGCCACCGCCGCCGACGTGGGGCGTCGAGGAGGTGACAGCGGCGCCTGCCATTAACCTGGTGCCTCAATCATTATTCTCCATGGCGAACTACGCGCCACCGCCGCCGTCCTACCAGCAACCCACCTCGTTCGCCCCATCGCCGCTGGGCGGCAGAGTGGATCCATACCCGCCGTACCTTGTCGCGGACCAGCCGCCGCAATGGCCTCCTCCCCGACCGGCGGCTGCTGATTCCTCCATGCCGCACTCCAACTTCACCGTCTTCTTCCCCAGGAATCCATACGACCATGACATGCAGCTGCGAGC

GACCGCGCTCTTCGGCGGCAGCAGCGGCTTGCACGCGCACGCCCTGCCGCCGCCGCCGCCAGCCATCGAGCAGCCGGCGAAGGACGGTTACAGCTGGCGCAAGTACGGGCAGAAGCAGCTCAAGGACGCCGAGTCGCCGCGGAGCTACTACAAGTGCACCCGCGACGGGTGCCCCGTCAAGAAGGTCGTGGAGCGCTCCTTCGACGGGTTCATCAAGGAGATCACCTACAAGGGCCGCCACAACCACCCGCGCCCCCAGGAGCGCGGCCTCGCCGGCGGCGGGAACGATGCCCTCGCCGCCGCCGAGGAGGACGTGGACGGCCCCAGCGACGACGACGACGATGACGTCGACGGGGCTCCCGGCCGGGCCGCGGACGGCGTTGTGGCCGGGCAGAGGGTGGTGAAGAAGCCCAAGATCATCCTCCAAACGCCGAGCGAGGTGGATCTCCTGGACGACGGCTACCGGTGGCGCAAGTACGGGCAGAAGGTGGTCAAGGGCAACCACCGGCCAAGGAGCTACTACAAGTGCATCGCCGACAAGTGCAACGTGCGCAAGCAGATCGAGAGGGCGTCCACCGACCCCAGGTGCGTCCTGACGACGTACACCGGCCGCCACAACCACGACCCACCGGGCCAAGGCAACGAAGCCGCCGCCGCCACCGTCGCCGCAGGCGGCTCTTCTGCTGATCCGGGCCCTCCGTCCAGGAACACGGCTAGTGGAAGTGGGGCGTTTCAGGAGAACTGGGGGGCTCGGCAGCTGAAGGAGGAGTGCTAG

>SbWRKY9

ATGGCGGCCCGCGAGGCCTCCGCCGCGGCGCCGGCCCCGGGCCCCGCAGGCGACGGGCCGAGCCGCCCGCCGCGGCCCACGCTCGCCCTGCCGCCGCGCTCCGCCGTCGAGTCCCTCTTCGCCTCCTCCGGCGCCTCCTCTGCCGGCGCCGCCGCCGAGACCAGCCCCGGCCCGCTCACCCTCGCCGCCGCGCTCTTCCCCGATGGCGCGCCCTCCCCGGCTTTCCACGGCTCCTTCACCCAGCTCCTCGTCGGCGCCATAGGCTCCCCGGCCGCCGTACCCTCGCCGCCCTCTCCGTTCGCCGTCCCGCCGGGGCTCAGCCCGGCCACGCTCCTTGGCTCCCCCGGCCTCTTCTCTCCCACGGGGAGTTTTGAGATGTCTCATCAACAAGCCTTAGCACAAGTAACAGCACAAGCAGTCCATTCTCAGTACAATATGATAAATCACGCAGATTACGCTATCCCTTTTTCGTCTACAACAACACCAGCTTTGATCACAGCACAGCATGCCAATTCTTCTGCCAATGTGACATCAGCACAGGAGAAACCAGCTCTGCCGTCACATACAGGTAATAGCAAAATTGAATCAAATGAGGTTTCACAAGGACTCAAACCTTCTGCACCCACTTTTGATAAACCTGCTGACGATGGGTACAACTGGCGGAAGTATGGCCAGAAGGCAGTTAAGGGTGGCGAGTATCCGAGGAGTTACTACAAATGTACCCACGCGAGTTGTCCAGTTAAGAAAAAAGTGGAGCGCTCAGCAGAAGGATACATCACTCAAATAATTTATAGAGGTCAGCACAACCACCAGCGACCTCCAAAAAGGAGATCCAAAGATGGTGGTGGTTTACTAAATGAAGCAGATGATTTCCATGAGAATGAAGACACTTCAACTAGATCAGAACCTGGTTCTCAAGATCACTCTGGAAAACATGAGGGGTCAAATGATGGCATAGCAGGGCCTTCAGTGTCAAGAAGGGGAGAGGGACATGAGCAATTGTCAGGCTCAAGTGATAGTGATGAGGAGCGAGATGATGAACAAAGGGCTGGCAATGGAGATCCTGGCTATGCAAATGCAAACAGAAGACATGTGCCAACTCCAGCTCAAAGGATAATTGTGCAAACAAACAGCGAGGTTGATCTTCTGGATGATGGCTATCGATGGCGCAAGTATGGACAGAAAGTGGTAAAAGGGAATCCCCATCCGAGGAGTTACTACAAATGCACCTATCAAGGGTGTGATGTGAAGAAGCATATTGAAAGATCATCCCAAGACCCAAAGGCCGTCATAACAACATACGAAGGGAAGCACAGCCATGATGTTCCAGCAGCCAGGAACAGTAGCCATGCTGCTGCCAATGCAAATTGTTCATCTTCCACCAGTGTACCACACAGGGTCCAGAGTTCAGCGTCCAGTAGTCGCAGAGTGGCAGACTTACAAAGCACATCCTCAGCTTCTTCTATGCTACTAAAAGAGGAAAACGAAATAACATAA

>SbWRKY73

ATGTCCGCGCGCCCGCCGCCGCCGCCGCGCCCGCGCCTGGCGCTGCCACCGCGCTCGGCGGCGGAGTCGCTCTTCACGGGCGCCGGCGACGCCAGCCCGGGCCCGCTCACGCTCGCCTCAGCGCTCTTCCCCTCCTCTGACAGCGACGGCGGCGGAGGCGGAGGCGGAGGCGCCAACTCCTCCTCCGGGGCCGCGACGACCTTCACGCAGCTCCTCACCGGCTCCCTCGCGCCGCCTCCGCAGCAGCAGCATGAGGCGGAGAGGGGACGAGGAGGAGGCGGGGTCGCCAGGGCCGGCCCGGCGCTCTCGGTGGCTCCGCCGGCGTCGGCGTCCGCTGGCGCGTCCGTCTTCACCGTGCCCCCCGGCCTCAGCCCCTCCGGTTTGCTCGACTCCCCTGGGCTGCTCTTCTCGCCCGCCATGGGGGGTTTTGGAATGTCGCACCAGCAGGCTCTGGCTCAGGTGACGGCCCAAGCAACCCATTCTCCACTCAGAATGTTTGATCACCTTGAACAGCCATCTTTCTCCACAGCTGCTACAACGTCGGGAGCTCTACAGCATATAAATTCTGCAGCCAGTATGGCAGGAATTTCAGATATGACAATGGCAACAGCAAACAATGAGAATGCATCATTTCAGTCTGCTGAGGCATCTCAGAGGTATCAAGTTAATGCCCCTGTTGATAAGCCTGCTGATGATGGCTATAACTGGCGGAAATATGGTCAGAAGGTGGTAAAGGGCAGTGATTGTCCAAGAAGCTATTACAAATGTACTCATCCCAGTTGTCCGGTCAAGAAAAAGGTAGAGCACGCAGAGGATGGTCAGATATCTGAGATCATATACAAAGGCAAACACAATCACCAACGTCCACCAAATAAGCGGGCAAAAGACGGCAACTCTTCAGCAGCTGATCAAAATGAACAATCTAATGACACCACATCTGGCTTGTCAGGTGCCAAGAGAGATCAGGACAATATATATGGGATGTCTGAGCAAGCATCTGGTTTAAGTGATGGAGATGATATGGATGATGGTGAATCAAGGCCACGCGAAGCGGATGATGCTGATAATGAGAGCAAAAGAAGGAATATACAAATTTCTTCACAAAGGACCTTGTCAGAGCCTAAGATTATTGTTCAAACAACCAGTGAGGTTGATCTTTTGGATGATGGTTATAGATGGCGCAAGTATGGACAGAAGGTAGTCAAAGGGAATCCTCATCCAAGGAGTTACTACAAATGCACATTTGCTGGATGCAATGTCAGGAAGCACATTGAGAGGGCTTCGTCAGACCCTAAGGCTGTCATAACAACCTATGAAGGAAAACATAACCATGAACCACCGGTTGGTCGGGGCAACAACCAGAATGCAGGAATATCACAACAGAGAGGGCAGAACAACATATCTAGTAATCAAGCTTCACTTCCGAGACCAGACTTCAGCAACACTAACCAGATGCCGCTAGGGATCTTGCAGTTCAAGAGCGAGCAATAG

>SbWRKY19

ATGGCCGGCGCAAGTAACCATGGATCCCTCATGGACGAATGGTTGCCGCCCCCTACACCAAGCCCAAGAACACTCATGTCAAGCTTCCTGAATGAAGAATTCAGCTCCGAGCCATTCTCTGGTTTTTTCAGTGAACATGGCACCAACAAGCCCCATGATCAATCCGAAAAGAGCAGAGAAGTTGTGAATTCGAGCGAGGAGGTCCCTGCTCATGCTGTCAATGATCCATTTCAAAAGGGTTTCTCCCTGAAGCCAAATTTGTTCAGTGCTAATCATAAATCAAACTCCAATGGTGGTTTGGCGGAGCGCAGGGCTGCGAGAGCAGGTTTCAGCGTCGCAAAAATTGATACTTCTCGAGTTGGTTCATCAGCAGTTATTCGATCTCCTGTGTCAATTCCACCAGGTCTAAGTCCAACTACACTTCTTGAGTCTCCTGTGTTTCTCTACAATAAAATGGCACAACCTTCTCCAACCACTGGCACGTTGCCATTTTTGATGGCTACGAATGATAAGTCAACAATACCACCAGCTGCCAAGATAACTGAAGATTCTCCATTTGATAATGATGTGTTTTCTTTCCAACCCCACTTAGGTTCTGAAGCAACAGGTTTCTCTACTGCAGAAAAGGACTATGGCGCCTATCAGCAAAAGCAGTCCTTGTCGAATATTCATCAGCAGGAATCCAGTCTTCAGTCAAGCTTTACAGCAGTCAAGGATAACACTAGTGCAACAATTGTTAAAGCGAAGACGTCTAGCTCCATGTTCAGTGATAGTCACTATTCAGCTGACCAACAGCAAGCTGACGAGACAAATATAAAGGTGCAAGGCAAAGGTGTCGAGGCTAGATCAGCTGCTTTTCTTCCTGTATCAGCGCATAGTGATGCATCTCTCTTGGAGTCTCAAGATGCAGTTGATGTCTCGTCAACACTGTCTAATGAAGAGGAGAGGGCAACGCATGGTACTGTTTCTATAGAGTGTGATGGTGATGAAGATGAGACTGAATCCAAAAGAAGGAAGCTGGAACTAGATGCTTTAGGAGCTACTGCTATCACTACTACCTCCACCACCAGTACCATTGACATGGGGCCTGGAGCCTCAAGAGCTGTCCGGGAGCCTAGGGTTGTTGTTCAGACCACAAGTGAGGTAGACATTCTTGATGACGGTTATCGGTGGCGCAAGTATGGGCAGAAGGTTGTTAAGGGCAATCCAAATCCAAGGAGCTACTACAAATGTACACACCCAGGATGTTCAGTGCGCAAGCATGTGGAAAGAGCATCACATGATCTGAAATCAGTCATCACAACATATGAGGGAAAGCACAACCATGAAGTTCCAGCAGCCAGAAATAGTGGGCAAGGCAGTTCTGGTTCCGGCAGTGCTCCATCTGCACCACAAGCTGGTGGTTCTCACCGTAGGCAAGAATCAGCACAAGCCAGCTTTGCTCACTTCGGCACGACCAGTCCTTTCGGCTCCTTCGGTCTCCCACCGAGCAGACAGTTGGGACCAACAACTGGCAATTTCCGCTTCGGGATGGTTCCGCCGGGCATGACGATCCCAATGCCCTCTCTAGGATCACTTGCCCCTACAAAGATGGTAGGAAGTTCATCAAGCATGCAGGGGTACCCAGGGCTTATGATGCCAGGAGAGCCCAAGGTGGAGCCTGTCTCGCAACCCCTCTTCCCAATGGCAAATGCATCTCC

ACCAGCTTACCAACAGATGCTGAGCAGGCCTCCTTTTGGTCATCAGATGTAA

>SbWRKY67

ATGGAGGATTGGATGCTTCCCTCACCCAGCCCAAGAACACTGATGCCAAGCTTCTTCAACGAAGAATTCAGCTCTGCTCCCTTCTCCAACATCTTCAGTGATGATAGAAGCAACAAACCCCTGGATGAAATCGAGAAGAGTAAAACTTTCATTGGCTCGAGTGCTCAAGAAACCTCTCAAGATACGAAAGACCATCCACAGACTGAATCAAACCTTTTCAGCGCAAATCAGAAATCAACCTCTCCAGGAGGTCTTGCAGAGAGGATGGCCGCAAGAGCGGGTTTTGGTGTCCTGAAAATTGACACGTCCCGTGTCAGTTCATCTGGTGCACCAATCCGGTCTCCGGTGACAATTCCACCTGGTGTGAGCCCAAGGGAACTTCTTGAGTCACCGGTTTTCCTTCCAAATGCCACTTCGCAACCTTCTCCTACCACTGGTAAATTGCCATTCCTGATGCCTAATAATTTTAAATCAACAATGCCGTCCGTCCCCGAGAAGTCTGAAGATCACTCACATGAAGATTCTGCGTTTTCCTTCCAGCCCATTTTGAGGTCTAAACCATCAACCTTATGGACTGCAGAGAAGGGATCAAGTGTTGTTCACCAAACCCAGTCCTTAACGAAGGATAGTCAGGGGTTAAATGTTCATGCTAACCCAACTGCGACCAAGCATGAAACTGAGGAAAATCTAGTTAAACCTAAGACATGTGATTCCATGTTTGATGAACAGAGCGAGGAAATCCAAAATGGGGAAGATTCTTCAGCTCCAGACACTGGCACCGCCGATGATGGATATTTTTTGAGGGTAAACAGGAGGGGCATGCCCCTACTGGATGATGGATATAACTGGAGAAAATATGGAGAAAAGCAAGTGAAAAAAAGTGAACACCCAAGGAGCTACTACAAATGCACTCACCCAAAATGTCCTGTCAAGAAAATGGTGGAGCGTTCTCTAGAAGGTCATATAACGGAGATAGTCTACAGAGGTTCTCATAGTCACCCACTGCCTCTTCCCAACAGCCGGCCAAGTGTCCCTTTGTCGCATTTCAATGATTCAGAAGCTGATGGTAACTTCAGTTCCAAGCCTGGGCCTGGCTATGACTCGTCAACTTCACAGGGAATTGCTCCAAAAGGTCAGTTCCAAGATGTGCACAGCGGAGCTCTTGAAACAAAACTGTCTGGCTCTCTTACTACAACTGAGATTGCTGACACGTCTGTTATGGAGTCTATGGATGTCTCATCAACACTCTCCTCCAATGAGAAGGGTGATAGGGCCATGAATGGTGCCGTTCCTTCAACCAATGACATGAACGAAGACGAGACTGAATCTAAAAGAAGGAAAATGGAGGTTTCTGTCGCTAGTAACACTGCCAACATTGTCACTGATATGGCAGCTATGGCATCAAGGACTGCCCGGGAGCCTCGGATTGTTGTGCAAACAACAAGTGAGGTCGACATCCTTGACGATGGCTACCGCTGGCGCAAGTATGGACAGAAAGTTGTGAAAGGCAACCCAAACCCAAGGAGCTACTACAAATGCACTTATGCTGGCTGCTCAGTGCGCAAACATGTGGAGAGGGCCTCAAATGATCTCAAGTCCGTGATCACAACATATGAGGGGAGACACAACCATGAAGTTCCAGCTGCCAGGAACAGCAATGGGCATCCGAGCTATGGCTCCAGTGCTGCACCACAGGGTAG

CAGTCTTCACCGGAGGCCAGAGCCTCCACAATTCAGCATGCCTCACGCTGCTGCCGCCGCTGCCTACGGTTCACTTTGTCTCCCACCACAACTCAATGCAGCTTCAGGGGGTTTCTCCTTCGGAATGCTCCCCCCTGCAATGGCAATGGCAATTCCAGTACCATCTCTGGGTAACTTCATGCCAGCGCAGATGCCTGGCCATGGATCACCAATGCAGGGTTGTTCAGGCCTTATGCTGCCGAGAGGTGAGGAGAAGGTGAACCCAGAGCAGCAGTCCAGATTGCCGGTAGCGAATGGGAATGCGGCAGCAACTTACCAGCAGCTCATGGGAAGGTGGCCTCAGGGTCATCAGATGTAA

>SbWRKY59

ATGGAAGGCCATGTAGCAATGGAGTGGAAGGATCCTAAACCAGGCCCAGAATCCTTGATGGGCTTCCAAACCAGAGGTGTTCGACCAGACACCGTAGGGGGGCACAGCAACGAGGACGCTAAACCCGGATTTGAGAAGCATGGCTTCTCAGTCGACATAAGCTCGCCTCAGGAGGAAGGTCGGTCACTTCCACTCACCCCGCAGTTTGGCCAGAAGACCAGTCCTGGCAGCAGCCTCGCCGAGAGGATGCAGGCAAGAGCTGGATTCAAGGTGCCGAAGCTCAACATGCCTTTCAGCACAGCAGCTGGAGCTGATAATTCAGTACCAGGAGCCCCATCGCCCTACCTCACGATCCCGCCTGGCTTGAGCCCAGCAACGCTGCTCGAGTCACCAGTCTTCGTTTCTAATGCCATGGGCCAACCCTCGCCAACCACGGGGAAGCTATTCATGTCTGGTAGCACAAATGACAATGATCCTATTAGATTTGGAGGTCCTCCAGTTGGAGATGGCCCTGATGCTTTTTCTTTCAAGCCCTTAGATCTGAAATCCTCACACTACACTGCTGAAGCAATGAAGGAACAAAATACGCAGGTTTCTGTCAAGACTAAAACTAAGACTCAGCCAGTACAAGAAGCTAATTTGCTGGGCCAACTGAACCAGCAGAATCACAATGTGCAGACCAATATGAACATCGGCGGTCCTCATGACTCCAAGCTCAGCCGTCTTGCATCTGGCACCGGTGCCTGCAACGAACACGTATCACCCCCGGATTACGGCCAGACAGCTGAGGAAGGTGGTGATGCGAGGGAAGACTACCCGCCGGCAATGGCCGCCGCTACAGCGCCAGCAGAGGACGGGTACAGCTGGAGGAAGTACGGGCAGAAGCAGGTGAAGCACAGCGAGTACCCGCGGAGCTACTTCAAGTGCACGCATCCCAACTGCCAGGTGAAGAAGAAGGTGGAGCGCTCGCACGAGGGGCACATCACGGAGATCATCTACAAGGGCGCGCACAACCACCCGAAGCCCACGCCGAGCCGCCGGCCAGGCGTCCAGGTCCAGCCCGTGCACCCGTTTGGCGACGCCGGCGGTGCGCAGCAGGC

GGACGCCGCGGCCGACAACAACCTGGGGTCACAGTCACAGCAGGCAAACGCTGCAGCGGAAGCCAACCACCAGCCGTGGCGTGCGGGCGTTCAGGATGGCATGGACGCCGCGACGTCGTCGCCCTCGGTTCCCGGTGAGCTCTGTGACTCGTCGGCGTCGATGCAGCAGGTCGAGTACGCACCCCGAGGGTTTGGGTCCCCCGAGGGCGCGGATGTGACGTCTGCTCCGTCTGACGAGGTGGACGGCGGCGACAGGGTGACTCTTGGCAGCATGTCCCACGCCGGTGCCGACGCGGAGGGCGACGAACTGGAATCTAAACGAAGGAAGGTGGAGGCATATGCCATGGATATGAGCACTGCGTCGAGAGCTATTCGCGAGCCTCGCGTGGTGATCCAGACGACGAGCGAGGTGGACATCCTCGACGACGGCTACCGCTGGCGCAAGTACGGGCAGAAGGTCGTCAAGGGCAACCCGAATCCAAGGAGCTACTACAAGTGCACGCACCCGGGGTGCACGGTGCGGAAGCACGTGGAGCGCGCGTCGCACGACCTCAAGTCCGTGATCACCACGTACGAGGGCAAGCACAACCACGAGGTCCCCGCGGCGAGGAACAGCGGCGGCCACCCGAGCACCGCGGCGGCGGCGACAGGCGCGGGTGGTGGTGGTCCGCGGAGGCCGGAGCACACGTCGTCGGTGCACGACGGCCTGATGATGAGGCACCTGGGCGGCTGCGGCGTGCCGTTCGGCCTGCCGCTGCAGCCGCCTAGCAGGGACCCGCTGGCGCCGATGGGCAACTACCCGACGTACCCGTTCACTGCCCTCGGCGGCGGCGGTGGCAGCGGCGGCGACGGCGGCCTCACGTCGCTGCCGAGCTTGCCGATGGCCACGGGGAACCTCAGCGCCGTGGAGGGGCTGAAGCTCCCGATGCTGGCAACCTCGTCGCCCCTGCACCACCAGCACCCGCTGCTGAGGCACCGGCAGGCGATGCAGGCCGCCGCCCTCGCCGCGGCGCCCATGGCGCAAGTGAAGGTCGAAGACAACGTCGCCGCCGGCGTTACCGCTGCCCCGTCGGTGTACCAGCAGATGGTGCGCAGCGGGCTGCGGCTGGGCCACCAGATGTAG

>SbWRKY20

ATGGCCGATTCGCCAAACCCTAGCTCCGGGGACCTCCCCGCCGGCGCTGGGGGCTCGACGGAGAAGCCGGTCCTGGCGGATCGACGTGTGGCTGCGCTCGCCGGCGCCGGCGCCAGGTACAAGGCCATGTCCCCCGCTCGGCTGCCGATCTCGCGGGAGCCGTGCCTCACCATCCCCGCCGGCTTCAGCCCCGGCGCTCTCCTCGAGTCCCCCGTCCTCCTCAACAACTTCAAGGTTGAACCGTCTCCCACAACTGGTACTCTGAGCATGGCTGCAATCATCAACAAGAGCACTCATCGAGACATACTGCCTTCGCCTAGGGATAATTCAGCTGGTAGTGGCCAGGAAGATGGAGGCTCTCGGGATTTTGAATTCAAGCCTCATCTCAATTCTCAATTGGCGGCTCCTGCGGTCAACAATCAAAATCGTCATGACACCCCTATGCAAAATCATAGCTCAAATCATGCCTCACCATCTAGCAATTTGATGACTGAAAATAAACCTCTCTGCTCACGGGAATCGAGTCACACAGCAAATGTTTCAAGTGCTCCAAACCAACCTGTTTCAATAGTTTGTCCATCTGACAATATGCCTGCTGAAGTTGGTACATCAGAGATGCACCAGATAAATAGCTCTGAAAATGCTGCCCAGGAGGCACAAACTGAAAATGTAGCTGAAAAATCAGCAGAGGATGGCTATAATTGGCGCAAATATGGACAGAAGCATGTCAAGGGAAGTGAAAATCCTAGAAGTTATTACAAGTGCACGCATCCTAATTGTGAAGTTAAAAAGCTATTAGAGCGTTCGCTTGATGGTCAGATTACTGAAGTTGTTTATAAAGGGCGTCATAATCATCCTAAGCCCCAACCGAATAGAAGGTTAGCTGCTGGTGCAGTTCCTTCAAGCCAAGGTGAAGAAAGATATGATGGTGTGGCACCTATTGAAGACAAGCCTTCAAATATTTATTCCAACCTCTGTAACCAAGTACATTCAGCTGGCATGATTGATACTGTTCCGGGTCCAGCTAGTGATGATGATGTTGATGCTGGAGGTGGAAGGCCCTACCCTGGGGATGACGCTAACGATGATGATGATTTGGACTCGAAACGCAGGAAAATGGAATCTGCTGGTATCGACGCTGCTTTGATGGGTAAACCGAATCGCGAGCCCCGTGTTGTTGTACAAACTGTTAGTGAAGTTGATATCTTGGATGATGGGTATCGCTGGCGCAAATATGGGCAGAAAGTAGTGAAAGGAAACCCCAACCCACGGAGTTACTACAAATGCACACATACAGGATGCCCGGTCAGGAAGCATGTTGAGAGAGCATCACATGACCCGAAGTCAGTGATCACAACATATGAAGGAAAACATAACCATGAAGTCCCTGCTTCCAGGAATGCAAGCCATGAGATGTCCACAGCTCCCATGAAGCCTGTGGTGCATCCTATTAACAGCAACATGCCAGGCCTTGGTGGCATGATGAGAGCATGTGATGCCAGGGCCTTCACCAATCAATATTCTCAGGCAGCTGAAAGTGACACCATCAGCCTTGACCTTGGTGTAGGTATCAGCCCTAACCACAGCGATGCAACAAACCAAATGCAGCCCTCAGTTCCAGAACCTATGCAGTATCAAATGCAACACATGGCTCCTGTTTATGGTAGCATGGGACTTCCAGGAATGCCTGTGGCAGCAGT

ACCTGGAAATGCAGCTAGCAGTATTTACGGTTCCAGAGACGAAAAAGGAAATGAAGGGTTTACTTTCAAAGCCACACCTTTGGACCGATCAGCTAACTTTTACAGTAGTGCTGGTAACTTAGTGATGGGTCCATGA

>SbWRKY42

ATGACCACCTCGTCGTCAGGGAGCATCGAAGCACCGGCGGCGAGCTCGAGGCCCGGCTCGTTCTCGTTCGCGAGCACGAGCTTCACGGACATGCTGGGAGGATCCGCGGATGCGGCGGCCGGCGGGGCGTCGAGGTACAAGGCCATGACCCCGCCGTCCCTGCCGCTGACGCCGTCGTCCTTCTTCAGCAACATCCCCGGCGGCCTCAACCCCGCCGACTTCCTCGACTCGCCGGCCCTCTTGAGCTCCAGTATCTTCCCCTCGCCGACGACGAACGCATTCGCGTCGCAGCAGTTCAGCTGGCTGACGACGCCGGGCGCGGAGCAAGGCGTCAAGGAGGAGCAGAGGCAGTCCTACCCGGACTTCTCGTTCCAGACAGCGCCGACGACCCAAGAGGCCGTGCGGACGACGACGACCTTCCAGCCACCGATTCCAGCGGCCCCACTGGGTGAAGAGGCGTATAGAAGTCAGCAGCAGCAGCAGCAGCCATGGGGCTACCAGCAGCAGCAGCAGCAGCCTGCAGGCATGGACGCGGGATCCAGCCAGGCTGCCTACGGCGGGGCGTTCCAGGCAGGCTCGTCGGACGCCGGCGCGATGGCGCCGCACGTGCCGGCGAGCGGCGGGTACAGCCACCAGGCGCAGAGGCGGTCGTCGGACGACGGGTACAACTGGCGCAAGTACGGGCAGAAGCAGGTGAAGGGGAGCGAGAACCCGCGCAGCTACTACAAGTGCACCTTCCCGAGCTGCCCCACCAAGAAGAAGGTGGAGCGGTCGCTGGACGGCCAGATCACCGAGATCGTGTACAAGGGCACGCACAACCACGCCAAGCCGCAGAACACGCGCAGGAACTCCGGCGCCGCCGCGCAGCTGCTGCAGGGCGGCGACGCGTCCGAGCACTCGTTCGGCGGCACGCCCGTCGCCACGCCCGAGAACTCCTCGGCGTCGTTCGGGGACGACGAGGTCGGCGTGGGCTCGCCGCGCGCCGCCAACGCCGCCGGCGACGAGTTCGACGAGGACGAGCCGGATTCCAAGAGATGGAGGAAAGACGGTGACGGCGAGGGTATCTCCATGGCCGGCAACCGGACGGTGCGTGAGCCGAGGGTCGTTGTCCAGACCATGAGCGACATCGACATCCTCGATGACGGCTATCGGTGGAGGAAGTACGGGCAGAAGGTGGTGAAGGGAAATCCAAACCCAAGGAGCTACTACAAGTGCACGACGGCCGGGTGCCCGGTGCGCAAGCATGTGGAGCGCGCGTCGCACGACCTGCGCGCCGTGATCACCACGTACGAGGGCAAGCACAACCACGACGTGCCCGCGGCGCGTGGCAGCGCCGCACTCTACCGGCCCGCGCCTCCGCCGCCGCCGTCCGCGGACAACGCCGGCCACTACCTCGCGGCGCAGCCGGGCATGGCGTACCAGACGGGGCAGCAGCAGTACGGGTTCGGCGGTCAGGGCTCGTTCGGCCTCAGCGGCGGCGCCGGCGCGCCGGCGCAGAGCAGCGGCAGCTTCGCGTTCTCCTCCGCCGGGTTCGACAACCCGATGGGTTCGTACATGAGCCAGCACCAGCAGCAGCAGAGGCAGAACGACGCCATGCACGCGTCGAGGGCCAAGGAGGAGCCCCGTGACGACATGTCGTTTTTCCCGCAGTCGATGCTCTACACTGACTGA

>SbWRKY80

ATGGTCAAACTTTCGAACGTCTATATAGGCCGCAGACGGGTCGGTCTCGACTCTCGACATCACTTCACCATCCTATACGCCGTCGCCCGCCCGGCTGCTTCTCGTTCCATTCGTCTTCGTCTTGCCTCCCTCCGGCAGCCTCCGACGACCCTCCTCTCCTCGCCTGCGATGACCTCGACGCCGGGGAGCTTCGGCGGAACGCTGGCGGCTAACTCTGGGCCGGTCGCGCTCTCGTTCCCGACCACCTCCTTCGCCAACTTCCTAGGCGGCGGTGGCTCCTCAGCTTCTAGCAGCGGAGCGGCGGACAACGGGGGAGTCGGGCTGTCCAAGTTCAAGGCCATGACCCCGCCTTCCCTCCCGCTGTCGTCGTCGCACCCGCCGGCGTCGCCGGCGTCGTACCTCCACGCCTTCTCCGGCATCCTCGACTCGCCGATCCTCCTCACTCCCAGCCTATTCCCGTCGCCGACGACGGGCGCGATCCCGTCAGAGCCCTTCAACTGGATGGGGACGTCGGAGAGCCTAAGCGGCAGCGTAAAGACCGAGCAGCAGCAGTACACCGACTTCACGTTCCAGACGGCGGCGTCCGCACCGCCGGCGACGTCGACGTCGACGATGACCGGTGCCTCGCACTCGGCGTCCTATCTGCAGTCATCAGTGCTGATGGCGCCGTTGGGACGAGTAGGAGACTCGTACAACGGCGGCGAGTTGCAGCAGCAGCAGCAGCAGCCGCCATGGGCCTACCAGGAACCGTGTACGCAATTCGAGGCGCCGGCGGCGGCGCAGCCTGACAACAGCATGCTCGGGAACGGCGGCTACGGCGGGGCTCCCGGGCCGGCGGTATCCGGCTGCTTCCGCGAGCAGAGCCAGAGCAACCGGCCGTCGTCGGACGACGGGTACAACTGGCGCAAGTACGGGCAGAAGAATATGAAGGGGAGCGAGAACCCGCGCAGCTACTACAAGTGCAGCTTCCCGGGCTGCCCCACCAAGAAGAAGGTGGAGCGGTCGCCGGACGGGCAGGTCACGGAGATCGTGTACAAGGGCGCGCACAACCACCCGAAGCCGCAGAGCACCCGCCGGAGCGCAAGCTCGGCGCCGGCGCCGGCGTCGCACGTGCTGCAGAGCGTCGGCGACGCCGTGCCCGAGCATTCCTTCGGCGCGCTGTCCGGCACACCCGTGGCGACGCCCGAGAACTCGTCGGGGTCATTCGGCGGCGACGACGAGATCAACGGCGTCAGCTCGCGGCTGGCCGGTAACTTCGCCGGCGCCGACGATCTCGACGACGATGAACCCGACTCCAAGAGATGGAGGAAAGATGGTGGCGACGGCGACGGAGGGGTCTCGTTGTCCGGCAACAACCGGACGGTGCGGGAGCCGAGGGTCGTCGTGCAAACGATGAGCGACATCGACGTCCTCGACGACGGCTACCGGTGGCGCAAGTACGGGCAGAAGGTCGTCAAGGGCAACCCGAACCCGAGGAGCTACTACAAGTGCACGACTGCAGGGTGCCCAGTGCGGAAGCACGTGGAGCGTGCGTGCCACGACACGCGCGCGGTGGTCACCACGTACGAGGGCAAGCACAACCACGACGTGCCGCCGGCGCGCGGCAGCAGCGCCTCGCTCTACCACCGCGCCGCGCTGGCGGCGCATCAGATGCCGCAGCAGGCCGGCGGCGGGAGCTGCTACCAGCAGCAGCAGCAGCA

TGGCGGCCTCGTCCGGACCGCCGATGGGTTCGGCTTCGGGGCCAGCGGCGGCCTGCACGGCGGCGCGCCGATGATGCAGGCCGCGGAGAGCGGCTTCGCCTTGTCCGGGTTCGGCCACCCGGCGGGCACGGCGGCGTACTCTTACACGAGCCACCAGCAGCAGCAGACGACGACGACGAACGAGGCGATGTACTACGCCAAGGACGAGCCACGAGACGACATGTTTTTTGAGCAGCCGCTCCTGTTCTGA

>SbWRKY79

ATGGCGTCCTCGACGGGGAGCTTGGAGCACGGCGGGTTCACGTTCACGCCGCCGCCCTTCATCACCTCGTTCACGGAGCTACTCTCCGGGACAGGGGACATGCTAGGAGGAGCCGGCGGCGCCGACCAGGAGCGGTCGCCGAGGGGGCTGTTCCACCGCGGCGCCAGGGGCGGCGTGGGCGTGCCCAAGTTCAAGTCCGCGCAGCCGCCCAGCCTGCCCATCTCATCGCCGCCGCCGATGTCGCCGTCCTCCTACTTCGCCATCCCGGCCGGCCTCAGCCCCGCCGAGCTGCTCGACTCGCCCGTCCTGCTCCACTCTTCCTCCAACATCTTGGCGTCTCCCACCACCGGCGCCATCCCGGCGCAGAGGTTCGACTGGAAGCAGGCCGCCGATCTCATCGCGTCTCAGTCTCATCAGCAAGACGACACCCGGGCTGCCGCCGCCGCCGGCGGCTTCAACGACTTCTCCTTCCACACGGCCACCACCTCCAACGCCATGCCCGCGCAGACCACGTCCTTCCCTTCCTTCAAGCAGGAACAGCAGCAGCAAGTCGAAGCGGCAGCGACGACCAATAAGCAGAGCGCCGTCGTGGCGTCGAGCAACAACAAGCAGGGGAGCAGCGGCGGCGGGAACAGCAGCAACACGAAGCTGGAGGACGGTTACAACTGGCGCAAGTACGGGCAGAAGCAGGTGAAAGGGAGCGAGAACCCGCGCAGCTACTACAAGTGCACGTACCACAGCTGCTCCATGAAGAAGAAGGTGGAGCGGTCCCTGGCCGACGGCCGCATCACGCAGATCGTCTACAAGGGCGCGCACAACCACCCCAAGCCGCTGTCCACGCGCCGCAACTCGTCCTCCGGTGGGGTCGTCGCGGCGGGGGAGGAGCAGCAGGCCGCCGCAAACAGCCTCTCCGCCGCCGCCGCCGCGGCGGGTGGCTGTGGGCCGGAGCACTCCGGCGCCACCGCCGAGAACTCGTCCGTCACCTTCGGCGACGACGAGGCGGAGAACGCGTCGCACCGGAGCGACGGCGACGAGCCCGACGCCAAGCGCTGGAAGCAGGAGGATGGCGAGAACGAGGGCAGCTCTGGCGGCGCCGGCGGCAAGCCGGTGCGCGAGCCCCGGCTGGTGGTGCAGACGCTGAGCGACATCGACATCCTGGACGACGGGTTCCGGTGGCGCAAGTACGGGCAGAAAGTGGTGAAGGGGAACCCGAACCCGCGGAGCTACTACAAGTGCACCACGGTGGGTTGCCCCGTGCGGAAGCACGTGGAGCGCGCGTCCCACGACACGCGCGCCGTGATCACCACTTACGAGGGCAAGCACAACCACGACGTGCCCGTGGGCCGCGGCGCCGCGAGCCGCGCGGCGGCGGCGGCGGCGGTGGCGCCGACGATGGGAGCCTTGATGGCCGCCGGCGGCCATCAGCAGCAGCAGCAGCCCTACACGCTGGAGATGCTGAGCGGCGGAGTAGGCGGTGGCGCCGCATACGGCGGCGGCTACGCGGCCAAGGACGAGCCGCGGGACGACCTGTTCGTCGACTCGCTCCTGTGCTAG

>SbWRKY65

ATGGCCGACGGCGATCCGGCGCCGGCGCTGGCACTGGCCAACGAGAAGCTCCCTGCCCCGGCGGCGGCTGACGTGGATGAGACGCGCCCGGCGCCACCGCTGGAGCCCTCCCGGGGTCCGGACGAGGAGAAGCGCCCGCTCGAAGAGGAAGAGGCGGAGGTGGAGGCGCACCCGCCGCGGGAACCAACCGGGGCGCCGCCAGTAGATCGCTTGGGGATGGAGGTGGTGGCGGCGGCAGAAGCGGACATGAAGGCGAACGAAGTGGAGAAGGAGAGGGGGGATCGGGCGAAGGAGAAGCGGGAGAAGGATAAGGGGAAGGGGAAGGAGGGGAAGGAGAAGGAGAAGGTGGAGGAAGAGGCGAAGCTGAAAGTGACTGCGGTGGTGAAGGTGGAGGGGACGGAGAAGGAGGTCAAGGTGACCCGGCCGCCAGCTGGGGCCAGTGCGGAGACGCCCATCCTAGCGGTGCCGGTGGTGGCCGTGCCTTGCTTCATTGCGCCTCC

AGGGTTTGCGGGCCAGTTTGCAATGACTCATCAAGCAGCTTTGGCGAGTGTTACAGCACAGGCACAAATGCACTTGCAGTCACCAACTTCATCTGCATGTTCAGAAGTGCCATCAAGTCCATTTTATATGACACCAAGATCTCTAGTGCCACTTCAGCAATCACCATCAGTAACTGAAGGAAATATTTGTAAACCAATTGCTGACAAGTCATTTTCATCTGATTCAAAATCACACCATGTTGTTGTAAATATGGTAGCTGATGGCTTCAACTGGAGGAAATATGGTCAGAAGCAGGTGAAAAGTAGTGATAATTCCAGGAGCTACTATAGATGCACAAACTCAGGTTGTCTGGCTAAAAAGAAGGTTGAGCATTTCCCTGATGGCCGTGTTGTGGAAATCATTTATAGAGGAGCTCACAATCATGAACCACCACAAAAGACCAGATTTGCAAAAGAAAGAGTGACTCCAATTGGTGTTCCCTCTGGAGGCGAAACCTTGAGACTTGTGAACACAGAAATTGTAGAATCCAGTACCCCTACATGTAAATTGGAACAGAGTGCCATCTCAGAAACTTCTGAGCAGCATCTCTTTTGCTCAAGTGACTGTGAAGGGGATGCTGGTAACAAATCTGAAAATGAACATCCTAGTGCAGAACCCCTGCCAAAGCGAAGGACACTTGAGACCACAGCGCCTAACTTAACGCCAGTTCTCAGAACAGTTAGAGAGCAAAAGATTATTGTGCAGGCTGGGAAGATGAGTGATGGATACAGATGGCGCAAGTATGGGCAGAAAATCGTGAAGGGAAATCCAAACCCCAGGAGTTATTACCGCTGCACGCATGGTGGATGCCCTGTCCGTAAGCATGTGGAAAAGGCACCAGATGATGTTAACAATATTGTTGTAACTTATGAAGGTAAACACAATCACGATGAACCATTCAGAAGCAGCAGCATACCAGTTTCTGCAATCAGTCCGTCAGCAACAACCACTGAGCAACCCAACACATCAACTACATCAGATGAGAAACCTCCAACCATTACTCAGAAGGATGCCAACAGTGAGTCTGATAAGGAGACAACTTTGGAATTTGGAGGTGAGAAGGCACTTGAATCTGCACAAACACTGCTCAGCATCAAAACTAACTCCGATGACATGAAGAACTCTGTCCTGAAAGAAACTTCTGCTGCAGTACAAGTCCAAAACAGTTGA

>SbWRKY87

ATGGTTGTCCAGCTGAGTGACAGCCGCCGTGACGCCGACGCCGATGGTCAGATGGCCGGCGCGGCGGCCGTCACGCCGGCCAACTCATCGGTGCTGTCGTCATCCAGCTGCGAGGCGGGAGCGGATGCCAACGACGACGACGAGGAGCCGTCGCGCCGGCGGTGCGGCAAGAAAGGTAGGATCGAGGGAGAGGAGGAGCAGGAGGGAGAAGGAGAAGCTGACGATGATGCAGCCGATCGGAACTGCAAGAGCAGCAAGGAGAATAAGAAAAGGAGAGGCGAGAAGAAGGCGCGGGAGCCCCGTGTGGCGTTCATGACCAAGAGCGAGGTCGACCACCTCGAGGACGGCTACCGCTGGCGCAAGTACGGCCAGAAGGCCGTCAAGAACAGCACATACCCAAGGAGCTACTACCGGTGCACGACGGCGCGGTGCGGGGTGAAGAAGCGGGTGGAGCGGTCGCAGCAGGACCCGTCCACGGTGATCACCACGTACGAGGGGCAGCACACGCACCCGAGCCCCATCGACCTCCTCAGGAGAGGAGGAGGCGCCGCCGCCCTCATGCGCTCCGCCGCCGTTGCCGGCGGCTTTCGCCGCCCCGACGACCTGCTCAAGATCGACGATTACGCTGGCACGCCGATCGGCTTCCTGCCGCTTCTCCCTCCCGGTGGCATCGGTGCTGGTGGTGGTGGCCGCCTGCTGCATCACCGAGCTCGTTCTTCGCAGCTGGCCGCCGTGGACGCGTATGGCGGCATGCTGGAGCTGGACTTCATCCCTTCCATTCCGCGATGA

>SbWRKY88

ATGTCTGGAGGAGCAACAGGAGTTGGAGGAGGCAGCGGCTACGGGGGCTTCTACCACGGGGACGATCCTG

CCACCTCCGACCAGCTCATCACAGCCTTCGACAACGACGGCGGCGGCGGCTTCTTCTTCCAGCAGACGGT

CTCGCCGCCGTGCGCCGGGGAAGTGGACGGCGGCACGGCGCCGTACGCGAGCATCGCGGACTACCTGCAG

GGATTCCTGGACCCCGCGGGCCTGGCCGCGCACTTCGGCAGCGACGACGCGCCGCCGCCGTGCCGGTTGG

GCGGCGGCGCCGACGACGAGTACGACGCTGTGGTGGCGGTGAAGCAAGAAATGGTTGTCCAGCTGAGTGA

CAGCCGCCGTGACGCCGACGCCGATGGTCAGATGGCCGGCGCGGCGGCCGTCACGCCGGCCAACTCATCG

GTGCTGTCGTCATCCAGCTGCGAGGCGGGAGCGGATGCCAACGACGACGACGAGGAGCCGTCGCGCCGGC

GGTGCGGCAAGAAAGGTAGGATCGAGGGAGAGGAGGAGCAGGAGGGAGAAGGAGAAGCTGACGATGATGC

AGCCGATCGGAACTGCAAGAGCAGCAAGGAGAATAAGAAAAGGAGAGGCGAGAAGAAGGCGCGGGAGCCC

CGTGTGGCGTTCATGACCAAGAGCGAGGTCGACCACCTCGAGGACGGCTACCGCTGGCGCAAGTACGGCC

AGAAGGCCGTCAAGAACAGCACATACCCAAGGAGCTACTACCGGTGCACGACGGCGCGGTGCGGGGTGAA

GAAGCGGGTGGAGCGGTCGCAGCAGGACCCGTCCACGGTGATCACCACGTACGAGGGGCAGCACACGCAC

CCGAGCCCCATCGACCTCCTCAGGAGAGGAGGAGGCGCCGCCGCCCTCATGCGCTCCGCCGCCGTTGCCG

GCGGCTTTCGCCGCCCCGACGACCTGCTCAAGATCGACGATTACGCTGGCACGCCGATCGGCTTCCTGCC

GCTTCTCCCTCCCGGTGGCATCGGTGCTGGTGGTGGTGGCCGCCTGCTGCATCACCGAGCTCGTTCTTCG

CAGCTGGCCGCCGTGGACGCGTATGGCGGCATGCTGGAGCTGGACTTCATCCCTTCCATTCCGCGATGA

>SbWRKY30

ATGTCTTCTGGTGGAGGAGGAAGCAGTGGCGGAGGAGGAGATCACCATGGCGTCTACCACCAGCATGGCCACGGCCACCTCGCCCGTGCCGATGCCGGCGCCGAGTACGTGTTCCACAGCAACGACATGGAGAGCTTCTTCTTCAATCAGCCGGCGGCGTCGGCGGGCGTCGATGGATCAGGCAGCAGAACTACTGGCGCCGCCGACGAGCTCATGCCGCCCTACTCCAGCATCACGGACTACCTGCAGGGGTTCCTGCAGGACCCCTCCGGGCTAGCTCGGCACCTCGACGCGCCGTGTCTTCCCGCGGAGGACGCCCCGCTCAAGCACGAGCTGTCTGTCGATGTGAGCCACGACAGCCAGGGCACCAGCGGCGCGCCCGGAGGAGAAGGGGCGGCGATGCACACGCCGAACTCGTCGGTGTCTCTCTCGTCAAGCGACCGGGAGGGGGAGGGCGGCCAGCAGCCTCGTCGGTGCAAGAAGGGCAGGCCCAAGGCGGAGGATGCGGAGGGGGATGAGAAAGAGCAGGAAGATGGGGAGAATTCCTCCAAAGCGAACAAATCCAAGAAGAAGGCTGAGAAGAGGCAGAGGCAGCCTCGCGTTGCCTTCCTCACCAAGAGCGAGGTGGATCACCTTGAGGACGGCTACCGGTGGCGCAAATACGGCCAGAAGGCCGTCAAGAACAGTCCTTACCCAAGGAGCTACTACCGTTGCACGACGCCCAAGTGCGGCGTGAAGAAGCGCGTGGAGCGGTCGTACCAGGACCCATCGACGGTGATCACGACGTACGAGGGGCAGCACACGCACCACAGCCCCGCCAGCCTCCGCGCCGGCGGCGCGCATCTCTTCATGTCAAACGCGCACGGCGGGCTGCCGCCGCACCTGATGCCGTCCAGCTTCGGCCGCCCGGACCTGATGAGCATGATGCACCCCGCCATGGGCGCAAACCCTAGCATGTTCCTGCCAAGTATGCCTCCTCCTCACATGTCAACACCATCTCCTGCTCCTCCTCTTCAGCAGCACCACTTCACTGACTACGCCCTCTTGCAAGACCTGTTCCCTTCCACAATGCCCAACAACCCATAA

>SbWRKY32

ATGTCGTCGGGAGACTTCCACTTCCACGACGAGCTGGCGTCGCTGTTCGCGCAGCGGCCGGCGGCGCCGGGAGAGATGATGATGGCGCAGCAGCAGCAGCAGCAGCAGCAGGCGCCGGCGTCGTGGTTCGCGGACTACCTGCACGGCGCGGGCGTCCCAGGGATGGGCGGGATGGACTACGACCTGCTGTGCCGCGCGCTGGACCTGCCGCTGCCGGGGGACGACGTCGTCAAGAGGGAGCTGCTGGTGGTGGACACAGGCGGGGGAGGGGGAGGCTTAGGCTTCGCCGCGCCCACGCCCAGCGGGGGCGGCACGGCGCCGGTCACGCCCAACACGACGTCGTCCATGTCCTCCTCGTCTAGCGAGGCGGCGGGTGGCGGCGCCGCCGGAGGAGGAGGAGGAGGAAGCTTTGGTGGTGCCGGGGAGGAGGACTCGCCGCATCAGGGGAGGTGCAAGAAGGAGGAAGGGGATGGGGAGGAGAGCAAGGCGCTCGACAAGGGGGAGGAGGACGCTGACAAGGGCAAGAAAGGGTCACCGGCGGCGGCGAAGGGCAAGGGCAAGGGCGAGAAGCGGCAGCGGCAGCCGCGCTTCGCCTTCATGACCAAGAGCGAGGTCGACCACCTCGAGGACGGCTACCGGTGGCGCAAGTACGGCCAGAAGGCCGTCAAGAACAGCCCATACCCCAGGAGCTACTACCGGTGCACGACGCAGAAGTGCCCCGTGAAGAAGCGGGTGGAGCGGTCGTACCAGGACCCGGCGGTGGTGATCACCACGTACGAGGGCAAGCACACGCACCCGATCCCGGCCACGCTCCGCGGCAGCACCCACCTCCTCGCCGCGCAGTTGCACGGCGGTCACCACCACCACCACCACCTCGGCGGCGCCTTCCCGCCGCCGGCCCCGCTCCCGCAGCAGATGGCGGGCGCGCCGTTCGGGCGGGCAGGCGGCGGAGGCGGAGGCGTCATCGACATGCTGGGGCTCCTGCCCCCGCGCAACAACAACCACGCCGCCATGCCGCCGGCGATAGGCCTCGCGTCGTCCCGCGGCATGAGCGGCGGCGGTCCGATGAGCACCGTGGCAGGCGCGACGGCAGCGACGGCCGCCGCCACCACTACCTCGTCGTCGTCTCCTCCCTCGCTCCAGATGCAGCACTTCATGGCGCAGGACTTCGGGCTCCTGCAGGACATGCTGCCGTCCTTCGTCCACGGCAATGGCGGCAACGTCCAGCCCTGA

>SbWRKY84

ATGTCGGGCGCGAGGCATGAGCACCACCTGTCCGGTGACTTCCAGTTCCACGACGAGCTGGCGTCGCTGTTCGCGCATCAGCGGCCCGACGCGGCGCCGATGGCGCAGCCGTGGTTCATGGACTACCTGCACGCGACCGCGGCGGCGGCGTCGCCGCTGGACTGCGACGCCTTCGTGGGGGACTTCATCGACGTGCCGGCGGTGGCCGCGGACGAGGTAGTCAAGAGGGAGCTCGTGATGGTGGATACTGCGGCTGCCGGCAGCGGCGGCGGGACGCCGACGCCGACGACGGCGCCGCTCACTCCCAACAGCATGTCGATGTCGTCCACGTCCAGCGAGGCTTGCGGCGCCGGGGCCGGAGCGGGCGAGGAATCGGCGGCTGGTAAGTGCAAGAAGGAGGACGGCGAGGAGGAGGGGCTCGAGAGCAAGGACGACGGATCGGCGGCGGGCAAGGGAGACGGTGGGGAAGGAGAGGAGAAGAACAAGAAAGGGGCGGCCAACAAGGGCAAGGGCAAGGGCGAGAAGCGGCCCCGGCAGCCGCGGTTCGCGTTCATGACCAAGAGCGAGGTCGACCACCTCGAGGACGGCTACAGGTGGCGCAAGTACGGCCAGAAAGCTGTCAAGAACAGCCCATTTCCGAGGAGCTACTACCGGTGCACGACGCAGAAGTGCCCGGTGAAGAAGCGGGTGGAGCGGTCGTACCAGGACGCGGCGGTGGTGATCACCACGTACGAGGGCAAGCACACGCACCCCATCCCCGCCACGCTGCGCGGGAGCTCGCACCTCCTCGCCGCCGCGCACCACCACCCGATGGGCGGCCTCCACCACGTGCACCCGCACTTCCGGATGGCGCCGCCGCCGCCGCCGGCGGCGCTCGGCGGCTTCAGGCCCGGCGGCGGCGCCAACGCCTTCGACGCGCTCGGACTCGGACTCCTGCAGCCGCCGTCGTCGTCGCAGCAGCAGCAGCAGGGCCACCATCACCACCACGGCGCCGCCGCCATGCAGCAGCTGGCCGTCAGCGGCGGCGCCGCCGGAGTGCAGCAGGTGAATGCTGCCGCCGCCATGGCGAGCCACGCGGCGCTGCCAGACGATGGTGACCAGCACGGCTTGGCTGCCATCGCAGGTGCGGCTGGTACTACGACGGCGGCAACTACTGCCGCCAGTGCTCCGCTCCGGATGCAGCACTTCATGGCGCAAGACTATGCTGGGCTCCTGCAGGACATGTTTCCATCCTTCGTTCACAGCGACGATGATGGCCACCATCACCACCATTGA

>SbWRKY4

ATGGCCGGCGCCGCCGGCGACAGATCGGAGGACGTCGGCGCCGACTGGCCATTCGGTGGCGGCGCCGCCGACGCCTTCACGGAGTACTCGTCCGTGTTCGCGGAGCTCGGCTGGCCGGGCGGCCTTCTAGCCAGCGGGGAGCTCCCGGTGCTGGATCTGCCCGACCCAGCAGCACCGCTGCCGTCGTCGTCGCAGCTGCTGTCCATAGAGCCGTCGGAGGACCCGGCGCCCGCCCGGTCGGGCGACGCCGGCGCGTCGTCGAGCTCGAGCGGGGACGGCGACGGCGCCGCGCCGGGCAACGACGACGACGATCGGAAGGCGGCGCCGGCCGCCGAGGCAGCGGGTAGGAAGCCGGCGGCGGCGACGGCGAAGAAGGGGCAGAAGCGGCCGCGACAGCCGCGGTTCGCGTTCATGACAAAGAGCGAGATCGATCACCTCGAGGACGGCTACAGATGGAGGAAGTATGGCCAGAAAGCTGTCAAGAACAGCCCTTTCCCAAGGAGTTACTACAGATGCACCAACAGCAAATGCACGGTGAAGAAGCGCGTGGAGCGGTCCTCCACCGATCCCTCCGTGGTCATCACCACCTACGAGGGCCAGCACTGCCACCACATTGGTCCATTCCAACGCGGCGGCGGCGGCGGCGGCGGAGGCGCAGCCACGGCGCGCTACCACAGCGCGGCGGCCGTGGCACTAGCGGAGCAAATGTCTTCATCGTCGTCGTTCATCCCAGCGCGGCAGCTCTACAGCTTGCCGCCGTTGCACCCACCACAGAGCTCCCTGTCCTCAGAAGCCGTCGTTAGCTCGGCGGCGACTACATCTTTTCATCAGCATGTTAACGACGGCGACGAGCTGCGGCAGGCTAGCTACAGCTCGAGGGTGTCCATGGCGCAGTCGCCATCAACTCCATCGTCGGTGCCTCCGGCCATTTCAGTTGAGAAGGCCGGGCTACTGGACGATATGGTGCCCCATGGTGTGAGGCATGGAACACCATGA

>SbWRKY12

ATGCAACAAGCGCAAAGGCGGCCGGTCACCGGCCGTGCATATACCCTCAGCACGCAGCACATGGCGTCTTCACCGAGCAAGCCGCCGGCGCCGGGGAAGACGACGAAGTCGTCGGCGGCGGGGCAGAAGCGGGCGCGGCAGCCGCGGTTCGCGTTCATGACCAAGAGCGACGTGGACCACCTGGAGGACGGATACCGATGGAGGAAGTACGGACAGAAGGCCGTCAAGAACAGCCCCTTCCCCAGGAGCTACTACCGGTGCACCAACAGCAAGTGCACGGTGAAGAAGCGGGTGGAGCGGTCCTCCGACGACCCCTCCGTCGTCGTCACCACCTACGAGGGCCAGCACTGCCACCACACCGTCGCCTTCCCGCGCGCCCACCACCTCCACGCCGCCCTCGCCGCCGCCGGCCACCACCACATGCCATTCAATTTCTCTGCCGCCGCGCACCACCACCACCTCTACGGCACCACCAGCGGCGTCGTCACCGACCACGGCCACCTGCCGCCGCTGCTTCTCCCGACGACACCCGCGCCTCAGCACAACGCTCTCAACGACAGCGACAACAACGGCTCGCCGCTCGCCTGCAGGACGTCGACGACGACGTCGTCGCTGCTAAGGCCACTCGACTGCAACCACCAGGAGCTTCTGCTGGCGGCGGCAGCGAGCTACCCCTTGTCGTCCTCAGCTGCGATGTCGTCGATGCCAGTGCCTTCCATGTCAACGACGACGACGACATCGTTGCCGCCGCCGGCTAGCAGTGCCGTCGACAAGGGGCTTCTTGACGACATGGTGCCGCCGGCGATGAGGCATGGATAG

>SbWRKY25

ATGTTCCCGTCGCCAGGGAGGACGGTGATGGCGCTGGGCCACGGTGGCCAGCACATGACCTCGTCGTCCACCGCCGGAGCCGCCGGCGGCATGGCGGCCGCCTCGTCGTCGTCCACCCCCACCATAACCTTCGCGTTCCAACCGTCCCCTCCGCCGACGAGCGGCCTCGCCCTCGCCCACCATGGCGTGCTAGGTTACGGCTCCTCTTCCCTTCTCCTGGACCACCACCACCACCCAACAACCACCACCACCTCCTCGGCGGCCTCTTCCTCGCACGCGGCTTCGTCCATCACCCTCCACCACCACCTCCATGGCCATGCAGCAGCAGCAGCGCCGCACGCTTCCCTTTCCCCTCCCACGAGGGCGTCGCCTCCTCCTCACCCATGGTCGACGACGACGACGGCCTGCGAAGAAGCAGGAGGGCCGGCGCCGGCGCATGATCGCCAAGCAGGCCAACAAGGCGGGAGGCCGCCGAGGGGGAAGGGAGCTGCTGCGGTGATCAGCGAGGGGTCGGCGGCGGCGGCGCTGGGGGTGGGCGCCGTGAGGATGAAGAAGGCGGGCGGCGGCGGAGGAGGAGGAGGAGGGAAGGCGCGGCGGAAGGTGCGCGAGCCGCGGTTCTGCTTCAAGACGATGAGCGACGTCGACGTGCTCGACGACGGCTACAAGTGGCGCAAGTACGGCCAGAAGGTCGTCAAGAACACGCAGCACCCAAGGAGCTACTACCGTTGCACGCAGGACAACTGTAGGGTGAAGAAGCGGGTGGAGCGGCTAGCTGAGGACCCTCGCATGGTGATCACCACCTACGAGGGCCGCCATGTCCACTCCCCGTCCCGCGACGATGACGACGACGCTGCGCGCGCCAACGCCGAGATGAGCTTCATCTGGTAG

>SbWRKY49

ATGCAGGCATATATGGAGGGAGGCCAGTTGAGTGCTTGCCTTCCTGGCTTCCTTGTGCCGGATCACTACGCCTTCCCTCTTCCTCTCCCGCTACAACTTCCTAGCAGCCAAAACAAGCTTTTCCAGATGCCGTTTGTAGTTGACCAGGAAGCAGAGACCGAAAACCATGGCGGCGGCGGGATGCTCTCCTCCGACCATTGTGGACTATACCCGCTGCCGGCACTGCCCTTCGGCAGCTGCTCCGGTGCCGCCGGCGCCGCAACAGCGTGCGGTGGGAAGCCTACGGCCGGTTTCATGCCCAGTGCTATTGTCGCTGAGGAGGTCTGCACCTCGGTGACTACTAAATTAGGTTGCAACGACAGTAATGGCACATGGTGGAAGGGTTCGGCAGCTACAACGATAGCGGAGAGAGGGAAGATGAAGGTGAGGAGGAAGATGAGGGAACCGAGGTTTTGCTTCCAGACCAGAAGCGACGTGGATGTACTGGATGATGGCTACAAGTGGAGGAAGTATGGGCAGAAGGTTGTCAAGAACAGCCTCCATCCAAGGAGCTATTTCCGGTGCACTCACAGCAACTGCCGCGTGAAGAAACGGGTGGAGCGGCTGTCGACGGACTGCCGCATGGTGATGACCACGTACGAGGGCCGCCACACGCACTCTCCCTGCAGCGACGACGCTTCCTCCGCCGACCACACCGATTGCTTCACCTCCTTCTGA

>SbWRKY60

ATGCATGCGTGCATGGAGGGGAGCAGCCAGCTGTTGGAGACCTGCCTTCCTGCTAGTAGCCTCTACGCGCTCAGTCCGCATCATCCTCTTCTTGCCCCGCTGCCGAACCAGCACAAGCTTCTGCAGATGCCGTTGGTCCAGGAGCAGGCTGCTGCGAATAATCATGGCGTGATGCTCTATTCGGACCACCACCACCACGGCGGCGGCCTCCTGTACCCGCTGCTTCTTCCCGGCATCCCGTTCTGCCCCTTCTCCGCCGCCGCCGACGCCGCCACCTGCGATAAGACCACCACCACCGGCGGCTTCGCGGCGCTCGATGCCGGCGAGGCGGGCACCTCAGTGGCGAAAGCCGCCGGCGAGATCGCTAGTACCACCACCACATGCAACGGCCCAAGTTCCTGCAATTGGTGGAAGGGCCCGGCGGCGGCGGGGGAGAAAGGCGGACGGATGAAGGTGAGGAGGAAGATGAGGGAACCCAGGTTCTGCTTCCAGACCAGGAGCGACGTGGATGTGCTGGACGACGGCTACAAGTGGAGGAAGTACGGCCAGAAGGTTGTCAAGAACAGCCTCCATCCAAGTTCGCTGGTCAGCGCCTTCTCTAAATTATCACTATGCATGCGTACATCCAGGAGCTACTACCGGTGCACCCACAGCAACTGCCGCGTGAAGAAGCGAGTGGAGAGGCTGTCGGAGGACTGCCGCATGGTGATCACCACCTACGAGGGCCGCCACACGCACTCCCCCTGCAGCGACGACGCCGACGCCGCCGCCGGCGACCACACTGGCAGCTGCGCTTTCACGTCGCTCTAG

>SbWRKY35

ATGGAGAACCAGCATCTCCAAGGAGACGAGTCGTCGTCGTCGCACGCGCTCCCCAGCTTCCCCTACTTCGCCGTGCCGTCGCCGCCGTATGCGCCGCCGCCGCCATCGGAGGACCAGCACAGTACCCTCATCACAGCGCTCCAGCAGCAACCGTCGTCGTCGGCGTGCAATAACGACGACCTTCCTCCTCTGGGTCTGGGGCCTGATCAGCTAGCGGCGGTGGCGGCGCCGATGATTCTCCCGCCAATGGTGGACTGGTCGGCGCTGCTCCAGCAGGCCAGCTTGATGGGGCCCCAGCTCGTGCCGGGACTACTGCAGCAAGTACCGCCCCTGGAGCCGTTGGACCAGAGCGGGGAGAACGACGGCGGCGACGCAGGGAGTAGCAGTAGTAGCAAGGAGAAGGTGGTGGCGAAGGGCGGCGGCGGCGCGGGGAGGTCTGGGAAGAAGAAGGCGAGCAGGCCGCGGTTCGCGTTCCAGACGAGGAGCGTCAACGACATCCTGGACGACGGCTACCGGTGGAGGAAGTACGGGCAGAAGGCCGTCAAGAACAGCGAGCACCCCAGGAGCTACTACCGGTGCACCCACCATACGTGCAATGTGAAGAAACAGGTGCAGCGCCTGGCCAAGGACACGAGCATCGTGGTCACTACTTACGAGGGCGTCCATAACCACCCCTGCGAGAAGCTCATGGAAGCGCTCAGCCCCATCCTCAAGCAGCTCCAGTTCCTCTCGCAGTTCTAA

>SbWRKY82

ATGGAAAACTCGCCGCTCCATGGAGTCATCCGGCAACCAAACACTCCGCCGCTGGCTACGGGTACGTGCCTTGCCCCGCTGCCGCCGGCCGTAGCCGCGCCACAGCCGCCGGAGCAGCATGCGTGCAGCAGCGATGCGACGACGACGTCCCTCGTCCCGGGCGCGGCCACGATGATGAGTTGCCCGCCGGCGGCCGTGGACTGGGCGTCGCTGCTTCTCCCGCGCGCGCCGGGGACGCTGCACGTCGGGACGACGCCGCCGCCGGTCGCGAGCGAAGTCGAGAGCGGCGGTAGTAGCGCTGTGACGGTGGCCGGGAGCAGTGCTAGTGCGACGGCGGCGGGAGAGGGAGATAATAATTATAAGGCCGGAAAAGCTGGGAAGGCCGGCGGCGGCGGGAGGGGGAAGAAGAAGGCGAGCCGGCCACGGTTCGCGTTCCAGACGCGGAGCGACAACGACGTCCTCGACGACGGCTACCGGTGGAGGAAGTACGGGCAGAAGGCCGTCAAGAACAGCGCATTTCCAAGGAGCTACTACCGGTGCACGCACCACACGTGCGACGTGAAGAAGCAGGTGCAACGGCTGGCCAAGGACACGAGCATCGTGGTGACCACGTACGAGGGCGTGCACAACCACCCGTGCGAGAAGCTCATGGAGGCACTCAGCCCCATCCTCAAGCAGCTCCAGCTCCTCTCGCAACTCCAGTCTTGCACTAATCAACTCCTCTGA

>SbWRKY57

ATGGAGAATTATCACATGCTCTTTGGGGCGGCGTCCACGCACGCCTCATCTGCTGCCACCCCCAGCTCGTACAACTTCATGGCCACTGCTGCTGGAACCAGCGGCGGCGGCGGCGGCGGCTTCCACGACCACGATCGAGGCCAGCGCAGCAGCGGGCACGGCGGCGGCGGATCGTCGTCGTCCTTCTTCGCGGAGCTGTCGTCCAACAACGACGACTCCAAGGAATATGGTGGTGCATCTAGCCCTCCAGGTCCAGCTGCCGGTAGTGGCCGTGGGGAGTCGTCCGTGGGGCCTGCGGCGGCCGCCGGCGAGGTGGACAGGCCGCCCAAGAGGAAGGGGGAGAAGAAGGAGCGCCGGCCACGGTACGCCTTCCAGACGCGCAGCCAGGTCGACATCCTCGACGACGGCTACCGGTGGAGGAAGTACGGACAGAAGGCAGTCAAGAACAACAACTTCCCAAGAAGCTACTACAGATGCACTCACCAAGGGTGCAACGTGAAGAAGCAGGTGCAGCGGCTGTCAAGGGATGAGGGCGTGGTGGTGACCACATACGAGGGCACCCATACACACCCCATTGAGAAGTCTAATGACAACTTCGAGCACATACTCACCCAGATGCAGATCTACTCCGGCATGGGATCAACCTTTAGCAGTAGTAGCCACAACATGTTTCACTGA

>SbWRKY23

ATGGCGGCATCGCTAGGACTCGCCCACGACGCCAGCTGCTACGCCGCCTACCCGCCGGCCGCCGCCGCTGCCTCCTCGTACTTCCCATCACCACCACCACCCGGCGACCTCGTGGCGGAGTTCCCACCGACCGCCGCCGCCACGGCCATGGCTGATGACTACTACTACTACTACTTCCAGTTCGGCGAGGAGATGGGCGGCGCTCGCGCTCCCGGCTGCGGCGGCGGCTACTGCTCGCCGCCGGCGCCGGCGTTCGACAATGGCATGAGCCTGCTGAGCTATGGCGGCGTCGACGGCGACGGGAGGAGGCCGATGAGCGGACCAGCTGCTGGCACTGGTGGGAACGGCGGCGGCGGCCGGCCGCCGGCGTCACGGATCGGGTTCCGGACGAGGTCGGAGGTGGACGTGCTGGACGACGGCTTCAAGTGGCGCAAGTACGGCAAGAAGGCGGTCAAGAGCAGCCCCAACCCGAGGAACTACTACCGGTGCTCGTCGGAGGGCTGCGGCGTCAAGAAGCGCGTGGAGCGGGACAGCGACGACCCGCGCTACGTCATCACCACCTACGACGGCGTCCACAACCACGCCGCGCCGGGCGCCGCCTACCTCTGCCCGCCGCCGCCGCGCGGCGCAACCGCAACCGCAGCAGCGCCCTGCTTCTCGTCTCCATGCTCGGGCTCGGCGTCGGCGGCGCTGGTGGCAGCACCCAGCTGGAGCGGCGCTTTTGACGCGTGGGAGGCGCAGCTGGCGGCGGCGGCGGCTCACTCGTCGGAGTCGTCGTACTGA

>SbWRKY77

ATGCAAATGGCGGCTTCCCTGGGACTGAACCCTGAAGCTCTCTTCGCTTCATACTCGTCTGCCTACTCCTCCTCCTCGCCGTTCGTGTCCGACTACGCGGCGAGCTTCCCGGCGGCCGTCGACTCCGCCACGGCCTTCTCCGCGGAGCTCGATGACCTTCACCACTTCGACTACTCACCGGCGCCGATCTTCACAGCTGTCGGAGCCGGGGCTGGCGGCGATCGCAACGAGAAGATGATGATGTGGTGTGAGGGCGGTGGTGATGAAAAGAGACTCAGAAGCAGTGGAAGGATCGGGTTCAGAACGAGATCAGAGGTGGAGATCTTGGACGATGGATTCAAATGGAGGAAGTATGGGAAGAAGGCTGTCAAGAACAGCCCAAATCCAAGGAACTACTACCGCTGCTCGTCGGAGGGCTGCGGCGTGAAGAAGCGGGTGGAGAGGGACCGCGACGACCCCCGCTACGTCATCACCACCTACGACGGCGTCCACAACCACGCCAGTCCCGGAGCCGCCGCCATCATCCAGTACGGCGGCGGCGGCGGCAATAGCGGCTTCTACAGCCCGCCGCACAGTGGCTCGCCGTCGGCTGCCTCATACTCGGGCTCCTTCGTCTTCTGA

>SbWRKY33

ATGCACATGGCGCTGTCGTCCCGCAGCTCGTTCGCCGCCGCCGACGTCCTCCTCCCAGCAGCCATGGCGTATCGTCAGCCGTGCAGCGGCGGCGGCGGCGGCCCTGCTACCTCCAGCTACTTCGGTTCTCGGCCCGCGGCCCCTTTTTTCCCGTTCGGTACGGCGGCGCAGCTGGACGTCTTCGAGTGCCTGTCGGACGAAGGCGGCGCCGTCCCGGCACCACCGGCAGCTGTACCTGGTGCGTTCGCGACGCCGCCGCCGCCGCTGCCGCTTATGCCGGCCGAGCGCGTCGTCCCGGACGCTGCTGCAGGCTATAGTAGTCATGCTAGGAGTGCGGCGGCGGCGGCGGCGGGTGAGGGGCCGCCGAGGAGGACGGACAGAATTGCGTTCCGGGTGAGGTCGGACGACGAGGAGGTACTCGATGACGGCTACAAATGGAGGAAGTACGGCAAGAAGTCCGTCAAGAACAGCCCGAATCCGAGGAACTACTACCGGTGCTCGACGGAGGGCTGCAGCGTCAAGAAAAGGGTAGAGCGAGACAAGGACGACCAGAGATATGTGGTGACCATGTACGAGGGAGTGCACAACCATGTGAGTCCTGGTACCATCTATTACGCCACCCAGGACGCCGCCTCCGGACGCTTCTTCGTCGCCGGGATGCATCAGCCAGGCCACTGA

>SbWRKY83

ATGGCGGCAGTCGGAGCGCGCCCAGTGCTGTACCACCACCCGGCGCCGGCGGGCGACGCCGCCTCCATGTCCTCCTACTTCTCCCAGGGAGGCAGCTCCACCACCTCCAGCTCCGCGTCCGCCAGCTTCTCCGCCGCGCTCGCGCCGACGACGACCACGCTCGCCGAGCAGTTCGACATCTCCGAGTTCCTCTTCGACGACGCGGGAGTCGCCGGCGCGCCAGGCGTGTTCGCCGATGGCTCGGCCCCCGTCGTCGTGTCGGATGCCGCCGCCGCTGCTGGTGGTGGTGCAATCAGCGCAGCCGCTGGGAGCGCGGCTGCGGCGGCGGAGGCCGTGCCGGAGCGGCCGCGGACGGAGCGGATCGCGTTCCGGACGAGGTCGGAGATCGAGATCCTGGACGACGGCTACAAGTGGAGGAAGTACGGCAAGAAGTCCGTCAAGAACAGTCCCAACCCAAGGAACTACTACCGGTGCTCGACGGAAGGGTGCAACGTGAAGAAGCGGGTGGAGCGGGACAGGGACGACCCCAGCTACGTGGTGACGACATACGAGGGGACGCACAACCACGTCAGCCCCAGCACGGTGTACTACGCCAGCCAGGACGCCGCCTCCGGCCGCTTCTTCGTCGCCGGCACGCAGCCGCCGGGCTCCCTCAACTAA

>SbWRKY27

ATGTCGTCGTCCTACTCGTCACTCCTCTCACCGAGCCGCGCCCACGCGGACGATCATCGGGTTCTTCTCGGCGGCGATGCCGACGACGACGACATGGCGGCCGTGTCAAGCTACCTCTCACTCGACGACATCGTAGATGATGTCGTCGGCGGGGAGTGGTACCGCCCGCTGGCGGAGGAGTCGTCGTCGGCGGCGGCGGCTGAGTTGCAGCCGGAGCCACTCTTGTTTGCCACGCTGCAAGCGGAGGATGGCTACTGCGTCAGTGGCAGCGGCGGCGAGCAGAGCTCAGCGGCACTAGCCAACGACAACCACGACAGGATCGACCTGACGCAGGACGGAGGCTCGAGGAGGCTCCTGAGGAGCGAGCACGGCAAGATCGCCTTCAAGACGCGGTCGGACGTGGACGTGCTGGACGACGGGTACCGGTGGCGCAAGTACGGCAAGAAGCTGGTCAAGAACAGCCCAAACCCGAGGAACTACTACCGGTGCTCCAGCGAGGGGTGCCGCGTGAAGAAGCGGGTGGAGCGGGAACGGGACGACGCGCGCTTCGTCATCACCACCTACGACGGCGTCCACAACCACCCGGCCGCCGCGCCGCCACGGTCGCCGGCCTATCGCCTCGGGGAGCCGCCGCACGGGCACCACGTCTAG

>SbWRKY63

ATGTCAGGGAAGATGCAACCTCATGAGAAGGTTGCTGTTTTGAAGCCTGTGGCCTCCAGGCCTTTCTCCAGGTTCAGGCCCTTTCCGAATGTTCTGCAAGACTTCAATGCCAATGGTTCCCCAACAATCACCGTCCCAGAGGAGACTGAACTAATCAGGCCAAAAGCCACTCGATCTGCATCCCTACTGGGTAATCTTCCAACACAGATAGCAGCAACAATAGATGCTGGGTCAGATGCCATATCTGAGGAGGTGGAAGCCAATGCAGAGCATTTGACGTGTTGTGACCATGTAACAGCATGCCAAGCTGCCAGGCGGAATGGTGTGCGCAGCCGTCTGTCACTCGATGGGTACAACTGGAGGAAATATGGGCAAAAGAAAGTGAAAGGCAGCGAGTTCCCACGGAGCTACTACAAGTGCACTCACCCTAGCTGCCCTGTGAAGAGGAAGGTGGAGACGACAATAGATGGCCGGATCGCTGAAATTGTGTACAGTGGCGAACACAACCACCTGAAGCCAGGCAAGCCTTGTCTCCCCAGGAAGCCATTGTCGTCGACAAGCACAGAGGTTGTGGTGTGCGACATGCGTGGTACTGACGACACGATGAGGGAGTAG

>SbWRKY44

ATGGAGGAGGACCTGCTTCTTCTGGAAGCATCAGTCGCTGTGCCGCCGCTTGCTGAGAGCATCAATAAGTACTGCTGGCCCAACGGCAGCGATTTCGCGACGACGGAGGAGCTGATGATGATGAGTGACCTCGTCGACGAGGCGGCCTTGTCGTCGTCGCCGGTGCAGCAGCAGGAGGAGGAGGAGCCTCGTCGCCAGCGGGAGTCGATGCTCAACAAGCTCATCTCCACAGTCTACTCCGGACCCACCATCAGCGACATAGAGAGCGCGCTCTCTTTCACCGGCGCCGACCAGGCCGCCGCCGTGGATGCTCACATCTACAACTCTGCCGGCCCAGTCGTTTTCTCCCCGGAGAAGGTGCTGAGCAAGATGGAGAACAAGTACACGCTCAAGATCAAGACCTGTGGGAACGGCCTTGCAGAGGATGGATACAAATGGAGGAAATACGGCCAGAAATCCATCAAGAACAGCCCCAACCCAAGGAGCTACTACCGGTGCACGAACCCTCGGTGCAACGCCAAGAAGCAGGTGGAGCGGTCGACGGAGGAGGCGGACACGCTGGTCGTCACCTACGAGGGCCTCCACCTGCACTACACCTACTCGCACTTCCTCCAGCCCCAGCCCCAGCCCCAGCCCCAGCAGCCCAAAAAGCCCAAGCTTGGAGGCCCACCACCACAACCACAGCCCATCATCATGCTGGAGGACCTCGACGGCCCGGCCCAACAAGATATCACCACCTGCCCACTGGACGCTACCGCCATGGCTCCAGCACCACCACCAGCTGCTTTGTGCTATCTTGACGATATGTTCCAAAAGCCTGCCTTTTTCGAGGAGGAGCTGCAGCAGCAGACATCACCAATGGCGGGTTGCTGGAAGACATGGTGCCGCTGCTGGTCCGGCGACCCTGCAGCAGCACAGGGGCCACCACCACCACCACTGGCAGCAGCACCACCTCGTCGTCGCCGCAGCTGGCACCGTCACCGGACCTTTCCACATCATCTTCTGTCTCATGGAACCCCACCTCTCCTTACATCGACATGGCCATTCTCTCCAACATTTTCTAG

>SbWRKY1

ATGGACATAGCGGAGGAGGCCGTCGCCGGCACCGCAGCTCAGGGCGACCTCGCCGAGGTTGTGGCCCGTGCAGGTGCAATGGCCATGGCCGCCGCGCCGAGTCACCACCGGCGGCCACCCTCTCCCTCTCCTGCAGCAGCAGATCACAGTCACGTCATGTCGAGGGCTGCCGGCCAGATCATGGCCATCCCTCCTGCTTGTTACGACGAGGAGCAGGAGCTACGGCCTGCTGCTGCTTGCGGTGACGCGGTGATGTTCGACGTGCCGTCGTCTATGGTCGTCGATCCATACCATCAGCTGTCTTCGTCGGCGGCCACGGCGCCGCCGCACGGGCACGGCGGGTACTGGCTGCCGCCACAACAGATCTCCCAGCAGGCCTGCTATGGCCTCGACGTGGCCATGGGAGGCGCTGCCGCCGCCGATGCCGACGGCGACGAGCCCATGATGATGAGGATCTCTCCAGTCACTCCGCCGCCGCCTTCTCATCATCAAATCATGAAGAGCAGAAAAAATGAGGTGAAGAAGGTGGTGTGTATCCCGGCGCTACCTCCGGCGAGCAGCCGGCCAGGAGGAGGAGAGGTGATTCCCTCTGATCTTTGGGCGTGGAGGAAATACGGCCAGAAGCCCATCAAGGGCTCGCCTTACCCAAGAGGTTACTACAGATGCAGCAGCTCAAAGGGATGTATGGCGAGGAAGCAAGTAGAGCGCAGCCGCAGTGACCCGAATATGCTAGTGATTACCTACACGGCAGAACACAACCACCCTTGGCCAATGCAACGAAATGTACTTGCTGGGTACTCCCGGCCTCACACTCACATGTCCAACTGCAAGAAGAAGAACAGCTGCAGGGTTGAACCGACGAGTTCATGGCCAACATCATCATCGTCGTCATCATCATCAAAGAATGCCAACTACTTCGAGCATAATGTAGTTCCCAGTAGCAACATCGAGTGTCAACAAATGACAAACATGATGGAAGATAATGCAGCTGGGTATGTTGCCTATGCCATCGATACTCTTGATGAGGAGGGTGTTGCGATGCATCAGCCGATCAATCGCAATAGCATCCAACCTTCAGATGAAGTTTTCGCAGAGTTGGAGGAATTGGAGCCTAGTAATAATCCTGTGAATGCAAACATCTACTCTAGGGGGGTAAGTTATGAGTGGCAAAAGTTCTAA

>SbWRKY8

ATGTGCGACTTCTTTTGGCTGTCGCCGGCCGATCAAGCAGGCGACCTCTCGGACGTTGTCCGGGCGAGCCTGCAGCCACCGCCGCCGCCGCCTCACCACCACCGACTGCCGCCGCCGGAGGAGGAAGAGGGGTTGCTGCTGCTGCAAGCCCGAGTGAACTTCGATGGCGACGATCACGATGAAGCGCGCTCGCAGCAGCTGGTGCATGGCAATGGCAGCATGAGGCTCATGCTGGGCAGCAACGGCAGTGGCGGCTGCGCTTGTGATCATGCTGCTGCCCTGTGTCCCCAGCACCATCCAGAGGCAGAGCGACTGATTCCTCAGCCGCCCATGTCCGGGCCGCAGCCACAGTTGTGCGCTGCTTCCAGCTTCGTCGTCGAGAGGGACGATGATGATGCTCCCGTTCTGGAGGAGCATGTCCTCGACATGGCGACGGCCCCTCACCCTCATCCTCATACCTCGGCAATCAAGCGAAGGAAGAGCCAGACGAAGAAGGTGGTGTGCATCCCTGCGCCGGTGGCAGCGCCGCCACCGGGGGTGGGCGGGCGGCCGAGCACGAGCGGCGAGGTGGTGCCGTCGGACCTGTGGGCGTGGAGGAAGTACGGGCAGAAGCCGATCAAGGGGTCGCCGTACCCGAGGGGGTACTACCGCTGCAGCAGCTCCAAAGGCTGCTCCGCCCGCAAGCAGGTGGAGCGCAGCCGCACCGACCCTTCCATGCTCGTCATCACCTACACCTCCGACCACAACCACCCATGGCCGACGCAGCGCAACGCGCTCGCTGGCTCGACCAGGCCGGCCTTCTCCTCCTCGTCGGCGGCCAGGAGTCATCACCATCACCACCATCACCATCACTCCGCGGCTGCCGTTCCGGATACGACACTGGCCCACCGTCACGCCAGCAACGTCGCCGTCGCCGACAATGCGACGCCAGGGTGGTCCATCATTAGCGCCAGTGTTCATCACCAGCTGCTCAAGCAGGAGGTGGTCGACGTGGATAACCACCCCATCAGCAAGCAGCCGGCGCAGGACGCTGCAGCTGACCGTGACTGCTTGGACATGTTTGCTGATATGGACGGCGCCCTCGACGTCCTGTGCGCCTCCAACTTCCACCCAAAAAAGCAGCAACAGCAGGTCACTGCTGCTCAGCACTTGGAGAAGCTGCCGGAGGAAGAAGACGAGCACTTGCTGCTGGGTCCGGATCCTTTCAGCTTCAGCTTCTTGGACTGGGTTGGCGCTTCATTTGGAGTTGGAGAGACAGCAGCAGATAACGGTGATCACAGTTAG

>SbWRKY48

ATGTGTGACTACTTCCTGCAAAGGATGGAGGGCGACCAGCATCACCAGGCCGGGGACCTCACGGACGTCGTCCGAGCCGGCGGCGCGATGCATCAGCAGGGAGCTATCGCGGAGCTCTCCTCCTCCACGGCCACGGGGTGGCAGCTCCCGGCCGAGCCAGCTCCAGCTGGGCCCGGCCTCTTCCTGCCACCGCAGCCGTCGTCGTCGGATGGCGGCGACGGCTTCGCGGACGCCTTCGCCGGCCTCCCGGACCCGTTCGCCAGCGACTTCGTCCGCGCCTCTTCCTCCTCCGGCGGCGGCCCCGTCCCTGCTGCCGACTTCTTCGACTTCGAGGCGCCTGCCGCTGCTGTCGGTGGTGGCGCCAGGAGAGGCGGAGGCGGCGTACTGGTGGATAGCGGCGGAGGCGGAGTGGTGGTGGAGAGGGGGGTGCCGCAGATGCCCGCGCTGTCGCCGAGGGAGATACGGCCGTACCCGGTGACGATGATTGGCGGCGACACCGTGAAGATCGGCGTGCCGACGATGATGCCCGGGCTGGCGGTTGGGCCGGCCTGTGCGTTCGACGCCATCGCCGGGTTGCAGATGCCGTCGCCGCACGGCGGCGGGATCAAGCGCAGGAAGAACCAGGCAAGGAAGGTGGTCTGCATCCCAGCACCTGCAGCCGCGGGAGGAAGGACCACTGGGGAGGTTGTTCCTTCTGATCTCTGGGCTTGGAGGAAGTATGGACAGAAGCCTATCAAAGGATCACCCTACCCAAGAGGGTACTACAGATGCAGCAGCTCTAAAGGATGCCCGGCGCGGAAGCAGGTGGAGCGCAGCCGGACCGACCCAAGTCTGCTGGTCATCACCTACAACTCGGAGCACAACCACCCTTGGCCGACGCAGCGGAACGCGCTCGCCGGCTCAACACGCTCTCACCACGCCAAGAACAGCAAGAACAACCCCTCGCAGCACAACCTGCAGAAGCCAGACCTTAAAGCCGAACCTGAGCATCATCAGGCCTCGGCGGCAGTAGTCCCTACTGGCTGCGCCACCACCGCGACGACCGCAGCCACCAGCACCACCACCACAGCGACCACGAGCACCACCAGCAACAGCACTCCTCCGCCGGCGACGATGGCAGTGAAAGAGGAGGCAATGGTGGGGTCGGAAATGGAGAAAGGGATGGACCATGACGCTTCTGTCTTGCTGGATCACGGTGATCTCATGCAGCAGATGTTCAGCCAGAGCTACTACAGGCCGATGATACCGGAGGCCGGCGGCGGCGGTGGCGGCCACCACGCCGATGACTTCTTCGCTGATCTCGCCGAGCTGGAGTCGGATCCCATGAGCCTCATCTTCCCAGGTGGTGGTGATCCTGGAAAGGAGAAGGAAATGATGCCCAACAAGAGCTTGGGCGCCGATCCATTATTCGGCATGCTAGATTGGGGTGCCACTAATAATGGTGTTGCTACTTCTGCAGGGAGTTCATTTGAGCAAGACGAGAGTGGTTGGTGA

>SbWRKY61

ATGTGCGACTACTTCCTTCCGAGGATGGAGGGCGACCAGGCCGGCGGCGGCGGCGGCGACCTTACCGACATCGTCCGGTCCGGCGGGGCCATCCCTGGCAACGCCGCTGAGATGTCGTCCACGGCCGCCGCGGACGAGTGGCAGCTCCAGGGGGACCCGATGCTCTTCCCGCCGCTTCCCTCGTCCACGACGTCGGAAGCAGCAGCCTGCTCCGCCGGCGGGGGCACAGGTGCCGACGTCTTCGGCGCCGACCCTTTCTCGGGGCTCGTGGACCCGTTTAGTACCGACTACTCCTCAGGCGCCGACTTCTTGGACGCCATGCCGGACGCGATGGCCAAGGTCGGCTTCGACACGGCTATCTGTGGCGGCAGCGGCAGCGGCTGCGGAGGAGGCGGAGCAGGCGGAGGCGGCCAGCTGATAGACATGAGCCGGAAGCAGCCTCTCTTGCCGCGGGGGGTGCAGATGCCGGCGCTTGGAGTGCTGGCGCCGAGGATGGTGTTGCCGTCGCCGTTGTCGTCGCCGAGGGAGATACGACCGTACCCGCCGTTAGCCGGCGACATGGTCAAGCTCGGGATCACGGCCGGGCAGGTGGCCGGGTGCGCCATCGACGCAGCCGTCGTCGGCATGCAGATGTCTTCGCCTCGCTCCGCGGGCGGGATCAAGCGCAGGAAGAACCAGGCAAGGAAGGTGGTATGCATCCCGGCGCCGACAGCAGCTGGGGGAAGACCAACTGGAGAGGTGGTTCCTTCTGATCTCTGGGCCTGGAGGAAGTACGGCCAGAAGCCTATCAAGGGTTCTCCTTATCCAAGAGGGTACTACAGATGCAGCAGCTCAAAAGGGTGCTCAGCACGGAAGCAAGTGGAACGCAGTCGGACTGACCCAAACATGCTCGTCATCACCTACACCTCTGAGCACAACCATCCATGGCCGACTCAGCGCAACGCCCTGGCCGGGTCAACTCGGAATCACCACGGCAAGAACAGCGGCGGCAGCTCAGGTTCCAAGAGCTCACAGAACGAGAAGCAACAGCAACAGCAGCAACCAAACAACGTCAAGGAAGAGCCCAAGGATCCGGCGGCCACGACGACAACCACTAGCACGATTACTACCACAACTACTAGTACTTCTCCGGCAGCGGTTGTGAAAGAGGAGACGCTGGCAGCTGGATCATCGTCAGAGGCACTGGGGCAACAAGTCATGGATACTACAGCGCTAGCAGTAGTAGACCACAACATTGAACTCATGGACCAGGTGTTCGGCGAGAGCTACAAGCCGATGATACCGGAGGCCGGCCACTCTGACGACTTCTTCTCCGACCTCGCGGAGCTGGAGTCAGATCCCATGAGCCTAATCTTCTCCAAAGAGTACATGGAAGCGAAACCCAGCAGCGGTGGTGATCGTGGCCACCATCAGGAGAAAGCAATGTCCAAGGACTTGGATCCATTGTTTGACATGCTAGATTGGTCTACTAATTCCTCATCAGCTGGGAGCCCATTTGAGCAAGGAAAGAGAGGCTAA

>SbWRKY29

ATGCGCGCGTGCGGGGGGAGGGCCCTGCTGGACTGTCAGTGCCAAAAGTCAACCCACCCCTTCCTCCTTCTCCAGTCCCCCGCATACGGGCAGGAGAGGAGCAGCACGCACGCGTCCCCTCTGTGCTCGTCAAAGGCTCCAGCGCGGAGAGCCCGGTCGTGGTTCATGCGCGGCGTGCTGCTGCGCATGGCGGAGCACTTCAACGACTGGGACCTGCAGGCCGTCGTCAGGAGCTGCGGCAGCGTCGCCGCGCACCCGGACCCCGCGGCGCCGAGGGCGGAGCCAGACGCAGCGCCGCCGGAGCCGACGACGACCACGCCCGTGGCGGCGCCGCCGGAGCGTCCGCCCGGCGCGCGTGCCACGCCGCCGACGCCGGTGGCTGTGCCTGTGCCTGTGCCTGTCCGGGGACAGGAGCAGCGGGCGCCGCCGCCCGTGGCGGCCAAGGCCGCGGCGCTCCTGTACGACCTCGAGTACCTGGATTTGGATCACAAGCCGTTCCTGATGCCGGTCGTCGCGCCGTCGCCGCGCGCGGGGGACAACGGGCGCGGCGAGCGCGAGGTGATGATCTCCTTCCCAGCGGGGGCCGCGTCCACGTCCGGGATGCAGCAGAGGGCGTCGCCGCCAGGCCGGAAGCCAGGCGCGCGCACGCCGCGGCCGAAAAGAAGCAAGAAGAGCCAGCTGAAGAAGGTGGTGCGCGAGATGCCGGTGGCCGACGGCGGCTCGTCGTCGTCGGACCCGTGGGCGTGGCGCAAGTATGGCCAAAAGCCCATCAAGGGCTCGCCTTATCCACGGGGGTACTACAAGTGCAGCAGCATGAAGGGGTGCATGGCGCGGAAGCTGGTGGAGCGCAGCCCGGCCAAGCCCGGGGTCCTCATCGTCACGTACATGGCCGAGCACTGCCACCCCGTGCCCACGCAGCTCAACGCGCTCGCGGGCACTACTCGCCACAAGACGTCGTCGTCCGGCGCGGCCTCCTCTCCCAAGAGCCACGAGCAGGGCCAGGCGGTCGAGAAAGCGGCCGGCCGTGGCGCCGGCGACCGCGAGCATGGTAACAACGAGACGTCGTCATCAATGGCCGGGGAGTTTGGCGGCGAGGAGATAGCGGTAGCCATCGACGACGACGAGTTCTGGCCTGCGGGGATGGACCTGGACGAGCTCTTGGCGCCCGTGGACGACGACTTCGATTTCGAGCACGTCGTGGAGGAGGAAGATGGCGTGCTGGGACGGCGGCTCTCCCTCTAG

>SbWRKY89

ATGCCAAAGTCAACCGTCTCCGTATATATGAACCCTCTCCCGGTCTCCCCCCACCTTGGCACACATACACACGCACGATCCATCGCCATGGACTGCTCCAACGACTGGGACCTGCAGGCGCTCGTGCGGAGCTGCGGCGGCGGCGGAACGGCAGCCGCAGCAGCGTGCAACAGCGGAGCGGCGCCGACGGCGACGAGGGGAGGGTACGACGCACCTTCACGGGAAGCTGCTGATGATGCTAGCGTCGTCGTCGGCGGCGGCGGCCGTGTGGTGGCAACGGCGGCGGCGGGACAGGAATTCCTCGGGCAGCCGGTGGCGGCGTGGCGGCGCAACCTCGACTACTTGGACCTCGTGGACCACGAGCTGCTGCGCATGCCCTTCTCCATCACGCCATCGTCGTCTCGAGAGACAACGTCTGGTGGGGCGCCTGGGCAGCAGATGATCCGGCAGCCCAGGAGGCAGCCCGGCCGCAAGCCGGGGGTTCGCACTCCCAGGGCCAAGAGAAGCAAGAAGAGGCAGGTGAAGAAAGTGGTGTGCGAGGTGCCGGCGGCCGGCGGCGGCGTCTCCTCGGACCTCTGGGCATGGCGCAAGTACGGCCAGAAGCCTATCAAGGGCTCACCCTATCCACGGGGATACTACAAGTGCAGCAGCCTCAAGAGCTGCATGGCCAGGAAGCTGGTGGAGCGCAGCCCGGCCAAACCCGGGGTGCTCGTCGTCACCTACATCGCCGACCACTGCCACGCGGTGCCCACCATGCTCAACGCACTGGCCGGCACCACGCGGAACAGGCCGGCGGCGGAGTCCCCCGACGATGGTGACCACCATCACCACCAAGAACACCATGACCACGAGACGTCGGACGGGGCGCCGGCGGCGTCCGCCGATAACAAGCTCGACGACGACGGGGCCGACGCCGCGTCCACCATGACCGTGGAAGAGAACGACGCGTGGCTGGTGGATCATATGGCGCTGGAGGACGACGTCGTCGACGGCGACTGCCCATTCGATGATTTCTTGTGGCCGTTCGATGACGACTTGGATCAGTTTCTCGACGTCGACGGCGGCGGCGTTCTTGGACGCCGGCTGTCGCTGTAG

>SbWRKY46

ATGGAGGAGGAGCGCTGCTTCAACAACTGGGATCTGGACGCCGTCGTGCGCCTGGGCTGCCGCCGCCGCCTCTCCCCGCCGGGCCAGCCCGACCCGTTCGCGTCGTTTCTGCCTCCGCCGCCGCCGTCGCCGCCGCACAAGGAGAAGCCCGTGGTGCCAGCGCCGGCCGCCAAGGAGCCAGAGCCATATGCGGCGTGGCGCTTCCCTGACCTCGGTGCAGCTGGCGGCGGGCAAGACGGCGACGAGCTCCTCAGGGCCCTGCTAGCCGCCCCCCCGCCTCCCCCGCCTCAGCCTCTGCCAACGCCAACTCCGCTGCCTCCCCCGCCGCCACAGCAGCAACGGCAGCCGGCTGTGGCGGCGGTGGACGTGCCGCTACCCCAGGCGCGTCCCGCTCCGGCGAGGGCGCAGCCGAGCGGACGGCAGGTGCCCGGTGGCGTGCCAAGATCCAAGAGAAGGAAGAACCAAGTGAAGAAGGTGGTCTGCCATGTTCCGGCGGACGGCTCGTCGTCGGACGTGTGGGCGTGGCGCAAGTACGGCCAGAAGCCCATCAAGGGCTCTCCCTACCCAAGGGGATACTACCGGTGCAGCAGCTCCAAGGGGTGCGCGGCGCGGAAGCAGGTGGAGCGAAGCCGCGCGGACCCCAACACCTTCATCCTCACCTACACCGGCGAGCACAACCACGCGGCGCCGACCCACCGGAACTCGCTCGCCGGCACCACCCGCCACAAGTTCCCCGCCTCGGCGACGCCTCAGCCGCCGCCGCCGTCCGTCGTGGTGGGCGGCGCCGGCGCCGGAGCCGGAGCCGCCCCCGGCGACGCGCAGCACCAGCACCAGCAGCCGAGCCCGAGCCCGACGTCGACGTCGACCGCGGGGCTCTCGCCCACGACGCCGCTGCGCACGCCGTCCATGGAGGAGGACGACGAGGAGGAGGAGGACGAGCTGCTGGTGGAGGACATGGAGATGGCCGGCGAGGACGAGCTCCTGTTCCTCAACCCCGACGCCGACGCCGGGGCACCCATGTCCTCGCTCTTCGACGTCGTCGACGAGCCCTTCCTGAGCTCCCCCTGGGTGACAGCCACCAGCAGCGCCGGCGAGCCAGCCACAGGGGCAGCCGGCGCCGGGAGCTGA

>SbWRKY92

ATGGACGACGGCCGCGACGGCAACAACTGGGACCTGAACGCCGTACTGCGCTCCGGCTGCCATGGTCCCATGCCGCCGCCGCCGCCGCCGCCGACAAGGACGGCCAACAACCCCGTCGCACGGTACGCCCCGCCACCGCCAGCTCAGCCGAGTTATGCCTTCACTGTCTTGGCCGGCGGCCTCGGCCATCAGGCCATCTCTGTGCTTCCTCAGCCGCAGGCGCTGGATCAGGATCCGCCCCACGCTGCAGGGCGGGGTCCGAGCCTGGATCTGCCGCTGCTGCCGGAGCCGGACTACACCGCAGCCGTCGGCAATACACCTGCGCCACTGAACCCACCGTGGCCAAGAAACGAAATTCCGGTGCCGTCCGTCCAGCAGCGACCAGCCGACAAACACAAGACGCCGCCTTCCAGCGGCTGTGACGCTGCAGAAGGGTCCAGTCGATCCAAGAAAAGGGACAACAGGACAACCAAAGAGAGCAAGGTGGTTCTGGTGCTAGCCGAGGATCCGACGCCACCAGACTCGTGGGCGTGGCGCAAGTACGGGCAGAAGTCGATCAAGGACACCCCGTACCATCGCAGCTACTATCGATGCAGCACCGACAAGAAGTGCAAGGCGCGGAAGCATGTGCAACGCTGCCTCACCCAAAGCTTCCTGGCCGTCTCCTACATCGGCGAGCACAGCCACCCGATGCCGCTGG

CCCGCAACGGCCAAGCCGGCACCACTCACCAGAAGCCGCCGCCGCGGCAGCCTACGTCGCCGTTCATCAGGACCCCCGCCAAGGAGGACCAGCCGCATCATCAGGCTCCAGCTCCGCCGCCGGCCACGTCGTCGTCGCCATTCGCCATGATCTCCGCCGCGAAGCAGACTCCGGCCCCGCCGGCCGCGCTGTTGCCCCCGCCGTCTGTGGAGTTCGACAACGAGAAGGACGATGACGCCGTCGCCGTCAGAATGCTGCTCAATGACATGGACATGACTCCCGAGGATGCACTGAAGTTTGTTAACCCTGAAGAAGAGCCCCTGGATGGCGTTGGCGACGACCTTCTGATCCCGACGCCTGAAGAGCTAGCACCGTTCTACTACGGTGATGAGGAAAACATGCTATACCCGATGGCTTCTGAGCCCGCATCAGGAGGCAGCCGCAACACTAAGGCATGA

>SbWRKY93

ATGGAGGGCGATCTGCGTTGGTGCTGTGGTAGCAGCAGCAACGACTGGGACCTACACGCCGTGGTGCGCCTCGCCAGCTGCAGTGGTGGCAGCCGCAGCCGCGTCACCTCGCCGTCGCCGTGGGCCTCGGACGAATCATTCTCCTGCCTGCCCCCGCCGCCGCAGTCACAGAAGGACGAGGTGACGACGGACGCCGCAGCGTTGCAGCAGCCCCTGATCAGCCCTGCCGTCGACGACCTCTGCGGCCTGCAGCAGGCCTTCTTGGCCGCCACGCCGCAGCCAAGAAGCGAAGCGCCGCCGCCGCAGCCGCCGGCGAAACCACGAACTTCCTACCGCAATAACGACGGCGGCGTTGGCGGCGGACCGACACGATCCAAGAGAAAGAAGAAGAAGAGCCAGGTGACCAGCAAGGAGGTGACGCGGGTGCCTGTGGGCACGTCTGCGGACCCCTGGGCGTGGCGCAAGTACGGGCAGAAGCCGATCAAGGGGTCGCCGTACCCGCGCGGCTACTACCGGTGCAGCACCGACAAGGACTGCAGGGCGCGGAAGCAGGTGGAGCGCTGCCGCACCGACGCCTCTACCCTCATCGTCAGCTACACCGGCGAGCACAGCCACCCAGTCCCACTCCACCGCAACGCCCTCGCCGGCACCACGCGCAACAAGCCGCAGCCAGCACCGTCCACCTCCCCAGCCGAGCAGCCTCCAGCTGCGTCGCCGATCGTGGGAGTGGAGTACGAGGAGGACGACACAGTCGCCGCCAGTGTGCTGCTCGAGGATGCCGAGACGGAGGGAGAGGAAGACGTGCTGTCGTTGTTCCTCGAGCTCGCTCCGAGTCCCAGCAACGGCAGTGGCTCCAAGGACGTCATGGTATCCACGGAGCTCCACAATGGCAGAGGCTCGCAGAAAGTCGTGGCACTCTCGAAGCTCCACGAGTTCCGACATCCAGCAACGACGAGCAGCAGCAGAACGAGCGATGGCTTAGGGGCGGCTCCGGCGGCCATGAACGTGACCCACGAGAACTGCCCTTTCTCAGGACTCCGACTCACGACGTAG

>SbWRKY2

ATGAAAGCCATGGAGGTAGTGGAGGAAGCCAACCGGGCCGCCGTGGAGAGCTGCAAGAAGCTCGTCGCCGTGCTCTCGCTCTCCGGTGCCGACGCGTTCCGGCCTTTGCCCGTGGCCGCGGAGACCGACGAGGCGGTCGCCCGGTTCGGCAAGGTGGTCGCCGTCCTGAGCGACAGGCTCGGCCATGCCAGAGCAAGGGTTGCTGGCAAGAGGAGCCCACCGGCGCCTCCCGTCGATGCGAGCTGCCTCTTGGAGTACCACCCGTCGCTGGCAGTGGCACCGCGACACACTACCAACGGCGGCCATCTGCTGGTCAGTGCTACTTCTCCTCCTCCTCCTCCGCCGCCGCCGCCGACGACGACGACGTCGCTGCTTGCTAGCATGACGATGCGAAGCGCTGCGGAGCCGATGACGATGAGAAGCCAAAAGGCGGAGGTAGTGGCGCCGGCGGTGCTGGTGTCGCCACCTTGTGCCAGCAACGTGACACTGACGCCGGCCCCGGCCAAGAAGTTTGACAGGAGCATGTTCCTCGAGACGTCGCTGCTAGAGTTAAACAACTCTTGCAGCGTGCCTCCATCTTCGTCGCCGGCCATGGCGGTGCAGAAGAGCAGCCCGAAAGTCGCCGCGCCCAATCCGTGCACCAGCACCCCCCACATCCAGTTGCAGCCCACCACCCAGTTCCAGCCACCGCCACAGCAGCAGGCGGCGAAGAAGCAGAAGAGCTTCCAGTTCGACCAGACGCCGAGCGGCGAGCAGTTCCACATCGAGGTCCCTGTGCCGCTGCCCCGTGGCGCCGCCCCCGCCGCGAAGGAGGTGATCAGCTTCAGCTTCGACAACAACTCGGTGTGCACGTCGTCGGCGGCCACGTCCTTCTTCACGTCCATCAGCAGTCAGCTGATCAGCATGTCCGACGCCGCGACGAGCTCCGCCGCCAGGCCCGCCACGGCGAAGAAGATGTGCGGCAAGGGAGTGGAGGACGGCGGCGGCGGCGTCAGATGCCATTGCCCGAAGAAGAAGAAGCCGAGGGAGAAGAGGGTGGTGAGGGTGCCGGCGATCAGCGACAAGAACGCCGACATCCCGGCGGACAACTACTCTTGGAGGAAGTATGGGCAGAAACCCATCAAAGGCTCTCCTCACCCAAGGGGATACTACAGGTGCAGCAGCAAGAAGGACTGCCCGGCGAGGAAGCACGTGGAGCGGTGCCGCAGCGACGCCGCCATGCTGATCGTCACCTACGAGAACGACCACAACCACGCGCAGCCGCTCGACCCCTCCGTGCTCACCGCCGCCAACGCGGAACCTTGA

>SbWRKY36

ATGTCGTCGGCCGAGGACGGCTACTGCAGCTCGGATTCGCCGCGGGCGGAATCCCCCGACGAGCCGCTGCTGCCTGCGGCGGTGGCGGACGCGGACGCGGAGTCCCCTCGCGCCGCCGGGTCCGGGATGAACAAGCGCGAGCGCGACCTCAGCGACCTCCCCGCCTCGCCGTCGTCGCCGCTGCCGCCAGCTAAGCGCAGCCGGAGATCGGTGGAGAAGCGGGTCGTGTCGGTGCCGCTGGCCGAGTGCGGGGACCGGCCCAGAGGGGCCACCGGGGAGGGGCCGCCGCCGTCGGACTCGTGGGCGTGGCGCAAGTATGGGCAGAAGCCCATCAAGGGCTCCCCCTACCCACGTGGGTACTACCGTTGCAGCAGCTCCAAGGGGTGCCCGGCGAGGAAGCAGGTGGAGCGCAGCCGCGCGGACCCCACCGTGCTGCTCGTCACCTACACCTTCGACCACAACCACGAGGCGCCGCAGCCGAAGAGCAGCAGCTGCCACCAGCAAGGCAAGCCGTCCACGCGGCCGCCGGCGCCGAAGCCTGAGCCCGTGGTCGAGCAGGATGAGCTTGGTCCGGAGCATGAGCTGGCAGAAACAGAAGTGCCAGAGCAGCAGGAGCCGGTGGAAGAGGAGCAGGAGCAGAAGGTCGTCCCAGGTCTGGCCGGGCCGGAAGCGGAAGCGGAAGCGGAACCAACCGCAACGGTGGCGCCGGCGGCAGCCGAGGAGGACGAAAGCTTCGACTTTGGGTGGTTCGACCAGTACCCGACGTGGCACCGTTCGGCGCTGTACGCGCCGCTGCTGCCGCCGGAGGAGTGGGAGCGGGAGCTGCAGGGGGAAGACGCCCTGTTCGCGGGGCTCGGCGAGCTGCCCGAGTGCGCCGTCGTGTTCGGGCGACGCCGCGAGCTCAGCCTGGCCACCACCGCGCCGTGCTCCTGA

>SbWRKY34

ATGGACGCCGAGTGGAGCGACGGCGCGGCGGCGGCGTCACCGCCGACGGTGTCCGGCGGCGAGAGCAAGCCTGGTGCTGCTGGTGCCGTCTCGTCGTCGGCTGATTGCCCAGGGTCGCCGCCGGTGTCGCCTGCGCCACCGTCGACGACGTCGCCAGCTGCTGCTGCTGCTGCTGCCGGGAGCGGGAGGAGGCGGTCGGCGAACAAGCGGGTGGTGACCGTGCCGCTGGCAGACGTCAGCGGGCCTCGGCCTAAGGGCGTCGGCGAGGGCAACACGCCCACGGACTCGTGGGCGTGGCGGAAGTACGGCCAGAAGCCCATCAAGGGCTCGCCTTTTCCGAGGGCTTACTACAGGTGCAGCAGCTCCAAGGGGTGCCCGGCGAGGAAGCAGGTGGAGCGGAGCCGGGCCGAGCCGGACAAGGTGATCGTCACCTACTCGTTCGAGCACAGCCACTCCGACGCCGTGGCGAGGGCGCAACAGAACCGCCAGCAGGCCTCGAAGCCAAAGGCGGTCCAGCGGCAGCCAGTCCCGCCGGAGCCGGCGGCGGAGTCCCCGTCATCCGGAAGCTACGACGTCGCTGCTGCCACGGTTTGCGGTGCCGGCGCGCCTGCTGCTGCTGCAGCCGGGACCGAGGTCGGCGGCGCGGCGTCCGTCGAGGTGCGCGACGAGTTCAGGTGGCTCTACGACGGCGTGTCCGTCACCTCCTCGGCGTCGCCCTCGGACGTCGAGGCCGCGGACGAGATGCTGTACGGGGCGATGTTCTTCGGCGCCGCCGCCGCCCCGCCCGCCGCGCCCCTCCCCGACGAGTTCGTCGGCGACGTCGGCGGGCTGTTCGACTACGGGGAAGGAGGCGGCGAGGAGGACGCCATGTTCGCGGGTCTCGGCGAGCTGCCCGAGTGCGCCATGGTGTTCCGGCGGCACGCCGGCGACGGGCTTTCGGTGGCCGGCGGGGTGAAGTGA

>SbWRKY5

ATGGGGCCGTCGTCCATTCAGGAGATGGAGGAGGCCCGAAGGACCGCAGTGCAGAGCTGCCACCGGGTGCTGGCGCTCCTCTCCAACCCGCACGGCCAGCTCGTCCCCAGCAAGGACCTCATGGCTGCCACCGGGGAGGCCGTCGCCAAGTTCGGCTCCCTGACGGCCAAGCTCACCAACTCCAACTCCAACTCCAACGGCAATGGCCTGCAGCTGCAGGGCCACGCTAGGGTCAGGAAGATCAAGAAGCCCCTGCCCATCTTCGACAGCAACCTCTTCCTCGAGAGCTCTGCGGTGGCCGCCGCCGCCGCCGCCACTGTGGCCAAGACGCCCAGCCCGAGCCCGATCACTGGCCTCCAGCTGTTCCCGAGGTACCACCAGATGGAGGGCTCGTCGTCTAAGGATCCTGTCAGGATCCCTACCCAGTTCCCCAAGAGGTTACTGCTAGAGAACCCGGCTGCCGGTCTGGAGGGGCTGCCGTCCAAGGCCCCTCCGGTCCAGATGGTCCAGCCGGTGTCGGTTGCGCCTCCTGCAGGGACGCCTACCCCGGCATTGCCCGCTGCTCACCTTCATTTCCTCCAGCAGAACCAGAGCTACCAGAGGTTTCAGCTCATGCATCAGATGAAGATTCAGAACGAGATGATGAAGAGGAGCAATCTTGGTGATCAGGGTGGTAGCTTAAGCGGTGGTGGTGGTGGTAAGGGTGTGAATCTCAAGTTTGATAGCTCGAATTGCACAGCGTCATCGTCTCGCTCCTTCCTTTCGTCTCTGAGCATGGAAGGGACTCTTGCGAGTTTGGATGGAAGCCGGGCCAGCAGGCCATTCCAGCTAGTTAGTGGCTCTCAGACATCTAGCACACCGGAGATGGGCCTGGTGCATAGGAAAAGGTGCGCTGGTAGGGAGGATGGGGGTGGTCGGTGCACTACCGGGAGCCGGTGCCATTGTTCAAAGAAAAGGAAGCTTAGGATAAGGAGGTCCATCAAGGTCCCTGCAATAAGCAACAAGGTTGCAGACATCCCAGCTGATGAGTTCTCGTGGAGGAAGTATGGGCAGAAGCCAATTAAGGGATCCCCACATCCTAGGGGTTATTACAAGTGTAGCAGCGTGAGAGGGTGCCCCGCGAGGAAGCATGTCGAGAGGTGCGTGGACGACCCCTCGATGCTGATTGTTACCTATGAAGGTGACCACAACCACAACCGAGTTCTAGCCCAACCAGCCTGA

>SbWRKY7

ATGGAGGAAGTGGAGGAGGCCAACAGGGAAGCCGTGGAGAGCTGCCACAGGGTGCTCGCCTTGCTCTCGCAGCCGCATGACCCCGCACAGGTCAGGAGCATAGCTCTGGGCACGGACGAAGCATGCGCCAAGTTTAGGAAGGTGGTCTCCCTGCTCAGCAATGGAGGAGTGGGAGTAGGAGAAGCCGGACCATCAGGCGCAAGTGGAAGCGGAAGCCATCCGAGAGCTAAGCTTGTTAGCAGAAGACAGAATCCAGGGTTCTTAACTCAGAAAGGCTTCCTGGATAGCAACACCCCGGTTGTGGTGTTGAACAGCGCCCATCCTTCTCCTGCCTCTGCGCAGGTGTATCCTAGAACTGCTGGAGCTCTGGATGCGCAGGGCGTGCATCCCCTCGGAGGACCGCCTAAGCTGGTCCAGCCTTTATCTGCGCATTTTCAGTTTGGCAATGTGTCGTCACGGTATCAGTTCCAGAATCAGCAGCAGCAGCAGCAGAAGTTGCAGGCTGAGATGTTCAAGAGAAGCAACAGTGGGGTTAACTTGAAGTTTGAGAGCACCAGTGGCACGGGGACAATGTCATCGGCGAGGTCCTTCTTGTCGTCTTTGAGCATGGATGGTAGTGTGGCTAGCCTGGATGGCAAGTCATCGTCGTTCCATTTGATCGGTGGGCCTGCAATGAGCGATCCGGTGAACGTGCAGCAGGCCCCAAGGAGGCGATGCACGGGTCGTGGGGAGGATGGGACTGGCAAGTGCGCTGTGACAGGGAGGTGCCATTGTTCAAAGAGAAGTAGGAAGTTGCGGGTAAAGAGGTCGATTAAGGTTCCCGCCATTAGTAATAAGATTGCTGATATACCTCCGGATGAATACTCGTGGAGGAAGTATGGGCAGAAGCCAATTAAGGGTTCCCCTCATCCTAGGGGTTACTACAAATGCAGTAGTGTGAGGGGCTGCCCAGCTAGGAAGCATGTTGAACGGTGTGTAGATGATTCATCAATGCTCATTGTGACATATGAGGGCGAGCACAACCACACCAGAATGCCAACTCAGTCAGCACAGGTTTAG

>SbWRKY74

ATGGAGGAGGTGGAGGTGGCCAACAGGGCCGCGGTGGAGAGCTGCCACCGGGTGCTGGCCTTGCTCTCGCAGCAGCAGGACCCGGCCCTGCTCAAGAGCATAGCTTCAGAGACGGCCGAAGCCTGCGCCAAGTTCAGGAAAGTAGCCGCCCTCCTCGGCAGTGGCAGTGGCGGTGGCGGCGGCTGCGGCCATGCTAGAGGCAGGTTCTCCAGACGAGTCCGGCCTATGGGTCTCGTGAACCAGAAGAGTCCCTTGGGGAGCGGCAGTGGTGGCGGCGGCAGCCCGCTGGAGATGATGCCCAGCACCGCTGCTGCTGCTGCTGCGGTGGCGGCTCCGTCTCCATCGACTAGCTACGCACAAATGCGAGCTCGGCTTAACGGTGTGCCAGACTCACGAGGGCTGGATTTGGCCTGCTCCAGCAGCAAGAGTGGCGGCCCTCATCCGTTCGGAGCCCCCAAGCTGGTCCAGCCGCTTTCTGTGCAGTTCCAGATTGGGAATGTTGCGCATAGGTACCCGTTCCACCAGCAGCCCCCGTCGCGGCAGAAGCTGCAGGCCGAGATGTTCAAGAGGAGCAACAGTGGGATCAGCCTCAAGTTCGAGAGCCCCAGCCCCAGTGGCGGCGCTGCTGGCACGATGTCGTCTGCGAGATCATTCATGTCGTCCTTGAGCATGGATGGGAGCATGGCTAGCTTGGATGGGAAGCGGCCATTCCATTTGGTTGGCACCCCGGTGGCGAGCGACCCAGCTGATGCCCACCGCGCACCCAAACGGCGGTGCACGGGTAGAGGGGAGGATGGAAGAGGCAAGTGTGCCACTACCGGCAGGTGCCATTGCTCAAAGAGAAGGAAACTGCGGATTAAGAGATCAATTAAAGTGCCAGCCATTAGCAACAAAATCGCTGATATACCTCCTGATGAGTACTCGTGGCGCAAGTACGGGCAAAAGCCAATTAAGGGTTCCCCCCACCCGAGGGGTTACTACAAATGCAGCAGCGTCAGGGGCTGCCCGGCAAGGAAGCATGTTGAGCGATGCGTAGATGACCCAGCGATGCTAATCGTGACATACGAAGGCGAGCACAACCATAACCAGTTGCCAGCACAGGCTGCCCAGACCTAG

>SbWRKY47

ATGATCACCATCGATGATCTGCTGAGGAGCTGTGGCGGCGACAGCGGCAGTATTCCAGTTCCTAGCAGCGACGACGGGCGGCAGATGCTTGCGATGGGCGACCACCACCAGCTGACGGTGTCCAGGATCCGCACGGCTGTGTCCATGCTCAACCGCCGCACGGGCCACGCGCGCTTCCGCCGCGGCCCGGTCGTCGCGGAGCAGCATGCATCAGCGCGCCCCGGTGTCGTGGCGCTGGACTTCGTCAATAAGGCTTGCGAGGCGAGGTTCAGCGCGTCGGCCTCGGGGACCAGCTCGTCGCTGCCGTCGAGCCTCACAGTCACAGCCGGCGAAGGGAGCGTCTCCAACGGCCGCGCTCAGCCTCAGGGTCAGTACCCCTTCCAGCCTGTGAGCGGCGGCGGCGGCAGCGACGGCCACTCTGCCAGGAAGCCGCTGCCGCTGGCGGTATCCATGCAGCAGCAGCAGCATGCCTCCCCCGATCACTCCGCCCCTGCAGGCACCGCGCTTAAAAACGGCAAGTGCCACGACCGCGCGCGCTCCGAGAACGACGCCGGCGGCAAGACGCACGGCCACCGCTGCCACTGCTCTAAGAAACGGAAATCGCGCGTGAAGCGGACGGTCCGCGTGCCGGCGATCAGCTCCCGGAACGCGGACATCCCGGCGGATGACTACTCGTGGCGCAAGTACGGCCAGAAGCCCATCAAGGGATCGCCGTATCCCCGCGGCTACTACAAGTGCAGCACGGTGCGCGGGTGCCCGGCGCGGAAGCACGTGGAGCGAGACCCCGGCGAGCCGGCGATGCTTATCGTCACCTACGAGGGCGACCACCGCCACGACGACCAGCAGCAGGAACGGTCGGCCGGCGGCGCGCAGACGGATCACACGACGACGTCCAGTTGA

>SbWRKY62

ATGGCCGTGGACCTGATGGGCTGCTACGCCCCGCGCCGCGCCGACGACCAGCTCGCCATCCAGGAGGCGGCGGCGGAAAGTCTCCGCAGCCTGGAGCTCCTGGTGTCGTCCCTGTCCACCCAGGCCGGCGCGCCGCACAGGGCCGCTCATCACCTGCAGCAGCAGCAGCCGTTCGGCGAGATCGCCGACCAGGCCGTCTCCAAGTTCCGCAAGGTCATCTCCATCCTGGACCGCACCGGCCACGCCCGCTTCCGCCGCGGGCCCGTCGAGTCCCCGCCGCGGGCGGCCGCGGCGCCTCCGGTCCCCGCTCCCGCTCCGGCTCTCTCCCTGGCTCCGTTGGCTCACGTGGCGCCCGTCAGCGCGGCGCAGCCGGCGCCGGCTTCCCAGCCGCCGCAGAGCCTGACGCTGGACTTCACGAAGCCTAACCTGACCATGTCGGGCGCCACGTCCGTCACCTCCACGTCGTTCTTCTCCTCCGTCACGGCCGGCGAGGGCAGCGTCTCCAAGGGCCGGAGCCTGATGTCCTCCGGCAAGCCGCCGCTGTCCGGCCACAAGCGGAAGCCCTGCGCCGGCGCGCACTCCGAGGCCACCACCAACGGCGGCCGCTGCCACTGCTCCAAGAGAAGGAAAAACCGCGTGAAGAGGACCATCAGAGTGCCGGCGATCAGCTCGAAGATCGCCGACATCCCGCCGGACGAGTACTCGTGGAGGAAGTACGGCCAGAAGCCCATCAAGGGCTCCCCTTACCCACGGGGCTACTACAAGTGCAGCACTGTGCGGGGATGCCCGGCGAGGAAGCACGTGGAGCGCGCCACCGACGACCCGGCCATGCTGGTGGTGACCTACGAGGGCGAGCACCGCCACACGCCCGGAGCGGCCGGGCCCAGCCCCCTGGCGACCGCGTCTCCGGTGGCCGCCGCTGTCTCCGCCGGCAACGGCCATGTCTAG

>SbWRKY64

ATGGCGGTGGACCTGATGTCGTCCTGCGGCGGCCGGGCCGGGGCGTACGAGCAGCTGGCGTTCCAGGAGGCGGCCGCGGCGGGGCTGCGCAGCCTGGAGCTGCTGGCTTCGTCGCTGTCGTCCCCGTGCGGAGCGGGGCAGCGGGCCGAGTCGCCGCCGCTCGGGCAGATCGCGGACCAGGCCGTGTCCCGCTTCCGCCGCGTCATCAACCTGCTGGACCGCACGGGGCACGCCCGCTTCCGCCGCGCGCCCGTCGCCGCGGTGGAGACGGAGACGACGCTCCAGGCCGCGGTGGAGGAGCCGCAGCCGCCTCAGAAGAAGGCCGCCCTGACGCTGGACTTCACCAAGCCGGTCCCGGTCCCGGCGGCGGCGGCGACCAAGCCGGCAGCGCCGGCGCCGGCGCCGGCCGTGTCCGGTACCTCGACCTCGTTCCTGTCGTCCGTGACCGCGGGCGGCGGCGGCGAAGGGAGCGTGTCCAAGGGGTGCAGTCTGGCCGTGTCCTCCGGCAAGCCTCCCCTCCCGAAGCGCAAGCTCCCGTGCCCCGCCTCCGCTCCCCAGCAGGCGCAGGCGCACCAGCACCAGCACCAGCACCAGCACCTCGCGGAGTCGTCCGCCGGGCGTTGCCACTGCTCGAAGAAGAAGCGGAGCCGTCAGGGCCTGTCCCGGCGCACGGTGCGCGTGCCCGCGGCAGCCGCGGCCGCAGGAGCGCCGGGGTCCCACGTGCCGGCGTCGTCGGACATCCCCGCCGACGACTACTCGTGGCGCAAGTACGGGCAGAAGCCCATCAAGGGGTCCCCTTACCCGCGCGGCTACTACCGCTGCAGCAGCGCCAAGGGCTGCCCCGCGCGGAAGCACGTGGAGCGCGCCGCCGACGACCCGGCCATGCTCGTCGTCACCTACGAGGGCGACCACCGCCACGACGCCGCCGCCGCCGCCGTCCGCGCCCGCGCGGCCTGA
