# Supplementary material for: Genome-wide Identification of WRKY transcription factor family members in sorghum (Sorghum bicolor (L.) moench)
Source: PLoS One. 2020 Aug 17;15(8):e0236651. doi: 10.1371/journal.pone.0236651 (PMC7430707; doi:10.1371/journal.pone.0236651)
Supplement: S5 File — (DOCX) [file pone.0236651.s005.docx]

>AT1G13960.1WRKY4 l

MSEKEEAPSTSKSTGAPSRPTLSLPPRPFSEMFFNGGVGFSPGPMTLVSNMFPDSDEFRS

FSQLLAGAMSSPATAAAAAAAATASDYQRLGEGTNSSSGDVDPRFKQNRPTGLMISQSQS

PSMFTVPPGLSPAMLLDSPSFLGLFSPVQGSYGMTHQQALAQVTAQAVQANANMQPQTEY

PPPSQVQSFSSGQAQIPTSAPLPAQRETSDVTIIEHRSQQPLNVDKPADDGYNWRKYGQK

QVKGSEFPRSYYKCTNPGCPVKKKVERSLDGQVTEIIYKGQHNHEPPQNTKRGNKDNTAN

INGSSINNNRGSSELGASQFQTNSSNKTKREQHEAVSQATTTEHLSEASDGEEVGNGETD

VREKDENEPDPKRRSTEVRISEPAPAASHRTVTEPRIIVQTTSEVDLLDDGYRWRKYGQK

VVKGNPYPRSYYKCTTPGCGVRKHVERAATDPKAVVTTYEGKHNHDLPAAKSSSHAAAAA

QLRPDNRPGGLANLNQQQQQQPVARLRLKEEQTT

>AT1G18860.1WRKY61 llb

MDEAKEENRRLKSSLSKIKKDFDILQTQYNQLMAKHNEPTKFQSKGHHQDKGEDEDREKV

NEREELVSLSLGRRLNSEVPSGSNKEEKNKDVEEAEGDRNYDDNEKSSIQGLSMGIEYKA

LSNPNEKLEIDHNQETMSLEISNNNKIRSQNSFGFKNDGDDHEDEDEILPQNLVKKTRVS

VRSRCETPTMNDGCQWRKYGQKIAKGNPCPRAYYRCTIAASCPVRKQVQRCSEDMSILIS

TYEGTHNHPLPMSATAMASATSAAASMLLSGASSSSSAAADLHGLNFSLSGNNITPKPKT

HFLQSPSSSGHPTVTLDLTTSSSSQQPFLSMLNRFSSPPSNVSRSNSYPSTNLNFSNNTN

TLMNWGGGGNPSDQYRAAYGNINTHQQSPYHKIIQTRTAGSSFDPFGRSSSSHSPQINLD

HIGIKNIISHQVPSLPAETIKAITTDPSFQSALATALSSIMGGDLKIDHNVTRNEAEKSP

>AT1G29280.1WRKY65 lle

MKRGLDMARSYNDHESSQETGPESPNSSTFNGMKALISSHSPKRSRRSVEKRVVNVPMKE

MEGSRHKGDTTPPSDSWAWRKYGQKPIKGSPYPRGYYRCSSTKGCPARKQVERSRDDPTM

ILITYTSEHNHPWPLTSSTRNGPKPKPEPKPEPEPEVEPEAEEEDNKFMVLGRGIETTPS

CVDEFAWFTEMETTSSTILESPIFSSEKKTAVSGADDVAVFFPMGEEDESLFADLGELPE

CSVVFRHRSSVVGSQVEIF

>AT1G29860.1WRKY71 llc

MDDHVEHNYNTSLEEVHFKSLSDCLQSSLVMDYNSLEKVFKFSPYSSPFQSVSPSVNNPY

LNLTSNSPVVSSSSNEGEPKENTNDKSDQMEDNEGDLHGVGESSKQLTKQGKKKGEKKER

EVRVAFMTKSEIDHLEDGYRWRKYGQKAVKNSPYPRSYYRCTTQKCNVKKRVERSFQDPS

IVITTYEGKHNHPIPSTLRGTVAAEHLLVHRGGGGSLLHSFPRHHQDFLMMKHSPANYQS

VGSLSYEHGHGTSSYNFNNNQPVVDYGLLQDIVPSMFSKNES

>AT1G30650.1 WRKY14 Glle

MCSVSELLDMENFQGDLTDVVRGIGGHVLSPETPPSNIWPLPLSHPTPSPSDLNINPFGD

PFVSMDDPLLQELNSITNSGYFSTVGDNNNNIHNNNGFLVPKVFEEDHIKSQCSIFPRIR

ISHSNIIHDSSPCNSPAMSAHVVAAAAAASPRGIINVDTNSPRNCLLVDGTTFSSQIQIS

SPRNLGLKRRKSQAKKVVCIPAPAAMNSRSSGEVVPSDLWAWRKYGQKPIKGSPFPRGYY

RCSSSKGCSARKQVERSRTDPNMLVITYTSEHNHPWPIQRNALAGSTRSSTSSSSNPNPS

KPSTANVNSSSIGSQNTIYLPSSTTPPPTLSSSAIKDERGDDMELENVDDDDDNQIAPYR

PELHDHQHQPDDFFADLEELEGDSLSMLLSHGCGGDGKDKTTASDGISNFFGWSGDNNYN

NYDDQDSRSL

>AT1G62300.1 WRKY6 Gllb

MDRGWSGLTLDSSSLDLLNPNRISHKNHRRFSNPLAMSRIDEEDDQKTRISTNGSEFRFP

VSLSGIRDREDEDFSSGVAGDNDREVPGEVDFFSDKKSRVCREDDEGFRVKKEEQDDRTD

VNTGLNLRTTGNTKSDESMIDDGESSEMEDKRAKNELVKLQDELKKMTMDNQKLRELLTQ

VSNSYTSLQMHLVSLMQQQQQQNNKVIEAAEKPEETIVPRQFIDLGPTRAVGEAEDVSNS

SSEDRTRSGGSSAAERRSNGKRLGREESPETESNKIQKVNSTTPTTFDQTAEATMRKARV

SVRARSEAPMISDGCQWRKYGQKMAKGNPCPRAYYRCTMATGCPVRKQVQRCAEDRSILI

TTYEGNHNHPLPPAAVAMASTTTAAANMLLSGSMSSHDGMMNPTNLLARAVLPCSTSMAT

ISASAPFPTVTLDLTHSPPPPNGSNPSSSAATNNNHNSLMQRPQQQQQQMTNLPPGMLPH

VIGQALYNQSKFSGLQFSGGSPSTAAFSQSHAVADTITALTADPNFTAALAAVISSMING

TNHHDGEGNNKNQ

>AT1G66560.1WRKY64 lll

MFSNIDQTAVAALLRGQGCANSLKRLLENHKLSSDSTEPLIYTILNSFSLALSFVDPPSL

LPHNESSLQNMTSHVLQRSSKKKYYGAEDLEYYRDESPTPRPDDGFTWRKYGQKTIKTSP

YQRCYYRCTYAKDQNCNARKRVQMIQDNPPVYRTTYLGKHVCKAVAVHDDTYGSEMIKFD

QVVSESVMPQLATIDEQAITMEDEAIDHIMNQECDINDFSVDDDPFWASQFPPFSSEDIM

FFDNIANLD

AT1G66600.1WRKY63 lll

MFSNIDHKAVAALLHGQGCANILKTVLDNCKVSSVSTEPLINTILDSFSLALSSVNSPNR

QPHHESSSRDMAGLVPQRSSKKKICGVKGLEIYRDDSPNPRLDDGFTWRKYGQKTIKTSL

YQRCYYRCAYAKDQNCYATKRVQMIQDSPPVYRTTYLGQHTCKAFGVHDNTYGSEMINFD

QVVSESVMRQLATIGEQAVLMEDEANHIMNQEYDINDYLVDDEVFWGNEFPLFSSEDLML

F

>AT1G68150.1 WRKY9 Gllb

MGFDFSTSKSKAKRQKRIEVRFASPLMGIDLSLKLEAEEKKKEIEGSKHSRENKEDEEHD

ASGDEDEQMVKEDEDDSSSLGLRTREEENEREELLQLQIQMESVKEENTRLRKLVEQTLE

DYRHLEMKFPVIDKTKKMDLEMFLGVQGKRCVDITSKARKRGAERSPSMEREIGLSLSLE

KKQKQEESKEAVQSHHQRYNSSSLDMNMPRIISSSQGNRKARVSVRARCETATMNDGCQW

RKYGQKTAKGNPCPRAYYRCTVAPGCPVRKQVQRCLEDMSILITTYEGTHNHPLPVGATA

MASTASTSPFLLLDSSDNLSHPSYYQTPQAIDSSLITYPQNSSYNNRTIRSLNFDGPSRG

DHVSSSQNRLNWMM

>AT1G69310.1WRKY57 llc

MNDPDNPDLSNDDSAWRELTLTAQDSDFFDRDTSNILSDFGWNLHHSSDHPHSLRFDSDL

TQTTGVKPTTVTSSCSSSAAVSVAVTSTNNNPSATSSSSEDPAENSTASAEKTPPPETPV

KEKKKAQKRIRQPRFAFMTKSDVDNLEDGYRWRKYGQKAVKNSPFPRSYYRCTNSRCTVK

KRVERSSDDPSIVITTYEGQHCHQTIGFPRGGILTAHDPHSFTSHHHLPPPLPNPYYYQE

LLHQLHRDNNAPSPRLPRPTTEDTPAVSTPSEEGLLGDIVPQTMRNP

>AT1G69810.1WRKY36 llb

MIKEETVSYFQTFDGVMAESDKEEELDATKAKVEKVREENEKLKLLLSTILNNYNSLQMQ

VSKVLGQQQGASSMELDHIDRQDENNDYDVDISLRLGRSEQKISKKEENKVDKISTKNVE

ESKDKRSALGFGFQIQSYEASKLDDLCRQVKLANAENKCVSSRKDVKSVRNENHQDVLEE

HEQTGLKKTRVCVKASCEDPSINDGCQWRKYGQKTAKTNPLPRAYYRCSMSSNCPVRKQV

QRCGEEETSAFMTTYEGNHDHPLPMEASHMAAGTSAAASLLQSGSSSSSSSTSASLSYFF

PFHHFSISTTNSHPTVTLDLTRPNYPNQLPDDYPLSSSSFSLNFSSPDPPPPSSHDHTLN

FSGLRTQAPLSTDSLLARYRTRLSGQQ

AT1G80590.1WRKY66 lll

MSLEIDAKAVSALLLGQGCANNLKTLLKNHETGSVSTEPLINSILDSFSFALSSQNIPRH

VSQRSSKKKMCGIQGMEDSPTPAHIDGFIWRKYGQKTIKTSPHQRWYYRCAYAKDQNCDA

TKRVQKIQDNPPVYRNTYVGQHACEAPAYAVNNGGTYGSKMIKFDYVIPESVMPQPLSID

SQEITMEDKDTDDHILNYINEHLMEDEAYDVFPDVLGERCCFGLEPFPGLNINKS

>AT1G80840.1WRKY40 lla

MDQYSSSLVDTSLDLTIGVTRMRVEEDPPTSALVEELNRVSAENKKLSEMLTLMCDNYNV

LRKQLMEYVNKSNITERDQISPPKKRKSPAREDAFSCAVIGGVSESSSTDQDEYLCKKQR

EETVVKEKVSRVYYKTEASDTTLVVKDGYQWRKYGQKVTRDNPSPRAYFKCACAPSCSVK

KKVQRSVEDQSVLVATYEGEHNHPMPSQIDSNNGLNRHISHGGSASTPVAANRRSSLTVP

VTTVDMIESKKVTSPTSRIDFPQVQKLLVEQMASSLTKDPNFTAALAAAVTGKLYQQNHT

EK

>AT2G03340.1 WRKY3 1

MAEKEEKEPSKLKSSTGVSRPTISLPPRPFGEMFFSGGVGFSPGPMTLVSNLFSDPDEFK

SFSQLLAGAMASPAAAAVAAAAVVATAHHQTPVSSVGDGGGSGGDVDPRFKQSRPTGLMI

TQPPGMFTVPPGLSPATLLDSPSFFGLFSPLQGTFGMTHQQALAQVTAQAVQGNNVHMQQ

SQQSEYPSSTQQQQQQQQQASLTEIPSFSSAPRSQIRASVQETSQGQRETSEISVFEHRS

QPQNADKPADDGYNWRKYGQKQVKGSDFPRSYYKCTHPACPVKKKVERSLDGQVTEIIYK

GQHNHELPQKRGNNNGSCKSSDIANQFQTSNSSLNKSKRDQETSQVTTTEQMSEASDSEE

VGNAETSVGERHEDEPDPKRRNTEVRVSEPVASSHRTVTEPRIIVQTTSEVDLLDDGYRW

RKYGQKVVKGNPYPRSYYKCTTPDCGVRKHVERAATDPKAVVTTYEGKHNHDVPAARTSS

HQLRPNNQHNTSTVNFNHQQPVARLRLKEEQIT

>AT2G04880.WRYKY1 Gl

MAEVGKVLASDMELDHSNETKAVDDVVATTDKAEVIPVAVTRTETVVESLESTDCKELEK

LVPHTVASQSEVDVASPVSEKAPKVSESSGALSLQSGSEGNSPFIREKVMEDGYNWRKYG

QKLVKGNEFVRSYYRCTHPNCKAKKQLERSAGGQVVDTVYFGEHDHPKPLAGAVPINQDK

RSDVFTAVSKGEQRIDIVSLIYKLCIVSYDIMFVEKTSGSSVQTLRQTEPPKIHGGLHVS

VIPPADDVKTDISQSSRITGDNTHKDYNSPTAKRRKKGGNIELSPVERSTNDSRIVVHTQ

TLFDIVNDGYRWRKYGQKSVKGSPYPRSYYRCSSPGCPVKKHVERSSHDTKLLITTYEGK

HDHDMPPGRVVTHNNMLDSEVDDKEGDANKTPQSSTLQSITKDQHVEDHLRKKTKTNGFE

KSLDQGPVLDEKLKEEIKERSDANKDHAANHAKPEAKSDDKTTVCQEKAVGTLESEEQKP

KTEPAQS

>AT2G21900.1WRKY59 llc

MNYPSNPNPSSTDFTEFFKFDDFDDTFEKIMEEIGREDHSSSPTLSWSSSEKLVAAEITS

PLQTSLATSPMSFEIGDKDEIKKRKRHKEDPIIHVFKTKSSIDEKVALDDGYKWRKYGKK

PITGSPFPRHYHKCSSPDCNVKKKIERDTNNPDYILTTYEGRHNHPSPSVVYCDSDDFDL

NSLNNWSFQTANTYSFSHSAPY

>AT2G23320.1WRKY15 lld

MAVELMTRNYISGVGADSFAVQEAAASGLKSIENFIGLMSRDSFNSDQPSSSSASASASA

AADLESARNTTADAAVSKFKRVISLLDRTRTGHARFRRAPVHVISPVLLQEEPKTTPFQS

PLPPPPQMIRKGSFSSSMKTIDFSSLSSVTTESDNQKKIHHHQRPSETAPFASQTQSLST

TVSSFSKSTKRKCNSENLLTGKCASASSSGRCHCSKKRKIKQRRIIRVPAISAKMSDVPP

DDYSWRKYGQKPIKGSPHPRGYYKCSSVRGCPARKHVERAADDSSMLIVTYEGDHNHSLS

AADLAGAAVADLILESS

>AT2G24570.1 WRKY17 lld

MTVDIMRLPKMEDQTAIQEAASQGLKSMEHLIRVLSNRPEERNVDCSEITDFTVSKFKKV

ISLLNRSGHARFRRGPVHSPPSSSVPPPVKVTTPAPTQISAPAPVSFVQANQQSVTLDFT

RPSVFGAKTKSSEVVEFAKESFSVSSNSSFMSSAITGDGSVSKGSSIFLAPAPAVPVTSS

GKPPLSGLPYRKRCFEHDHSEGFSGKISGSGNGKCHCKKSRKNRMKRTVRVPAVSAKIAD

IPPDEYSWRKYGQKPIKGSPHPRGYYKCSTFRGCPARKHVERALDDSTMLIVTYEGEHRH

HQSTMQEHVTPSVSGLVFGSA

>AT2G25000.1WRKY60 lla

MDYDPNTNPFDLHFSGKLPKREVSASASKVVEKKWLVKDEKRNMLQDEINRVNSENKKLT

EMLARVCEKYYALNNLMEELQSRKSPESVNFQNKQLTGKRKQELDEFVSSPIGLSLGPIE

NITNDKATVSTAYFAAEKSDTSLTVKDGYQWRKYGQKITRDNPSPRAYFRCSFSPSCLVK

KKVQRSAEDPSFLVATYEGTHNHTGPHASVSRTVKLDLVQGGLEPVEEKKERGTIQEVLV

QQMASSLTKDPKFTAALATAISGRLIEHSRT

>AT2G30250.1WRKY25 l

MSSTSFTDLLGSSGVDCYEDDEDLRVSGSSFGGYYPERTGSGLPKFKTAQPPPLPISQSS

HNFTFSDYLDSPLLLSSSHSLISPTTGTFPLQGFNGTTNNHSDFPWQLQSQPSNASSALQ

ETYGVQDHEKKQEMIPNEIATQNNNQSFGTERQIKIPAYMVSRNSNDGYGWRKYGQKQVK

KSENPRSYFKCTYPDCVSKKIVETASDGQITEIIYKGGHNHPKPEFTKRPSQSSLPSSVN

GRRLFNPASVVSEPHDQSENSSISFDYSDLEQKSFKSEYGEIDEEEEQPEMKRMKREGED

EGMSIEVSKGVKEPRVVVQTISDIDVLIDGFRWRKYGQKVVKGNTNPRSYYKCTFQGCGV

KKQVERSAADERAVLTTYEGRHNHDIPTALRRS

>AT2G30590.1 WRKY21 lld

MEEIEGTNRAAVESCHRVLNLLHRSQQQDHVGFEKNLVSETREAVIRFKRVGSLLSSSVG

HARFRRAKKLQSHVSQSLLLDPCQQRTTEVPSSSSQKTPVLRSGFQELSLRQPSDSLTLG

TRSFSLNSNAKAPLLQLNQQTMPPSNYPTLFPVQQQQQQQQQQQQQEQQQQQQQQQQQFH

ERLQAHHLHQQQQLQKHQAELMLRKCNGGISLSFDNSSCTPTMSSTRSFVSSLSIDGSVA

NIEGKNSFHFGVPSSTDQNSLHSKRKCPLKGDEHGSLKCGSSSRCHCAKKRKHRVRRSIR

VPAISNKVADIPPDDYSWRKYGQKPIKGSPYPRGYYKCSSMRGCPARKHVERCLEDPAML

IVTYEAEHNHPKLPSQAITT

>AT2G34830.1WRKY35 lle

MDNFQGDLTDVVRGIGSGHVSPSPGPPEGPSPSSMSPPPTSDLHVEFPSAATSASCLANP

FGDPFVSMKDPLIHLPASYISGAGDNKSNKSFAIFPKIFEDDHIKSQCSVFPRIKISQSN

NIHDASTCNSPAITVSSAAVAASPWGMINVNTTNSPRNCLLVDNNNNTSSCSQVQISSSP

RNLGIKRRKSQAKKVVCIPAPAAMNSRSSGEVVPSDLWAWRKYGQKPIKGSPYPRGYYRC

SSSKGCSARKQVERSRTDPNMLVITYTSEHNHPWPTQRNALAGSTRSSSSSSLNPSSKSS

TAAATTSPSSRVFQNNSSKDEPNNSNLPSSSTHPPFDAAAIKEENVEERQEKMEFDYNDV

ENTYRPELLQEFQHQPEDFFADLDELEGDSLTMLLSHSSGGGNMENKTTIPDVFSDFFDD

DESSRSL

>AT2G37260.1WRKY44 l

MEVNDGERVVIAKPVASRPSSSSGFRTFTELLTDSVTVSPQTTCHEIVDAAIRPKTLRFN

QPVAASVSCPRAEVKGIGNGMSCDDDSDSRNYVVYKPKAKLVSKATVSALANMLQGNRQQ

TWRQSEAVSYGKSVSQGTHRAGPNLVQKVPSFTESETSTGDRSSVDGYNWRKYGQKQVKG

SECPRSYYKCTHPKCPVKKKVERSVEGQVSEIVYQGEHNHSKPSCPLPRRASSSISSGFQ

KPPKSIASEGSMGQDPNNNLYSPLWNNQSNDSTQNRTEKMSEGCVITPFEFAVPRSTNSN

PGTSDSGCKSSQCDEGELDDPSRSKRRKNEKQSSEAGVSQGSVESDSLEDGFRWRKYGQK

VVGGNAYPRSYYRCTSANCRARKHVERASDDPRAFITTYEGKHNHHLLLSPPSSSTLPFN

SPQLSKQTI

>AT2G38470.1WRKY33 l

MAASFLTMDNSRTRQNMNGSANWSQQSGRTSTSSLEDLEIPKFRSFAPSSISISPSLVSP

STCFSPSLFLDSPAFVSSSANVLASPTTGALITNVTNQKGINEGDKSNNNNFNLFDFSFH

TQSSGVSAPTTTTTTTTTTTTTNSSIFQSQEQQKKNQSEQWSQTETRPNNQAVSYNGREQ

RKGEDGYNWRKYGQKQVKGSENPRSYYKCTFPNCPTKKKVERSLEGQITEIVYKGSHNHP

KPQSTRRSSSSSSTFHSAVYNASLDHNRQASSDQPNSNNSFHQSDSFGMQQEDNTTSDSV

GDDEFEQGSSIVSRDEEDCGSEPEAKRWKGDNETNGGNGGGSKTVREPRIVVQTTSDIDI

LDDGYRWRKYGQKVVKGNPNPRSYYKCTTIGCPVRKHVERASHDMRAVITTYEGKHNHDV

PAARGSGYATNRAPQDSSSVPIRPAAIAGHSNYTTSSQAPYTLQMLHNNNTNTGPFGYAM

NNNNNNSNLQTQQNFVGGGFSRAKEEPNEETSFFDSFMP

>AT2G40740.1WRKY55 lll

MYSYKKISYQMEEVMSMIFHGMKLVKSLESSLPEKPPESLLTSLDEIVKTFSDANERLKM

LLEIKNSETALNKTKPVIVSVANQMLMQMEPGLMQEYWLRYGGSTSSQGTEAMFQTQLMA

VDGGGERNLTAAVERSGASGSSTPRQRRRKDEGEEQTVLVAALRTGNTDLPPDDNHTWRK

YGQKEILGSRFPRAYYRCTHQKLYNCPAKKQVQRLNDDPFTFRVTYRGSHTCYNSTAPTA

SSATPSTIPISSVTTGHSVDYGLAVVDMADVMFGSGGVGTNMDFIFPKNDPS

>AT2G40750.1WRKY54 lll

MDSNSNNTKSIKRKVVDQLVEGYEFATQLQLLLSHQHSNQYHIDETRLVSGSGSVSGGPD

PVDELMSKILGSFHKTISVLDSFDPVAVSVPIAVEGSWNASCGDDSATPVSCNGGDSGES

KKKRLGVGKGKRGCYTRKTRSHTRIVEAKSSEDRYAWRKYGQKEILNTTFPRSYFRCTHK

PTQGCKATKQVQKQDQDSEMFQITYIGYHTCTANDQTHAKTEPFDQEIIMDSEKTLAAST

AQNHVNAMVQEQENNTSSVTAIDAGMVKEEQNNNGDQSKDYYEGSSTGEDLSLVWQETMM

FDDHQNHYYCGETSTTSHQFGFIDNDDQFSSFFDSYCADYERTSAM

>AT2G44745.1 WRKY12 Gllc

MEGGGRRVFSNYDLQQVTSSSTTIQENMNFLVPFEETNVLTFFSSSSSSSLSSPSFPIHN

SSSTTTTHAPLGFSNNLQGGGPLGSKVVNDDQENFGGGTNNDAHSNSWWRSNSGSGDMKN

KVKIRRKLREPRFCFQTKSDVDVLDDGYKWRKYGQKVVKNSLHPRSYYRCTHNNCRVKKR

VERLSEDCRMVITTYEGRHNHIPSDDSTSPDHDCLSSF

>AT2G46130.1WRKY43 llc

MNGLVDSSRDKKMKNPRFSFRTKSDADILDDGYRWRKYGQKSVKNSLYPRSYYRCTQHMC

NVKKQVQRLSKETSIVETTYEGIHNHPCEELMQTLTPLLHQLQFLSKFT

>AT2G46400.1WRKY46 lll

MMMEEKLVINELELGKELANRLMNNLKHTSSVDSNKTLISDILRIYQNAIFMLSFNQDKN

ILKRSLEIDGKDSKNVFKKRKVSEKNTEKVKVFVATEQENGSIDDGHCWRKYGQKEIHGS

KNPRAYYRCTHRFTQDCLAVKQVQKSDTDPSLFEVKYLGNHTCNNITSPKTTTNFSVSLT

NTNIFEGNRVHVTEQSEDMKPTKSEEVMISLEDLENKKNIFRTFSFSNHEIENGVWKSNL

FLGNFVEDLSPATSGSAITSEVLSAPAAVENSETADSYFSSLDNIIDFGQDWLWS

>AT2G47260.1 WRKY23 llc

MEFTDFSKTSFYYPSSQSVWDFGDLAAAERHSLGFMELLSSQQHQDFATVSPHSFLLQTS

QPQTQTQPSAKLSSSIIQAPPSEQLVTSKVESLCSDHLLINPPATPNSSSISSASSEALN

EEKPKTEDNEEEGGEDQQEKSHTKKQLKAKKNNQKRQREARVAFMTKSEVDHLEDGYRWR

KYGQKAVKNSPFPRSYYRCTTASCNVKKRVERSFRDPSTVVTTYEGQHTHISPLTSRPIS

TGGFFGSSGAASSLGNGCFGFPIDGSTLISPQFQQLVQYHHQQQQQELMSCFGGVNEYLN

SHANEYGDDNRVKKSRVLVKDNGLLQDVVPSHMLKEE

>AT3G01080.1WRKY58 l

MAVEDDVSLIRTTTLVAPTRPTITVPHRPPAIETAAYFFGGGDGLSLSPGPLSFVSSLFV

DNFPDVLTPDNQRTTSFTHLLTSPMFFPPQSSAHTGFIQPRQQSQPQPQRPDTFPHHMPP

STSVAVHGRQSLDVSQVDQRARNHYNNPGNNNNNRSYNVVNVDKPADDGYNWRKYGQKPI

KGCEYPRSYYKCTHVNCPVKKKVERSSDGQITQIIYKGQHDHERPQNRRGGGGRDSTEVG

GAGQMMESSDDSGYRKDHDDDDDDDEDDEDLPASKIRRIDGVSTTHRTVTEPKIIVQTKS

EVDLLDDGYRWRKYGQKVVKGNPHPRSYYKCTTPNCTVRKHVERASTDAKAVITTYEGKH

NHDVPAARNGTAAATAAAVGPSDHHRMRSMSGNNMQQHMSFGNNNNTGQSPVLLRLKEEK

ITI

>AT3G04670.1WRKY39 lld

MEEVEAANRSAIESCHGVLNLLSQRTSDPKSLTVETGEVVSKFKRVASLLTRGLGHGKFR

STNKFRSSFPQHIFLESPICCGNDLSGDYTQVLAPEPLQMVPASAVYNEMEPKHQLGHPS

LMLSHKMCVDKSFLELKPPPFRAPYQLIHNHQQIAYSRSNSGVNLKFDGSGSSCYTPSVS

NGSRSFVSSLSMDASVTDYDRNSFHLTGLSRGSDQQHTRKMCSGSLKCGSRSKCHCSKKR

KLRVKRSIKVPAISNKIADIPPDEYSWRKYGQKPIKGSPHPRGYYKCSSVRGCPARKHVE

RCIDETSMLIVTYEGEHNHSRILSSQSAHT

>AT3G56400.1WRKY70 lll

MDTNKAKKLKVMNQLVEGHDLTTQLQQLLSQPGSGLEDLVAKILVCFNNTISVLDTFEPI

SSSSSLAAVEGSQNASCDNDGKFEDSGDSRKRLGPVKGKRGCYKRKKRSETCTIESTILE

DAFSWRKYGQKEILNAKFPRSYFRCTHKYTQGCKATKQVQKVELEPKMFSITYIGNHTCN

TNAETPKSKTCDHHDEIFMDSEDHKSPSLSTSMKEEDNPHRHHGSSTENDLSLVWPEMVF

EEDYHHQASYVNGKTSTSIDVLGSQDLMVFGGGGDFEFSENEHFSIFSSCSNLS

>AT3G58710.1WRKY69 lle

MHRRAAIQESDDEEDETYNDVVPESPSSCEDSKISKPTPKKSRRNVEKRVVSVPIADVEG

SKSRGEVYPPSDSWAWRKYGQKPIKGSPYPRGYYRCSSSKGCPARKQVERSRVDPSKLMI

TYACDHNHPFPSSSANTKSHHRSSVVLKTAKKEEEYEEEEEELTVTAAEEPPAGLDLSHV

DSPLLLGGCYSEIGEFGWFYDASISSSSGSSNFLDVTLERGFSVGQEEDESLFGDLGDLP

DCASVFRRGTVATEEQHRRCDFGAIPFCDSSR

>AT3G62340.1WRKY68 llc

MENVGVGMPFYDLGQTRVYPLLSDFHDLSAERYPVGFMDLLGVHRHTPTHTPLMHFPTTP

NSSSSEAVNGDDEEEEDGEEQQHKTKKRFKFTKMSRKQTKKKVPKVSFITRSEVLHLDDG

YKWRKYGQKPVKDSPFPRNYYRCTTTWCDVKKRVERSFSDPSSVITTYEGQHTHPRPLLI

MPKEGSSPSNGSASRAHIGLPTLPPQLLDYNNQQQQAPSSFGTEYINRQEKGINHDDDDD

HVVKKSRTRDLLDGAGLVKDHGLLQDVVPSHIIKEEY

>AT4G01250.1 WRKY22 lle

MADDWDLHAVVRGCSAVSSSATTTVYSPGVSSHTNPIFTVGRQSNAVSFGEIRDLYTPFT

QESVVSSFSCINYPEEPRKPQNQKRPLSLSASSGSVTSKPSGSNTSRSKRRKIQHKKVCH

VAAEALNSDVWAWRKYGQKPIKGSPYPRGYYRCSTSKGCLARKQVERNRSDPKMFIVTYT

AEHNHPAPTHRNSLAGSTRQKPSDQQTSKSPTTTIATYSSSPVTSADEFVLPVEDHLAVG

DLDGEEDLLSLSDTVVSDDFFDGLEEFAAGDSFSGNSAPASFDLSWVVNSAATTTGGI

>AT4G01720.1WRKY47 llb

MEEHIQDRREIAFLHSGEFLHGDSDSKDHQPNESPVERHHESSIKEVDFFAAKSQPFDLG

HVRTTTIVGSSGFNDGLGLVNSCHGTSSNDGDDKTKTQISRLKLELERLHEENHKLKHLL

DEVSESYNDLQRRVLLARQTQVEGLHHKQHEDVPQAGSSQALENRRPKDMNHETPATTLK

RRSPDDVDGRDMHRGSPKTPRIDQNKSTNHEEQQNPHDQLPYRKARVSVRARSDATTVND

GCQWRKYGQKMAKGNPCPRAYYRCTMAVGCPVRKQVQRCAEDTTILTTTYEGNHNHPLPP

SATAMAATTSAAAAMLLSGSSSSNLHQTLSSPSATSSSSFYHNFPYTSTIATLSASAPFP

TITLDLTNPPRPLQPPPQFLSQYGPAAFLPNANQIRSMNNNNQQLLIPNLFGPQAPPREM

VDSVRAAIAMDPNFTAALAAAISNIIGGGNNDNNNNTDINDNKVDAKSGGSSNGDSPQLP

QSCTTFSTN

>AT4G04450.1WRKY42 llb

MFRFPVSLGGGPRENLKPSDEQHQRAVVNEVDFFRSAEKRDRVSREEQNIIADETHRVHV

KRENSRVDDHDDRSTDHINIGLNLLTANTGSDESMVDDGLSVDMEEKRTKCENAQLREEL

KKASEDNQRLKQMLSQTTNNFNSLQMQLVAVMRQQEDHHHLATTENNDNVKNRHEVPEMV

PRQFIDLGPHSDEVSSEERTTVRSGSPPSLLEKSSSRQNGKRVLVREESPETESNGWRNP

NKVPKHHASSSICGGNGSENASSKVIEQAAAEATMRKARVSVRARSEAPMLSDGCQWRKY

GQKMAKGNPCPRAYYRCTMAVGCPVRKQVQRCAEDRTILITTYEGNHNHPLPPAAMNMAS

TTTAAASMLLSGSTMSNQDGLMNPTNLLARTILPCSSSMATISASAPFPTITLDLTESPN

GNNPTNNPLMQFSQRSGLVELNQSVLPHMMGQALYYNQQSKFSGLHMPSQPLNAGESVSA

ATAAIASNPNFAAALAAAITSIINGSNNQQNGNNNNSNVTTSNVDNRQ

>AT4G11070.1WRKY41 lll

MEMMNWERRSLLNELIHGLKAAKQLQGSSSPSLSASSSYLTTEIKENLLHNIVSSFKKAI

LMLNGSTTQHNPTIELAPDPLAHPGKVPGSPASITGNPRSEEFFNVRSKEFNLSSKKRKM

LPKWTEQVRISPERGLEGPHDDIFSWRKYGQKDILGAKFPRSYYRCTFRNTQYCWATKQV

QRSDGDPTIFEVTYRGTHTCSQGIPLPEKRETKPKHTVAVNYQNLRASLTVRTGGLGSEA

FSFPVTSPLYTYESINGGGTFYHHVGSSGPSDFTGLISTNTSTGSSPIFDVNFQFDPTAE

INTGFPTFFHNSI

>AT4G18170.1WRKY28 llc

MSNETRDLYNYQYPSSFSLHEMMNLPTSNPSSYGNLPSQNGFNPSTYSFTDCLQSSPAAY

ESLLQKTFGLSPSSSEVFNSSIDQEPNRDVTNDVINGGACNETETRVSPSNSSSSEADHP

GEDSGKSRRKRELVGEEDQISKKVGKTKKTEVKKQREPRVSFMTKSEVDHLEDGYRWRKY

GQKAVKNSPYPRSYYRCTTQKCNVKKRVERSFQDPTVVITTYEGQHNHPIPTNLRGSSAA

AAMFSADLMTPRSFAHDMFRTAAYTNGGSVAAALDYGYGQSGYGSVNSNPSSHQVYHQGG

EYELLREIFPSIFFKQEP

>AT4G22070.1WRKY31llb

MFRFPVSLGGSRDEDRHDQITPLDDHRVVVDEVDFFSEKRDRVSRENINDDDDEGNKVLI

KMEGSRVEENDRSRDVNIGLNLLTANTGSDESTVDDGLSMDMEDKRAKIENAQLQEELKK

MKIENQRLRDMLSQATTNFNALQMQLVAVMRQQEQRNSSQDHLLAQESKAEGRKRQELQI

MVPRQFMDLGPSSGAAEHGAEVSSEERTTVRSGSPPSLLESSNPRENGKRLLGREESSEE

SESNAWGNPNKVPKHNPSSSNSNGNRNGNVIDQSAAEATMRKARVSVRARSEAAMISDGC

QWRKYGQKMAKGNPCPRAYYRCTMAGGCPVRKQVQRCAEDRSILITTYEGNHNHPLPPAA

TAMASTTTAAASMLLSGSMSSQDGLMNPTNLLARAILPCSSSMATISASAPFPTITLDLT

NSPNGNNPNMTTNNPLMQFAQRPGFNPAVLPQVVGQAMYNNQQQSKFSGLQLPAQPLQIA

ATSSVAESVSAASAAIASDPNFAAALAAAITSIMNGSSHQNNNTNNNNVATSNNDSRQ

>AT4G23550.1 WRKY29 lle

MDEGDLEAIVRGYSGSGDAFSGESSGTFSPSFCLPMETSSFYEPEMETSGLDELGELYKP

FYPFSTQTILTSSVSLPEDSKPFRDDKKQRSHGCLLSNGSRADHIRISESKSKKSKKNQQ

KRVVEQVKEENLLSDAWAWRKYGQKPIKGSPYPRSYYRCSSSKGCLARKQVERNPQNPEK

FTITYTNEHNHELPTRRNSLAGSTRAKTSQPKPTLTKKSEKEVVSSPTSNPMIPSADESS

VAVQEMSVAETSTHQAAGAIEGRRLSNGLPSDLMSGSGTFPSFTGDFDELLNSQEFFSGY

LWNY

>AT4G23810.1WRKY53 lll

MEGRDMLSWEQKTLLSELINGFDAAKKLQARLREAPSPSSSFSSPATAVAETNEILVKQI

VSSYERSLLLLNWSSSPSVQLIPTPVTVVPVANPGSVPESPASINGSPRSEEFADGGGSS

ESHHRQDYIFNSKKRKMLPKWSEKVRISPERGLEGPQDDVFSWRKYGQKDILGAKFPRSY

YRCTHRSTQNCWATKQVQRSDGDATVFEVTYRGTHTCSQAITRTPPLASPEKRQDTRVKP

AITQKPKDILESLKSNLTVRTDGLDDGKDVFSFPDTPPFYNYGTINGEFGHVESSPIFDV

VDWFNPTVEIDTTFPAFLHESIYY

>AT4G24240.1 WRKY7 G lld

MTVELMMSSYSGGGGGGDGFPAIAAAAKMEDTALREAASAGIHGVEEFLKLIGQSQQPTE

KSQTEITAVTDVAVNSFKKVISLLGRSRTGHARFRRAPASTQTPFKQTPVVEEEVEVEEK

KPETSSVLTKQKTEQYHGGGSAFRVYCPTPIHRRPPLSHNNNNNQNQTKNGSSSSSPPML

ANGAPSTINFAPSPPVSATNSFMSSHRCDTDSTHMSSGFEFTNPSQLSGSRGKPPLSSAS

LKRRCNSSPSSRCHCSKKRKSRVKRVIRVPAVSSKMADIPSDEFSWRKYGQKPIKGSPHP

RGYYKCSSVRGCPARKHVERALDDAMMLIVTYEGDHNHALVLETTTMNHDKTL

>AT4G26440.1WRKY34 l

MAGIDNKAAVMGEWFDCSTTNHRKRSKAELGREFSLNYIKNEDSLQTTFQESSRGALRER

IAARSGFNAPWLNTEDILQSKSLTISSPGLSPATLLESPVFLSNPLLSPTTGKLSSVPSD

KAKAELFDDITTSLAFQTISGSGLDPTNIALEPDDSQDYEERQLGGLGDSMACCAPADDG

YNWRKYGQKLVKGSEYPRSYYKCTHPNCEAKKKVERSREGHIIEIIYTGDHIHSKPPPNR

RSGIGSSGTGQDMQIDATEYEGFAGTNENIEWTSPVSAELEYGSHSGSMQVQNGTHQFGY

GDAAADALYRDENEDDRTSHMSVSLTYDGEVEESESKRRKLEAYATETSGSTRASREPRV

VVQTTSDIDILDDGYRWRKYGQKVVKGNPNPRSYYKCTANGCTVTKHVERASDDFKSVLT

TYIGKHTHVVPAARNSSHVGAGSSGTLQGSLATQTHNHNVHYPMPHSRSEGLATANSSLF

DFQSHLRHPTGFSVYIGQSELSDLSMPGLTIGQEKLTSLQAPDIGDPTGLMLQLAAQPKV

EPVSPQQGLDLSASSLICREMLSRLRQI

>AT4G26640.2 WRKY20 l

MNPQANDRKEFQGDCSATGDLTAKHDSAGGNGGGGARYKLMSPAKLPISRSTDITIPPGL

SPTSFLESPVFISNIKPEPSPTTGSLFKPRPVHISASSSSYTGRGFHQNTFTEQKSSEFE

FRPPASNMVYAELGKIRSEPPVHFQGQGHGSSHSPSSISDAAGSSSELSRPTPPCQMTPT

SSDIPAGSDQEESIQTSQNDSRGSTPSILADDGYNWRKYGQKHVKGSEFPRSYYKCTHPN

CEVKKLFERSHDGQITDIIYKGTHDHPKPQPGRRNSGGMAAQEERLDKYPSSTGRDEKGS

GVYNLSNPNEQTGNPEVPPISASDDGGEAAASNRNKDEPDDDDPFSKRRRMEGAMEITPL

VKPIREPRVVVQTLSEVDILDDGYRWRKYGQKVVRGNPNPRSYYKCTAHGCPVRKHVERA

SHDPKAVITTYEGKHDHDVPTSKSSSNHEIQPRFRPDETDTISLNLGVGISSDGPNHASN

EHQHQNQQLVNQTHPNGVNFRFVHASPMSSYYASLNSGMNQYGQRETKNETQNGDISSLN

NSSYPYPPNMGRVQSGP

>AT4G30935.1WRKY32 l

MEEDTGIDEAKTYTVEKSEKVEPEKDGLSQFRDEEKSLGADMEDLHDETVRETLGKDQVQ

GVRENSSVEPNVEDVLEVNETDSVKETVVSAIVPVDEVEENRQVETSPSLAASSDSLTVT

PCLSLDPATASTAQDLPLVSVPTKQEQRSDSPVVNRLSVTPVPRTPARDGYNWRKYGQKQ

VKSPKGSRSYYRCTYTECCAKKIECSNDSGNVVEIVNKGLHTHEPPRKTSFSPREIRVTT

AIRPVSEDDTVVEELSIVPSGSDPSASTKEYICESQTLVDRKRHCENEAVEEPEPKRRLK

KDNSQSSDSVSKPGKKNKFVVHAAGDVGICGDGYRWRKYGQKMVKGNPHPRNYYRCTSAG

CPVRKHIETAVENTKAVIITYKGVHNHDMPVPKKRHGPPSSMLVAAAAPTSMRTRTDDQV

NIPTSSQCSVGRESEKQSKEALDVGGEKVMESARTLLSIGFEIKQC

>AT4G31550.1 WRKY11 G lld

MAVDLMRFPKIDDQTAIQEAASQGLQSMEHLIRVLSNRPEQQHNVDCSEITDFTVSKFKT

VISLLNRTGHARFRRGPVHSTSSAASQKLQSQIVKNTQPEAPIVRTTTNHPQIVPPPSSV

TLDFSKPSIFGTKAKSAELEFSKENFSVSLNSSFMSSAITGDGSVSNGKIFLASAPLQPV

NSSGKPPLAGHPYRKRCLEHEHSESFSGKVSGSAYGKCHCKKSRKNRMKRTVRVPAISAK

IADIPPDEYSWRKYGQKPIKGSPHPRGYYKCSTFRGCPARKHVERALDDPAMLIVTYEGE

HRHNQSAMQENISSSGINDLVFASA

>AT4G31800.1WRKY18 lla

MDGSSFLDISLDLNTNPFSAKLPKKEVSVLASTHLKRKWLEQDESASELREELNRVNSEN

KKLTEMLARVCESYNELHNHLEKLQSRQSPEIEQTDIPIKKRKQDPDEFLGFPIGLSSGK

TENSSSNEDHHHHHQQHEQKNQLLSCKRPVTDSFNKAKVSTVYVPTETSDTSLTVKDGFQ

WRKYGQKVTRDNPSPRAYFRCSFAPSCPVKKKVQRSAEDPSLLVATYEGTHNHLGPNASE

GDATSQGGSSTVTLDLVNGCHRLALEKNERDNTMQEVLIQQMASSLTKDSKFTAALAAAI

SGRLMEQSRT

>AT4G39410.1 WRKY13 G llc

MGAINQGISLFDESQTVINPINTNHLGFFFSFPSHSTLSSSSSSSSSSPSSLVSPFLGHN

SLNSFLHNNPSSFISHPQDSINLMTNLPETLISSLSSSKQRDDHDGFLNLDHHRLTGSIS

SQRPLSNPWAWSCQAGYGSSQKNNHGSEIDVDDNDDEVGDGGGINDDDNGRHHHHDTPSR

HDKHNTASLGVVSSLKMKKLKTRRKVREPRFCFKTLSEVDVLDDGYRWRKYGQKVVKNTQ

HPRSYYRCTQDKCRVKKRVERLADDPRMVITTYEGRHLHSPSNHLDDDSLSTSHLHPPLS

NFFW

>AT5G01900.1WRKY62 lll

MNSCQQKAMEKLLHGHGCANQLLIMDQTESDSSMEREDLAKSVLHCFSDALSILIDTNDH

QDDQSNNSSPQDSSPVLESSRKPLHKRGRKTSMAESSDYHRHESSTPIYHDGFLWRKYGQ

KQIKESEYQRSYYKCAYTKDQNCEAKKQVQKIQHNPPLYSTTYFGQHICQLHQAYATFPI

DTSDFEEHEGSHMIRFGHPNISFSSSTSNLRQHQNHQDRIKDEYMKPVIAEDWSPSQWMS

SEVALAVEAFEFNPFWTSHDLSS

>AT5G07100.WRKY26 l

MGSFDRQRAVPKFKTATPSPLPLSPSPYFTMPPGLTPADFLDSPLLFTSSNILPSPTTGT

FPAQSLNYNNNGLLIDKNEIKYEDTTPPLFLPSMVTQPLPQLDLFKSEIMSSNKTSDDGY

NWRKYGQKQVKGSENPRSYFKCTYPNCLTKKKVETSLVKGQMIEIVYKGSHNHPKPQSTK

RSSSTAIAAHQNSSNGDGKDIGEDETEAKRWKREENVKEPRVVVQTTSDIDILDDGYRWR

KYGQKVVKGNPNPRSYYKCTFTGCFVRKHVERAFQDPKSVITTYEGKHKHQIPTPRRGPV

LRLLGKTET

>AT5G13080.1WRKY75 lld

MEGYDNGSLYAPFLSLKSHSKPELHQGEEESSKVRSEGCSKSVESSKKKGKKQRYAFQTR

SQVDILDDGYRWRKYGQKAVKNNKFPRSYYRCTYGGCNVKKQVQRLTVDQEVVVTTYEGV

HSHPIEKSTENFEHILTQMQIYSSF

>AT5G15130.1WRKY72 llb

MEVLLKLPSSESPLKDKFGSVQIHEANKGDGDHQELESAKAEMSEVKEENEKLKGMLERI

ESDYKSLKLRFFDIIQQEPSNTATKNQNMVDHPKPTTTDLSSFDQERELVSLSLGRRSSS

PSDSVPKKEEKTDAISAEVNADEELTKAGLTLGINNGNGGEPKEGLSMENRANSGSEEAW

APGKVTGKRSSPAPASGGDADGEAGQQNHVKRARVCVRARCDTPTMNDGCQWRKYGQKIA

KGNPCPRAYYRCTVAPGCPVRKQVQRCADDMSILITTYEGTHSHSLPLSATTMASTTSAA

ASMLLSGSSSSPAAEMIGNNLYDNSRFNNNNKSFYSPTLHSPLHPTVTLDLTAPQHSSSS

SSSLLSLNFNKFSNSFQRFPSTSLNFSSTSSTSSNPSTLNLPAIWGNGYSSYTPYPYNNV

QFGTSNLGKTVQNSQSLTETLTKALTSDPSFHSVIAAAISTMVGSNGEQQIVGPRHSISN

NIQQTNTTNNNKGCGGYFSSLLMSNIMASNQTGASLDQPSSQLPPFSMFKNSSSSSSTTN

FVNKEEKS

>AT5G22570.1WRKY38 lll

MEMNSPHEKAVQAIRYGHSCAMRLKRRLNHPMADGGPLSSYDLAKSIVESFSNAISILSA

KPETEDDQFSDLSSRDSSPPPQGSPSKKRKIDSTNSSENWRDDSPDPIYYDGYLWRKYGQ

KSIKKSNHQRSYYRCSYNKDHNCEARKHEQKIKDNPPVYRTTYFGHHTCKTEHNLDAIFI

AGQDPLDDFKSTQMIRFGKDQDQEKESRSNGFSLSVKHEEDIIKEQAIDQYREITSNDQD

CQDVIEEYLSSPSGSYPPSSSSGSESADFNSDLLFDNPDSWDRYDQFYF

>AT5G24110.1 WRKY30 lll

MEKNHSSGEWEKMKNEINELMIEGRDYAHQFGSASSQETREHLAKKILQSYHKSLTIMNY

SGELDQVSQGGGSPKSDDSDQEPLVIKSSKKSMPRWSSKVRIAPGAGVDRTLDDGFSWRK

YGQKDILGAKFPRGYYRCTYRKSQGCEATKQVQRSDENQMLLEISYRGIHSCSQAANVGT

TMPIQNLEPNQTQEHGNLDMVKESVDNYNHQAHLHHNLHYPLSSTPNLENNNAYMLQMRD

QNIEYFGSTSFSSDLGTSINYNFPASGSASHSASNSPSTVPLESPFESYDPNHPYGGFGG

FYS

>AT5G26170.1WRKY50 llc

MNDADTNLGSSFSDDTHSVFEFPELDLSDEWMDDDLVSAVSGMNQSYGYQTSDVAGALFS

GSSSCFSHPESPSTKTYVAATATASADNQNKKEKKKIKGRVAFKTRSEVEVLDDGFKWRK

YGKKMVKNSPHPRNYYKCSVDGCPVKKRVERDRDDPSFVITTYEGSHNHSSMN

>AT5G28650.1WRKY74 lld

MEEVEAANKAAVESCHGVLNLLSQQTNDSKSIMVETREAVCKFKRVSSLLSRGLGQRKIK

KLNNNNYKFSSSLLPQHMFLESPVCSNNAISGCIPILAPKPLQIVPAGPPPLMLFNQNMC

LDKSFLELKPPSSRAVDPKPYQFIHTHQQGVYSRSKSGLNLKFDGSIGASCYSPSISNGS

RSFVSSLSMDGSVTDYDRNSFHLIGLPQGSDHISQHSRRTSCSGSLKCGSKSKCHCSKKR

KLRVKRSIKVPAISNKIADIPPDEYSWRKYGQKPIKGSPHPRGYYKCSSVRGCPARKHVE

RCVEETSMLIVTYEGEHNHSRILSSQSAHT

>AT5G41570.1WRKY24 llc

MDREDINPMLSRLDVENNNTFSSFVDKTLMMMPPSTFSGEVEPSSSSSWYPESFHVHAPP

LPPENDQIGEKGKELKEKRSRKVPRIAFHTRSDDDVLDDGYRWRKYGQKSVKHNAHPRSY

YRCTYHTCNVKKQVQRLAKDPNVVVTTYEGVHNHPCEKLMETLNPLLRQLQFLSSFSNL

>AT5G43290.1|Arabidopsis_thaliana|WRKY|AT5G43290.1

MEEEGYQWARRCGNNAVEDPFVYEPPLFFLPQDQHHMHGLMPNEDFIANKFVTSTLYSGP

RIQDIANALALVEPLTHPVREISKSTVPLLERSTLSKVDRYTLKVKNNSNGMCDDGYKWR

KYGQKSIKNSPNPRSYYKCTNPICNAKKQVERSIDESNTYIITYEGFHFHYTYPFFLPDK

TRQWPNKKTKIHKHNAQDMNKKSQTQEESKEAQLGELTNQNHPVNKAQENTPANLEEGLF

FPVDQCRPQQGLLEDVVAPAMKNIPTRDSVLTAS

>AT5G45050.1 WRKY16Glle

MTESEQIVYISCIEEVRYSFVSHLSKALQRKGVNDVFIDSDDSLSNESQSMVERARVSVM

ILPGNRTVSLDKLVKVLDCQKNKDQVVVPVLYGVRSSETEWLSALDSKGFSSVHHSRKEC

SDSQLVKETVRDVYEKLFYMERIGIYSKLLEIEKMINKQPLDIRCVGIWGMPGIGKTTLA

KAVFDQMSGEFDAHCFIEDYTKAIQEKGVYCLLEEQFLKENAGASGTVTKLSLLRDRLNN

KRVLVVLDDVRSPLVVESFLGGFDWFGPKSLIIITSKDKSVFRLCRVNQIYEVQGLNEKE

ALQLFSLCASIDDMAEQNLHEVSMKVIKYANGHPLALNLYGRELMGKKRPPEMEIAFLKL

KECPPAIFVDAIKSSYDTLNDREKNIFLDIACFFQGENVDYVMQLLEGCGFFPHVGIDVL

VEKSLVTISENRVRMHNLIQDVGRQIINRETRQTKRRSRLWEPCSIKYLLEDKEQNENEE

QKTTFERAQVPEEIEGMFLDTSNLSFDIKHVAFDNMLNLRLFKIYSSNPEVHHVNNFLKG

SLSSLPNVLRLLHWENYPLQFLPQNFDPIHLVEINMPYSQLKKLWGGTKDLEMLKTIRLC

HSQQLVDIDDLLKAQNLEVVDLQGCTRLQSFPATGQLLHLRVVNLSGCTEIKSFPEIPPN

IETLNLQGTGIIELPLSIVKPNYRELLNLLAEIPGLSGVSNLEQSDLKPLTSLMKISTSY

QNPGKLSCLELNDCSRLRSLPNMVNLELLKALDLSGCSELETIQGFPRNLKELYLVGTAV

RQVPQLPQSLEFFNAHGCVSLKSIRLDFKKLPVHYTFSNCFDLSPQVVNDFLVQAMANVI

AKHIPRERHVTGFSQKTVQRSSRDSQQELNKTLAFSFCAPSHANQNSKLDLQPGSSSMTR

LDPSWRNTLVGFAMLVQVAFSEGYCDDTDFGISCVCKWKNKEGHSHRREINLHCWALGKA

VERDHTFVFFDVNMRPDTDEGNDPDIWADLVVFEFFPVNKQRKPLNDSCTVTRCGVRLIT

AVNCNTSIENISPVLSLDPMEVSGNEDEEVLRVRYAGLQEIYKALFLYIAGLFNDEDVGL

VAPLIANIIDMDVSYGLKVLAYRSLIRVSSNGEIVMHYLLRQMGKEILHTESKKTDKLVD

NIQSSMIATKEIEITRSKSRRKNNKEKRVVCVVDRGSRSSDLWVWRKYGQKPIKSSPYPR

SYYRCASSKGCFARKQVERSRTDPNVSVITYISEHNHPFPTLRNTLAGSTRSSSSKCSDV

TTSASSTVSQDKEGPDKSHLPSSPASPPYAAMVVKEEDMEQWDNMEFDVDVEEDTFIPEL

FPEDTFADMDKLEENSQTMFLSRRSSGGNMEAQGKNSSDDREVNLPSKILNR

>AT5G46350.1WRKY8 G llc

MSHEIKDLNNYHYTSSYNHYNINNQNMINLPYVSGPSAYNANMISSSQVGFDLPSKNLSP

QGAFELGFELSPSSSDFFNPSLDQENGLYNAYNYNSSQKSHEVVGDGCATIKSEVRVSAS

PSSSEADHHPGEDSGKIRKKREVRDGGEDDQRSQKVVKTKKKEEKKKEPRVSFMTKTEVD

HLEDGYRWRKYGQKAVKNSPYPRSYYRCTTQKCNVKKRVERSYQDPTVVITTYESQHNHP

IPTNRRTAMFSGTTASDYNPSSSPIFSDLIINTPRSFSNDDLFRVPYASVNVNPSYHQQQ

HGFHQQESEFELLKEMFPSVFFKQEP

>AT5G49520.1WRKY48llc

MEKKKEEDHHHQQQQQQQKEIKNTETKIEQEQEQEQKQEISQASSSSNMANLVTSSDHHP

LELAGNLSSIFDTSSLPFPYSYFEDHSSNNPNSFLDLLRQDHQFASSSNSSSFSFDAFPL

PNNNNNTSFFTDLPLPQAESSEVVNTTPTSPNSTSVSSSSNEAANDNNSGKEVTVKDQEE

GDQQQEQKGTKPQLKAKKKNQKKAREARFAFLTKSDIDNLDDGYRWRKYGQKAVKNSPYP

RSYYRCTTVGCGVKKRVERSSDDPSIVMTTYEGQHTHPFPMTPRGHIGMLTSPILDHGAT

TASSSSFSIPQPRYLLTQHHQPYNMYNNNSLSMINRRSSDGTFVNPGPSSSFPGFGYDMS

QASTSTSSSIRDHGLLQDILPSQIRSDTINTQTNEENKK

>AT5G52830.1 WRKY27 lle

MSSEDWDLFAVVRSCSSSVSTTNSCAGHEDDIGNCKQQQDPPPPPLFQASSSCNELQDSC

KPFLPVTTTTTTTWSPPPLLPPPKASSPSPNILLKQEQVLLESQDQKPPLSVRVFPPSTS

SSVFVFRGQRDQLLQQQSQPPLRSRKRKNQQKRTICHVTQENLSSDLWAWRKYGQKPIKG

SPYPRNYYRCSSSKGCLARKQVERSNLDPNIFIVTYTGEHTHPRPTHRNSLAGSTRNKSQ

PVNPVPKPDTSPLSDTVKEEIHLSPTTPLKGNDDVQETNGDEDMVGQEVNMEEEEEEEEV

EEDDEEEEDDDDVDDLLIPNLAVRDRDDLFFAGSFPSWSAGSAGDGGG

>AT5G56270.1WRKY2 l

MAGFDENVAVMGEWVPRSPSPGTLFSSAIGEEKSSKRVLERELSLNHGQVIGLEEDTSSN

HNKDSSQSNVFRGGLSERIAARAGFNAPRLNTENIRTNTDFSIDSNLRSPCLTISSPGLS

PATLLESPVFLSNPLAQPSPTTGKFPFLPGVNGNALSSEKAKDEFFDDIGASFSFHPVSR

SSSSFFQGTTEMMSVDYGNYNNRSSSHQSAEEVKPGSENIESSNLYGIETDNQNGQNKTS

DVTTNTSLETVDHQEEEEEQRRGDSMAGGAPAEDGYNWRKYGQKLVKGSEYPRSYYKCTN

PNCQVKKKVERSREGHITEIIYKGAHNHLKPPPNRRSGMQVDGTEQVEQQQQQRDSAATW

VSCNNTQQQGGSNENNVEEGSTRFEYGNQSGSIQAQTGGQYESGDPVVVVDASSTFSNDE

DEDDRGTHGSVSLGYDGGGGGGGGEGDESESKRRKLEAFAAEMSGSTRAIREPRVVVQTT

SDVDILDDGYRWRKYGQKVVKGNPNPRSYYKCTAPGCTVRKHVERASHDLKSVITTYEGK

HNHDVPAARNSSHGGGGDSGNGNSGGSAAVSHHYHNGHHSEPPRGRFDRQVTTNNQSPFS

RPFSFQPHLGPPSGFSFGLGQTGLVNLSMPGLAYGQGKMPGLPHPYMTQPVGMSEAMMQR

GMEPKVEPVSDSGQSVYNQIMSRLPQI

>AT5G64810.1WRKY51 llc

MNISQNPSPNFTYFSDENFINPFMDNNDFSNLMFFDIDEGGNNGLIEEEISSPTSIVSSE

TFTGESGGSGSATTLSKKESTNRGSKESDQTKETGHRVAFRTRSKIDVMDDGFKWRKYGK

KSVKNNINKRNYYKCSSEGCSVKKRVERDGDDAAYVITTYEGVHNHESLSNVYYNEMVLS

YDHDNWNQHSLLRS
