# Supplementary material for: Genome-wide Identification of WRKY transcription factor family members in sorghum (Sorghum bicolor (L.) moench)
Source: PLoS One. 2020 Aug 17;15(8):e0236651. doi: 10.1371/journal.pone.0236651 (PMC7430707; doi:10.1371/journal.pone.0236651)
Supplement: S7 File — (DOCX) [file pone.0236651.s007.docx]

| **Application** | **Name** | **Sequence 5'→ 3'** |
| --- | --- | --- |
| qRT-PCR | SbWRKY45-Forward | CTCTGGAGACGGAGCTACAC |
|  | SbWRKY45-Reverse | CCACCATCTCCGTGTACTGG |
|  | SbWRKY72-Forward | CCAAAGGCCTACTTCCGGTG |
|  | SbWRKY72-Reverse | GTGCATGCCGATGTAGGTGA |
|  | SbWRKY74-Forward | AGCATAGCTTCAGAGACGGC |
|  | SbWRKY74-Reverse | GGTTCACGAGACCCATAGGC |
|  | SbWRKY75-Forward | TGAAGCTTGCGTTCAATGCC |
|  | SbWRKY75-Reverse | TTCCTCAGCACCGCAGATTT |
|  | SbWRKY79-Forward | TCATCAGCAAGACGACACCC |
|  | SbWRKY79-Reverse | GAAGGAAGGGAAGGACGTGG |
| Internal control | GAPDH-Forward | AAGGCCGGCATTGCTTTGAAT |
|  | GAPDH-Reverse | ACATGTGGCAGATCAGGTCGA |

***SbWRKY* specific primers used in qRT-PCR analysis**
