# Supplementary material for: Genome-wide Identification of WRKY transcription factor family members in sorghum (Sorghum bicolor (L.) moench)
Source: PLoS One. 2020 Aug 17;15(8):e0236651. doi: 10.1371/journal.pone.0236651 (PMC7430707; doi:10.1371/journal.pone.0236651)
Supplement: Attachment — Submitted filename: PONE-D-20-02381_reviewer.pdf [file pone.0236651.s008.pdf]

PLOS ONE

Genome-wide identification of WRKY transcription factor family members in sorghum (Sorghum bicolor (L.) moench)

--Manuscript Draft--

**Manuscript Number:**	PONE-D-20-02381
**Article Type:**	Research Article
**Full Title:**	Genome-wide identification of WRKY transcription factor family members in sorghum (Sorghum bicolor (L.) moench)
**Short Title:**	WRKY transcription factor family in sorghum Genome
**Corresponding Author:**	Elamin Hafiz Baillo<br>Institute of Genetics and Developmental Biology Center for Agricultural Resources Research<br>shijiazhuang, Hebe CHINA
**Keywords:**	
**Abstract:**	WRKY transcription factors regulate diverse biological processes in plants, including abiotic and biotic stress responses, and they compose one of the largest transcription factor families in higher plants. Although the last decade has seen significant progress towards the identification and functional characterization of WRKY genes in diverse species, little is known about the WRKY family in sorghum (Sorghum bicolor (L.) moench). Here we report a comprehensive identification of 94 putative WRKY transcription factors (SbWRKYs). The SbWRKYs were divided into three groups (I, II, and III), with those in group II further classified into five subgroups (IIa-IIe), based on their conserved domains and zinc finger motif type. WRKYs from the model plant Arabidopsis thaliana were used for the phylogenetic analysis of the SbWRKY genes. Motif analysis showed that all SbWRKYs contained either one or two WRKY domains and that SbWRKYs within the same group had similar motif compositions. SbWRKY genes were located on all 10 sorghum chromosomes, and some gene clusters and two tandem duplications were detected. SbWRKY gene structure analysis showed that they had 0-7 introns and that the majority of SbWRKYs consisted of two introns and three exons. Publicly available microarray data were used to analyze the expression profiles of 78 SbWRKY genes at different growth stages and in different tissues. Additionally, the induction of SbWRKYs by different abiotic stresses hinted at their potential involvement in stress response. qRT-PCR analysis revealed different expression pattern of SbWRKYs under drought stress treatment. Functionally characterized WRKY genes in Arabidopsis and other species will provide clues for the functional characterization of the orthologs in sorghum. Thus, the present findings provide a solid foundation for future functional studies of SbWRKY genes and their roles in the response to critical stresses such as drought.
**Order of Authors:**	Elamin Hafiz Baillo
	Yinghui Guo
	Zhengbin Zhang
	Muhammad Sajid Hanif
	Ping Xu
**Additional Information:**	
**Question**	**Response**
**Financial Disclosure**<br><br>Enter a financial disclosure statement that describes the sources of funding for the work included in this submission. Review the submission guidelines for detailed	The authors received no specific funding for this work

Powered by Editorial Manager® and ProduXion Manager® from Aries Systems Corporation

requirements. View published research articles from *PLOS ONE* for specific examples.<br><br>This statement is required for submission and **will appear in the published article** if the submission is accepted. Please make sure it is accurate.<br><br>**Unfunded studies**<br>Enter: *The author(s) received no specific funding for this work.*<br><br>**Funded studies**<br>Enter a statement with the following details:<br>• Initials of the authors who received each award<br>• Grant numbers awarded to each author<br>• The full name of each funder<br>• URL of each funder website<br>• Did the sponsors or funders play any role in the study design, data collection and analysis, decision to publish, or preparation of the manuscript?<br>• **NO** - Include this sentence at the end of your statement: *The funders had no role in study design, data collection and analysis, decision to publish, or preparation of the manuscript.*<br>• **YES** - Specify the role(s) played.<br><br>* typeset	
**Competing Interests**<br><br>Use the instructions below to enter a competing interest statement for this submission. On behalf of all authors, disclose any competing interests that could be perceived to bias this work—acknowledging all financial support and any other relevant financial or non-financial competing interests.<br><br>This statement **will appear in the published article** if the submission is accepted. Please make sure it is accurate. View published research articles from *PLOS ONE* for specific examples.	The authors have declared that no competing interests exist.

Powered by Editorial Manager® and ProduXion Manager® from Aries Systems Corporation

**NO authors have competing interests**<br><br>Enter: *The authors have declared that no competing interests exist.*<br><br>**Authors with competing interests**<br><br>Enter competing interest details beginning with this statement:<br><br>*I have read the journal's policy and the authors of this manuscript have the following competing interests: [insert competing interests here]*<br><br>* typeset	
**Ethics Statement**<br><br>Enter an ethics statement for this submission. This statement is required if the study involved:<br><br>• Human participants<br>• Human specimens or tissue<br>• Vertebrate animals or cephalopods<br>• Vertebrate embryos or tissues<br>• Field research<br><br>Write "N/A" if the submission does not require an ethics statement.<br><br>General guidance is provided below. Consult the submission guidelines for detailed instructions. **Make sure that all information entered here is included in the Methods section of the manuscript.**	N/A

Powered by Editorial Manager® and ProduXion Manager® from Aries Systems Corporation

**Format for specific study types**<br><br>**Human Subject Research (involving human participants and/or tissue)**<br>• Give the name of the institutional review board or ethics committee that approved the study<br>• Include the approval number and/or a statement indicating approval of this research<br>• Indicate the form of consent obtained (written/oral) or the reason that consent was not obtained (e.g. the data were analyzed anonymously)<br><br>**Animal Research (involving vertebrate animals, embryos or tissues)**<br>• Provide the name of the Institutional Animal Care and Use Committee (IACUC) or other relevant ethics board that reviewed the study protocol, and indicate whether they approved this research or granted a formal waiver of ethical approval<br>• Include an approval number if one was obtained<br>• If the study involved *non-human primates*, add *additional details* about animal welfare and steps taken to ameliorate suffering<br>• If anesthesia, euthanasia, or any kind of animal sacrifice is part of the study, include briefly which substances and/or methods were applied<br><br>**Field Research**<br><br>Include the following details if this study involves the collection of plant, animal, or other materials from a natural setting:<br>• Field permit number<br>• Name of the institution or relevant body that granted permission	
**Data Availability**<br><br>Authors are required to make all data underlying the findings described fully available, without restriction, and from the time of publication. PLOS allows rare exceptions to address legal and ethical concerns. See the PLOS Data Policy and FAQ for detailed information.	Yes - all data are fully available without restriction

Powered by Editorial Manager® and ProduXion Manager® from Aries Systems Corporation

A Data Availability Statement describing where the data can be found is required at submission. Your answers to this question constitute the Data Availability Statement and **will be published in the article**, if accepted.<br><br>**Important:** Stating 'data available on request from the author' is not sufficient. If your data are only available upon request, select 'No' for the first question and explain your exceptional situation in the text box.<br><br>Do the authors confirm that all data underlying the findings described in their manuscript are fully available without restriction?	
**Describe where the data may be found in full sentences. If you are copying our sample text, replace any instances of XXX with the appropriate details.**<br><br>• If the data are **held or will be held in a public repository**, include URLs, accession numbers or DOIs. If this information will only be available after acceptance, indicate this by ticking the box below. For example: *All XXX files are available from the XXX database (accession number(s) XXX, XXX.).*<br>• If the data are all contained **within the manuscript and/or Supporting Information files**, enter the following: *All relevant data are within the manuscript and its Supporting Information files.*<br>• If neither of these applies but you are able to provide **details of access elsewhere**, with or without limitations, please do so. For example:<br><br>*Data cannot be shared publicly because of [XXX]. Data are available from the XXX Institutional Data Access / Ethics Committee (contact via XXX) for researchers who meet the criteria for access to confidential data.*<br><br>*The data underlying the results presented in the study are available from (include the name of the third party*	All relevant data are within the manuscript and its Supporting Information files.

and contact information or URL).<br>• This text is appropriate if the data are owned by a third party and authors do not have permission to share the data.<br><br>* typeset	
Additional data availability information:	

Manuscript
Click here to access/download;Manuscript;MANUSCRIPT-PLOS ONE-BAILLO 2020-WORD.pdf

RESEARCH ARTICLE

Genome-wide identification of WRKY transcription factor family members in sorghum (*Sorghum bicolor* (L.) moench)

Elamin Hafiz Baillo[1,2,3,4], Yinghui Guo[2], Zhengbin Zhang[1,2,3,*], Muhammad Sajid Hanif[2], and Ping Xu[1,2,3]

[1] Key Laboratory of Agricultural Water Resources, Hebei Laboratory of Agricultural Water Saving, Center for Agricultural Resources Research, Institute of Genetics and Developmental Biology, University of Chinese Academy of Sciences, Shijiazhuang, Hebei, China.
[2] University of Chinese Academy of Sciences, Beijing, China.
[3] Innovation Academy for Seed Design, Chinese Academy of Sciences, Beijing, China.
[4] Agricultural Research Corporation (ARC), Ministry of Agriculture, Gezira, Sudan.

Abstract

WRKY transcription factors regulate diverse biological processes in plants, including abiotic and biotic stress responses, and they compose one of the largest transcription factor families in higher plants. Although the last decade has seen significant progress towards the identification and functional characterization of *WRKY* genes in diverse species, little is known about the WRKY family in sorghum (*Sorghum bicolor* (L.) moench). Here we report a comprehensive identification of 94 putative WRKY transcription factors (SbWRKYs). The SbWRKYs were divided into three groups (I, II, and III), with those in group II further classified into five subgroups (IIa-IIe), based on their conserved domains and zinc finger motif type. WRKYs from the model plant *Arabidopsis thaliana* were used for the phylogenetic analysis of the *SbWRKY* genes. Motif analysis showed that all SbWRKYs contained either one or two WRKY domains and that SbWRKYs within the same group had similar motif compositions. *SbWRKY* genes were located on all 10 sorghum chromosomes, and some gene clusters and two tandem duplications were detected. *SbWRKY* gene structure analysis showed that they had 0-7 introns and that the majority of *SbWRKYs* consisted of two introns and three exons. Publicly available microarray data were used to analyze the expression profiles of 78 *SbWRKY* genes at different growth stages and in different tissues. Additionally, the induction of *SbWRKYs* by different abiotic stresses hinted at their potential involvement in stress response. qRT-PCR analysis revealed different expression pattern of *SbWRKYs* under drought stress treatment. Functionally characterized *WRKY* genes in *Arabidopsis* and other species will provide clues for the functional characterization of the orthologs in sorghum. Thus, the present findings provide a solid foundation for future functional studies of *SbWRKY* genes and their roles in the response to critical stresses such as drought.

Introduction

Transcription factors (TFs) bind to *cis*-elements in the promoter regions of their target genes to regulate their expression at the transcriptional level. As a result, individual TFs may control several biological processes. WRKY TFs compose one of the largest TF families in plants, and they are known to regulate various stress responses in addition to other biological processes. The WRKY family is a plant-specific gene family whose name derives from the conserved WRKYGQK heptapeptide motif at the N-terminal of the highly conserved WRKY domain, which is 60 amino acids long and also has a zinc finger motif at the C-terminal end [1]. These two motifs are essential for the binding of WRKY TFs to the W-box *cis*-element in the promoters of their target genes. WRKY proteins can be classified into three groups (I, II, and III) according to the number of WRKY domains and the zinc finger motif type, which is either C_2H_2 or C2HC [2]. For example, the classification of the 72 *WRKY* genes in *Arabidopsis* is as follows: 32, 26, and 14 genes in groups I, II, and III, respectively. Group I WRKY proteins have two WRKY domains and the C_2H_2-type zinc finger. Group II members have one WRKY domain and the same C_2H_2 pattern, and these proteins can be further classified into five subgroups (IIa, IIb, IIc, IId, and IIe) [3]. Finally, group III WRKY proteins have one WRKY domain and the C2HC-type zinc finger. Notably, the two WRKY domains of group I members probably function in different ways: the C-terminal WRKY domain contributes to binding specificity, while the precise role of the N-terminal WRKY domain is unresolved [4].

In the time since the identification of the first *WRKY* gene, *SPF1* from *Ipomoea batatas* sweet potato (Ishiguro & Nakamura, 1994), numerous *WRKY* genes have been characterized in many different species, including wheat [5], millet [6], sesame [7], chickpea [8], and *Populus trichocarpa* [9].

WRKY TFs regulate diverse biological processes, including the transcriptional reprogramming associated with plant stress responses. Considering that the sessile nature of plants leaves them exposed to various abiotic and biotic stresses, the involvement of plant-specific WRKY TF proteins in stress responses underscores that plants have evolved mechanisms to help counteract the detrimental effects of environmental stress [4].

Recent studies have provided different examples of the roles of WRKY TFs in response to various stresses. One common strategy is the introduction of WRKY TF genes from another species into *Arabidopsis* to assess how transgenic expression of the gene impacts stress tolerance. For example, transgenic *Arabidopsis* overexpressing maize *ZmWRKY40* exhibited significant drought tolerance, which reflected the regulation of ROS and stress-related genes by the WRKY TF [10]. Several studies have demonstrated that WRKY TFs regulate various biological processes and control gene expression via combinations of positive or negative regulation [4]. For example, in *Arabidopsis*, overexpression of *WRKY13* enhances cadmium tolerance in transgenic plants, attributable in part to WRKY13 binding to the *PDR8* gene; the resulting transcriptional regulation leads to a decrease in cadmium accumulation and thus enhanced tolerance [11]. On the other hand, *WRKY12* act as a negative regulators for cadmium tolerance. Transgenic *Arabidopsis* plants overexpressing *WRKY12* have shown reduced cadmium tolerance. WRKY12 represses *GSH1* expression via binding directly to the W-box in its promoter [12]. Likewise, overexpression of *ZmWRKY17* reduce salt stress tolerance in transgenic *Arabidopsis* and decrease ABA by regulating ABA-dependent and stress-responsive genes [13]. In the last few years numerous studies have identified *WRKY* genes responsible for drought tolerance in different species. For instance, in wheat,

overexpressing of *TaWRKY1* and *TaWRKY33* enhanced drought and heat tolerance in transgenic *Arabidopsis* plants [14]. Beyond the functional characterization of *WRKY* genes in *Arabidopsis*, however, the functions of many *WRKY* genes remain to be validated in non-model species. Overexpression of *TaWRKY2* enhanced drought stress and as well ~~as showed that it~~ increas~~ed~~ yield in the transgenic wheat plants [15]. Furthermore, transgenic wheat overexpressing *Arabidopsis* *WRKY30* exhibited drought and heat tolerance, and expression analysis revealed that stress-related genes and enzyme-encoding genes were induced [16]. In wild type sorghum (*Sorghum propinquum*) the *SpWRKY* ~~conferred to be~~ controll~~ing~~ seed shattering ~~trait~~ by a locus which unrelated to domestication [17]. These studies have ~~ap~~proved that WRKY TFs play important roles and raise the prospect of their potential for crop improvement in terms of stress tolerance.

Genome-wide analyses in different species have identified a large number of *WRKY* genes, including 171 *WRKY* genes in wheat [5], 119 in maize [18], 103 in rice [19], 71 in sesame [20], 70 in chickpea [8], and 79 in potato [21]. Sorghum (*Sorghum bicolor* (L.) moench) is the fifth most important cereal crop, and it has unique adaptations that allow it to withstand harsh conditions at different growth stages. Sorghum is a C4 species with a small genome size (730 Mb), approximately 25% of the genome sizes of maize and sugarcane, and it is diploid (2n = 2x = 20) and self-pollinating [22]. These characteristics make it an ideal model for genomic and functional studies of plant growth under stress conditions. Because sorghum thrives under water scarcity and high temperatures, it is also an excellent model species for TF studies aimed at enhancing tolerance or resistance to abiotic stresses, particularly drought [23,24]. The availability of a complete genome assembly for sorghum provides an opportunity for the genome-wide identification of *SbWRKY* genes, which we report

here. The following analyses were also performed for the *SbWRKY* genes and the corresponding proteins: classification, phylogenetic analysis, characterization of physical and chemical properties, chromosome mapping, exon-intron structure and conserved motif analysis, and expression profiling.

Material and Methods

Identification of WRKY family genes in sorghum

We collected data from the following databases to identify putative *SbWRKY* genes. The Plant Transcription Factor Database version 4 (http://planttfdb.cbi.pku.edu.cn/) was used to download the amino acid sequences of sorghum WRKY proteins, and "WRKY" was used as a query to search against the Grassius Transcription Factor Database (https://grassius.org/grasstfdb.php). The WRKY domain ID (PF03106) was used to obtain the putative WRKY proteins from *S. bicolor* (v3.1) through the Joint Genome Institute (JGI) (https://phytozome.jgi.doe.gov/pz/portal.html#). The keyword “WRKY” was also used as a search query in the MOROCOSHI Sorghum Transcription Factor Database (http://sorghum.riken.jp/morokoshi/Home.html).

CD-HIT suite (http://weizhong-lab.ucsd.edu/cdhit_suite/cgi-bin/index.cgi?cmd=cd-hit) was used to remove the redundant and incomplete sequences, and SMART (http://smart.embl-heidelberg.de/#) [25] was used to confirm that the sequences contained the WRKY domain.

For all identified SbWRKY proteins, the predicted isoelectric point (pI) and molecular weight (MW) were obtained from the ExPASy proteomic server (http://web.expasy.org/protparam).

Chromosome mapping and tandem duplication of *SbWRKY* genes

Information about the chromosomal locations of all identified *SbWRKY* genes was obtained using the Phytozome BioMart tool (https://phytozome.jgi.doe.gov/biomart/martview/), and the genes were mapped onto the sorghum chromosomes by MapChart (v.2.32.). Tandem duplication of *SbWRKYs* was based on the following criteria: genes within a 100 kb region on an individual chromosome with a sequence similarity ≥ 70% [26]. Sequence similarities were quantified using EMBOSS Water, which uses the Smith-Waterman local pairwise alignment algorithm (https://www.ebi.ac.uk/Tools/psa/emboss_water/).

Classification and phylogenetic analysis of *SbWRKY* genes

Arabidopsis WRKY amino acid sequences obtained from the *Arabidopsis* Information Resource (TAIR) (https://www.arabidopsis.org/) were used to construct the phylogenetic tree and classify *SbWRKY* genes. Multiple sequence alignment was performed using ClustalW for AtWRKY and SbWRKY protein sequences, and the phylogenetic tree was constructed using MEGA v7.0 (https://www.megasoftware.net/) and the p-distance model. Pairwise deletion and 1000 bootstrap replicates were used for the neighbor-joining method [27]. Based on the phylogenetic tree of AtWRKY and SbWRKY sequences, the *SbWRKY* genes were classified into different groups and subgroups. The identification of WRKY orthologs in sorghum and *Arabidopsis* was based on the sequence alignment data and the phylogenetic tree [21].

Gene structure analysis of *SbWRKY* genes

The exon-intron structure of *SbWRKYs* was analyzed using the Gene Structure Display Server (GSDS v. 2.0) (http://gsds.cbi.pku.edu.cn/) from the Center for Bioinformatics at Peking University

[28]. The genomic sequence and coding sequence (CDS) of each *SbWRKY* gene were used to predict the exon-intron pattern.

2.5 Conserved motif distribution analysis of *SbWRKY* genes

Conserved motifs in the SbWRKY proteins were identified using Multiple Em for Motif Elicitation (MEME) (v. 4.9.0; http://meme.nbcr.net/meme/) with the following parameters: maximum motif number, 20; site distributions, any number of repetitions; minimum and maximum width, 10 and 50 [29].

Digital expression pattern analysis of *SbWRKY* genes

To survey the *SbWRKY* expression profiles, Affymetrix transcriptomic array data for sorghum were obtained from the Sorghum Functional Genomics Database (http://structuralbiology.cau.edu.cn/sorghum/pattern.php). The Genevestigator platform was used to analyze the expression profiles of *SbWRKYs* under different environmental stresses (drought, cold, salt, and heat) with different samples stored in the Genevestigator platform [30]. The *SbWRKY* expression profile heatmaps were generated using a hierarchical clustering analysis tool on the Genevestigator platform.

Drought stress treatment and samples collection

Seeds of *Sorghum bicolor* genotype (SX44B) used in this study provided by Professor Zhang Fuyao (Sorghum Institute, Shanxi Academy of Agricultural Sciences, Shanxi, China). The seeds were surface-sterilized with 75% ethanol and 5% sodium hypochlorite (v/v) for 1-2 minutes then rinsed three times with distilled water. The sterilized seeds were germinated on two layers of wetted paper

and incubated at 25°C in dark for 3 days. The seedlings were transferred to pots contained soil mixture of vermiculite and peat moss in a ratio (1:1). The seedlings were grown in a growth chamber with 60-70% relative humidity, 28/23°C day/night, 16-h light/8-h dark photoperiod. The seedlings were grown under normal condition for two weeks then exposed to drought treatment, seedlings were treated with PEG(8000) 20%. The seedlings shoot samples were collected at 0, 3, 6, 12, and 24 h, all samples were frozen in liquid nitrogen and stored at -80°C until it used for RNA extraction.

Gene expression analysis by quantitative real-time PCR (qRT-PCR)

RNAs were extracted from the samples using (Promega, China) according to manufacturer's specifications. The first-strand cDNA was synthesized by reverse transcription of RNA was generated using Easy Script First-strand cDNA Synthesis SuperMix Kit (TransGen Biotech, China). Synthesized cDNA was diluted 1:10 with nuclease-free water to use in qRT-PCR. The expression levels of gene were normalized using housekeeping gene sorghum *GAPDH* gene as internal control. The gene specific primers were designed by using the National Center for Biotechnology Information (NCBI) Primer-BLAST and synthesized by Sangon (Beijing, China). The primers used are listed in a supplementary file (6). The qRT-PCR was carried out using *TransStart* Green qPCR SuperMix UDG (TransGen Biotech, China) following the manufacturer's instructions. The experiment was conducted using Bio-Rad CFX96TM real-time PCR detection system (Bio-Rad, USA). The reaction parameters for the thermal cycling were as follows: 94°C for 10 min and 40 cycles of 94°C for 5s and 55°C for 15s, last 72°C for 10s. Three biological replicates were used, the $2^{-\Delta\Delta Ct}$ was used as a method for quantification [31].

Results

3.1 Identification of WRKY family members in sorghum

Taking advantage of the availability of a complete genome assembly for sorghum, we identified SbWRKY family members using the keyword "WRKY" and the WRKY domain consensus sequence (PF03106) as queries in different databases. In this study, the presence of the WRKYGQK or WRKYGQK-like conserved domain was the basic criterion for the inclusion of genes in the SbWRKY family. Initially, 134, 94, 99, and 97 transcripts were identified from TFDB, Grassius, MOROCOSHI, and JGI, respectively. CD-HIT and multiple sequence alignment were used to remove redundant SbWRKY protein sequences, and the presence of the WRKY domain in the remaining sequences was confirmed by SMART. Thus, redundant sequences and those with an incomplete WRKY domain were removed. A total of 94 non-redundant SbWRKY sequences were identified, and the following gene and protein data were obtained (**Table 1**): gene locus ID, chromosomal location, conserved heptapeptide, gene start and end, protein length, gene group, zinc finger type, and the predicted molecular weight (MW) and isoelectric point (pI). The identified *SbWRKYs* were named according to their physical positions from the top to bottom of the sorghum chromosomes in ascending order (*SbWRKY1* to *SbWRKY94*) as described by [1] (**Table 1**). The identified SbWRKY amino acid sequences were used for further study.

The peptide lengths of the 94 SbWRKYs ranged from 110 to 1584 amino acids, with an average length of 390 amino acids. The predicted MW and pI values ranged from 12.23 to 78.24 and from 4.75 to 10.06, respectively.

Table 1. Characteristics of the identified *SbWRKY* genes

Gene Name	Gene Locus ID	Chromosome Location	Gene Start	Gene End	pI	MW	Conserved Heptapeptide	Zinc Finger Type	Domain Number	Group	Protein Length (aa)
SbWRKY1	Sobic.001G006600.1	Chr01	661532	669834	6.10	42.50	WRKYGQK	C2H2	1	IIe	392
SbWRKY2	Sobic.001G055400.1	Chr01	4145000	4147281	9.42	46.08	WRKYGQK	C2H2	1	IIe	434
SbWRKY3	Sobic.001G083000.1	Chr01	6408709	6412370	6.76	45.19	WRKYGQK	C2H2	2	I	424
SbWRKY4	Sobic.001G084000.1	Chr01	6506824	6511079	6.2	34.37	WRKYGQK	C2H2	1	IIc	331
SbWRKY5	Sobic.001G095500.1	Chr01	7340447	7343822	10.05	43.61	WRKYGQK	C2H2	1	IId	406
SbWRKY6	Sobic.001G148000.1	Chr01	11929277	11931246	8.89	15.12	WRKYGQK	C2H2	1	NG	141
SbWRKY7	Sobic.001G162100.1	Chr01	13349344	13352564	9.78	37.88	WRKYGQK	C2H2	1	IId	352
SbWRKY8	Sobic.001G282400.1	Chr01	55413200	55415329	5.97	46.31	WRKYGQK	C2H2	1	IIe	427
SbWRKY9	Sobic.001G332500.1	Chr01	62096233	62103315	7.34	52.62	WRKYGQK	C2HC	2	I	498
SbWRKY10	Sobic.001G381300.1	Chr01	66930715	66931988	5.60	33.95	WRKYGQK	C2HC	1	III	328
SbWRKY11	Sobic.001G389000.1	Chr01	67559616	67561361	8.71	24.20	**WRKYGEK**	C2H2	1	III	225
SbWRKY12	Sobic.002G008600.2	Chr02	791610	795938	9.71	29.42	WRKYGQK	C2H2	1	IIc	272
SbWRKY13	Sobic.002G128400.2	Chr02	17755739	17757847	5.75	29.09	WRKYGQK	C2H2	1	III	275
SbWRKY14	Sobic.002G168300.3	Chr02	52688090	52696535	6.92	17.74	**WRKYGSK**	C2HC	2	III	1584
SbWRKY15	Sobic.002G174200.1	Chr02	55134836	55136822	5.67	32.89	WRKYGQK	C2HC	1	III	310
SbWRKY16	Sobic.002G174300.1	Chr02	55188598	55190855	5.99	34.60	WRKYGQK	C2HC	1	III	328
SbWRKY17	Sobic.002G202700.1	Chr02	59258884	59260622	6.46	34.72	WRKYGQK	C2H2	1	IIa	327
SbWRKY18	Sobic.002G202800.1	Chr02	59275303	59277597	6.72	31.77	WRKYGQK	C2H2	1	IIa	295
SbWRKY19	Sobic.002G242500.1	Chr02	63158788	63162729	6.94	64.54	WRKYGQK	C2H2	1	I	602
SbWRKY20	Sobic.002G355000.1	Chr02	71806701	71813179	6.24	65.56	WRKYGQK	C2H2	2	I	611
SbWRKY21	Sobic.002G418500.1	Chr02	76629585	76632298	9.17	37.05	WRKYGQK	C2HC	1	III	354
SbWRKY22	Sobic.003G000600.1	Chr03	65678	68474	8.62	59.48	WRKYGQK	C2H2	1	IIb	570
SbWRKY23	Sobic.003G037400.1	Chr03	3517021	3519078	6.07	26.65	**WRKYGKK**	C2H2	1	IIc	260
SbWRKY24	Sobic.003G037500.1	Chr03	3520321	3522720	7.27	56.13	WRKYGQK	C2H2	1	IIb	548
SbWRKY25	Sobic.003G040800.1	Chr03	3787973	3794918	9.75	30.73	WRKYGQK	C2H2	1	IIc	295
SbWRKY26	Sobic.003G138400.1	Chr03	13400561	13405829	6.77	60.58	WRKYGQK	C2H2	1	IIb	582
SbWRKY27	Sobic.003G199400.1	Chr03	52648841	52650568	5.57	23.75	**WRKYGKK**	C2H2	1	IIc	216

SbWRKY28	Sobic.003G200700.1	Chr03	52845063	52846563	9.17	33.94	WRKYGQK	C2H2	1	IIb	332
SbWRKY29	Sobic.003G226600.1	Chr03	56293002	56295461	8.7	44.22	WRKYGQK	C2H2	1	IIe	413
SbWRKY30	Sobic.003G227300.1	Chr03	56472263	56479136	6.62	38.87	WRKYGQK	C2H2	1	IIc	361
SbWRKY31	Sobic.003G242800.2	Chr03	58197072	58201064	6.45	35.97	WRKYGQK	C2HC	1	III	346
SbWRKY32	Sobic.003G248400.1	Chr03	58718991	58722963	7.32	42.36	WRKYGQK	C2H2	1	IIc	410
SbWRKY33	Sobic.003G276000.1	Chr03	61248940	61250384	8.64	23.95	**WRKYGKK**	C2H2	1	IIc	225
SbWRKY34	Sobic.003G285500.1	Chr03	61880213	61882652	5.27	32.23	WRKYGQK	C2H2	1	IIe	319
SbWRKY35	Sobic.003G287200.1	Chr03	62028638	62032127	7.08	26.67	WRKYGQK	C2H2	1	IIc	246
SbWRKY36	Sobic.003G296300.1	Chr03	62835017	62837081	4.75	33.69	WRKYGQK	C2H2	1	IIe	310
SbWRKY37	Sobic.003G337500.1	Chr03	66057329	66064893	5.85	26.64	WRKYGQK	C2HC	1	III	236
SbWRKY38	Sobic.003G337600.1	Chr03	66070688	66076206	5.98	29.56	WRKYGQK	C2HC	1	III	264
SbWRKY39	Sobic.003G337700.1	Chr03	66081053	66083714	5.87	40.92	WRKYGQK	C2HC	1	III	377
SbWRKY40	Sobic.003G337800.1	Chr03	66089525	66092348	5.17	36.43	WRKYGQK	C2HC	1	III	333
SbWRKY41	Sobic.003G337900.1	Chr03	66104667	66107290	5.91	29.63	WRKYGQK	C2HC	1	III	277
SbWRKY42	Sobic.003G341100.1	Chr03	66392943	66395715	6.32	59.35	WRKYGQK	C2H2	2	I	556
SbWRKY43	Sobic.003G353000.1	Chr03	67192266	67198503	6.13	39.68	WRKYGQK	C2H2	1	NG	377
SbWRKY44	Sobic.003G444000.1	Chr03	74207255	74210127	4.76	38.85	WRKYGQK	C2H2	1	IIc	354
SbWRKY45	Sobic.004G065900.1	Chr04	5352084	5353418	8.45	38.95	WRKYGQK	C2H2	1	IIa	364
SbWRKY46	Sobic.004G117600.1	Chr04	12353902	12355528	5.81	39.57	WRKYGQK	C2H2	1	IIe	375
SbWRKY47	Sobic.004G138400.2	Chr04	38911863	38913035	9.78	32.03	WRKYGQK	C2H2	1	IId	299
SbWRKY48	Sobic.004G271800.1	Chr04	61583724	61588753	5.67	51.61	WRKYGQK	C2H2	1	IIe	497
SbWRKY49	Sobic.004G298400.1	Chr04	63778460	63782424	8.43	26.10	WRKYGQK	C2H2	1	IIc	238
SbWRKY50	Sobic.004G312200.1	Chr04	64900147	64902898	5.09	61.31	WRKYGQK	C2H2	1	IIb	578
SbWRKY51	Sobic.005G013400.1	Chr05	1211003	1212487	5.69	36.49	**WRKYGEK**	C2HC	1	III	334
SbWRKY52	Sobic.005G013500.1	Chr05	1224392	1225924	6.31	38.95	**WRKYGEK**	C2HC	1	III	361
SbWRKY53	Sobic.005G013600.2	Chr05	1235265	1241922	8.85	25.24	**WRKYGEK**	C2HC	1	III	227
SbWRKY54	Sobic.005G013800.1	Chr05	1245144	1246547	5.88	30.67	WRKYGQK	C2HC	1	III	271
SbWRKY55	Sobic.005G014000.1	Chr05	1261347	1263314	6.27	33.04	WRKYGQK	C2HC	1	III	289
SbWRKY56	Sobic.005G014200.1	Chr05	1302596	1304021	6	30.80	WRKYGQK	C2HC	1	III	272
SbWRKY57	Sobic.005G117400.2	Chr05	51657060	51660442	9.18	24.24	WRKYGQK	C2H2	1	IIc	225
SbWRKY58	Sobic.006G051700.1	Chr06	38157163	38162677	5.97	12.23	WRKYGQK	C2HC	1	III	110
SbWRKY59	Sobic.006G115700.1	Chr06	48356715	48362695	6.77	78.24	WRKYGQK	C2H2	2	I	740
SbWRKY60	Sobic.006G166300.1	Chr06	52377828	52383758	8.66	29.10	WRKYGQK	C2H2	1	IIc	269

SbWRKY61	Sobic.006G201000.1	Chr06	55241047	55245365	7.01	55.79	WRKYGQK	C2H2	1	IIe	532
SbWRKY62	Sobic.006G206000.1	Chr06	55572908	55574447	10.05	33.13	WRKYGQK	C2H2	1	IId	315
SbWRKY63	Sobic.007G077466.1	Chr07	8936871	8940145	7.7	49.41	WRKYGQK	C2H2	1	IIc	455
SbWRKY64	Sobic.007G085300.1	Chr07	10754655	10756113	9.87	32.97	WRKYGQK	C2H2	1	IId	318
SbWRKY65	Sobic.007G111600.1	Chr07	41587770	41594141	6.05	61.78	WRKYGQK	C2H2	2	I	569
SbWRKY66	Sobic.007G118301.1	Chr07	51192936	51194468	6.48	35.62	WRKYGQK	C2HC	1	III	340
SbWRKY67	Sobic.007G217700.3	Chr07	64590499	64600679	6.89	74.52	WRKYGQK	C2H2	2	I	685
SbWRKY68	Sobic.008G028600.1	Chr08	2541872	2544263	5.35	36.20	WRKYGQK	C2HC	1	III	341
SbWRKY69	Sobic.008G029000.2	Chr08	2604929	2606770	6.72	33.68	WRKYGQK	C2HC	1	III	294
SbWRKY70	Sobic.008G029200.1	Chr08	2612734	2614762	5.99	30.33	WRKYGQK	C2HC	1	III	267
SbWRKY71	Sobic.008G029400.1	Chr08	2625199	2632242	8.94	25.19	**WRKYGEK**	C2HC	1	III	225
SbWRKY72	Sobic.008G060300.1	Chr08	6462595	6464519	5.15	35.55	WRKYGQK	C2HC	1	III	334
SbWRKY73	Sobic.008G107500.1	Chr08	50603514	50609969	7.71	52.42	WRKYGQK	C2H2	2	I	496
SbWRKY74	Sobic.008G153600.1	Chr08	58584243	58587317	10.06	39.29	WRKYGQK	C2H2	1	IId	371
SbWRKY75	Sobic.008G174100.1	Chr08	60851165	60856614	8.89	13.13	**WRKSGQR**	C2HC	1	III	1163
SbWRKY76	Sobic.009G034800.1	Chr09	3182138	3188861	5.79	58.46	WRKYGQK	C2H2	1	IIb	567
SbWRKY77	Sobic.009G068900.1	Chr09	7567284	7568764	6.96	22.09	**WRKYGKK**	C2H2	1	IIc	206
SbWRKY78	Sobic.009G092100.1	Chr09	20912936	20928927	6.39	40.83	WRKYGQK	C2HC	1	III	378
SbWRKY79	Sobic.009G100500.1	Chr09	39826393	39831148	7.69	54.06	WRKYGQK	C2H2	2	I	517
SbWRKY80	Sobic.009G171600.1	Chr09	52696414	52699630	8.28	68.54	WRKYGQK	C2H2	2	I	649
SbWRKY81	Sobic.009G174300.1	Chr09	52963804	52966801	6.25	26.62	**WRKYGEK**	C2HC	1	III	235
SbWRKY82	Sobic.009G206800.1	Chr09	55463596	55464452	9.2	25.32	WRKYGQK	C2H2	1	IIc	241
SbWRKY83	Sobic.009G212800.1	Chr09	55836119	55837874	6.08	22.71	**WRKYGKK**	C2H2	1	IIc	219
SbWRKY84	Sobic.009G234100.1	Chr09	57342319	57345920	6.38	44.44	WRKYGQK	C2H2	1	IIc	424
SbWRKY85	Sobic.009G234900.1	Chr09	57418453	57422639	6.52	65.84	WRKYGQK	C2H2	1	IIb	631
SbWRKY86	Sobic.009G238200.1	Chr09	57628850	57630763	5.81	29.08	WRKYGQK	C2HC	1	III	272
SbWRKY87	Sobic.009G247300.2	Chr09	58261832	58265060	8.22	28.36	WRKYGQK	C2H2	1	IIc	262
SbWRKY88	Sb09g029810	Chr09	58261882	58265547	6.07	36.12	WRKYGQK	C2H2	1	IIc	343
SbWRKY89	Sobic.009G247700.1	Chr09	58309106	58310986	6.05	38.89	WRKYGQK	C2H2	1	IIe	364
SbWRKY90	Sobic.010G035300.2	Chr10	2858141	2865169	6.79	65.39	WRKYGQK	C2H2	1	IIb	625
SbWRKY91	Sobic.010G045700.1	Chr10	3566332	3570889	6.11	40.52	WRKYGQK	C2HC	1	III	378
SbWRKY92	Sobic.010G148600.2	Chr10	42567454	42569300	6.01	41.69	WRKYGQK	C2H2	1	IIe	385
SbWRKY93	Sobic.010G148800.1	Chr10	42829856	42831874	7.58	37.43	WRKYGQK	C2H2	1	IIe	350

SbWRKY94	Sobic.010G209100.1	Chr10	55261687	55263735	9.64	37.82	WRKYGQK	C2H2	1	IIa	348

pI, isoelectric point; MW, molecular weight; aa, amino acid.

Chromosome mapping of the *SbWRKY* genes and tandem duplication analysis

MapChart was used to determine the locations of the 94 *SbWRKY* genes, and they were distributed on all 10 sorghum chromosomes (**Fig 1**). Chr3 had the highest number of *SbWRKYs* with 23 genes, corresponding to 24.47% of the total, followed by 14 on Chr9, 11 on Chr1, and 10 on Chr2. Chr6, Chr7, and Chr10 had the fewest, with only five *SbWRKYs* each. Chr4, Chr5, and Chr8 had six, seven, and eight *SbWRKYs*, respectively.

Tandem repeats were identified based on previously reported criteria [32], namely, two or more genes within a 100 kb region and a sequence similarity ≥ 70%. Gene cluster events were observed on six chromosomes. Specifically, there were five clusters on Chr3; two each on Chr2 and Chr9; and one cluster each on Chr1, Chr5, and Chr8. No clusters were found on Chr4, Chr6, Chr7, and Chr10. High-density clusters were detected on Chr5 and Chr3. Tandem repeats were identified on two chromosomes: two group III genes (*SbWRKY51* and *SbWRKY52*) on Chr5 and two group IIc genes (*SbWRKY87* and *SbWRKY88*) on Chr9 (**Fig 1**). The tandem duplicated genes clustered together in the phylogenetic tree within the respective clades, and the sequence similarity metrics for the gene pairs are provided in **S1**.

Classification and phylogenetic analysis of *SbWRKYs*

To investigate the evolution of SbWRKY family members, an unrooted phylogenetic tree was constructed based on multiple sequence alignment of the full-length amino acid sequences of 65

Arabidopsis AtWRKYs and all 94 SbWRKYs. The phylogenetic tree was constructed using the neighbor-joining (NJ) method in MEGA7.0 (**Fig 2**). The constructed phylogenetic tree was used to classify the SbWRKYs into three major groups (I, II, and III), in accordance with the classification in *Arabidopsis* [1] (**Fig 2**). Of the 11 group I SbWRKYs, 10 had two WRKYGQK motifs and two C_2H_2-type zinc finger motifs ($C\text{-}X_{3\text{-}4}\text{-}C\text{-}X_{22\text{-}23}\text{-}H\text{-}X_1\text{-}H$), corresponding to two WRKY domains. Although the protein encoded by *SbWRKY19* had only one WRKY domain, it also belonged to group I. Fifty protein sequences with one WRKY domain and the C_2H_2-type zinc finger motif ($C\text{-}X_{4\text{-}5}\text{-}C\text{-}X_{23}\text{-}H\text{-}X_1\text{-}H$) were classified into group II. This group was further divided into five subgroups, IIa, IIb, IIc, IId, and IIe, with 4, 8, 20, 6, and 12 members, respectively. Group III contained 31 members with one WRKY domain and the C_2HC-type zinc finger motif ($C\text{-}X_7\text{-}C\text{-}X_{23}\text{-}H\text{-}X_1\text{-}C$). SbWRKY14 was unique because it comprised two WRKY domains with the C_2HC-type zinc finger motif ($C\text{-}X_7\text{-}C\text{-}X_{23}\text{-}H\text{-}X_1\text{-}C$). Thus, it had features associated with group I and group III, but it was classified into group III in the phylogenetic tree. *SbWRKY6* and *SbWRKY43* did not belong to any group (Fig 2 and Table 2). Group II was the largest group and accounted for 53.2% of all putative *SbWRKYs*, which is similar to the results reported in wheat, soybean, and pepper. Overall, the classification of *SbWRKYs* confirms their diversification, which indicates that the different family members have varied functions.

The highly conserved heptapeptide motif WRKYGQK was present in 81 SbWRKY proteins. The heptapeptide variants in the remaining 13 proteins were as follows: WRKYGEK in six proteins (SbWRKY11, SbWRKY51, SbWRKY52, SbWRKY53, SbWRKY71, and SbWRKY81); WRKYGKK in five proteins (SbWRKY23, SbWRKY27, SbWRKY33, SbWRKY77, and SbWRKY83); WRKYGSK in

SbWRKY14; and WRKSGQR in SbWRKY75. Among the 94 identified SbWRKYs, only 11 had two WRKY domains, while the remaining members had one WRKY domain.

The SbWRKY protein sequences, genomic sequences, and CDS are provided in S2, S3, and S4, respectively. Also AtWRKY protein sequences were provvided in S 5

***SbWRKY* gene structure analysis**

To obtain additional clues about the evolution of SbWRKY family members and their specific features, the *SbWRKY* exon-intron structures were analyzed. The intron number in these genes ranged from 0-7, and the introns varied in size. Among the 94 *SbWRKY* genes, 10 had two exons and one intron, 58 had three exons and two introns, eight had four exons and three introns, eight had five exons and four introns, five had six exons and five introns, and one (*SbWRKY14*) had eight exons and seven introns (**Fig 3**). Four genes, *SbWRKY51*, *SbWRKY64*, *SbWRKY66*, and *SbWRKY88*, lacked introns. The results showed that the exon-intron distribution patterns of genes within the same group had some similarities in terms of the numbers and positions of the exons and introns. However, there were also differences within groups. For instance, all genes in group II, the introns number ranged from zero to five, all members of *SbWRKY* genes in subgroup IIb had five introns except *SbWRKY22* and *SbWRKY50* had four, *SbWRKY24* had two, and *SbWRKY28* had one, while introns numbers in group III ranged from zero to seven, *SbWRKY14* was the only gene with seven introns among all *SbWRKYs*. These results indicate that there is considerable structural variation among *SbWRKYs*, which may correspond to functional diversification between closely related members (**Fig 3**).

Motif composition analysis of SbWRKYs

MEME (version 4.11) was used to analyze all the SbWRKY peptide sequences to identify conserved motifs. The 20 distinct conserved motifs that were identified ranged from 6 to 50 residues in length (**Fig 4**). Motifs 1, 2, and 3 corresponded to the WRKY domain at the C-terminal end in sorghum. Most SbWRKY members within the same group or subgroup had a similar motif composition. Motifs 15, 12, and 20 were unique to group III. Motifs 7, 10, 11, and 18 were unique to group IIb, and motif 14 was exclusively detected in group I. All group I members had two WRKY domains except for SbWRKY19, which had only one WRKY domain at the C-terminal end. Thus, the N-terminal WRKY domain may have been lost. Motif 13 was unique to group IIe. Examples of motifs shared by different groups included motif 8 shared by group IIe and IId, and motif 5 shared by group I and IIc. Although SbWRKY6 and SbWRKY43 were with a members of group III, but were not associated with any group it contained motifs 1, 2, 6, 3, and 17 (Fig 4A). Group IIe and IId were two close subgroups in the phylogenetic tree, and motifs 8, 2, 4, 3 and 17 were frequently detected in the vast majority of the members of these two subgroups. Both subgroups had similar distribution patterns, which may be indicative of functional similarity. Some motifs occurred in only a few SbWRKY members, such as motifs 12 and 20, which were only present in SbWRKY14, SbWRKY75, and SbWRKY58.

Digital expression analysis of *SbWRKY* genes at different growth stages and in different tissues

As a preliminary survey of the potential roles of *SbWRKY* genes in sorghum growth and development, the temporal and spatial expression profiles of *SbWRKY* genes were investigated

using microarray data available from SorghumFDB. The Genevestigator platform was used for the analysis, and the results are presented as heatmaps. Microarray data for different developmental stages and tissues were obtained, and the represented sorghum accessions included R159, Fremont, Atlas, PI455230, PI152611, and AR2400 [33]. The expression data of 37 samples were used, and the different tissues included leaf, roots, shoot, stem (pith and rind), and internode. Seventy-eight of the 94 *SbWRKY* genes were represented in the microarray data. The results summarized in the expression heatmap revealed that these 78 *SbWRKY* genes were expressed differently in all tested tissues. For example, 78 genes were expressed in leaf, 76 in roots, 77 in shoot and pith, 76 in internode, and 75 in rind. Thirty-two of the 78 genes had high expression levels (**Fig 5A**). In the different tissues, the numbers of genes with high expression levels (>65% expression) were as follows: 17 in roots, six in rind, nine in pith, five in internode, four in shoot, and seven in leaf. Among the different tissues, the highest number of the highly expressed genes was observed in roots. The genes with the highest expression levels were *SbWRKY19*, *SbWRKY83*, *SbWRKY45*, *SbWRKY79*, *SbWRKY5*, *SbWRKY42*, *SbWRKY73*, *SbWRKY22*, *SbWRKY34*, *SbWRKY72*, *SbWRKY25*, and *SbWRKY70* in different tissues (**Fig 5A**). Notably, *SbWRKY72* was highly expressed in all tissues. In contrast, *SbWRKY70* expression was only detected in leaf and shoot. Clustering analysis of the *SbWRKY* expression patterns grouped together rind and pith, which is consistent with their biological features.

Since the spatiotemporal expression profiles of *SbWRKYs* may inform future functional studies, *SbWRKY* expression patterns at different growth and developmental stages (seedling, stem elongation, flowering, booting, and dough stage) were also analyzed (**Fig 5B**). *SbWRKY* genes were

expressed differently (up- or down-regulated) at all stages, and those with high expression at different stages included *SbWRKY74, SbWRKY75, SbWRKY19, SbWRKY5, SbWRKY45, SbWRKY79, SbWRKY25,* and *SbWRKY72. SbWRKY* expression was slightly higher at the seedling, flowering, and dough stages, suggesting that they may be involved in stress response at sensitive stages to improve plant tolerance (**Fig 5B**).

Hierarchical clustering of the expression patterns of 78 *SbWRKY* genes under different environmental stresses was performed by Genevestigator. Two major clusters were obtained, which basically divided the *SbWRKY* genes into two groups. The first major cluster consisted of highly expressed genes, including *SbWRKY45*, *SbWRKY79*, *SbWRKY83*, and *SbWRKY16*, under different abiotic stress conditions (**Fig 5C**). The second major cluster contained several sub-clusters of *SbWRKY* genes with different expression patterns, i.e., up- or down-regulation from -2.5 to 2.5 fold change, in response to different abiotic stress conditions (**Fig 5C**). Several genes were found to have stable expression across different tissues which can be considered as constitutive gene expression, and we can conclude that the *SbWRKY* genes were shown low expression level may work cooperatively with other proteins throught plant development.

qRT-PCR expression analysis of *SbWRKY* genes in response to drought stress

Previous studies proved that *WRKY* genes involved in response to drought stress in several crops such as maize, wheat and rice [34]. To provide some clues in regards to *SbWRKY* genes response to drought stress in sorghum. Five genes (*SbWRKY45, SbWRKY72, SbWRKY74, SbWRKY75,* and *SbWRKY79*) were selected for expression analysis, qRT-PCR was used to perform the expression analysis in the shoot of the sorghum seedlings subjected to drought stress. qRT-PCR results revealed

that the transcripts abundances of the five *SbWRKY* genes were remarkably increased under drought stress at different time points (**Fig 6**) suggesting that these genes may play an important role in response to drought stress. The relative expression of the selected *SbWRKY* genes revealed a varied level of expression under drought stress. The expression level of the genes ~~was~~ peaked at different time points. *SbWRKY75* gene exhibited the highest expression level after 6 h with fold change (89-fold) after drought stress treatment onset, with a final decline after 24h. The peak expression of *SbWRKY74* was occurred at 12 h, while *SbWRKY45* and *SbWRKY79* reached their peak at 24 h (**Fig 6**). The peak expression of *SbWRKY72* and *SbWRKY75* were at 6 h, however, the expression of *SbWRKY72* ~~was~~ increased and decreased gradually. *SbWRKY79* showed the lowest expression trend in comparison with the other tested genes. Overall, the expression pattern of the selected genes under drought stress condition suggesting that different *SbWRKY* genes may play an essential role in drought stress tolerance.

Discussion

WRKY TFs are key regulators of many processes in plants, including the response to abiotic and biotic stresses. In both model and non-model plants, considerable progress has been made towards

the identification and functional characterization of WRKY TFs, and many *WRKY* genes have been found to promote stress tolerance [34]. The completed genome assembly of sorghum made it possible to perform this genome-wide analysis of *SbWRKY* genes. A total of 94 *SbWRKY* genes were identified in *S. bicolor,* which is slightly higher than the number in other species, such as 75 in *Arabidopsis* [1], 71 in sesame [7], 79 in potato, 85 in cassava [35], and 59 in grapevine [36]. In contrast, there are fewer *WRKY* genes in sorghum than in maize, soybean, and rice [18,37,38]. The present findings in sorghum, an important cereal crop and a model plant for drought tolerance, add to the recent identification of *WRKY* genes in different plant species, including chickpea [8], Chinese jujube [39], sugar beet [], and coffee [41].

Gene duplication events affect genome expansion, family size, and the distribution of genes on chromosomes. Distinct types of duplication events, such as tandem and segmental duplication, differ in terms of the resulting number of gene copies and their distribution, and these factors are important for functional prediction. Tandem duplication of chromosome regions can give rise to a cluster of family members, with subsequent structural and functional divergence over time leading to the expansion and evolution of the gene family [42]. Our analysis identified two tandem duplications of *SbWRKY* genes, which is lower than the number reported in potato [21], rice [19], wheat [5], and *Arabidopsis* [43]. Despite this difference, tandem duplication may have played a significant role in the evolution of *SbWRKYs* in sorghum.

As established by previous findings, the presence of the conserved WRKY domain, which binds to the W-box in downstream target genes, is the most essential characteristic of the WRKY family [44,45]. The classification of *SbWRKY* genes in the present study was performed according to the

approach used in other crop species based on phylogenetic tree classification. Also, the WRKY family group and subgroup divisions described in *Arabidopsis* were adopted here: groups I, II, and III according to the number of WRKY domains and the zinc finger motif pattern, along with further subdivision of group II into subgroups IIa, IIb, IIc, IId, and IIe [1]. Briefly, group I WRKYs have two WRKY domains and the C_2H_2-type zinc finger motif, whereas group II and III have a single WRKY domain, with the C_2H_2 pattern in group II and the C_2HC pattern in group III [2]. In this study, 11, 50, and 31 *SbWRKY* genes were classified into groups I, II, and III, respectively, and two genes did not belong to any of the groups. Group II was the largest with 50 members, accounting for 53.2% of all *SbWRKYs*, and this result was consistent with the findings in sesame, *Arabidopsis*, and sugar beet [7,1,38]. Among the subgroups, IIc was the largest with 20 *SbWRKY* genes, accounting for 40% of the genes in group II (Fig. 2), which is similar to the results in sugar beet [40], *Arabidopsis* [1], and soybean [46].

While most of the *SbWRKYs* contained the conserved WRKYGQK motif, other heptapeptide variants were found in *SbWRKY11*, *SbWRKY14*, *SbWRKY23*, *SbWRKY27*, *SbWRKY33*, *SbWRKY51*, *SbWRKY52*, *SbWRKY53*, *SbWRKY71*, *SbWRKY75*, *SbWRKY77*, *SbWRKY81*, and *SbWRKY83* (Table 1). Similar variations of the heptapeptide motif were also found in potato [21]. Several examples indicate that these differences may affect the binding ability of WRKY TFs to the W-box element. For example, two soybean WRKYs with the WRKYGKK motif variant were unable to bind to the W-box element [44]. In tobacco, NtWRKY12 with the WRKYGKK motif bound to TTTTCCAC instead of the W-box consensus sequence (TTGACT/C) [47]. Therefore, further investigation is needed to dissect the preferred DNA-binding sequences associated with the different WRKYGQK-like motifs.

Group-specific patterns were detected for the conserved motifs and exon-intron structure. In other words, *SbWRKY* genes within the same group had similar exon-intron patterns. The number of introns in *SbWRKY* genes ranged from 0 to 7, which is similar to the range reported in chickpea [8]. Four *SbWRKY* genes lacked introns, indicating that intron loss may have occurred during the evolution of this gene family, and a similar result was reported in rice [38]. Aside from the exon-intron structural diversity among the groups, the variations in intron size within and among the groups of *SbWRKYs* may have resulted from duplication, inversion, and fusion events [48]. These results were similar to the findings in wheat [5], carrot [48], and cassava [35]. Overall, the diversification of the exon-intron pattern should provide important clues about the evolution of *SbWRKY* genes.

The vital roles of WRKY TFs in plant growth, development, and stress tolerance are supported by *WRKY* gene expression data from several species. As a result of the extensive studies in the model plant *Arabidopsis*, many *AtWRKYs* have been functionally characterized. Therefore, identifying the closest *Arabidopsis* homologs of individual *SbWRKYs* will provide general insight into their potential functions. For example, the highly expressed gene *SbWRKY72* is orthologous to *AtWRKY70* and *AtWRKY54* in *Arabidopsis*, which have been reported to modulate osmotic stress tolerance by regulating stomatal aperture in *Arabidopsis* [49]. Thus, *SbWRKY72* or other close homologs in sorghum may have a related function. *SbWRKY79*, *SbWRKY80*, and *SbWRKY42* are orthologous to *AtWRKY25*, *AtWRKY26*, and *AtWRKY33*, which regulate heat shock proteins and the heat-induced ethylene-dependent response [50]. The orthologs of the highly expressed gene *SbWRKY45* are *AtWRKY18*, *AtWRKY40*, and *AtWRKY60*, which are involved in the response to abscisic acid and

abiotic stress [51]. Additionally, the ortholog of *SbWRKY45* in maize is *ZmWRKY40*, which was found to confer drought resistance in transgenic *Arabidopsis* [10]. An ortholog of *SbWRKY8* in *Arabidopsis* is *AtWRKY57*, which can improve drought tolerance through elevated abscisic acid levels in *Arabidopsis* [52]. Based on the available sorghum transcriptomic data, the present analysis of *SbWRKY* genes revealed their different expression patterns at different growth stages and in different tissues. As indicated by the above examples, the known functions of *AtWRKY* orthologs together with expression profile data will guide future functional analyses of *SbWRKYs*, particularly studies focused on their roles in response to environmental stress. The qRT-PCR results provided general clues on *SbWRKYs* in response to drought stress. There are several *SbWRKYs* orthologs in other crops have been confirmed to be involved in drought stress tolerance. For example, Among the tested genes, *SbWRKY45* was the orthology gene of *ZmWRKY40* in maize which was confirmed to be involved in drought stress tolerance [10]. In addition to these genes maybe there are other *SbWRKY* genes are involved in drought stress response. Therefore, further investigation into the *SbWRKY* expression under other abiotic stress (cold, salinity and heat) is needed. Our results provided some *SbWRKY* candidate for future studies.

Conclusion

This study identified 94 *SbWRKY* genes from sorghum, and the following analyses were performed: characterization and classification, gene structure analysis, chromosome mapping, and

conserved motif analysis. The *SbWRKY* gene expression profiles indicated that *SbWRKY* genes may be important in different tissues and at different developmental stages, and several genes were found to have tissue-specific expression patterns. Additionally, several *SbWRKY* genes were highly expressed in response to environmental stresses. qRT-PCR analysis revealed that *SbWRKYs* induced by drought stress. In *Arabidopsis*, many *AtWRKYs* are known to regulate abiotic and biotic stress responses, and the available information about specific *WRKY* members will facilitate functional validation and characterization of the orthologs in sorghum. Overall, the present findings provide a foundation for future functional analyses of *SbWRKY* genes in response to abiotic and biotic stress in sorghum.

Supporting information: Detail information on sequence similarity of putative paralogous pairs provided in supplemental File 1. The SbWRKY protein sequences provided in supplementary File 2, genomic sequences in supplementary File 3, and CDS in supplementary File 4. AtWRKY protein sequences are provided in Supplementary File 5. *SbWRKY* specific primers used in qRT-PCR analysis are provided in supplementary file 6.

Author Contributions: EHB designed and performed the experiment EHB, MSH, and YG prepared the manuscript. EHB, YG, and MSH analyzed the results. ZZ and PX revised the manuscript. All authors have made substantial intellectual contributions to the work and approved it for publication.

Acknowledgements: This project was supported by the Innovation Academy for Seed Design, Chinese Academy of Sciences. The authors sincerely thank the Center for Agricultural Resources Research, Institute of Genetics and Developmental Biology (CAS) for providing the facilities.

Conflicts of Interest: The authors declare no conflicts of interest.

References

1. Eulgem T, Rushton PJ, Robatzek S, Somssich IE. The WRKY superfamily of plant transcription factors, Trends in Plant Sci, *5*(5), 2000; 199–206. https://doi.org/10.1016/S1360-1385(00)01600-9
2. Rushton PJ, Somssich IE, Ringler P, Shen QJ. WRKY transcription factors, Trends in Plant Sci, 15(5), 2010; 247–258. https://doi.org/10.1016/j.tplants.2010.02.006
3. Rinerson CI, Rabara RC, Tripathi P, Shen QJ, Rushton PJ. The evolution of WRKY transcription factors, 2015; 1–18. https://doi.org/10.1186/s12870-015-0456-y
4. Bakshi M, Oelmüller R. WRKY transcription factors, Plant Signalling and Behavior. J. Plant Signal. Behav. 2014; *9*, e27700, doi:10.4161/psb.27700.
5. Ning P, Liu C, Kang J, Lv J. Genome-wide analysis of WRKY transcription factors in wheat (Triticum aestivum L.) and differential expression under water deficit condition, PeerJ, 2017; *5*, e3232. https://doi.org/10.7717/peerj.3232
6. Liu H, Qin J, Fan H, Cheng J, Li L, Liu Z. Genome-wide identification , phylogeny and expression analyses of SCARECROW - LIKE (SCL) genes in millet (Setaria italica). Phys and Moleo Biol of Plants. 2017;. https://doi.org/10.1007/s12298-017-0455-6
7. Li D, Liu P, Yu J, Wang L, Dossa K, Zhang Y. et al, Genome-wide analysis of WRKY gene family in the sesame genome and identification of the WRKY genes involved in responses to abiotic stresses. *BMC Plant Biology*, *17*(1), 2017; 1–19. https://doi.org/10.1186/s12870-017-1099-y
8. Waqas M, Azhar MT, Rana IA, Azeem F, Ali MA, Nawaz MA, et al, Genome-wide identification and expression analyses of WRKY transcription factor family members from

chickpea (Cicer arietinum L.) reveal their role in abiotic stress-responses. Genes and Genomics, *0*(0), 2019; 0. https://doi.org/10.1007/s13258-018-00780-9

9. He H, Dong Q, Shao Y. Genome-wide survey and characterization of the WRKY gene family in Populus trichocarpa, Plant Cell Rep, 2012; 1199–1217. https://doi.org/10.1007/s00299-012-1241-0
10. Wang CT, Ru JN, Liu YW, Yang JF, Li M, Xu ZS, et al. The Maize WRKY Transcription Factor ZmWRKY40 Confers Drought Resistance in Transgenic Arabidopsis. Inter J of Mole Sci, 19(9) 2018; 2580. https://doi.org/10.3390/ijms19092580
11. Sheng Y, Yan X, Huang Y, Han Y, Zhang C, Ren Y, et al. The WRKY transcription factor, WRKY13, activates PDR8 expression to positively regulate cadmium tolerance in Arabidopsis. Plant Cell and Envir, 2018; .https://doi.org/10.1111/pce.13457
12. Cao S, Wu X, Ouyang J, Jiang L, Zhu X, Fan T, et al. WRKY12 represses GSH1 expression to negatively regulate cadmium tolerance in Arabidopsis. Plant Mol. Biol., *99*, 2019; 149–159, doi:10.1007/s11103-018-0809-7.
13. Cai R, Dai W, Zhang C, Wang Y, Wu M, Zhao Y, et al. Cheng, B, The maize WRKY transcription factor ZmWRKY17 negatively regulates salt stress tolerance in transgenic Arabidopsis plants. Planta, *246* 2017; 1215–1231, doi:10.1007/s00425-017-2766-9.
14. He GH, Xu JY, Wang YX, Liu JM, Li PS, Chen M, et al. Drought-responsive WRKY transcription factor genes TaWRKY1 and TaWRKY33 from wheat confer drought and/or heat resistance in Arabidopsis. BMC Plant Biol, *16*, 2016; 1–16, doi:10.1186/s12870-016-0806-4.

15. Gao H, Wang Y, Xu P, Zhang Z. Overexpression of a WRKY Transcription Factor TaWRKY2 Enhances Drought Stress Tolerance in Transgenic Wheat. Front. Plant Sci., *9*, 997 2018; doi:10.3389/fpls.2018.00997.

16. El-Esawi MA, Al-Ghamdi AA, Ali HM, Ahmad M. Overexpression of AtWRKY30 Transcription Factor Enhances Heat and Drought Stress Tolerance in Wheat (*Triticum aestivum* L.), Genes, *10*, 2019; 163, doi:10.3390/genes10020163.

17. Tang H, Cuevas HE, Das S, Sezen UU, Zhou C, Guo H, Goff VH, Ge Z, Clemente TE, Paterson AH. Seed shattering in a wild sorghum is conferred by a locus unrelated to domestication. Proceedings of the National Academy of Sciences,110:15824, 2013, 10.1073/pnas.1305213110.

18. Wei KF, Chen J, Chen YF, Wu LJ, Xie DX. Molecular phylogenetic and expression analysis of the complete WRKY transcription factor family in maize. *DNA Research*, *19*(2), 2012; 153–164. https://doi.org/10.1093/dnares/dsr048

19. Ramamoorthy R, Jiang SY, Kumar N, Venkatesh PN, Ramachandran S. A comprehensive transcriptional profiling of the WRKY gene family in rice under various abiotic and phytohormone treatments. Plant and Cell Physio, *49*(6), 2008; 865–879. https://doi.org/10.1093/pcp/pcn061

20. Li D, Liu P, Yu J, Wang L, Dossa K, Zhang Y, et al. Genome-wide analysis of WRKY gene family in the sesame genome and identification of the WRKY genes involved in responses to abiotic stresses. BMC Plant Biol, *17*(1), 2017; 1–19. https://doi.org/10.1186/s12870-017-1099-y

21. Zhang C, Wang D, Yang C, Kong N, Shi Z, Zhao P, et al. Genome-wide identification of the potato WRKY transcription factor family. PLoS ONE, *12*(7), 2017; 1–20. https://doi.org/10.1371/journal.pone.0181573

22. Kumar A, Reddy B, Sharma H, Hash C, Rao P, Ramiah B, et al. Recent advances in Sorghum Genetic Enhancement Research at ICRISAT. American J of plant Sci, 02(04), 2011; 589-600. doi.org/10.4236/ajps.2011.24070

23. Woldesemayat AA, Ntwasa M. Pathways and Network Based Analysis of Candidate Genes to Reveal Cross-Talk and Specificity in the Sorghum (Sorghum bicolor (L.) Moench) Responses to Drought and It's Co-occurring Stresses. *Frontiers in Genetics*, *9*(11), 2018; 1–22 https://doi.org/10.3389/fgene.2018.00557

24. Woldesemayat AA, Heusden PVan, Ndimba BK, Christoffels A. An integrated and comparative approach towards identification, characterization and functional annotation of candidate genes for drought tolerance in sorghum (Sorghum bicolor (L.) Moench), BMC Genetics, 18 (1) 2017; 1–24. https://doi.org/10.1186/s12863-017-0584-5

25. Letunic I, Bork P. 20 years of the SMART protein domain annotation resource. Nucleic Acids Research, 46(D1), 2018; 493–D496. https://doi.org/10.1093/nar/gkx922

26. Ren A, Ahmed R, Chen H, Han L, Sun J, Ding A, et al. Genome-Wide Identification, Characterization and Expression Patterns of the Pectin Methylesterase Inhibitor Genes in Sorghum bicolor, Genes, *10*(10) 2019; 755. https://doi.org/10.3390/genes10100755

27. Kumar S, Stecher G, Tamura K. MEGA7: Molecular Evolutionary Genetics Analysis Version 7.0 for Bigger Datasets. Molec Biol and Evolution, *33*(7), 2016; 1870–1874. https://doi.org/10.1093/molbev/msw054

28. Hu B, Jin J, Guo AY, Zhang H, Luo J, Gao G. GSDS 2.0: An upgraded gene feature visualization server. Bioinformatics, *31*(8), 2015; 1296–1297. https://doi.org/10.1093/bioinformatics/btu817

29. Zou Z, Yang L, Wang D, Huang Q, Mo Y, Xie G. Gene structures, evolution and transcriptional profiling of the WRKY gene family in castor bean (Ricinus communis L.). PLoS ONE, 11(2), 2016; 1–23. https://doi.org/10.1371/journal.pone.0148243

30. Gernnam AK. Genevestigator Impact Facilitating Analysis. plant physio, 141(4), 2006; 1164–1166. https://doi.org/10.1104/pp.104.900198.1164

31. Livak KJ, Schmittgen TD, Analysis of relative gene expression data using real-time quantitative PCR and the $2^{-\Delta\Delta Ct}$ method. Elsevier Science, 25: 2001, 402-408, doi:101006/meth.2001.1262

32. Cheng Y, Yao ZP, Ruan MY, Ye QJ, Wang RQ, Zhou GZ. In silico identification and characterization of the WRKY gene superfamily in pepper (Capsicum annuum L .), Genet Mol Res,15(3), 2016 1–12.

33. Shakoor N, Nair R, Crasta O, Morris G, Feltus A, Kresovich S, A Sorghum bicolor expression atlas reveals dynamic genotype-specific expression profiles for vegetative tissues of grain, sweet and bioenergy sorghums, BMC Plant Biol, *14*(1) 2014; https://doi.org/10.1186/1471-2229-14-35

34. Baillo EH, Kimotho RJ, Zhang ZB, Xu P. Transcription Factors Associated with Abiotic and Biotic Stress Tolerance and Their Potential for Crops Improvement, Genes, *10*(10), 2019; 771. https://doi.org/10.3390/genes10100771

35. Wei Y, Shi H, Xia Z, Tie W, Ding Z, Yan Y, et al. Genome-Wide Identification and Expression Analysis of the WRKY Gene Family in Cassava. Front in Plant Sci, *7*(2), 2016; 1–18. https://doi.org/10.3389/fpls.2016.00025

36. Wang M, Vannozzi A, Wang G, Liang YH, Tornielli GB, Zenoni S, et al, Genome and transcriptome analysis of the grapevine (Vitis vinifera L.) WRKY gene family. Horticulture Research, 1(2), 1 2014; 4016. https://doi.org/10.1038/hortres.2014.16

37. Yu Y, Wang N, Hu R, Xiang F. Genome-wide identification of soybean WRKY transcription factors in response to salt stress. SpringerPlus. 5 2016; 920 https://doi.org/10.1186/s40064-016-2647-x

38. Ross CA, Liu Y, Shen QJ. The WRKY Gene family in Rice (*Oryza sativa*), J of integr Pl Biol, 49(6), 2007; 827–842. https://doi.org/10.1111/j.1672-9072.2007.00504.x

39. Chen X, Chen R, Wang Y, Wu C, Huang J. Genome-Wide Identification of WRKY Transcription Factors in Chinese jujube (Ziziphus jujuba Mill.) and Their Involvement in Fruit Developing, Ripening, and Abiotic Stress. genes, 10, 2019; 360; doi: 10.3390/genes10050360

40. Wu G, Li Z. W, Cao H, Wang J. Genome-wide identification and expression analysis of the WRKY genes in sugar beet (Beta vulgaris L .) under alkaline stress, PeerJ, 18 (6) 2019; https://doi.org/10.7287/peerj.preprints.27810v1.

41. Dong X, Yang Y, Zhang Z, Xiao Z, Bai X, Gao J, Genome-Wide Identification of WRKY Genes and Their Response to Cold Stress in Coffea canephora, Forests, 10 2019; 335 1–15. doi: 10.3390/f10040335.

42. Grassi ADe, Lanave C, Saccone C. Genome duplication and gene-family evolution : The case of three OXPHOS gene families, BMC Evol Biol, *421*, 2008; 1–6. https://doi.org/10.1016/j.gene.2008.05.011

43. Cannon SB, Mitra A, Baumgarten A, Young ND, May G. The roles of segmental and tandem gene duplication in the evolution of large gene families in Arabidopsis thaliana, BMC plant Biol, *21* 2004; 1–21. https://doi.org/10.1186/1471-2229-4-10

44. Bi C, Xu Y, Ye Q, Yin T, Ye N. Genome-wide identification and characterization of WRKY gene family in *Salix suchowensis*. PeerJ, 4, 2016; e2437. https://doi.org/10.7717/peerj.2437

45. Ding M, Chen J, Jiang Y, Lin L, Cao Y, Wang M. Genome-wide investigation and transcriptome analysis of the WRKY gene family in Gossypium, Mol Genet Genomics, 5 (9) 2014; 4-7 https://doi.org/10.1007/s00438-014-0904-7

46. Luo X, Bai X, Sun X, Zhu D, Liu B, Ji W, et al. Expression of wild soybean WRKY20 in Arabidopsis enhances drought tolerance and regulates ABA signalling, J of Exp Bot, 6 (12) 2013; https://doi.org/10.1093/jxb/ert073

47. Verk Van MC, Pappaioannou D, Neeleman L, Bol JF, Linthorst HJM. A Novel WRKY Transcription Factor Is Required for Induction of PR-1a Gene Expression by Salicylic Acid, plant physiol, *146*(4), 2008; 1983–1995. https://doi.org/10.1104/pp.107.112789

48. Li MY, Xu ZS, Tian C, Huang Y, Wang F, Xiong AS. Genomic identification of WRKY transcription factors in carrot (Daucus carota) and analysis of evolution and homologous groups for plants, Scientific Reports, *6*(7) 2016; 1–17. https://doi.org/10.1038/srep23101

49. Li J, Besseau S, Petri T. Defense-related transcription factors WRKY70 and WRKY54 modulate osmotic stress tolerance by regulating stomatal aperture in Arabidopsis, New Phytol, 4 2013; 57–472. https://doi: 10.1111/nph.12378.

50. Li S, Fu Q, Chen L. Arabidopsis thaliana WRKY25, WRKY26, and WRKY33 coordinate induction of plant thermotolerance, Planta, 1 2011; 237–1252. https://doi.org/10.1007/s00425-011-1375-2

51. Chen H, Lai Z, Shi J, Xiao Y, Chen Z, Xu X. Roles of arabidopsis WRKY18, WRKY40 and WRKY60 transcription factors in plant responses to abscisic acid and abiotic stress, BMC Plant Biology, *10*(1) 2010; 281. https://doi.org/10.1186/1471-2229-10-281

52. Jiang Y, Liang G, Yu D. Activated expression of WRKY57 confers drought tolerance in arabidopsis, Molecular Plant, *5*(6), 1 2012; 375–1388. https://doi.org/10.1093/mp/sss080

Click here to access/download;Figure;Figure 2.tiff

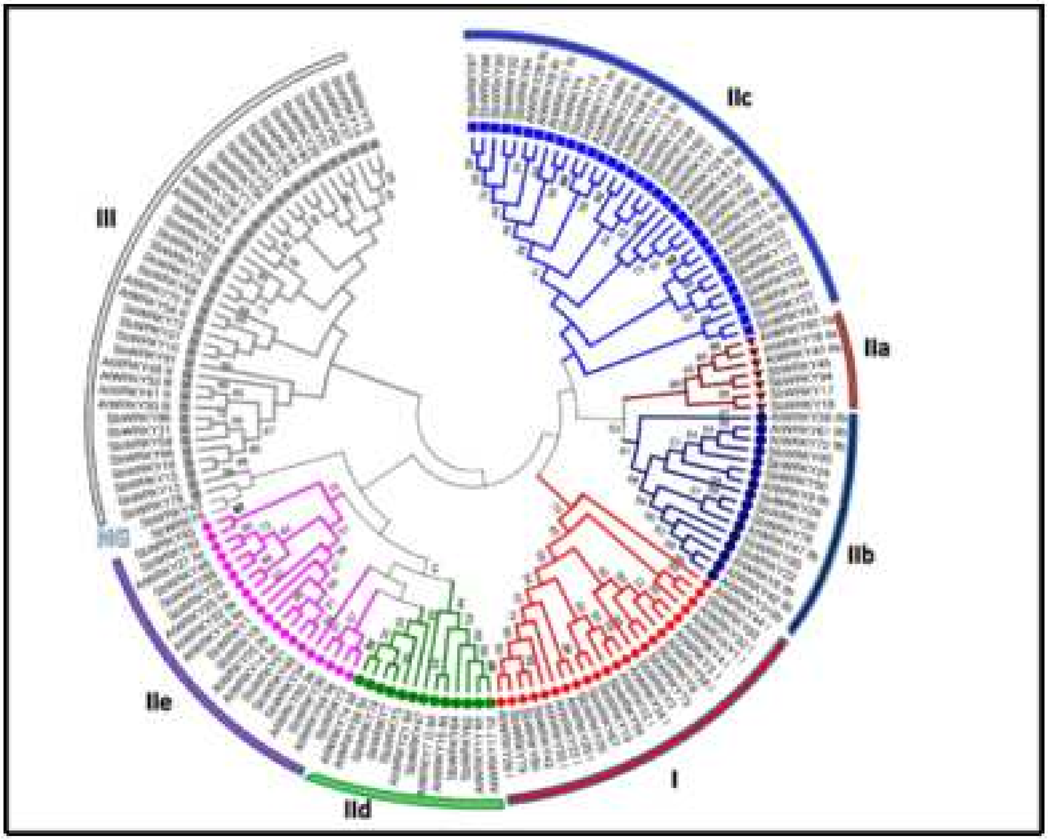

Figure 2. Phylogenetic tree showing the relationships among WRKY members in soybean and *Arabidopsis*. *GmWRKY* and *AtWRKY* amino-acid sequences were aligned with ClustalW, and a phylogenetic tree was constructed with MEGA7.0 using the neighbor-joining method and 1000 bootstrap replication. The members were divided into groups I, II, and III, and group II was further divided into subgroups IIa, IIb, IIc, IId, and IIe.

Click here to access/download;Figure;Figure 3.tiff

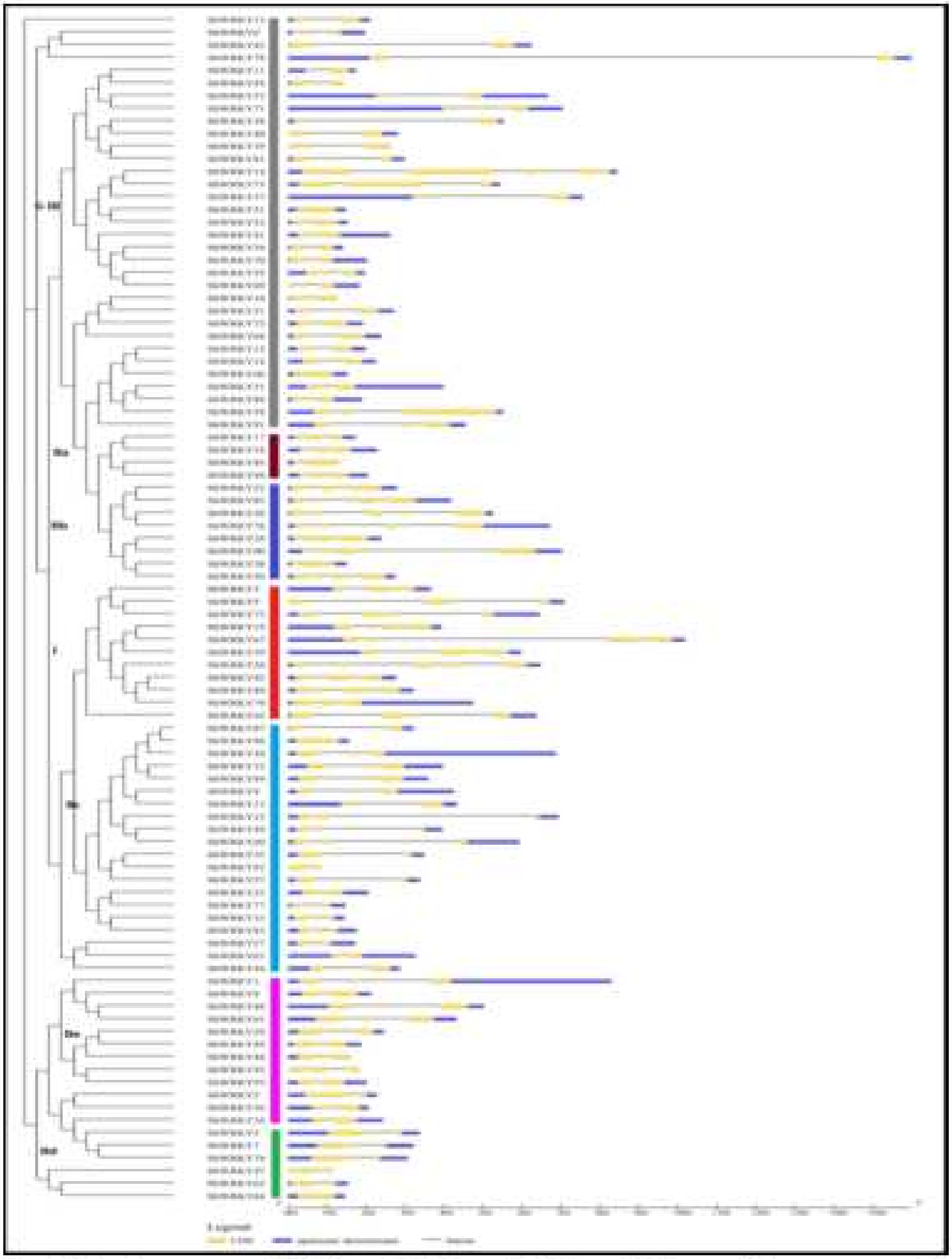

Figure 3. Exon-intron structures of SiWRKY genes. Gene Structure Display Server (GSDS) was used for gene structure analysis, and the phylogenetic tree was constructed using MEGA7.0. In the gene diagrams, blue bars indicate upstream and downstream UTRs, yellow bars indicate coding sequences (CDS), and the black lines indicate introns.

Click here to access/download;Figure;Figure 4.tiff

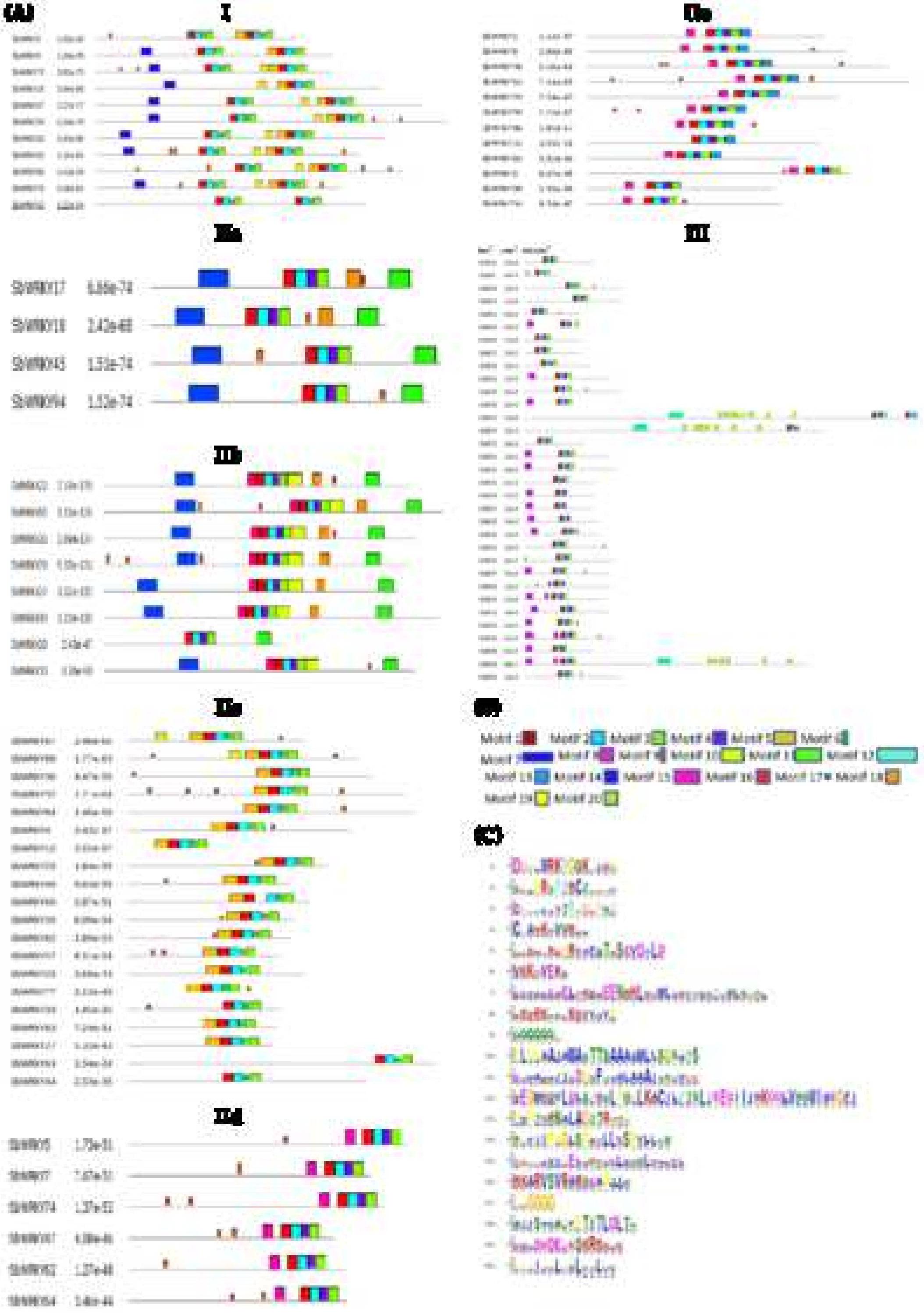

(A) The distribution of 20 conserved motifs identified by MEME in the different groups of SlWRKYs. (B) Each motif is indicated by a different color. (C) Sequence logos for motifs 1-20.

Click here to access/download;Figure;Figure 5C.tiff

(C)

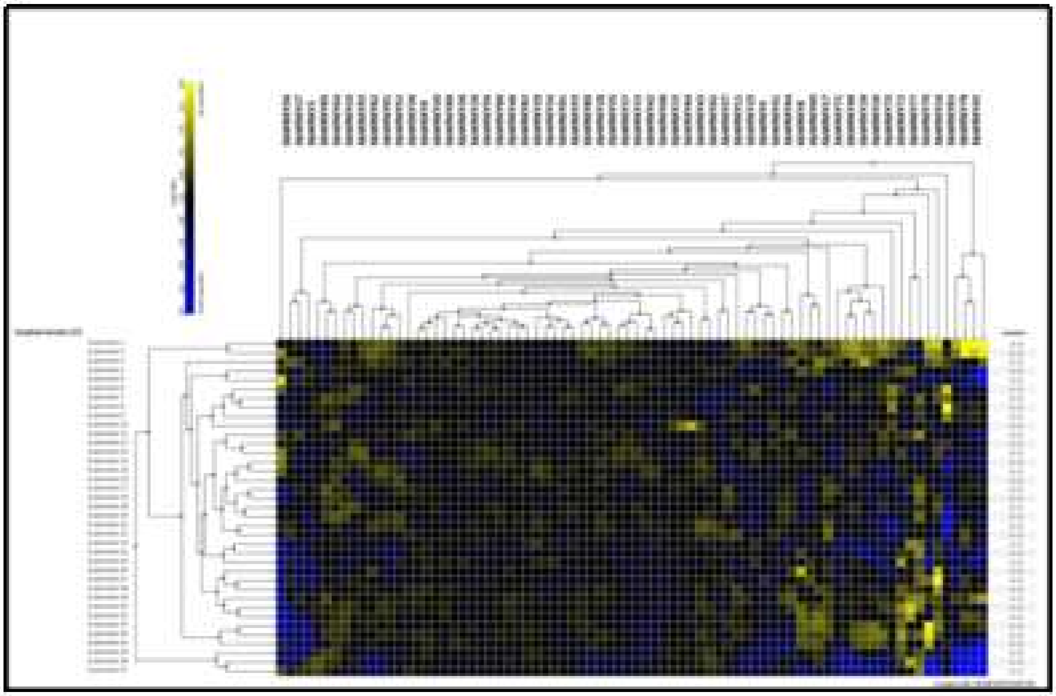

Figure 5. Heatmaps of [illegible]WRKY gene expression levels (A) in different tissues and (B) at different growth stages. (C) Hierarchical clustering of [illegible]WRKY gene expression patterns under different environmental conditions.

Click here to access/download;Figure;Figure 6.tiff

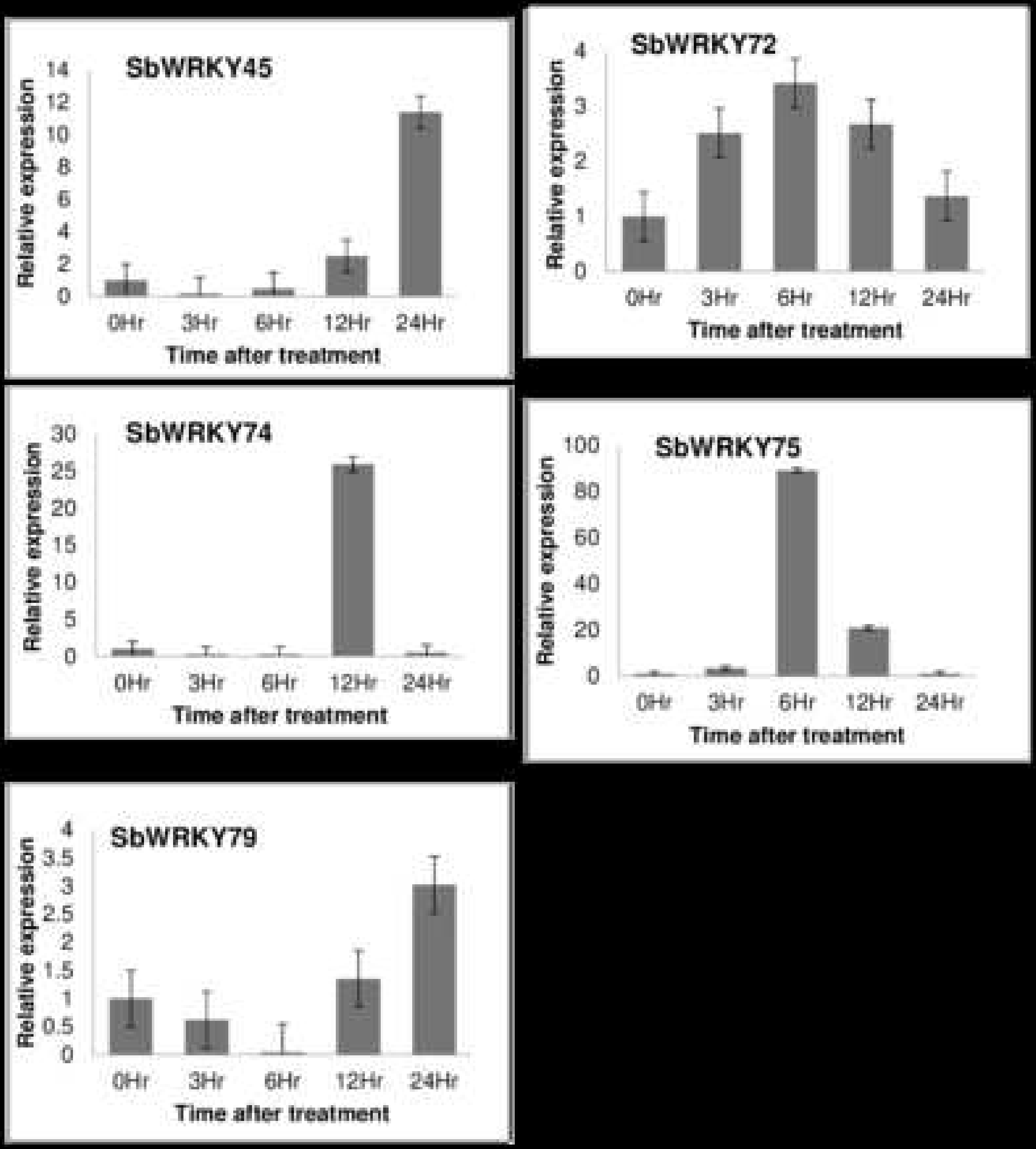

Click here to access/download;Figure;Figure-5 A and B.tiff

(A)

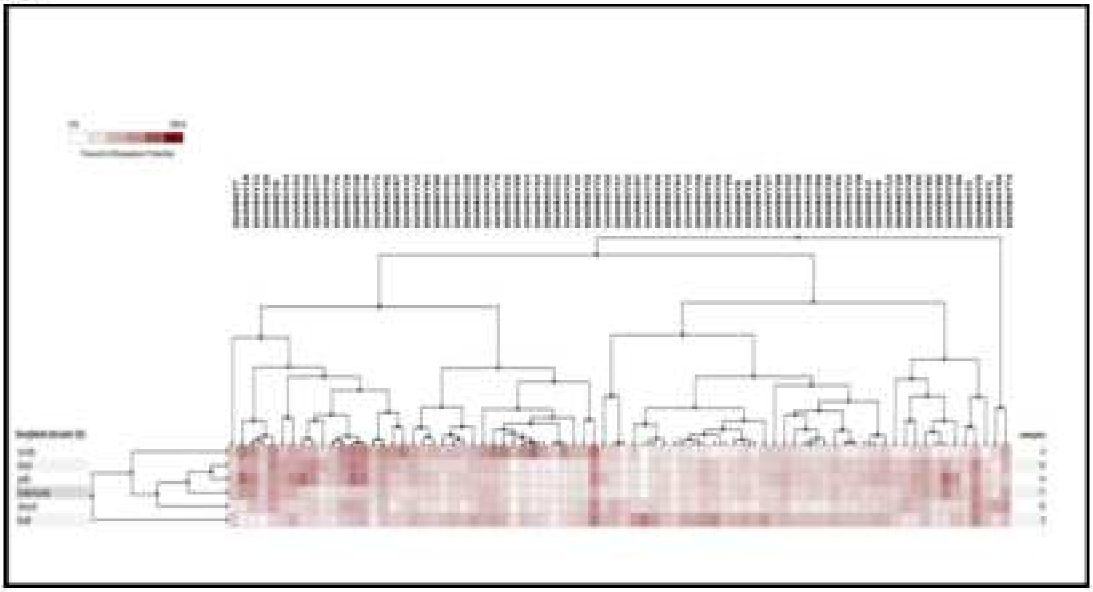

(B)

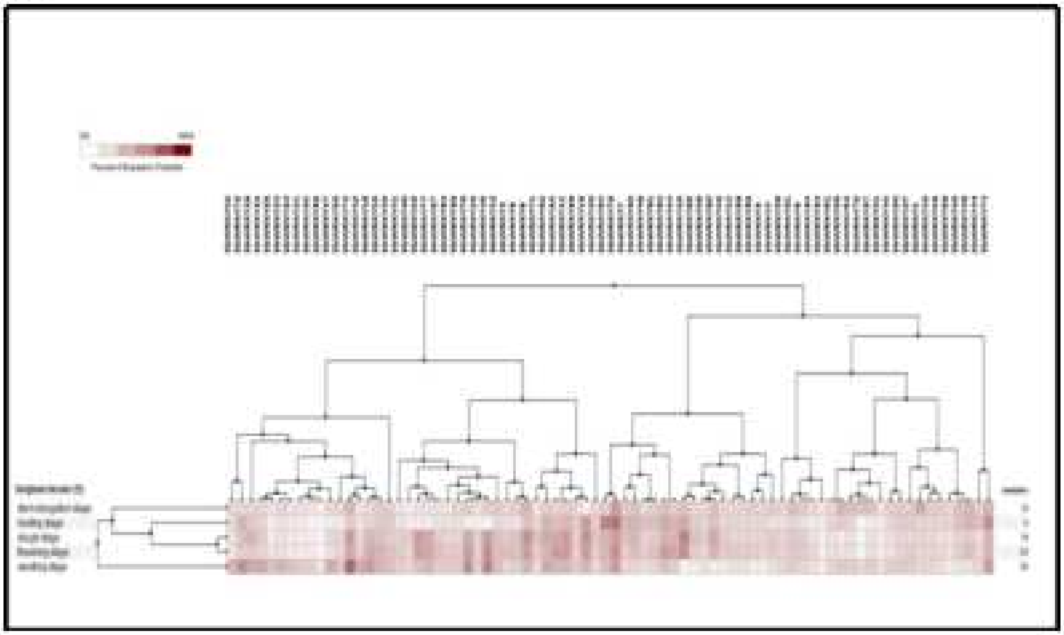

Click here to access/download;Figure;Figure 1.tiff

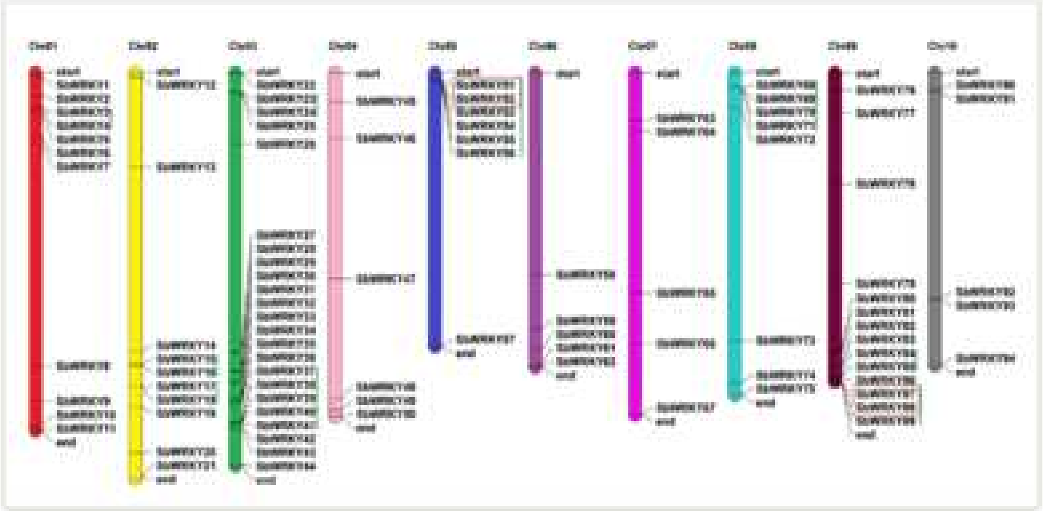

Figure 1. Distribution of 94 *SbWRKY* genes on sorghum chromosomes. Chr01-Chr10 above the colored bars indicate the chromosome (Chr) number. The physical location of each *SbWRKY* gene is shown, and the gene name is indicated on the right side of each bar as *SbWRKYX*. The red boxes indicate tandem duplications, and the green lines denote gene clusters.

Click here to access/download
Supporting Information - Compressed/ZIP File Archive
Supporting information.zip
